# Action Mechanism Underlying Improvement Effect of Fuzi Lizhong Decoction on Nonalcoholic Fatty Liver Disease: A Study Based on Network Pharmacology and Molecular Docking

**DOI:** 10.1155/2022/1670014

**Published:** 2022-01-20

**Authors:** Zheng Luo, Lu-Yun Xia, Yu-Qin Tang, Lili Huang, Dan Liu, Wen-Ying Huai, Chun-Jiang Zhang, Yan-Qiu Wang, Yong-Mei Xie, Qiao-Zhi Yin, Yun-Hui Chen, Tian-E. Zhang

**Affiliations:** ^1^College of Basic Medicine, Chengdu University of Traditional Chinese Medicine, Chengdu, China; ^2^West China Hospital, Sichuan University, Chengdu, China; ^3^Key Biology Laboratory for TCM Viscera-Manifestation Research of Sichuan University, Chinese Medical Center of Chengdu University of TCM, Chengdu, China

## Abstract

**Objective:**

This study aimed to decipher the bioactive compounds and potential mechanism of traditional Chinese medicine (TCM) formula Fuzi Lizhong Decoction (FLD) for nonalcoholic fatty liver disease (NAFLD) treatment via an integrative network pharmacology approach.

**Methods:**

The candidate compounds of FLD and its relative targets were obtained from the TCMSP and PharmMapper web server, and the intersection genes for NAFLD were discerned using OMIM, GeneCards, and DisGeNET. Then, the PPI and component-target-pathway networks were constructed. Moreover, GO enrichment and KEGG pathway analysis were performed to investigate the potential signaling pathways associated with FLD's effect on NAFLD. Eventually, molecular docking simulation was carried out to validate the binding affinity between potential core components and key targets.

**Results:**

A total of 143 candidate active compounds and 129 relative drug targets were obtained, in which 61 targets were overlapped with NAFLD. The PPI network analysis identified ALB, MAPK1, CASP3, MARK8, and AR as key targets, mainly focusing on cellular response to organic cyclic compound, steroid metabolic process, and response to steroid hormone in the biological processes. The KEGG pathway analysis demonstrated that 16 signaling pathways were closely correlated with FLD's effect on NALFD with cancer pathways, Th17 cell differentiation, and IL-17 signaling pathways as the most significant ones. In addition, the molecular docking analysis revealed that the core active compounds of FLD, such as 3′-methoxyglabridin, chrysanthemaxanthin, and Gancaonin H, had a high binding activity with such key targets as ALB, MAPK1, and CASP3.

**Conclusions:**

This study suggested that FLD exerted its effect on NAFLD via modulating multitargets with multicompounds through multipathways. It also demonstrated that the network pharmacology-based approach might provide insights for understanding the interrelationship between complex diseases and interventions of the TCM formula.

## 1. Introduction

Nonalcoholic fatty liver disease (NAFLD) is a metabolic disorder with excessive hepatic fat deposition in the absence of significant alcoholic consumption, application of susceptible medication, or other preexisting liver conditions [1]. Epidemiological surveys indicate that its global prevalence reaches approximately 25%, with an increasing annual incidence [2]. As the most common chronic liver disease, NAFLD encompasses a broad spectrum of liver damage and has been considered a cause of end-stage liver disease. It is assumed to be associated with increased rates of hepatocellular carcinoma and death and is projected to become the leading cause of cirrhosis that requires liver transplantation within the next ten years [3]. This progression is potentially reversible with proper management. Although insulin resistance, metabolic complications, and genetic variants, including PNPLA3 and TM6SF2, may play a role [4], the pathogenesis of NAFLD remains incompletely decoded, and no pharmacological interventions have been officially approved. Furthermore, the treatment options available for NAFLD patients are still limited and mainly based on nutrition and exercise [5]. Therefore, there is an urgent need for the research and development of novel medical interventions.

Traditional Chinese medicine (TCM) has a long history of treating liver disease in China, and compelling lines of preclinical and clinical evidence have demonstrated that the TCM formula may yield effective outcomes for the management of NAFLD. Fuzi Lizhong Decoction (FLD) is a classic TCM formula from Volume 2 of *San Yin Ji Yi Bing Zheng Fang Lun* (*Treatise on Diseases, Patterns, and Formulas Related to the Unification of the Three Etiologies*, 1174 A.D.) written by Dr. Wu-ze Chen. It has been widely utilized to treat NAFLD clinically. Previous studies have shown that FLD can promote the proliferation and inhibit the apoptosis of NAFLD cells via upregulating the expression of cyclin-associated and antiapoptotic proteins and downregulating the expression of proapoptotic proteins and apoptotic actors [6]. It can also effectively reduce blood lipid content, improve liver function, and decrease liver index, thus alleviating the progression of NAFLD in rats. In addition, its pharmacological effects are closely related to activating AMPK, inhibiting the liver fat synthesis, and inhibiting the NF-*κ*B signaling pathway and the secretion of inflammatory response factors TNF-*α* and IL-6 in the liver [7]. Moreover, it substantially reduces the fat deposition capacity of NAFLD cells and alleviates the inflammatory response [8]. These studies partially decipher the molecular mechanism of FLD's action on NAFLD. However, TCM formulas often contain complex constituents with multiple pharmacological activities at a variety of targets. From this perspective, the action mechanism of FLD on NAFLD may involve a complex of multicomponents, multitargets, and multipathways and requires a further study following the holistic strategy.

**Figure 1:**
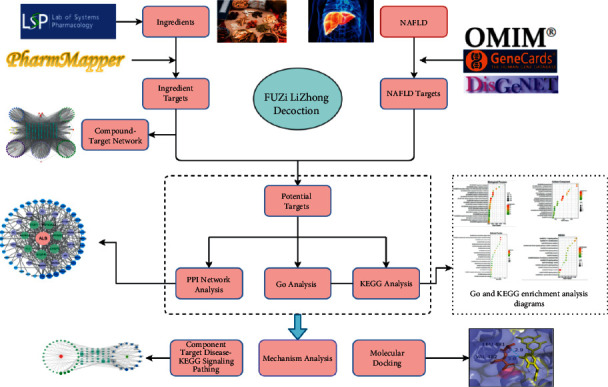
Detailed flowchart of the study design.

Network pharmacology integrates systematic medicine and bioinformation science and is consistent with the holistic concept of TCM treatment. It systematically decodes the therapeutic effects of complex TCM formula on disease via analyzing the interactions between herbal components, targets, diseases, and pathways. Hence, it has been considered as a frontier in the field of drug research and development. A growing body of research has currently verified its potential in investigating the possible molecular mechanisms of the TCM formula [9]. In this study, network pharmacology was utilized to reveal the components, targets, and pathways interaction relationships between FLD intervention and NAFLD management: (1) the candidate compounds and intersection targets were obtained; (2) the protein-protein interaction (PPI) and component-target-pathway-disease networks were constructed; (3) Gene Ontology (GO) and Kyoto Encyclopedia of Genes and Genomes (KEGG) analysis were conducted; and (4) molecular docking simulation was performed to validate the binding affinity between potential core components and key targets. The detailed flowchart of the study design is shown in Figure 1.

## 2. Material and Methods

### 2.1. Collection of FLD Active Components and Corresponding Targets

FLD comprises five herbs, namely FuZi (Radix Saposhnikoviae divaricate, FZ), GanJiang (Rhizoma Zingiberis, GJ), RenShen (Panax ginseng, RS), BaiZhu (Rhizoma Atractylodis Macrocephalae, BZ), and GanCao (Radix et Rhizoma Glycyrrhizae, GC). The active compounds of FLD were extracted from the TCMSP data platform (https://tcmspw.com/tcmsp.php) [10], which contains 499 Chinese herbs registered in *Chines Pharmacopoeia* (2010 edition) with 29,384 ingredients, 3,311targets, and 837 related diseases. The thresholds further filtering the obtained active compounds were set as Oral bioavailability (OB) ≥ 30% and drug-likeness (DL) ≥ 0.18. The protein targets of the collected active compounds were screened with a standard of “Norm Fit≥0.90” using the PharmMapper web server (http://www.msftconnecttest.com/redirect) [11]. The protein targets corresponding to the active components of herbs were imported into the Uniport data platform (https://www.uniprot.org/) to acquire the gene names, IDs, and functions, respectively [12].

### 2.2. Construction of FLD-Compound-Target Network

The FLD-active compound-target network was constructed and visualized using Cytoscape 3.8.2 software [13], and the core active ingredients of FLD were identified.

### 2.3. Acquisition of NAFLD-Associated Targets and Candidate Genes

Keywords such as “nonacholic fat liver disease” or “NAFLD” were employed to search the disease-related targets from the Online Mendelian Inheritance in Man (OMIM) (http://www.omim.org), Human Gene Database (GeneCards) (https://www.genecards.org), and DisGeNET (http://www.disgenet.org/) databases. After removing the duplicate targets, the protein targets related to NAFLD were imported to the Uniprot data platform. Then, the acquired genes were input to draw Venn diagrams using Venny2.1(https://bioinfogp.cnb.csic.es/tools/venny/), and the intersection genes were obtained as candidate genes.

### 2.4. Construction of PPI Network

The candidate genes were imported into the Search Tool for the Retrieval of Interacting Genes online analysis software (STRING, https://www.string-db.org/) to construct a PPI network. The visual network graphs were created by Cytoscape 3.8.2 software. Degree, betweenness, and closeness were three major topological parameters used to identify the key genes.

### 2.5. GO and KEGG Pathway Enrichment Analysis

The functional annotation of GO and KEGG pathways was performed using the Metascape platform (http://metascape.org/gp/index.html) to explore the relevant biological processes (BPs), cellular components (CCs), molecular functions (MFs), and signal pathways of potential anti-NAFLD targets. The results were saved and sorted with the False Discovery Rate algorithm for each term. The bioinformatics platform (http://www.bioinformatics.com.cn/) was applied to visualize GO and KEGG enrichment results by bubble diagrams.

### 2.6. Construction of Compound-Target-Pathway Network Construction

To further investigate the therapeutic mechanisms of FLD for NAFLD, the compound-target-pathway (C-T-P) network was constructed using Cytoscape 3.8.2. In this network, the compounds, target genes, and pathways are symbolized with nodes in different colors and shapes, and the association between nodes is represented by the edge.

### 2.7. Molecular Docking Simulation

A molecular docking simulation was conducted to assess the binding energy of the core compounds with the key targets. Autodock Vina 1.5.6 software developed by Olson's research group in Scripps Research Institute was adopted to assess molecular docking [14]. The top three targets of the PPI network and the top ten core compounds of FLD were selected, and their chemical structures were downloaded from the RCSB PDB database (http://www.rcsb.org/) and TCMSP data platform, respectively. The AutoDockTools were utilized to convert the candidate compounds into PDB format. The proteins were virtually dehydrated and hydrogenated, and the core compounds were hydrogenated. The original ligands were extracted and stored separately. AutoDockTool was utilized to convert compounds, ligands, and proteins into the “pdbqt” format and to define if the location of each protein or its ligands was the active pocket of the protein. When a binding energy value < 0, the molecular proteins were considered spontaneously binding and interacting. Accordingly, the lower the binding energy required for docking, the more stable the molecular conformation.

## 3. Results

### 3.1. Active Ingredients and Target Genes

Initially, 738 compounds were retrieved from the five herbs in FLD. Among them, 65, 55, 190, 148, and 280 compounds were from FZ, BZ, RS, GJ, and GC, respectively. A total of 147 active compounds were acquired upon OB ≥ 30% and DL ≥ 0.18. Specifically, 21, 7, 22, 5, and 92 compounds were from FZ, BZ, RS, GJ, and GC, respectively. In addition, some compounds were overlapped across different herbs, including sitosterol (ID: MOL000359) in FZ, GJ, and GC, beta-sitosterol (ID: MOL000358) in GJ and RS, and kaempferol (ID: MOL000422) in RS and GC. A total of 143 active compounds were identified after deleting duplicated entries. After the protein targets corresponding to the active components of herbs were imported into the Uniport data platform to acquire the gene names and eliminate the duplications, 129 gene targets with those putative components were acquired. Among which, 45 were associated with FZ, 7 with BZ, 30 with RS, 4 with GJ, and 43 with GC. The information of each candidate compound is shown in Table 1.

### 3.2. FLD-Compound-Target Network

**Figure 2:**
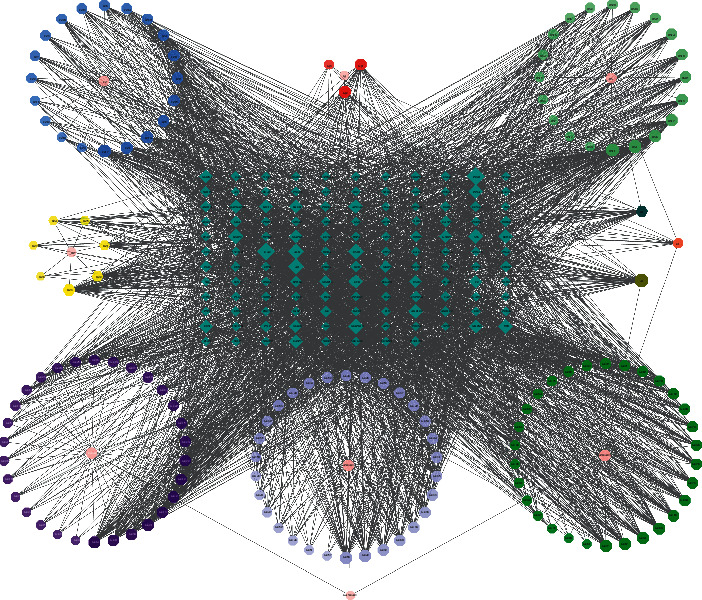
Compound-target network: pink circles represent the herbs in FLD; hexagons represent active compounds of each herb, and A1, B1, and C1 hexagons correspond to active compounds shared by different herbs; blue diamonds represent related targets (the IDs of the components are presented in Table 1).

The FLD-C-T network contained 280 nodes (143 nodes for compound, 129 nodes for target, and 8 nodes for herbs) and 3,199 edges (Figure 2). Sitosterol showed the highest degree of connectivity in the network with 57 targets, followed by beta-sitosterol with 44 targets, and ginsenoside rh2 with 35 targets. The properties of this network were suitable for displaying complex compounds, multiple targets, and interactions between compounds and targets. The OB of the three compounds mentioned above was 36.91%, 36.91%, and 36.32%, respectively, indicating they were potential key active compounds. Detailed information about the compound-target network is presented in Supplement Table 1.

### 3.3. NAFLD-Associated Targets and Candidate Genes

**Figure 3:**
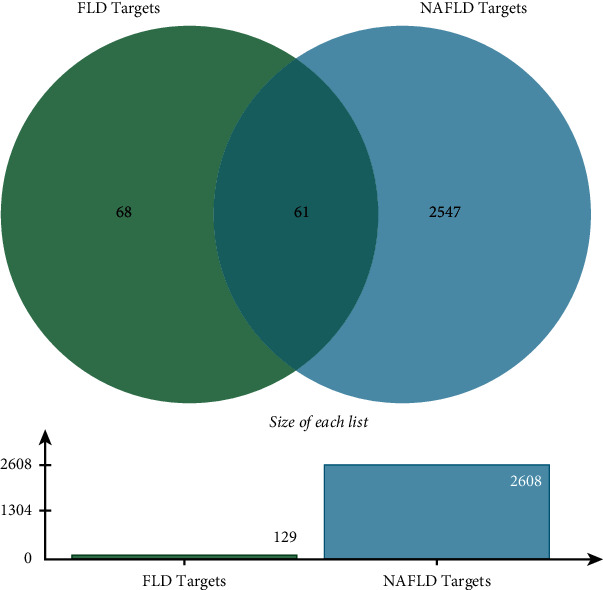
Venn's diagram of intersection targets of FLD and NAFLD.

A total of 3,181 targets for NAFLD were integrated from multiple databases, including 512 targets from OMIM, 1,611 from GeneCards, and 1,058 from DisGeNET. The final list of 2,608 NAFLD-related targets was obtained after eliminating duplicates (Supplement Table 2). Among them, 61 intersection targets between FLD and NAFLD were identified and collected for further mechanism investigation (Figure 3 and Supplement Table 3).

### 3.4. PPI Network of FLD and NAFLD Targets Construction

**Figure 4:**
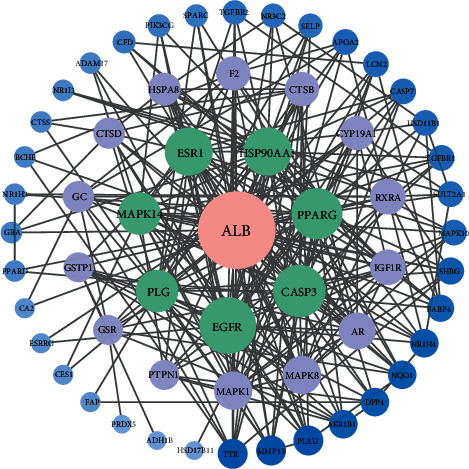
PPI network of FLD for NAFLD treatment. Each node represents a protein target, and each edge symbolizes the interaction between two nodes. The PPI network diagram is arranged according to the df. The greater the significance, the more central the node is.

A visualized PPI network composed of 61 nodes representing proteins with 277 edges representing the interactions between proteins was constructed (Figure 4 and Supplement Table 4). The average node degree value of the PPT network was 9.08, and the average local clustering coefficient was 0.604. The genes with higher values of degree, betweenness, and closeness above the median were acquired as the key targets of FLD for NAFLD. Consequently, the targets including ALB, MAPK1, CASP3, MARK8, AR, HSP90AA1, and EGFR were defined as key genes.

### 3.5. GO and KEGG Pathway Enrichment Analysis

**Figure 5:**
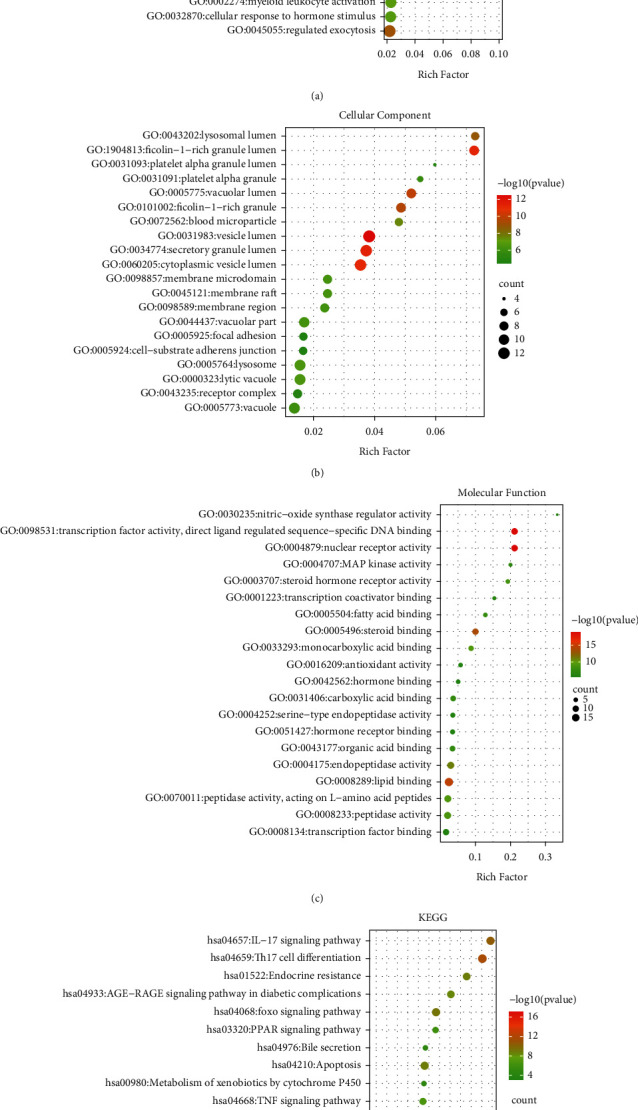
The 20 most significant of GO analysis (a) for BP, (b) for CC and (c) for MF and pathway enrichment (d) KEGG analysis of therapeutic target genes of FLD on NFALD.

To further explore the biological functions of the 61 target genes of FLD for NAFLD, GO enrichment analysis was performed based on BP, CC, and MF and yielded 722 entries. BP enrichment analysis provided 617 entries, and the top 20 most enriched terms were presented in a bubble chart with cellular response to organic cyclic compound, steroid metabolic process, and response to steroid hormone as the top three (Figure 5(a)). CC analysis obtained 44 entries primarily involving the vesicle lumen, secretory granule lumen, and ficolin-1-rich granule lumen (Figure 5(b)). MF enrichment analysis revealed 61 entries with nuclear receptor activity, transcription factor activity, direct ligand regulated sequence-specific DNA binding, and lipid binding at the top ones (Figure 5(c)). To further investigate the biological processes of these targets, the KEGG pathway analysis was conducted and yielded 181 items (*p*<0.01), and the 16 pathways associated with NAFLD were displayed in Figure 5(d) with pathways in cancer, Th17 cell differentiation, and IL-17 signaling pathway as the top ones, indicating they might be essential pathways of FLD for NAFLD treatment and are worthy of further study. The pathway results were also intensively enriched in cancer, immune, bile secretion, substance metabolism, and apoptosis-related pathways. The detailed information of functional analysis is presented in Supplement Table 5.

### 3.6. Compound-Target-Pathway Network

**Figure 6:**
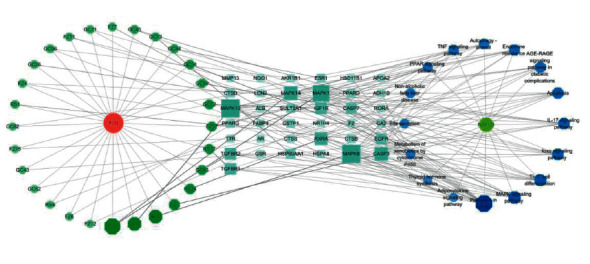
Component-target-pathway network. Green represents active ingredients of FLD; blue represents targets; dark blue represents signaling pathways of NAFLD.

The compound-target-pathway network contained 83 nodes (including 28 for compounds, 37 for targets, and 16 for pathways) and 268 edges (Figure 6). Analysis on the network revealed that multiple components from FLD targeted at least one gene, and formononetin (GC11) was considered the most potent compound that interacted with 17 genes. Most genes were regulated by at least two active compounds, and at least five genes were potentially involved in each pathway related to NAFLD. Moreover, MAPK8 and MAPK10 had the highest volume, followed by MAPK1, MAPK14, CASP3, and TGFBR1. The results revealed that FLD might exert therapeutic effects on NAFLD via modulating multiple targets and pathways with multiple compounds.

### 3.7. Molecular Docking

**Figure 7:**
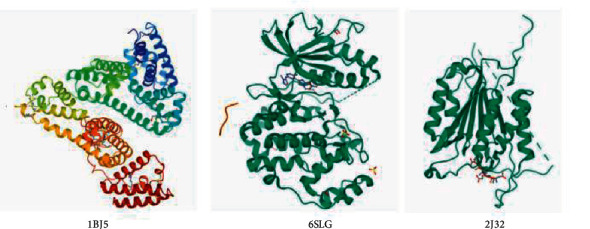
3D structure diagram of the top three target proteins.

**Figure 8:**
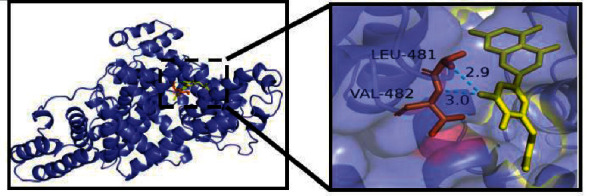
Molecular docking of 3′-methoxyglabridin and ALB. The dashed blue lines represent hydrogen bonds.

The docking of the top ten active compounds obtained by the C-T network analysis with three potential core targets, CASP3, ALB, and MAPK1 acquired from the PPT network analysis, was performed. The information of compounds and targets is shown in Table 2 and Figure 7. The binding energies are presented in Table 3. A binding energy value less than 0 indicates that the ligand molecules can spontaneously bind to the receptor protein, while a value less than −5.0 kJ ·mol-1 suggests that the ligand molecules have a desirable binding affinity [15]. The results of the molecular docking analysis revealed that all compound-target pairs are lower than 0, indicating that every core compound has a good binding affinity to the top three targets. Among them, the 3′-methoxyglabridin and ALB presented the tightest binding energy (−9.5) with the LEU-481 and VAL-482 as active sites for hydrogen bond interaction (Figure 8). The docking prediction might provide a preliminary foundation for further investigation of drug targets.

## 4. Discussion

The treatment of NAFLD remains a major public health challenge globally. Increasing lines of evidence indicate that FLD can treat NALFD effectively, but its mechanism of action remains obscure, especially from a perspective of holistic review. Network pharmacology can reveal the interaction relationships between multicompounds, multitargets, and multipathways of complex formula and therefore and therefore may play a guiding role in drug research and development [16]. Moreover, it is consistent with the holistic view of TCM. Therefore, this study utilized the network pharmacology approach with molecular docking to explore the potential molecular mechanism of FLZ's therapeutic effect on NAFLD to provide further insight.

In this study, 143 active compounds and 61 relative intersection targets were acquired. The topological analysis on the C-T network indicated the core compounds included sitosterol, beta-sitosterol, and ginsenoside rh2. Intriguingly, previous studies have shown that both sitosterol and beta-sitosterol can reduce hepatic lipid accumulation and regulate the hepatic lipid metabolism of NAFLD. In addition, sitosterol has been reported to reduce inflammation caused by FLD [17, 18]. Ginsenoside rh2 can reduce lipid deposition and improve glucose tolerance [19]. These previous investigations may imply that FLD is prone to exert therapeutic effects on NAFLD with the compound of sitosterol, beta-sitosterol, and ginsenoside rh2, which may provide a new idea and direction for further study.

The PPI network was constructed with 61 potential intersection targets. The results indicated that the active compounds in FLD might play pivotal roles in anti-NAFLD through the core targets such as albumin (ALB), mitogen-activated protein kinase1 (MAPKI), caspase-3 (CASP3), mitogen-activated protein kinase 8 (MAPK8), and androgen receptor (AR). ALB binding function has been reported as a novel biomarker to evaluate early liver damage and disease progression of NAFLD [20]. ALB binding function is strongly associated with NAFLD. Such key mediators of NAFLD as metabolism, reactive oxygen species, oxidative stress, and inflammation interfere with posttranslational modifications of ALB, which may partially explain why ALB binding function decreases earlier than other liver enzymes in NAFLD patients. Reduced ALB binding capacity may lead to toxic metabolite accumulation and poor antioxidant capacity, thus resulting in exacerbation of NAFLD [20, 21]. In addition, ALB could readily bind to Ca2^+^, Na^+^ and fatty acids and promote the occurrence and development of NAFLD through the interaction between Yes-associated protein and TGF-*β* signaling pathways [22]. Previous studies have also shown that MAPK1 can regulate liver lipid metabolism, and CASP3 plays a vital role in the initiation and propagation of apoptosis, which is involved in various disorders, such as neurodegenerative and inflammatory diseases [23, 24]. Moreover, MAPK8 was confirmed to be related to liver regeneration in mice and might contribute to a preventive effect on NAFLD [25]. In addition, extensive investigations have demonstrated that androgen plays an essential role in the process of glycolipid metabolism. As a member of the nuclear receptor superfamily distributed in many tissues and organs of the body, AR can increase the release of androgens, thus strengthening the regulation of liver lipid metabolism and maintaining the homeostasis of fat synthesis and decomposition [26]. It can also decrease the expression levels of TNF-*α*, IL-6, and IL-1*β* and reduce the damage of inflammatory factors to liver cells [27]. The above research results suggest that FLD may exert anti-NAFLD activity through modulating such core targets as ALB, MAPKI, CASP3, MAPK8, and AR.

**Figure 9:**
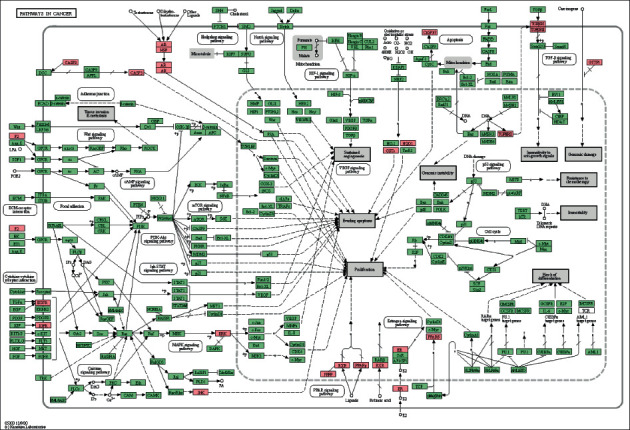
Potential target proteins of FLD regulating NAFLD on the predicted pathways (the pink nodes are potential target proteins of FLD, and the green nodes are relevant targets in the pathway).

GO and KEGG analyses were conducted using the Metascape data platform. The Go results suggested that putative targets were mainly enriched in cellular response to organic cyclic compound, steroid metabolic process, and response to steroid hormones in BP, vesicle lumen, secretory granule lumen, and ficolin-1-rich granule lumen in CC, and nuclear receptor activity, transcription factor activity, direct ligand regulated sequence-specific DNA binding, and lipid binding in MF. Additionally, the KEGG analysis yielded 181 entries, 16 of which were directly related to the NAFLD, and three pathways that significantly enriched, including pathways in cancer, Th17 cell differentiation, and IL-17 signaling pathway, were retrieved as core pathways for FLD to treat NAFLD. Specifically, the most aggregated targets were pathways in cancer, such as PPAR signaling, MAPK signaling pathway, and Estrogen signaling pathway (Figure 9). Their roles in regulating fat metabolism, improving liver fibrosis and cell apoptosis, and reducing oxidative damage are well-documented [28–30]. It is worth noting that GC and FZ in the FLD have 11 and 4 compounds that act on the pathways in cancer, respectively, indicating that modifying these two herbs may significantly influence the ratio of active compounds in FLD and provide references for conducting further experiments. Previous studies have also revealed that Th17 is involved in the inflammatory response of NAFLD progression to nonalcoholic steatohepatitis [31]. Moreover, IL-17 is classic proinflammatory cytokines and has proinflammatory effects that can accelerate NAFLD progression in mice [32]. Hence, it is rational to presume that various mechanisms may get involved in the FLD's actions on NAFLD.

Further, the molecular docking analysis results revealed that all compound-target pairs were lower than 0, indicating that all ten core compounds have good binding affinity to each of the three key targets, namely CASP3, ALB, and MAPK1. The docking pair of 3′-methoxyglabridin-ALB presented the tightest binding (−9.5), followed by chrysanthemaxanthin-MAPK1(−9.2) and Gancaonin H-ALB (−9.1), suggesting that the core compounds of FLD might relieve NAFLD through binding ALB, MAPK1, and H-ALB.

## 5. Conclusion

In conclusion, this network pharmacology-based study revealed the active compounds and potential mechanism through which FLD is effective on NAFLD by modulating various pathways, such as pathways in cancer, Th17 cell differentiation, and IL-17 signaling pathway. Subsequent molecular docking demonstrated that the top ten compounds of FLD presented desirable binding energy with CASP3, ALB, and MAPK1, further revealing the potentional mechanism of FLD's action on NAFLD. Thus, this study preliminarily reflected the multicomponent, multitarget, and multipathway characteristics of FLD and may provide some insights for future research and development of new anti-NAFLD drugs. However, further experiments are still needed to validate these findings. Special attention will be paid to interpret the mutual interactions between various compounds (or compound compatibility) that may attenuate potential toxicity, decode a structure-activity relationship between the compounds that suggests a chemical nucleus and implies a possibility of structural modification to increase activity, and identify the potential drug “cocktail” - a small collection of drug molecules ideally binding all desired variants [33].

## Figures and Tables

**Table 1:** Active compounds of FLD in NAFLD treatment.

Molecule ID	Molecule name	OB (%)	DL (%)	Source
MOL002211	11,14-Eicosadienoic acid	39.99	0.2	FZ1
MOL002388	Delphin_qt	57.76	0.28	FZ2
MOL002392	Deltoin	46.69	0.37	FZ3
MOL002393	Demethyldelavaine A	34.52	0.18	FZ4
MOL002394	Demethyldelavaine B	34.52	0.18	FZ5
MOL002395	Deoxyandrographolide	56.3	0.31	FZ6
MOL002397	Karakoline	51.73	0.73	FZ7
MOL002398	Karanjin	69.56	0.34	FZ8
MOL002401	Neokadsuranic acid B	43.1	0.85	FZ9
MOL002406	2,7-Dideacetyl-2,7-dibenzoyl-taxayunnanine F	39.43	0.38	FZ10
MOL002410	Benzoylnapelline	34.06	0.53	FZ11
MOL002415	6-Demethyldesoline	51.87	0.66	FZ12
MOL002416	Deoxyaconitine	30.96	0.24	FZ13
MOL002419	(R)-Norcoclaurine	82.54	0.21	FZ14
MOL002421	Ignavine	84.08	0.25	FZ15
MOL002422	Isotalatizidine	50.82	0.73	FZ16
MOL002423	Jesaconitine	33.41	0.19	FZ17
MOL002433	(3R,8S,9R,10 R,13R,14S,17R)-3-Hydroxy-4,4,9,13,14-pentamethyl-17-[(E,2R)-6-methyl-7-[(2R,3 R,4S,5S,6R)-3,4,5-trihydroxy-6-[[(2R,3 R,4S,5S,6R)-3,4,5-trihydroxy-6-(hydroxymethyl)oxan-2-yl]oxymethyl]oxan-2-yl]oxyhept-5-en-2-yl]-1,2,3,7,8,10,12,15,16,17-decahydr	41.52	0.22	FZ18
MOL002434	Carnosifloside I_qt	38.16	0.8	FZ19
MOL000359	Sitosterol	36.91	0.75	FZ20
MOL000538	Hypaconitine	31.39	0.26	FZ21
MOL002464	1-Monolinolein	37.18	0.3	GJ1
MOL002501	[(1S)-3-[(E)-but-2-enyl]-2-Methyl-4-oxo-1-cyclopent-2-enyl] (1r,3 R)-3-[(E)-3-methoxy-2-methyl-3-oxoprop-1-enyl]-2,2-dimethylcyclopropane-1-carboxylate	62.52	0.31	GJ2
MOL002514	Sexangularetin	62.86	0.3	GJ3
MOL000358	Beta-sitosterol	36.91	0.75	GJ4
MOL000359	Sitosterol	36.91	0.75	GJ5
MOL000020	12-Senecioyl-2E,8 E,10E-atractylentriol	62.4	0.22	BZ1
MOL000021	14-Acetyl-12-senecioyl-2E,8 E,10E-atractylentriol	60.31	0.31	BZ2
MOL000022	14-Acetyl-12-senecioyl-2E,8Z,10E-atractylentriol	63.37	0.3	BZ3
MOL000028	*α*-Amyrin	39.51	0.76	BZ4
MOL000033	(3S,8S,9S,10R,13R,14S,17R)-10,13-Dimethyl-17-[(2R,5S)-5-propan-2-yloctan-2-yl]-2,3,4,7,8,9,11,12,14,15,16,17-dodecahydro-1h-cyclopenta[a]phenanthren-3-ol	36.23	0.78	BZ5
MOL000049	3*β*-Acetoxyatractylone	54.07	0.22	BZ6
MOL000072	8*β*-Ethoxy atractylenolide III	35.95	0.21	BZ7
MOL002879	Diop	43.59	0.39	RS1
MOL000449	Stigmasterol	43.83	0.76	RS2
MOL000358	Beta-sitosterol	36.91	0.75	RS3
MOL003648	Inermin	65.83	0.54	RS4
MOL000422	Kaempferol	41.88	0.24	RS5
MOL004492	Chrysanthemaxanthin	38.72	0.58	RS6
MOL005308	Aposiopolamine	66.65	0.22	RS7
MOL005314	Celabenzine	101.88	0.49	RS8
MOL005317	Deoxyharringtonine	39.27	0.81	RS9
MOL005318	Dianthramine	40.45	0.2	RS10
MOL005320	Arachidonate	45.57	0.2	RS11
MOL005321	Frutinone A	65.9	0.34	RS12
MOL005344	Ginsenoside rh2	36.32	0.56	RS13
MOL005348	Ginsenoside-Rh4_qt	31.11	0.78	RS14
MOL005356	Girinimbin	61.22	0.31	RS15
MOL005357	Gomisin B	31.99	0.83	RS16
MOL005360	Malkangunin	57.71	0.63	RS17
MOL005376	Panaxadiol	33.09	0.79	RS18
MOL005384	Suchilactone	57.52	0.56	RS19
MOL005399	Alexandrin_qt	36.91	0.75	RS20
MOL005401	Ginsenoside Rg5_qt	39.56	0.79	RS21
MOL000787	Fumarine	59.26	0.83	RS22
MOL001484	Inermine	75.18	0.54	GC1
MOL001792	DFV	32.76	0.18	GC2
MOL000211	Mairin	55.38	0.78	GC3
MOL002311	Glycyrol	90.78	0.67	GC4
MOL000239	Jaranol	50.83	0.29	GC5
MOL002565	Medicarpin	49.22	0.34	GC6
MOL000354	Isorhamnetin	49.6	0.31	GC7
MOL000359	Sitosterol	36.91	0.75	GC8
MOL003656	Lupiwighteone	51.64	0.37	GC9
MOL003896	7-Methoxy-2-methyl isoflavone	42.56	0.2	GC10
MOL000392	Formononetin	69.67	0.21	GC11
MOL000417	Calycosin	47.75	0.24	GC12
MOL000422	Kaempferol	41.88	0.24	GC13
MOL004328	Naringenin	59.29	0.21	GC14
MOL004805	(2S)-2-[4-Hydroxy-3-(3-methylbut-2-enyl)phenyl]-8,8-dimethyl-2,3-dihydropyrano[2,3-f]chromen-4-one	31.79	0.72	GC15
MOL004806	Euchrenone	30.29	0.57	GC16
MOL004808	Glyasperin B	65.22	0.44	GC17
MOL004810	Glyasperin F	75.84	0.54	GC18
MOL004811	Glyasperin C	45.56	0.4	GC19
MOL004814	Isotrifoliol	31.94	0.42	GC20
MOL004815	(E)-1-(2,4-Dihydroxyphenyl)-3-(2,2-dimethylchromen-6-yl)prop-2-en-1-one	39.62	0.35	GC21
MOL004820	Kanzonols W	50.48	0.52	GC22
MOL004824	(2S)-6-(2,4-Dihydroxyphenyl)-2-(2-hydroxypropan-2-yl)-4-methoxy-2,3-dihydrofuro[3,2-g]chromen-7-one	60.25	0.63	GC23
MOL004827	Semilicoisoflavone B	48.78	0.55	GC24
MOL004828	Glepidotin A	44.72	0.35	GC25
MOL004829	Glepidotin B	64.46	0.34	GC26
MOL004833	Phaseolinisoflavan	32.01	0.45	GC27
MOL004835	Glypallichalcone	61.6	0.19	GC28
MOL004838	8-(6-Hydroxy-2-benzofuranyl)-2,2-dimethyl-5-chromenol	58.44	0.38	GC29
MOL004841	Licochalcone B	76.76	0.19	GC30
MOL004848	Licochalcone G	49.25	0.32	GC31
MOL004849	3-(2,4-Dihydroxyphenyl)-8-(1,1-dimethylprop-2-enyl)-7-hydroxy-5-methoxy-coumarin	59.62	0.43	GC32
MOL004855	Licoricone	63.58	0.47	GC33
MOL004856	Gancaonin A	51.08	0.4	GC34
MOL004857	Gancaonin B	48.79	0.45	GC35
MOL004860	Licorice glycoside E	32.89	0.27	GC36
MOL004863	3-(3,4-Dihydroxyphenyl)-5,7-dihydroxy-8-(3-methylbut-2-enyl)chromone	66.37	0.41	GC37
MOL004864	5,7-Dihydroxy-3-(4-methoxyphenyl)-8-(3-methylbut-2-enyl)chromone	30.49	0.41	GC38
MOL004866	2-(3,4-Dihydroxyphenyl)-5,7-dihydroxy-6-(3-methylbut-2-enyl)chromone	44.15	0.41	GC39
MOL004879	Glycyrin	52.61	0.47	GC40
MOL004882	Licocoumarone	33.21	0.36	GC41
MOL004883	Licoisoflavone	41.61	0.42	GC42
MOL004884	Licoisoflavone B	38.93	0.55	GC43
MOL004885	Licoisoflavanone	52.47	0.54	GC44
MOL004891	Shinpterocarpin	80.3	0.73	GC45
MOL004898	(E)-3-[3,4-Dihydroxy-5-(3-methylbut-2-enyl)phenyl]-1-(2,4-dihydroxyphenyl)prop-2-en-1-one	46.27	0.31	GC46
MOL004903	Liquiritin	65.69	0.74	GC47
MOL004904	Licopyranocoumarin	80.36	0.65	GC48
MOL004905	3,22-Dihydroxy-11-oxo-delta(12)-oleanene-27-alpha-methoxycarbonyl-29-oic acid	34.32	0.55	GC49
MOL004907	Glyzaglabrin	61.07	0.35	GC50
MOL004908	Glabridin	53.25	0.47	GC51
MOL004910	Glabranin	52.9	0.31	GC52
MOL004911	Glabrene	46.27	0.44	GC53
MOL004912	Glabrone	52.51	0.5	GC54
MOL004913	1,3-Dihydroxy-9-methoxy-6-benzofurano [3,2-c]chromenone	48.14	0.43	GC55
MOL004914	1,3-Dihydroxy-8,9-dimethoxy-6-benzofurano [3,2-c]chromenone	62.9	0.53	GC56
MOL004915	Eurycarpin A	43.28	0.37	GC57
MOL004917	Glycyroside	37.25	0.79	GC58
MOL004924	(-)-Medicocarpin	40.99	0.95	GC59
MOL004935	Sigmoidin-B	34.88	0.41	GC60
MOL004941	(2R)-7-Hydroxy-2-(4-hydroxyphenyl)chroman-4-one	71.12	0.18	GC61
MOL004945	(2S)-7-Hydroxy-2-(4-hydroxyphenyl)-8-(3-methylbut-2-enyl)chroman-4-one	36.57	0.32	GC62
MOL004948	Isoglycyrol	44.7	0.84	GC63
MOL004949	Isolicoflavonol	45.17	0.42	GC64
MOL004957	HMO	38.37	0.21	GC65
MOL004959	1-Methoxyphaseollidin	69.98	0.64	GC66
MOL004961	Quercetin der.	46.45	0.33	GC67
MOL004966	3'-Hydroxy-4'-O-Methylglabridin	43.71	0.57	GC68
MOL000497	Licochalcone a	40.79	0.29	GC69
MOL004974	3'-Methoxyglabridin	46.16	0.57	GC70
MOL004978	2-[(3R)-8,8-Dimethyl-3,4-dihydro-2h-pyrano [6,5-f]chromen-3-yl]-5-methoxyphenol	36.21	0.52	GC71
MOL004980	Inflacoumarin A	39.71	0.33	GC72
MOL004985	Icos-5-enoic acid	30.7	0.2	GC73
MOL004988	Kanzonol F	32.47	0.89	GC74
MOL004989	6-Prenylated eriodictyol	39.22	0.41	GC75
MOL004990	7,2',4'-Trihydroxy－5-methoxy-3－arylcoumarin	83.71	0.27	GC76
MOL004991	7-Acetoxy-2-methylisoflavone	38.92	0.26	GC77
MOL004993	8-Prenylated eriodictyol	53.79	0.4	GC78
MOL004996	Gadelaidic acid	30.7	0.2	GC79
MOL000500	Vestitol	74.66	0.21	GC80
MOL005000	Gancaonin G	60.44	0.39	GC81
MOL005001	Gancaonin H	50.1	0.78	GC82
MOL005003	Licoagrocarpin	58.81	0.58	GC83
MOL005007	Glyasperins M	72.67	0.59	GC84
MOL005008	Glycyrrhiza flavonol A	41.28	0.6	GC85
MOL005012	Licoagroisoflavone	57.28	0.49	GC86
MOL005013	18*α*-Hydroxyglycyrrhetic acid	41.16	0.71	GC87
MOL005016	Odoratin	49.95	0.3	GC88
MOL005017	Phaseol	78.77	0.58	GC89
MOL005018	Xambioona	54.85	0.87	GC90
MOL005020	Dehydroglyasperins C	53.82	0.37	GC91
MOL000098	Quercetin	46.43	0.28	GC92
MOL000359	Sitosterol	36.91	0.75	FZ20, Gj5, GC8 (A1)
MOL000358	Beta-sitosterol	36.91	0.75	GJ4, RS3(B1)
MOL000422	Kaempferol	41.88	0.24	RS5,GC13(C1)

**Table 2:** Core active compounds of FLD.

Molecule ID	Molecule name	Degree	Source
MOL000022	14-Acetyl-12-senecioyl-2E,8Z,10E-atractylentriol	51	BZ3
MOL005320	Arachidonate	45	RS11
MOL002423	Jesaconitine	45	FZ17
MOL002879	Diop	44	RS1
MOL004974	3′-Methoxyglabridin	44	GC56
MOL004898	(E)-3-[3,4-Dihydroxy-5-(3-methylbut-2-enyl)phenyl]-1-(2,4-dihydroxyphenyl)prop-2-en-1-one	40	GC42
MOL004492	Chrysanthemaxanthin	39	RS6
MOL002211	11,14-Eicosadienoic acid	38	FZ1
MOL005360	Malkangunin	38	RS17
MOL005001	Gancaonin H	38	GC77

**Table 3:** The binding energy values of core compounds of FLD and core targets.

Molecule name	Target	PDB ID	Binding affinity (KJ·mol^−1^)
14-Acetyl-12-senecioyl-2E,8Z,10E-atractylentriol	ALB	1BJ5	−6.9
	MAPK1	6SLG	−5.3
	CASP3	2J32	−5.0

Arachidonate	ALB	1BJ5	−7.3
	MAPK1	6SLG	−5.0
	CASP3	2J32	−3.5

Jesaconitine	ALB	1BJ5	−7.0
	MAPK1	6SLG	−6.5
	CASP3	2J32	−5.3

Diop	ALB	1BJ5	−7.5
	MAPK1	6SLG	−6.0
	CASP3	2J32	−4.3

3′-Methoxyglabridin	ALB	1BJ5	−9.5
	MAPK1	6SLG	−7.5
	CASP3	2J32	−7.4

(E)-3-[3,4-Dihydroxy-5-(3-methylbut-2-enyl)phenyl]-1-(2,4-dihydroxyphenyl)prop-2-en-1-one	ALB	1BJ5	−7.9
	MAPK1	6SLG	−7.1
	CASP3	2J32	−6.4

Chrysanthemaxanthin	ALB	1BJ5	−6.1
	MAPK1	6SLG	−9.1
	CASP3	2J32	−8.2

11,14-Eicosadienoic acid	ALB	1BJ5	−6.3
	MAPK1	6SLG	−4.4
	CASP3	2J32	−4.4

Malkangunin	ALB	1BJ5	−6.8
	MAPK1	6SLG	−6.5
	CASP3	2J32	−7.1

Gancaonin H	ALB	1BJ5	−9.2
	MAPK1	6SLG	−7.6
	CASP3	2J32	−7.6

ALB: albumin; MAPK1: mitogen-activated protein kinase 1; CASP3: caspase-3.

## Data Availability

The datasets generated and/or analyzed during the current study are available from the corresponding author on request.

## Abbreviations

FLD: Fuzi Lizhong Decoction
NAFLD: Nonalcoholic fatty liver disease
TCMSP: Traditional Chinese Medicine Systems Pharmacology
DL: Drug-likeness
OB: Oral bioavailability
GO: Gene ontology
BP: Biological process
KEGG: Kyoto Encyclopedia of Genes and Genomes
ALB: Albumin
MAPK1: Mitogen-activated protein kinase 1
CASP3: Caspase-3
MAPK8: Mitogen-activated protein kinase 8
AR: Androgen receptor
HSP90AA1: Heat shock protein HSP 90-alpha
EGFR: Epidermal growth factor receptor
CC: Cellular component
MF: Molecular Function
MAPK14: Mitogen-activated protein kinase 14
TGFBR1: TGF-beta receptor type-1
TCM: Traditional Chinese medicine.

## Supplementary Materials

Supplementary Materials Supplement Table 1: detailed information about the compound-target network. Supplement Table 2: information of 2,608 NAFLD-related targets. Supplement Table 3: information of 61 intersection targets between FLD and NAFLD. Supplement Table 4: detailed information about the protein-protein network. Supplement Table 5: detailed information about functional analysis.
