# Supplementary material for: Action Mechanism Underlying Improvement Effect of Fuzi Lizhong Decoction on Nonalcoholic Fatty Liver Disease: A Study Based on Network Pharmacology and Molecular Docking
Source: Evid Based Complement Alternat Med. 2022 Jan 20;2022:1670014. doi: 10.1155/2022/1670014 (PMC8794673; doi:10.1155/2022/1670014)
Supplement: Supplementary Materials — Supplement Table 1: detailed information about the compound-target network. Supplement Table 2: information of 2,608 NAFLD-related targets. Supplement Table 3: information of 61 intersection targets between FLD and NAFLD. Supplement Table 4: detailed information about the protein-protein network. Supplement Table 5: detailed information about functional analysis. [file 1670014.f1.zip › 1670014.f1/Supplement Table 1 Detailed information about the compound-target network.pdf]

Detailed Information of 738 Compounds

Mol ID	Molecule Name	MW	AlogP	Hdon	Hacc	OB (%)	Caco-2	BBB	DL	FASA-	HL	Save	Source
MOL000012	Arachic acid	312.6	8.19	1	2	16.66	1.18	1.09	0.19	0.18			
MOL000131	EIC	280.5	6.39	1	2	41.9	1.16	0.9	0.14	0.25	7.5		
MOL001393	myristic acid	228.42	5.46	1	2	21.18	1.07	0.99	0.07	0.19			
MOL001744	uracil	112.1	-1.01	2	4	42.53	0.05	-0.08	0.02	0	11.77		
MOL002089	mesaconitine	631.79	-1.33	3	12	8.7	-0.35	-0.95	0.25	0.2			
MOL002093	benzoylmesaconine	589.75	-1.71	4	11	8.55	-0.52	-1.11	0.27	0.21			
MOL002211	11,14-eicosadienoic acid	308.56	7.3	1	2	39.99	1.22	0.76	0.2	0.23	5.6		
MOL002383	13-Deoxo-13 alpha-acetyloxy-1-deoxynortaxine I	583.79	3	4	9	25.68	-0.42	-1.01	0.79	0.28			
MOL002384	14-Deoxy-11,12-didehydroandrographolide	332.48	2.1	2	4	13.6	0.09	-0.68	0.32	0.29			
MOL002385	Delcorine	479.68	-0.94	1	8	7.52	0.4	0.42	0.36	0.09			
MOL002386	Delphamine	453.64	-1.97	4	8	13.74	-0.04	-0.25	0.66	0.1			
MOL002387	Delphin	627.58	-2.32	12	17	2.61	-3.16	-4.36	0.67	0.26			
MOL002388	Delphin_qt	303.26	1.67	6	7	57.76	0.12	-0.66	0.28	0.35	1.43		
MOL002389	Delsemine B	699.93	-0.72	5	13	18.32	-0.61	-1.07	0.18	0.22			
MOL002390	Delsoline	467.67	-1.56	3	8	17.23	0.03	-0.43	0.63	0.1			
MOL002391	Deltamine	465.65	-1.11	2	8	9.62	0.3	0.13	0.37	0.12			
MOL002392	Deltoin	328.39	2.48	0	5	46.69	0.55	-0.12	0.37	0.32	7.7		
MOL002393	Demethyldelavaine A	700.91	-0.12	4	13	34.52	-0.69	-1.19	0.18	0.23	26.05		
MOL002394	Demethyldelavaine B	700.91	-0.12	4	13	34.52	-0.7	-1.01	0.18	0.22	26.75		
MOL002395	Deoxyandrographolide	334.5	3.02	2	4	56.3	0.18	-0.49	0.31	0.27	2.79		
MOL002396	14-acetyltalatisamine	463.68	0.06	1	7	13.64	0.15	-0.11	0.63	0.15			
MOL002397	karakoline	377.58	-0.05	3	5	51.73	0.32	-0.03	0.73	0.15	11.1		
MOL002398	Karanjin	292.3	2.94	0	4	69.56	1.22	0.62	0.34	0.26	13.15		
MOL002399	TMPEA	211.29	1.21	2	4	47.54	0.64	0	0.06	0.15	3.52		
MOL002400	Neojiangyouaconitine	601.81	0.28	2	10	9.83	0.01	-0.42	0.26	0.19			
MOL002401	Neokadsuranic acid B	452.74	7.05	1	3	43.1	0.69	-0.01	0.85	0.29	12.05		
MOL002402	Senbusine A	423.61	-1.67	4	7	22.26	-0.18	-0.52	0.71	0.13			
MOL002403	Senbusine B	423.61	-1.3	4	7	14.54	-0.5	-0.82	0.71	0.14			
MOL002404	Senbusine C	453.64	-1.83	4	8	19.29	-0.26	-0.43	0.66	0.14			
MOL002405	Tamarixinol	446.84	12.07	1	2	12.18	1.54	1.07	0.59	0.2			
MOL002406	2,7-Dideacetyl-2,7-dibenzoyl-taxayunnanine F	776.9	3.12	1	14	39.43	-0.75	-1.3	0.38	0.38	10.95		
MOL002407	beiwutine	647.79	-2.3	4	13	14.88	-0.92	-1.31	0.24	0.21			
MOL002408	benzoylaconine	603.78	-1.36	4	11	12.83	-0.48	-1.12	0.25	0.21			
MOL002409	benzoylhypaconine	573.75	-0.48	3	10	8.7	-0.29	-0.74	0.29	0.24			
MOL002410	benzoylnapelline	463.67	3.12	2	5	34.06	0.19	-0.38	0.53	0.31	15.72		
MOL002411	chasmaconitine	613.82	0.82	1	10	8.65	0.39	-0.1	0.26	0.2			

MOL002412	chasmanine	451.67	-0.85	2	7	15.77	0.45	0.26	0.67	0.08		FuZi
MOL002413	coryneine	196.3	0.34	2	2	3.55	1.14	1.12	0.05	0.22		
MOL002414	denudatine	343.56	2.25	2	3	16.55	0.87	0.57	0.67	0.24		
MOL002415	6-Demethyldesoline	453.64	-1.97	4	8	51.87	-0.26	-0.57	0.66	0.12	13.14	
MOL002416	deoxyaconitine	629.82	0.25	2	11	30.96	-0.23	-0.61	0.24	0.19	22.64	
MOL002417	fuzitine	342.45	3.12	2	4	25.79	1.02	0.69	0.54	0.17		
MOL002418	hetisine	329.48	-0.41	3	4	29.12	-0.56	-1.09	0.59	0.22		
MOL002419	(R)-Norcoclaurine	271.34	2.57	4	4	82.54	0.63	0.03	0.21	0.34	3.85	
MOL002420	hokbusine A	603.78	-1.3	3	11	21.74	-0.5	-1.05	0.26	0.22		
MOL002421	ignavine	449.59	1.22	3	6	84.08	-0.07	-0.49	0.25	0.32	28.93	
MOL002422	isotalatizidine	407.61	-0.73	3	6	50.82	-0.11	-0.62	0.73	0.14	11.59	
MOL002423	jesaconitine	675.85	-0.99	3	13	33.41	-0.44	-1.16	0.19	0.19	26.06	
MOL002424	Aconitine	645.82	-0.98	3	12	7.87	-0.58	-1.13	0.23	0.22		
MOL002425	Zoba EG	109.14	0.82	3	2	66.14	0.56	0.31	0.02	0.39	12.01	
MOL002426	neoline	437.64	-1.26	3	7	13.32	0.14	-0.14	0.69	0.13		
MOL002427	Zoba 3GA	109.14	0.82	3	2	65.8	0.69	0.73	0.02	0.37	11.99	
MOL002428	UNAL	109.14	0.82	3	2	67.62	0.51	0.2	0.02	0.39	11.94	
MOL002429	Pyrrolezanthine	245.3	2.51	2	3	11.99	0.42	-0.03	0.12	0.26		
MOL002430	Salsolinol	179.24	1.29	3	3	36.83	0.81	0.67	0.06	0.29	1.87	
MOL002431	songorine	357.54	0.9	2	4	24.46	0.03	-0.27	0.72	0.26		
MOL002432	talatizamine	421.64	-0.32	2	6	16.94	0.51	0.15	0.72	0.1		
MOL002433	(3R,8S,9R,10R,13R,14S,17R)-3-hydroxy-4,4,9,13,14-pentamethyl-17-[(E,2R)-6-methyl-7-[(2R,3R,4S,5S,6R)-3,4,5-trihydroxy-6-[[(2R,3R,4S,5S,6R)-3,4,5-trihydroxy-6-(hydroxymethyl)oxan-2-yl]oxymethyl]oxan-2-yl]oxyhept-5-en-2-yl]-1,2,3,7,8,10,12,15,16,17-decahydr	781.1	2.2	8	13	41.52	-2.31	-3.18	0.22	0.25	10.51	
MOL002434	Carnosifloside I_qt	456.78	5.69	2	3	38.16	0.28	-0.77	0.8	0.25	7	
MOL002435	STOCK1N-53073	499.67	-3.4	5	10	8	-0.45	-0.9	0.58	0.12		
MOL002436	Delavaconitine	497.69	1.38	2	7	9.91	0.3	-0.06	0.37	0.26		
MOL002437	Delbruline	479.68	-0.94	1	8	13.6	0.3	0	0.36	0.11		
MOL002438	Delbrusine	493.71	-0.54	0	8	7.51	0.68	0.44	0.33	0.09		
MOL000359	sitosterol	414.79	8.08	1	1	36.91	1.32	0.87	0.75	0.22	5.37	
MOL000538	hypaconitine	615.79	-0.1	2	11	31.39	-0.34	-0.78	0.26	0.21	19.87	

MOL001055	5-isopropyl-2-methylbicyclo[3.1.0]hex-2-ene	136.26	2.87	0	0	47.19	1.82	2.18	0.04	0.29	11.44
MOL001121	19894-97-4	152.26	1.78	1	1	49.98	1.25	1.5	0.06	0.21	11.55
MOL000117	Cymol	134.24	3.51	0	0	27.2	1.86	2.1	0.02	0.34	
MOL000118	(L)-alpha-Terpineol	154.28	2.42	1	1	48.8	1.39	1.72	0.03	0.27	11.35
MOL000119	ZINC02040970	222.41	4.56	1	1	40.43	1.44	1.31	0.06	0.3	4.73
MOL001201	(1R,5R,7S)-4,7-dimethyl-7-(4-methylpent-3-enyl)bicyclo[3.1.1]hept-3-ene	204.39	4.7	0	0	16.23	1.86	1.96	0.09	0.27	
MOL000121	Decanal	156.3	3.68	0	1	29.81	1.34	1.44	0.02	0.2	
MOL001210	(4S)-4-isopropylcyclohexene-1-carbaldehyde	152.26	2.86	0	1	40.36	1.36	1.69	0.03	0.28	1.98
MOL000122	1,8-cineole	154.28	2.15	0	1	39.73	1.57	2.06	0.05	0.24	11.29
MOL000123	geraniol	154.28	2.93	1	1	23.93	1.19	1.14	0.02	0.27	
MOL000124	citral	152.26	3.19	0	1	22.52	1.33	1.43	0.02	0.33	
MOL000125	(-)-alpha-Pinene	136.26	2.87	0	0	46.25	1.85	2.3	0.05	0.25	11.42
MOL000126	(-)-nopinene	136.26	2.93	0	0	44.84	1.8	2.12	0.05	0.27	11.32
MOL000127	Neral	152.26	3.19	0	1	19.48	1.36	1.51	0.02	0.34	
MOL000128	NERYLACETATE	196.32	3.31	0	2	25.94	1.28	1.28	0.04	0.27	
MOL000131	EIC	280.5	6.39	1	2	41.9	1.16	0.9	0.14	0.25	7.5
MOL001394	Oktadekan	254.56	8.58	0	0	9.81	1.83	1.83	0.09	0.14	
MOL001396	PENTADECYLIC ACID	242.45	5.91	1	2	20.18	1.08	0.88	0.08	0.18	
MOL001600	copaene	204.39	4.17	0	0	29.47	1.81	2.04	0.12	0.22	
MOL001619	UPL	268.59	9.04	0	0	8.52	1.83	1.7	0.11	0.16	
MOL000170	guaiene	204.39	5.13	0	0	28.21	1.83	2.03	0.07	0.23	
MOL001719	2-[(2S,5R)-5-ethenyl-5-methyloxolan-2-yl]propan-2-ol	170.28	1.43	1	2	68.08	0.99	1.14	0.04	0.31	11
MOL000018	(+/-)-Isoborneol	154.28	1.98	1	1	86.98	1.27	1.6	0.05	0	11.36
MOL001899	ZINC02169908	268.54	6.2	0	1	23.3	1.42	1.24	0.1	0.23	
MOL000019	D-Camphene	136.26	2.93	0	0	34.98	1.81	2.19	0.04	0	11.29
MOL000196	L-Bornyl acetate	196.32	2.35	0	2	65.52	1.29	1.59	0.08	0.23	6.94
MOL000197	Myrcene	136.26	3.69	0	0	24.96	1.84	1.98	0.02	0.37	
MOL000198	(R)-linalool	154.28	2.74	1	1	39.8	1.33	1.36	0.02	0.32	6.48
MOL000200	(S)-(+)-alpha-Phellandrene	136.26	3.25	0	0	27.9	1.87	2.2	0.02	0.29	
MOL000201	p-Ocimene	136.26	3.63	0	0	15.06	1.85	1.99	0.02	0.39	
MOL000202	Moslene	136.26	3.45	0	0	33.02	1.88	2.05	0.02	0.27	11.08
MOL002028	(+)-beta-Phellandrene	136.26	3.31	0	0	40.3	1.83	2.2	0.02	0.3	11.28

MOL000204	-cis-.beta.-Elemene diastereomer	204.39	4.79	0	0	28.62	1.85	2.09	0.06	0.33	
MOL002085	alpha-Cubebene	204.39	4.17	0	0	16.73	1.83	2.1	0.11	0.25	
MOL002175	3-cyclohexen-1-ol	98.16	1.06	1	1	70.57	1.14	1.52	0.01	0.25	11.25
MOL000023	Hemo-sol	136.26	3.5	0	0	39.84	1.83	2.12	0.02	0	11.68
MOL002361	Terragon	148.22	2.82	0	1	36.59	1.72	1.83	0.03	0.34	1.79
MOL000244	()-Borneol	154.28	1.98	1	1	81.8	1.22	1.47	0.05	0.24	11.36
MOL002450	(+)-1,5-Epoxy-nor-ketoguaia-11-ene	220.34	2.46	0	2	62.84	1.05	1.05	0.12	0.31	6.4
MOL002451	尾-humulene	190.36	4.63	0	0	40.36	1.83	2	0.05	0.3	2.42
MOL002452	尾-sesquiphellandrene	204.39	5.14	0	0	16.2	1.88	2.07	0.06	0.31	
MOL002453	(-)-Comphene	136.26	2.93	0	0	34.98	1.81	2.21	0.04	0.28	11.29
MOL002454	10-Gingerdione	348.53	5.68	2	4	21.42	0.69	0.11	0.29	0.24	
MOL002455	[(3S)-3,7-dimethyloct-6-enyl] acetate	198.34	3.43	0	2	22.68	1.31	1.38	0.04	0.24	
MOL002456	[(3R)-3,7-dimethyloct-6-enyl] butanoate	226.4	4.55	0	2	21.03	1.33	1.31	0.06	0.25	
MOL002457	Cyclopropyl ketone	110.17	1.27	0	1	62.18	1.25	1.62	0.02	0.37	11.55
MOL002458	ZINC01850974	154.28	2.11	1	1	78.86	1.31	1.6	0.05	0.23	11.19
MOL002459	(S)-5-Hydroxy-1-(4-hydroxy-3-methoxyphenyl)-3-tetradecanone	350.55	5.3	2	4	19.14	0.48	0.04	0.28	0.23	
MOL002460	12,13-Di-acetoxyl-1,4,6,11-eudesmanetetol	388.51	0.15	4	8	17.51	-0.54	-1.11	0.34	0.22	
MOL002461	Gingerglycolipid A	676.89	2.8	8	14	13.62	-1.71	-2.44	0.47	0.25	
MOL002462	gingerglycolipid A_qt	514.73	4.54	5	9	11.71	-0.36	-1.13	0.64	0.27	
MOL002463	[(2S)-2-hydroxy-3-[(2R,3R,4S,5R,6R)-3,4,5-trihydroxy-6-[[(2S,3R,4S,5R,6R)-3,4,5-trihydroxy-6-(hydroxymethyl)tetrahydropyran-2-yl]oxymethyl]tetrahydropyran-2-yl]oxy-propyl] (9Z,12Z)-octadeca-9,12-dienoate	678.91	2.1	8	14	22.78	-2.11	-3.11	0.4	0.23	
MOL002464	1-Monolinolein	354.59	5.59	2	4	37.18	0.32	-0.32	0.3	0.23	4.36

MOL002465	9-Octadecenoic acid (Z)-, 3-((6-O-alpha-D-galactopyranosyl-beta-D-galactopyranosyl)oxy)-2-hydroxypropyl ester, (S)-	680.93	3.69	8	14	10.02	-1.45	-1.99	0.48	0.21		
MOL002466	gingerglycolipid C_qt	518.77	5.43	5	9	9.93	-0.41	-0.87	0.62	0.21		
MOL002467	6-gingerol	294.43	3.47	2	4	35.64	0.54	-0.08	0.16	0.23	3.05	
MOL002468	hexahydrocurcumin	374.47	3.31	3	6	4.88	0.36	-0.5	0.41	0.26		
MOL002469	12-Gingerol	378.61	6.21	2	4	17.81	0.53	-0.27	0.37	0.24		
MOL000247	(Z,Z)-farnesol	222.41	4.76	1	1	41.14	1.25	1.15	0.06	0.28	6.29	
MOL002470	Isogingerenone-B	386.48	4.37	2	6	3.8	0.79	0.05	0.46	0.23		
MOL002471	[(1R,2R,5R)-2-isopropyl-5-methyl-cyclohexyl] acetate	198.34	3.16	0	2	22.19	1.29	1.57	0.05	0.22		
MOL002472	Neryl propionate	210.35	3.98	0	2	36.64	1.32	1.28	0.05	0.29	4.51	
MOL002473	ZINC04081584	302.5	3.34	1	2	13.09	0.75	0.37	0.39	0.26		
MOL002474	p-mentha-1,5-dien-8-ol	152.26	1.97	1	1	28.63	1.29	1.5	0.03	0.3		
MOL002475	himachalene	204.39	4.89	0	0	17.59	1.87	2.11	0.08	0.24		
MOL002476	2,6-Nonamethylene	203.36	4.49	0	1	68.94	1.74	1.98	0.09	0.24	9.46	
MOL002477	(2R)-2,6-dimethylhept-5-enal	140.25	2.77	0	1	28.86	1.37	1.61	0.02	0.29		
MOL002478	(2R)-heptan-2-ol	116.23	2.26	1	1	24.38	1.13	1.25	0.01	0.22		GanJiang
MOL002479	(2S)-2-methylpentanal	100.18	1.86	0	1	63.34	1.27	1.65	0.01	0.27	11.26	
MOL002480	Methylbutenol	86.15	0.84	1	1	54.58	1.12	1.32	0.01	0.33	11.47	
MOL002481	2-Methylhept-2-ene	112.24	3.57	0	0	25.56	1.81	2.07	0.01	0.26		
MOL002482	()-beta-Citronellene	138.28	3.88	0	0	23.21	1.83	2.1	0.01	0.3		
MOL002483	1-(4-Hydroxy-3-methoxyphenyl)decan-5-one	278.43	4.58	1	3	7.55	0.93	0.45	0.14	0.24		
MOL002484	Isovaleral	86.15	1.19	0	1	44.71	1.2	1.6	0	0.27	11.36	
MOL002485	4-(1,5-Dimethylhex-4-enyl)cyclohex-2-enone	206.36	3.94	0	1	45.5	1.46	1.43	0.06	0.29	4.12	
MOL002486	4-Gingerol	266.37	2.56	2	4	8.62	0.45	-0.14	0.12	0.25		
MOL002487	5-hydroxy-7-(4-hydroxyphenyl)-1-(4-hydroxy-3-methoxyphenyl)-3-heptanone	344.44	3.33	3	5	5.34	0.3	-0.81	0.32	0.3		
MOL002488	6-Gingerdione	292.41	3.85	2	4	8.87	0.75	0.22	0.16	0.27		
MOL002489	[(3S)-3,7-dimethyloct-7-enyl] acetate	198.34	3.48	0	2	31.38	1.29	1.25	0.04	0.25	5.76	
MOL002490	6-gingediacetate	380.53	4.3	1	6	6.71	0.55	-0.3	0.29	0.25		
MOL002491	6-gingediol-3-acetate	338.49	3.92	2	5	7.11	0.45	-0.32	0.22	0.23		
MOL002492	6-gingediol-5-acetate	338.49	3.92	2	5	7.06	0.41	-0.4	0.23	0.24		
MOL002493	6-gingediol	296.45	3.54	3	4	6.02	0.22	-0.58	0.16	0.25		

MOL002494	6-gingesulfonic acid	358.5	3.05	2	6	8.56	0.11	-0.18	0.23	0.33	
MOL002495	6-shogaol	276.41	4.55	1	3	31	1.07	0.49	0.14	0.25	4.07
MOL002496	Sulcatone	126.22	1.79	0	1	26.36	1.35	1.52	0.01	0.31	
MOL002497	(8)-Gingerol	322.49	4.39	2	4	7.01	0.56	-0.09	0.22	0.24	
MOL002498	PGR	76.11	-0.52	2	2	46.54	-0.14	-1.41	0	0.23	11.48
MOL002499	Bicyclo[3. 1. 0]hex-2-ene,4-methyl-1-(1-methylethyl)-	136.26	2.68	0	0	50.91	1.84	2.21	0.04	0.27	11.16
MOL002500	Campherenol	222.41	3.8	1	1	18.25	1.35	1.32	0.09	0.26	
MOL002501	[(1S)-3-[(E)-but-2-enyl]-2-methyl-4-oxo-1-cyclopent-2-enyl] (1R,3R)-3-[(E)-3-methoxy-2-methyl-3-oxoprop-1-enyl]-2,2-dimethylcyclopropane-1-carboxylate	360.49	3.68	0	5	62.52	0.37	-0.36	0.31	0.29	3.21
MOL002502	copaene	204.39	4.17	0	0	24.08	1.81	2.06	0.12	0.23	
MOL002503	Cynanuriculoside A	1085.43	1.15	7	21	14.92	-2.35	-3.33	0.03	0.2	
MOL002504	Peruviol	222.41	4.56	1	1	29.61	1.47	1.44	0.06	0.29	
MOL002505	C10230	952.68	1.04	14	27	3.01	-3.56	-4.13	0.02	0.4	
MOL002506	Gingerenone A	356.45	4.39	2	5	4.2	0.71	-0.37	0.38	0.3	
MOL002507	Gingerenone B	386.48	4.37	2	6	3.79	0.77	-0.29	0.46	0.24	
MOL002508	Gingerenone-C	326.42	4.41	2	4	4.39	0.82	-0.08	0.3	0.34	
MOL002509	Ginkgetin	566.54	4.86	4	10	22.19	0.12	-1.48	0.59	0.34	
MOL002510	Heptaphylline	279.36	4.67	2	2	14.81	1.24	0.6	0.25	0.32	
MOL002511	Isoginkgetin	566.54	4.86	4	10	21.56	0.02	-1.69	0.58	0.33	
MOL002512	Mesotrihydroxypiperidine	133.17	-1.93	4	4	50.1	-0.66	-1.46	0.02	0.21	10.65
MOL002513	Xanthorrhizol	218.37	5.07	1	1	32.1	1.72	1.64	0.07	0.32	3.5
MOL002514	Sexangularetin	316.28	1.76	4	7	62.86	0.31	-0.5	0.3	0.32	15.19
MOL002515	Shyobunone	220.39	3.84	0	1	44.28	1.34	1.58	0.07	0.35	8.84
MOL002516	zingerone	194.25	1.63	1	3	25.23	0.87	0.48	0.05	0.29	
MOL002517	Zingiberoside A3	885.17	0.28	9	17	27.64	-2.15	-3.09	0.06	0.23	
MOL002518	Zingiberoside A3_qt	414.69	4.63	1	3	14.15	0.7	0.03	0.81	0.21	
MOL002519	Decamethylenediol	174.32	2.48	2	2	26.51	0.3	-0.51	0.03	0.16	
MOL002520	.beta.-Fenchyl acetate, exo-	196.32	2.49	0	2	108.68	1.27	1.62	0.07	0.22	-2.37
MOL002521	beta-Curcumene	204.39	5.33	0	0	4.48	1.92	2.07	0.06	0.3	
MOL002522	beta-Rhodinol	156.3	3.05	1	1	38.05	1.19	1.12	0.02	0.24	5.19
MOL000264	Tereben	136.26	3.64	0	0	29.62	1.86	2.11	0.02	0.28	
MOL000267	beta-Citronellol	156.3	3.05	1	1	38.89	1.2	1.14	0.02	0.24	5.21
MOL000268	(1S,5S)-1-isopropyl-4-methylenebicyclo[3.1.0]hexane	136.26	2.93	0	0	46.21	1.83	2.18	0.04	0.31	11.47
MOL000027	alpha-Curcumene	202.37	5.34	0	0	4.68	1.93	1.99	0.06	0	

MOL000032	beta-Eudesmol	222.41	3.72	1	1	26.09	1.32	1.38	0.1	0	
MOL000034	2-[(1R,3S,4S)-3-isopropenyl-4-methyl-4-vinylcyclohexyl]propan-2-ol	222.41	3.7	1	1	19.03	1.37	1.46	0.07	0	
MOL000357	Sitogluside	576.95	6.34	4	6	20.63	-0.14	-0.93	0.62	0.23	
MOL000358	beta-sitosterol	414.79	8.08	1	1	36.91	1.32	0.99	0.75	0.23	5.36
MOL000359	sitosterol	414.79	8.08	1	1	36.91	1.32	0.87	0.75	0.22	5.37
MOL000479	Farnesene	204.39	5.52	0	0	17.42	1.95	2.21	0.05	0	
MOL000489	(1S,4aR,8aR)-1-isopropyl-7-methyl-4-methylene-2,3,4a,5,6,8a-hexahydro-1H-naphthalene	204.39	4.8	0	0	19.8	1.86	2.03	0.08	0	
MOL000597	Neryl acetate	196.32	3.31	0	2	57.47	1.25	1.4	0.04	0.27	7.92
MOL000612	(-)-alpha-cedrene	204.39	4.12	0	0	55.56	1.81	2.16	0.1	0.24	4.82
MOL000666	hexanal	100.18	1.85	0	1	55.71	1.25	1.52	0.01	0.23	10.96
MOL000675	oleic acid	282.52	6.84	1	2	33.13	1.17	0.78	0.14	0.2	4.99
MOL000676	DBP	278.38	4.2	0	4	64.54	0.8	0.56	0.13	0.34	5.41
MOL000069	palmitic acid	256.48	6.37	1	2	19.3	1.09	1	0.1	0	
MOL000710	OYA	128.24	2.77	0	1	19.07	1.3	1.52	0.01	0.23	
MOL000712	o-Cymol	134.24	3.51	0	0	51.89	1.88	2.07	0.02	0.37	11.74
MOL000765	Citraurin beta	432.7	7.1	1	2	20.53	1.07	-0.85	0.56	0.36	
MOL000774	(-)-Citronellal	154.28	3.02	0	1	35.71	1.34	1.62	0.02	0.27	6.57
MOL000863	Dekan	142.32	4.93	0	0	17.74	1.78	1.93	0.01	0.17	
MOL000867	Heptadekan	240.53	8.13	0	0	8.64	1.84	1.87	0.07	0.14	
MOL000868	LFA	282.62	9.5	0	0	8.46	1.83	1.8	0.13	0.13	
MOL000869	Henicosane	296.65	9.95	0	0	8.41	1.84	1.8	0.15	0.13	
MOL000879	methyl palmitate	270.51	6.62	0	2	18.09	1.37	1.18	0.12	0.14	
MOL000886	tetradecane	198.44	6.76	0	0	15.94	1.79	1.78	0.04	0.15	
MOL000890	(+)-alpha-Curcumene	202.37	5.34	0	0	26.56	1.93	2.03	0.06	0.33	
MOL000895	Zingiberene	204.39	5.08	0	0	18.8	1.91	2.01	0.06	0.3	
MOL000911	Terpilene	136.26	3.45	0	0	33.95	1.84	2.1	0.02	0.27	11.05
MOL000913	tricyclene	136.26	2.3	0	0	36.11	1.8	2.22	0.07	0.25	11.36
MOL000918	2-NONANONE	142.27	2.7	0	1	8.51	1.34	1.62	0.02	0.24	
MOL000924	Mnk	170.33	3.62	0	1	17.66	1.4	1.64	0.03	0.22	
MOL000926	cis-beta-farnesene	204.39	5.52	0	0	6.75	1.92	2.15	0.05	0.34	
MOL000932	alpha-Farnesene	204.39	5.46	0	0	21.7	1.97	1.89	0.05	0.32	
MOL000968	beta-Bisabolene	204.39	5.33	0	0	29.59	1.88	2.13	0.06	0.29	
MOL000018	(+/-)-Isoborneol	154.28	1.98	1	1	86.98	1.27	1.6	0.05	0	11.36
MOL000019	D-Camphene	136.26	2.93	0	0	34.98	1.81	2.19	0.04	0	11.29
MOL000020	12-senecioyl-2E,8E,10E-atractylentriol	312.39	2.5	0	4	62.4	0.01	-1.37	0.22	0.12	6.07

MOL000021	14-acetyl-12-senecioyl-2E,8E,10E-atractylentriol	355.44	3.21	0	5	60.31	0.33	-1.09	0.31	0.05	5.32	
MOL000022	14-acetyl-12-senecioyl-2E,8Z,10E-atractylentriol	356.45	3.54	1	5	63.37	0.42	-1.14	0.3	0	6.43	
MOL000023	Hemo-sol	136.26	3.5	0	0	39.84	1.83	2.12	0.02	0	11.68	
MOL000024	alpha-humulene	204.39	5.04	0	0	22.98	1.88	2.08	0.06	0		
MOL000025	伪-Longipinene	204.39	4.12	0	0	53.26	1.83	2.14	0.12	0	12.06	
MOL000026	stigmast-22E-en-3beta-ol	414.79	7.89	1	1	10.39	1.43	1.13	0.75	0		
MOL000027	alpha-Curcumene	202.37	5.34	0	0	4.68	1.93	1.99	0.06	0		
MOL000028	伪-Amyrin	426.8	7.35	1	1	39.51	1.42	1.28	0.76	0	3.83	
MOL000029	beta-Humulene	204.39	5.09	0	0	26.87	1.82	2.01	0.06	0		
MOL000030	(1R)-2-methyl-1-phenylprop-2-en-1-ol	148.22	2.32	1	1	75.1	1.27	1.38	0.03	0	-2.13	
MOL000031	(3S)-3-[(1R)-1,5-dimethylhex-4-enyl]-6-methylenecyclohexene	204.39	5.14	0	0	19.86	1.88	1.99	0.06	0		
MOL000032	beta-Eudesmol	222.41	3.72	1	1	26.09	1.32	1.38	0.1	0		
MOL000033	(3S,8S,9S,10R,13R,14S,17R)-10,13-dimethyl-17-[(2R,5S)-5-propan-2-yloctan-2-yl]-2,3,4,7,8,9,11,12,14,15,16,17-dodecahydro-1H-cyclopenta[a]phenanthren-3-ol	428.82	8.54	1	1	36.23	1.45	1.09	0.78	0	5.22	
MOL000034	2-[(1R,3S,4S)-3-isopropenyl-4-methyl-4-vinylcyclohexyl]propan-2-ol	222.41	3.7	1	1	19.03	1.37	1.46	0.07	0		
MOL000035	beta-Selinene	204.39	4.81	0	0	24.39	1.83	2.12	0.08	0		
MOL000036	beta-caryophyllene	204.39	4.75	0	0	29.7	1.83	2.07	0.09	0		
MOL000037	纬-elemene	204.39	4.93	0	0	23.79	1.87	2.1	0.06	0		
MOL000038	Akridin	179.23	3.35	0	1	33.71	1.63	1.53	0.1	0	6.82	
MOL000039	(1S,2R,4R)-Neoiso-dihydrocarveol	154.28	2.58	1	1	52.4	1.38	1.69	0.03	0	11.46	
MOL000040	Scopoletol	192.18	1.62	1	4	27.77	0.71	0.3	0.08	0		BaiZhu
MOL000041	PHA	165.21	0.96	3	3	41.62	0.36	0.22	0.04	0	4.62	
MOL000042	LPG	89.11	-0.6	3	3	87.69	-0.34	-0.77	0.01	0	11.58	
MOL000043	atractylenolide i	230.33	3.32	0	2	37.37	1.3	1.29	0.15	0	7.1	
MOL000044	atractylenolideII	232.35	3.57	0	2	47.5	1.3	1.37	0.15	0	7.21	
MOL000045	atractylenolide iii	248.35	2.93	1	3	68.11	0.75	0.63	0.17	0	7.17	
MOL000046	atractylone	216.35	4.11	0	1	41.1	1.76	1.85	0.13	0	1.74	
MOL000047	juniper camphor	222.41	3.93	1	1	33.3	1.44	1.61	0.1	0	9.27	

MOL000048	(5E,9Z)-3,6,10-trimethyl-4,7,8,11-tetrahydrocyclodeca[b]furan	216.35	4.63	0	1	43.17	1.77	1.83	0.1	0	-1.4
MOL000049	3尾-acetoxyatractylone	274.39	3.39	0	3	54.07	1.13	1.08	0.22	0	-1.31
MOL000050	GLY	75.08	-0.98	3	3	48.74	-0.56	-1.03	0	0	11.95
MOL000051	Polymannose	180.18	-2.68	5	6	1.76	-1.94	-4.53	0.03	0	
MOL000052	Gulutamine	147.15	-0.92	4	5	6.66	-1.05	-1.97	0.02	0	
MOL000053	Methose	180.18	-2.69	5	6	1.68	-1.8	-4.58	0.03	0	
MOL000054	L-	174.24	-1.11	7	6	47.64	-0.49	-1.04	0.03	0	0.85
MOL000055	L-Lysin	146.22	-0.68	5	4	29.33	-0.66	-1.44	0.02	0	
MOL000056	DTY	181.21	0.69	4	4	57.55	-0.1	-0.49	0.05	0	0.96
MOL000057	DIBP	278.38	3.92	0	4	49.63	0.85	0.68	0.13	0	3.94
MOL000058	2-[(2R,5S,6S)-6,10-dimethylspiro[4.5]dec-9-en-2-yl]propan-2-ol	222.41	3.67	1	1	38.59	1.34	1.42	0.09	0	7.61
MOL000059	uridine	244.23	-2.45	4	8	10.49	-1.14	-1.61	0.11	0	
MOL000060	selina-4(14),7(11)-dien-8-one	218.37	3.81	0	1	32.31	1.42	1.57	0.1	0	7.68
MOL000061	Prolinum	115.15	-0.06	2	3	77.57	0.22	0.29	0.01	0	11.13
MOL000062	biatractylolide	462.68	6.68	0	4	17.45	0.83	0.37	0.81	0	
MOL000063	ATRACTYLODES MACROCEPHALA	462.68	6.68	0	4	14.6	0.88	0.6	0.81	0	
MOL000064	D-Serin	105.11	-1.49	4	4	83.59	-0.94	-1.75	0.01	0	11.51
MOL000065	ASI	133.12	-1.25	4	5	79.74	-1.02	-1.53	0.02	0	11.38
MOL000066	alloaromadedrene	204.39	4.22	0	0	53.46	1.83	2.1	0.1	0	12.51
MOL000067	L-Valin	117.17	0.24	3	3	53.33	0.04	-0.14	0.01	0	11.34
MOL000068	L-Ile	131.2	0.7	3	3	59.05	0.06	-0.11	0.02	0	11.21
MOL000069	palmitic acid	256.48	6.37	1	2	19.3	1.09	1	0.1	0	
MOL000070	Ethyl pivaloylacetate	172.25	1.69	0	3	40.52	0.82	0.83	0.03	0	1.92
MOL000071	Istidina	155.18	-1.01	4	4	53.18	-0.25	-0.4	0.03	0	-5.72
MOL000072	8β-ethoxy atractylenolide III	276.41	3.68	0	3	35.95	1.08	1.12	0.21	0	8.34
MOL001212	Loxanol V	214.44	5.53	1	1	14.19	1.27	0.98	0.05	0.14	
MOL001218	Pisol	186.38	4.62	1	1	18.5	1.23	1.06	0.03	0.14	
MOL001312	9-HEXADECENOIC ACID	254.46	5.92	1	2	35.78	1.1	0.85	0.1	0.2	5.61
MOL001392	Methyl myristate	242.45	5.71	0	2	19.68	1.36	1.19	0.08	0.14	
MOL001396	PENTADECYLIC ACID	242.45	5.91	1	2	20.18	1.08	0.88	0.08	0.18	
MOL001641	METHYL LINOLEATE	294.53	6.64	0	2	41.93	1.44	1.08	0.17	0.21	6.05
MOL001706	2,6-dimethyl-3,7-octadiene-2,6-diol	170.28	1.33	2	2	52.4	0.47	0.51	0.03	0.33	6.57
MOL001738	MLI	104.07	-0.44	2	4	21.69	-0.23	-0.25	0.01	0.44	
MOL001817	Methyl stearate	298.57	7.53	0	2	16.8	1.41	1.29	0.16	0.16	

MOL001818	Methyl palmitelaidate	268.49	6.17	0	2	34.61	1.4	1.22	0.12	0.17	5.58
MOL001819	METHYL PENTADECANOATE	256.48	6.16	0	2	18.82	1.37	1.28	0.1	0.17	
MOL001949	panaxynol	244.41	5.68	1	1	42.44	1.52	1.03	0.1	0.32	7.14
MOL001965	Dauricine (8CI)	624.84	7.22	1	8	23.65	0.9	0.03	0.37	0.22	
MOL002121	(1S,4E,8E,10R)-4,8,11,11-tetramethylbicyclo[8.1.0]undeca-4,8-diene	204.39	4.7	0	0	21.69	1.86	2.02	0.08	0.26	
MOL002136	neocnidilide	194.3	3.37	0	2	83.83	1.23	1.32	0.07	0.28	5.38
MOL002137	OCT	114.26	4.02	0	0	29.72	1.78	2.02	0.01	0.19	
MOL002307	20-Hexadecanoylingenol	586.94	7.38	3	6	28.2	0.3	-0.04	0.68	0.24	
MOL002312	[(3S,4R,5R)-5-[[(2R,3S,4S,5R,6S)-6-(2-acetyl-5-methoxyphenoxy)-3,4,5-trihydroxyoxan-2-yl]methoxy]-3,4-dihydroxyoxolan-3-yl]methyl 3,4,5-trihydroxybenzoate	612.59	-0.79	8	16	5.61	-2.17	-3.07	0.63	0.34	
MOL002323	L-Adenosine	267.28	-2.02	5	8	18.06	-1.42	-1.99	0.18	0.22	
MOL002377	Kaempferol-3-arabofuranoside	418.38	0.19	6	10	2.73	-1.08	-2.01	0.65	0.34	
MOL002526	3691-11-0	204.39	4.99	0	0	23.66	1.86	2.05	0.07	0.25	
MOL002669	Campesteryl ferulate	576.94	9.86	1	4	22.1	1.1	0.06	0.59	0.26	
MOL000269	Elemicin	208.28	2.79	0	3	21.94	1.41	1.28	0.06	0.2	
MOL002879	Diop	390.62	7.44	0	4	43.59	0.79	0.26	0.39	0.28	3.6
MOL000029	beta-Humulene	204.39	5.09	0	0	26.87	1.82	2.01	0.06	0	
MOL000449	Stigmasterol	412.77	7.64	1	1	43.83	1.44	1	0.76	0.22	5.57
MOL003346	Psuedohypericin	520.46	3.8	7	9	16.94	-0.2	-1.61	0.07	0.34	
MOL000358	beta-sitosterol	414.79	8.08	1	1	36.91	1.32	0.99	0.75	0.23	5.36
MOL000036	beta-caryophyllene	204.39	4.75	0	0	29.7	1.83	2.07	0.09	0	
MOL003648	Inermin	284.28	2.44	1	5	65.83	0.91	0.36	0.54	0.3	11.73
MOL003845	Folinic acid	473.5	-0.04	8	14	23.6	-1.7	-2.58	0.74	0.34	
MOL003902	methyl (Z)-icos-11-enoate	324.61	8	0	2	29.49	1.41	1.11	0.23	0.16	
MOL004100	N-Salicylidene-salicylamine	227.28	2.9	2	3	95.46	1.12	0.48	0.11	0	3.84
MOL004174	epsilon-Cadinene	204.39	4.85	0	0	16.41	1.82	2.12	0.08	0.29	
MOL000422	kaempferol	286.25	1.77	4	6	41.88	0.26	-0.55	0.24	0	14.74
MOL004237	末-elemene	204.39	4.73	0	0	25.99	1.84	2.1	0.06	0.31	
MOL004275	(1R,4E,7E,11R)-1,5,9,9-tetramethyl-12-oxabicyclo[9.1.0]dodeca-4,7-diene	220.39	3.8	0	1	23.66	1.58	1.77	0.1	0.29	

MOL004492	Chrysanthemaxanthin	584.96	8.24	2	3	38.72	0.51	-0.98	0.58	0.3	17.47
MOL004498	12-O-Nicotinoylisolineolone	469.63	1.32	3	7	20.7	-0.54	-1.19	0.83	0.31	
MOL004647	TDA	214.39	5	1	2	22.32	1.04	0.89	0.05	0.21	
MOL005155	ginsenoside Ro_qt	455.77	5.74	1	3	17.62	0.38	0.05	0.76	0.04	
MOL005269	(+)-Maalioxide	222.41	3.47	0	1	55.93	1.59	1.93	0.13	0.22	16.64
MOL005270	n-Heptadecanol	256.53	6.9	1	1	12.97	1.31	0.98	0.09	0.15	
MOL005271	1-HEXADECYNE	222.46	7.86	0	0	3.94	1.89	1.87	0.06	0.23	
MOL005272	13-Tetradecenyl acetate	254.46	5.52	0	2	36.76	1.36	1.06	0.1	0.2	5.71
MOL005273	16-Oxoseratenediol	456.78	5.52	2	3	15.1	0.4	-0.25	0.75	0.24	
MOL005274	Neohexane	86.2	2.65	0	0	37.81	1.77	2.16	0.01	0.25	11.05
MOL005275	2,3,4-Trimethyldecane	184.41	5.69	0	0	16.15	1.82	2.08	0.03	0.21	
MOL005276	2,3,8-Trimethyldecane	184.41	5.69	0	0	5.51	1.79	1.95	0.03	0.22	
MOL005277	2,6,10,15-tetramethylheptadecane	296.65	9.13	0	0	13.73	1.85	1.86	0.13	0.19	
MOL005278	2-METHYLTRIDECANE	198.44	6.55	0	0	5.75	1.81	1.9	0.04	0.18	
MOL005279	ginsenoside-Rh1	638.98	2.87	7	9	3.86	-1.17	-2.14	0.57	0.25	
MOL005280	ginsenoside-Rh1_qt	476.82	4.62	4	4	20.13	-0.02	-0.9	0.78	0.24	
MOL005281	20(S)-Ginsenoside-Rh1	638.98	2.87	7	9	3.94	-1.1	-2.04	0.57	0.25	
MOL005282	20(S)-Ginsenoside-Rh1_qt	476.82	4.62	4	4	20.13	0.17	-0.56	0.78	0.24	
MOL005283	20(S)-ginsenoside-Rg2	785.14	2.02	9	13	10.09	-1.94	-2.78	0.26	0.23	
MOL005284	(3S,5R,6S,8R,9R,10R,12R,13R,14R,17S)-17-[(2S)-2-hydroxy-6-methylhept-5-en-2-yl]-4,4,8,10,14-pentamethyl-2,3,5,6,7,9,11,12,13,15,16,17-dodecahydro-1H-cyclopenta[a]phenanthrene-3,6,12-triol	476.82	4.62	4	4	20.13	0.01	-0.82	0.78	0.25	
MOL005285	20(s)-protopanaxadiol	460.82	5.79	3	3	29.69	0.57	-0.07	0.77	0.23	
MOL005286	20(R)-ginsenoside Rg2	785.14	2.02	9	13	10.09	-1.96	-2.95	0.26	0.27	
MOL005287	20-(S)-Ginsenoside-Rg3	785.14	2.3	9	13	13.69	-1.65	-2.47	0.22	0.24	
MOL005288	20-(S)-Ginsenoside-Rg3_qt	460.82	5.79	3	3	29.69	0.55	-0.12	0.77	0.24	
MOL005289	3,4-Dimethylheptane	128.29	4.07	0	0	46.67	1.78	2.05	0.01	0.24	11.02
MOL005290	3,5-Dimethyl-p-anisic acid	180.22	2.39	1	3	61.11	0.75	0.56	0.05	0.34	2.11
MOL005291	3-O-beta-D-Glucuronopyranosyl gypsogenin	646.9	4.05	5	10	8.68	-0.78	-1.37	0.32	0.27	
MOL005292	3-O-beta-D-Glucuronopyranosyl gypsogenin_qt	470.76	5.59	2	4	23.72	0.22	-0.4	0.75	0.26	

MOL005293	3-Ethyl-3-methylheptane	142.32	4.48	0	0	37.33	1.81	2.16	0.02	0.23	10.45
MOL005294	3-methylheptane	114.26	3.82	0	0	37.1	1.8	2.18	0.01	0.22	10.55
MOL005295	3-methylundecane	170.38	5.64	0	0	6.57	1.79	1.96	0.02	0.19	
MOL005296	4-Methyldodecane	184.41	6.1	0	0	6.39	1.81	2.05	0.03	0.17	
MOL005297	5-Isobutylnonane	184.41	5.89	0	0	6.08	1.82	2.1	0.03	0.2	
MOL005298	5-heptadec-12-enylresorcinol	346.61	8.64	2	2	3.29	1.38	0.79	0.32	0.25	
MOL005299	5-methyl-tetradecane	212.47	7.01	0	0	16.15	1.83	2.02	0.05	0.18	
MOL005300	6'-Malonylginsenoside Rd1	1195.51	-1.03	15	26	6.7	-4.21	-5.39	0.03	0.29	
MOL005301	6'-Malonylginsenoside Rd1_qt1	460.82	5.79	3	3	29.69	0.56	-0.13	0.77	0.25	
MOL005302	7-(beta-Xylosyl)cephalomannine	962.15	1.97	6	18	27.33	-1.67	-2.25	0.17	0.32	
MOL005303	7-Tetradecyne	194.4	6.44	0	0	20.07	1.93	1.81	0.04	0.19	
MOL005304	7alpha-L-Rhamnosyl-6-methoxylutcolin	462.44	1.03	6	11	15.03	-0.69	-1.99	0.79	0.29	
MOL005305	Nepetin	316.28	2.05	4	7	26.75	0.37	-0.78	0.31	0.27	
MOL005306	Acetal	118.2	0.78	0	2	26.4	1.25	1.46	0.01	0.23	
MOL005307	Adenosine triphosphate	491.22	-4.3	8	16	8.23	-3.1	-3.75	0.58	0.38	
MOL005308	Aposiopolamine	271.34	1.39	1	4	66.65	0.66	0.4	0.22	0.35	3.54
MOL005309	Araloside A	927.21	1.74	10	18	16.96	-3.32	-3.74	0.06	0.26	
MOL005310	(4aS,6aR,6aS,6bR,8aR,10S,12aR,14bR)-10-hydroxy-2,2,6a,6b,9,9,12a-heptamethyl-1,3,4,5,6,6a,7,8,8a,10,11,12,13,14b-tetradecahydropicene-4a-carboxylic acid	456.78	6.42	2	3	14.36	0.6	0.1	0.76	0.25	
MOL005311	Argininyl-fructosyl-glucose	498.56	-4.63	13	16	0.74	-3.12	-3.75	0.62	0.25	
MOL005312	Argininyl-fructosyl-glucose_qt	336.4	-2.88	10	11	11.25	-1.74	-2.3	0.22	0.25	
MOL005313	5-[(3aS,6R,6aR)-2-keto-1,3,3a,4,6,6a-hexahydrothieno[3,4-d]imidazol-6-yl]valeric acid	244.35	0.65	3	5	75.75	-0.04	-0.28	0.1	0.32	3.72
MOL005314	Celabenzine	379.55	2.29	2	5	101.88	0.77	0.05	0.49	0.35	8.15
MOL005315	(R)-()-Citronellal	154.28	3.02	0	1	50.78	1.37	1.61	0.02	0.28	5.35
MOL005316	MAV	194.16	-2.31	5	7	56.17	-2.14	-4.77	0.06	0.34	11.27
MOL005317	Deoxyharringtonine	515.66	3.13	1	9	39.27	0.19	-0.25	0.81	0.23	7.9
MOL005318	Dianthramine	289.26	2.05	5	7	40.45	-0.23	-0.97	0.2	0.42	5.14
MOL005319	Ditertbutyl phthalate	278.38	3.4	0	4	43.67	1.13	1.13	0.13	0.33	-1.91

MOL005320	arachidonate	304.52	6.41	1	2	45.57	1.27	0.58	0.2	0.26	7.56	
MOL005321	Frutinone A	264.24	2.7	0	4	65.9	0.89	0.46	0.34	0.47	19.1	
MOL005322	Gamma-Selinene	204.39	4.95	0	0	22.58	1.84	2.11	0.08	0.26		
MOL005323	ginsenoside La	783.12	1.84	8	13	17.74	-1.9	-3.25	0.14	0.24		
MOL005324	ginsenoside La_qt	458.8	5.33	2	3	15.7	0.65	0.04	0.78	0.24		
MOL005325	ginsenoside Ro	957.24	1.23	11	19	1.98	-2.86	-3.92	0.05	0.26		
MOL005326	Ginsenoside-Ra0	1271.62	-2.94	18	28	7.3	-5.56	-6.76	0.01	0.22		
MOL005327	Gypnoside V_qt	460.82	5.79	3	3	29.69	0.3	-0.57	0.77	0.25		
MOL005328	Ginsenoside-Ra1	1211.56	-1.92	16	26	7.09	-4.51	-6.41	0.02	0.22		RenShen
MOL005329	Ginsenoside-Ra2	1211.56	-1.92	16	26	7.62	-4.43	-6.28	0.02	0.24		
MOL005330	Ginsenoside-Ra3	1241.59	-2.43	17	27	7.19	-4.67	-6.3	0.02	0.26		
MOL005331	ginsenoside Rb1	1109.46	-1.2	15	23	6.24	-3.99	-5.6	0.04	0.23		
MOL005332	(3R,5R,8R,9R,10R,12R,13R,14R,17S)-17-[(2S)-2-hydroxy-6-methylhept-5-en-2-yl]-4,4,8,10,14-pentamethyl-2,3,5,6,7,9,11,12,13,15,16,17-dodecahydro-1H-cyclopenta[a]phenanthrene-3,12-diol	460.82	5.79	3	3	29.69	0.35	-0.54	0.77	0.25		
MOL005333	ginsenoside-Rb2	1079.43	-0.69	14	22	6.02	-3.92	-5.43	0.04	0.26		
MOL005334	(3S,5R,8R,9R,10R,12R,13R,14R,17S)-17-[(2S)-2-hydroxy-6-methylhept-5-en-2-yl]-4,4,8,10,14-pentamethyl-2,3,5,6,7,9,11,12,13,15,16,17-dodecahydro-1H-cyclopenta[a]phenanthrene-3,12-diol	460.82	5.79	3	3	29.69	0.37	-0.52	0.77	0.24		
MOL005335	Gypenoside LXIX	1079.43	-0.69	14	22	7.73	-3.58	-5.38	0.04	0.24		
MOL005336	ginsenoside-Rc	1079.43	-0.69	14	22	8.16	-3.97	-5.69	0.04	0.25		

MOL005337	(2S,3R,4S,5S,6R)-2-[(2S)-2-[(3S,5R,8R,9R,10R,12R,13R,14R,17S)-3-[(2R,3R,4S,5S,6R)-4,5-dihydroxy-6-(hydroxymethyl)-3-[(2S,3R,4S,5S,6R)-3,4,5-trihydroxy-6-(hydroxymethyl)oxan-2-yl]oxyoxan-2-yl]oxy-12-hydroxy-4,4,8,10,14-pentamethyl-	947.3	0.55	12	18	5.5	-3.17	-4.47	0.09	0.24	
MOL005338	Ginsenoside Re	947.3	0.27	12	18	4.27	-3.2	-4.39	0.12	0.25	
MOL011400	ginsenoside rf	801.14	1.13	10	14	17.74	-2.23	-3.35	0.24	0.27	
MOL005340	(3S,5R,6S,8R,9R,10R,12R,13R,14R,17S)-17-[(2R)-2-hydroxy-6-methylhept-5-en-2-yl]-4,4,8,10,14-pentamethyl-2,3,5,6,7,9,11,12,13,15,16,17-dodecahydro-1H-cyclopenta[a]phenanthrene-3,6,12-triol	476.82	4.62	4	4	20.13	0.21	-0.43	0.78	0.24	
MOL005341	Sanchinoside C1	801.14	1.13	10	14	10.04	-2.27	-3.5	0.28	0.24	
MOL005342	Ginsenoside-Rg3	785.14	2.3	9	13	17.75	-2.02	-2.75	0.22	0.25	
MOL005343	Ginsenoside-Rg3_qt	460.82	5.79	3	3	29.69	0.31	-0.58	0.77	0.26	
MOL005344	ginsenoside rh2	622.98	4.04	6	8	36.32	-0.51	-1.38	0.56	0.24	11.08
MOL005345	(2R,3S,4S,5R,6R)-2-(hydroxymethyl)-6-[[(3S,5R,8R,9R,10R,12R,13R,14R,17S)-12-hydroxy-4,4,8,10,14-pentamethyl-17-[(2Z)-6-methylhepta-2,5-dien-2-yl]-2,3,5,6,7,9,11,12,13,15,16,17-dodecahydro-1H-cyclopenta[a]phenanthren-3-yl]oxy]oxane-3,4,5-triol	604.96	5.01	5	7	12.09	-0.35	-1.11	0.59	0.25	
MOL005346	Ginsenoside-Rh3_qt	442.8	6.76	2	2	13.09	0.97	0.42	0.76	0.26	
MOL005347	Ginsenoside-Rh4	620.96	3.84	6	8	5.22	-0.73	-1.65	0.6	0.25	
MOL005348	Ginsenoside-Rh4_qt	458.8	5.59	3	3	31.11	0.5	-0.18	0.78	0.25	6.97

MOL005349	Ginsenoside-Rs1	1121.47	-0.31	13	23	6.27	-3.69	-5.37	0.04	0.24	
MOL005350	Ginsenoside-Rs2	1121.47	-0.31	13	23	8.14	-4.03	-5.58	0.04	0.24	
MOL005351	Ginsenoyne A	258.39	4.3	1	2	66.22	0.99	0.02	0.13	0.36	5.14
MOL005352	Ginsenoyne B	294.85	4.74	2	2	39.79	0.79	-0.1	0.13	0.42	5.82
MOL005353	Ginsenoyne C	276.41	3.53	3	3	43.38	0.15	-0.81	0.13	0.36	5.5
MOL005354	Ginsenoyne D	262.43	4.94	1	2	19.32	1.07	0.28	0.13	0.31	
MOL005355	Ginsenoyne E	258.39	4.93	0	2	36.53	1.05	0.46	0.13	0.37	6.72
MOL005356	Girinimbin	263.36	4.6	1	1	61.22	1.72	1.22	0.31	0.33	8.17
MOL005357	Gomisin B	514.62	2.73	1	9	31.99	0.6	0.18	0.83	0.19	7.81
MOL005358	L-erythro-isocitric acid	192.14	-1.34	4	7	32.95	-1.46	-1.71	0.04	0.41	11.65
MOL005359	D-erythro-Isocitric acid	192.14	-1.34	4	7	65.43	-1.67	-2.22	0.04	0.4	11.43
MOL005360	malkangunin	432.56	1.84	2	7	57.71	0.22	-0.17	0.63	0.3	4.09
MOL005361	Malonylginsenoside Rc	1165.48	-0.52	14	25	7.84	-4.27	-5.65	0.03	0.25	
MOL005362	Malonylginsenoside Rc_qt1	460.82	5.79	3	3	29.69	0.45	-0.44	0.77	0.25	
MOL005363	Malonylginsenoside Rd	1033.35	0.71	12	21	8.84	-3.72	-4.76	0.07	0.28	
MOL005364	Malonylginsenoside Rd_qt	460.82	5.79	3	3	29.69	0.47	-0.23	0.77	0.26	
MOL005365	MAL	342.34	-4.26	8	11	1.8	-2.7	-6.56	0.24	0.21	
MOL005366	Malvic acid	280.5	6.44	1	2	30.99	1.22	0.81	0.15	0.21	3.74
MOL005367	GUP	180.18	-2.51	5	6	43.04	-1.82	-4.46	0.04	0.26	11.1
MOL005368	Methyl tricosanoate	368.72	9.81	0	2	14.61	1.43	1.08	0.33	0.16	
MOL005369	Mycosinol	214.23	1.46	1	3	82.12	0.87	-0.05	0.09	0.42	5.67
MOL005370	NN-Dimethyldecanamide	199.38	3.44	0	2	55.5	1.51	1.72	0.04	0.14	3.58
MOL005371	Nonacosanediol-6,8	188.35	2.79	2	2	17.79	0.56	0.2	0.03	0.22	
MOL005372	notoginsenoside R2	771.11	1.64	9	13	17.74	-2.22	-3.12	0.28	0.25	
MOL005373	notoginsenoside R2_qt	476.82	4.62	4	4	20.13	0.2	-0.49	0.78	0.24	
MOL005374	Notoginsenoside R6	963.3	-0.62	13	19	4.7	-3.46	-4.39	0.12	0.24	
MOL005375	Stearyl acetate	312.6	7.74	0	2	16.27	1.39	1.06	0.19	0.15	
MOL005376	Panaxadiol	460.82	5.46	2	3	33.09	0.82	0.23	0.79	0.22	6.34
MOL007500	panaxatriol	476.82	4.29	3	4	15.42	0.52	-0.07	0.79	0.22	
MOL005378	Panaxytriol	278.43	3.92	3	3	33.76	0.06	-0.98	0.13	0.33	3.74
MOL005379	Pancratistatin	325.3	-1.94	6	9	13.13	-1.17	-1.79	0.46	0.32	
MOL005380	Pandamine	552.79	3.87	4	9	16.15	0.11	-0.34	0.79	0.35	
MOL005381	2-Formylpyrrole	95.11	0.97	1	1	41.58	1.12	1.44	0.01	0.14	4.34
MOL005382	Ramalic acid	346.36	3.77	3	7	6	0.35	-0.13	0.3	0.33	
MOL005383	Methylselenocysteine	182.1	-0.27	3	3	35.74	0.01	-0.13	0.01	0.43	11.69
MOL005384	suchilactone	368.41	3.73	0	6	57.52	0.82	0.28	0.56	0.28	9.03
MOL005385	Suffruticoside A_qt1	302.26	-0.93	6	9	13.9	-1.35	-2.1	0.2	0.35	
MOL005386	Vulgarin	264.35	1.31	1	4	29.21	0.03	-0.27	0.2	0.36	
MOL006651	Trifolirhizin	446.44	0.54	4	10	7.62	-0.83	-1.58	0.79	0.28	
MOL005388	Undecane, 3,6-dimethyl	184.41	5.89	0	0	12.85	1.79	2.03	0.03	0.21	
MOL005389	PANGAMIC ACID	436.62	1.11	5	10	10.08	-0.92	-0.96	0.32	0.31	

MOL005390	3-[[(2S)-2,4-dihydroxy-3,3-dimethylbutanoyl]amino]propanoic acid	219.27	-0.98	4	6	21.29	-0.85	-1.25	0.06	0.29	
MOL005391	(Z,Z)-alpha-farnesene	204.39	5.46	0	0	8.47	1.92	1.85	0.05	0.36	
MOL005392	alpha-Guttiferin	452.59	6.41	3	6	4.43	0.59	0.24	0.65	0.3	
MOL000908	beta-elemene	204.39	4.79	0	0	25.63	1.84	2.07	0.06	0.33	
MOL005394	(Z)-2-methyl-5-[(1S,2R,4R)-2-methyl-3-methylene-2-	220.39	3.66	1	1	35.28	1.28	1.25	0.09	0.27	1.94
MOL000035	beta-Selinene	204.39	4.81	0	0	24.39	1.83	2.12	0.08	0	
MOL005396	cis-Widdrol alpha-epoxide	238.41	2.6	1	2	69.04	1.07	1	0.15	0.22	6.86
MOL005397	Dammarane	414.84	9.53	0	0	19.73	1.81	1.81	0.7	0.22	
MOL005398	alexandrin	576.95	6.34	4	6	20.63	-0.1	-0.79	0.63	0.22	
MOL005399	alexandrin_qt	414.79	8.08	1	1	36.91	1.3	0.88	0.75	0.23	5.53
MOL005400	ginsenoside Rg5	767.12	3.31	8	12	6.15	-1.92	-2.83	0.23	0.26	
MOL005401	ginsenoside Rg5_qt	442.8	6.8	2	2	39.56	0.88	0.21	0.79	0.24	5.65
MOL005402	Methyl margarate	284.54	7.08	0	2	17.41	1.37	1.25	0.14	0.17	
MOL005403	oleanane	412.82	8.66	0	0	6.69	1.83	1.92	0.76	0.22	
MOL005404	p-Glucosyloxymandelonitrile	298.37	-0.47	5	6	12.12	-0.92	-1.61	0.18	0.27	
MOL000628	darutoside	574.93	5.89	4	6	21.32	-0.26	-0.96	0.63	0.22	
MOL000066	alloaromadedrene	204.39	4.22	0	0	53.46	1.83	2.1	0.1	0	12.51
MOL000676	DBP	278.38	4.2	0	4	64.54	0.8	0.56	0.13	0.34	5.41
MOL000069	palmitic acid	256.48	6.37	1	2	19.3	1.09	1	0.1	0	
MOL000749	Linoleic	280.5	6.39	1	2	41.9	1.23	0.81	0.14	0.23	5.27
MOL000787	Fumarine	353.4	2.95	0	6	59.26	0.56	-0.13	0.83	0.3	23.46
MOL000864	MYS	212.47	7.22	0	0	13.98	1.81	1.92	0.05	0.15	
MOL000874	paeonol	166.19	1.29	1	3	28.79	0.93	0.84	0.04	0.32	
MOL000879	methyl palmitate	270.51	6.62	0	2	18.09	1.37	1.18	0.12	0.14	
MOL000886	tetradecane	198.44	6.76	0	0	15.94	1.79	1.78	0.04	0.15	
MOL000935	Hepanal	204.39	4.36	0	0	53.83	1.86	2.17	0.1	0.24	11.92
MOL000942	(1R,4S,4aR,8aR)-4-isopropyl-1,6-dimethyl-3,4,4a,7,8,8a-hexahydro-2H-naphthalen-1-ol	222.41	3.78	1	1	31.67	1.32	1.3	0.09	0.24	6.87
MOL000968	beta-Bisabolene	204.39	5.33	0	0	29.59	1.88	2.13	0.06	0.29	
MOL000105	protocatechuic acid	154.13	0.9	3	4	25.37	0.1	-0.17	0.04	0.43	
MOL001097	o-xylene	106.18	2.8	0	0	45.55	1.85	2.08	0.01	0.41	11.86
MOL001098	m-xylene	106.18	2.8	0	0	47.43	1.83	2.02	0.01	0.39	11.82
MOL001099	p-xylene	106.18	2.8	0	0	48.74	1.83	2.06	0.01	0.39	11.71
MOL000118	(L)-alpha-Terpineol	154.28	2.42	1	1	48.8	1.39	1.72	0.03	0.27	11.35
MOL000012	Arachic acid	312.6	8.19	1	2	16.66	1.18	1.09	0.19	0.18	

MOL001484	Inermine	284.28	2.44	1	5	75.18	0.89	0.4	0.54	0.3	11.72
MOL001543	Vicenin-2	594.57	-2.45	11	15	3.42	-3.15	-3.87	0.78	0.32	
MOL001599	伪-cubebol	208.38	3.28	1	1	64.81	1.32	1.43	0.09	0.26	7.24
MOL001696	Morusin	420.49	4.94	3	6	11.52	0.51	-0.22	0.76	0.31	
MOL001737	ICO	161.17	1.73	2	2	33.86	0.82	0.63	0.05	0.3	-7.7
MOL001789	isoliquiritigenin	256.27	2.9	3	4	85.32	0.44	-0.41	0.15	0.46	17.66
MOL001792	DFV	256.27	2.57	2	4	32.76	0.51	-0.29	0.18	0.42	17.89
MOL001850	Izoforon	138.23	2.06	0	1	44.98	1.28	1.66	0.03	0.32	11.4
MOL000211	Mairin	456.78	6.52	2	3	55.38	0.73	0.22	0.78	0.26	8.87
MOL002137	OCT	114.26	4.02	0	0	29.72	1.78	2.02	0.01	0.19	
MOL002166	ISOHEPTANE	100.23	3.36	0	0	59.94	1.81	2.25	0.01	0.22	10.67
MOL002198	Heptan	100.23	3.57	0	0	41.8	1.77	2.04	0	0.2	10.36
MOL002311	Glycyrol	366.39	4.85	2	6	90.78	0.71	-0.2	0.67	0.28	9.85
MOL000239	Jaranol	314.31	2.09	2	6	50.83	0.61	-0.22	0.29	0.29	15.5
MOL002547	21987_FLUKA	136.26	2.68	0	0	40.92	1.84	2.14	0.04	0.26	11.22
MOL002565	Medicarpin	270.3	2.66	1	4	49.22	1	0.53	0.34	0.31	8.46
MOL000263	oleanolic acid	456.78	6.42	2	3	29.02	0.59	0.07	0.76	0.25	
MOL002678	EB	106.18	2.77	0	0	49.38	1.83	2.15	0.01	0.38	11.76
MOL002693	nicotiflorin	594.57	-1.18	9	15	3.64	-1.77	-2.55	0.73	0.3	
MOL002844	Pinocembrin	256.27	2.57	2	4	64.72	0.61	0.12	0.18	0.43	17.96
MOL002850	butylated hydroxytoluene	220.39	4.85	1	1	40.02	1.75	1.8	0.07	0.3	10.36
MOL002943	BuOH	74.14	0.97	1	1	22.02	0.94	1.05	0	0.21	
MOL003218	Neouralenol	370.38	3.36	5	7	12.76	0.24	-0.75	0.46	0.37	
MOL000354	isorhamnetin	316.28	1.76	4	7	49.6	0.31	-0.54	0.31	0.32	14.34
MOL000359	sitosterol	414.79	8.08	1	1	36.91	1.32	0.87	0.75	0.22	5.37
MOL003656	Lupiwighteone	338.38	3.92	3	5	51.64	0.68	-0.23	0.37	0.36	15.63
MOL003662	7,4'-Dihydroxyflavone	254.25	2.6	2	4	19.18	0.56	-0.16	0.18	0.43	
MOL003686	Narcissoside	624.6	-1.19	9	16	5.09	-2.14	-2.74	0.65	0	
MOL003896	7-Methoxy-2-methyl isoflavone	266.31	3.36	0	3	42.56	1.16	0.56	0.2	0.33	16.89
MOL000392	formononetin	268.28	2.58	1	4	69.67	0.78	0.02	0.21	0	17.04
MOL003985	2-Caren-10-al	150.24	2.04	0	1	44.74	1.37	1.63	0.05	0.28	11.47
MOL000040	Scopoletol	192.18	1.62	1	4	27.77	0.71	0.3	0.08	0	
MOL000415	rutin	610.57	-1.45	10	16	3.2	-1.93	-2.75	0.68	0	
MOL000417	Calycosin	284.28	2.32	2	5	47.75	0.52	-0.43	0.24	0	17.1
MOL000422	kaempferol	286.25	1.77	4	6	41.88	0.26	-0.55	0.24	0	14.74
MOL004328	naringenin	272.27	2.3	3	5	59.29	0.28	-0.37	0.21	0.4	16.98
MOL000437	Hirsutrin	464.41	-0.59	8	12	1.86	-1.66	-2.31	0.77	0	
MOL000445	8-Prenylwighteone	406.51	5.78	3	5	23.22	0.93	0	0.54	0.33	
MOL004589	Methylheptane	114.26	3.82	0	0	28.65	1.79	2.12	0.01	0.21	
MOL000467	Castanin	298.31	2.57	1	5	23.54	0.77	-0.1	0.27	0	
MOL004723	beta-Terpinene	136.26	3.5	0	0	42.29	1.85	2.12	0.02	0.29	11.21

MOL000475	anethole	148.22	2.77	0	1	32.49	1.75	1.81	0.03	0	1.68
MOL004801	2',7-Dihydroxy-4'-methoxyisoflavan-7-O-尾-d-glucopyranoside	434.48	0.59	5	9	10.46	-1.02	-1.82	0.73	0.31	
MOL004802	(E)-1-butoxyhex-2-ene	156.3	3.31	0	1	41.72	1.5	1.56	0.02	0	6.68
MOL004803	3-Hydroxyglabrol	408.53	5.73	3	5	4.73	0.48	-0.31	0.58	0.33	
MOL004804	18beta-glycyrrhetinic acid	470.76	5.49	2	4	22.05	0.1	-0.53	0.74	0	
MOL004805	(2S)-2-[4-hydroxy-3-(3-methylbut-2-enyl)phenyl]-8,8-dimethyl-2,3-dihydropyrano[2,3-f]chromen-4-one	390.51	5.48	1	4	31.79	1	0.25	0.72	0.35	14.82
MOL004806	euchrenone	406.56	6.35	1	4	30.29	1.09	0.39	0.57	0	15.89
MOL004807	glucuronic acid	194.16	-2.31	5	7	46.18	-2.05	-4.58	0.06	0	11.13
MOL004808	glyasperin B	370.43	4.02	3	6	65.22	0.47	-0.09	0.44	0	16.1
MOL004809	glyasperin E	444.51	6.41	2	6	4.12	0.69	-0.41	0.75	0	
MOL004810	glyasperin F	354.38	2.97	3	6	75.84	0.43	-0.15	0.54	0	15.64
MOL004811	Glyasperin C	356.45	4.73	3	5	45.56	0.71	0.07	0.4	0	3.13
MOL004812	glyasperins D	370.48	4.99	2	5	29.91	0.89	0.25	0.43	0	
MOL004813	glyasperins Z	340.45	5	2	4	4.17	1.09	0.53	0.36	0	
MOL004814	Isotrifoliol	298.26	2.99	2	6	31.94	0.53	-0.25	0.42	0	7.91
MOL004815	(E)-1-(2,4-dihydroxyphenyl)-3-(2,2-dimethylchromen-6-yl)prop-2-en-1-one	322.38	3.96	2	4	39.62	0.66	-0.12	0.35	0	16.16
MOL004816	(2R)-1-[2,4-dihydroxy-5-(3-methylbut-2-enyl)phenyl]-2-hydroxy-3-[4-hydroxy-3-(3-methylbut-2-enyl)phenyl]propan-1-one	410.55	5.96	4	5	1.06	0.33	-0.35	0.48	0	
MOL004817	kanzonols K	436.54	5.76	3	6	0.97	0.76	0.05	0.66	0	
MOL004818	kanzonols L	490.64	7.04	3	6	0.98	0.7	0.11	0.78	0	
MOL004819	kanzonols T	440.53	4.41	5	7	17.87	0.05	-0.66	0.67	0	
MOL004820	kanzonols W	336.36	3.63	2	5	50.48	0.63	0.04	0.52	0	0.15
MOL004821	kanzonols X	394.55	6.61	3	4	7.56	1.1	0.34	0.56	0	
MOL004822	(E)-1-(2,4-dihydroxyphenyl)-3-[4-hydroxy-3-(3-methylbut-2-enyl)phenyl]prop-2-en-1-one	324.4	4.76	3	4	1.04	0.55	-0.22	0.27	0	
MOL004823	licoagropin	320.46	5.77	0	2	27.14	1.63	1.08	0.51	0	

MOL004824	(2S)-6-(2,4-dihydroxyphenyl)-2-(2-hydroxypropan-2-yl)-4-methoxy-2,3-dihydrofuro[3,2-g]chromen-7-one	384.41	2.96	3	7	60.25	0	-0.76	0.63	0	4.31
MOL004825	glyinflanin A	408.53	6.05	4	5	1.06	0.69	0.03	0.48	0.38	
MOL005812	naringin	580.59	-0.46	8	14	6.92	-1.99	-3	0.78	0.31	
MOL004827	Semilicoisoflavone B	352.36	2.85	3	6	48.78	0.45	-0.33	0.55	0	17.02
MOL004828	Glepidotin A	338.38	3.9	3	5	44.72	0.79	0.06	0.35	0	16.09
MOL004829	Glepidotin B	340.4	3.88	3	5	64.46	0.46	-0.09	0.34	0	15.98
MOL004830	Octadiene	110.22	3.19	0	0	34.53	1.81	2.07	0.01	0	3.75
MOL004831	(E)-1-[2,4-dihydroxy-3-(3-methylbut-2-enyl)phenyl]-3-[4-hydroxy-3-(3-methylbut-2-enyl)phenyl]prop-2-en-1-	392.53	6.61	3	4	1.02	0.81	0.01	0.45	0.37	
MOL004832	WLN: 4OVR	178.25	3.01	0	2	48.41	1.31	1.42	0.04	0	7.28
MOL004833	Phaseolinisoflavan	324.4	3.95	2	4	32.01	1.01	0.46	0.45	0	2.66
MOL004834	3-(2-hydroxy-4-methoxyphenyl)-2H-chromen-7-ol	270.3	2.96	2	4	4.66	0.89	0.16	0.21	0	
MOL004835	Glypallichalcone	284.33	3.4	1	4	61.6	0.76	0.23	0.19	0	17.01
MOL004836	echinatin	270.3	3.15	2	4	66.58	0.38	-0.18	0.17	0	19.56
MOL004837	Karenzu DK2	224.27	3.16	0	2	62.26	0.94	0.64	0.1	0	33.72
MOL004838	8-(6-hydroxy-2-benzofuranyl)-2,2-dimethyl-5-chromenol	308.35	4.2	2	4	58.44	1	0.34	0.38	0.34	8.71
MOL004839	(1S,2S)-1,2-dimethylcyclopentane	98.21	2.79	0	0	41.78	1.78	2.26	0.01	0	10.93
MOL004840	Liconeolignan	354.43	5.23	2	5	4.41	1	0.04	0.4	0	
MOL004841	Licochalcone B	286.3	2.88	3	5	76.76	0.47	-0.46	0.19	0	17.02
MOL004842	licochalcone C	338.43	5.01	2	4	4.44	0.63	0.05	0.29	0	
MOL004843	licochalconeD	354.43	4.74	3	5	1.01	0.47	0.06	0.34	0	
MOL004844	glabrol	392.53	6.28	2	4	4.25	0.84	0.06	0.54	0	
MOL004845	apioglycyrrhizin	779.03	2.54	7	14	17.8	-1.91	-2.67	0.14	0	
MOL004846	apioglycyrrhizin_qt	470.76	5.49	2	4	23.73	0.1	-0.56	0.74	0	
MOL004847	2,2-	100.23	3.11	0	0	55.33	1.79	2.11	0.01	0	11
MOL004848	licochalcone G	354.43	4.35	3	5	49.25	0.64	-0.04	0.32	0.35	15.75

MOL004849	3-(2,4-dihydroxyphenyl)-8-(1,1-dimethylprop-2-enyl)-7-hydroxy-5-methoxy-coumarin	368.41	4.03	3	6	59.62	0.4	-0.23	0.43	0	0.69
MOL004850	liquoric acid	484.74	4.05	2	5	25.44	-0.01	-0.52	0.55	0	
MOL004851	Licoflavone	322.38	4.46	2	4	18.75	0.82	-0.31	0.33	0	
MOL004852	7-hydroxy-2-[4-hydroxy-3-(3-methylbut-2-enyl)phenyl]-6-(3-methylbut-2-enyl)chromone	390.51	6.32	2	4	4.44	0.88	-0.17	0.56	0	
MOL004853	Licoflavonol	354.38	3.63	4	6	8.75	0.49	-0.3	0.4	0	
MOL004385	Yinyanghuo D	338.38	4.19	3	5	13.99	0.61	-0.53	0.38	0	
MOL004855	Licoricone	382.44	4.16	2	6	63.58	0.53	-0.14	0.47	0	16.37
MOL004856	Gancaonin A	352.41	4.17	2	5	51.08	0.8	0.13	0.4	0	16.82
MOL004857	Gancaonin B	368.41	3.91	3	6	48.79	0.58	-0.1	0.45	0	16.49
MOL004858	Gancaonin C	354.38	2.83	4	6	2.87	0.14	-0.87	0.42	0	
MOL004859	2,3-dimethylhexane	114.26	3.61	0	0	46.24	1.78	2.16	0.01	0	11.01
MOL000486	Prunetin	284.28	2.32	2	5	5.41	0.65	-0.25	0.24	0	
MOL004860	licorice glycoside E	693.71	1.59	7	14	32.89	-2.06	-2.8	0.27	0.31	25.39
MOL004861	Gancaonin D	384.41	2.81	4	7	2.72	-0.11	-0.93	0.51	0	
MOL004862	(2R)-2-[3,4-dihydroxy-5-(3-methylbut-2-enyl)phenyl]-5,7-dihydroxy-8-(3-methylbut-2-enyl)chroman-4-one	424.53	5.74	4	6	1.21	0.51	-0.35	0.63	0	
MOL004863	3-(3,4-dihydroxyphenyl)-5,7-dihydroxy-8-(3-methylbut-2-enyl)chromone	354.38	3.65	4	6	66.37	0.52	-0.13	0.41	0	15.81
MOL004864	5,7-dihydroxy-3-(4-methoxyphenyl)-8-(3-methylbut-2-enyl)chromone	352.41	4.17	2	5	30.49	0.9	0.21	0.41	0	14.99
MOL004865	5,7-dihydroxy-3-(2-hydroxy-4-methoxy-phenyl)-6-(3-methylbut-2-	368.41	3.91	3	6	2.47	0.58	-0.14	0.45	0	
MOL004866	2-(3,4-dihydroxyphenyl)-5,7-dihydroxy-6-(3-methylbut-2-enyl)chromone	354.38	3.92	4	6	44.15	0.48	-0.28	0.41	0	16.77
MOL004867	Gancaonin P	370.38	3.36	5	7	1.41	0.27	-0.4	0.45	0	
MOL004868	Gancaonin Q	406.51	6.05	3	5	8.98	0.86	-0.01	0.6	0	
MOL004869	Gancaonin R	382.54	6.9	4	4	1.26	1.03	0.25	0.37	0	
MOL004870	Gancaonin S	382.54	6.9	4	4	1.26	0.98	0.18	0.38	0	
MOL004871	(3S)-2,3-dimethylpentane	100.23	3.16	0	0	35.57	1.78	2.23	0.01	0	11.19

MOL004872	gancaonin T	398.54	5.45	4	5	1.04	0.5	-0.43	0.53	0		
MOL004873	Gancaonin U	380.52	6.59	4	4	14.53	1.08	0.6	0.53	0		
MOL004874	Gancaonin V	312.39	4.74	4	4	1.24	0.73	0.04	0.34	0		
MOL004875	3-[4,6-dihydroxy-2-methoxy-3-(3-methylbut-2-enyl)phenyl]-7-hydroxy-chromone	368.41	3.91	3	6	2.47	0.43	-0.3	0.44	0		
MOL004876	Glycyram	823.04	2.42	8	16	19.62	-2.66	-2.86	0.11	0		
MOL004877	Licoricidin	424.58	6.59	3	5	0.99	0.96	0.3	0.62	0		
MOL004878	Glycycoumarin	368.41	4.42	3	6	23.56	0.52	-0.24	0.44	0		
MOL004879	Glycyrin	382.44	4.67	2	6	52.61	0.59	-0.13	0.47	0	1.31	
MOL004880	5,6,7,8-Tetrahydro-2,4-dimethylquinoline	161.27	2.97	0	1	49.77	1.64	1.7	0.05	0	-2.84	
MOL004881	(E)-1-[2,4-dihydroxy-3-(3-methylbut-2-enyl)phenyl]-3-(2,4-dihydroxyphenyl)prop-2-en-	340.4	4.49	4	5	1.36	0.5	-0.22	0.3	0.38		
MOL004882	Licocoumarone	340.4	4.98	3	5	33.21	0.84	0.06	0.36	0	9.66	
MOL004883	Licoisoflavone	354.38	3.65	4	6	41.61	0.37	-0.27	0.42	0	16.09	
MOL004884	Licoisoflavone B	352.36	2.85	3	6	38.93	0.46	-0.18	0.55	0	15.73	GanCao
MOL004885	licoisoflavanone	354.38	2.97	3	6	52.47	0.39	-0.22	0.54	0	15.67	
MOL004886	licorice-saponin C2	807.04	3.1	8	15	59.66	-2.28	-2.89	0.11	0	3.56	
MOL004887	licorice-saponin C2_qt	454.76	6.17	2	3	17.33	0.48	-0.3	0.76	0		
MOL004888	licorice-saponin F3	983.18	1.32	10	21	17.68	-2.82	-3.47	0.03	0		
MOL004889	licorice-saponin F3_qt	454.76	5.93	1	3	27.53	0.74	0.07	0.64	0		
MOL004890	(4S)-2,4-dimethylhexane	114.26	3.61	0	0	37.13	1.77	2.19	0.01	0	10.95	
MOL004891	shinpterocarpin	322.38	3.46	1	4	80.3	1.1	0.68	0.73	0.32	6.5	
MOL004892	licorice-saponin G2	839.04	1.33	9	17	6.39	-2.01	-2.84	0.11	0		
MOL004893	licorice-saponin G2_qt	486.76	4.4	3	5	22.78	-0.27	-0.74	0.72	0		
MOL004894	licorice-saponin H2	823.04	2.42	8	16	44.37	-2.08	-2.83	0.11	0	5.09	
MOL004895	licorice-saponin H2_qt	470.76	5.49	2	4	22.91	0.01	-0.48	0.74	0		
MOL004896	licorice-saponin J2	825.06	2.26	9	16	6.25	-2.25	-2.84	0.11	0		
MOL004897	licorice-saponin J2_qt	472.78	5.33	3	4	28.3	0.04	-0.82	0.74	0		
MOL004898	(E)-3-[3,4-dihydroxy-5-(3-methylbut-2-enyl)phenyl]-1-(2,4-dihydroxyphenyl)prop-2-en-	340.4	4.49	4	5	46.27	0.41	-0.4	0.31	0.43	15.24	
MOL004899	licorice-saponin B2	809.06	3.35	8	15	58.55	-2.4	-2.96	0.11	0.3	4.24	
MOL004900	licorice-saponin K2	823.04	2.01	9	16	7.82	-2.64	-3.25	0.11	0		
MOL004901	licorice-saponin K2_qt	470.76	5.08	3	4	27.79	0.05	-0.67	0.75	0		
MOL004902	glycyrrhetol	456.78	5.28	2	3	14.66	0.36	-0.26	0.75	0		
MOL004903	liquiritin	418.43	0.66	5	9	65.69	-1.06	-1.93	0.74	0	17.96	

MOL004904	licopyranocoumarin	384.41	3.04	3	7	80.36	0.13	-0.62	0.65	0	0.08
MOL004905	3,22-Dihydroxy-11-oxo-delta(12)-oleanene-27-alpha-methoxycarbonyl-29-oic acid	512.75	4.37	1	6	34.32	-0.06	-0.75	0.55	0	3.56
MOL004906	Hispaglabridin B	390.51	5	1	4	22.94	1.18	0.62	0.88	0.31	
MOL004907	Glyzaglabrin	298.26	2.1	2	6	61.07	0.34	-0.2	0.35	0	21.2
MOL004908	Glabridin	324.4	3.95	2	4	53.25	0.97	0.36	0.47	0	0.03
MOL004909	glabrolide	468.74	5	1	4	17.46	0.29	-0.48	0.61	0	
MOL004910	Glabranin	324.4	4.42	2	4	52.9	0.97	0.31	0.31	0	16.24
MOL004911	Glabrene	322.38	3.77	2	4	46.27	0.99	0.04	0.44	0	3.63
MOL004912	Glabrone	336.36	3.12	2	5	52.51	0.59	-0.11	0.5	0	16.09
MOL004913	1,3-dihydroxy-9-methoxy-6-benzofurano[3,2-c]chromenone	298.26	2.99	2	6	48.14	0.48	-0.19	0.43	0	8.87
MOL004914	1,3-dihydroxy-8,9-dimethoxy-6-benzofurano[3,2-c]chromenone	328.29	2.98	2	7	62.9	0.4	-0.34	0.53	0	9.32
MOL004915	Eurycarpin A	338.38	3.92	3	5	43.28	0.43	-0.06	0.37	0	17.1
MOL004916	2-methyl-5-propyl -nonane	184.41	5.89	0	0	15.28	1.81	2.03	0.03	0	
MOL004917	glycyroside	562.57	-0.73	6	13	37.25	-1.58	-2.56	0.79	0	14.62
MOL004918	HEX	86.2	3.11	0	0	52.5	1.78	2.14	0	0	10.8
MOL004919	Sextone B	98.21	2.99	0	0	56.2	1.77	2.14	0.01	0	10.85
MOL004920	Methylcyclopentane	84.18	2.53	0	0	55.78	1.79	2.22	0.01	0	10.97
MOL004921	Docosyl caffeate	488.83	11.16	2	4	3.14	1.01	0.31	0.59	0	
MOL004922	2-methyl-6-ethyl decane	184.41	5.89	0	0	5.5	1.81	2	0.03	0	
MOL000391	Ononin	430.44	0.68	4	9	11.52	-0.74	-1.67	0.78	0	
MOL004924	(-)-Medicocarpin	432.46	0.75	4	9	40.99	-0.6	-1.34	0.95	0	13.2
MOL004925	vitexin	432.41	-0.06	7	10	3.05	-1.52	-2.41	0.71	0	
MOL004926	4H-1-Benzopyran-4-one, 2-(4-(beta-D-glucopyranosyloxy)phenyl)-2,3-dihydro-5,7-dihydroxy-, (2S)-	434.43	0.39	6	10	14.03	-1.13	-2.08	0.78	0	
MOL004927	Hispaglabridin A	392.53	5.81	2	4	14.6	1.12	0.43	0.73	0	
MOL004928	violanthin	578.57	-1.56	10	14	4.17	-2.01	-2.77	0.81	0	
MOL004929	Pentadecanol	228.47	5.99	1	1	13.73	1.3	1.06	0.06	0	
MOL004930	Uralenol	370.38	3.36	5	7	8.55	0.35	-0.57	0.46	0	
MOL004931	Uralenol-3-methylether	384.41	3.43	4	7	1.41	0.39	-0.62	0.49	0	
MOL004932	glycyrrhizin	823.04	2.42	8	16	9.06	-2.23	-3.13	0.11	0	
MOL004933	uralsaponin B	823.04	2.42	8	16	7.92	-2.6	-3.17	0.11	0	

MOL004934	Isohexane	86.2	2.9	0	0	56.13	1.77	2.08	0	0	11.03
MOL004935	Sigmoidin-B	356.4	3.89	4	6	34.88	0.42	-0.41	0.41	0	14.49
MOL004936	Uralene	384.41	3.43	4	7	11.7	0.63	-0.22	0.49	0	
MOL004937	uralenneoside	286.26	-0.49	5	8	24.96	-1.01	-1.61	0.17	0	
MOL004938	schaftoside	596.54	-1.5	10	16	7.88	-2.46	-3.48	0.75	0	
MOL004939	Nortangeretin	302.25	1.8	5	7	17.9	0.24	-0.68	0.27	0	
MOL004940	neoliquiritin	418.43	0.66	5	9	13.01	-1.08	-1.95	0.71	0	
MOL004941	(2R)-7-hydroxy-2-(4-hydroxyphenyl)chroman-4-one	256.27	2.57	2	4	71.12	0.41	-0.25	0.18	0	18.09
MOL004942	(E)-dodec-2-ene	168.36	5.4	0	0	17.74	1.83	2	0.02	0	
MOL004943	neoisoliquiritin	418.43	1	6	9	21.18	-1.41	-2.05	0.58	0	
MOL004944	Cyclobutanol, 1-ethyl-	100.18	1.32	1	1	93.23	1.13	1.35	0.02	0	11.08
MOL004945	(2S)-7-hydroxy-2-(4-hydroxyphenyl)-8-(3-methylbut-2-enyl)chroman-4-one	324.4	4.42	2	4	36.57	0.72	-0.04	0.32	0	17.95
MOL004946	2-Tetradecanone	212.42	4.99	0	1	17.71	1.46	1.6	0.05	0	
MOL004947	Isoviolanthin	578.57	-1.56	10	14	18.79	-2.43	-3.14	0.81	0	
MOL004948	Isoglycyrol	366.39	4.36	1	6	44.7	0.91	0.05	0.84	0	6.69
MOL004949	Isolicoflavonol	354.38	3.63	4	6	45.17	0.54	-0.42	0.42	0	15.55
MOL004950	isoglycycoumarin	368.41	3.92	2	6	22.09	0.55	-0.16	0.6	0	
MOL004951	Isoliquiritin	418.43	1	6	9	8.61	-1.36	-1.93	0.6	0	
MOL004952	licuraside	550.56	-0.41	8	13	5.25	-1.92	-3	0.77	0	
MOL004953	Liquiritin apioside	550.56	-0.75	7	13	29.23	-1.88	-2.69	0.82	0.31	
MOL004954	isograbrol	392.53	6.28	2	4	11.04	0.87	-0.07	0.5	0	
MOL004955	isoglabrolide	468.74	5.15	1	4	14.77	0.32	-0.16	0.62	0	
MOL004956	Isoononin	430.44	0.68	4	9	8.29	-1	-1.57	0.79	0	
MOL004957	HMO	268.28	2.58	1	4	38.37	0.79	0.25	0.21	0	16.56
MOL004958	Isoschaftoside	564.54	-1.94	10	14	17.38	-2.62	-3.36	0.83	0	
MOL004959	1-Methoxyphaseollidin	354.43	4.25	2	5	69.98	1.01	0.48	0.64	0	9.53
MOL004960	22尾-acetylglabric acid	528.8	4.77	2	6	17.76	-0.21	-0.98	0.64	0	
MOL004961	Quercetin der.	330.31	1.82	3	7	46.45	0.39	-0.44	0.33	0	16.61
MOL004962	24-Hydroxy-11-deoxyglycyrrhetic acid	458.75	5.13	3	4	17.57	0.26	-0.36	0.76	0	
MOL004963	24-Hydroxyglycyrrhetic acid	486.76	4.4	3	5	24.17	-0.1	-0.8	0.72	0	
MOL004964	(Z)-1-(2,4-dihydroxyphenyl)-3-phenylprop-2-en-1-one	240.27	3.17	2	3	73.18	0.57	0.14	0.12	0	20.35
MOL004965	3'(纬,纬-dimethylallyl)-kievitone	424.53	5.63	4	6	1.21	0.51	-0.15	0.63	0	

MOL004966	3'-Hydroxy-4'-O-Methylglabridin	354.43	3.93	2	5	43.71	1	0.73	0.57	0	-0.61
MOL004967	3,3-Dimethylpentane	100.23	3.11	0	0	41.97	1.75	2.1	0.01	0	11.09
MOL004968	3,4,3',4'-Tetrahydroxy-2-methoxychalcone	288.32	3.19	4	5	1.33	0.64	0.01	0.2	0	
MOL004969	2-Ethyl-p-xylene	134.24	3.75	0	0	20.6	1.89	2.03	0.02	0	
MOL000497	licochalcone a	338.43	4.62	2	4	40.79	0.82	-0.21	0.29	0	16.2
MOL004970	3-methylheptane	114.26	3.82	0	0	36.61	1.79	2.16	0.01	0	10.56
MOL004971	3-methylhexane	100.23	3.36	0	0	38.19	1.78	2.15	0.01	0	10.9
MOL004972	3-Methylpentane	86.2	2.9	0	0	35.77	1.76	2.14	0	0	11.1
MOL004973	3-Ethylpentane	100.23	3.36	0	0	35.74	1.79	2.16	0.01	0	11.5
MOL004974	3'-Methoxyglabridin	354.43	3.93	2	5	46.16	0.94	0.47	0.57	0	0.52
MOL004975	3尾-formylglabrolide	496.75	5.33	0	5	16.36	0.26	-0.5	0.55	0	
MOL004976	Daidzein dimethyl ether	282.31	2.83	0	4	24.29	0.98	0.17	0.24	0	
MOL004977	1-Methoxyficifolinol	422.56	6.1	2	5	14.61	1.09	0.2	0.86	0	
MOL004978	2-[(3R)-8,8-dimethyl-3,4-dihydro-2H-pyrano[6,5-f]chromen-3-yl]-5-methoxyphenol	338.43	4.2	1	4	36.21	1.12	0.61	0.52	0	-0.13
MOL004979	4,2',4',alpha-Tetrahydroxydihydrochalcone	274.29	2.24	4	5	2.45	0.1	-0.4	0.16	0	
MOL004980	Inflacoumarin A	322.38	4.7	2	4	39.71	0.73	-0.24	0.33	0	2.31
MOL004981	1-(5-hydroxy-2,2-dimethylchromen-6-yl)-3-(4-hydroxyphenyl)prop-2-en-1-one	322.38	3.96	2	4	5.2	0.86	-0.01	0.34	0	
MOL004982	2,6,10-trimethyl-dodecane	144.14	-1.02	2	4	37.8	0.08	0.04	0.03	0	11.49
MOL004983	5,6,7,8-Tetrahydro-4-methylquinoline	147.24	2.69	0	1	59.18	1.63	1.79	0.04	0	11.39
MOL005015	Licoriisoflavan A	438.61	6.84	2	5	3.68	1.1	0.28	0.66	0	
MOL004985	icos-5-enoic acid	310.58	7.75	1	2	30.7	1.22	1.09	0.2	0	5.28
MOL004986	6″-O-acetylliquiritin	444.47	2.33	3	9	6.26	-0.48	-1.24	0.82	0	
MOL004987	11-deoxyglycyrrhetic acid	456.78	6.42	2	3	16.21	0.51	-0.22	0.76	0	
MOL004988	Kanzonol F	420.54	5.3	1	5	32.47	1.18	0.56	0.89	0.28	9.98
MOL004989	6-prenylated eriodictyol	356.4	3.89	4	6	39.22	0.4	-0.29	0.41	0	16.52
MOL004990	7,2',4'-trihydroxy－5-methoxy-3－arylcoumarin	300.28	2.56	3	6	83.71	0.24	-0.59	0.27	0	0.99
MOL004991	7-Acetoxy-2-methylisoflavone	294.32	3.15	0	4	38.92	0.74	0.16	0.26	0	17.49
MOL004992	7-hydroxy-2-methyl-3-phenyl-chromone	252.28	3.11	1	3	25.8	1	0.4	0.18	0	

MOL004993	8-prenylated eriodictyol	356.4	3.89	4	6	53.79	0.43	-0.44	0.4	0	15.7
MOL004994	12-methyltetradecanoate	256.48	5.96	0	2	17.36	1.35	1.15	0.09	0	
MOL004995	Kanzonol H	424.58	6.1	2	5	16.92	0.96	0.38	0.8	0.27	
MOL004996	gadelaidic acid	310.58	7.75	1	2	30.7	1.2	0.94	0.2	0	5.25
MOL004997	Araboglycyrrhizin	779.03	2.72	7	14	17.73	-2.46	-2.73	0.14	0	
MOL004998	Araboglycyrrhizin_qt	470.76	5.49	2	4	17.71	0.11	-0.52	0.74	0	
MOL004999	Artonin E	436.49	4.67	4	7	11.38	0.34	-0.33	0.8	0	
MOL000500	Vestitol	272.32	3.15	2	4	74.66	0.86	0.3	0.21	0	3
MOL005000	Gancaonin G	352.41	4.17	2	5	60.44	0.78	0.23	0.39	0	16.13
MOL005001	Gancaonin H	420.49	4.71	3	6	50.1	0.6	-0.14	0.78	0	16.64
MOL005002	beta-Glycyrrhetinic acid	470.76	5.49	2	4	17.41	0.19	-0.4	0.74	0	
MOL005003	Licoagrocarpin	338.43	4.51	1	4	58.81	1.23	0.61	0.58	0.27	9.45
MOL005004	Gancaonin I	354.43	5.23	2	5	21.9	0.93	0.37	0.39	0	
MOL005005	Glyasperin A	422.51	5.48	4	6	2.46	0.6	-0.26	0.63	0	
MOL005006	Glyasperins K	368.46	4.54	1	5	10.15	0.74	0.22	0.44	0	
MOL005007	Glyasperins M	368.41	3.22	2	6	72.67	0.49	-0.04	0.59	0	15.57
MOL005008	Glycyrrhiza flavonol A	370.38	2.17	4	7	41.28	-0.09	-0.81	0.6	0	13.71
MOL005009	Corylifolinin	324.4	4.76	3	4	1.04	0.81	-0.03	0.27	0	
MOL005010	Kanzonol E	388.49	5.51	1	4	5.77	0.98	0.06	0.71	0	
MOL005011	Kanzonol Z	406.51	4.93	2	5	21.77	0.5	-0.18	0.76	0	
MOL005012	Licoagroisoflavone	336.36	3.48	2	5	57.28	0.71	0.09	0.49	0	19.64
MOL005013	18伪-hydroxyglycyrrhetic acid	486.76	4.55	3	5	41.16	-0.29	-0.78	0.71	0	4.96
MOL005014	Licorice glycoside A	726.74	1.81	8	16	5.95	-2.37	-3.26	0.35	0.32	
MOL005016	Odoratin	314.31	2.3	2	6	49.95	0.42	-0.24	0.3	0	16.35
MOL005017	Phaseol	336.36	4.87	2	5	78.77	0.76	-0.06	0.58	0	9.64
MOL005018	Xambioona	388.49	4.68	0	4	54.85	1.09	0.52	0.87	0	14.5
MOL005019	(2R)-7-hydroxy-2-[4-hydroxy-3-(3-methylbut-2-enyl)phenyl]chroman-4-one	324.4	4.42	2	4	5.99	0.74	-0.06	0.33	0	
MOL005020	dehydroglyasperins C	340.4	4.3	4	5	53.82	0.68	-0.12	0.37	0	2.75
MOL005021	Mipax	194.2	1.54	0	4	57.4	0.64	0.63	0.06	0	5.56
MOL000511	ursolic acid	456.78	6.47	2	3	16.77	0.67	0.07	0.75	0.26	
MOL000561	Astragalin	448.41	-0.32	7	11	14.03	-1.34	-1.97	0.74	0.34	
MOL000057	DIBP	278.38	3.92	0	4	49.63	0.85	0.68	0.13	0	3.94
MOL000668	PENTYLFURAN	138.23	3.12	0	1	54.59	1.72	1.94	0.02	0.1	-1.96
MOL000671	()-Menthol	156.3	2.78	1	1	59.33	1.27	1.42	0.03	0.22	11.03
MOL000676	DBP	278.38	4.2	0	4	64.54	0.8	0.56	0.13	0.34	5.41
MOL000703	2-heptanone	114.21	1.79	0	1	46.56	1.31	1.57	0.01	0.25	10.58
MOL000705	WLN: VH6	114.21	2.31	0	1	19.59	1.29	1.49	0.01	0.23	
MOL000098	quercetin	302.25	1.5	5	7	46.43	0.05	-0.77	0.28	0.38	14.4

Detailed Information of 147 Compounds				
Mol ID	Molecule Name	OB (%)	DL	Source
MOL002211	11,14-eicosadienoic acid	39.99	0.2	Fu Zi
MOL002388	Delphin_qt	57.76	0.28	
MOL002392	Deltoin	46.69	0.37	
MOL002393	Demethyldelavaine A	34.52	0.18	
MOL002394	Demethyldelavaine B	34.52	0.18	
MOL002395	Deoxyandrographolide	56.3	0.31	
MOL002397	karakoline	51.73	0.73	
MOL002398	Karanjin	69.56	0.34	
MOL002401	Neokadsuranic acid B	43.1	0.85	
MOL002406	2,7-Dideacetyl-2,7-dibenzoyl-taxayunnanine F	39.43	0.38	
MOL002410	benzoylnapelline	34.06	0.53	
MOL002415	6-Demethyldesoline	51.87	0.66	
MOL002416	deoxyaconitine	30.96	0.24	
MOL002419	(R)-Norcoclaurine	82.54	0.21	
MOL002421	ignavine	84.08	0.25	
MOL002422	isotalatizidine	50.82	0.73	
MOL002423	jesaconitine	33.41	0.19	
MOL002433	(3R,8S,9R,10R,13R,14S,17R)-3-hydroxy-4,4,9,13,14-pentamethyl-17-[(E,2R)-6-methyl-7-[(2R,3R,4S,5S,6R)-3,4,5-trihydroxy-6-[[(2R,3R,4S,5S,6R)-3,4,5-trihydroxy-6-(hydroxymethyl)oxan-2-yl]oxymethyl]oxan-2-yl]oxyhept-5-en-2-yl]-1,2,3,7,8,10,12,15,16,17-decahydr	41.52	0.22	
MOL002434	Carnosifloside I_qt	38.16	0.8	
MOL000359	sitosterol	36.91	0.75	
MOL000538	hypaconitine	31.39	0.26	
MOL002464	1-Monolinolein	37.18	0.3	

MOL002501	[(1S)-3-[(E)-but-2-enyl]-2-methyl-4-oxo-1-cyclopent-2-enyl] (1R,3R)-3-[(E)-3-methoxy-2-methyl-3-oxoprop-1-enyl]-2,2-dimethylcyclopropane-1-carboxylate	62.52	0.31	Gan Jiang
MOL002514	Sexangularetin	62.86	0.3	
MOL000358	beta-sitosterol	36.91	0.75	
MOL000359	sitosterol	36.91	0.75	
MOL000020	12-senecioyl-2E,8E,10E-atractylentriol	62.4	0.22	BaiZhu
MOL000021	14-acetyl-12-senecioyl-2E,8E,10E-atractylentriol	60.31	0.31	
MOL000022	14-acetyl-12-senecioyl-2E,8Z,10E-atractylentriol	63.37	0.3	
MOL000028	α-Amyrin	39.51	0.76	
MOL000033	(3S,8S,9S,10R,13R,14S,17R)-10,13-dimethyl-17-[(2R,5S)-5-propan-2-yloctan-2-yl]-2,3,4,7,8,9,11,12,14,15,16,17-dodecahydro-1H-cyclopenta[a]phenanthren-3-ol	36.23	0.78	
MOL000049	3β-acetoxyatractylone	54.07	0.22	
MOL000072	8β-ethoxy atractylenolide III	35.95	0.21	
MOL002879	Diop	43.59	0.39	Ren Shen
MOL000449	Stigmasterol	43.83	0.76	
MOL000358	beta-sitosterol	36.91	0.75	
MOL003648	Inermin	65.83	0.54	
MOL000422	kaempferol	41.88	0.24	
MOL004492	Chrysanthemaxanthin	38.72	0.58	
MOL005308	Aposiopolamine	66.65	0.22	
MOL005314	Celabenzine	101.88	0.49	
MOL005317	Deoxyharringtonine	39.27	0.81	
MOL005318	Dianthramine	40.45	0.2	
MOL005320	arachidonate	45.57	0.2	
MOL005321	Frutinone A	65.9	0.34	

MOL005344	ginsenoside rh2	36.32	0.56
MOL005348	Ginsenoside-Rh4_qt	31.11	0.78
MOL005356	Girinimbin	61.22	0.31
MOL005357	Gomisin B	31.99	0.83
MOL005360	malkangunin	57.71	0.63
MOL005376	Panaxadiol	33.09	0.79
MOL005384	suchilactone	57.52	0.56
MOL005399	alexandrin_qt	36.91	0.75
MOL005401	ginsenoside Rg5_qt	39.56	0.79
MOL000787	Fumarine	59.26	0.83
MOL001484	Inermine	75.18	0.54
MOL001792	DFV	32.76	0.18
MOL000211	Mairin	55.38	0.78
MOL002311	Glycyrol	90.78	0.67
MOL000239	Jaranol	50.83	0.29
MOL002565	Medicarpin	49.22	0.34
MOL000354	isorhamnetin	49.6	0.31
MOL000359	sitosterol	36.91	0.75
MOL003656	Lupiwighteone	51.64	0.37
MOL003896	7-Methoxy-2-methyl isoflavone	42.56	0.2
MOL000392	formononetin	69.67	0.21
MOL000417	Calycosin	47.75	0.24
MOL000422	kaempferol	41.88	0.24
MOL004328	naringenin	59.29	0.21
MOL004805	(2S)-2-[4-hydroxy-3-(3-methylbut-2-enyl)phenyl]-8,8-dimethyl-2,3-dihydropyrano[2,3-f]chromen-4-one	31.79	0.72
MOL004806	euchrenone	30.29	0.57
MOL004808	glyasperin B	65.22	0.44
MOL004810	glyasperin F	75.84	0.54
MOL004811	Glyasperin C	45.56	0.4
MOL004814	Isotrifoliol	31.94	0.42
MOL004815	(E)-1-(2,4-dihydroxyphenyl)-3-(2,2-dimethylchromen-6-yl)prop-2-en-1-one	39.62	0.35
MOL004820	kanzonols W	50.48	0.52

MOL004824	(2S)-6-(2,4-dihydroxyphenyl)-2-(2-hydroxypropan-2-yl)-4-methoxy-2,3-dihydrofuro[3,2-g]chromen-7-one	60.25	0.63
MOL004827	Semilicoisoflavone B	48.78	0.55
MOL004828	Glepidotin A	44.72	0.35
MOL004829	Glepidotin B	64.46	0.34
MOL004833	Phaseolinisoflavan	32.01	0.45
MOL004835	Glypallichalcone	61.6	0.19
MOL004838	8-(6-hydroxy-2-benzofuranyl)-2,2-dimethyl-5-chromenol	58.44	0.38
MOL004841	Licochalcone B	76.76	0.19
MOL004848	licochalcone G	49.25	0.32
MOL004849	3-(2,4-dihydroxyphenyl)-8-(1,1-dimethylprop-2-enyl)-7-hydroxy-5-methoxy-coumarin	59.62	0.43
MOL004855	Licoricone	63.58	0.47
MOL004856	Gancaonin A	51.08	0.4
MOL004857	Gancaonin B	48.79	0.45
MOL004860	licorice glycoside E	32.89	0.27
MOL004863	3-(3,4-dihydroxyphenyl)-5,7-dihydroxy-8-(3-methylbut-2-enyl)chromone	66.37	0.41
MOL004864	5,7-dihydroxy-3-(4-methoxyphenyl)-8-(3-methylbut-2-enyl)chromone	30.49	0.41
MOL004866	2-(3,4-dihydroxyphenyl)-5,7-dihydroxy-6-(3-methylbut-2-enyl)chromone	44.15	0.41
MOL004879	Glycyrin	52.61	0.47
MOL004882	Licocoumarone	33.21	0.36
MOL004883	Licoisoflavone	41.61	0.42
MOL004884	Licoisoflavone B	38.93	0.55
MOL004885	licoisoflavanone	52.47	0.54
MOL004891	shinpterocarpin	80.3	0.73

MOL004898	(E)-3-[3,4-dihydroxy-5-(3-methylbut-2-enyl)phenyl]-1-(2,4-dihydroxyphenyl)prop-2-en-	46.27	0.31	Gan Cao
MOL004903	liquiritin	65.69	0.74	
MOL004904	licopyranocoumarin	80.36	0.65	
MOL004905	3,22-Dihydroxy-11-oxo-delta(12)-oleanene-27-alpha-methoxycarbonyl-29-oic acid	34.32	0.55	
MOL004907	Glyzaglabrin	61.07	0.35	
MOL004908	Glabridin	53.25	0.47	
MOL004910	Glabranin	52.9	0.31	
MOL004911	Glabrene	46.27	0.44	
MOL004912	Glabrone	52.51	0.5	
MOL004913	1,3-dihydroxy-9-methoxy-6-benzofurano[3,2-c]chromenone	48.14	0.43	
MOL004914	1,3-dihydroxy-8,9-dimethoxy-6-benzofurano[3,2-c]chromenone	62.9	0.53	
MOL004915	Eurycarpin A	43.28	0.37	
MOL004917	glycyroside	37.25	0.79	
MOL004924	(-)-Medicocarpin	40.99	0.95	
MOL004935	Sigmoidin-B	34.88	0.41	
MOL004941	(2R)-7-hydroxy-2-(4-hydroxyphenyl)chroman-4-one	71.12	0.18	
MOL004945	(2S)-7-hydroxy-2-(4-hydroxyphenyl)-8-(3-methylbut-2-enyl)chroman-4-one	36.57	0.32	
MOL004948	Isoglycyrol	44.7	0.84	
MOL004949	Isolicoflavonol	45.17	0.42	
MOL004957	HMO	38.37	0.21	
MOL004959	1-Methoxyphaseollidin	69.98	0.64	
MOL004961	Quercetin der.	46.45	0.33	
MOL004966	3'-Hydroxy-4'-O-Methylglabridin	43.71	0.57	
MOL000497	licochalcone a	40.79	0.29	
MOL004974	3'-Methoxyglabridin	46.16	0.57	

MOL004978	2-[(3R)-8,8-dimethyl-3,4-dihydro-2H-pyrano[6,5-f]chromen-3-yl]-5-methoxyphenol	36.21	0.52
MOL004980	Inflacoumarin A	39.71	0.33
MOL004985	icos-5-enoic acid	30.7	0.2
MOL004988	Kanzonol F	32.47	0.89
MOL004989	6-prenylated eriodictyol	39.22	0.41
MOL004990	7,2',4'-trihydroxy－5-methoxy-3－arylcoumarin	83.71	0.27
MOL004991	7-Acetoxy-2-methylisoflavone	38.92	0.26
MOL004993	8-prenylated eriodictyol	53.79	0.4
MOL004996	gadelaidic acid	30.7	0.2
MOL000500	Vestitol	74.66	0.21
MOL005000	Gancaonin G	60.44	0.39
MOL005001	Gancaonin H	50.1	0.78
MOL005003	Licoagrocarpin	58.81	0.58
MOL005007	Glyasperins M	72.67	0.59
MOL005008	Glycyrrhiza flavonol A	41.28	0.6
MOL005012	Licoagroisoflavone	57.28	0.49
MOL005013	18α-hydroxyglycyrrhetic acid	41.16	0.71
MOL005016	Odoratin	49.95	0.3
MOL005017	Phaseol	78.77	0.58
MOL005018	Xambioona	54.85	0.87
MOL005020	dehydroglyasperins C	53.82	0.37
MOL000098	quercetin	46.43	0.28

Specification:The duplicate value is marked in light red

Information of 147 Targets						
MOLID	ID	（pinyin_n	ıarma Mod	Norm Fit	Uniplot	GENE
MOL000359	A1	FU ZI, GAN JIANG, GA N CAO	1j96_v	0.9987	AK1C2_HUMAN	AKR1C2
MOL000359	A1	FU ZI, GAN JIANG, GAN CAO	1e7e_v	0.9838	ALBU_HUMAN	ALB

MOL000359	A1	FU ZI, GAN JIANG, GA N CAO	1oj9_v	0.9779	AOFB_HUMAN	MAOB
MOL000359	A1	FU ZI, GAN JIANG,GA N CAO	1l6l_v	0.9707	APOA2_HUMAN	APOA2
MOL000359	A1	FU ZI,GAN JIANG,GA N CAO	1reu_v	0.9914	BMP2_HUMAN	BMP2
MOL000359	A1	FU ZI,GAN JIANG,GA N CAO	1ze8_v	0.9893	CAH2_HUMAN	CA2
MOL000359	A1	FU ZI,GAN JIANG,GA N CAO	1shj_v	0.9523	CASP7_HUMAN	CASP7
MOL000359	A1	FU ZI,GAN JIANG,GA N CAO	2pg2_v	0.9431	KIF11_HUMAN	KIF11
MOL000359	A1	FU ZI,GAN JIANG,GA N CAO	1ya3_v	0.9497	MCR_HUMAN	NR3C2
MOL000359	A1	FU ZI,GAN JIANG,GA N CAO	1pme_v	0.9959	MK01_HUMAN	MAPK1
MOL000359	A1	FU ZI,GAN JIANG,GA N CAO	830c_v	0.9002	MMP13_HUMAN	MMP13
MOL000359	A1	FU ZI,GAN JIANG,GA N CAO	2pin_v	0.9845	P10828	THRB
MOL000359	A1	FU ZI,GAN JIANG,GA N CAO	3bgp_v	0.9921	P11309	PIM1
MOL000359	A1	FU ZI,GAN JIANG,GA N CAO	1qyx_v	0.9168	P14061	HSD17B1

MOL000359	A1	FU ZI,GAN JIANG,GAN CAO	2jbp_v	0.9954	P49137	MAPKAPK2
MOL000359	A1	FU ZI,GAN JIANG,GAN CAO	1a28_v	0.9705	PRGR_HUMAN	PGR
MOL000359	A1	FU ZI,GAN JIANG,GAN CAO	1n83_v	0.964	RORA_HUMAN	RORA
MOL000359	A1	FU ZI,GAN JIANG,GAN CAO	1p49_v	0.9734	STS_HUMAN	STS
MOL000358	B1	GAN JIANG,REN SHEN	1j96_v	0.9991	AK1C2_HUMAN	AKR1C2
MOL000358	B1	GANJIANG ,RENSHEN	1e7e_v	0.9792	ALBU_HUMAN	ALB
MOL000358	B1	GANJIANG ,RENSHEN	2pir_v	0.9135	ANDR_HUMAN	AR
MOL000358	B1	GANJIANG ,RENSHEN	1oj9_v	0.9913	AOFB_HUMAN	MAOB
MOL000358	B1	GANJIANG ,RENSHEN	1l6l_v	0.9596	APOA2_HUMAN	APOA2
MOL000358	B1	GANJIANG ,RENSHEN	1reu_v	0.9917	BMP2_HUMAN	BMP2
MOL000358	B1	GANJIANG ,RENSHEN	1okl_v	0.9574	CAH2_HUMAN	CA2
MOL000358	B1	GANJIANG ,RENSHEN	1shj_v	0.9261	CASP7_HUMAN	CASP7
MOL000358	B1	GANJIANG ,RENSHEN	2fky_v	0.9563	KIF11_HUMAN	KIF11
MOL000358	B1	GANJIANG ,RENSHEN	1ya3_v	0.9585	MCR_HUMAN	NR3C2
MOL000358	B1	GANJIANG ,RENSHEN	1pme_v	0.9058	MK01_HUMAN	MAPK1
MOL000358	B1	GANJIANG ,RENSHEN	1o6u_v	0.9122	O76054	SEC14L2
MOL000358	B1	GANJIANG ,RENSHEN	2pin_v	0.9799	P10828	THRB
MOL000358	B1	GANJIANG ,RENSHEN	3bgp_v	0.9822	P11309	PIM1
MOL000358	B1	GANJIANG ,RENSHEN	1qyx_v	0.9318	P14061	HSD17B1
MOL000358	B1	GANJIANG ,RENSHEN	2jbp_v	0.9944	P49137	MAPKAPK2
MOL000358	B1	GANJIANG ,RENSHEN	1a28_v	0.9727	PRGR_HUMAN	PGR

MOL000358	B1	GANJIANG ,RENSHEN	1j99_v	0.9077	Q06520	SULT2A1
MOL000358	B1	GANJIANG ,RENSHEN	1n83_v	0.9803	RORA_HUMAN	RORA
MOL000358	B1	GANJIANG ,RENSHEN	1p49_v	0.974	STS_HUMAN	STS
MOL000358	B1	GANJIANG ,RENSHEN	1j78_v	0.9182	VTDB_HUMAN	GC
MOL000422	C1	REN SHEN,GAN CAO	1xlu_v	0.9711	CHLE_HUMAN	BCHE
MOL000422	C1	REN SHEN,GAN CAO	2itx_v	0.9376	EGFR_HUMAN	EGFR
MOL000422	C1	REN SHEN,GAN CAO	2pog_v	0.9774	ESR1_HUMAN	ESR1
MOL000422	C1	REN SHEN,GAN CAO	1ogs_v	0.922	GLCM_HUMAN	GBA
MOL000422	C1	REN SHEN,GAN CAO	2hck_v	0.9938	HCK_HUMAN	HCK
MOL000422	C1	REN SHEN,GAN CAO	1yxx_v	0.9592	PIM1_HUMAN	PIM1
MOL000020	BZ1	BAI ZHU	1xf0_v	0.9865	AK1C3_HUMAN	AKR1C3
MOL000020	BZ1	BAI ZHU	2pir_v	0.9814	ANDR_HUMAN	AR
MOL000020	BZ1	BAI ZHU	1hak_v	0.9128	ANXA5_HUMAN	ANXA5
MOL000020	BZ1	BAI ZHU	3bmp_v	0.9903	BMP2_HUMAN	BMP2
MOL000020	BZ1	BAI ZHU	2qo8_v	0.9904	CAH2_HUMAN	CA2
MOL000020	BZ1	BAI ZHU	1gfw_v	0.9559	CASP3_HUMAN	CASP3
MOL000020	BZ1	BAI ZHU	1rs0_v	0.9655	CFAB_HUMAN	CFB
MOL000020	BZ1	BAI ZHU	1p0i_v	0.9791	CHLE_HUMAN	BCHE
MOL000020	BZ1	BAI ZHU	1vec_v	0.9489	DDX6_HUMAN	DDX6
MOL000020	BZ1	BAI ZHU	20gs_v	0.9823	GSTP1_HUMAN	GSTP1
MOL000020	BZ1	BAI ZHU	2fky_v	0.9543	KIF11_HUMAN	KIF11
MOL000020	BZ1	BAI ZHU	1pme_v	0.9527	MK01_HUMAN	MAPK1
MOL000020	BZ1	BAI ZHU	1uki_v	0.9862	MK08_HUMAN	MAPK8
MOL000020	BZ1	BAI ZHU	1pmv_v	0.9949	MK10_HUMAN	MAPK10
MOL000020	BZ1	BAI ZHU	830c_v	0.9749	MMP13_HUMAN	MMP13
MOL000020	BZ1	BAI ZHU	1bm6_v	0.9927	MMP3_HUMAN	MMP3
MOL000020	BZ1	BAI ZHU	1pq9_v	0.9507	NR1H2_HUMAN	NR1H2
MOL000020	BZ1	BAI ZHU	3eqm_v	0.953	P11511	CYP19A1
MOL000020	BZ1	BAI ZHU	2jbp_v	0.9929	P49137	MAPKAPK2
MOL000020	BZ1	BAI ZHU	1w8l_v	0.9903	P62937	PPIA
MOL000020	BZ1	BAI ZHU	2c3i_v	0.9762	PIM1_HUMAN	PIM1

MOL000020	BZ1	BAI ZHU	2f6t_v	0.9922	PTN1_HUMAN	PTPN1
MOL000020	BZ1	BAI ZHU	1zyj_v	0.9167	Q16539	MAPK14
MOL000020	BZ1	BAI ZHU	1q8m_v	0.9868	Q9NP99	TREM1
MOL000020	BZ1	BAI ZHU	1no9_v	0.9032	THRB_HUMAN	F2
MOL000020	BZ1	BAI ZHU	2roy_v	0.9892	TTHY_HUMAN	TTR
MOL000021	BZ2	BAI ZHU	1e7e_v	0.9987	ALBU_HUMAN	ALB
MOL000021	BZ2	BAI ZHU	2ama_v	0.9904	ANDR_HUMAN	AR
MOL000021	BZ2	BAI ZHU	1hak_v	0.9276	ANXA5_HUMAN	ANXA5
MOL000021	BZ2	BAI ZHU	1oj9_v	0.9473	AOFB_HUMAN	MAOB
MOL000021	BZ2	BAI ZHU	1l6l_v	0.9981	APOA2_HUMAN	APOA2
MOL000021	BZ2	BAI ZHU	3bmp_v	0.9557	BMP2_HUMAN	BMP2
MOL000021	BZ2	BAI ZHU	1oq5_v	0.9929	CAH2_HUMAN	CA2
MOL000021	BZ2	BAI ZHU	3dej_v	0.9824	CASP3_HUMAN	CASP3
MOL000021	BZ2	BAI ZHU	1shj_v	0.9524	CASP7_HUMAN	CASP7
MOL000021	BZ2	BAI ZHU	1lyw_v	0.9606	CATD_HUMAN	CTSD
MOL000021	BZ2	BAI ZHU	2uzd_v	0.9491	CCNA2_HUMAN	CCNA2
MOL000021	BZ2	BAI ZHU	1unh_v	0.9424	CD5R1_HUMAN	CDK5R1
MOL000021	BZ2	BAI ZHU	1rs0_v	0.99	CFAB_HUMAN	CFB
MOL000021	BZ2	BAI ZHU	2brb_v	0.918	CHK1_HUMAN	CHEK1
MOL000021	BZ2	BAI ZHU	1p0i_v	0.9918	CHLE_HUMAN	BCHE
MOL000021	BZ2	BAI ZHU	1vec_v	0.9688	DDX6_HUMAN	DDX6
MOL000021	BZ2	BAI ZHU	2ocf_v	0.9841	ESR1_HUMAN	ESR1
MOL000021	BZ2	BAI ZHU	1ya8_v	0.9865	EST1_HUMAN	CES1
MOL000021	BZ2	BAI ZHU	1f40_v	0.9982	FKB1A_HUMAN	FKBP1A
MOL000021	BZ2	BAI ZHU	1lbk_v	0.988	GSTP1_HUMAN	GSTP1
MOL000021	BZ2	BAI ZHU	2pg2_v	0.9966	KIF11_HUMAN	KIF11
MOL000021	BZ2	BAI ZHU	1pme_v	0.9302	MK01_HUMAN	MAPK1
MOL000021	BZ2	BAI ZHU	1uki_v	0.9995	MK08_HUMAN	MAPK8
MOL000021	BZ2	BAI ZHU	1pmv_v	0.9973	MK10_HUMAN	MAPK10
MOL000021	BZ2	BAI ZHU	1bm6_v	0.9917	MMP3_HUMAN	MMP3
MOL000021	BZ2	BAI ZHU	1qqs_v	0.9954	NGAL_HUMAN	LCN2
MOL000021	BZ2	BAI ZHU	1pq9_v	0.9314	NR1H2_HUMAN	NR1H2
MOL000021	BZ2	BAI ZHU	2pin_v	0.9864	P10828	THRB
MOL000021	BZ2	BAI ZHU	3bgp_v	0.9785	P11309	PIM1
MOL000021	BZ2	BAI ZHU	3eqm_v	0.9021	P11511	CYP19A1
MOL000021	BZ2	BAI ZHU	2jbp_v	0.9849	P49137	IAPKAPK2
MOL000021	BZ2	BAI ZHU	1w8l_v	0.9917	P62937	PPIA
MOL000021	BZ2	BAI ZHU	2f6v_v	0.9882	PTN1_HUMAN	PTPN1
MOL000021	BZ2	BAI ZHU	1p49_v	0.9972	STS_HUMAN	STS
MOL000021	BZ2	BAI ZHU	1no9_v	0.9712	THRB_HUMAN	F2
MOL000021	BZ2	BAI ZHU	1f86_v	0.9927	TTHY_HUMAN	TTR
MOL000022	BZ3	BAI ZHU	1j96_v	0.9234	AK1C2_HUMAN	AKR1C2

MOL000022	BZ3	BAI ZHU	1xf0_v	0.9603	AK1C3_HUMAN	AKR1C3
MOL000022	BZ3	BAI ZHU	1e7e_v	0.9919	ALBU_HUMAN	ALB
MOL000022	BZ3	BAI ZHU	2agt_v	0.9237	ALDR_HUMAN	AKR1B1
MOL000022	BZ3	BAI ZHU	2pir_v	0.9892	ANDR_HUMAN	AR
MOL000022	BZ3	BAI ZHU	1hak_v	0.9402	ANXA5_HUMAN	ANXA5
MOL000022	BZ3	BAI ZHU	1oj9_v	0.9913	AOFB_HUMAN	MAOB
MOL000022	BZ3	BAI ZHU	1l6l_v	0.9764	APOA2_HUMAN	APOA2
MOL000022	BZ3	BAI ZHU	2q11_v	0.9858	BACE1_HUMAN	BACE1
MOL000022	BZ3	BAI ZHU	3bmp_v	0.9824	BMP2_HUMAN	BMP2
MOL000022	BZ3	BAI ZHU	1if4_v	0.9932	CAH2_HUMAN	CA2
MOL000022	BZ3	BAI ZHU	3dej_v	0.959	CASP3_HUMAN	CASP3
MOL000022	BZ3	BAI ZHU	1shj_v	0.9857	CASP7_HUMAN	CASP7
MOL000022	BZ3	BAI ZHU	1lyw_v	0.9538	CATD_HUMAN	CTSD
MOL000022	BZ3	BAI ZHU	2uzd_v	0.9117	CCNA2_HUMAN	CCNA2
MOL000022	BZ3	BAI ZHU	1unh_v	0.9188	CD5R1_HUMAN	CDK5R1
MOL000022	BZ3	BAI ZHU	1rs0_v	0.9832	CFAB_HUMAN	CFB
MOL000022	BZ3	BAI ZHU	1dic_v	0.9593	CFAD_HUMAN	CFD
MOL000022	BZ3	BAI ZHU	2brg_v	0.9736	CHK1_HUMAN	CHEK1
MOL000022	BZ3	BAI ZHU	1p0i_v	0.9764	CHLE_HUMAN	BCHE
MOL000022	BZ3	BAI ZHU	1vec_v	0.9684	DDX6_HUMAN	DDX6
MOL000022	BZ3	BAI ZHU	2ity_v	0.9295	EGFR_HUMAN	EGFR
MOL000022	BZ3	BAI ZHU	2vwu_v	0.9688	EPHB4_HUMAN	EPHB4
MOL000022	BZ3	BAI ZHU	2ocf_v	0.9712	ESR1_HUMAN	ESR1
MOL000022	BZ3	BAI ZHU	1qkm_v	0.9412	ESR2_HUMAN	ESR2
MOL000022	BZ3	BAI ZHU	1ya8_v	0.986	EST1_HUMAN	CES1
MOL000022	BZ3	BAI ZHU	1f40_v	0.9933	FKB1A_HUMAN	FKBP1A
MOL000022	BZ3	BAI ZHU	1ogs_v	0.9184	GLCM_HUMAN	GBA
MOL000022	BZ3	BAI ZHU	1md3_v	0.9876	GSTP1_HUMAN	GSTP1
MOL000022	BZ3	BAI ZHU	2pg2_v	0.9859	KIF11_HUMAN	KIF11
MOL000022	BZ3	BAI ZHU	1pme_v	0.9908	MK01_HUMAN	MAPK1
MOL000022	BZ3	BAI ZHU	1uki_v	0.9992	MK08_HUMAN	MAPK8
MOL000022	BZ3	BAI ZHU	1pmv_v	0.9984	MK10_HUMAN	MAPK10
MOL000022	BZ3	BAI ZHU	1eub_v	0.9905	MMP13_HUMAN	MMP13
MOL000022	BZ3	BAI ZHU	1bm6_v	0.999	MMP3_HUMAN	MMP3
MOL000022	BZ3	BAI ZHU	2pin_v	0.9832	P10828	THRB
MOL000022	BZ3	BAI ZHU	3eqm_v	0.9649	P11511	CYP19A1
MOL000022	BZ3	BAI ZHU	3cjf_v	0.9974	P35968	KDR
MOL000022	BZ3	BAI ZHU	2jbp_v	0.9967	P49137	MAPKAPK2
MOL000022	BZ3	BAI ZHU	1w8l_v	0.9806	P62937	PPIA
MOL000022	BZ3	BAI ZHU	2c3i_v	0.9821	PIM1_HUMAN	PIM1
MOL000022	BZ3	BAI ZHU	1v3q_v	0.9833	PNPH_HUMAN	PNP
MOL000022	BZ3	BAI ZHU	1zgy_v	0.9765	PPARG_HUMAN	PPARG

MOL000022	BZ3	BAI ZHU	2ovm_v	0.9359	PRGR_HUMAN	PGR
MOL000022	BZ3	BAI ZHU	1q8m_v	0.9905	Q9NP99	TREM1
MOL000022	BZ3	BAI ZHU	1n83_v	0.9082	RORA_HUMAN	RORA
MOL000022	BZ3	BAI ZHU	1p49_v	0.9974	STS_HUMAN	STS
MOL000022	BZ3	BAI ZHU	1vjy_v	0.9063	TGFR1_HUMAN	TGFBR1
MOL000022	BZ3	BAI ZHU	1no9_v	0.9943	THRB_HUMAN	F2
MOL000022	BZ3	BAI ZHU	1f86_v	0.9971	TTHY_HUMAN	TTR
MOL000022	BZ3	BAI ZHU	1j78_v	0.9779	VTDB_HUMAN	GC
MOL000028	BZ4	BAI ZHU	1j96_v	0.9957	AK1C2_HUMAN	AKR1C2
MOL000028	BZ4	BAI ZHU	1e7e_v	0.9837	ALBU_HUMAN	ALB
MOL000028	BZ4	BAI ZHU	1l6l_v	0.9556	APOA2_HUMAN	APOA2
MOL000028	BZ4	BAI ZHU	1reu_v	0.9718	BMP2_HUMAN	BMP2
MOL000028	BZ4	BAI ZHU	1okl_v	0.9484	CAH2_HUMAN	CA2
MOL000028	BZ4	BAI ZHU	1shj_v	0.9693	CASP7_HUMAN	CASP7
MOL000028	BZ4	BAI ZHU	1xdd_v	0.9998	ITAL_HUMAN	ITGAL
MOL000028	BZ4	BAI ZHU	2pg2_v	0.963	KIF11_HUMAN	KIF11
MOL000028	BZ4	BAI ZHU	1eub_v	0.9832	MMP13_HUMAN	MMP13
MOL000028	BZ4	BAI ZHU	3bgp_v	0.9448	P11309	PIM1
MOL000028	BZ4	BAI ZHU	2p3g_v	0.9533	P49137	MAPKAPK2
MOL000028	BZ4	BAI ZHU	1wbo_v	0.9146	Q16539	MAPK14
MOL000028	BZ4	BAI ZHU	1p49_v	0.9868	STS_HUMAN	STS
MOL000028	BZ4	BAI ZHU	2roy_v	0.9521	TTHY_HUMAN	TTR
MOL000033	BZ5	BAI ZHU	1j96_v	0.9987	AK1C2_HUMAN	AKR1C2
MOL000033	BZ5	BAI ZHU	1e7e_v	0.9729	ALBU_HUMAN	ALB
MOL000033	BZ5	BAI ZHU	1oj9_v	0.9987	AOFB_HUMAN	MAOB
MOL000033	BZ5	BAI ZHU	1l6l_v	0.9787	APOA2_HUMAN	APOA2
MOL000033	BZ5	BAI ZHU	1reu_v	0.991	BMP2_HUMAN	BMP2
MOL000033	BZ5	BAI ZHU	1ze8_v	0.9859	CAH2_HUMAN	CA2
MOL000033	BZ5	BAI ZHU	1shj_v	0.9661	CASP7_HUMAN	CASP7
MOL000033	BZ5	BAI ZHU	1qku_v	0.9256	ESR1_HUMAN	ESR1
MOL000033	BZ5	BAI ZHU	1f40_v	0.9558	FKB1A_HUMAN	FKBP1A
MOL000033	BZ5	BAI ZHU	2pg2_v	0.9503	KIF11_HUMAN	KIF11
MOL000033	BZ5	BAI ZHU	2aa6_v	0.9071	MCR_HUMAN	NR3C2
MOL000033	BZ5	BAI ZHU	1eub_v	0.9364	MMP13_HUMAN	MMP13
MOL000033	BZ5	BAI ZHU	2pin_v	0.9645	P10828	THRB
MOL000033	BZ5	BAI ZHU	3bgp_v	0.9902	P11309	PIM1
MOL000033	BZ5	BAI ZHU	2jbp_v	0.9968	P49137	MAPKAPK2
MOL000033	BZ5	BAI ZHU	1n83_v	0.9427	RORA_HUMAN	RORA
MOL000033	BZ5	BAI ZHU	1p49_v	0.9761	STS_HUMAN	STS
MOL000033	BZ5	BAI ZHU	2roy_v	0.9003	TTHY_HUMAN	TTR
MOL000033	BZ5	BAI ZHU	1j78_v	0.9642	VTDB_HUMAN	GC
MOL000049	BZ6	BAI ZHU	1j96_v	0.9316	AK1C2_HUMAN	AKR1C2

MOL000049	BZ6	BAI ZHU	1if4_v	0.9635	CAH2_HUMAN	CA2
MOL000049	BZ6	BAI ZHU	1xdd_v	0.9891	ITAL_HUMAN	ITGAL
MOL000049	BZ6	BAI ZHU	1w8l_v	0.9762	P62937	PPIA
MOL000072	BZ7	BAI ZHU	1j96_v	0.9022	AK1C2_HUMAN	AKR1C2
MOL000072	BZ7	BAI ZHU	1okl_v	0.9128	CAH2_HUMAN	CA2
MOL000072	BZ7	BAI ZHU	1md3_v	0.9744	GSTP1_HUMAN	GSTP1
MOL000072	BZ7	BAI ZHU	1pme_v	0.9177	MK01_HUMAN	MAPK1
MOL000072	BZ7	BAI ZHU	1uki_v	0.911	MK08_HUMAN	MAPK8
MOL000072	BZ7	BAI ZHU	1pmv_v	0.9371	MK10_HUMAN	MAPK10
MOL000072	BZ7	BAI ZHU	2p3g_v	0.9396	P49137	MAPKAPK2
MOL000072	BZ7	BAI ZHU	2a4z_v	0.9136	PK3CG_HUMAN	PIK3CG
MOL002211	FZ1	FU ZI	3b92_v	0.9105	ADA17_HUMAN	ADAM17
MOL002211	FZ1	FU ZI	1j96_v	0.9384	AK1C2_HUMAN	AKR1C2
MOL002211	FZ1	FU ZI	1e7e_v	0.9723	ALBU_HUMAN	ALB
MOL002211	FZ1	FU ZI	1x97_v	0.9554	ALDR_HUMAN	AKR1B1
MOL002211	FZ1	FU ZI	2pir_v	0.987	ANDR_HUMAN	AR
MOL002211	FZ1	FU ZI	1oj9_v	0.9978	AOFB_HUMAN	MAOB
MOL002211	FZ1	FU ZI	1l6l_v	0.9652	APOA2_HUMAN	APOA2
MOL002211	FZ1	FU ZI	1reu_v	0.9997	BMP2_HUMAN	BMP2
MOL002211	FZ1	FU ZI	2qo8_v	0.9883	CAH2_HUMAN	CA2
MOL002211	FZ1	FU ZI	1h1p_v	0.9869	CCNA2_HUMAN	CCNA2
MOL002211	FZ1	FU ZI	1rs0_v	0.938	CFAB_HUMAN	CFB
MOL002211	FZ1	FU ZI	2cgw_v	0.9255	CHK1_HUMAN	CHEK1
MOL002211	FZ1	FU ZI	1p0i_v	0.9431	CHLE_HUMAN	BCHE
MOL002211	FZ1	FU ZI	1tg6_v	0.9523	CLPP_HUMAN	CLPP
MOL002211	FZ1	FU ZI	1yb1_v	0.941	DHB11_HUMAN	HSD17B11
MOL002211	FZ1	FU ZI	1qku_v	0.9444	ESR1_HUMAN	ESR1
MOL002211	FZ1	FU ZI	1md3_v	0.9498	GSTP1_HUMAN	GSTP1
MOL002211	FZ1	FU ZI	2fky_v	0.9723	KIF11_HUMAN	KIF11
MOL002211	FZ1	FU ZI	1pme_v	0.9979	MK01_HUMAN	MAPK1
MOL002211	FZ1	FU ZI	3cgf_v	0.9107	MK10_HUMAN	MAPK10
MOL002211	FZ1	FU ZI	1eub_v	0.9886	MMP13_HUMAN	MMP13
MOL002211	FZ1	FU ZI	1qqs_v	0.9827	NGAL_HUMAN	LCN2
MOL002211	FZ1	FU ZI	1xv9_v	0.9828	NR1I3_HUMAN	NR1I3
MOL002211	FZ1	FU ZI	2pin_v	0.9618	P10828	THRB
MOL002211	FZ1	FU ZI	3bgp_v	0.9905	P11309	PIM1
MOL002211	FZ1	FU ZI	3eqm_v	0.9827	P11511	CYP19A1
MOL002211	FZ1	FU ZI	1e1x_v	0.9895	P24941	CDK2
MOL002211	FZ1	FU ZI	3cjf_v	0.9105	P35968	KDR
MOL002211	FZ1	FU ZI	2jbp_v	0.9974	P49137	MAPKAPK2
MOL002211	FZ1	FU ZI	1oni_v	0.9578	P52758	RIDA
MOL002211	FZ1	FU ZI	1w8l_v	0.9748	P62937	PPIA

MOL002211	FZ1	FU ZI	1zgy_v	0.9465	PPARG_HUMAN	PPARG
MOL002211	FZ1	FU ZI	1hd2_v	0.9666	PRDX5_HUMAN	PRDX5
MOL002211	FZ1	FU ZI	2ovm_v	0.9603	PRGR_HUMAN	PGR
MOL002211	FZ1	FU ZI	2f6t_v	0.9937	PTN1_HUMAN	PTPN1
MOL002211	FZ1	FU ZI	1p49_v	0.9693	STS_HUMAN	STS
MOL002211	FZ1	FU ZI	1f86_v	0.9931	TTHY_HUMAN	TTR
MOL002211	FZ1	FU ZI	1j78_v	0.9172	VTDB_HUMAN	GC
MOL002406	FZ10	FU ZI	1reu_v	0.9386	BMP2_HUMAN	BMP2
MOL002406	FZ10	FU ZI	1rs0_v	0.9551	CFAB_HUMAN	CFB
MOL002406	FZ10	FU ZI	1pmv_v	0.9807	MK10_HUMAN	MAPK10
MOL002406	FZ10	FU ZI	1eub_v	0.9462	MMP13_HUMAN	MMP13
MOL002406	FZ10	FU ZI	1bm6_v	0.9341	MMP3_HUMAN	MMP3
MOL002406	FZ10	FU ZI	1pq9_v	0.925	NR1H2_HUMAN	NR1H2
MOL002406	FZ10	FU ZI	1dvz_v	0.9172	TTHY_HUMAN	TTR
MOL002410	FZ11	FU ZI	1j96_v	1	AK1C2_HUMAN	AKR1C2
MOL002410	FZ11	FU ZI	1e7a_v	1	ALBU_HUMAN	ALB
MOL002410	FZ11	FU ZI	2agt_v	0.9069	ALDR_HUMAN	AKR1B1
MOL002410	FZ11	FU ZI	1oj9_v	1	AOFB_HUMAN	MAOB
MOL002410	FZ11	FU ZI	1l6l_v	0.9881	APOA2_HUMAN	APOA2
MOL002410	FZ11	FU ZI	2ohp_v	0.9865	BACE1_HUMAN	BACE1
MOL002410	FZ11	FU ZI	3bmp_v	1	BMP2_HUMAN	BMP2
MOL002410	FZ11	FU ZI	1if4_v	0.9976	CAH2_HUMAN	CA2
MOL002410	FZ11	FU ZI	3dej_v	0.9445	CASP3_HUMAN	CASP3
MOL002410	FZ11	FU ZI	1shj_v	0.9979	CASP7_HUMAN	CASP7
MOL002410	FZ11	FU ZI	1rs0_v	0.9878	CFAB_HUMAN	CFB
MOL002410	FZ11	FU ZI	1p0i_v	0.9778	CHLE_HUMAN	BCHE
MOL002410	FZ11	FU ZI	2vwu_v	0.9897	EPHB4_HUMAN	EPHB4
MOL002410	FZ11	FU ZI	1ya8_v	0.9368	EST1_HUMAN	CES1
MOL002410	FZ11	FU ZI	1lbk_v	0.939	GSTP1_HUMAN	GSTP1
MOL002410	FZ11	FU ZI	2fky_v	1	KIF11_HUMAN	KIF11
MOL002410	FZ11	FU ZI	2oji_v	0.9905	MK01_HUMAN	MAPK1
MOL002410	FZ11	FU ZI	1uki_v	0.9672	MK08_HUMAN	MAPK8
MOL002410	FZ11	FU ZI	1pmv_v	0.9871	MK10_HUMAN	MAPK10
MOL002410	FZ11	FU ZI	1eub_v	1	MMP13_HUMAN	MMP13
MOL002410	FZ11	FU ZI	1bm6_v	0.9923	MMP3_HUMAN	MMP3
MOL002410	FZ11	FU ZI	1pq9_v	0.9707	NR1H2_HUMAN	NR1H2
MOL002410	FZ11	FU ZI	2jbp_v	1	P49137	[illegible]APKAPK2
MOL002410	FZ11	FU ZI	1w8l_v	0.9904	P62937	PPIA
MOL002410	FZ11	FU ZI	1xoz_v	0.9907	PDE5A_HUMAN	PDE5A
MOL002410	FZ11	FU ZI	2o65_v	1	PIM1_HUMAN	PIM1
MOL002410	FZ11	FU ZI	1hnn_v	0.9861	PNMT_HUMAN	PNMT
MOL002410	FZ11	FU ZI	1p49_v	1	STS_HUMAN	STS

MOL002410	FZ11	FU ZI	1no9_v	0.9981	THRB_HUMAN	F2
MOL002410	FZ11	FU ZI	1f86_v	0.9984	TTHY_HUMAN	TTR
MOL002410	FZ11	FU ZI	1j78_v	1	VTDB_HUMAN	GC
MOL002415	FZ12	FU ZI	1u3u_v	0.9218	ADH1B_HUMAN	ADH1B
MOL002415	FZ12	FU ZI	2pit_v	0.9644	ANDR_HUMAN	AR
MOL002415	FZ12	FU ZI	1lbk_v	0.9825	GSTP1_HUMAN	GSTP1
MOL002415	FZ12	FU ZI	1pq9_v	0.9021	NR1H2_HUMAN	NR1H2
MOL002415	FZ12	FU ZI	1hpk_v	0.9729	P00747	PLG
MOL002415	FZ12	FU ZI	1qmv_v	0.9497	P32119	PRDX2
MOL002415	FZ12	FU ZI	1w8m_v	0.9926	P62937	PPIA
MOL002415	FZ12	FU ZI	1xn0_v	0.9602	PDE4B_HUMAN	PDE4B
MOL002415	FZ12	FU ZI	1q8m_v	0.9627	Q9NP99	TREM1
MOL002416	FZ13	FU ZI	2pir_v	0.9659	ANDR_HUMAN	AR
MOL002416	FZ13	FU ZI	2ohp_v	0.9644	BACE1_HUMAN	BACE1
MOL002416	FZ13	FU ZI	3bmp_v	1	BMP2_HUMAN	BMP2
MOL002416	FZ13	FU ZI	1jd0_v	0.9139	CAH12_HUMAN	CA12
MOL002416	FZ13	FU ZI	1okl_v	0.9873	CAH2_HUMAN	CA2
MOL002416	FZ13	FU ZI	1lyw_v	0.9202	CATD_HUMAN	CTSD
MOL002416	FZ13	FU ZI	1md3_v	0.9791	GSTP1_HUMAN	GSTP1
MOL002416	FZ13	FU ZI	2g01_v	0.9737	MK08_HUMAN	MAPK8
MOL002416	FZ13	FU ZI	1pmv_v	0.9825	MK10_HUMAN	MAPK10
MOL002416	FZ13	FU ZI	1bm6_v	0.9896	MMP3_HUMAN	MMP3
MOL002416	FZ13	FU ZI	1pq9_v	0.9424	NR1H2_HUMAN	NR1H2
MOL002416	FZ13	FU ZI	1le6_v	0.9348	O15496	PLA2G10
MOL002416	FZ13	FU ZI	1w8l_v	0.9973	P62937	PPIA
MOL002416	FZ13	FU ZI	1xn0_v	0.9268	PDE4B_HUMAN	PDE4B
MOL002416	FZ13	FU ZI	2pe0_v	0.9548	PDPK1_HUMAN	PDPK1
MOL002416	FZ13	FU ZI	1yi3_v	0.9825	PIM1_HUMAN	PIM1
MOL002416	FZ13	FU ZI	1hnn_v	0.9106	PNMT_HUMAN	PNMT
MOL002416	FZ13	FU ZI	1v3q_v	0.9114	PNPH_HUMAN	PNP
MOL002416	FZ13	FU ZI	1ov4_v	0.9163	Q06520	SULT2A1
MOL002416	FZ13	FU ZI	1zyj_v	0.9081	Q16539	MAPK14
MOL002416	FZ13	FU ZI	2p2h_v	0.9251	VGFR2_HUMAN	KDR
MOL002416	FZ13	FU ZI	1t84_v	0.9007	WASP_HUMAN	WAS
MOL002419	FZ14	FU ZI	2oi0_v	0.9069	ADA17_HUMAN	ADAM17
MOL002419	FZ14	FU ZI	2pir_v	0.9143	ANDR_HUMAN	AR
MOL002419	FZ14	FU ZI	2ohp_v	0.9777	BACE1_HUMAN	BACE1
MOL002419	FZ14	FU ZI	2nmx_v	0.9726	CAH1_HUMAN	CA1
MOL002419	FZ14	FU ZI	1oq5_v	0.941	CAH2_HUMAN	CA2
MOL002419	FZ14	FU ZI	1rs0_v	0.9527	CFAB_HUMAN	CFB
MOL002419	FZ14	FU ZI	1xlv_v	0.9871	CHLE_HUMAN	BCHE
MOL002419	FZ14	FU ZI	2pog_v	0.9936	ESR1_HUMAN	ESR1

MOL002419	FZ14	FU ZI	1x7j_v	0.965	ESR2_HUMAN	ESR2
MOL002419	FZ14	FU ZI	1lbk_v	0.959	GSTP1_HUMAN	GSTP1
MOL002419	FZ14	FU ZI	1uki_v	0.9508	MK08_HUMAN	MAPK8
MOL002419	FZ14	FU ZI	3cgf_v	0.993	MK10_HUMAN	MAPK10
MOL002419	FZ14	FU ZI	1pq9_v	0.9819	NR1H2_HUMAN	NR1H2
MOL002419	FZ14	FU ZI	1jvp_v	0.9483	P24941	CDK2
MOL002419	FZ14	FU ZI	3cjf_v	0.993	P35968	KDR
MOL002419	FZ14	FU ZI	1w8m_v	0.9785	P62937	PPIA
MOL002419	FZ14	FU ZI	4pah_v	0.9809	PH4H_HUMAN	PAH
MOL002419	FZ14	FU ZI	2c3i_v	0.9892	PIM1_HUMAN	PIM1
MOL002419	FZ14	FU ZI	1hnn_v	0.9571	PNMT_HUMAN	PNMT
MOL002419	FZ14	FU ZI	1v3q_v	0.9387	PNPH_HUMAN	PNP
MOL002419	FZ14	FU ZI	1no9_v	0.9838	THRB_HUMAN	F2
MOL002419	FZ14	FU ZI	1dvz_v	0.9939	TTHY_HUMAN	TTR
MOL002419	FZ14	FU ZI	1gjd_v	0.9787	UROK_HUMAN	PLAU
MOL002421	FZ15	FU ZI	2pit_v	0.9888	ANDR_HUMAN	AR
MOL002421	FZ15	FU ZI	1hak_v	0.923	ANXA5_HUMAN	ANXA5
MOL002421	FZ15	FU ZI	1oj9_v	1	AOFB_HUMAN	MAOB
MOL002421	FZ15	FU ZI	2ohq_v	0.9138	BACE1_HUMAN	BACE1
MOL002421	FZ15	FU ZI	1czm_v	0.9851	CAH1_HUMAN	CA1
MOL002421	FZ15	FU ZI	3ca2_v	0.9616	CAH2_HUMAN	CA2
MOL002421	FZ15	FU ZI	1rs0_v	0.9727	CFAB_HUMAN	CFB
MOL002421	FZ15	FU ZI	2brg_v	0.9494	CHK1_HUMAN	CHEK1
MOL002421	FZ15	FU ZI	1xlv_v	0.9775	CHLE_HUMAN	BCHE
MOL002421	FZ15	FU ZI	1klt_v	0.9229	CMA1_HUMAN	CMA1
MOL002421	FZ15	FU ZI	2onc_v	0.919	DPP4_HUMAN	DPP4
MOL002421	FZ15	FU ZI	1ya8_v	0.9497	EST1_HUMAN	CES1
MOL002421	FZ15	FU ZI	1lbk_v	0.9657	GSTP1_HUMAN	GSTP1
MOL002421	FZ15	FU ZI	1g1q_v	0.9582	LYAM3_HUMAN	SELP
MOL002421	FZ15	FU ZI	2h96_v	0.9092	MK08_HUMAN	MAPK8
MOL002421	FZ15	FU ZI	1pmv_v	0.9915	MK10_HUMAN	MAPK10
MOL002421	FZ15	FU ZI	1eub_v	1	MMP13_HUMAN	MMP13
MOL002421	FZ15	FU ZI	1bm6_v	0.964	MMP3_HUMAN	MMP3
MOL002421	FZ15	FU ZI	1pq9_v	0.9769	NR1H2_HUMAN	NR1H2
MOL002421	FZ15	FU ZI	1hpk_v	0.9654	P00747	PLG
MOL002421	FZ15	FU ZI	2pin_v	1	P10828	THRB
MOL002421	FZ15	FU ZI	2jbp_v	0.9707	P49137	MAPKAPK2
MOL002421	FZ15	FU ZI	1w8m_v	0.9823	P62937	PPIA
MOL002421	FZ15	FU ZI	1xlz_v	0.9132	PDE4B_HUMAN	PDE4B
MOL002421	FZ15	FU ZI	2pe0_v	0.9007	PDPK1_HUMAN	PDPK1
MOL002421	FZ15	FU ZI	1v3q_v	0.9764	PNPH_HUMAN	PNP
MOL002421	FZ15	FU ZI	1zyj_v	0.9164	Q16539	MAPK14

MOL002421	FZ15	FU ZI	1nub_v	0.9365	SPRC_HUMAN	SPARC
MOL002421	FZ15	FU ZI	2h8h_v	0.9422	SRC_HUMAN	SRC
MOL002421	FZ15	FU ZI	1vjy_v	0.9127	TGFR1_HUMAN	TGFBR1
MOL002421	FZ15	FU ZI	1no9_v	0.9633	THRB_HUMAN	F2
MOL002421	FZ15	FU ZI	1f86_v	1	TTHY_HUMAN	TTR
MOL002422	FZ16	FU ZI	1j96_v	1	AK1C2_HUMAN	AKR1C2
MOL002422	FZ16	FU ZI	1reu_v	1	BMP2_HUMAN	BMP2
MOL002422	FZ16	FU ZI	1okl_v	0.9899	CAH2_HUMAN	CA2
MOL002422	FZ16	FU ZI	1lyw_v	0.9511	CATD_HUMAN	CTSD
MOL002422	FZ16	FU ZI	1s3w_v	0.9209	DYR_HUMAN	DHFR
MOL002422	FZ16	FU ZI	3bgq_v	0.927	P11309	PIM1
MOL002422	FZ16	FU ZI	1w8m_v	0.9422	P62937	PPIA
MOL002422	FZ16	FU ZI	1a28_v	0.9784	PRGR_HUMAN	PGR
MOL002422	FZ16	FU ZI	1q8m_v	0.9399	Q9NP99	TREM1
MOL002422	FZ16	FU ZI	1ijn_v	0.9147	TTHY_HUMAN	TTR
MOL002423	FZ17	FU ZI	2bx8_v	0.9991	ALBU_HUMAN	ALB
MOL002423	FZ17	FU ZI	2pdw_v	0.9223	ALDR_HUMAN	AKR1B1
MOL002423	FZ17	FU ZI	1oj9_v	1	AOFB_HUMAN	MAOB
MOL002423	FZ17	FU ZI	2ohq_v	0.9872	BACE1_HUMAN	BACE1
MOL002423	FZ17	FU ZI	1czm_v	0.969	CAH1_HUMAN	CA1
MOL002423	FZ17	FU ZI	1if4_v	0.9973	CAH2_HUMAN	CA2
MOL002423	FZ17	FU ZI	2uzd_v	0.9679	CCNA2_HUMAN	CCNA2
MOL002423	FZ17	FU ZI	1rs0_v	0.9774	CFAB_HUMAN	CFB
MOL002423	FZ17	FU ZI	1dic_v	0.9219	CFAD_HUMAN	CFD
MOL002423	FZ17	FU ZI	2ywp_v	0.9588	CHK1_HUMAN	CHEK1
MOL002423	FZ17	FU ZI	1p0i_v	0.9554	CHLE_HUMAN	BCHE
MOL002423	FZ17	FU ZI	1n1m_v	0.958	DPP4_HUMAN	DPP4
MOL002423	FZ17	FU ZI	1qkt_v	0.9846	ESR1_HUMAN	ESR1
MOL002423	FZ17	FU ZI	1qkm_v	0.973	ESR2_HUMAN	ESR2
MOL002423	FZ17	FU ZI	1ogs_v	0.984	GLCM_HUMAN	GBA
MOL002423	FZ17	FU ZI	1lbk_v	0.9863	GSTP1_HUMAN	GSTP1
MOL002423	FZ17	FU ZI	2fky_v	0.9211	KIF11_HUMAN	KIF11
MOL002423	FZ17	FU ZI	1uki_v	0.9841	MK08_HUMAN	MAPK8
MOL002423	FZ17	FU ZI	1pmv_v	0.9847	MK10_HUMAN	MAPK10
MOL002423	FZ17	FU ZI	1bm6_v	0.997	MMP3_HUMAN	MMP3
MOL002423	FZ17	FU ZI	1pq9_v	0.9357	NR1H2_HUMAN	NR1H2
MOL002423	FZ17	FU ZI	1osh_v	0.9027	NR1H4_HUMAN	NR1H4
MOL002423	FZ17	FU ZI	1o6u_v	1	O76054	SEC14L2
MOL002423	FZ17	FU ZI	1xan_v	0.9145	P00390	GSR
MOL002423	FZ17	FU ZI	1hpk_v	0.9878	P00747	PLG
MOL002423	FZ17	FU ZI	3fzk_v	0.9245	P11142	HSPA8
MOL002423	FZ17	FU ZI	1qyw_v	0.9953	P14061	HSD17B1

MOL002423	FZ17	FU ZI	2rl5_v	0.9516	P35968	KDR
MOL002423	FZ17	FU ZI	2zkc_v	0.9291	P62508	ESRRG
MOL002423	FZ17	FU ZI	1w8l_v	0.9895	P62937	PPIA
MOL002423	FZ17	FU ZI	1xn0_v	0.9482	PDE4B_HUMAN	PDE4B
MOL002423	FZ17	FU ZI	1xor_v	0.9683	PDE4D_HUMAN	PDE4D
MOL002423	FZ17	FU ZI	2pe0_v	0.9073	PDPK1_HUMAN	PDPK1
MOL002423	FZ17	FU ZI	1yi3_v	0.9958	PIM1_HUMAN	PIM1
MOL002423	FZ17	FU ZI	1hnn_v	0.9487	PNMT_HUMAN	PNMT
MOL002423	FZ17	FU ZI	1v3q_v	0.9736	PNPH_HUMAN	PNP
MOL002423	FZ17	FU ZI	1fm6_v	0.9914	PPARG_HUMAN	PPARG
MOL002423	FZ17	FU ZI	1a28_v	0.9681	PRGR_HUMAN	PGR
MOL002423	FZ17	FU ZI	1ov4_v	0.9939	Q06520	SULT2A1
MOL002423	FZ17	FU ZI	1bmk_v	0.9369	Q16539	MAPK14
MOL002423	FZ17	FU ZI	1n83_v	0.9909	RORA_HUMAN	RORA
MOL002423	FZ17	FU ZI	1lhv_v	0.9089	SHBG_HUMAN	SHBG
MOL002423	FZ17	FU ZI	1dvz_v	0.991	TTHY_HUMAN	TTR
MOL002423	FZ17	FU ZI	1j78_v	1	VTDB_HUMAN	GC
MOL002423	FZ17	FU ZI	1t84_v	0.9685	WASP_HUMAN	WAS
MOL002433	FZ18	FU ZI	1j96_v	0.9197	AK1C2_HUMAN	AKR1C2
MOL002433	FZ18	FU ZI	1e7e_v	0.9348	ALBU_HUMAN	ALB
MOL002433	FZ18	FU ZI	1jdn_v	0.9497	ANPRC_HUMAN	NPR3
MOL002433	FZ18	FU ZI	1oj9_v	0.9716	AOFB_HUMAN	MAOB
MOL002433	FZ18	FU ZI	1l6l_v	0.9329	APOA2_HUMAN	APOA2
MOL002433	FZ18	FU ZI	2ohs_v	0.989	BACE1_HUMAN	BACE1
MOL002433	FZ18	FU ZI	3bmp_v	0.9533	BMP2_HUMAN	BMP2
MOL002433	FZ18	FU ZI	1okl_v	0.971	CAH2_HUMAN	CA2
MOL002433	FZ18	FU ZI	1shj_v	0.9349	CASP7_HUMAN	CASP7
MOL002433	FZ18	FU ZI	2f1g_v	0.9651	CATS_HUMAN	CTSS
MOL002433	FZ18	FU ZI	1xlw_v	0.9397	CHLE_HUMAN	BCHE
MOL002433	FZ18	FU ZI	1yb1_v	0.9671	DHB11_HUMAN	HSD17B11
MOL002433	FZ18	FU ZI	3czr_v	0.9113	DHI1_HUMAN	HSD11B1
MOL002433	FZ18	FU ZI	2itx_v	0.9447	EGFR_HUMAN	EGFR
MOL002433	FZ18	FU ZI	1qkm_v	0.9542	ESR2_HUMAN	ESR2
MOL002433	FZ18	FU ZI	1d7i_v	0.9245	FKB1A_HUMAN	FKBP1A
MOL002433	FZ18	FU ZI	1ogs_v	0.9817	GLCM_HUMAN	GBA
MOL002433	FZ18	FU ZI	1md3_v	0.9146	GSTP1_HUMAN	GSTP1
MOL002433	FZ18	FU ZI	2pg2_v	0.9188	KIF11_HUMAN	KIF11
MOL002433	FZ18	FU ZI	2oji_v	0.9593	MK01_HUMAN	MAPK1
MOL002433	FZ18	FU ZI	2gmx_v	0.9216	MK08_HUMAN	MAPK8
MOL002433	FZ18	FU ZI	1pmv_v	0.9621	MK10_HUMAN	MAPK10
MOL002433	FZ18	FU ZI	1smd_v	0.9455	P04745	AMY1A
MOL002433	FZ18	FU ZI	2pin_v	0.9176	P10828	THRB

MOL002433	FZ18	FU ZI	3fzh_v	0.9013	P11142	HSPA8
MOL002433	FZ18	FU ZI	3bgp_v	0.9875	P11309	PIM1
MOL002433	FZ18	FU ZI	1w8l_v	0.9758	P62937	PPIA
MOL002433	FZ18	FU ZI	1xn0_v	0.937	PDE4B_HUMAN	PDE4B
MOL002433	FZ18	FU ZI	1cb0_v	0.905	Q13126	MTAP
MOL002433	FZ18	FU ZI	1q33_v	0.965	Q9BW91	NUDT9
MOL002433	FZ18	FU ZI	1z68_v	0.9822	SEPR_HUMAN	FAP
MOL002433	FZ18	FU ZI	1p49_v	0.9722	STS_HUMAN	STS
MOL002433	FZ18	FU ZI	1m9z_v	0.9321	TGFR2_HUMAN	TGFBR2
MOL002433	FZ18	FU ZI	2roy_v	0.9871	TTHY_HUMAN	TTR
MOL002433	FZ18	FU ZI	1fv9_v	0.9126	UROK_HUMAN	PLAU
MOL002433	FZ18	FU ZI	1j78_v	0.9405	VTDB_HUMAN	GC
MOL002434	FZ19	FU ZI	1j96_v	0.9946	AK1C2_HUMAN	AKR1C2
MOL002434	FZ19	FU ZI	1e7e_v	0.9347	ALBU_HUMAN	ALB
MOL002434	FZ19	FU ZI	1x97_v	0.9623	ALDR_HUMAN	AKR1B1
MOL002434	FZ19	FU ZI	2q7j_v	0.925	ANDR_HUMAN	AR
MOL002434	FZ19	FU ZI	1oj9_v	0.9026	AOFB_HUMAN	MAOB
MOL002434	FZ19	FU ZI	1l6l_v	0.9696	APOA2_HUMAN	APOA2
MOL002434	FZ19	FU ZI	1reu_v	0.9644	BMP2_HUMAN	BMP2
MOL002434	FZ19	FU ZI	1okl_v	0.9466	CAH2_HUMAN	CA2
MOL002434	FZ19	FU ZI	1shj_v	0.9016	CASP7_HUMAN	CASP7
MOL002434	FZ19	FU ZI	1tg6_v	0.9878	CLPP_HUMAN	CLPP
MOL002434	FZ19	FU ZI	1yb1_v	0.9843	DHB11_HUMAN	HSD17B11
MOL002434	FZ19	FU ZI	3czr_v	0.9032	DHI1_HUMAN	HSD11B1
MOL002434	FZ19	FU ZI	2vwu_v	0.9695	EPHB4_HUMAN	EPHB4
MOL002434	FZ19	FU ZI	1ya8_v	0.9529	EST1_HUMAN	CES1
MOL002434	FZ19	FU ZI	2pg2_v	0.9094	KIF11_HUMAN	KIF11
MOL002434	FZ19	FU ZI	2oji_v	0.9412	MK01_HUMAN	MAPK1
MOL002434	FZ19	FU ZI	2pin_v	0.9307	P10828	THRB
MOL002434	FZ19	FU ZI	3bgp_v	0.9819	P11309	PIM1
MOL002434	FZ19	FU ZI	3eqm_v	0.9574	P11511	CYP19A1
MOL002434	FZ19	FU ZI	2p3g_v	0.9531	P49137	[illegible]APKAPK2
MOL002434	FZ19	FU ZI	1zgy_v	0.9142	PPARG_HUMAN	PPARG
MOL002434	FZ19	FU ZI	2ovm_v	0.9456	PRGR_HUMAN	PGR
MOL002434	FZ19	FU ZI	1bl6_v	0.9048	Q16539	MAPK14
MOL002434	FZ19	FU ZI	1tt6_v	0.9363	TTHY_HUMAN	TTR
MOL002388	FZ2	FU ZI	2pio_v	0.937	ANDR_HUMAN	AR
MOL002388	FZ2	FU ZI	1czm_v	0.9504	CAH1_HUMAN	CA1
MOL002388	FZ2	FU ZI	1cnx_v	0.962	CAH2_HUMAN	CA2
MOL002388	FZ2	FU ZI	1h1p_v	0.9398	CCNA2_HUMAN	CCNA2
MOL002388	FZ2	FU ZI	1p0i_v	0.9765	CHLE_HUMAN	BCHE
MOL002388	FZ2	FU ZI	1x7r_v	0.9576	ESR1_HUMAN	ESR1

MOL002388	FZ2	FU ZI	1qkm_v	0.9634	ESR2_HUMAN	ESR2
MOL002388	FZ2	FU ZI	1lbk_v	0.9527	GSTP1_HUMAN	GSTP1
MOL002388	FZ2	FU ZI	2hck_v	0.9905	HCK_HUMAN	HCK
MOL002388	FZ2	FU ZI	1mfu_v	0.9278	P04745	AMY1A
MOL002388	FZ2	FU ZI	1e1x_v	0.9336	P24941	CDK2
MOL002388	FZ2	FU ZI	1w8l_v	0.9638	P62937	PPIA
MOL002388	FZ2	FU ZI	2o63_v	0.9872	PIM1_HUMAN	PIM1
MOL002388	FZ2	FU ZI	1q33_v	0.9344	Q9BW91	NUDT9
MOL002388	FZ2	FU ZI	1m9z_v	0.9174	TGFR2_HUMAN	TGFBR2
MOL000538	FZ20	FU ZI	2bx8_v	0.9212	ALBU_HUMAN	ALB
MOL000538	FZ20	FU ZI	2ama_v	0.9343	ANDR_HUMAN	AR
MOL000538	FZ20	FU ZI	1oj9_v	0.9112	AOFB_HUMAN	MAOB
MOL000538	FZ20	FU ZI	3bmp_v	1	BMP2_HUMAN	BMP2
MOL000538	FZ20	FU ZI	1if4_v	0.9916	CAH2_HUMAN	CA2
MOL000538	FZ20	FU ZI	2uzd_v	0.9417	CCNA2_HUMAN	CCNA2
MOL000538	FZ20	FU ZI	1dic_v	0.9732	CFAD_HUMAN	CFD
MOL000538	FZ20	FU ZI	1xkb_v	0.9097	FA10_HUMAN	F10
MOL000538	FZ20	FU ZI	1uki_v	0.9705	MK08_HUMAN	MAPK8
MOL000538	FZ20	FU ZI	1pmv_v	0.9452	MK10_HUMAN	MAPK10
MOL000538	FZ20	FU ZI	1pq9_v	0.9378	NR1H2_HUMAN	NR1H2
MOL000538	FZ20	FU ZI	1o6u_v	0.9174	O76054	SEC14L2
MOL000538	FZ20	FU ZI	1hpk_v	0.972	P00747	PLG
MOL000538	FZ20	FU ZI	1qmv_v	0.9254	P32119	PRDX2
MOL000538	FZ20	FU ZI	1xlz_v	0.9751	PDE4B_HUMAN	PDE4B
MOL000538	FZ20	FU ZI	1fm6_v	0.9001	PPARG_HUMAN	PPARG
MOL000538	FZ20	FU ZI	1ov4_v	0.9013	Q06520	SULT2A1
MOL000538	FZ20	FU ZI	1cb0_v	0.9325	Q13126	MTAP
MOL000538	FZ20	FU ZI	1iii_v	0.9419	TTHY_HUMAN	TTR
MOL002392	FZ3	FU ZI	1j96_v	0.9888	AK1C2_HUMAN	AKR1C2
MOL002392	FZ3	FU ZI	1xf0_v	0.9034	AK1C3_HUMAN	AKR1C3
MOL002392	FZ3	FU ZI	1e7a_v	0.9982	ALBU_HUMAN	ALB
MOL002392	FZ3	FU ZI	1x97_v	0.9573	ALDR_HUMAN	AKR1B1
MOL002392	FZ3	FU ZI	1xow_v	0.9277	ANDR_HUMAN	AR
MOL002392	FZ3	FU ZI	1reu_v	0.9685	BMP2_HUMAN	BMP2
MOL002392	FZ3	FU ZI	1okl_v	0.9834	CAH2_HUMAN	CA2
MOL002392	FZ3	FU ZI	1gfw_v	0.9972	CASP3_HUMAN	CASP3
MOL002392	FZ3	FU ZI	1rs0_v	0.9787	CFAB_HUMAN	CFB
MOL002392	FZ3	FU ZI	2oax_v	0.9486	MCR_HUMAN	NR3C2
MOL002392	FZ3	FU ZI	2oji_v	0.9164	MK01_HUMAN	MAPK1
MOL002392	FZ3	FU ZI	1pmv_v	0.955	MK10_HUMAN	MAPK10
MOL002392	FZ3	FU ZI	2p3g_v	0.9203	P49137	⁄APKAPK2
MOL002392	FZ3	FU ZI	1w8l_v	0.9919	P62937	PPIA

MOL002392	FZ3	FU ZI	2o65_v	0.9972	PIM1_HUMAN	PIM1
MOL002392	FZ3	FU ZI	1v3q_v	0.945	PNPH_HUMAN	PNP
MOL002392	FZ3	FU ZI	1dvz_v	0.9698	TTHY_HUMAN	TTR
MOL002393	FZ4	FU ZI	2ama_v	0.9346	ANDR_HUMAN	AR
MOL002393	FZ4	FU ZI	1reu_v	1	BMP2_HUMAN	BMP2
MOL002393	FZ4	FU ZI	1czm_v	0.9723	CAH1_HUMAN	CA1
MOL002393	FZ4	FU ZI	1jd0_v	0.9621	CAH12_HUMAN	CA12
MOL002393	FZ4	FU ZI	1if8_v	0.973	CAH2_HUMAN	CA2
MOL002393	FZ4	FU ZI	1gfw_v	0.9124	CASP3_HUMAN	CASP3
MOL002393	FZ4	FU ZI	1lyw_v	0.9148	CATD_HUMAN	CTSD
MOL002393	FZ4	FU ZI	1p0i_v	0.991	CHLE_HUMAN	BCHE
MOL002393	FZ4	FU ZI	1qkt_v	0.9268	ESR1_HUMAN	ESR1
MOL002393	FZ4	FU ZI	1mx1_v	0.9641	EST1_HUMAN	CES1
MOL002393	FZ4	FU ZI	1tow_v	0.9332	FABP4_HUMAN	FABP4
MOL002393	FZ4	FU ZI	7jdw_v	0.9893	GATM_HUMAN	GATM
MOL002393	FZ4	FU ZI	1ogs_v	0.9161	GLCM_HUMAN	GBA
MOL002393	FZ4	FU ZI	1lbk_v	0.9776	GSTP1_HUMAN	GSTP1
MOL002393	FZ4	FU ZI	2g01_v	0.9116	MK08_HUMAN	MAPK8
MOL002393	FZ4	FU ZI	1pmv_v	0.9438	MK10_HUMAN	MAPK10
MOL002393	FZ4	FU ZI	830c_v	0.9415	MMP13_HUMAN	MMP13
MOL002393	FZ4	FU ZI	1bm6_v	0.9474	MMP3_HUMAN	MMP3
MOL002393	FZ4	FU ZI	1qqs_v	0.9985	NGAL_HUMAN	LCN2
MOL002393	FZ4	FU ZI	1pq9_v	0.9631	NR1H2_HUMAN	NR1H2
MOL002393	FZ4	FU ZI	1w8m_v	0.9869	P62937	PPIA
MOL002393	FZ4	FU ZI	1xlz_v	0.9037	PDE4B_HUMAN	PDE4B
MOL002393	FZ4	FU ZI	1yi3_v	0.9788	PIM1_HUMAN	PIM1
MOL002393	FZ4	FU ZI	1v3q_v	0.9526	PNPH_HUMAN	PNP
MOL002393	FZ4	FU ZI	1fm6_v	0.9809	PPARG_HUMAN	PPARG
MOL002393	FZ4	FU ZI	1a28_v	0.9093	PRGR_HUMAN	PGR
MOL002393	FZ4	FU ZI	1w84_v	0.903	Q16539	MAPK14
MOL002393	FZ4	FU ZI	2roy_v	0.9418	TTHY_HUMAN	TTR
MOL002394	FZ5	FU ZI	2pit_v	0.967	ANDR_HUMAN	AR
MOL002394	FZ5	FU ZI	1reu_v	1	BMP2_HUMAN	BMP2
MOL002394	FZ5	FU ZI	1czm_v	0.9702	CAH1_HUMAN	CA1
MOL002394	FZ5	FU ZI	1if8_v	0.9899	CAH2_HUMAN	CA2
MOL002394	FZ5	FU ZI	1gfw_v	0.9637	CASP3_HUMAN	CASP3
MOL002394	FZ5	FU ZI	1fh0_v	0.9657	CATL2_HUMAN	CTSV
MOL002394	FZ5	FU ZI	1rs0_v	0.9411	CFAB_HUMAN	CFB
MOL002394	FZ5	FU ZI	1dic_v	0.9553	CFAD_HUMAN	CFD
MOL002394	FZ5	FU ZI	1p0i_v	0.9812	CHLE_HUMAN	BCHE
MOL002394	FZ5	FU ZI	1vec_v	0.9056	DDX6_HUMAN	DDX6
MOL002394	FZ5	FU ZI	2vwu_v	0.9348	EPHB4_HUMAN	EPHB4

MOL002394	FZ5	FU ZI	1mx1_v	0.9818	EST1_HUMAN	CES1
MOL002394	FZ5	FU ZI	2bq7_v	0.9219	FA10_HUMAN	F10
MOL002394	FZ5	FU ZI	7jdw_v	0.9884	GATM_HUMAN	GATM
MOL002394	FZ5	FU ZI	1ogs_v	0.9143	GLCM_HUMAN	GBA
MOL002394	FZ5	FU ZI	20gs_v	0.9722	GSTP1_HUMAN	GSTP1
MOL002394	FZ5	FU ZI	1pmv_v	0.996	MK10_HUMAN	MAPK10
MOL002394	FZ5	FU ZI	1bm6_v	0.9607	MMP3_HUMAN	MMP3
MOL002394	FZ5	FU ZI	1qqs_v	0.9714	NGAL_HUMAN	LCN2
MOL002394	FZ5	FU ZI	1pq9_v	0.9572	NR1H2_HUMAN	NR1H2
MOL002394	FZ5	FU ZI	3fzk_v	0.9124	P11142	HSPA8
MOL002394	FZ5	FU ZI	3eqm_v	0.952	P11511	CYP19A1
MOL002394	FZ5	FU ZI	2vth_v	0.9482	P24941	CDK2
MOL002394	FZ5	FU ZI	1w8l_v	0.9521	P62937	PPIA
MOL002394	FZ5	FU ZI	1xn0_v	0.911	PDE4B_HUMAN	PDE4B
MOL002394	FZ5	FU ZI	1yi3_v	0.989	PIM1_HUMAN	PIM1
MOL002394	FZ5	FU ZI	1fm6_v	0.9836	PPARG_HUMAN	PPARG
MOL002394	FZ5	FU ZI	1hd2_v	0.9626	PRDX5_HUMAN	PRDX5
MOL002394	FZ5	FU ZI	1a28_v	0.9016	PRGR_HUMAN	PGR
MOL002394	FZ5	FU ZI	1z68_v	0.9535	SEPR_HUMAN	FAP
MOL002394	FZ5	FU ZI	1m9z_v	0.9244	TGFR2_HUMAN	TGFBR2
MOL002394	FZ5	FU ZI	1no9_v	0.9921	THRB_HUMAN	F2
MOL002394	FZ5	FU ZI	1pk2_v	0.9146	TPA_HUMAN	PLAT
MOL002394	FZ5	FU ZI	2roy_v	0.9821	TTHY_HUMAN	TTR
MOL002395	FZ6	FU ZI	1j96_v	1	AK1C2_HUMAN	AKR1C2
MOL002395	FZ6	FU ZI	1e7e_v	1	ALBU_HUMAN	ALB
MOL002395	FZ6	FU ZI	1x97_v	0.9155	ALDR_HUMAN	AKR1B1
MOL002395	FZ6	FU ZI	1l6l_v	0.9766	APOA2_HUMAN	APOA2
MOL002395	FZ6	FU ZI	1reu_v	0.9323	BMP2_HUMAN	BMP2
MOL002395	FZ6	FU ZI	1okl_v	0.9885	CAH2_HUMAN	CA2
MOL002395	FZ6	FU ZI	3dej_v	0.9716	CASP3_HUMAN	CASP3
MOL002395	FZ6	FU ZI	1lyw_v	0.9405	CATD_HUMAN	CTSD
MOL002395	FZ6	FU ZI	2uzd_v	0.9402	CCNA2_HUMAN	CCNA2
MOL002395	FZ6	FU ZI	2brg_v	0.9292	CHK1_HUMAN	CHEK1
MOL002395	FZ6	FU ZI	1p0i_v	0.9679	CHLE_HUMAN	BCHE
MOL002395	FZ6	FU ZI	1klt_v	0.9693	CMA1_HUMAN	CMA1
MOL002395	FZ6	FU ZI	1ya8_v	0.9722	EST1_HUMAN	CES1
MOL002395	FZ6	FU ZI	1lbk_v	0.9905	GSTP1_HUMAN	GSTP1
MOL002395	FZ6	FU ZI	2oji_v	0.9697	MK01_HUMAN	MAPK1
MOL002395	FZ6	FU ZI	2pin_v	0.9327	P10828	THRB
MOL002395	FZ6	FU ZI	2obj_v	0.984	P11309	PIM1
MOL002395	FZ6	FU ZI	3eqm_v	0.9033	P11511	CYP19A1
MOL002395	FZ6	FU ZI	1w8l_v	0.9765	P62937	PPIA

MOL002395	FZ6	FU ZI	1v3q_v	0.94	PNPH_HUMAN	PNP
MOL002395	FZ6	FU ZI	1zuc_v	0.9274	PRGR_HUMAN	PGR
MOL002395	FZ6	FU ZI	1p49_v	1	STS_HUMAN	STS
MOL002397	FZ7	FU ZI	1j96_v	0.9559	AK1C2_HUMAN	AKR1C2
MOL002397	FZ7	FU ZI	1e7e_v	1	ALBU_HUMAN	ALB
MOL002397	FZ7	FU ZI	1x97_v	0.9173	ALDR_HUMAN	AKR1B1
MOL002397	FZ7	FU ZI	1hak_v	0.9438	ANXA5_HUMAN	ANXA5
MOL002397	FZ7	FU ZI	1l6l_v	0.9174	APOA2_HUMAN	APOA2
MOL002397	FZ7	FU ZI	1reu_v	1	BMP2_HUMAN	BMP2
MOL002397	FZ7	FU ZI	1okl_v	0.9219	CAH2_HUMAN	CA2
MOL002397	FZ7	FU ZI	2brm_v	0.9478	CHK1_HUMAN	CHEK1
MOL002397	FZ7	FU ZI	1lbk_v	0.9917	GSTP1_HUMAN	GSTP1
MOL002397	FZ7	FU ZI	1xdd_v	0.9035	ITAL_HUMAN	ITGAL
MOL002397	FZ7	FU ZI	2fky_v	0.9011	KIF11_HUMAN	KIF11
MOL002397	FZ7	FU ZI	1pmv_v	0.9834	MK10_HUMAN	MAPK10
MOL002397	FZ7	FU ZI	3bgq_v	0.9007	P11309	PIM1
MOL002397	FZ7	FU ZI	1w8l_v	0.917	P62937	PPIA
MOL002397	FZ7	FU ZI	1a28_v	0.941	PRGR_HUMAN	PGR
MOL002397	FZ7	FU ZI	1p49_v	1	STS_HUMAN	STS
MOL002397	FZ7	FU ZI	1no9_v	0.9359	THRB_HUMAN	F2
MOL002398	FZ8	FU ZI	1md3_v	0.9404	GSTP1_HUMAN	GSTP1
MOL002401	FZ9	FU ZI	1j96_v	0.9976	AK1C2_HUMAN	AKR1C2
MOL002401	FZ9	FU ZI	1xf0_v	0.9593	AK1C3_HUMAN	AKR1C3
MOL002401	FZ9	FU ZI	1e7a_v	0.975	ALBU_HUMAN	ALB
MOL002401	FZ9	FU ZI	2agt_v	0.9572	ALDR_HUMAN	AKR1B1
MOL002401	FZ9	FU ZI	2pit_v	0.9622	ANDR_HUMAN	AR
MOL002401	FZ9	FU ZI	1oj9_v	0.9934	AOFB_HUMAN	MAOB
MOL002401	FZ9	FU ZI	1reu_v	0.9861	BMP2_HUMAN	BMP2
MOL002401	FZ9	FU ZI	1if8_v	0.9852	CAH2_HUMAN	CA2
MOL002401	FZ9	FU ZI	3dej_v	0.9883	CASP3_HUMAN	CASP3
MOL002401	FZ9	FU ZI	1shj_v	0.9985	CASP7_HUMAN	CASP7
MOL002401	FZ9	FU ZI	1rs0_v	0.9436	CFAB_HUMAN	CFB
MOL002401	FZ9	FU ZI	2brg_v	0.9535	CHK1_HUMAN	CHEK1
MOL002401	FZ9	FU ZI	1p0i_v	0.9237	CHLE_HUMAN	BCHE
MOL002401	FZ9	FU ZI	1yb1_v	0.985	DHB11_HUMAN	HSD17B11
MOL002401	FZ9	FU ZI	1md3_v	0.9477	GSTP1_HUMAN	GSTP1
MOL002401	FZ9	FU ZI	2pg2_v	0.9669	KIF11_HUMAN	KIF11
MOL002401	FZ9	FU ZI	1pme_v	0.9402	MK01_HUMAN	MAPK1
MOL002401	FZ9	FU ZI	1pmv_v	0.969	MK10_HUMAN	MAPK10
MOL002401	FZ9	FU ZI	1eub_v	0.9062	MMP13_HUMAN	MMP13
MOL002401	FZ9	FU ZI	2pin_v	0.9991	P10828	THRB
MOL002401	FZ9	FU ZI	3eqm_v	0.9326	P11511	CYP19A1

MOL002401	FZ9	FU ZI	3cjf_v	0.9528	P35968	KDR
MOL002401	FZ9	FU ZI	2p3g_v	0.9569	P49137	MAPKAPK2
MOL002401	FZ9	FU ZI	3exo_v	0.914	P56817	BACE1
MOL002401	FZ9	FU ZI	1w8l_v	0.9859	P62937	PPIA
MOL002401	FZ9	FU ZI	2o65_v	0.9535	PIM1_HUMAN	PIM1
MOL002401	FZ9	FU ZI	1zgy_v	0.9882	PPARG_HUMAN	PPARG
MOL002401	FZ9	FU ZI	1w7h_v	0.944	Q16539	MAPK14
MOL002401	FZ9	FU ZI	1p49_v	0.968	STS_HUMAN	STS
MOL002401	FZ9	FU ZI	1f86_v	0.9817	TTHY_HUMAN	TTR
MOL002401	FZ9	FU ZI	1j78_v	1	VTDB_HUMAN	GC
MOL000211	GC1	GAN CAO	1j96_v	0.9843	AK1C2_HUMAN	AKR1C2
MOL000211	GC1	GAN CAO	1e7e_v	0.974	ALBU_HUMAN	ALB
MOL000211	GC1	GAN CAO	1l6l_v	0.9012	APOA2_HUMAN	APOA2
MOL000211	GC1	GAN CAO	1reu_v	0.9742	BMP2_HUMAN	BMP2
MOL000211	GC1	GAN CAO	2nnv_v	0.9358	CAH2_HUMAN	CA2
MOL000211	GC1	GAN CAO	2vwu_v	0.9023	EPHB4_HUMAN	EPHB4
MOL000211	GC1	GAN CAO	3cgf_v	0.9476	MK10_HUMAN	MAPK10
MOL000211	GC1	GAN CAO	3bgp_v	0.9836	P11309	PIM1
MOL000211	GC1	GAN CAO	3cjf_v	0.9476	P35968	KDR
MOL000211	GC1	GAN CAO	2p3g_v	0.9649	P49137	MAPKAPK2
MOL000211	GC1	GAN CAO	1w8l_v	0.9489	P62937	PPIA
MOL000211	GC1	GAN CAO	1p49_v	0.9788	STS_HUMAN	STS
MOL000211	GC1	GAN CAO	1j78_v	0.9174	VTDB_HUMAN	GC
MOL004328	GC10	GAN CAO	2ama_v	0.9529	ANDR_HUMAN	AR
MOL004328	GC10	GAN CAO	2ohp_v	0.9645	BACE1_HUMAN	BACE1
MOL004328	GC10	GAN CAO	2osf_v	0.972	CAH2_HUMAN	CA2
MOL004328	GC10	GAN CAO	1p0i_v	0.9622	CHLE_HUMAN	BCHE
MOL004328	GC10	GAN CAO	2itx_v	0.9418	EGFR_HUMAN	EGFR
MOL004328	GC10	GAN CAO	1uki_v	0.9024	MK08_HUMAN	MAPK8
MOL004328	GC10	GAN CAO	3cgf_v	0.9435	MK10_HUMAN	MAPK10
MOL004328	GC10	GAN CAO	3cjf_v	0.9436	P35968	KDR
MOL004805	GC11	GAN CAO	1j96_v	0.9895	AK1C2_HUMAN	AKR1C2
MOL004805	GC11	GAN CAO	1e7e_v	0.9856	ALBU_HUMAN	ALB
MOL004805	GC11	GAN CAO	2pir_v	0.9409	ANDR_HUMAN	AR
MOL004805	GC11	GAN CAO	1oj9_v	0.9352	AOFB_HUMAN	MAOB
MOL004805	GC11	GAN CAO	1l6l_v	0.9915	APOA2_HUMAN	APOA2
MOL004805	GC11	GAN CAO	2ohp_v	0.9028	BACE1_HUMAN	BACE1
MOL004805	GC11	GAN CAO	1reu_v	0.922	BMP2_HUMAN	BMP2
MOL004805	GC11	GAN CAO	1if4_v	0.9693	CAH2_HUMAN	CA2
MOL004805	GC11	GAN CAO	3dej_v	0.9903	CASP3_HUMAN	CASP3
MOL004805	GC11	GAN CAO	1shj_v	0.9815	CASP7_HUMAN	CASP7
MOL004805	GC11	GAN CAO	1rs0_v	0.9972	CFAB_HUMAN	CFB

MOL004805	GC11	GAN CAO	2vwu_v	0.9462	EPHB4_HUMAN	EPHB4
MOL004805	GC11	GAN CAO	1qku_v	0.9687	ESR1_HUMAN	ESR1
MOL004805	GC11	GAN CAO	2fky_v	0.9204	KIF11_HUMAN	KIF11
MOL004805	GC11	GAN CAO	1pme_v	0.9396	MK01_HUMAN	MAPK1
MOL004805	GC11	GAN CAO	1uki_v	0.9563	MK08_HUMAN	MAPK8
MOL004805	GC11	GAN CAO	3cgf_v	0.9812	MK10_HUMAN	MAPK10
MOL004805	GC11	GAN CAO	1eub_v	0.9682	MMP13_HUMAN	MMP13
MOL004805	GC11	GAN CAO	2pin_v	0.9153	P10828	THRB
MOL004805	GC11	GAN CAO	2obj_v	0.9959	P11309	PIM1
MOL004805	GC11	GAN CAO	3eqm_v	0.9692	P11511	CYP19A1
MOL004805	GC11	GAN CAO	3cjf_v	0.9812	P35968	KDR
MOL004805	GC11	GAN CAO	2jbp_v	0.9825	P49137	MAPKAPK2
MOL004805	GC11	GAN CAO	1w8l_v	0.9559	P62937	PPIA
MOL004805	GC11	GAN CAO	2a4z_v	0.9597	PK3CG_HUMAN	PIK3CG
MOL004805	GC11	GAN CAO	1zgy_v	0.9491	PPARG_HUMAN	PPARG
MOL004805	GC11	GAN CAO	2ovm_v	0.9617	PRGR_HUMAN	PGR
MOL004805	GC11	GAN CAO	1wbo_v	0.9992	Q16539	MAPK14
MOL004805	GC11	GAN CAO	1p49_v	0.9752	STS_HUMAN	STS
MOL004805	GC11	GAN CAO	1no9_v	0.9382	THRB_HUMAN	F2
MOL004805	GC11	GAN CAO	1dvz_v	0.9656	TTHY_HUMAN	TTR
MOL004806	GC12	GAN CAO	1j96_v	0.9966	AK1C2_HUMAN	AKR1C2
MOL004806	GC12	GAN CAO	1e7e_v	0.9475	ALBU_HUMAN	ALB
MOL004806	GC12	GAN CAO	1oj9_v	0.9878	AOFB_HUMAN	MAOB
MOL004806	GC12	GAN CAO	1l6l_v	0.9713	APOA2_HUMAN	APOA2
MOL004806	GC12	GAN CAO	1ze8_v	0.9742	CAH2_HUMAN	CA2
MOL004806	GC12	GAN CAO	3dej_v	0.9449	CASP3_HUMAN	CASP3
MOL004806	GC12	GAN CAO	1shj_v	0.9708	CASP7_HUMAN	CASP7
MOL004806	GC12	GAN CAO	2bkz_v	0.9007	CCNA2_HUMAN	CCNA2
MOL004806	GC12	GAN CAO	1rs0_v	0.9986	CFAB_HUMAN	CFB
MOL004806	GC12	GAN CAO	2brb_v	0.9906	CHK1_HUMAN	CHEK1
MOL004806	GC12	GAN CAO	1ere_v	0.9175	ESR1_HUMAN	ESR1
MOL004806	GC12	GAN CAO	1ya8_v	0.9504	EST1_HUMAN	CES1
MOL004806	GC12	GAN CAO	2j38_v	0.9353	FA10_HUMAN	F10
MOL004806	GC12	GAN CAO	1f40_v	0.9015	FKB1A_HUMAN	FKBP1A
MOL004806	GC12	GAN CAO	2uyi_v	0.9489	KIF11_HUMAN	KIF11
MOL004806	GC12	GAN CAO	1pme_v	0.9799	MK01_HUMAN	MAPK1
MOL004806	GC12	GAN CAO	1eub_v	0.9767	MMP13_HUMAN	MMP13
MOL004806	GC12	GAN CAO	1bm6_v	0.9254	MMP3_HUMAN	MMP3
MOL004806	GC12	GAN CAO	3f5p_v	0.9339	P08069	IGF1R
MOL004806	GC12	GAN CAO	2pin_v	0.9667	P10828	THRB
MOL004806	GC12	GAN CAO	2obj_v	0.9873	P11309	PIM1
MOL004806	GC12	GAN CAO	2p3g_v	0.9858	P49137	MAPKAPK2

MOL004806	GC12	GAN CAO	2a4z_v	0.9928	PK3CG_HUMAN	PIK3CG
MOL004806	GC12	GAN CAO	2ovm_v	0.9419	PRGR_HUMAN	PGR
MOL004806	GC12	GAN CAO	1w7h_v	0.9275	Q16539	MAPK14
MOL004806	GC12	GAN CAO	1p49_v	0.9733	STS_HUMAN	STS
MOL004806	GC12	GAN CAO	1no9_v	0.9311	THRB_HUMAN	F2
MOL004806	GC12	GAN CAO	1dvz_v	0.9841	TTHY_HUMAN	TTR
MOL004808	GC13	GAN CAO	1e7e_v	0.9419	ALBU_HUMAN	ALB
MOL004808	GC13	GAN CAO	2pit_v	0.9819	ANDR_HUMAN	AR
MOL004808	GC13	GAN CAO	1l6l_v	0.9659	APOA2_HUMAN	APOA2
MOL004808	GC13	GAN CAO	2ohp_v	0.9279	BACE1_HUMAN	BACE1
MOL004808	GC13	GAN CAO	1czm_v	0.9691	CAH1_HUMAN	CA1
MOL004808	GC13	GAN CAO	1zfq_v	0.9037	CAH2_HUMAN	CA2
MOL004808	GC13	GAN CAO	1shj_v	0.9654	CASP7_HUMAN	CASP7
MOL004808	GC13	GAN CAO	1rs0_v	0.9804	CFAB_HUMAN	CFB
MOL004808	GC13	GAN CAO	1xlu_v	0.9675	CHLE_HUMAN	BCHE
MOL004808	GC13	GAN CAO	2vwu_v	0.9235	EPHB4_HUMAN	EPHB4
MOL004808	GC13	GAN CAO	1qku_v	0.9327	ESR1_HUMAN	ESR1
MOL004808	GC13	GAN CAO	1qkm_v	0.9786	ESR2_HUMAN	ESR2
MOL004808	GC13	GAN CAO	1ya8_v	0.9521	EST1_HUMAN	CES1
MOL004808	GC13	GAN CAO	2uyi_v	0.9677	KIF11_HUMAN	KIF11
MOL004808	GC13	GAN CAO	1pme_v	0.9615	MK01_HUMAN	MAPK1
MOL004808	GC13	GAN CAO	1uki_v	0.9624	MK08_HUMAN	MAPK8
MOL004808	GC13	GAN CAO	1pmv_v	0.9772	MK10_HUMAN	MAPK10
MOL004808	GC13	GAN CAO	1bm6_v	0.9733	MMP3_HUMAN	MMP3
MOL004808	GC13	GAN CAO	3eko_v	0.9412	P07900	HSP90AA1
MOL004808	GC13	GAN CAO	1jvp_v	0.955	P24941	CDK2
MOL004808	GC13	GAN CAO	3cjf_v	0.9513	P35968	KDR
MOL004808	GC13	GAN CAO	2jbp_v	0.9195	P49137	MAPKAPK2
MOL004808	GC13	GAN CAO	1yxx_v	0.9836	PIM1_HUMAN	PIM1
MOL004808	GC13	GAN CAO	1v3q_v	0.9294	PNPH_HUMAN	PNP
MOL004808	GC13	GAN CAO	1wbw_v	0.9915	Q16539	MAPK14
MOL004808	GC13	GAN CAO	1q8m_v	0.9839	Q9NP99	TREM1
MOL004808	GC13	GAN CAO	1p49_v	0.9707	STS_HUMAN	STS
MOL004808	GC13	GAN CAO	1dvz_v	0.9764	TTHY_HUMAN	TTR
MOL004810	GC14	GAN CAO	1j96_v	0.9576	AK1C2_HUMAN	AKR1C2
MOL004810	GC14	GAN CAO	1e7a_v	0.9248	ALBU_HUMAN	ALB
MOL004810	GC14	GAN CAO	2pir_v	0.948	ANDR_HUMAN	AR
MOL004810	GC14	GAN CAO	2v5z_v	0.9374	AOFB_HUMAN	MAOB
MOL004810	GC14	GAN CAO	2ohp_v	0.9825	BACE1_HUMAN	BACE1
MOL004810	GC14	GAN CAO	1reu_v	0.9076	BMP2_HUMAN	BMP2
MOL004810	GC14	GAN CAO	1czm_v	0.9686	CAH1_HUMAN	CA1
MOL004810	GC14	GAN CAO	3dej_v	0.9372	CASP3_HUMAN	CASP3

MOL004810	GC14	GAN CAO	1rs0_v	0.9686	CFAB_HUMAN	CFB
MOL004810	GC14	GAN CAO	2brg_v	0.9489	CHK1_HUMAN	CHEK1
MOL004810	GC14	GAN CAO	1xlu_v	0.9657	CHLE_HUMAN	BCHE
MOL004810	GC14	GAN CAO	2vwu_v	0.9866	EPHB4_HUMAN	EPHB4
MOL004810	GC14	GAN CAO	2i0j_v	0.9918	ESR1_HUMAN	ESR1
MOL004810	GC14	GAN CAO	1x7j_v	0.9686	ESR2_HUMAN	ESR2
MOL004810	GC14	GAN CAO	1ya8_v	0.9679	EST1_HUMAN	CES1
MOL004810	GC14	GAN CAO	1lbk_v	0.9156	GSTP1_HUMAN	GSTP1
MOL004810	GC14	GAN CAO	2hck_v	0.9166	HCK_HUMAN	HCK
MOL004810	GC14	GAN CAO	2pg2_v	0.9841	KIF11_HUMAN	KIF11
MOL004810	GC14	GAN CAO	1pme_v	0.9562	MK01_HUMAN	MAPK1
MOL004810	GC14	GAN CAO	1uki_v	0.9902	MK08_HUMAN	MAPK8
MOL004810	GC14	GAN CAO	1pmv_v	0.9889	MK10_HUMAN	MAPK10
MOL004810	GC14	GAN CAO	1eub_v	0.9905	MMP13_HUMAN	MMP13
MOL004810	GC14	GAN CAO	1bm6_v	0.9216	MMP3_HUMAN	MMP3
MOL004810	GC14	GAN CAO	2pin_v	0.9161	P10828	THRB
MOL004810	GC14	GAN CAO	3eqm_v	0.9246	P11511	CYP19A1
MOL004810	GC14	GAN CAO	3gam_v	0.9203	P16083	NQO2
MOL004810	GC14	GAN CAO	3cjf_v	0.9468	P35968	KDR
MOL004810	GC14	GAN CAO	2p3g_v	0.9343	P49137	MAPKAPK2
MOL004810	GC14	GAN CAO	1w8m_v	0.9675	P62937	PPIA
MOL004810	GC14	GAN CAO	1yxx_v	0.9891	PIM1_HUMAN	PIM1
MOL004810	GC14	GAN CAO	1v3q_v	0.9456	PNPH_HUMAN	PNP
MOL004810	GC14	GAN CAO	2ovm_v	0.9746	PRGR_HUMAN	PGR
MOL004810	GC14	GAN CAO	1w7h_v	0.9748	Q16539	MAPK14
MOL004810	GC14	GAN CAO	1q8m_v	0.9857	Q9NP99	TREM1
MOL004810	GC14	GAN CAO	1no9_v	0.9131	THRB_HUMAN	F2
MOL004810	GC14	GAN CAO	1dvz_v	0.9594	TTHY_HUMAN	TTR
MOL004811	GC15	GAN CAO	1e7e_v	0.9604	ALBU_HUMAN	ALB
MOL004811	GC15	GAN CAO	2pir_v	0.9007	ANDR_HUMAN	AR
MOL004811	GC15	GAN CAO	1oj9_v	0.9967	AOFB_HUMAN	MAOB
MOL004811	GC15	GAN CAO	1l6l_v	0.9436	APOA2_HUMAN	APOA2
MOL004811	GC15	GAN CAO	1okl_v	0.9602	CAH2_HUMAN	CA2
MOL004811	GC15	GAN CAO	1shj_v	0.9537	CASP7_HUMAN	CASP7
MOL004811	GC15	GAN CAO	1fh0_v	0.9578	CATL2_HUMAN	CTSV
MOL004811	GC15	GAN CAO	1x7r_v	0.9643	ESR1_HUMAN	ESR1
MOL004811	GC15	GAN CAO	1qkm_v	0.9831	ESR2_HUMAN	ESR2
MOL004811	GC15	GAN CAO	1ya8_v	0.9967	EST1_HUMAN	CES1
MOL004811	GC15	GAN CAO	2uyi_v	0.9738	KIF11_HUMAN	KIF11
MOL004811	GC15	GAN CAO	1pme_v	0.9687	MK01_HUMAN	MAPK1
MOL004811	GC15	GAN CAO	1pmv_v	0.9582	MK10_HUMAN	MAPK10
MOL004811	GC15	GAN CAO	1bm6_v	0.9323	MMP3_HUMAN	MMP3

MOL004811	GC15	GAN CAO	3cjf_v	0.9085	P35968	KDR
MOL004811	GC15	GAN CAO	2p3g_v	0.968	P49137	MAPKAPK2
MOL004811	GC15	GAN CAO	2o65_v	0.9664	PIM1_HUMAN	PIM1
MOL004811	GC15	GAN CAO	2a4z_v	0.9402	PK3CG_HUMAN	PIK3CG
MOL004811	GC15	GAN CAO	1w7h_v	0.9731	Q16539	MAPK14
MOL004811	GC15	GAN CAO	1q8m_v	0.9866	Q9NP99	TREM1
MOL004811	GC15	GAN CAO	1p49_v	0.9505	STS_HUMAN	STS
MOL004811	GC15	GAN CAO	1no9_v	0.9185	THRB_HUMAN	F2
MOL004811	GC15	GAN CAO	2roy_v	0.9371	TTHY_HUMAN	TTR
MOL004811	GC15	GAN CAO	1j78_v	0.9064	VTDB_HUMAN	GC
MOL004814	GC16	GAN CAO	1e7e_v	0.906	ALBU_HUMAN	ALB
MOL004814	GC16	GAN CAO	1xj7_v	0.941	ANDR_HUMAN	AR
MOL004814	GC16	GAN CAO	1l6l_v	0.9683	APOA2_HUMAN	APOA2
MOL004814	GC16	GAN CAO	1kwq_v	0.9653	CAH2_HUMAN	CA2
MOL004814	GC16	GAN CAO	1rs0_v	0.9577	CFAB_HUMAN	CFB
MOL004814	GC16	GAN CAO	2ocf_v	0.9981	ESR1_HUMAN	ESR1
MOL004814	GC16	GAN CAO	2i0g_v	0.9889	ESR2_HUMAN	ESR2
MOL004814	GC16	GAN CAO	1ya8_v	0.9857	EST1_HUMAN	CES1
MOL004814	GC16	GAN CAO	1uki_v	0.9447	MK08_HUMAN	MAPK8
MOL004814	GC16	GAN CAO	1pmv_v	0.962	MK10_HUMAN	MAPK10
MOL004814	GC16	GAN CAO	1bm6_v	0.9329	MMP3_HUMAN	MMP3
MOL004814	GC16	GAN CAO	3eqm_v	0.9068	P11511	CYP19A1
MOL004814	GC16	GAN CAO	1xlz_v	0.9692	PDE4B_HUMAN	PDE4B
MOL004814	GC16	GAN CAO	2pe0_v	0.9294	PDPK1_HUMAN	PDPK1
MOL004814	GC16	GAN CAO	1yxx_v	0.9819	PIM1_HUMAN	PIM1
MOL004814	GC16	GAN CAO	1p49_v	0.9725	STS_HUMAN	STS
MOL004814	GC16	GAN CAO	1no9_v	0.9723	THRB_HUMAN	F2
MOL004815	GC17	GAN CAO	1shj_v	0.9816	CASP7_HUMAN	CASP7
MOL004815	GC17	GAN CAO	1p0i_v	0.9558	CHLE_HUMAN	BCHE
MOL004815	GC17	GAN CAO	2i0j_v	0.9734	ESR1_HUMAN	ESR1
MOL004815	GC17	GAN CAO	1ya8_v	0.9651	EST1_HUMAN	CES1
MOL004815	GC17	GAN CAO	1ogs_v	0.9752	GLCM_HUMAN	GBA
MOL004815	GC17	GAN CAO	1lbk_v	0.9438	GSTP1_HUMAN	GSTP1
MOL004815	GC17	GAN CAO	2hck_v	0.9272	HCK_HUMAN	HCK
MOL004815	GC17	GAN CAO	3dcw_v	0.9002	P00918	CA2
MOL004815	GC17	GAN CAO	1yxx_v	0.9678	PIM1_HUMAN	PIM1
MOL004815	GC17	GAN CAO	2a4z_v	0.9552	PK3CG_HUMAN	PIK3CG
MOL004815	GC17	GAN CAO	2qd9_v	0.9623	Q16539	MAPK14
MOL004815	GC17	GAN CAO	2roy_v	0.9787	TTHY_HUMAN	TTR
MOL004820	GC18	GAN CAO	1j96_v	0.9304	AK1C2_HUMAN	AKR1C2
MOL004820	GC18	GAN CAO	1xf0_v	0.903	AK1C3_HUMAN	AKR1C3
MOL004820	GC18	GAN CAO	1e7a_v	0.9426	ALBU_HUMAN	ALB

MOL004820	GC18	GAN CAO	1xow_v	0.9699	ANDR_HUMAN	AR
MOL004820	GC18	GAN CAO	1reu_v	0.9198	BMP2_HUMAN	BMP2
MOL004820	GC18	GAN CAO	2qo8_v	0.9176	CAH2_HUMAN	CA2
MOL004820	GC18	GAN CAO	1gfw_v	0.9264	CASP3_HUMAN	CASP3
MOL004820	GC18	GAN CAO	2uzd_v	0.9196	CCNA2_HUMAN	CCNA2
MOL004820	GC18	GAN CAO	2ocf_v	0.9359	ESR1_HUMAN	ESR1
MOL004820	GC18	GAN CAO	1qkm_v	0.9815	ESR2_HUMAN	ESR2
MOL004820	GC18	GAN CAO	1ya8_v	0.9385	EST1_HUMAN	CES1
MOL004820	GC18	GAN CAO	1pme_v	0.9201	MK01_HUMAN	MAPK1
MOL004820	GC18	GAN CAO	1pmv_v	0.9942	MK10_HUMAN	MAPK10
MOL004820	GC18	GAN CAO	1bm6_v	0.9494	MMP3_HUMAN	MMP3
MOL004820	GC18	GAN CAO	1yxx_v	0.9852	PIM1_HUMAN	PIM1
MOL004820	GC18	GAN CAO	1wbw_v	0.9781	Q16539	MAPK14
MOL004820	GC18	GAN CAO	1dvz_v	0.9767	TTHY_HUMAN	TTR
MOL004824	GC19	GAN CAO	2pir_v	0.9433	ANDR_HUMAN	AR
MOL004824	GC19	GAN CAO	1if4_v	0.9706	CAH2_HUMAN	CA2
MOL004824	GC19	GAN CAO	1rs0_v	0.9268	CFAB_HUMAN	CFB
MOL004824	GC19	GAN CAO	1p0i_v	0.9595	CHLE_HUMAN	BCHE
MOL004824	GC19	GAN CAO	1klt_v	0.9101	CMA1_HUMAN	CMA1
MOL004824	GC19	GAN CAO	2i0j_v	0.9324	ESR1_HUMAN	ESR1
MOL004824	GC19	GAN CAO	1qkm_v	0.942	ESR2_HUMAN	ESR2
MOL004824	GC19	GAN CAO	1ya8_v	0.9442	EST1_HUMAN	CES1
MOL004824	GC19	GAN CAO	1d7i_v	0.9231	FKB1A_HUMAN	FKBP1A
MOL004824	GC19	GAN CAO	1ogs_v	0.9637	GLCM_HUMAN	GBA
MOL004824	GC19	GAN CAO	1uki_v	0.9907	MK08_HUMAN	MAPK8
MOL004824	GC19	GAN CAO	1pmv_v	0.9962	MK10_HUMAN	MAPK10
MOL004824	GC19	GAN CAO	1bm6_v	0.9597	MMP3_HUMAN	MMP3
MOL004824	GC19	GAN CAO	1w8l_v	0.9438	P62937	PPIA
MOL004824	GC19	GAN CAO	1xn0_v	0.9153	PDE4B_HUMAN	PDE4B
MOL004824	GC19	GAN CAO	1yxx_v	0.9864	PIM1_HUMAN	PIM1
MOL004824	GC19	GAN CAO	1wbw_v	0.9931	Q16539	MAPK14
MOL004824	GC19	GAN CAO	1q8m_v	0.9562	Q9NP99	TREM1
MOL002311	GC2	GAN CAO	1e7e_v	0.9091	ALBU_HUMAN	ALB
MOL002311	GC2	GAN CAO	1xj7_v	0.9248	ANDR_HUMAN	AR
MOL002311	GC2	GAN CAO	1oj9_v	0.9059	AOFB_HUMAN	MAOB
MOL002311	GC2	GAN CAO	1l6l_v	0.9138	APOA2_HUMAN	APOA2
MOL002311	GC2	GAN CAO	2ohp_v	0.9174	BACE1_HUMAN	BACE1
MOL002311	GC2	GAN CAO	1reu_v	0.9902	BMP2_HUMAN	BMP2
MOL002311	GC2	GAN CAO	2nnv_v	0.9864	CAH2_HUMAN	CA2
MOL002311	GC2	GAN CAO	1shj_v	0.9502	CASP7_HUMAN	CASP7
MOL002311	GC2	GAN CAO	2uzd_v	0.9201	CCNA2_HUMAN	CCNA2
MOL002311	GC2	GAN CAO	1unh_v	0.9324	CD5R1_HUMAN	CDK5R1

MOL002311	GC2	GAN CAO	1rs0_v	0.9732	CFAB_HUMAN	CFB
MOL002311	GC2	GAN CAO	1p0i_v	0.9585	CHLE_HUMAN	BCHE
MOL002311	GC2	GAN CAO	3czr_v	0.9146	DHI1_HUMAN	HSD11B1
MOL002311	GC2	GAN CAO	2ocf_v	0.9902	ESR1_HUMAN	ESR1
MOL002311	GC2	GAN CAO	2jj3_v	0.963	ESR2_HUMAN	ESR2
MOL002311	GC2	GAN CAO	1ya8_v	0.9095	EST1_HUMAN	CES1
MOL002311	GC2	GAN CAO	1pme_v	0.998	MK01_HUMAN	MAPK1
MOL002311	GC2	GAN CAO	1uki_v	0.9225	MK08_HUMAN	MAPK8
MOL002311	GC2	GAN CAO	3cgf_v	0.959	MK10_HUMAN	MAPK10
MOL002311	GC2	GAN CAO	3bgp_v	0.9868	P11309	PIM1
MOL002311	GC2	GAN CAO	1jvp_v	0.946	P24941	CDK2
MOL002311	GC2	GAN CAO	3cjf_v	0.9589	P35968	KDR
MOL002311	GC2	GAN CAO	1v3q_v	0.9145	PNPH_HUMAN	PNP
MOL002311	GC2	GAN CAO	1fm6_v	0.9571	PPARG_HUMAN	PPARG
MOL002311	GC2	GAN CAO	1wbo_v	0.9968	Q16539	MAPK14
MOL002311	GC2	GAN CAO	1p49_v	0.9654	STS_HUMAN	STS
MOL002311	GC2	GAN CAO	1dvz_v	0.9225	TTHY_HUMAN	TTR
MOL002311	GC2	GAN CAO	1j78_v	0.9007	VTDB_HUMAN	GC
MOL004827	GC20	GAN CAO	1j96_v	0.9598	AK1C2_HUMAN	AKR1C2
MOL004827	GC20	GAN CAO	2pir_v	0.9001	ANDR_HUMAN	AR
MOL004827	GC20	GAN CAO	1reu_v	0.9192	BMP2_HUMAN	BMP2
MOL004827	GC20	GAN CAO	2osf_v	0.9724	CAH2_HUMAN	CA2
MOL004827	GC20	GAN CAO	1rs0_v	0.9336	CFAB_HUMAN	CFB
MOL004827	GC20	GAN CAO	1p0i_v	0.9597	CHLE_HUMAN	BCHE
MOL004827	GC20	GAN CAO	2i0j_v	0.9385	ESR1_HUMAN	ESR1
MOL004827	GC20	GAN CAO	1qkm_v	0.9117	ESR2_HUMAN	ESR2
MOL004827	GC20	GAN CAO	1ya8_v	0.9164	EST1_HUMAN	CES1
MOL004827	GC20	GAN CAO	1ogs_v	0.9252	GLCM_HUMAN	GBA
MOL004827	GC20	GAN CAO	2o65_v	0.9506	PIM1_HUMAN	PIM1
MOL004827	GC20	GAN CAO	1w7h_v	0.9426	Q16539	MAPK14
MOL004827	GC20	GAN CAO	1z68_v	0.9373	SEPR_HUMAN	FAP
MOL004827	GC20	GAN CAO	1no9_v	0.96	THRB_HUMAN	F2
MOL004828	GC21	GAN CAO	1j96_v	0.9857	AK1C2_HUMAN	AKR1C2
MOL004828	GC21	GAN CAO	1e7e_v	0.9058	ALBU_HUMAN	ALB
MOL004828	GC21	GAN CAO	2pit_v	0.9824	ANDR_HUMAN	AR
MOL004828	GC21	GAN CAO	1l6l_v	0.9402	APOA2_HUMAN	APOA2
MOL004828	GC21	GAN CAO	1reu_v	0.9678	BMP2_HUMAN	BMP2
MOL004828	GC21	GAN CAO	2osf_v	0.97	CAH2_HUMAN	CA2
MOL004828	GC21	GAN CAO	3dej_v	0.9617	CASP3_HUMAN	CASP3
MOL004828	GC21	GAN CAO	1shj_v	0.9402	CASP7_HUMAN	CASP7
MOL004828	GC21	GAN CAO	1h1p_v	0.9012	CCNA2_HUMAN	CCNA2
MOL004828	GC21	GAN CAO	1rs0_v	0.951	CFAB_HUMAN	CFB

MOL004828	GC21	GAN CAO	2brb_v	0.9997	CHK1_HUMAN	CHEK1
MOL004828	GC21	GAN CAO	1xlu_v	0.9722	CHLE_HUMAN	BCHE
MOL004828	GC21	GAN CAO	2itx_v	0.9497	EGFR_HUMAN	EGFR
MOL004828	GC21	GAN CAO	2vwu_v	0.9627	EPHB4_HUMAN	EPHB4
MOL004828	GC21	GAN CAO	2ocf_v	0.9494	ESR1_HUMAN	ESR1
MOL004828	GC21	GAN CAO	1ya8_v	0.9169	EST1_HUMAN	CES1
MOL004828	GC21	GAN CAO	1lbk_v	0.9711	GSTP1_HUMAN	GSTP1
MOL004828	GC21	GAN CAO	2hck_v	0.9856	HCK_HUMAN	HCK
MOL004828	GC21	GAN CAO	2pg2_v	0.9837	KIF11_HUMAN	KIF11
MOL004828	GC21	GAN CAO	3cgf_v	0.9459	MK10_HUMAN	MAPK10
MOL004828	GC21	GAN CAO	1eub_v	0.9913	MMP13_HUMAN	MMP13
MOL004828	GC21	GAN CAO	1bm6_v	0.9264	MMP3_HUMAN	MMP3
MOL004828	GC21	GAN CAO	1kbo_v	0.9183	NQO1_HUMAN	NQO1
MOL004828	GC21	GAN CAO	2pin_v	0.924	P10828	THRB
MOL004828	GC21	GAN CAO	3fzh_v	0.9487	P11142	HSPA8
MOL004828	GC21	GAN CAO	2obj_v	0.9587	P11309	PIM1
MOL004828	GC21	GAN CAO	3eqm_v	0.9501	P11511	CYP19A1
MOL004828	GC21	GAN CAO	3cjf_v	0.946	P35968	KDR
MOL004828	GC21	GAN CAO	1p49_v	0.9509	STS_HUMAN	STS
MOL004828	GC21	GAN CAO	1dvz_v	0.993	TTHY_HUMAN	TTR
MOL004829	GC22	GAN CAO	1j96_v	0.9912	AK1C2_HUMAN	AKR1C2
MOL004829	GC22	GAN CAO	1e7e_v	0.9169	ALBU_HUMAN	ALB
MOL004829	GC22	GAN CAO	2pit_v	0.9504	ANDR_HUMAN	AR
MOL004829	GC22	GAN CAO	1l6l_v	0.9474	APOA2_HUMAN	APOA2
MOL004829	GC22	GAN CAO	1reu_v	0.9371	BMP2_HUMAN	BMP2
MOL004829	GC22	GAN CAO	1if4_v	0.9619	CAH2_HUMAN	CA2
MOL004829	GC22	GAN CAO	1shj_v	0.9663	CASP7_HUMAN	CASP7
MOL004829	GC22	GAN CAO	1rs0_v	0.9577	CFAB_HUMAN	CFB
MOL004829	GC22	GAN CAO	2brb_v	0.9983	CHK1_HUMAN	CHEK1
MOL004829	GC22	GAN CAO	1p0i_v	0.9465	CHLE_HUMAN	BCHE
MOL004829	GC22	GAN CAO	1yb1_v	0.9057	DHB11_HUMAN	HSD17B11
MOL004829	GC22	GAN CAO	2itx_v	0.916	EGFR_HUMAN	EGFR
MOL004829	GC22	GAN CAO	2vwu_v	0.9101	EPHB4_HUMAN	EPHB4
MOL004829	GC22	GAN CAO	1qku_v	0.949	ESR1_HUMAN	ESR1
MOL004829	GC22	GAN CAO	1f40_v	0.9513	FKB1A_HUMAN	FKBP1A
MOL004829	GC22	GAN CAO	1lbk_v	0.9494	GSTP1_HUMAN	GSTP1
MOL004829	GC22	GAN CAO	2hck_v	0.9903	HCK_HUMAN	HCK
MOL004829	GC22	GAN CAO	2pg2_v	0.9511	KIF11_HUMAN	KIF11
MOL004829	GC22	GAN CAO	1pme_v	0.9073	MK01_HUMAN	MAPK1
MOL004829	GC22	GAN CAO	1uki_v	0.9097	MK08_HUMAN	MAPK8
MOL004829	GC22	GAN CAO	1pmv_v	0.9303	MK10_HUMAN	MAPK10
MOL004829	GC22	GAN CAO	2obj_v	0.9541	P11309	PIM1

MOL004829	GC22	GAN CAO	3exo_v	0.9481	P56817	BACE1
MOL004829	GC22	GAN CAO	1w8l_v	0.9525	P62937	PPIA
MOL004829	GC22	GAN CAO	1wbo_v	0.9792	Q16539	MAPK14
MOL004829	GC22	GAN CAO	1p49_v	0.9573	STS_HUMAN	STS
MOL004829	GC22	GAN CAO	1dvz_v	0.9819	TTHY_HUMAN	TTR
MOL004833	GC23	GAN CAO	1e7e_v	0.9567	ALBU_HUMAN	ALB
MOL004833	GC23	GAN CAO	1oj9_v	0.9626	AOFB_HUMAN	MAOB
MOL004833	GC23	GAN CAO	2ohp_v	0.9918	BACE1_HUMAN	BACE1
MOL004833	GC23	GAN CAO	1if4_v	0.9683	CAH2_HUMAN	CA2
MOL004833	GC23	GAN CAO	2i0j_v	0.9977	ESR1_HUMAN	ESR1
MOL004833	GC23	GAN CAO	1qkm_v	0.9652	ESR2_HUMAN	ESR2
MOL004833	GC23	GAN CAO	1ya8_v	0.9244	EST1_HUMAN	CES1
MOL004833	GC23	GAN CAO	1w8l_v	0.9039	P62937	PPIA
MOL004833	GC23	GAN CAO	1yxx_v	0.9744	PIM1_HUMAN	PIM1
MOL004833	GC23	GAN CAO	2a4z_v	0.9657	PK3CG_HUMAN	PIK3CG
MOL004833	GC23	GAN CAO	1w7h_v	0.9818	Q16539	MAPK14
MOL004833	GC23	GAN CAO	1q8m_v	0.9654	Q9NP99	TREM1
MOL004833	GC23	GAN CAO	1p49_v	0.9568	STS_HUMAN	STS
MOL004835	GC24	GAN CAO	1unh_v	0.918	CD5R1_HUMAN	CDK5R1
MOL004835	GC24	GAN CAO	2i0j_v	0.9192	ESR1_HUMAN	ESR1
MOL004835	GC24	GAN CAO	1x7j_v	0.9318	ESR2_HUMAN	ESR2
MOL004835	GC24	GAN CAO	1ya8_v	0.9512	EST1_HUMAN	CES1
MOL004835	GC24	GAN CAO	1q8m_v	0.9864	Q9NP99	TREM1
MOL004838	GC25	GAN CAO	1e7e_v	0.9538	ALBU_HUMAN	ALB
MOL004838	GC25	GAN CAO	1l6l_v	0.9643	APOA2_HUMAN	APOA2
MOL004838	GC25	GAN CAO	2ohp_v	0.9835	BACE1_HUMAN	BACE1
MOL004838	GC25	GAN CAO	1reu_v	0.908	BMP2_HUMAN	BMP2
MOL004838	GC25	GAN CAO	1if4_v	0.951	CAH2_HUMAN	CA2
MOL004838	GC25	GAN CAO	1gfw_v	0.941	CASP3_HUMAN	CASP3
MOL004838	GC25	GAN CAO	2brg_v	0.9771	CHK1_HUMAN	CHEK1
MOL004838	GC25	GAN CAO	2i0j_v	0.9879	ESR1_HUMAN	ESR1
MOL004838	GC25	GAN CAO	1ya8_v	0.9444	EST1_HUMAN	CES1
MOL004838	GC25	GAN CAO	1pme_v	0.9919	MK01_HUMAN	MAPK1
MOL004838	GC25	GAN CAO	1eub_v	0.9701	MMP13_HUMAN	MMP13
MOL004838	GC25	GAN CAO	2p3g_v	0.9587	P49137	APKAPK2
MOL004838	GC25	GAN CAO	2o65_v	0.9837	PIM1_HUMAN	PIM1
MOL004838	GC25	GAN CAO	2ovm_v	0.9156	PRGR_HUMAN	PGR
MOL004838	GC25	GAN CAO	1p49_v	0.9621	STS_HUMAN	STS
MOL004838	GC25	GAN CAO	1j78_v	0.9043	VTDB_HUMAN	GC
MOL004841	GC26	GAN CAO	2qo8_v	0.9445	CAH2_HUMAN	CA2
MOL004841	GC26	GAN CAO	1lyw_v	0.964	CATD_HUMAN	CTSD
MOL004841	GC26	GAN CAO	1qku_v	0.975	ESR1_HUMAN	ESR1

MOL004841	GC26	GAN CAO	2jj3_v	0.932	ESR2_HUMAN	ESR2
MOL004841	GC26	GAN CAO	1ya8_v	0.9246	EST1_HUMAN	CES1
MOL004841	GC26	GAN CAO	1yxx_v	0.9378	PIM1_HUMAN	PIM1
MOL004841	GC26	GAN CAO	1q8m_v	0.9677	Q9NP99	TREM1
MOL004841	GC26	GAN CAO	1z68_v	0.9222	SEPR_HUMAN	FAP
MOL004848	GC27	GAN CAO	1j96_v	0.9991	AK1C2_HUMAN	AKR1C2
MOL004848	GC27	GAN CAO	1xf0_v	0.9796	AK1C3_HUMAN	AKR1C3
MOL004848	GC27	GAN CAO	2agt_v	0.959	ALDR_HUMAN	AKR1B1
MOL004848	GC27	GAN CAO	2pir_v	0.9642	ANDR_HUMAN	AR
MOL004848	GC27	GAN CAO	3dej_v	0.9723	CASP3_HUMAN	CASP3
MOL004848	GC27	GAN CAO	1xlu_v	0.9654	CHLE_HUMAN	BCHE
MOL004848	GC27	GAN CAO	2vwu_v	0.9699	EPHB4_HUMAN	EPHB4
MOL004848	GC27	GAN CAO	1qku_v	0.9614	ESR1_HUMAN	ESR1
MOL004848	GC27	GAN CAO	1ya8_v	0.9292	EST1_HUMAN	CES1
MOL004848	GC27	GAN CAO	1ogs_v	0.9946	GLCM_HUMAN	GBA
MOL004848	GC27	GAN CAO	1lbk_v	0.9404	GSTP1_HUMAN	GSTP1
MOL004848	GC27	GAN CAO	2hck_v	0.9084	HCK_HUMAN	HCK
MOL004848	GC27	GAN CAO	3dcw_v	0.9833	P00918	CA2
MOL004848	GC27	GAN CAO	2obj_v	0.9554	P11309	PIM1
MOL004848	GC27	GAN CAO	3eqm_v	0.9719	P11511	CYP19A1
MOL004848	GC27	GAN CAO	2ovm_v	0.9228	PRGR_HUMAN	PGR
MOL004848	GC27	GAN CAO	1wbo_v	0.9948	Q16539	MAPK14
MOL004848	GC27	GAN CAO	1q8m_v	0.9496	Q9NP99	TREM1
MOL004849	GC28	GAN CAO	1j96_v	0.9977	AK1C2_HUMAN	AKR1C2
MOL004849	GC28	GAN CAO	1xf0_v	0.9416	AK1C3_HUMAN	AKR1C3
MOL004849	GC28	GAN CAO	1xj7_v	0.9856	ANDR_HUMAN	AR
MOL004849	GC28	GAN CAO	1reu_v	0.9088	BMP2_HUMAN	BMP2
MOL004849	GC28	GAN CAO	1okl_v	0.929	CAH2_HUMAN	CA2
MOL004849	GC28	GAN CAO	1gfw_v	0.9838	CASP3_HUMAN	CASP3
MOL004849	GC28	GAN CAO	1rs0_v	0.988	CFAB_HUMAN	CFB
MOL004849	GC28	GAN CAO	1klt_v	0.9628	CMA1_HUMAN	CMA1
MOL004849	GC28	GAN CAO	1yb1_v	0.9772	DHB11_HUMAN	HSD17B11
MOL004849	GC28	GAN CAO	2vwu_v	0.9603	EPHB4_HUMAN	EPHB4
MOL004849	GC28	GAN CAO	1qku_v	0.9627	ESR1_HUMAN	ESR1
MOL004849	GC28	GAN CAO	2jj3_v	0.9291	ESR2_HUMAN	ESR2
MOL004849	GC28	GAN CAO	1ya8_v	0.9888	EST1_HUMAN	CES1
MOL004849	GC28	GAN CAO	1d7i_v	0.9446	FKB1A_HUMAN	FKBP1A
MOL004849	GC28	GAN CAO	1ogs_v	0.9671	GLCM_HUMAN	GBA
MOL004849	GC28	GAN CAO	1g1q_v	0.9466	LYAM3_HUMAN	SELP
MOL004849	GC28	GAN CAO	1uki_v	0.9943	MK08_HUMAN	MAPK8
MOL004849	GC28	GAN CAO	1pmv_v	0.9986	MK10_HUMAN	MAPK10
MOL004849	GC28	GAN CAO	1bm6_v	0.9681	MMP3_HUMAN	MMP3

MOL004849	GC28	GAN CAO	3bgp_v	1	P11309	PIM1
MOL004849	GC28	GAN CAO	3eqm_v	0.9748	P11511	CYP19A1
MOL004849	GC28	GAN CAO	1w8m_v	0.9282	P62937	PPIA
MOL004849	GC28	GAN CAO	1v3q_v	0.9519	PNPH_HUMAN	PNP
MOL004849	GC28	GAN CAO	1nub_v	0.9482	SPRC_HUMAN	SPARC
MOL004849	GC28	GAN CAO	1no9_v	0.9945	THRB_HUMAN	F2
MOL004855	GC29	GAN CAO	1e7e_v	0.9282	ALBU_HUMAN	ALB
MOL004855	GC29	GAN CAO	2pio_v	0.9823	ANDR_HUMAN	AR
MOL004855	GC29	GAN CAO	1l6l_v	0.9263	APOA2_HUMAN	APOA2
MOL004855	GC29	GAN CAO	1reu_v	0.9168	BMP2_HUMAN	BMP2
MOL004855	GC29	GAN CAO	1if4_v	0.9526	CAH2_HUMAN	CA2
MOL004855	GC29	GAN CAO	1shj_v	0.9641	CASP7_HUMAN	CASP7
MOL004855	GC29	GAN CAO	1rs0_v	0.9034	CFAB_HUMAN	CFB
MOL004855	GC29	GAN CAO	1qku_v	0.9362	ESR1_HUMAN	ESR1
MOL004855	GC29	GAN CAO	1qkm_v	0.9496	ESR2_HUMAN	ESR2
MOL004855	GC29	GAN CAO	1ya8_v	0.9448	EST1_HUMAN	CES1
MOL004855	GC29	GAN CAO	1ogs_v	0.9524	GLCM_HUMAN	GBA
MOL004855	GC29	GAN CAO	1lbk_v	0.9737	GSTP1_HUMAN	GSTP1
MOL004855	GC29	GAN CAO	2obj_v	0.9642	P11309	PIM1
MOL004855	GC29	GAN CAO	2jbp_v	0.984	P49137	[illegible]APKAPK2
MOL004855	GC29	GAN CAO	3exo_v	0.9667	P56817	BACE1
MOL004855	GC29	GAN CAO	1w8l_v	0.9714	P62937	PPIA
MOL004855	GC29	GAN CAO	1v3q_v	0.972	PNPH_HUMAN	PNP
MOL004855	GC29	GAN CAO	1w7h_v	0.9773	Q16539	MAPK14
MOL004855	GC29	GAN CAO	1q8m_v	0.94	Q9NP99	TREM1
MOL004855	GC29	GAN CAO	1p49_v	0.9613	STS_HUMAN	STS
MOL004855	GC29	GAN CAO	1dvz_v	0.9599	TTHY_HUMAN	TTR
MOL000239	GC3	GAN CAO	1xlu_v	0.9691	CHLE_HUMAN	BCHE
MOL000239	GC3	GAN CAO	2pog_v	0.9754	ESR1_HUMAN	ESR1
MOL000239	GC3	GAN CAO	1ogs_v	0.9662	GLCM_HUMAN	GBA
MOL000239	GC3	GAN CAO	1yxx_v	0.9619	PIM1_HUMAN	PIM1
MOL004856	GC30	GAN CAO	1e7e_v	0.9484	ALBU_HUMAN	ALB
MOL004856	GC30	GAN CAO	1x97_v	0.9038	ALDR_HUMAN	AKR1B1
MOL004856	GC30	GAN CAO	2pir_v	0.9779	ANDR_HUMAN	AR
MOL004856	GC30	GAN CAO	1l6l_v	0.9705	APOA2_HUMAN	APOA2
MOL004856	GC30	GAN CAO	2ohp_v	0.928	BACE1_HUMAN	BACE1
MOL004856	GC30	GAN CAO	1if4_v	0.9541	CAH2_HUMAN	CA2
MOL004856	GC30	GAN CAO	1shj_v	0.9812	CASP7_HUMAN	CASP7
MOL004856	GC30	GAN CAO	2bkz_v	0.9228	CCNA2_HUMAN	CCNA2
MOL004856	GC30	GAN CAO	1unh_v	0.9291	CD5R1_HUMAN	CDK5R1
MOL004856	GC30	GAN CAO	1rs0_v	0.9903	CFAB_HUMAN	CFB
MOL004856	GC30	GAN CAO	1p0i_v	0.9269	CHLE_HUMAN	BCHE

MOL004856	GC30	GAN CAO	2vwu_v	0.9512	EPHB4_HUMAN	EPHB4
MOL004856	GC30	GAN CAO	1x7r_v	0.9783	ESR1_HUMAN	ESR1
MOL004856	GC30	GAN CAO	1qkm_v	0.9809	ESR2_HUMAN	ESR2
MOL004856	GC30	GAN CAO	1ya8_v	0.9433	EST1_HUMAN	CES1
MOL004856	GC30	GAN CAO	1ogs_v	0.969	GLCM_HUMAN	GBA
MOL004856	GC30	GAN CAO	2uyi_v	0.9685	KIF11_HUMAN	KIF11
MOL004856	GC30	GAN CAO	1pme_v	0.996	MK01_HUMAN	MAPK1
MOL004856	GC30	GAN CAO	1uki_v	0.9535	MK08_HUMAN	MAPK8
MOL004856	GC30	GAN CAO	3cgf_v	0.9642	MK10_HUMAN	MAPK10
MOL004856	GC30	GAN CAO	1bm6_v	0.9827	MMP3_HUMAN	MMP3
MOL004856	GC30	GAN CAO	3eqm_v	0.9244	P11511	CYP19A1
MOL004856	GC30	GAN CAO	1jvp_v	0.9573	P24941	CDK2
MOL004856	GC30	GAN CAO	3cjf_v	0.9642	P35968	KDR
MOL004856	GC30	GAN CAO	2jbp_v	0.9889	P49137	MAPKAPK2
MOL004856	GC30	GAN CAO	1xoz_v	0.9839	PDE5A_HUMAN	PDE5A
MOL004856	GC30	GAN CAO	1yxx_v	0.9942	PIM1_HUMAN	PIM1
MOL004856	GC30	GAN CAO	1wbo_v	0.9964	Q16539	MAPK14
MOL004856	GC30	GAN CAO	1p49_v	0.9725	STS_HUMAN	STS
MOL004856	GC30	GAN CAO	1no9_v	0.9121	THRB_HUMAN	F2
MOL004856	GC30	GAN CAO	1dvz_v	0.9569	TTHY_HUMAN	TTR
MOL004857	GC31	GAN CAO	1e7e_v	0.942	ALBU_HUMAN	ALB
MOL004857	GC31	GAN CAO	2pir_v	0.9942	ANDR_HUMAN	AR
MOL004857	GC31	GAN CAO	1l6l_v	0.9666	APOA2_HUMAN	APOA2
MOL004857	GC31	GAN CAO	1if4_v	0.9817	CAH2_HUMAN	CA2
MOL004857	GC31	GAN CAO	1shj_v	0.9772	CASP7_HUMAN	CASP7
MOL004857	GC31	GAN CAO	1unh_v	0.9045	CD5R1_HUMAN	CDK5R1
MOL004857	GC31	GAN CAO	1rs0_v	0.9984	CFAB_HUMAN	CFB
MOL004857	GC31	GAN CAO	1p0i_v	0.9366	CHLE_HUMAN	BCHE
MOL004857	GC31	GAN CAO	1vec_v	0.9172	DDX6_HUMAN	DDX6
MOL004857	GC31	GAN CAO	2ocf_v	0.98	ESR1_HUMAN	ESR1
MOL004857	GC31	GAN CAO	1qkm_v	0.9742	ESR2_HUMAN	ESR2
MOL004857	GC31	GAN CAO	1ya8_v	0.9506	EST1_HUMAN	CES1
MOL004857	GC31	GAN CAO	1ogs_v	0.9624	GLCM_HUMAN	GBA
MOL004857	GC31	GAN CAO	2fky_v	0.943	KIF11_HUMAN	KIF11
MOL004857	GC31	GAN CAO	1uki_v	0.9415	MK08_HUMAN	MAPK8
MOL004857	GC31	GAN CAO	3cgf_v	0.9841	MK10_HUMAN	MAPK10
MOL004857	GC31	GAN CAO	1xur_v	0.9128	MMP13_HUMAN	MMP13
MOL004857	GC31	GAN CAO	3cjf_v	0.9842	P35968	KDR
MOL004857	GC31	GAN CAO	2jbp_v	0.9975	P49137	MAPKAPK2
MOL004857	GC31	GAN CAO	1w8l_v	0.9178	P62937	PPIA
MOL004857	GC31	GAN CAO	1yxx_v	0.9843	PIM1_HUMAN	PIM1
MOL004857	GC31	GAN CAO	2ovm_v	0.9365	PRGR_HUMAN	PGR

MOL004857	GC31	GAN CAO	1w7h_v	0.9955	Q16539	MAPK14
MOL004857	GC31	GAN CAO	1z68_v	0.9797	SEPR_HUMAN	FAP
MOL004857	GC31	GAN CAO	1p49_v	0.9709	STS_HUMAN	STS
MOL004857	GC31	GAN CAO	1no9_v	0.9114	THRB_HUMAN	F2
MOL004857	GC31	GAN CAO	1dvz_v	0.9707	TTHY_HUMAN	TTR
MOL004860	GC32	GAN CAO	1k59_v	0.9523	ANGI_HUMAN	ANG
MOL004860	GC32	GAN CAO	1bzm_v	0.9423	CAH1_HUMAN	CA1
MOL004860	GC32	GAN CAO	1jd0_v	0.9403	CAH12_HUMAN	CA12
MOL004860	GC32	GAN CAO	1oq5_v	0.9722	CAH2_HUMAN	CA2
MOL004860	GC32	GAN CAO	1lyw_v	0.935	CATD_HUMAN	CTSD
MOL004860	GC32	GAN CAO	1h1p_v	0.9789	CCNA2_HUMAN	CCNA2
MOL004860	GC32	GAN CAO	2cgw_v	0.9568	CHK1_HUMAN	CHEK1
MOL004860	GC32	GAN CAO	1p0i_v	0.9443	CHLE_HUMAN	BCHE
MOL004860	GC32	GAN CAO	1klt_v	0.92	CMA1_HUMAN	CMA1
MOL004860	GC32	GAN CAO	1vec_v	0.9196	DDX6_HUMAN	DDX6
MOL004860	GC32	GAN CAO	2itx_v	0.9051	EGFR_HUMAN	EGFR
MOL004860	GC32	GAN CAO	2vwu_v	0.9607	EPHB4_HUMAN	EPHB4
MOL004860	GC32	GAN CAO	1qku_v	0.928	ESR1_HUMAN	ESR1
MOL004860	GC32	GAN CAO	1ovz_v	0.9172	FCAR_HUMAN	FCAR
MOL004860	GC32	GAN CAO	1ogs_v	0.9577	GLCM_HUMAN	GBA
MOL004860	GC32	GAN CAO	1md3_v	0.9328	GSTP1_HUMAN	GSTP1
MOL004860	GC32	GAN CAO	2hck_v	0.9889	HCK_HUMAN	HCK
MOL004860	GC32	GAN CAO	1eub_v	0.9087	MMP13_HUMAN	MMP13
MOL004860	GC32	GAN CAO	1smd_v	0.9129	P04745	AMY1A
MOL004860	GC32	GAN CAO	2pin_v	0.9348	P10828	THRB
MOL004860	GC32	GAN CAO	3fzh_v	0.9483	P11142	HSPA8
MOL004860	GC32	GAN CAO	1e1x_v	0.9639	P24941	CDK2
MOL004860	GC32	GAN CAO	1w8m_v	0.954	P62937	PPIA
MOL004860	GC32	GAN CAO	1xn0_v	0.9831	PDE4B_HUMAN	PDE4B
MOL004860	GC32	GAN CAO	1yxx_v	0.9692	PIM1_HUMAN	PIM1
MOL004860	GC32	GAN CAO	1v3q_v	0.9555	PNPH_HUMAN	PNP
MOL004860	GC32	GAN CAO	1cb0_v	0.9018	Q13126	MTAP
MOL004860	GC32	GAN CAO	1q33_v	0.9855	Q9BW91	NUDT9
MOL004860	GC32	GAN CAO	1z68_v	0.9697	SEPR_HUMAN	FAP
MOL004860	GC32	GAN CAO	1m9z_v	0.9441	TGFR2_HUMAN	TGFBR2
MOL004860	GC32	GAN CAO	1dvz_v	0.9779	TTHY_HUMAN	TTR
MOL004860	GC32	GAN CAO	1fv9_v	0.9054	UROK_HUMAN	PLAU
MOL004863	GC33	GAN CAO	1j96_v	0.9853	AK1C2_HUMAN	AKR1C2
MOL004863	GC33	GAN CAO	1e7e_v	0.9137	ALBU_HUMAN	ALB
MOL004863	GC33	GAN CAO	2pir_v	0.9246	ANDR_HUMAN	AR
MOL004863	GC33	GAN CAO	1l6l_v	0.9507	APOA2_HUMAN	APOA2
MOL004863	GC33	GAN CAO	1reu_v	0.9548	BMP2_HUMAN	BMP2

MOL004863	GC33	GAN CAO	2osf_v	0.975	CAH2_HUMAN	CA2
MOL004863	GC33	GAN CAO	1shj_v	0.9631	CASP7_HUMAN	CASP7
MOL004863	GC33	GAN CAO	1lyw_v	0.9005	CATD_HUMAN	CTSD
MOL004863	GC33	GAN CAO	1rs0_v	0.9736	CFAB_HUMAN	CFB
MOL004863	GC33	GAN CAO	2cgx_v	0.9595	CHK1_HUMAN	CHEK1
MOL004863	GC33	GAN CAO	1xlu_v	0.9806	CHLE_HUMAN	BCHE
MOL004863	GC33	GAN CAO	2itx_v	0.9436	EGFR_HUMAN	EGFR
MOL004863	GC33	GAN CAO	2qa8_v	0.9624	ESR1_HUMAN	ESR1
MOL004863	GC33	GAN CAO	1qkm_v	0.9486	ESR2_HUMAN	ESR2
MOL004863	GC33	GAN CAO	1ya8_v	0.9206	EST1_HUMAN	CES1
MOL004863	GC33	GAN CAO	1md3_v	0.9244	GSTP1_HUMAN	GSTP1
MOL004863	GC33	GAN CAO	2fky_v	0.95	KIF11_HUMAN	KIF11
MOL004863	GC33	GAN CAO	2g01_v	0.9478	MK08_HUMAN	MAPK8
MOL004863	GC33	GAN CAO	1eub_v	0.9515	MMP13_HUMAN	MMP13
MOL004863	GC33	GAN CAO	3eqm_v	0.9133	P11511	CYP19A1
MOL004863	GC33	GAN CAO	2jbp_v	0.9826	P49137	MAPKAPK2
MOL004863	GC33	GAN CAO	1w8l_v	0.9399	P62937	PPIA
MOL004863	GC33	GAN CAO	1yxx_v	0.9759	PIM1_HUMAN	PIM1
MOL004863	GC33	GAN CAO	1v3q_v	0.9555	PNPH_HUMAN	PNP
MOL004863	GC33	GAN CAO	1w7h_v	0.9925	Q16539	MAPK14
MOL004863	GC33	GAN CAO	1z68_v	0.9828	SEPR_HUMAN	FAP
MOL004863	GC33	GAN CAO	1p49_v	0.9559	STS_HUMAN	STS
MOL004863	GC33	GAN CAO	1dvz_v	0.9807	TTHY_HUMAN	TTR
MOL004864	GC34	GAN CAO	1j96_v	0.9907	AK1C2_HUMAN	AKR1C2
MOL004864	GC34	GAN CAO	1e7e_v	0.916	ALBU_HUMAN	ALB
MOL004864	GC34	GAN CAO	1xow_v	0.9672	ANDR_HUMAN	AR
MOL004864	GC34	GAN CAO	1l6l_v	0.9514	APOA2_HUMAN	APOA2
MOL004864	GC34	GAN CAO	2ohp_v	0.9265	BACE1_HUMAN	BACE1
MOL004864	GC34	GAN CAO	1reu_v	0.9499	BMP2_HUMAN	BMP2
MOL004864	GC34	GAN CAO	1if4_v	0.9819	CAH2_HUMAN	CA2
MOL004864	GC34	GAN CAO	1shj_v	0.968	CASP7_HUMAN	CASP7
MOL004864	GC34	GAN CAO	1rs0_v	0.9829	CFAB_HUMAN	CFB
MOL004864	GC34	GAN CAO	1xlu_v	0.9811	CHLE_HUMAN	BCHE
MOL004864	GC34	GAN CAO	2vwu_v	0.9128	EPHB4_HUMAN	EPHB4
MOL004864	GC34	GAN CAO	1x7r_v	0.9706	ESR1_HUMAN	ESR1
MOL004864	GC34	GAN CAO	1qkm_v	0.9821	ESR2_HUMAN	ESR2
MOL004864	GC34	GAN CAO	1lbk_v	0.9476	GSTP1_HUMAN	GSTP1
MOL004864	GC34	GAN CAO	2fky_v	0.9186	KIF11_HUMAN	KIF11
MOL004864	GC34	GAN CAO	2g01_v	0.9155	MK08_HUMAN	MAPK8
MOL004864	GC34	GAN CAO	2obj_v	0.9643	P11309	PIM1
MOL004864	GC34	GAN CAO	2jbp_v	0.9885	P49137	MAPKAPK2
MOL004864	GC34	GAN CAO	1w8l_v	0.9126	P62937	PPIA

MOL004864	GC34	GAN CAO	1p49_v	0.953	STS_HUMAN	STS
MOL004864	GC34	GAN CAO	1dvz_v	0.9943	TTHY_HUMAN	TTR
MOL004866	GC35	GAN CAO	1j96_v	0.9932	AK1C2_HUMAN	AKR1C2
MOL004866	GC35	GAN CAO	1e7e_v	0.9341	ALBU_HUMAN	ALB
MOL004866	GC35	GAN CAO	1x97_v	0.9308	ALDR_HUMAN	AKR1B1
MOL004866	GC35	GAN CAO	2ama_v	0.9339	ANDR_HUMAN	AR
MOL004866	GC35	GAN CAO	1oj9_v	0.9407	AOFB_HUMAN	MAOB
MOL004866	GC35	GAN CAO	1l6l_v	0.9636	APOA2_HUMAN	APOA2
MOL004866	GC35	GAN CAO	2ohp_v	0.9037	BACE1_HUMAN	BACE1
MOL004866	GC35	GAN CAO	1reu_v	0.9161	BMP2_HUMAN	BMP2
MOL004866	GC35	GAN CAO	1oq5_v	0.9783	CAH2_HUMAN	CA2
MOL004866	GC35	GAN CAO	1shj_v	0.9746	CASP7_HUMAN	CASP7
MOL004866	GC35	GAN CAO	1lyw_v	0.9237	CATD_HUMAN	CTSD
MOL004866	GC35	GAN CAO	2f1g_v	0.9097	CATS_HUMAN	CTSS
MOL004866	GC35	GAN CAO	2bkz_v	0.9367	CCNA2_HUMAN	CCNA2
MOL004866	GC35	GAN CAO	1xo2_v	0.904	CDK6_HUMAN	CDK6
MOL004866	GC35	GAN CAO	1rs0_v	0.9616	CFAB_HUMAN	CFB
MOL004866	GC35	GAN CAO	2ywp_v	0.9616	CHK1_HUMAN	CHEK1
MOL004866	GC35	GAN CAO	1xlu_v	0.9674	CHLE_HUMAN	BCHE
MOL004866	GC35	GAN CAO	2pog_v	0.9595	ESR1_HUMAN	ESR1
MOL004866	GC35	GAN CAO	1ogs_v	0.9668	GLCM_HUMAN	GBA
MOL004866	GC35	GAN CAO	2hck_v	0.9869	HCK_HUMAN	HCK
MOL004866	GC35	GAN CAO	3cgf_v	0.9709	MK10_HUMAN	MAPK10
MOL004866	GC35	GAN CAO	3cjf_v	0.9709	P35968	KDR
MOL004866	GC35	GAN CAO	1yxx_v	0.9527	PIM1_HUMAN	PIM1
MOL004866	GC35	GAN CAO	1z68_v	0.9237	SEPR_HUMAN	FAP
MOL004866	GC35	GAN CAO	1p49_v	0.9676	STS_HUMAN	STS
MOL004866	GC35	GAN CAO	1no9_v	0.9429	THRB_HUMAN	F2
MOL004866	GC35	GAN CAO	1dvz_v	0.9713	TTHY_HUMAN	TTR
MOL004879	GC36	GAN CAO	1j96_v	0.9664	AK1C2_HUMAN	AKR1C2
MOL004879	GC36	GAN CAO	1e7e_v	0.9512	ALBU_HUMAN	ALB
MOL004879	GC36	GAN CAO	2pio_v	0.9313	ANDR_HUMAN	AR
MOL004879	GC36	GAN CAO	1oj9_v	1	AOFB_HUMAN	MAOB
MOL004879	GC36	GAN CAO	1l6l_v	0.9713	APOA2_HUMAN	APOA2
MOL004879	GC36	GAN CAO	1reu_v	0.9391	BMP2_HUMAN	BMP2
MOL004879	GC36	GAN CAO	3dej_v	0.9833	CASP3_HUMAN	CASP3
MOL004879	GC36	GAN CAO	1shj_v	0.9791	CASP7_HUMAN	CASP7
MOL004879	GC36	GAN CAO	1rs0_v	0.9258	CFAB_HUMAN	CFB
MOL004879	GC36	GAN CAO	1p0i_v	0.9308	CHLE_HUMAN	BCHE
MOL004879	GC36	GAN CAO	1yb1_v	0.962	DHB11_HUMAN	HSD17B11
MOL004879	GC36	GAN CAO	1x7r_v	0.9828	ESR1_HUMAN	ESR1
MOL004879	GC36	GAN CAO	1qkm_v	0.9877	ESR2_HUMAN	ESR2

MOL004879	GC36	GAN CAO	1ya8_v	0.9504	EST1_HUMAN	CES1
MOL004879	GC36	GAN CAO	1ya3_v	0.9498	MCR_HUMAN	NR3C2
MOL004879	GC36	GAN CAO	1pme_v	0.9875	MK01_HUMAN	MAPK1
MOL004879	GC36	GAN CAO	1uki_v	0.9917	MK08_HUMAN	MAPK8
MOL004879	GC36	GAN CAO	1pmv_v	0.9961	MK10_HUMAN	MAPK10
MOL004879	GC36	GAN CAO	1bm6_v	0.9651	MMP3_HUMAN	MMP3
MOL004879	GC36	GAN CAO	3dcw_v	0.9844	P00918	CA2
MOL004879	GC36	GAN CAO	2jbp_v	0.9836	P49137	MAPKAPK2
MOL004879	GC36	GAN CAO	1w8l_v	0.9751	P62937	PPIA
MOL004879	GC36	GAN CAO	1yxx_v	0.9901	PIM1_HUMAN	PIM1
MOL004879	GC36	GAN CAO	2a4z_v	0.9767	PK3CG_HUMAN	PIK3CG
MOL004879	GC36	GAN CAO	1wbw_v	0.9922	Q16539	MAPK14
MOL004879	GC36	GAN CAO	1q8m_v	0.9741	Q9NP99	TREM1
MOL004879	GC36	GAN CAO	1p49_v	0.9744	STS_HUMAN	STS
MOL004879	GC36	GAN CAO	1j78_v	0.9198	VTDB_HUMAN	GC
MOL004882	GC37	GAN CAO	1xf0_v	0.9272	AK1C3_HUMAN	AKR1C3
MOL004882	GC37	GAN CAO	1e7e_v	0.9622	ALBU_HUMAN	ALB
MOL004882	GC37	GAN CAO	1oj9_v	0.9865	AOFB_HUMAN	MAOB
MOL004882	GC37	GAN CAO	1l6l_v	0.9671	APOA2_HUMAN	APOA2
MOL004882	GC37	GAN CAO	2ohp_v	0.9014	BACE1_HUMAN	BACE1
MOL004882	GC37	GAN CAO	1if4_v	0.9819	CAH2_HUMAN	CA2
MOL004882	GC37	GAN CAO	1gfw_v	0.9153	CASP3_HUMAN	CASP3
MOL004882	GC37	GAN CAO	1shj_v	0.9641	CASP7_HUMAN	CASP7
MOL004882	GC37	GAN CAO	1rs0_v	0.9651	CFAB_HUMAN	CFB
MOL004882	GC37	GAN CAO	1yb1_v	0.9087	DHB11_HUMAN	HSD17B11
MOL004882	GC37	GAN CAO	1qku_v	0.9911	ESR1_HUMAN	ESR1
MOL004882	GC37	GAN CAO	2jj3_v	0.9083	ESR2_HUMAN	ESR2
MOL004882	GC37	GAN CAO	1ya8_v	0.9434	EST1_HUMAN	CES1
MOL004882	GC37	GAN CAO	1pme_v	0.96	MK01_HUMAN	MAPK1
MOL004882	GC37	GAN CAO	1uki_v	0.9103	MK08_HUMAN	MAPK8
MOL004882	GC37	GAN CAO	1pmv_v	0.9065	MK10_HUMAN	MAPK10
MOL004882	GC37	GAN CAO	1jvp_v	0.955	P24941	CDK2
MOL004882	GC37	GAN CAO	1w8l_v	0.9802	P62937	PPIA
MOL004882	GC37	GAN CAO	1yxx_v	0.9638	PIM1_HUMAN	PIM1
MOL004882	GC37	GAN CAO	1wbo_v	0.9838	Q16539	MAPK14
MOL004882	GC37	GAN CAO	1q8m_v	0.9147	Q9NP99	TREM1
MOL004882	GC37	GAN CAO	1p49_v	0.9707	STS_HUMAN	STS
MOL004882	GC37	GAN CAO	1no9_v	0.9664	THRB_HUMAN	F2
MOL004883	GC38	GAN CAO	1e7e_v	0.9706	ALBU_HUMAN	ALB
MOL004883	GC38	GAN CAO	1l6l_v	0.9775	APOA2_HUMAN	APOA2
MOL004883	GC38	GAN CAO	2ohp_v	0.9459	BACE1_HUMAN	BACE1
MOL004883	GC38	GAN CAO	1if4_v	0.9878	CAH2_HUMAN	CA2

MOL004883	GC38	GAN CAO	1gfw_v	0.952	CASP3_HUMAN	CASP3
MOL004883	GC38	GAN CAO	1shj_v	0.9673	CASP7_HUMAN	CASP7
MOL004883	GC38	GAN CAO	2f1g_v	0.9136	CATS_HUMAN	CTSS
MOL004883	GC38	GAN CAO	1rs0_v	0.9865	CFAB_HUMAN	CFB
MOL004883	GC38	GAN CAO	1p0i_v	0.9674	CHLE_HUMAN	BCHE
MOL004883	GC38	GAN CAO	1klt_v	0.9801	CMA1_HUMAN	CMA1
MOL004883	GC38	GAN CAO	2vwu_v	0.941	EPHB4_HUMAN	EPHB4
MOL004883	GC38	GAN CAO	1x7r_v	0.9514	ESR1_HUMAN	ESR1
MOL004883	GC38	GAN CAO	1qkm_v	0.987	ESR2_HUMAN	ESR2
MOL004883	GC38	GAN CAO	1ya8_v	0.9545	EST1_HUMAN	CES1
MOL004883	GC38	GAN CAO	1ogs_v	0.9828	GLCM_HUMAN	GBA
MOL004883	GC38	GAN CAO	2oji_v	0.9664	MK01_HUMAN	MAPK1
MOL004883	GC38	GAN CAO	1uki_v	0.9252	MK08_HUMAN	MAPK8
MOL004883	GC38	GAN CAO	3cgf_v	0.9441	MK10_HUMAN	MAPK10
MOL004883	GC38	GAN CAO	3cjf_v	0.9441	P35968	KDR
MOL004883	GC38	GAN CAO	2jbp_v	0.9904	P49137	MAPKAPK2
MOL004883	GC38	GAN CAO	1w8l_v	0.9886	P62937	PPIA
MOL004883	GC38	GAN CAO	1xoz_v	0.902	PDE5A_HUMAN	PDE5A
MOL004883	GC38	GAN CAO	2o65_v	0.9691	PIM1_HUMAN	PIM1
MOL004883	GC38	GAN CAO	1v3q_v	0.9558	PNPH_HUMAN	PNP
MOL004883	GC38	GAN CAO	1wbo_v	0.9899	Q16539	MAPK14
MOL004883	GC38	GAN CAO	1q8m_v	0.9345	Q9NP99	TREM1
MOL004883	GC38	GAN CAO	1p49_v	0.9767	STS_HUMAN	STS
MOL004883	GC38	GAN CAO	1dvz_v	0.9872	TTHY_HUMAN	TTR
MOL004884	GC39	GAN CAO	1j96_v	0.9693	AK1C2_HUMAN	AKR1C2
MOL004884	GC39	GAN CAO	1xow_v	0.9457	ANDR_HUMAN	AR
MOL004884	GC39	GAN CAO	2ohp_v	0.9916	BACE1_HUMAN	BACE1
MOL004884	GC39	GAN CAO	1reu_v	0.9151	BMP2_HUMAN	BMP2
MOL004884	GC39	GAN CAO	2osf_v	0.9731	CAH2_HUMAN	CA2
MOL004884	GC39	GAN CAO	1shj_v	0.96	CASP7_HUMAN	CASP7
MOL004884	GC39	GAN CAO	1xlu_v	0.971	CHLE_HUMAN	BCHE
MOL004884	GC39	GAN CAO	1klt_v	0.9643	CMA1_HUMAN	CMA1
MOL004884	GC39	GAN CAO	1m17_v	0.9132	EGFR_HUMAN	EGFR
MOL004884	GC39	GAN CAO	1qkm_v	0.975	ESR2_HUMAN	ESR2
MOL004884	GC39	GAN CAO	1ogs_v	0.931	GLCM_HUMAN	GBA
MOL004884	GC39	GAN CAO	2obj_v	0.9278	P11309	PIM1
MOL004884	GC39	GAN CAO	2p3g_v	0.9548	P49137	MAPKAPK2
MOL004884	GC39	GAN CAO	1w8l_v	0.9512	P62937	PPIA
MOL004884	GC39	GAN CAO	2a4z_v	0.9534	PK3CG_HUMAN	PIK3CG
MOL004884	GC39	GAN CAO	1w7h_v	0.9705	Q16539	MAPK14
MOL004884	GC39	GAN CAO	1q8m_v	0.9385	Q9NP99	TREM1
MOL004884	GC39	GAN CAO	2roy_v	0.9559	TTHY_HUMAN	TTR

MOL002565	GC4	GAN CAO	1e7e_v	0.9806	ALBU_HUMAN	ALB
MOL002565	GC4	GAN CAO	2pir_v	0.9576	ANDR_HUMAN	AR
MOL002565	GC4	GAN CAO	1l6l_v	0.96	APOA2_HUMAN	APOA2
MOL002565	GC4	GAN CAO	3bmp_v	0.9904	BMP2_HUMAN	BMP2
MOL002565	GC4	GAN CAO	1if4_v	0.9485	CAH2_HUMAN	CA2
MOL002565	GC4	GAN CAO	1qku_v	0.9752	ESR1_HUMAN	ESR1
MOL002565	GC4	GAN CAO	2obj_v	0.9683	P11309	PIM1
MOL002565	GC4	GAN CAO	1w8l_v	0.9047	P62937	PPIA
MOL002565	GC4	GAN CAO	1p49_v	1	STS_HUMAN	STS
MOL004885	GC40	GAN CAO	1j96_v	0.9699	AK1C2_HUMAN	AKR1C2
MOL004885	GC40	GAN CAO	2ohp_v	0.9912	BACE1_HUMAN	BACE1
MOL004885	GC40	GAN CAO	1reu_v	0.9151	BMP2_HUMAN	BMP2
MOL004885	GC40	GAN CAO	2osf_v	0.9722	CAH2_HUMAN	CA2
MOL004885	GC40	GAN CAO	3dej_v	0.9511	CASP3_HUMAN	CASP3
MOL004885	GC40	GAN CAO	1shj_v	0.9794	CASP7_HUMAN	CASP7
MOL004885	GC40	GAN CAO	1rs0_v	0.9296	CFAB_HUMAN	CFB
MOL004885	GC40	GAN CAO	1xlu_v	0.9788	CHLE_HUMAN	BCHE
MOL004885	GC40	GAN CAO	1klt_v	0.9927	CMA1_HUMAN	CMA1
MOL004885	GC40	GAN CAO	2hck_v	0.9694	HCK_HUMAN	HCK
MOL004885	GC40	GAN CAO	1pme_v	0.9422	MK01_HUMAN	MAPK1
MOL004885	GC40	GAN CAO	1uki_v	0.9756	MK08_HUMAN	MAPK8
MOL004885	GC40	GAN CAO	1pmv_v	0.9755	MK10_HUMAN	MAPK10
MOL004885	GC40	GAN CAO	1bm6_v	0.9108	MMP3_HUMAN	MMP3
MOL004885	GC40	GAN CAO	1xan_v	0.9395	P00390	GSR
MOL004885	GC40	GAN CAO	2obj_v	0.9813	P11309	PIM1
MOL004885	GC40	GAN CAO	3cjf_v	0.9424	P35968	KDR
MOL004885	GC40	GAN CAO	2p3g_v	0.9049	P49137	1APKAPK2
MOL004885	GC40	GAN CAO	1w8l_v	0.9562	P62937	PPIA
MOL004885	GC40	GAN CAO	2a4z_v	0.9488	PK3CG_HUMAN	PIK3CG
MOL004891	GC41	GAN CAO	1j96_v	0.9684	AK1C2_HUMAN	AKR1C2
MOL004891	GC41	GAN CAO	1e7e_v	1	ALBU_HUMAN	ALB
MOL004891	GC41	GAN CAO	1l6l_v	0.9847	APOA2_HUMAN	APOA2
MOL004891	GC41	GAN CAO	1reu_v	0.9829	BMP2_HUMAN	BMP2
MOL004891	GC41	GAN CAO	1if4_v	0.9335	CAH2_HUMAN	CA2
MOL004891	GC41	GAN CAO	1shj_v	0.9437	CASP7_HUMAN	CASP7
MOL004891	GC41	GAN CAO	2vwu_v	0.9395	EPHB4_HUMAN	EPHB4
MOL004891	GC41	GAN CAO	1ya8_v	0.968	EST1_HUMAN	CES1
MOL004891	GC41	GAN CAO	2pg2_v	0.9021	KIF11_HUMAN	KIF11
MOL004891	GC41	GAN CAO	2pin_v	0.9937	P10828	THRB
MOL004891	GC41	GAN CAO	2o65_v	0.9279	PIM1_HUMAN	PIM1
MOL004891	GC41	GAN CAO	2a4z_v	0.9238	PK3CG_HUMAN	PIK3CG
MOL004891	GC41	GAN CAO	1p49_v	1	STS_HUMAN	STS

MOL004898	GC42	GAN CAO	1j96_v	0.9921	AK1C2_HUMAN	AKR1C2
MOL004898	GC42	GAN CAO	1xf0_v	0.9532	AK1C3_HUMAN	AKR1C3
MOL004898	GC42	GAN CAO	1e7e_v	0.951	ALBU_HUMAN	ALB
MOL004898	GC42	GAN CAO	1x97_v	0.9326	ALDR_HUMAN	AKR1B1
MOL004898	GC42	GAN CAO	2pir_v	0.9747	ANDR_HUMAN	AR
MOL004898	GC42	GAN CAO	1l6l_v	0.9706	APOA2_HUMAN	APOA2
MOL004898	GC42	GAN CAO	2ohp_v	0.9855	BACE1_HUMAN	BACE1
MOL004898	GC42	GAN CAO	1reu_v	0.9249	BMP2_HUMAN	BMP2
MOL004898	GC42	GAN CAO	1if4_v	0.9818	CAH2_HUMAN	CA2
MOL004898	GC42	GAN CAO	3dej_v	0.9887	CASP3_HUMAN	CASP3
MOL004898	GC42	GAN CAO	1shj_v	0.9806	CASP7_HUMAN	CASP7
MOL004898	GC42	GAN CAO	2f1g_v	0.9675	CATS_HUMAN	CTSS
MOL004898	GC42	GAN CAO	1rs0_v	0.9944	CFAB_HUMAN	CFB
MOL004898	GC42	GAN CAO	2brg_v	0.909	CHK1_HUMAN	CHEK1
MOL004898	GC42	GAN CAO	1xlu_v	0.9633	CHLE_HUMAN	BCHE
MOL004898	GC42	GAN CAO	1tg6_v	0.9352	CLPP_HUMAN	CLPP
MOL004898	GC42	GAN CAO	2itx_v	0.9502	EGFR_HUMAN	EGFR
MOL004898	GC42	GAN CAO	1qku_v	0.9676	ESR1_HUMAN	ESR1
MOL004898	GC42	GAN CAO	2jj3_v	0.9119	ESR2_HUMAN	ESR2
MOL004898	GC42	GAN CAO	1ya8_v	0.9403	EST1_HUMAN	CES1
MOL004898	GC42	GAN CAO	1ogs_v	0.967	GLCM_HUMAN	GBA
MOL004898	GC42	GAN CAO	1lbk_v	0.9423	GSTP1_HUMAN	GSTP1
MOL004898	GC42	GAN CAO	2hck_v	0.9729	HCK_HUMAN	HCK
MOL004898	GC42	GAN CAO	1uki_v	0.9759	MK08_HUMAN	MAPK8
MOL004898	GC42	GAN CAO	3cgf_v	0.9937	MK10_HUMAN	MAPK10
MOL004898	GC42	GAN CAO	1eub_v	0.9953	MMP13_HUMAN	MMP13
MOL004898	GC42	GAN CAO	1bm6_v	0.9553	MMP3_HUMAN	MMP3
MOL004898	GC42	GAN CAO	2obj_v	0.9749	P11309	PIM1
MOL004898	GC42	GAN CAO	3eqm_v	0.9814	P11511	CYP19A1
MOL004898	GC42	GAN CAO	2exm_v	0.9265	P24941	CDK2
MOL004898	GC42	GAN CAO	3cjf_v	0.9937	P35968	KDR
MOL004898	GC42	GAN CAO	2jbp_v	0.9885	P49137	⁄APKAPK2
MOL004898	GC42	GAN CAO	1w8l_v	0.9301	P62937	PPIA
MOL004898	GC42	GAN CAO	1udu_v	0.9599	PDE5A_HUMAN	PDE5A
MOL004898	GC42	GAN CAO	1v3q_v	0.9619	PNPH_HUMAN	PNP
MOL004898	GC42	GAN CAO	1wbo_v	0.9941	Q16539	MAPK14
MOL004898	GC42	GAN CAO	1z68_v	0.9258	SEPR_HUMAN	FAP
MOL004898	GC42	GAN CAO	1p49_v	0.974	STS_HUMAN	STS
MOL004898	GC42	GAN CAO	1no9_v	0.9703	THRB_HUMAN	F2
MOL004898	GC42	GAN CAO	2roy_v	0.957	TTHY_HUMAN	TTR
MOL004903	GC43	GAN CAO	2pir_v	0.925	ANDR_HUMAN	AR
MOL004903	GC43	GAN CAO	1jd0_v	0.9644	CAH12_HUMAN	CA12

MOL004903	GC43	GAN CAO	1lyw_v	0.9287	CATD_HUMAN	CTSD
MOL004903	GC43	GAN CAO	1xlw_v	0.9765	CHLE_HUMAN	BCHE
MOL004903	GC43	GAN CAO	1vec_v	0.9421	DDX6_HUMAN	DDX6
MOL004903	GC43	GAN CAO	1md3_v	0.9462	GSTP1_HUMAN	GSTP1
MOL004903	GC43	GAN CAO	1pmv_v	0.9148	MK10_HUMAN	MAPK10
MOL004903	GC43	GAN CAO	1cb0_v	0.9611	Q13126	MTAP
MOL004903	GC43	GAN CAO	1q33_v	0.9591	Q9BW91	NUDT9
MOL004903	GC43	GAN CAO	1z68_v	0.9706	SEPR_HUMAN	FAP
MOL004903	GC43	GAN CAO	1m9z_v	0.962	TGFR2_HUMAN	TGFBR2
MOL004903	GC43	GAN CAO	1no9_v	0.9721	THRB_HUMAN	F2
MOL004903	GC43	GAN CAO	2roy_v	0.9342	TTHY_HUMAN	TTR
MOL004904	GC44	GAN CAO	1e7a_v	1	ALBU_HUMAN	ALB
MOL004904	GC44	GAN CAO	2pir_v	0.9073	ANDR_HUMAN	AR
MOL004904	GC44	GAN CAO	1oj9_v	0.9893	AOFB_HUMAN	MAOB
MOL004904	GC44	GAN CAO	1reu_v	0.9891	BMP2_HUMAN	BMP2
MOL004904	GC44	GAN CAO	1okl_v	0.9069	CAH2_HUMAN	CA2
MOL004904	GC44	GAN CAO	1shj_v	0.9815	CASP7_HUMAN	CASP7
MOL004904	GC44	GAN CAO	1rs0_v	0.9149	CFAB_HUMAN	CFB
MOL004904	GC44	GAN CAO	1dic_v	0.9336	CFAD_HUMAN	CFD
MOL004904	GC44	GAN CAO	1xlu_v	0.9681	CHLE_HUMAN	BCHE
MOL004904	GC44	GAN CAO	1yb1_v	0.9815	DHB11_HUMAN	HSD17B11
MOL004904	GC44	GAN CAO	1qku_v	0.926	ESR1_HUMAN	ESR1
MOL004904	GC44	GAN CAO	1qkm_v	0.986	ESR2_HUMAN	ESR2
MOL004904	GC44	GAN CAO	1ya8_v	0.9414	EST1_HUMAN	CES1
MOL004904	GC44	GAN CAO	1d7i_v	0.9107	FKB1A_HUMAN	FKBP1A
MOL004904	GC44	GAN CAO	1ogs_v	0.9709	GLCM_HUMAN	GBA
MOL004904	GC44	GAN CAO	1uki_v	0.973	MK08_HUMAN	MAPK8
MOL004904	GC44	GAN CAO	1pmv_v	0.9957	MK10_HUMAN	MAPK10
MOL004904	GC44	GAN CAO	830c_v	0.9376	MMP13_HUMAN	MMP13
MOL004904	GC44	GAN CAO	1bm6_v	0.9628	MMP3_HUMAN	MMP3
MOL004904	GC44	GAN CAO	2p3g_v	0.9046	P49137	MAPKAPK2
MOL004904	GC44	GAN CAO	1yxx_v	0.9749	PIM1_HUMAN	PIM1
MOL004904	GC44	GAN CAO	2a4z_v	0.9125	PK3CG_HUMAN	PIK3CG
MOL004904	GC44	GAN CAO	1zuc_v	0.9019	PRGR_HUMAN	PGR
MOL004904	GC44	GAN CAO	1w7h_v	0.9806	Q16539	MAPK14
MOL004904	GC44	GAN CAO	1q8m_v	0.9356	Q9NP99	TREM1
MOL004904	GC44	GAN CAO	2roy_v	0.9652	TTHY_HUMAN	TTR
MOL004905	GC45	GAN CAO	1j96_v	0.9391	AK1C2_HUMAN	AKR1C2
MOL004905	GC45	GAN CAO	1e7a_v	0.9645	ALBU_HUMAN	ALB
MOL004905	GC45	GAN CAO	2piq_v	0.984	ANDR_HUMAN	AR
MOL004905	GC45	GAN CAO	1oj9_v	0.9518	AOFB_HUMAN	MAOB
MOL004905	GC45	GAN CAO	1bn4_v	0.9982	CAH2_HUMAN	CA2

MOL004905	GC45	GAN CAO	1rs0_v	0.9381	CFAB_HUMAN	CFB
MOL004905	GC45	GAN CAO	1p0i_v	0.9267	CHLE_HUMAN	BCHE
MOL004905	GC45	GAN CAO	2j5f_v	0.9413	EGFR_HUMAN	EGFR
MOL004905	GC45	GAN CAO	1qkt_v	0.9656	ESR1_HUMAN	ESR1
MOL004905	GC45	GAN CAO	1ya8_v	0.97	EST1_HUMAN	CES1
MOL004905	GC45	GAN CAO	1uki_v	0.9883	MK08_HUMAN	MAPK8
MOL004905	GC45	GAN CAO	1pmv_v	0.99	MK10_HUMAN	MAPK10
MOL004905	GC45	GAN CAO	1bm6_v	0.9747	MMP3_HUMAN	MMP3
MOL004905	GC45	GAN CAO	1pq9_v	0.9214	NR1H2_HUMAN	NR1H2
MOL004905	GC45	GAN CAO	3bgp_v	1	P11309	PIM1
MOL004905	GC45	GAN CAO	3eqm_v	0.932	P11511	CYP19A1
MOL004905	GC45	GAN CAO	1w8l_v	0.9553	P62937	PPIA
MOL004905	GC45	GAN CAO	1fm6_v	0.9637	PPARG_HUMAN	PPARG
MOL004905	GC45	GAN CAO	3dy6_v	0.9685	Q03181	PPARD
MOL004905	GC45	GAN CAO	1p49_v	0.9241	STS_HUMAN	STS
MOL004905	GC45	GAN CAO	1no9_v	0.9142	THRB_HUMAN	F2
MOL004905	GC45	GAN CAO	1tz8_v	0.9227	TTHY_HUMAN	TTR
MOL004905	GC45	GAN CAO	1j78_v	0.9683	VTDB_HUMAN	GC
MOL004907	GC46	GAN CAO	2ama_v	0.9552	ANDR_HUMAN	AR
MOL004907	GC46	GAN CAO	1oq5_v	0.9567	CAH2_HUMAN	CA2
MOL004907	GC46	GAN CAO	1p0i_v	0.9322	CHLE_HUMAN	BCHE
MOL004907	GC46	GAN CAO	2i0j_v	0.9827	ESR1_HUMAN	ESR1
MOL004907	GC46	GAN CAO	2jj3_v	0.9676	ESR2_HUMAN	ESR2
MOL004907	GC46	GAN CAO	1ya8_v	0.9453	EST1_HUMAN	CES1
MOL004907	GC46	GAN CAO	1d7i_v	0.9569	FKB1A_HUMAN	FKBP1A
MOL004907	GC46	GAN CAO	1pme_v	0.968	MK01_HUMAN	MAPK1
MOL004907	GC46	GAN CAO	1yxx_v	0.9931	PIM1_HUMAN	PIM1
MOL004907	GC46	GAN CAO	1w7h_v	0.9717	Q16539	MAPK14
MOL004907	GC46	GAN CAO	1q8m_v	0.9172	Q9NP99	TREM1
MOL004908	GC47	GAN CAO	1j96_v	0.9601	AK1C2_HUMAN	AKR1C2
MOL004908	GC47	GAN CAO	1e7e_v	0.9719	ALBU_HUMAN	ALB
MOL004908	GC47	GAN CAO	2pir_v	0.9629	ANDR_HUMAN	AR
MOL004908	GC47	GAN CAO	1l6l_v	0.9738	APOA2_HUMAN	APOA2
MOL004908	GC47	GAN CAO	1reu_v	0.9656	BMP2_HUMAN	BMP2
MOL004908	GC47	GAN CAO	1oq5_v	0.967	CAH2_HUMAN	CA2
MOL004908	GC47	GAN CAO	1gwq_v	0.9417	ESR1_HUMAN	ESR1
MOL004908	GC47	GAN CAO	2jj3_v	0.9842	ESR2_HUMAN	ESR2
MOL004908	GC47	GAN CAO	1ya8_v	0.9659	EST1_HUMAN	CES1
MOL004908	GC47	GAN CAO	1yxx_v	0.9626	PIM1_HUMAN	PIM1
MOL004908	GC47	GAN CAO	2a4z_v	0.9715	PK3CG_HUMAN	PIK3CG
MOL004908	GC47	GAN CAO	1wbw_v	0.9872	Q16539	MAPK14
MOL004908	GC47	GAN CAO	1p49_v	0.9711	STS_HUMAN	STS

MOL004910	GC48	GAN CAO	1j96_v	0.9596	AK1C2_HUMAN	AKR1C2
MOL004910	GC48	GAN CAO	2pio_v	0.9753	ANDR_HUMAN	AR
MOL004910	GC48	GAN CAO	1l6l_v	0.9165	APOA2_HUMAN	APOA2
MOL004910	GC48	GAN CAO	2ohp_v	0.9066	BACE1_HUMAN	BACE1
MOL004910	GC48	GAN CAO	1reu_v	0.9698	BMP2_HUMAN	BMP2
MOL004910	GC48	GAN CAO	2osf_v	0.9755	CAH2_HUMAN	CA2
MOL004910	GC48	GAN CAO	1rs0_v	0.9483	CFAB_HUMAN	CFB
MOL004910	GC48	GAN CAO	2brb_v	0.9991	CHK1_HUMAN	CHEK1
MOL004910	GC48	GAN CAO	1p0i_v	0.9597	CHLE_HUMAN	BCHE
MOL004910	GC48	GAN CAO	2vwu_v	0.9258	EPHB4_HUMAN	EPHB4
MOL004910	GC48	GAN CAO	1qku_v	0.952	ESR1_HUMAN	ESR1
MOL004910	GC48	GAN CAO	1lbk_v	0.9396	GSTP1_HUMAN	GSTP1
MOL004910	GC48	GAN CAO	2hck_v	0.9943	HCK_HUMAN	HCK
MOL004910	GC48	GAN CAO	2pg2_v	0.9485	KIF11_HUMAN	KIF11
MOL004910	GC48	GAN CAO	1pme_v	0.9478	MK01_HUMAN	MAPK1
MOL004910	GC48	GAN CAO	1eub_v	0.9978	MMP13_HUMAN	MMP13
MOL004910	GC48	GAN CAO	2obj_v	0.9568	P11309	PIM1
MOL004910	GC48	GAN CAO	3eqm_v	0.9291	P11511	CYP19A1
MOL004910	GC48	GAN CAO	1w8l_v	0.9218	P62937	PPIA
MOL004910	GC48	GAN CAO	1p49_v	0.9424	STS_HUMAN	STS
MOL004910	GC48	GAN CAO	1dvz_v	0.9754	TTHY_HUMAN	TTR
MOL004911	GC49	GAN CAO	1j96_v	0.9493	AK1C2_HUMAN	AKR1C2
MOL004911	GC49	GAN CAO	1xf0_v	0.9923	AK1C3_HUMAN	AKR1C3
MOL004911	GC49	GAN CAO	1e7e_v	0.9558	ALBU_HUMAN	ALB
MOL004911	GC49	GAN CAO	1x97_v	0.9211	ALDR_HUMAN	AKR1B1
MOL004911	GC49	GAN CAO	1l6l_v	0.9616	APOA2_HUMAN	APOA2
MOL004911	GC49	GAN CAO	2ohp_v	0.9854	BACE1_HUMAN	BACE1
MOL004911	GC49	GAN CAO	1if4_v	0.9477	CAH2_HUMAN	CA2
MOL004911	GC49	GAN CAO	1gfw_v	0.9241	CASP3_HUMAN	CASP3
MOL004911	GC49	GAN CAO	1shj_v	0.9192	CASP7_HUMAN	CASP7
MOL004911	GC49	GAN CAO	2brg_v	0.9656	CHK1_HUMAN	CHEK1
MOL004911	GC49	GAN CAO	2j5f_v	0.9202	EGFR_HUMAN	EGFR
MOL004911	GC49	GAN CAO	2vwu_v	0.9903	EPHB4_HUMAN	EPHB4
MOL004911	GC49	GAN CAO	2i0j_v	0.9898	ESR1_HUMAN	ESR1
MOL004911	GC49	GAN CAO	1qkm_v	0.9878	ESR2_HUMAN	ESR2
MOL004911	GC49	GAN CAO	1ya8_v	0.9538	EST1_HUMAN	CES1
MOL004911	GC49	GAN CAO	2pg2_v	0.9859	KIF11_HUMAN	KIF11
MOL004911	GC49	GAN CAO	1pme_v	0.9983	MK01_HUMAN	MAPK1
MOL004911	GC49	GAN CAO	1eub_v	0.9855	MMP13_HUMAN	MMP13
MOL004911	GC49	GAN CAO	2pin_v	0.9028	P10828	THRB
MOL004911	GC49	GAN CAO	2p3g_v	0.9262	P49137	MAPKAPK2
MOL004911	GC49	GAN CAO	1yxx_v	0.9743	PIM1_HUMAN	PIM1

MOL004911	GC49	GAN CAO	1v3q_v	0.9403	PNPH_HUMAN	PNP
MOL004911	GC49	GAN CAO	1q8m_v	0.9804	Q9NP99	TREM1
MOL004911	GC49	GAN CAO	1p49_v	0.9528	STS_HUMAN	STS
MOL000354	GC5	GAN CAO	1bzm_v	0.911	CAH1_HUMAN	CA1
MOL000354	GC5	GAN CAO	1p0i_v	0.9556	CHLE_HUMAN	BCHE
MOL000354	GC5	GAN CAO	2itx_v	0.9434	EGFR_HUMAN	EGFR
MOL000354	GC5	GAN CAO	2ocf_v	0.9583	ESR1_HUMAN	ESR1
MOL000354	GC5	GAN CAO	1ogs_v	0.9655	GLCM_HUMAN	GBA
MOL000354	GC5	GAN CAO	2hck_v	0.9907	HCK_HUMAN	HCK
MOL000354	GC5	GAN CAO	3fzh_v	0.9424	P11142	HSPA8
MOL000354	GC5	GAN CAO	1zuc_v	0.9093	PRGR_HUMAN	PGR
MOL000354	GC5	GAN CAO	1no9_v	0.935	THRB_HUMAN	F2
MOL000354	GC5	GAN CAO	1dvz_v	0.9195	TTHY_HUMAN	TTR
MOL004912	GC50	GAN CAO	1j96_v	0.9708	AK1C2_HUMAN	AKR1C2
MOL004912	GC50	GAN CAO	1xf0_v	0.9538	AK1C3_HUMAN	AKR1C3
MOL004912	GC50	GAN CAO	1xow_v	0.9433	ANDR_HUMAN	AR
MOL004912	GC50	GAN CAO	2ohp_v	0.9918	BACE1_HUMAN	BACE1
MOL004912	GC50	GAN CAO	1reu_v	0.9146	BMP2_HUMAN	BMP2
MOL004912	GC50	GAN CAO	1if4_v	0.9646	CAH2_HUMAN	CA2
MOL004912	GC50	GAN CAO	1shj_v	0.9623	CASP7_HUMAN	CASP7
MOL004912	GC50	GAN CAO	1gwq_v	0.926	ESR1_HUMAN	ESR1
MOL004912	GC50	GAN CAO	1qkm_v	0.9339	ESR2_HUMAN	ESR2
MOL004912	GC50	GAN CAO	1ya8_v	0.9017	EST1_HUMAN	CES1
MOL004912	GC50	GAN CAO	2p3g_v	0.952	P49137	MAPKAPK2
MOL004912	GC50	GAN CAO	1w8l_v	0.9158	P62937	PPIA
MOL004912	GC50	GAN CAO	2a4z_v	0.9553	PK3CG_HUMAN	PIK3CG
MOL004912	GC50	GAN CAO	1w7h_v	0.9834	Q16539	MAPK14
MOL004913	GC51	GAN CAO	1kwq_v	0.9168	CAH2_HUMAN	CA2
MOL004913	GC51	GAN CAO	1p0i_v	0.9105	CHLE_HUMAN	BCHE
MOL004913	GC51	GAN CAO	1qku_v	0.9201	ESR1_HUMAN	ESR1
MOL004913	GC51	GAN CAO	1ya8_v	0.9486	EST1_HUMAN	CES1
MOL004913	GC51	GAN CAO	1pmv_v	0.9449	MK10_HUMAN	MAPK10
MOL004913	GC51	GAN CAO	1yxx_v	0.944	PIM1_HUMAN	PIM1
MOL004913	GC51	GAN CAO	1w7h_v	0.9551	Q16539	MAPK14
MOL004913	GC51	GAN CAO	1no9_v	0.9672	THRB_HUMAN	F2
MOL004914	GC52	GAN CAO	1e7e_v	0.9073	ALBU_HUMAN	ALB
MOL004914	GC52	GAN CAO	2q7j_v	0.9178	ANDR_HUMAN	AR
MOL004914	GC52	GAN CAO	1l6l_v	0.921	APOA2_HUMAN	APOA2
MOL004914	GC52	GAN CAO	1reu_v	1	BMP2_HUMAN	BMP2
MOL004914	GC52	GAN CAO	1okl_v	0.9867	CAH2_HUMAN	CA2
MOL004914	GC52	GAN CAO	2uzd_v	0.926	CCNA2_HUMAN	CCNA2
MOL004914	GC52	GAN CAO	1p0i_v	0.9583	CHLE_HUMAN	BCHE

MOL004914	GC52	GAN CAO	1tg6_v	0.9133	CLPP_HUMAN	CLPP
MOL004914	GC52	GAN CAO	2ocf_v	0.973	ESR1_HUMAN	ESR1
MOL004914	GC52	GAN CAO	1ya8_v	0.9161	EST1_HUMAN	CES1
MOL004914	GC52	GAN CAO	1md3_v	0.9082	GSTP1_HUMAN	GSTP1
MOL004914	GC52	GAN CAO	1uki_v	0.9285	MK08_HUMAN	MAPK8
MOL004914	GC52	GAN CAO	1pmv_v	0.9462	MK10_HUMAN	MAPK10
MOL004914	GC52	GAN CAO	3eqm_v	0.9545	P11511	CYP19A1
MOL004914	GC52	GAN CAO	2vth_v	0.9464	P24941	CDK2
MOL004914	GC52	GAN CAO	1yxx_v	0.9022	PIM1_HUMAN	PIM1
MOL004914	GC52	GAN CAO	1v3q_v	0.9477	PNPH_HUMAN	PNP
MOL004914	GC52	GAN CAO	1wbw_v	0.9153	Q16539	MAPK14
MOL004914	GC52	GAN CAO	1q8m_v	0.9648	Q9NP99	TREM1
MOL004914	GC52	GAN CAO	1p49_v	0.9268	STS_HUMAN	STS
MOL004914	GC52	GAN CAO	1no9_v	0.9705	THRB_HUMAN	F2
MOL004915	GC53	GAN CAO	1t63_v	0.9621	ANDR_HUMAN	AR
MOL004915	GC53	GAN CAO	1oq5_v	0.9595	CAH2_HUMAN	CA2
MOL004915	GC53	GAN CAO	1gfw_v	0.9123	CASP3_HUMAN	CASP3
MOL004915	GC53	GAN CAO	1rs0_v	0.9197	CFAB_HUMAN	CFB
MOL004915	GC53	GAN CAO	1x7r_v	0.9827	ESR1_HUMAN	ESR1
MOL004915	GC53	GAN CAO	1qkm_v	0.977	ESR2_HUMAN	ESR2
MOL004915	GC53	GAN CAO	1ya8_v	0.9662	EST1_HUMAN	CES1
MOL004915	GC53	GAN CAO	1d7i_v	0.9603	FKB1A_HUMAN	FKBP1A
MOL004915	GC53	GAN CAO	2uyi_v	0.9881	KIF11_HUMAN	KIF11
MOL004915	GC53	GAN CAO	1pme_v	0.9784	MK01_HUMAN	MAPK1
MOL004915	GC53	GAN CAO	1uki_v	0.9556	MK08_HUMAN	MAPK8
MOL004915	GC53	GAN CAO	1pmv_v	0.9636	MK10_HUMAN	MAPK10
MOL004915	GC53	GAN CAO	1pq9_v	0.9824	NR1H2_HUMAN	NR1H2
MOL004915	GC53	GAN CAO	1jvp_v	0.9745	P24941	CDK2
MOL004915	GC53	GAN CAO	2jbp_v	0.9949	P49137	MAPKAPK2
MOL004915	GC53	GAN CAO	1w8m_v	0.9281	P62937	PPIA
MOL004915	GC53	GAN CAO	1xoz_v	0.953	PDE5A_HUMAN	PDE5A
MOL004915	GC53	GAN CAO	1yxx_v	0.987	PIM1_HUMAN	PIM1
MOL004915	GC53	GAN CAO	1v3q_v	0.9204	PNPH_HUMAN	PNP
MOL004915	GC53	GAN CAO	1w7h_v	0.9977	Q16539	MAPK14
MOL004915	GC53	GAN CAO	1q8m_v	0.9435	Q9NP99	TREM1
MOL004915	GC53	GAN CAO	1no9_v	0.9547	THRB_HUMAN	F2
MOL004915	GC53	GAN CAO	1dvz_v	0.9789	TTHY_HUMAN	TTR
MOL004917	GC54	GAN CAO	1bzm_v	0.9607	CAH1_HUMAN	CA1
MOL004917	GC54	GAN CAO	1jd0_v	0.9681	CAH12_HUMAN	CA12
MOL004917	GC54	GAN CAO	1csb_v	0.9345	CATB_HUMAN	CTSB
MOL004917	GC54	GAN CAO	1fh0_v	0.9474	CATL2_HUMAN	CTSV
MOL004917	GC54	GAN CAO	2f1g_v	0.9269	CATS_HUMAN	CTSS

MOL004917	GC54	GAN CAO	1h1p_v	0.9636	CCNA2_HUMAN	CCNA2
MOL004917	GC54	GAN CAO	1xlw_v	0.942	CHLE_HUMAN	BCHE
MOL004917	GC54	GAN CAO	1vec_v	0.914	DDX6_HUMAN	DDX6
MOL004917	GC54	GAN CAO	2itx_v	0.9752	EGFR_HUMAN	EGFR
MOL004917	GC54	GAN CAO	1qkm_v	0.9917	ESR2_HUMAN	ESR2
MOL004917	GC54	GAN CAO	1d7i_v	0.9169	FKB1A_HUMAN	FKBP1A
MOL004917	GC54	GAN CAO	1ogs_v	0.9555	GLCM_HUMAN	GBA
MOL004917	GC54	GAN CAO	1lbk_v	0.9828	GSTP1_HUMAN	GSTP1
MOL004917	GC54	GAN CAO	2hck_v	0.9803	HCK_HUMAN	HCK
MOL004917	GC54	GAN CAO	1mfu_v	0.9169	P04745	AMY1A
MOL004917	GC54	GAN CAO	3fzh_v	0.9307	P11142	HSPA8
MOL004917	GC54	GAN CAO	2jbo_v	0.9772	P49137	MAPKAPK2
MOL004917	GC54	GAN CAO	1xn0_v	0.9352	PDE4B_HUMAN	PDE4B
MOL004917	GC54	GAN CAO	1yxu_v	0.962	PIM1_HUMAN	PIM1
MOL004917	GC54	GAN CAO	1v3q_v	0.9439	PNPH_HUMAN	PNP
MOL004917	GC54	GAN CAO	1cb0_v	0.9613	Q13126	MTAP
MOL004917	GC54	GAN CAO	1q33_v	0.9651	Q9BW91	NUDT9
MOL004917	GC54	GAN CAO	1ozn_v	0.9665	RTN4R_HUMAN	RTN4R
MOL004917	GC54	GAN CAO	1z68_v	0.9715	SEPR_HUMAN	FAP
MOL004917	GC54	GAN CAO	1m9z_v	0.9585	TGFR2_HUMAN	TGFBR2
MOL004924	GC55	GAN CAO	1e7e_v	0.9779	ALBU_HUMAN	ALB
MOL004924	GC55	GAN CAO	2pir_v	0.9502	ANDR_HUMAN	AR
MOL004924	GC55	GAN CAO	1l6l_v	0.9893	APOA2_HUMAN	APOA2
MOL004924	GC55	GAN CAO	1reu_v	0.9768	BMP2_HUMAN	BMP2
MOL004924	GC55	GAN CAO	1jd0_v	0.9192	CAH12_HUMAN	CA12
MOL004924	GC55	GAN CAO	3dej_v	0.9248	CASP3_HUMAN	CASP3
MOL004924	GC55	GAN CAO	1xlw_v	0.9188	CHLE_HUMAN	BCHE
MOL004924	GC55	GAN CAO	1vec_v	0.9279	DDX6_HUMAN	DDX6
MOL004924	GC55	GAN CAO	2i0j_v	0.9332	ESR1_HUMAN	ESR1
MOL004924	GC55	GAN CAO	1ogs_v	0.9576	GLCM_HUMAN	GBA
MOL004924	GC55	GAN CAO	1lbk_v	0.9531	GSTP1_HUMAN	GSTP1
MOL004924	GC55	GAN CAO	3dcw_v	0.9578	P00918	CA2
MOL004924	GC55	GAN CAO	2obj_v	0.949	P11309	PIM1
MOL004924	GC55	GAN CAO	1v3q_v	0.9067	PNPH_HUMAN	PNP
MOL004924	GC55	GAN CAO	1cb0_v	0.9499	Q13126	MTAP
MOL004924	GC55	GAN CAO	1q33_v	0.9561	Q9BW91	NUDT9
MOL004924	GC55	GAN CAO	1p49_v	1	STS_HUMAN	STS
MOL004924	GC55	GAN CAO	1m9z_v	0.958	TGFR2_HUMAN	TGFBR2
MOL004935	GC56	GAN CAO	1j96_v	0.9607	AK1C2_HUMAN	AKR1C2
MOL004935	GC56	GAN CAO	1xf0_v	0.926	AK1C3_HUMAN	AKR1C3
MOL004935	GC56	GAN CAO	1e7e_v	0.962	ALBU_HUMAN	ALB
MOL004935	GC56	GAN CAO	2agt_v	0.9956	ALDR_HUMAN	AKR1B1

MOL004935	GC56	GAN CAO	2pit_v	0.918	ANDR_HUMAN	AR
MOL004935	GC56	GAN CAO	1oj9_v	0.9493	AOFB_HUMAN	MAOB
MOL004935	GC56	GAN CAO	1l6l_v	0.9623	APOA2_HUMAN	APOA2
MOL004935	GC56	GAN CAO	2ohp_v	0.9115	BACE1_HUMAN	BACE1
MOL004935	GC56	GAN CAO	1reu_v	0.9799	BMP2_HUMAN	BMP2
MOL004935	GC56	GAN CAO	1if4_v	0.992	CAH2_HUMAN	CA2
MOL004935	GC56	GAN CAO	1gfw_v	0.9264	CASP3_HUMAN	CASP3
MOL004935	GC56	GAN CAO	1shj_v	0.9766	CASP7_HUMAN	CASP7
MOL004935	GC56	GAN CAO	1lyw_v	0.963	CATD_HUMAN	CTSD
MOL004935	GC56	GAN CAO	2f1g_v	0.9726	CATS_HUMAN	CTSS
MOL004935	GC56	GAN CAO	1rs0_v	0.9696	CFAB_HUMAN	CFB
MOL004935	GC56	GAN CAO	1p0i_v	0.9618	CHLE_HUMAN	BCHE
MOL004935	GC56	GAN CAO	2vwu_v	0.94	EPHB4_HUMAN	EPHB4
MOL004935	GC56	GAN CAO	2ocf_v	0.9751	ESR1_HUMAN	ESR1
MOL004935	GC56	GAN CAO	1ya8_v	0.9905	EST1_HUMAN	CES1
MOL004935	GC56	GAN CAO	1ogs_v	0.9658	GLCM_HUMAN	GBA
MOL004935	GC56	GAN CAO	2hck_v	0.9906	HCK_HUMAN	HCK
MOL004935	GC56	GAN CAO	2uyi_v	0.9621	KIF11_HUMAN	KIF11
MOL004935	GC56	GAN CAO	1g1q_v	0.9396	LYAM3_HUMAN	SELP
MOL004935	GC56	GAN CAO	1pme_v	0.9992	MK01_HUMAN	MAPK1
MOL004935	GC56	GAN CAO	1uki_v	0.9819	MK08_HUMAN	MAPK8
MOL004935	GC56	GAN CAO	3cgf_v	0.9913	MK10_HUMAN	MAPK10
MOL004935	GC56	GAN CAO	1eub_v	0.9023	MMP13_HUMAN	MMP13
MOL004935	GC56	GAN CAO	1bm6_v	0.9782	MMP3_HUMAN	MMP3
MOL004935	GC56	GAN CAO	1pq9_v	0.9292	NR1H2_HUMAN	NR1H2
MOL004935	GC56	GAN CAO	1xan_v	0.9394	P00390	GSR
MOL004935	GC56	GAN CAO	3bgp_v	0.9953	P11309	PIM1
MOL004935	GC56	GAN CAO	3eqm_v	0.9264	P11511	CYP19A1
MOL004935	GC56	GAN CAO	1jvp_v	0.9532	P24941	CDK2
MOL004935	GC56	GAN CAO	3cjf_v	0.9913	P35968	KDR
MOL004935	GC56	GAN CAO	2jbp_v	0.9908	P49137	MAPKAPK2
MOL004935	GC56	GAN CAO	1w8l_v	0.9788	P62937	PPIA
MOL004935	GC56	GAN CAO	1xoz_v	0.9487	PDE5A_HUMAN	PDE5A
MOL004935	GC56	GAN CAO	1zgy_v	0.9549	PPARG_HUMAN	PPARG
MOL004935	GC56	GAN CAO	2ovm_v	0.9592	PRGR_HUMAN	PGR
MOL004935	GC56	GAN CAO	1w7h_v	0.9393	Q16539	MAPK14
MOL004935	GC56	GAN CAO	1nub_v	0.9479	SPRC_HUMAN	SPARC
MOL004935	GC56	GAN CAO	1p49_v	0.9646	STS_HUMAN	STS
MOL004935	GC56	GAN CAO	1no9_v	0.9958	THRB_HUMAN	F2
MOL004935	GC56	GAN CAO	2roy_v	0.9823	TTHY_HUMAN	TTR
MOL004941	GC57	GAN CAO	1uki_v	0.9134	MK08_HUMAN	MAPK8
MOL004941	GC57	GAN CAO	1pmv_v	0.9276	MK10_HUMAN	MAPK10

MOL004941	GC57	GAN CAO	1no9_v	0.9636	THRB_HUMAN	F2
MOL004945	GC58	GAN CAO	1e7e_v	0.9388	ALBU_HUMAN	ALB
MOL004945	GC58	GAN CAO	1xow_v	0.9122	ANDR_HUMAN	AR
MOL004945	GC58	GAN CAO	1l6l_v	0.9149	APOA2_HUMAN	APOA2
MOL004945	GC58	GAN CAO	2ohp_v	0.9869	BACE1_HUMAN	BACE1
MOL004945	GC58	GAN CAO	1if4_v	0.9475	CAH2_HUMAN	CA2
MOL004945	GC58	GAN CAO	1rs0_v	0.9722	CFAB_HUMAN	CFB
MOL004945	GC58	GAN CAO	2br1_v	0.9922	CHK1_HUMAN	CHEK1
MOL004945	GC58	GAN CAO	1qku_v	0.9493	ESR1_HUMAN	ESR1
MOL004945	GC58	GAN CAO	1ya8_v	0.9766	EST1_HUMAN	CES1
MOL004945	GC58	GAN CAO	2pg2_v	0.9706	KIF11_HUMAN	KIF11
MOL004945	GC58	GAN CAO	1pme_v	0.9313	MK01_HUMAN	MAPK1
MOL004945	GC58	GAN CAO	1uki_v	0.9682	MK08_HUMAN	MAPK8
MOL004945	GC58	GAN CAO	1pmv_v	0.9787	MK10_HUMAN	MAPK10
MOL004945	GC58	GAN CAO	1eub_v	0.9816	MMP13_HUMAN	MMP13
MOL004945	GC58	GAN CAO	2obj_v	0.9559	P11309	PIM1
MOL004945	GC58	GAN CAO	2a4z_v	0.9405	PK3CG_HUMAN	PIK3CG
MOL004945	GC58	GAN CAO	1w7h_v	0.9928	Q16539	MAPK14
MOL004945	GC58	GAN CAO	1p49_v	0.9357	STS_HUMAN	STS
MOL004945	GC58	GAN CAO	1f86_v	0.9991	TTHY_HUMAN	TTR
MOL004948	GC59	GAN CAO	1j96_v	0.9485	AK1C2_HUMAN	AKR1C2
MOL004948	GC59	GAN CAO	1e7a_v	1	ALBU_HUMAN	ALB
MOL004948	GC59	GAN CAO	2pio_v	0.9793	ANDR_HUMAN	AR
MOL004948	GC59	GAN CAO	1reu_v	0.9707	BMP2_HUMAN	BMP2
MOL004948	GC59	GAN CAO	1shj_v	1	CASP7_HUMAN	CASP7
MOL004948	GC59	GAN CAO	2vwu_v	0.922	EPHB4_HUMAN	EPHB4
MOL004948	GC59	GAN CAO	1qkm_v	0.9707	ESR2_HUMAN	ESR2
MOL004948	GC59	GAN CAO	1uki_v	0.9492	MK08_HUMAN	MAPK8
MOL004948	GC59	GAN CAO	1pmv_v	0.9856	MK10_HUMAN	MAPK10
MOL004948	GC59	GAN CAO	1bm6_v	0.9025	MMP3_HUMAN	MMP3
MOL004948	GC59	GAN CAO	2o65_v	0.93	PIM1_HUMAN	PIM1
MOL004948	GC59	GAN CAO	1zyj_v	0.9291	Q16539	MAPK14
MOL003656	GC6	GAN CAO	1j96_v	0.9551	AK1C2_HUMAN	AKR1C2
MOL003656	GC6	GAN CAO	2pir_v	0.9709	ANDR_HUMAN	AR
MOL003656	GC6	GAN CAO	1l6l_v	0.9385	APOA2_HUMAN	APOA2
MOL003656	GC6	GAN CAO	2ohp_v	0.9242	BACE1_HUMAN	BACE1
MOL003656	GC6	GAN CAO	1reu_v	0.9723	BMP2_HUMAN	BMP2
MOL003656	GC6	GAN CAO	1if4_v	0.9813	CAH2_HUMAN	CA2
MOL003656	GC6	GAN CAO	1shj_v	0.9411	CASP7_HUMAN	CASP7
MOL003656	GC6	GAN CAO	1rs0_v	0.9203	CFAB_HUMAN	CFB
MOL003656	GC6	GAN CAO	1p0i_v	0.9633	CHLE_HUMAN	BCHE
MOL003656	GC6	GAN CAO	1x7r_v	0.9826	ESR1_HUMAN	ESR1

MOL003656	GC6	GAN CAO	1qkm_v	0.9868	ESR2_HUMAN	ESR2
MOL003656	GC6	GAN CAO	1lbk_v	0.945	GSTP1_HUMAN	GSTP1
MOL003656	GC6	GAN CAO	2fky_v	0.9246	KIF11_HUMAN	KIF11
MOL003656	GC6	GAN CAO	2obj_v	0.9658	P11309	PIM1
MOL003656	GC6	GAN CAO	1w8l_v	0.9253	P62937	PPIA
MOL003656	GC6	GAN CAO	2a4z_v	0.9792	PK3CG_HUMAN	PIK3CG
MOL003656	GC6	GAN CAO	1p49_v	0.9417	STS_HUMAN	STS
MOL003656	GC6	GAN CAO	1dvz_v	0.9857	TTHY_HUMAN	TTR
MOL004949	GC60	GAN CAO	1e7e_v	0.9615	ALBU_HUMAN	ALB
MOL004949	GC60	GAN CAO	1l6l_v	0.9749	APOA2_HUMAN	APOA2
MOL004949	GC60	GAN CAO	2ohp_v	0.9414	BACE1_HUMAN	BACE1
MOL004949	GC60	GAN CAO	1if4_v	0.9792	CAH2_HUMAN	CA2
MOL004949	GC60	GAN CAO	3dej_v	0.9906	CASP3_HUMAN	CASP3
MOL004949	GC60	GAN CAO	1shj_v	0.9826	CASP7_HUMAN	CASP7
MOL004949	GC60	GAN CAO	1fh0_v	0.9267	CATL2_HUMAN	CTSV
MOL004949	GC60	GAN CAO	1vyw_v	0.954	CCNA2_HUMAN	CCNA2
MOL004949	GC60	GAN CAO	1xo2_v	0.9264	CDK6_HUMAN	CDK6
MOL004949	GC60	GAN CAO	1rs0_v	0.9944	CFAB_HUMAN	CFB
MOL004949	GC60	GAN CAO	1xlu_v	0.9694	CHLE_HUMAN	BCHE
MOL004949	GC60	GAN CAO	2itx_v	0.9361	EGFR_HUMAN	EGFR
MOL004949	GC60	GAN CAO	2pog_v	0.9721	ESR1_HUMAN	ESR1
MOL004949	GC60	GAN CAO	1ya8_v	0.9001	EST1_HUMAN	CES1
MOL004949	GC60	GAN CAO	1ogs_v	0.9716	GLCM_HUMAN	GBA
MOL004949	GC60	GAN CAO	2hck_v	0.9928	HCK_HUMAN	HCK
MOL004949	GC60	GAN CAO	1uki_v	0.9533	MK08_HUMAN	MAPK8
MOL004949	GC60	GAN CAO	3cgf_v	0.9673	MK10_HUMAN	MAPK10
MOL004949	GC60	GAN CAO	1eub_v	0.9137	MMP13_HUMAN	MMP13
MOL004949	GC60	GAN CAO	1bm6_v	0.9508	MMP3_HUMAN	MMP3
MOL004949	GC60	GAN CAO	3fzm_v	0.9058	P11142	HSPA8
MOL004949	GC60	GAN CAO	2obj_v	0.9879	P11309	PIM1
MOL004949	GC60	GAN CAO	3cjf_v	0.9674	P35968	KDR
MOL004949	GC60	GAN CAO	2jbp_v	0.9787	P49137	ɅAPKAPK2
MOL004949	GC60	GAN CAO	1w8l_v	0.9544	P62937	PPIA
MOL004949	GC60	GAN CAO	2ovm_v	0.9149	PRGR_HUMAN	PGR
MOL004949	GC60	GAN CAO	1wbo_v	0.9985	Q16539	MAPK14
MOL004949	GC60	GAN CAO	1p49_v	0.9768	STS_HUMAN	STS
MOL004949	GC60	GAN CAO	1no9_v	0.94	THRB_HUMAN	F2
MOL004949	GC60	GAN CAO	1dvz_v	0.956	TTHY_HUMAN	TTR
MOL004957	GC61	GAN CAO	2i0j_v	0.9928	ESR1_HUMAN	ESR1
MOL004957	GC61	GAN CAO	1x7j_v	0.9865	ESR2_HUMAN	ESR2
MOL004957	GC61	GAN CAO	1yxx_v	0.993	PIM1_HUMAN	PIM1
MOL004959	GC62	GAN CAO	1j96_v	1	AK1C2_HUMAN	AKR1C2

MOL004959	GC62	GAN CAO	1e7e_v	0.9635	ALBU_HUMAN	ALB
MOL004959	GC62	GAN CAO	2agt_v	0.9338	ALDR_HUMAN	AKR1B1
MOL004959	GC62	GAN CAO	2pio_v	0.9948	ANDR_HUMAN	AR
MOL004959	GC62	GAN CAO	1l6l_v	0.9107	APOA2_HUMAN	APOA2
MOL004959	GC62	GAN CAO	1reu_v	0.9575	BMP2_HUMAN	BMP2
MOL004959	GC62	GAN CAO	1zfq_v	0.976	CAH2_HUMAN	CA2
MOL004959	GC62	GAN CAO	1shj_v	0.9358	CASP7_HUMAN	CASP7
MOL004959	GC62	GAN CAO	1rs0_v	0.988	CFAB_HUMAN	CFB
MOL004959	GC62	GAN CAO	2brg_v	0.9367	CHK1_HUMAN	CHEK1
MOL004959	GC62	GAN CAO	2ocf_v	0.9241	ESR1_HUMAN	ESR1
MOL004959	GC62	GAN CAO	1ya8_v	0.9059	EST1_HUMAN	CES1
MOL004959	GC62	GAN CAO	1d7i_v	0.9284	FKB1A_HUMAN	FKBP1A
MOL004959	GC62	GAN CAO	2pg2_v	1	KIF11_HUMAN	KIF11
MOL004959	GC62	GAN CAO	1pme_v	0.9705	MK01_HUMAN	MAPK1
MOL004959	GC62	GAN CAO	1uki_v	0.9588	MK08_HUMAN	MAPK8
MOL004959	GC62	GAN CAO	1pmv_v	0.9635	MK10_HUMAN	MAPK10
MOL004959	GC62	GAN CAO	1eub_v	0.9235	MMP13_HUMAN	MMP13
MOL004959	GC62	GAN CAO	1bm6_v	0.9695	MMP3_HUMAN	MMP3
MOL004959	GC62	GAN CAO	1pq9_v	0.9166	NR1H2_HUMAN	NR1H2
MOL004959	GC62	GAN CAO	1le6_v	0.9326	O15496	PLA2G10
MOL004959	GC62	GAN CAO	2pin_v	1	P10828	THRB
MOL004959	GC62	GAN CAO	3gam_v	0.933	P16083	NQO2
MOL004959	GC62	GAN CAO	2p3g_v	1	P49137	APKAPK2
MOL004959	GC62	GAN CAO	3exo_v	0.9636	P56817	BACE1
MOL004959	GC62	GAN CAO	1w8l_v	0.9768	P62937	PPIA
MOL004959	GC62	GAN CAO	2o65_v	0.9934	PIM1_HUMAN	PIM1
MOL004959	GC62	GAN CAO	1v3q_v	0.9015	PNPH_HUMAN	PNP
MOL004959	GC62	GAN CAO	1wbo_v	0.9933	Q16539	MAPK14
MOL004959	GC62	GAN CAO	1p49_v	0.9775	STS_HUMAN	STS
MOL004959	GC62	GAN CAO	1no9_v	0.9594	THRB_HUMAN	F2
MOL004959	GC62	GAN CAO	1dvz_v	0.9512	TTHY_HUMAN	TTR
MOL004959	GC62	GAN CAO	1j78_v	0.967	VTDB_HUMAN	GC
MOL004961	GC63	GAN CAO	2osf_v	0.9711	CAH2_HUMAN	CA2
MOL004961	GC63	GAN CAO	1xo2_v	0.9068	CDK6_HUMAN	CDK6
MOL004961	GC63	GAN CAO	1rs0_v	0.9088	CFAB_HUMAN	CFB
MOL004961	GC63	GAN CAO	1p0i_v	0.9483	CHLE_HUMAN	BCHE
MOL004961	GC63	GAN CAO	2pog_v	0.9598	ESR1_HUMAN	ESR1
MOL004961	GC63	GAN CAO	1ya8_v	0.9014	EST1_HUMAN	CES1
MOL004961	GC63	GAN CAO	1ogs_v	0.9687	GLCM_HUMAN	GBA
MOL004961	GC63	GAN CAO	2hck_v	0.9898	HCK_HUMAN	HCK
MOL004961	GC63	GAN CAO	3fzh_v	0.9475	P11142	HSPA8
MOL004961	GC63	GAN CAO	1w8m_v	0.9021	P62937	PPIA

MOL004961	GC63	GAN CAO	1yxx_v	0.9434	PIM1_HUMAN	PIM1
MOL004961	GC63	GAN CAO	1zuc_v	0.9307	PRGR_HUMAN	PGR
MOL004961	GC63	GAN CAO	1no9_v	0.9421	THRB_HUMAN	F2
MOL004961	GC63	GAN CAO	1dvz_v	0.92	TTHY_HUMAN	TTR
MOL004966	GC64	GAN CAO	1j96_v	0.9606	AK1C2_HUMAN	AKR1C2
MOL004966	GC64	GAN CAO	1e7a_v	0.9167	ALBU_HUMAN	ALB
MOL004966	GC64	GAN CAO	2ama_v	0.9806	ANDR_HUMAN	AR
MOL004966	GC64	GAN CAO	2ohp_v	0.9625	BACE1_HUMAN	BACE1
MOL004966	GC64	GAN CAO	1reu_v	0.9641	BMP2_HUMAN	BMP2
MOL004966	GC64	GAN CAO	1if4_v	0.9876	CAH2_HUMAN	CA2
MOL004966	GC64	GAN CAO	1shj_v	0.9357	CASP7_HUMAN	CASP7
MOL004966	GC64	GAN CAO	1qku_v	0.9152	ESR1_HUMAN	ESR1
MOL004966	GC64	GAN CAO	1ya8_v	0.9558	EST1_HUMAN	CES1
MOL004966	GC64	GAN CAO	1md3_v	0.9498	GSTP1_HUMAN	GSTP1
MOL004966	GC64	GAN CAO	2pin_v	0.9449	P10828	THRB
MOL004966	GC64	GAN CAO	3bgp_v	0.9331	P11309	PIM1
MOL004966	GC64	GAN CAO	2jbo_v	0.9368	P49137	MAPKAPK2
MOL004966	GC64	GAN CAO	2ovm_v	0.9292	PRGR_HUMAN	PGR
MOL004974	GC65	GAN CAO	1j96_v	0.963	AK1C2_HUMAN	AKR1C2
MOL004974	GC65	GAN CAO	1e7e_v	0.9705	ALBU_HUMAN	ALB
MOL004974	GC65	GAN CAO	2q7j_v	0.9192	ANDR_HUMAN	AR
MOL004974	GC65	GAN CAO	2v61_v	0.9031	AOFB_HUMAN	MAOB
MOL004974	GC65	GAN CAO	1l6l_v	0.9707	APOA2_HUMAN	APOA2
MOL004974	GC65	GAN CAO	2ohp_v	0.9314	BACE1_HUMAN	BACE1
MOL004974	GC65	GAN CAO	1reu_v	0.9629	BMP2_HUMAN	BMP2
MOL004974	GC65	GAN CAO	2itx_v	0.9171	EGFR_HUMAN	EGFR
MOL004974	GC65	GAN CAO	1qkm_v	0.9445	ESR2_HUMAN	ESR2
MOL004974	GC65	GAN CAO	1ya8_v	0.9348	EST1_HUMAN	CES1
MOL004974	GC65	GAN CAO	1lbk_v	0.98	GSTP1_HUMAN	GSTP1
MOL004974	GC65	GAN CAO	3dcw_v	0.958	P00918	CA2
MOL004974	GC65	GAN CAO	2p3g_v	0.9082	P49137	MAPKAPK2
MOL004974	GC65	GAN CAO	2o65_v	0.9212	PIM1_HUMAN	PIM1
MOL004974	GC65	GAN CAO	2a4z_v	0.9729	PK3CG_HUMAN	PIK3CG
MOL004974	GC65	GAN CAO	2qd9_v	0.9473	Q16539	MAPK14
MOL004974	GC65	GAN CAO	1q8m_v	0.9339	Q9NP99	TREM1
MOL004974	GC65	GAN CAO	1p49_v	0.9777	STS_HUMAN	STS
MOL004978	GC66	GAN CAO	1j96_v	0.9627	AK1C2_HUMAN	AKR1C2
MOL004978	GC66	GAN CAO	1e7e_v	0.9736	ALBU_HUMAN	ALB
MOL004978	GC66	GAN CAO	2pir_v	0.9354	ANDR_HUMAN	AR
MOL004978	GC66	GAN CAO	1l6l_v	0.9709	APOA2_HUMAN	APOA2
MOL004978	GC66	GAN CAO	2ohp_v	0.9174	BACE1_HUMAN	BACE1
MOL004978	GC66	GAN CAO	1reu_v	0.9628	BMP2_HUMAN	BMP2

MOL004978	GC66	GAN CAO	1gwq_v	0.9076	ESR1_HUMAN	ESR1
MOL004978	GC66	GAN CAO	2jj3_v	0.9835	ESR2_HUMAN	ESR2
MOL004978	GC66	GAN CAO	1ya8_v	0.9492	EST1_HUMAN	CES1
MOL004978	GC66	GAN CAO	3dcw_v	0.971	P00918	CA2
MOL004978	GC66	GAN CAO	1yxx_v	0.9784	PIM1_HUMAN	PIM1
MOL004978	GC66	GAN CAO	2a4z_v	0.9735	PK3CG_HUMAN	PIK3CG
MOL004978	GC66	GAN CAO	1wbw_v	0.99	Q16539	MAPK14
MOL004978	GC66	GAN CAO	1p49_v	0.9676	STS_HUMAN	STS
MOL004980	GC67	GAN CAO	1j96_v	0.9732	AK1C2_HUMAN	AKR1C2
MOL004980	GC67	GAN CAO	1xf0_v	0.9785	AK1C3_HUMAN	AKR1C3
MOL004980	GC67	GAN CAO	1e7e_v	1	ALBU_HUMAN	ALB
MOL004980	GC67	GAN CAO	2pdk_v	0.9857	ALDR_HUMAN	AKR1B1
MOL004980	GC67	GAN CAO	2pir_v	0.9914	ANDR_HUMAN	AR
MOL004980	GC67	GAN CAO	1hak_v	0.9003	ANXA5_HUMAN	ANXA5
MOL004980	GC67	GAN CAO	1l6l_v	0.9978	APOA2_HUMAN	APOA2
MOL004980	GC67	GAN CAO	2ohp_v	0.9738	BACE1_HUMAN	BACE1
MOL004980	GC67	GAN CAO	1reu_v	0.9586	BMP2_HUMAN	BMP2
MOL004980	GC67	GAN CAO	1okl_v	0.9835	CAH2_HUMAN	CA2
MOL004980	GC67	GAN CAO	1gfw_v	0.9505	CASP3_HUMAN	CASP3
MOL004980	GC67	GAN CAO	1shj_v	0.9713	CASP7_HUMAN	CASP7
MOL004980	GC67	GAN CAO	1rs0_v	0.9705	CFAB_HUMAN	CFB
MOL004980	GC67	GAN CAO	2brb_v	0.9886	CHK1_HUMAN	CHEK1
MOL004980	GC67	GAN CAO	1yb1_v	0.9499	DHB11_HUMAN	HSD17B11
MOL004980	GC67	GAN CAO	2vwu_v	0.9601	EPHB4_HUMAN	EPHB4
MOL004980	GC67	GAN CAO	2ocf_v	0.9851	ESR1_HUMAN	ESR1
MOL004980	GC67	GAN CAO	1ya8_v	0.918	EST1_HUMAN	CES1
MOL004980	GC67	GAN CAO	1md3_v	0.9688	GSTP1_HUMAN	GSTP1
MOL004980	GC67	GAN CAO	2pg2_v	0.9612	KIF11_HUMAN	KIF11
MOL004980	GC67	GAN CAO	1pme_v	0.9994	MK01_HUMAN	MAPK1
MOL004980	GC67	GAN CAO	1uki_v	0.9984	MK08_HUMAN	MAPK8
MOL004980	GC67	GAN CAO	1pmv_v	0.9988	MK10_HUMAN	MAPK10
MOL004980	GC67	GAN CAO	1eub_v	1	MMP13_HUMAN	MMP13
MOL004980	GC67	GAN CAO	2pin_v	0.9914	P10828	THRB
MOL004980	GC67	GAN CAO	3bgp_v	1	P11309	PIM1
MOL004980	GC67	GAN CAO	3eqm_v	0.9886	P11511	CYP19A1
MOL004980	GC67	GAN CAO	1fdu_v	0.9436	P14061	HSD17B1
MOL004980	GC67	GAN CAO	2vth_v	0.9814	P24941	CDK2
MOL004980	GC67	GAN CAO	3cjf_v	0.9785	P35968	KDR
MOL004980	GC67	GAN CAO	2p3g_v	0.97	P49137	MAPKAPK2
MOL004980	GC67	GAN CAO	1w8l_v	0.9169	P62937	PPIA
MOL004980	GC67	GAN CAO	1zuc_v	0.9705	PRGR_HUMAN	PGR
MOL004980	GC67	GAN CAO	1wbo_v	0.9967	Q16539	MAPK14

MOL004980	GC67	GAN CAO	1p49_v	0.9974	STS_HUMAN	STS
MOL004980	GC67	GAN CAO	1no9_v	0.9243	THRB_HUMAN	F2
MOL004980	GC67	GAN CAO	1dvz_v	0.956	TTHY_HUMAN	TTR
MOL004985	GC68	GAN CAO	2oi0_v	0.9187	ADA17_HUMAN	ADAM17
MOL004985	GC68	GAN CAO	1j96_v	0.9366	AK1C2_HUMAN	AKR1C2
MOL004985	GC68	GAN CAO	1e7e_v	0.9725	ALBU_HUMAN	ALB
MOL004985	GC68	GAN CAO	2ine_v	0.9723	ALDR_HUMAN	AKR1B1
MOL004985	GC68	GAN CAO	1oj9_v	0.9824	AOFB_HUMAN	MAOB
MOL004985	GC68	GAN CAO	1l6l_v	0.9685	APOA2_HUMAN	APOA2
MOL004985	GC68	GAN CAO	3bmp_v	0.9999	BMP2_HUMAN	BMP2
MOL004985	GC68	GAN CAO	1if4_v	0.9764	CAH2_HUMAN	CA2
MOL004985	GC68	GAN CAO	3dej_v	0.9033	CASP3_HUMAN	CASP3
MOL004985	GC68	GAN CAO	1shj_v	0.9653	CASP7_HUMAN	CASP7
MOL004985	GC68	GAN CAO	1h1p_v	0.965	CCNA2_HUMAN	CCNA2
MOL004985	GC68	GAN CAO	2cgw_v	0.9288	CHK1_HUMAN	CHEK1
MOL004985	GC68	GAN CAO	1p0i_v	0.9152	CHLE_HUMAN	BCHE
MOL004985	GC68	GAN CAO	1klt_v	0.901	CMA1_HUMAN	CMA1
MOL004985	GC68	GAN CAO	1yb1_v	0.9644	DHB11_HUMAN	HSD17B11
MOL004985	GC68	GAN CAO	2vwu_v	0.9053	EPHB4_HUMAN	EPHB4
MOL004985	GC68	GAN CAO	1lbk_v	0.9913	GSTP1_HUMAN	GSTP1
MOL004985	GC68	GAN CAO	2pg2_v	0.9876	KIF11_HUMAN	KIF11
MOL004985	GC68	GAN CAO	1pme_v	0.9983	MK01_HUMAN	MAPK1
MOL004985	GC68	GAN CAO	2gmx_v	0.9912	MK08_HUMAN	MAPK8
MOL004985	GC68	GAN CAO	1eub_v	0.9719	MMP13_HUMAN	MMP13
MOL004985	GC68	GAN CAO	1qqs_v	0.921	NGAL_HUMAN	LCN2
MOL004985	GC68	GAN CAO	1xv9_v	0.9782	NR1I3_HUMAN	NR1I3
MOL004985	GC68	GAN CAO	2pin_v	0.9668	P10828	THRB
MOL004985	GC68	GAN CAO	3bgp_v	0.9792	P11309	PIM1
MOL004985	GC68	GAN CAO	3eqm_v	0.9258	P11511	CYP19A1
MOL004985	GC68	GAN CAO	2exm_v	0.9715	P24941	CDK2
MOL004985	GC68	GAN CAO	2jbp_v	0.9887	P49137	APKAPK2
MOL004985	GC68	GAN CAO	1w8l_v	0.9676	P62937	PPIA
MOL004985	GC68	GAN CAO	1zgy_v	0.9538	PPARG_HUMAN	PPARG
MOL004985	GC68	GAN CAO	2f6v_v	0.99	PTN1_HUMAN	PTPN1
MOL004985	GC68	GAN CAO	1n83_v	0.9159	RORA_HUMAN	RORA
MOL004985	GC68	GAN CAO	1p49_v	0.9703	STS_HUMAN	STS
MOL004985	GC68	GAN CAO	2roy_v	0.9308	TTHY_HUMAN	TTR
MOL004985	GC68	GAN CAO	1j78_v	0.9084	VTDB_HUMAN	GC
MOL004988	GC69	GAN CAO	1j96_v	0.9601	AK1C2_HUMAN	AKR1C2
MOL004988	GC69	GAN CAO	1xf0_v	0.9657	AK1C3_HUMAN	AKR1C3
MOL004988	GC69	GAN CAO	1e7e_v	0.9921	ALBU_HUMAN	ALB
MOL004988	GC69	GAN CAO	2pir_v	0.9721	ANDR_HUMAN	AR

MOL004988	GC69	GAN CAO	1oj9_v	0.9261	AOFB_HUMAN	MAOB
MOL004988	GC69	GAN CAO	1l6l_v	1	APOA2_HUMAN	APOA2
MOL004988	GC69	GAN CAO	2ohp_v	0.9579	BACE1_HUMAN	BACE1
MOL004988	GC69	GAN CAO	1reu_v	0.9817	BMP2_HUMAN	BMP2
MOL004988	GC69	GAN CAO	3ca2_v	0.9661	CAH2_HUMAN	CA2
MOL004988	GC69	GAN CAO	3dej_v	0.9302	CASP3_HUMAN	CASP3
MOL004988	GC69	GAN CAO	1shj_v	0.9585	CASP7_HUMAN	CASP7
MOL004988	GC69	GAN CAO	1rs0_v	0.9413	CFAB_HUMAN	CFB
MOL004988	GC69	GAN CAO	1ya8_v	0.9564	EST1_HUMAN	CES1
MOL004988	GC69	GAN CAO	2fky_v	0.9298	KIF11_HUMAN	KIF11
MOL004988	GC69	GAN CAO	1pme_v	0.989	MK01_HUMAN	MAPK1
MOL004988	GC69	GAN CAO	1o6u_v	0.9135	O76054	SEC14L2
MOL004988	GC69	GAN CAO	3bgp_v	0.9937	P11309	PIM1
MOL004988	GC69	GAN CAO	3eqm_v	0.9061	P11511	CYP19A1
MOL004988	GC69	GAN CAO	1v3q_v	0.9571	PNPH_HUMAN	PNP
MOL004988	GC69	GAN CAO	1wbo_v	0.9945	Q16539	MAPK14
MOL004988	GC69	GAN CAO	1p49_v	1	STS_HUMAN	STS
MOL004988	GC69	GAN CAO	1dvz_v	0.9694	TTHY_HUMAN	TTR
MOL004988	GC69	GAN CAO	1j78_v	0.9171	VTDB_HUMAN	GC
MOL003896	GC7	GAN CAO	2pir_v	0.9146	ANDR_HUMAN	AR
MOL003896	GC7	GAN CAO	1uki_v	0.9176	MK08_HUMAN	MAPK8
MOL003896	GC7	GAN CAO	1pmv_v	0.9311	MK10_HUMAN	MAPK10
MOL003896	GC7	GAN CAO	3bgp_v	0.925	P11309	PIM1
MOL003896	GC7	GAN CAO	1no9_v	0.9819	THRB_HUMAN	F2
MOL004989	GC70	GAN CAO	1e7e_v	0.9381	ALBU_HUMAN	ALB
MOL004989	GC70	GAN CAO	2pit_v	0.9574	ANDR_HUMAN	AR
MOL004989	GC70	GAN CAO	1oj9_v	0.9451	AOFB_HUMAN	MAOB
MOL004989	GC70	GAN CAO	1l6l_v	0.9613	APOA2_HUMAN	APOA2
MOL004989	GC70	GAN CAO	1if4_v	0.9732	CAH2_HUMAN	CA2
MOL004989	GC70	GAN CAO	1shj_v	0.9766	CASP7_HUMAN	CASP7
MOL004989	GC70	GAN CAO	1lyw_v	0.9036	CATD_HUMAN	CTSD
MOL004989	GC70	GAN CAO	1rs0_v	0.9983	CFAB_HUMAN	CFB
MOL004989	GC70	GAN CAO	2ywp_v	0.9547	CHK1_HUMAN	CHEK1
MOL004989	GC70	GAN CAO	1xlu_v	0.9679	CHLE_HUMAN	BCHE
MOL004989	GC70	GAN CAO	2ocf_v	0.9832	ESR1_HUMAN	ESR1
MOL004989	GC70	GAN CAO	1ya8_v	0.9466	EST1_HUMAN	CES1
MOL004989	GC70	GAN CAO	2hck_v	0.9849	HCK_HUMAN	HCK
MOL004989	GC70	GAN CAO	1uki_v	0.9657	MK08_HUMAN	MAPK8
MOL004989	GC70	GAN CAO	3cgf_v	0.9602	MK10_HUMAN	MAPK10
MOL004989	GC70	GAN CAO	2obj_v	0.9571	P11309	PIM1
MOL004989	GC70	GAN CAO	3eqm_v	0.9175	P11511	CYP19A1
MOL004989	GC70	GAN CAO	3cjf_v	0.9602	P35968	KDR

MOL004989	GC70	GAN CAO	1w8l_v	0.9186	P62937	PPIA
MOL004989	GC70	GAN CAO	1z68_v	0.9295	SEPR_HUMAN	FAP
MOL004989	GC70	GAN CAO	1p49_v	0.9688	STS_HUMAN	STS
MOL004989	GC70	GAN CAO	1no9_v	0.9521	THRB_HUMAN	F2
MOL004989	GC70	GAN CAO	1dvz_v	0.9831	TTHY_HUMAN	TTR
MOL004990	GC71	GAN CAO	1x97_v	0.9525	ALDR_HUMAN	AKR1B1
MOL004990	GC71	GAN CAO	2q7j_v	0.9865	ANDR_HUMAN	AR
MOL004990	GC71	GAN CAO	1unh_v	0.9043	CD5R1_HUMAN	CDK5R1
MOL004990	GC71	GAN CAO	1rs0_v	0.9725	CFAB_HUMAN	CFB
MOL004990	GC71	GAN CAO	2j5f_v	0.9069	EGFR_HUMAN	EGFR
MOL004990	GC71	GAN CAO	2vwu_v	0.9293	EPHB4_HUMAN	EPHB4
MOL004990	GC71	GAN CAO	2i0j_v	0.9826	ESR1_HUMAN	ESR1
MOL004990	GC71	GAN CAO	1x7j_v	0.9867	ESR2_HUMAN	ESR2
MOL004990	GC71	GAN CAO	1ya8_v	0.993	EST1_HUMAN	CES1
MOL004990	GC71	GAN CAO	1lbk_v	0.9326	GSTP1_HUMAN	GSTP1
MOL004990	GC71	GAN CAO	1g1q_v	0.9485	LYAM3_HUMAN	SELP
MOL004990	GC71	GAN CAO	1uki_v	0.989	MK08_HUMAN	MAPK8
MOL004990	GC71	GAN CAO	1pmv_v	0.9974	MK10_HUMAN	MAPK10
MOL004990	GC71	GAN CAO	1bm6_v	0.9922	MMP3_HUMAN	MMP3
MOL004990	GC71	GAN CAO	1pq9_v	0.9609	NR1H2_HUMAN	NR1H2
MOL004990	GC71	GAN CAO	3eqm_v	0.9585	P11511	CYP19A1
MOL004990	GC71	GAN CAO	1w8m_v	0.9234	P62937	PPIA
MOL004990	GC71	GAN CAO	1xoz_v	0.9074	PDE5A_HUMAN	PDE5A
MOL004990	GC71	GAN CAO	1yxx_v	0.9775	PIM1_HUMAN	PIM1
MOL004990	GC71	GAN CAO	1w7h_v	0.9004	Q16539	MAPK14
MOL004990	GC71	GAN CAO	1q8m_v	0.9868	Q9NP99	TREM1
MOL004990	GC71	GAN CAO	1nub_v	0.9471	SPRC_HUMAN	SPARC
MOL004990	GC71	GAN CAO	1no9_v	0.9921	THRB_HUMAN	F2
MOL004991	GC72	GAN CAO	2qo8_v	0.9868	CAH2_HUMAN	CA2
MOL004991	GC72	GAN CAO	1vec_v	0.97	DDX6_HUMAN	DDX6
MOL004991	GC72	GAN CAO	1uki_v	0.905	MK08_HUMAN	MAPK8
MOL004991	GC72	GAN CAO	1pmv_v	0.9209	MK10_HUMAN	MAPK10
MOL004991	GC72	GAN CAO	2ovm_v	0.9189	PRGR_HUMAN	PGR
MOL004991	GC72	GAN CAO	1no9_v	0.9895	THRB_HUMAN	F2
MOL004993	GC73	GAN CAO	1j96_v	0.9832	AK1C2_HUMAN	AKR1C2
MOL004993	GC73	GAN CAO	1xj7_v	0.9592	ANDR_HUMAN	AR
MOL004993	GC73	GAN CAO	1l6l_v	0.9379	APOA2_HUMAN	APOA2
MOL004993	GC73	GAN CAO	2ohp_v	0.9763	BACE1_HUMAN	BACE1
MOL004993	GC73	GAN CAO	1reu_v	0.9632	BMP2_HUMAN	BMP2
MOL004993	GC73	GAN CAO	2osf_v	0.9712	CAH2_HUMAN	CA2
MOL004993	GC73	GAN CAO	1shj_v	0.9462	CASP7_HUMAN	CASP7
MOL004993	GC73	GAN CAO	1xo2_v	0.9174	CDK6_HUMAN	CDK6

MOL004993	GC73	GAN CAO	1rs0_v	0.9655	CFAB_HUMAN	CFB
MOL004993	GC73	GAN CAO	2br1_v	0.9868	CHK1_HUMAN	CHEK1
MOL004993	GC73	GAN CAO	1xlu_v	0.9643	CHLE_HUMAN	BCHE
MOL004993	GC73	GAN CAO	2vwu_v	0.9397	EPHB4_HUMAN	EPHB4
MOL004993	GC73	GAN CAO	2ocf_v	0.9542	ESR1_HUMAN	ESR1
MOL004993	GC73	GAN CAO	1f40_v	0.9508	FKB1A_HUMAN	FKBP1A
MOL004993	GC73	GAN CAO	1md3_v	0.9759	GSTP1_HUMAN	GSTP1
MOL004993	GC73	GAN CAO	2hck_v	0.9812	HCK_HUMAN	HCK
MOL004993	GC73	GAN CAO	2byi_v	0.9149	HS90A_HUMAN	HSP90AA1
MOL004993	GC73	GAN CAO	2pg2_v	0.9864	KIF11_HUMAN	KIF11
MOL004993	GC73	GAN CAO	3cgf_v	0.911	MK10_HUMAN	MAPK10
MOL004993	GC73	GAN CAO	1eub_v	0.9556	MMP13_HUMAN	MMP13
MOL004993	GC73	GAN CAO	2obj_v	0.9602	P11309	PIM1
MOL004993	GC73	GAN CAO	3eqm_v	0.9411	P11511	CYP19A1
MOL004993	GC73	GAN CAO	1e1x_v	0.942	P24941	CDK2
MOL004993	GC73	GAN CAO	3cjf_v	0.9111	P35968	KDR
MOL004993	GC73	GAN CAO	2p3g_v	0.9224	P49137	ɅAPKAPK2
MOL004993	GC73	GAN CAO	1w8l_v	0.9112	P62937	PPIA
MOL004993	GC73	GAN CAO	1zuc_v	0.9484	PRGR_HUMAN	PGR
MOL004993	GC73	GAN CAO	2j7x_v	0.9235	Q62986	Esr2
MOL004993	GC73	GAN CAO	1z68_v	0.9159	SEPR_HUMAN	FAP
MOL004993	GC73	GAN CAO	1no9_v	0.9589	THRB_HUMAN	F2
MOL004993	GC73	GAN CAO	1dvz_v	0.9492	TTHY_HUMAN	TTR
MOL004996	GC74	GAN CAO	2oi0_v	0.9261	ADA17_HUMAN	ADAM17
MOL004996	GC74	GAN CAO	1j96_v	0.939	AK1C2_HUMAN	AKR1C2
MOL004996	GC74	GAN CAO	1e7e_v	0.9722	ALBU_HUMAN	ALB
MOL004996	GC74	GAN CAO	2ine_v	0.9676	ALDR_HUMAN	AKR1B1
MOL004996	GC74	GAN CAO	1oj9_v	0.9828	AOFB_HUMAN	MAOB
MOL004996	GC74	GAN CAO	1l6l_v	0.9676	APOA2_HUMAN	APOA2
MOL004996	GC74	GAN CAO	1reu_v	0.9999	BMP2_HUMAN	BMP2
MOL004996	GC74	GAN CAO	1if4_v	0.988	CAH2_HUMAN	CA2
MOL004996	GC74	GAN CAO	1gfw_v	0.9408	CASP3_HUMAN	CASP3
MOL004996	GC74	GAN CAO	1shj_v	0.9755	CASP7_HUMAN	CASP7
MOL004996	GC74	GAN CAO	1h1p_v	0.9882	CCNA2_HUMAN	CCNA2
MOL004996	GC74	GAN CAO	1rs0_v	0.9643	CFAB_HUMAN	CFB
MOL004996	GC74	GAN CAO	2brg_v	0.9769	CHK1_HUMAN	CHEK1
MOL004996	GC74	GAN CAO	1p0i_v	0.9355	CHLE_HUMAN	BCHE
MOL004996	GC74	GAN CAO	1yb1_v	0.9798	DHB11_HUMAN	HSD17B11
MOL004996	GC74	GAN CAO	2vwu_v	0.9605	EPHB4_HUMAN	EPHB4
MOL004996	GC74	GAN CAO	2j38_v	0.9114	FA10_HUMAN	F10
MOL004996	GC74	GAN CAO	1lbk_v	0.9601	GSTP1_HUMAN	GSTP1
MOL004996	GC74	GAN CAO	2pg2_v	0.9873	KIF11_HUMAN	KIF11

MOL004996	GC74	GAN CAO	1pme_v	0.9974	MK01_HUMAN	MAPK1
MOL004996	GC74	GAN CAO	1pmv_v	0.9118	MK10_HUMAN	MAPK10
MOL004996	GC74	GAN CAO	1eub_v	0.9761	MMP13_HUMAN	MMP13
MOL004996	GC74	GAN CAO	1qqs_v	0.9651	NGAL_HUMAN	LCN2
MOL004996	GC74	GAN CAO	1xv9_v	0.9534	NR1I3_HUMAN	NR1I3
MOL004996	GC74	GAN CAO	2pin_v	0.9836	P10828	THRB
MOL004996	GC74	GAN CAO	3bgp_v	0.9995	P11309	PIM1
MOL004996	GC74	GAN CAO	1e1x_v	0.9824	P24941	CDK2
MOL004996	GC74	GAN CAO	2jbp_v	0.984	P49137	MAPKAPK2
MOL004996	GC74	GAN CAO	1oni_v	0.9489	P52758	RIDA
MOL004996	GC74	GAN CAO	1w8l_v	0.9581	P62937	PPIA
MOL004996	GC74	GAN CAO	1zgy_v	0.977	PPARG_HUMAN	PPARG
MOL004996	GC74	GAN CAO	1hd2_v	0.9619	PRDX5_HUMAN	PRDX5
MOL004996	GC74	GAN CAO	2f6t_v	0.9947	PTN1_HUMAN	PTPN1
MOL004996	GC74	GAN CAO	1bl6_v	0.9185	Q16539	MAPK14
MOL004996	GC74	GAN CAO	1p49_v	0.971	STS_HUMAN	STS
MOL004996	GC74	GAN CAO	2roy_v	0.9884	TTHY_HUMAN	TTR
MOL004996	GC74	GAN CAO	1j78_v	0.9505	VTDB_HUMAN	GC
MOL000500	GC75	GAN CAO	2agt_v	0.9788	ALDR_HUMAN	AKR1B1
MOL000500	GC75	GAN CAO	2pir_v	0.9686	ANDR_HUMAN	AR
MOL000500	GC75	GAN CAO	1l6l_v	0.9516	APOA2_HUMAN	APOA2
MOL000500	GC75	GAN CAO	1shj_v	0.9452	CASP7_HUMAN	CASP7
MOL000500	GC75	GAN CAO	1rs0_v	0.903	CFAB_HUMAN	CFB
MOL000500	GC75	GAN CAO	2vwu_v	0.9018	EPHB4_HUMAN	EPHB4
MOL000500	GC75	GAN CAO	2i0j_v	0.9909	ESR1_HUMAN	ESR1
MOL000500	GC75	GAN CAO	1qkm_v	0.9427	ESR2_HUMAN	ESR2
MOL000500	GC75	GAN CAO	1ya8_v	0.9417	EST1_HUMAN	CES1
MOL000500	GC75	GAN CAO	1bm6_v	0.9009	MMP3_HUMAN	MMP3
MOL000500	GC75	GAN CAO	3dcw_v	0.9343	P00918	CA2
MOL000500	GC75	GAN CAO	3eqm_v	0.9964	P11511	CYP19A1
MOL000500	GC75	GAN CAO	2p3g_v	0.9704	P49137	MAPKAPK2
MOL000500	GC75	GAN CAO	1yxx_v	0.9576	PIM1_HUMAN	PIM1
MOL000500	GC75	GAN CAO	1p49_v	0.9553	STS_HUMAN	STS
MOL005000	GC76	GAN CAO	1e7e_v	0.9349	ALBU_HUMAN	ALB
MOL005000	GC76	GAN CAO	1x97_v	0.9409	ALDR_HUMAN	AKR1B1
MOL005000	GC76	GAN CAO	1l6l_v	0.9485	APOA2_HUMAN	APOA2
MOL005000	GC76	GAN CAO	2ohp_v	0.9544	BACE1_HUMAN	BACE1
MOL005000	GC76	GAN CAO	1czm_v	0.9644	CAH1_HUMAN	CA1
MOL005000	GC76	GAN CAO	1if4_v	0.9466	CAH2_HUMAN	CA2
MOL005000	GC76	GAN CAO	1shj_v	0.981	CASP7_HUMAN	CASP7
MOL005000	GC76	GAN CAO	2f1g_v	0.9049	CATS_HUMAN	CTSS
MOL005000	GC76	GAN CAO	1rs0_v	0.9833	CFAB_HUMAN	CFB

MOL005000	GC76	GAN CAO	1xlu_v	0.9622	CHLE_HUMAN	BCHE
MOL005000	GC76	GAN CAO	1x7r_v	0.9643	ESR1_HUMAN	ESR1
MOL005000	GC76	GAN CAO	1qkm_v	0.9916	ESR2_HUMAN	ESR2
MOL005000	GC76	GAN CAO	1ya8_v	0.931	EST1_HUMAN	CES1
MOL005000	GC76	GAN CAO	3chs_v	0.9553	LKHA4_HUMAN	LTA4H
MOL005000	GC76	GAN CAO	1pme_v	0.9733	MK01_HUMAN	MAPK1
MOL005000	GC76	GAN CAO	1uki_v	0.9483	MK08_HUMAN	MAPK8
MOL005000	GC76	GAN CAO	1pmv_v	0.973	MK10_HUMAN	MAPK10
MOL005000	GC76	GAN CAO	1bm6_v	0.9671	MMP3_HUMAN	MMP3
MOL005000	GC76	GAN CAO	3eqm_v	0.9147	P11511	CYP19A1
MOL005000	GC76	GAN CAO	1jvp_v	0.9601	P24941	CDK2
MOL005000	GC76	GAN CAO	3cjf_v	0.9446	P35968	KDR
MOL005000	GC76	GAN CAO	1w8l_v	0.9179	P62937	PPIA
MOL005000	GC76	GAN CAO	1mkd_v	0.9101	PDE4D_HUMAN	PDE4D
MOL005000	GC76	GAN CAO	1xoz_v	0.9502	PDE5A_HUMAN	PDE5A
MOL005000	GC76	GAN CAO	1yxx_v	0.9916	PIM1_HUMAN	PIM1
MOL005000	GC76	GAN CAO	1wbo_v	0.9849	Q16539	MAPK14
MOL005000	GC76	GAN CAO	1p49_v	0.9691	STS_HUMAN	STS
MOL005000	GC76	GAN CAO	1no9_v	0.904	THRB_HUMAN	F2
MOL005000	GC76	GAN CAO	1dvz_v	0.987	TTHY_HUMAN	TTR
MOL005001	GC77	GAN CAO	1j96_v	0.9957	AK1C2_HUMAN	AKR1C2
MOL005001	GC77	GAN CAO	1e7e_v	0.9388	ALBU_HUMAN	ALB
MOL005001	GC77	GAN CAO	1x97_v	0.946	ALDR_HUMAN	AKR1B1
MOL005001	GC77	GAN CAO	1oj9_v	0.9495	AOFB_HUMAN	MAOB
MOL005001	GC77	GAN CAO	1l6l_v	0.9646	APOA2_HUMAN	APOA2
MOL005001	GC77	GAN CAO	2ohp_v	0.9676	BACE1_HUMAN	BACE1
MOL005001	GC77	GAN CAO	1reu_v	0.9561	BMP2_HUMAN	BMP2
MOL005001	GC77	GAN CAO	1czm_v	0.9608	CAH1_HUMAN	CA1
MOL005001	GC77	GAN CAO	1if4_v	0.9928	CAH2_HUMAN	CA2
MOL005001	GC77	GAN CAO	1shj_v	0.9787	CASP7_HUMAN	CASP7
MOL005001	GC77	GAN CAO	1unh_v	0.9412	CD5R1_HUMAN	CDK5R1
MOL005001	GC77	GAN CAO	1rs0_v	0.946	CFAB_HUMAN	CFB
MOL005001	GC77	GAN CAO	1xlu_v	0.9618	CHLE_HUMAN	BCHE
MOL005001	GC77	GAN CAO	2vwu_v	0.946	EPHB4_HUMAN	EPHB4
MOL005001	GC77	GAN CAO	2ocf_v	0.9756	ESR1_HUMAN	ESR1
MOL005001	GC77	GAN CAO	1qkm_v	0.9864	ESR2_HUMAN	ESR2
MOL005001	GC77	GAN CAO	1ya8_v	0.951	EST1_HUMAN	CES1
MOL005001	GC77	GAN CAO	1ogs_v	0.9198	GLCM_HUMAN	GBA
MOL005001	GC77	GAN CAO	2fky_v	0.9371	KIF11_HUMAN	KIF11
MOL005001	GC77	GAN CAO	3chs_v	0.9597	LKHA4_HUMAN	LTA4H
MOL005001	GC77	GAN CAO	1pme_v	0.9926	MK01_HUMAN	MAPK1
MOL005001	GC77	GAN CAO	1uki_v	0.9399	MK08_HUMAN	MAPK8

MOL005001	GC77	GAN CAO	3cgf_v	0.9511	MK10_HUMAN	MAPK10
MOL005001	GC77	GAN CAO	830c_v	0.9453	MMP13_HUMAN	MMP13
MOL005001	GC77	GAN CAO	1bm6_v	0.9706	MMP3_HUMAN	MMP3
MOL005001	GC77	GAN CAO	3eqm_v	0.9071	P11511	CYP19A1
MOL005001	GC77	GAN CAO	1jvp_v	0.9701	P24941	CDK2
MOL005001	GC77	GAN CAO	3cjf_v	0.9511	P35968	KDR
MOL005001	GC77	GAN CAO	2jbp_v	0.9677	P49137	MAPKAPK2
MOL005001	GC77	GAN CAO	1udu_v	0.9586	PDE5A_HUMAN	PDE5A
MOL005001	GC77	GAN CAO	1yxx_v	0.9728	PIM1_HUMAN	PIM1
MOL005001	GC77	GAN CAO	2a4z_v	0.9099	PK3CG_HUMAN	PIK3CG
MOL005001	GC77	GAN CAO	1v3q_v	0.9546	PNPH_HUMAN	PNP
MOL005001	GC77	GAN CAO	1wbo_v	0.9954	Q16539	MAPK14
MOL005001	GC77	GAN CAO	1z68_v	0.984	SEPR_HUMAN	FAP
MOL005001	GC77	GAN CAO	1p49_v	0.9681	STS_HUMAN	STS
MOL005001	GC77	GAN CAO	1no9_v	0.9653	THRB_HUMAN	F2
MOL005001	GC77	GAN CAO	1dvz_v	0.9788	TTHY_HUMAN	TTR
MOL005003	GC78	GAN CAO	1e7e_v	0.9684	ALBU_HUMAN	ALB
MOL005003	GC78	GAN CAO	2pir_v	0.9434	ANDR_HUMAN	AR
MOL005003	GC78	GAN CAO	1oj9_v	0.9707	AOFB_HUMAN	MAOB
MOL005003	GC78	GAN CAO	1l6l_v	0.9254	APOA2_HUMAN	APOA2
MOL005003	GC78	GAN CAO	1reu_v	0.9681	BMP2_HUMAN	BMP2
MOL005003	GC78	GAN CAO	1if4_v	0.9801	CAH2_HUMAN	CA2
MOL005003	GC78	GAN CAO	1shj_v	0.9277	CASP7_HUMAN	CASP7
MOL005003	GC78	GAN CAO	1rs0_v	0.9547	CFAB_HUMAN	CFB
MOL005003	GC78	GAN CAO	2br1_v	0.9563	CHK1_HUMAN	CHEK1
MOL005003	GC78	GAN CAO	2ocf_v	0.9177	ESR1_HUMAN	ESR1
MOL005003	GC78	GAN CAO	1ya8_v	0.9336	EST1_HUMAN	CES1
MOL005003	GC78	GAN CAO	2pg2_v	0.9837	KIF11_HUMAN	KIF11
MOL005003	GC78	GAN CAO	1pme_v	0.9798	MK01_HUMAN	MAPK1
MOL005003	GC78	GAN CAO	1uki_v	0.9796	MK08_HUMAN	MAPK8
MOL005003	GC78	GAN CAO	1pmv_v	0.9563	MK10_HUMAN	MAPK10
MOL005003	GC78	GAN CAO	1eub_v	0.9824	MMP13_HUMAN	MMP13
MOL005003	GC78	GAN CAO	1bm6_v	0.9248	MMP3_HUMAN	MMP3
MOL005003	GC78	GAN CAO	1le6_v	0.9295	O15496	PLA2G10
MOL005003	GC78	GAN CAO	2pin_v	0.9211	P10828	THRB
MOL005003	GC78	GAN CAO	3bgp_v	0.9866	P11309	PIM1
MOL005003	GC78	GAN CAO	1jvp_v	0.9544	P24941	CDK2
MOL005003	GC78	GAN CAO	2p3g_v	0.9887	P49137	MAPKAPK2
MOL005003	GC78	GAN CAO	1udu_v	0.9694	PDE5A_HUMAN	PDE5A
MOL005003	GC78	GAN CAO	2a4z_v	0.9803	PK3CG_HUMAN	PIK3CG
MOL005003	GC78	GAN CAO	1zgy_v	0.9356	PPARG_HUMAN	PPARG
MOL005003	GC78	GAN CAO	1p49_v	0.9483	STS_HUMAN	STS

MOL005003	GC78	GAN CAO	1dvz_v	0.9651	TTHY_HUMAN	TTR
MOL005007	GC79	GAN CAO	1j96_v	0.9613	AK1C2_HUMAN	AKR1C2
MOL005007	GC79	GAN CAO	1e7a_v	0.9013	ALBU_HUMAN	ALB
MOL005007	GC79	GAN CAO	1oj9_v	0.9787	AOFB_HUMAN	MAOB
MOL005007	GC79	GAN CAO	2ohp_v	0.9882	BACE1_HUMAN	BACE1
MOL005007	GC79	GAN CAO	1reu_v	0.9067	BMP2_HUMAN	BMP2
MOL005007	GC79	GAN CAO	1if4_v	0.9594	CAH2_HUMAN	CA2
MOL005007	GC79	GAN CAO	1shj_v	0.9248	CASP7_HUMAN	CASP7
MOL005007	GC79	GAN CAO	1rs0_v	0.9461	CFAB_HUMAN	CFB
MOL005007	GC79	GAN CAO	1xlu_v	0.9574	CHLE_HUMAN	BCHE
MOL005007	GC79	GAN CAO	2vwu_v	0.947	EPHB4_HUMAN	EPHB4
MOL005007	GC79	GAN CAO	1qku_v	0.9232	ESR1_HUMAN	ESR1
MOL005007	GC79	GAN CAO	1ogs_v	0.9219	GLCM_HUMAN	GBA
MOL005007	GC79	GAN CAO	1md3_v	0.9119	GSTP1_HUMAN	GSTP1
MOL005007	GC79	GAN CAO	2q2y_v	0.9403	KIF11_HUMAN	KIF11
MOL005007	GC79	GAN CAO	1uki_v	0.965	MK08_HUMAN	MAPK8
MOL005007	GC79	GAN CAO	3cgf_v	0.9675	MK10_HUMAN	MAPK10
MOL005007	GC79	GAN CAO	3bgp_v	0.9834	P11309	PIM1
MOL005007	GC79	GAN CAO	3cjf_v	0.9675	P35968	KDR
MOL005007	GC79	GAN CAO	1w8l_v	0.9653	P62937	PPIA
MOL005007	GC79	GAN CAO	2a4z_v	0.9345	PK3CG_HUMAN	PIK3CG
MOL005007	GC79	GAN CAO	1no9_v	0.9357	THRB_HUMAN	F2
MOL000392	GC8	GAN CAO	1x7r_v	0.9884	ESR1_HUMAN	ESR1
MOL000392	GC8	GAN CAO	2jj3_v	0.9486	ESR2_HUMAN	ESR2
MOL005008	GC80	GAN CAO	1j96_v	0.9061	AK1C2_HUMAN	AKR1C2
MOL005008	GC80	GAN CAO	2pix_v	0.9269	ANDR_HUMAN	AR
MOL005008	GC80	GAN CAO	1oj9_v	1	AOFB_HUMAN	MAOB
MOL005008	GC80	GAN CAO	2ohp_v	0.9067	BACE1_HUMAN	BACE1
MOL005008	GC80	GAN CAO	1reu_v	0.9425	BMP2_HUMAN	BMP2
MOL005008	GC80	GAN CAO	1czm_v	0.9367	CAH1_HUMAN	CA1
MOL005008	GC80	GAN CAO	1if8_v	0.9924	CAH2_HUMAN	CA2
MOL005008	GC80	GAN CAO	1gfw_v	0.94	CASP3_HUMAN	CASP3
MOL005008	GC80	GAN CAO	1shj_v	1	CASP7_HUMAN	CASP7
MOL005008	GC80	GAN CAO	1rs0_v	0.9772	CFAB_HUMAN	CFB
MOL005008	GC80	GAN CAO	1p0i_v	0.9595	CHLE_HUMAN	BCHE
MOL005008	GC80	GAN CAO	1yb1_v	0.9906	DHB11_HUMAN	HSD17B11
MOL005008	GC80	GAN CAO	2itx_v	0.938	EGFR_HUMAN	EGFR
MOL005008	GC80	GAN CAO	2i0j_v	0.9935	ESR1_HUMAN	ESR1
MOL005008	GC80	GAN CAO	2hck_v	0.9944	HCK_HUMAN	HCK
MOL005008	GC80	GAN CAO	1uki_v	0.9994	MK08_HUMAN	MAPK8
MOL005008	GC80	GAN CAO	1pmv_v	0.9961	MK10_HUMAN	MAPK10
MOL005008	GC80	GAN CAO	1bm6_v	0.9821	MMP3_HUMAN	MMP3

MOL005008	GC80	GAN CAO	2obj_v	0.9331	P11309	PIM1
MOL005008	GC80	GAN CAO	3eqm_v	0.9287	P11511	CYP19A1
MOL005008	GC80	GAN CAO	2vth_v	0.9508	P24941	CDK2
MOL005008	GC80	GAN CAO	2p3g_v	0.9741	P49137	MAPKAPK2
MOL005008	GC80	GAN CAO	1w8m_v	0.942	P62937	PPIA
MOL005008	GC80	GAN CAO	2j7x_v	0.9351	Q62986	Esr2
MOL005008	GC80	GAN CAO	1no9_v	0.9719	THRB_HUMAN	F2
MOL005008	GC80	GAN CAO	1dvz_v	0.9482	TTHY_HUMAN	TTR
MOL005012	GC81	GAN CAO	1e7e_v	0.9447	ALBU_HUMAN	ALB
MOL005012	GC81	GAN CAO	1xow_v	0.9665	ANDR_HUMAN	AR
MOL005012	GC81	GAN CAO	1l6l_v	0.9419	APOA2_HUMAN	APOA2
MOL005012	GC81	GAN CAO	2ohp_v	0.9787	BACE1_HUMAN	BACE1
MOL005012	GC81	GAN CAO	3bmp_v	0.9758	BMP2_HUMAN	BMP2
MOL005012	GC81	GAN CAO	1czm_v	0.9629	CAH1_HUMAN	CA1
MOL005012	GC81	GAN CAO	1xlu_v	0.9627	CHLE_HUMAN	BCHE
MOL005012	GC81	GAN CAO	1qku_v	0.9047	ESR1_HUMAN	ESR1
MOL005012	GC81	GAN CAO	2i0g_v	0.9463	ESR2_HUMAN	ESR2
MOL005012	GC81	GAN CAO	1yxx_v	0.9916	PIM1_HUMAN	PIM1
MOL005012	GC81	GAN CAO	1w7h_v	0.9548	Q16539	MAPK14
MOL005012	GC81	GAN CAO	1p49_v	0.9634	STS_HUMAN	STS
MOL005013	GC82	GAN CAO	1j96_v	1	AK1C2_HUMAN	AKR1C2
MOL005013	GC82	GAN CAO	1e7e_v	0.9779	ALBU_HUMAN	ALB
MOL005013	GC82	GAN CAO	2pio_v	0.9642	ANDR_HUMAN	AR
MOL005013	GC82	GAN CAO	1oj9_v	0.9265	AOFB_HUMAN	MAOB
MOL005013	GC82	GAN CAO	1l6l_v	0.9943	APOA2_HUMAN	APOA2
MOL005013	GC82	GAN CAO	2ohp_v	0.9931	BACE1_HUMAN	BACE1
MOL005013	GC82	GAN CAO	1reu_v	1	BMP2_HUMAN	BMP2
MOL005013	GC82	GAN CAO	1rs0_v	0.983	CFAB_HUMAN	CFB
MOL005013	GC82	GAN CAO	1p0i_v	0.9223	CHLE_HUMAN	BCHE
MOL005013	GC82	GAN CAO	3czr_v	0.9445	DHI1_HUMAN	HSD11B1
MOL005013	GC82	GAN CAO	1qku_v	0.9731	ESR1_HUMAN	ESR1
MOL005013	GC82	GAN CAO	2pg2_v	0.9115	KIF11_HUMAN	KIF11
MOL005013	GC82	GAN CAO	1uki_v	0.9385	MK08_HUMAN	MAPK8
MOL005013	GC82	GAN CAO	1pmv_v	0.9919	MK10_HUMAN	MAPK10
MOL005013	GC82	GAN CAO	1eub_v	0.9093	MMP13_HUMAN	MMP13
MOL005013	GC82	GAN CAO	1bm6_v	0.9445	MMP3_HUMAN	MMP3
MOL005013	GC82	GAN CAO	3dd8_v	0.9436	P00918	CA2
MOL005013	GC82	GAN CAO	2pin_v	0.9561	P10828	THRB
MOL005013	GC82	GAN CAO	3bgp_v	1	P11309	PIM1
MOL005013	GC82	GAN CAO	2p3g_v	0.9945	P49137	MAPKAPK2
MOL005013	GC82	GAN CAO	1w8l_v	0.9385	P62937	PPIA
MOL005013	GC82	GAN CAO	1hd2_v	0.9347	PRDX5_HUMAN	PRDX5

MOL005013	GC82	GAN CAO	1p49_v	1	STS_HUMAN	STS
MOL005013	GC82	GAN CAO	1no9_v	0.9852	THRB_HUMAN	F2
MOL005013	GC82	GAN CAO	1ijn_v	0.9889	TTHY_HUMAN	TTR
MOL005013	GC82	GAN CAO	1j78_v	0.9212	VTDB_HUMAN	GC
MOL005016	GC83	GAN CAO	2pio_v	0.9552	ANDR_HUMAN	AR
MOL005016	GC83	GAN CAO	2ohp_v	0.9335	BACE1_HUMAN	BACE1
MOL005016	GC83	GAN CAO	1oq5_v	0.9694	CAH2_HUMAN	CA2
MOL005016	GC83	GAN CAO	1h1p_v	0.9143	CCNA2_HUMAN	CCNA2
MOL005016	GC83	GAN CAO	1xlv_v	0.986	CHLE_HUMAN	BCHE
MOL005016	GC83	GAN CAO	1x7r_v	0.9795	ESR1_HUMAN	ESR1
MOL005016	GC83	GAN CAO	1x7j_v	0.9881	ESR2_HUMAN	ESR2
MOL005016	GC83	GAN CAO	1ogs_v	0.9318	GLCM_HUMAN	GBA
MOL005016	GC83	GAN CAO	1uki_v	0.974	MK08_HUMAN	MAPK8
MOL005016	GC83	GAN CAO	1pmv_v	0.9554	MK10_HUMAN	MAPK10
MOL005016	GC83	GAN CAO	1bm6_v	0.9752	MMP3_HUMAN	MMP3
MOL005016	GC83	GAN CAO	1pq9_v	0.9787	NR1H2_HUMAN	NR1H2
MOL005016	GC83	GAN CAO	3eqm_v	0.967	P11511	CYP19A1
MOL005016	GC83	GAN CAO	1w8m_v	0.9636	P62937	PPIA
MOL005016	GC83	GAN CAO	1yxx_v	0.9656	PIM1_HUMAN	PIM1
MOL005016	GC83	GAN CAO	1zuc_v	0.9042	PRGR_HUMAN	PGR
MOL005016	GC83	GAN CAO	2afu_v	0.9457	QPCT_HUMAN	QPCT
MOL005016	GC83	GAN CAO	1z68_v	0.9798	SEPR_HUMAN	FAP
MOL005016	GC83	GAN CAO	1dvz_v	0.9614	TTHY_HUMAN	TTR
MOL005017	GC84	GAN CAO	2oi0_v	0.916	ADA17_HUMAN	ADAM17
MOL005017	GC84	GAN CAO	1e7a_v	1	ALBU_HUMAN	ALB
MOL005017	GC84	GAN CAO	2pir_v	0.9078	ANDR_HUMAN	AR
MOL005017	GC84	GAN CAO	1oj9_v	1	AOFB_HUMAN	MAOB
MOL005017	GC84	GAN CAO	1l6l_v	1	APOA2_HUMAN	APOA2
MOL005017	GC84	GAN CAO	2ohp_v	0.9651	BACE1_HUMAN	BACE1
MOL005017	GC84	GAN CAO	1if4_v	0.9818	CAH2_HUMAN	CA2
MOL005017	GC84	GAN CAO	1shj_v	1	CASP7_HUMAN	CASP7
MOL005017	GC84	GAN CAO	1unh_v	0.9764	CD5R1_HUMAN	CDK5R1
MOL005017	GC84	GAN CAO	1rs0_v	0.9964	CFAB_HUMAN	CFB
MOL005017	GC84	GAN CAO	2ocf_v	0.9988	ESR1_HUMAN	ESR1
MOL005017	GC84	GAN CAO	2jj3_v	0.9744	ESR2_HUMAN	ESR2
MOL005017	GC84	GAN CAO	1ya8_v	0.9881	EST1_HUMAN	CES1
MOL005017	GC84	GAN CAO	2fky_v	1	KIF11_HUMAN	KIF11
MOL005017	GC84	GAN CAO	1pme_v	1	MK01_HUMAN	MAPK1
MOL005017	GC84	GAN CAO	1uki_v	0.9904	MK08_HUMAN	MAPK8
MOL005017	GC84	GAN CAO	3cgf_v	0.9887	MK10_HUMAN	MAPK10
MOL005017	GC84	GAN CAO	830c_v	0.986	MMP13_HUMAN	MMP13
MOL005017	GC84	GAN CAO	1bm6_v	0.9975	MMP3_HUMAN	MMP3

MOL005017	GC84	GAN CAO	1le6_v	0.9114	O15496	PLA2G10
MOL005017	GC84	GAN CAO	3bgp_v	1	P11309	PIM1
MOL005017	GC84	GAN CAO	2b53_v	0.9983	P24941	CDK2
MOL005017	GC84	GAN CAO	3cjf_v	0.9887	P35968	KDR
MOL005017	GC84	GAN CAO	2p3g_v	1	P49137	MAPKAPK2
MOL005017	GC84	GAN CAO	2pe0_v	0.9831	PDPK1_HUMAN	PDPK1
MOL005017	GC84	GAN CAO	1ov4_v	0.942	Q06520	SULT2A1
MOL005017	GC84	GAN CAO	1wbo_v	0.9984	Q16539	MAPK14
MOL005017	GC84	GAN CAO	1n83_v	1	RORA_HUMAN	RORA
MOL005017	GC84	GAN CAO	1p49_v	1	STS_HUMAN	STS
MOL005017	GC84	GAN CAO	1no9_v	0.9846	THRB_HUMAN	F2
MOL005017	GC84	GAN CAO	1dvz_v	0.9912	TTHY_HUMAN	TTR
MOL005017	GC84	GAN CAO	1j78_v	1	VTDB_HUMAN	GC
MOL005018	GC85	GAN CAO	1j96_v	0.9815	AK1C2_HUMAN	AKR1C2
MOL005018	GC85	GAN CAO	1e7e_v	0.9555	ALBU_HUMAN	ALB
MOL005018	GC85	GAN CAO	1oj9_v	0.9877	AOFB_HUMAN	MAOB
MOL005018	GC85	GAN CAO	1l6l_v	0.9644	APOA2_HUMAN	APOA2
MOL005018	GC85	GAN CAO	1reu_v	0.9548	BMP2_HUMAN	BMP2
MOL005018	GC85	GAN CAO	1if4_v	0.9687	CAH2_HUMAN	CA2
MOL005018	GC85	GAN CAO	1shj_v	0.9872	CASP7_HUMAN	CASP7
MOL005018	GC85	GAN CAO	2i0j_v	0.9624	ESR1_HUMAN	ESR1
MOL005018	GC85	GAN CAO	2obj_v	0.9901	P11309	PIM1
MOL005018	GC85	GAN CAO	2jbp_v	0.9924	P49137	MAPKAPK2
MOL005018	GC85	GAN CAO	2a4z_v	0.9557	PK3CG_HUMAN	PIK3CG
MOL005018	GC85	GAN CAO	1p49_v	0.9826	STS_HUMAN	STS
MOL005020	GC86	GAN CAO	1e7e_v	0.961	ALBU_HUMAN	ALB
MOL005020	GC86	GAN CAO	2ama_v	0.9214	ANDR_HUMAN	AR
MOL005020	GC86	GAN CAO	1oj9_v	0.9991	AOFB_HUMAN	MAOB
MOL005020	GC86	GAN CAO	1l6l_v	0.9668	APOA2_HUMAN	APOA2
MOL005020	GC86	GAN CAO	2ohp_v	0.9543	BACE1_HUMAN	BACE1
MOL005020	GC86	GAN CAO	1if4_v	0.9739	CAH2_HUMAN	CA2
MOL005020	GC86	GAN CAO	3dej_v	0.9622	CASP3_HUMAN	CASP3
MOL005020	GC86	GAN CAO	1shj_v	0.9775	CASP7_HUMAN	CASP7
MOL005020	GC86	GAN CAO	1unh_v	0.9749	CD5R1_HUMAN	CDK5R1
MOL005020	GC86	GAN CAO	1rs0_v	0.9804	CFAB_HUMAN	CFB
MOL005020	GC86	GAN CAO	2ocf_v	0.9779	ESR1_HUMAN	ESR1
MOL005020	GC86	GAN CAO	1qkm_v	0.9846	ESR2_HUMAN	ESR2
MOL005020	GC86	GAN CAO	1ya8_v	0.9497	EST1_HUMAN	CES1
MOL005020	GC86	GAN CAO	1d7i_v	0.9288	FKB1A_HUMAN	FKBP1A
MOL005020	GC86	GAN CAO	2fky_v	0.924	KIF11_HUMAN	KIF11
MOL005020	GC86	GAN CAO	1pme_v	0.994	MK01_HUMAN	MAPK1
MOL005020	GC86	GAN CAO	1uki_v	0.9071	MK08_HUMAN	MAPK8

MOL005020	GC86	GAN CAO	1pmv_v	0.9015	MK10_HUMAN	MAPK10
MOL005020	GC86	GAN CAO	1bm6_v	0.9417	MMP3_HUMAN	MMP3
MOL005020	GC86	GAN CAO	1jvp_v	0.9055	P24941	CDK2
MOL005020	GC86	GAN CAO	2jbp_v	0.9776	P49137	APKAPK2
MOL005020	GC86	GAN CAO	1w8l_v	0.9728	P62937	PPIA
MOL005020	GC86	GAN CAO	1udu_v	0.9186	PDE5A_HUMAN	PDE5A
MOL005020	GC86	GAN CAO	1yxx_v	0.9828	PIM1_HUMAN	PIM1
MOL005020	GC86	GAN CAO	1wbo_v	0.9927	Q16539	MAPK14
MOL005020	GC86	GAN CAO	1q8m_v	0.989	Q9NP99	TREM1
MOL005020	GC86	GAN CAO	1p49_v	0.9711	STS_HUMAN	STS
MOL005020	GC86	GAN CAO	2roy_v	0.9429	TTHY_HUMAN	TTR
MOL005020	GC86	GAN CAO	1j78_v	0.9171	VTDB_HUMAN	GC
MOL000098	GC87	GAN CAO	1oq5_v	0.9077	CAH2_HUMAN	CA2
MOL000098	GC87	GAN CAO	1p0i_v	0.9492	CHLE_HUMAN	BCHE
MOL000098	GC87	GAN CAO	2itx_v	0.9333	EGFR_HUMAN	EGFR
MOL000098	GC87	GAN CAO	2i0j_v	0.9615	ESR1_HUMAN	ESR1
MOL000098	GC87	GAN CAO	2hck_v	0.9854	HCK_HUMAN	HCK
MOL000098	GC87	GAN CAO	2o63_v	0.989	PIM1_HUMAN	PIM1
MOL000098	GC87	GAN CAO	1zuc_v	0.9356	PRGR_HUMAN	PGR
MOL000098	GC87	GAN CAO	1no9_v	0.9491	THRB_HUMAN	F2
MOL000098	GC87	GAN CAO	1dvz_v	0.9264	TTHY_HUMAN	TTR
MOL000497	GC88	GAN CAO	1j96_v	0.9992	AK1C2_HUMAN	AKR1C2
MOL000497	GC88	GAN CAO	1xf0_v	0.9478	AK1C3_HUMAN	AKR1C3
MOL000497	GC88	GAN CAO	1xj7_v	0.9697	ANDR_HUMAN	AR
MOL000497	GC88	GAN CAO	1gfw_v	0.9196	CASP3_HUMAN	CASP3
MOL000497	GC88	GAN CAO	1unh_v	0.908	CD5R1_HUMAN	CDK5R1
MOL000497	GC88	GAN CAO	2vwu_v	0.9674	EPHB4_HUMAN	EPHB4
MOL000497	GC88	GAN CAO	1qku_v	0.9615	ESR1_HUMAN	ESR1
MOL000497	GC88	GAN CAO	2jj3_v	0.9102	ESR2_HUMAN	ESR2
MOL000497	GC88	GAN CAO	3dcw_v	0.9682	P00918	CA2
MOL000497	GC88	GAN CAO	2obj_v	0.967	P11309	PIM1
MOL000497	GC88	GAN CAO	3eqm_v	0.949	P11511	CYP19A1
MOL000497	GC88	GAN CAO	1wbo_v	0.9631	Q16539	MAPK14
MOL000497	GC88	GAN CAO	1q8m_v	0.9863	Q9NP99	TREM1
MOL001484	GC89	GAN CAO	1e7e_v	0.9523	ALBU_HUMAN	ALB
MOL001484	GC89	GAN CAO	2pir_v	0.9579	ANDR_HUMAN	AR
MOL001484	GC89	GAN CAO	1l6l_v	0.9728	APOA2_HUMAN	APOA2
MOL001484	GC89	GAN CAO	1reu_v	0.9729	BMP2_HUMAN	BMP2
MOL001484	GC89	GAN CAO	1if4_v	0.9187	CAH2_HUMAN	CA2
MOL001484	GC89	GAN CAO	1vec_v	0.9772	DDX6_HUMAN	DDX6
MOL001484	GC89	GAN CAO	1qku_v	0.961	ESR1_HUMAN	ESR1
MOL001484	GC89	GAN CAO	1md3_v	0.9676	GSTP1_HUMAN	GSTP1

MOL001484	GC89	GAN CAO	2obj_v	0.9527	P11309	PIM1
MOL001484	GC89	GAN CAO	1w8m_v	0.9128	P62937	PPIA
MOL001484	GC89	GAN CAO	2a4z_v	0.9077	PK3CG_HUMAN	PIK3CG
MOL001484	GC89	GAN CAO	1p49_v	0.9964	STS_HUMAN	STS
MOL000417	GC9	GAN CAO	1zh9_v	0.9057	CAH2_HUMAN	CA2
MOL000417	GC9	GAN CAO	2i0j_v	0.9957	ESR1_HUMAN	ESR1
MOL000417	GC9	GAN CAO	2jj3_v	0.9708	ESR2_HUMAN	ESR2
MOL000417	GC9	GAN CAO	1yxx_v	0.9099	PIM1_HUMAN	PIM1
MOL000417	GC9	GAN CAO	1z68_v	0.9579	SEPR_HUMAN	FAP
MOL001792	GC90	GAN CAO	1xo2_v	0.9084	CDK6_HUMAN	CDK6
MOL001792	GC90	GAN CAO	2i0j_v	0.9539	ESR1_HUMAN	ESR1
MOL001792	GC90	GAN CAO	1yxx_v	0.9627	PIM1_HUMAN	PIM1
MOL001792	GC90	GAN CAO	1no9_v	0.9173	THRB_HUMAN	F2
MOL002464	GJ1	GAN JIANG	1j96_v	0.994	AK1C2_HUMAN	AKR1C2
MOL002464	GJ1	GAN JIANG	1e7a_v	0.9989	ALBU_HUMAN	ALB
MOL002464	GJ1	GAN JIANG	1oj9_v	0.9965	AOFB_HUMAN	MAOB
MOL002464	GJ1	GAN JIANG	1l6l_v	0.9764	APOA2_HUMAN	APOA2
MOL002464	GJ1	GAN JIANG	2q11_v	0.9562	BACE1_HUMAN	BACE1
MOL002464	GJ1	GAN JIANG	1reu_v	0.9999	BMP2_HUMAN	BMP2
MOL002464	GJ1	GAN JIANG	1jd0_v	0.9409	CAH12_HUMAN	CA12
MOL002464	GJ1	GAN JIANG	1shj_v	0.9937	CASP7_HUMAN	CASP7
MOL002464	GJ1	GAN JIANG	2f1g_v	0.948	CATS_HUMAN	CTSS
MOL002464	GJ1	GAN JIANG	2uzd_v	0.9449	CCNA2_HUMAN	CCNA2
MOL002464	GJ1	GAN JIANG	1rs0_v	0.9306	CFAB_HUMAN	CFB
MOL002464	GJ1	GAN JIANG	2cgw_v	0.9733	CHK1_HUMAN	CHEK1
MOL002464	GJ1	GAN JIANG	1xlu_v	0.9726	CHLE_HUMAN	BCHE
MOL002464	GJ1	GAN JIANG	1vec_v	0.9657	DDX6_HUMAN	DDX6
MOL002464	GJ1	GAN JIANG	1qkt_v	0.9115	ESR1_HUMAN	ESR1
MOL002464	GJ1	GAN JIANG	1f40_v	0.9661	FKB1A_HUMAN	FKBP1A
MOL002464	GJ1	GAN JIANG	1md3_v	0.9578	GSTP1_HUMAN	GSTP1
MOL002464	GJ1	GAN JIANG	2pg2_v	0.9763	KIF11_HUMAN	KIF11

MOL002464	GJ1	GAN JIANG	1pme_v	0.9957	MK01_HUMAN	MAPK1
MOL002464	GJ1	GAN JIANG	2gmx_v	0.94	MK08_HUMAN	MAPK8
MOL002464	GJ1	GAN JIANG	1eub_v	0.9702	MMP13_HUMAN	MMP13
MOL002464	GJ1	GAN JIANG	1pq9_v	0.9436	NR1H2_HUMAN	NR1H2
MOL002464	GJ1	GAN JIANG	3dbu_v	0.9789	P00918	CA2
MOL002464	GJ1	GAN JIANG	2pin_v	0.9905	P10828	THRB
MOL002464	GJ1	GAN JIANG	3bgp_v	0.9879	P11309	PIM1
MOL002464	GJ1	GAN JIANG	1e1x_v	0.9567	P24941	CDK2
MOL002464	GJ1	GAN JIANG	2jbp_v	0.9984	P49137	MAPKAPK2
MOL002464	GJ1	GAN JIANG	1w8l_v	0.9889	P62937	PPIA
MOL002464	GJ1	GAN JIANG	1xn0_v	0.9221	PDE4B_HUMAN	PDE4B
MOL002464	GJ1	GAN JIANG	1zgy_v	0.9794	PPARG_HUMAN	PPARG
MOL002464	GJ1	GAN JIANG	1p49_v	0.9896	STS_HUMAN	STS
MOL002464	GJ1	GAN JIANG	1m9z_v	0.9562	TGFR2_HUMAN	TGFBR2
MOL002464	GJ1	GAN JIANG	1dvz_v	0.9829	TTHY_HUMAN	TTR
MOL002464	GJ1	GAN JIANG	1j78_v	0.9123	VTDB_HUMAN	GC
MOL002501	GJ2	GAN JIANG	1j96_v	0.9996	AK1C2_HUMAN	AKR1C2
MOL002501	GJ2	GAN JIANG	1xf0_v	0.928	AK1C3_HUMAN	AKR1C3
MOL002501	GJ2	GAN JIANG	1e7e_v	0.9421	ALBU_HUMAN	ALB
MOL002501	GJ2	GAN JIANG	2agt_v	0.9333	ALDR_HUMAN	AKR1B1
MOL002501	GJ2	GAN JIANG	1xj7_v	0.9678	ANDR_HUMAN	AR
MOL002501	GJ2	GAN JIANG	1oj9_v	0.9834	AOFB_HUMAN	MAOB
MOL002501	GJ2	GAN JIANG	1l6l_v	0.9464	APOA2_HUMAN	APOA2
MOL002501	GJ2	GAN JIANG	1reu_v	0.9615	BMP2_HUMAN	BMP2
MOL002501	GJ2	GAN JIANG	1okl_v	0.9607	CAH2_HUMAN	CA2
MOL002501	GJ2	GAN JIANG	1shj_v	0.9266	CASP7_HUMAN	CASP7

MOL002501	GJ2	GAN JIANG	1lyw_v	0.9233	CATD_HUMAN	CTSD
MOL002501	GJ2	GAN JIANG	1rs0_v	0.9993	CFAB_HUMAN	CFB
MOL002501	GJ2	GAN JIANG	1p0i_v	0.9467	CHLE_HUMAN	BCHE
MOL002501	GJ2	GAN JIANG	1klt_v	0.9203	CMA1_HUMAN	CMA1
MOL002501	GJ2	GAN JIANG	1ya8_v	0.9221	EST1_HUMAN	CES1
MOL002501	GJ2	GAN JIANG	1md3_v	0.9725	GSTP1_HUMAN	GSTP1
MOL002501	GJ2	GAN JIANG	1xdd_v	0.9783	ITAL_HUMAN	ITGAL
MOL002501	GJ2	GAN JIANG	2pg2_v	0.95	KIF11_HUMAN	KIF11
MOL002501	GJ2	GAN JIANG	1pme_v	0.9566	MK01_HUMAN	MAPK1
MOL002501	GJ2	GAN JIANG	1uki_v	0.9335	MK08_HUMAN	MAPK8
MOL002501	GJ2	GAN JIANG	1pmv_v	0.9703	MK10_HUMAN	MAPK10
MOL002501	GJ2	GAN JIANG	1eub_v	0.9352	MMP13_HUMAN	MMP13
MOL002501	GJ2	GAN JIANG	1bm6_v	0.9789	MMP3_HUMAN	MMP3
MOL002501	GJ2	GAN JIANG	1pq9_v	0.9319	NR1H2_HUMAN	NR1H2
MOL002501	GJ2	GAN JIANG	3bgp_v	0.9874	P11309	PIM1
MOL002501	GJ2	GAN JIANG	3eqm_v	0.9509	P11511	CYP19A1
MOL002501	GJ2	GAN JIANG	2jbp_v	0.9711	P49137	MAPKAPK2
MOL002501	GJ2	GAN JIANG	1w8l_v	0.9394	P62937	PPIA
MOL002501	GJ2	GAN JIANG	1v3q_v	0.9985	PNPH_HUMAN	PNP
MOL002501	GJ2	GAN JIANG	1zgy_v	0.9141	PPARG_HUMAN	PPARG
MOL002501	GJ2	GAN JIANG	1zyj_v	0.9017	Q16539	MAPK14
MOL002501	GJ2	GAN JIANG	1q8m_v	0.9801	Q9NP99	TREM1
MOL002501	GJ2	GAN JIANG	1p49_v	0.9544	STS_HUMAN	STS
MOL002501	GJ2	GAN JIANG	1no9_v	0.9366	THRB_HUMAN	F2
MOL002501	GJ2	GAN JIANG	1dvz_v	0.9818	TTHY_HUMAN	TTR
MOL002501	GJ2	GAN JIANG	1j78_v	0.9029	VTDB_HUMAN	GC

MOL002514	GJ3	GAN JIANG	2osf_v	0.9358	CAH2_HUMAN	CA2
MOL002514	GJ3	GAN JIANG	1xo2_v	0.9324	CDK6_HUMAN	CDK6
MOL002514	GJ3	GAN JIANG	1xlu_v	0.9311	CHLE_HUMAN	BCHE
MOL002514	GJ3	GAN JIANG	2itx_v	0.9464	EGFR_HUMAN	EGFR
MOL002514	GJ3	GAN JIANG	2pog_v	0.9682	ESR1_HUMAN	ESR1
MOL002514	GJ3	GAN JIANG	1ya8_v	0.9682	EST1_HUMAN	CES1
MOL002514	GJ3	GAN JIANG	2hck_v	0.9929	HCK_HUMAN	HCK
MOL002879	RS1	REN SHEN	1zxc_v	0.9616	ADA17_HUMAN	ADAM17
MOL002879	RS1	REN SHEN	1j96_v	0.9853	AK1C2_HUMAN	AKR1C2
MOL002879	RS1	REN SHEN	1xf0_v	0.9549	AK1C3_HUMAN	AKR1C3
MOL002879	RS1	REN SHEN	1e7e_v	0.9335	ALBU_HUMAN	ALB
MOL002879	RS1	REN SHEN	1az1_v	0.9578	ALDR_HUMAN	AKR1B1
MOL002879	RS1	REN SHEN	1xj7_v	0.9597	ANDR_HUMAN	AR
MOL002879	RS1	REN SHEN	1hak_v	0.9035	ANXA5_HUMAN	ANXA5
MOL002879	RS1	REN SHEN	1oj9_v	0.9962	AOFB_HUMAN	MAOB
MOL002879	RS1	REN SHEN	1l6l_v	0.9641	APOA2_HUMAN	APOA2
MOL002879	RS1	REN SHEN	3bmp_v	0.9855	BMP2_HUMAN	BMP2
MOL002879	RS1	REN SHEN	3dej_v	0.9969	CASP3_HUMAN	CASP3
MOL002879	RS1	REN SHEN	1shj_v	0.9442	CASP7_HUMAN	CASP7
MOL002879	RS1	REN SHEN	1csb_v	0.9424	CATB_HUMAN	CTSB
MOL002879	RS1	REN SHEN	2uzd_v	0.9752	CCNA2_HUMAN	CCNA2
MOL002879	RS1	REN SHEN	1unh_v	0.9793	CD5R1_HUMAN	CDK5R1
MOL002879	RS1	REN SHEN	1rs0_v	0.965	CFAB_HUMAN	CFB
MOL002879	RS1	REN SHEN	1p0i_v	0.9891	CHLE_HUMAN	BCHE
MOL002879	RS1	REN SHEN	1klt_v	0.9698	CMA1_HUMAN	CMA1
MOL002879	RS1	REN SHEN	1vec_v	0.9873	DDX6_HUMAN	DDX6
MOL002879	RS1	REN SHEN	1m17_v	0.9351	EGFR_HUMAN	EGFR
MOL002879	RS1	REN SHEN	2ocf_v	0.9332	ESR1_HUMAN	ESR1
MOL002879	RS1	REN SHEN	1ya8_v	0.9491	EST1_HUMAN	CES1
MOL002879	RS1	REN SHEN	2j95_v	0.921	FA10_HUMAN	F10
MOL002879	RS1	REN SHEN	1f40_v	0.9596	FKB1A_HUMAN	FKBP1A
MOL002879	RS1	REN SHEN	1lbk_v	0.975	GSTP1_HUMAN	GSTP1
MOL002879	RS1	REN SHEN	2fky_v	0.9684	KIF11_HUMAN	KIF11
MOL002879	RS1	REN SHEN	1pme_v	0.9588	MK01_HUMAN	MAPK1
MOL002879	RS1	REN SHEN	1uki_v	0.9898	MK08_HUMAN	MAPK8
MOL002879	RS1	REN SHEN	1pmv_v	0.9918	MK10_HUMAN	MAPK10
MOL002879	RS1	REN SHEN	1eub_v	0.96	MMP13_HUMAN	MMP13
MOL002879	RS1	REN SHEN	1bm6_v	0.9951	MMP3_HUMAN	MMP3

MOL002879	RS1	REN SHEN	1pq9_v	0.9603	NR1H2_HUMAN	NR1H2
MOL002879	RS1	REN SHEN	3dcw_v	0.9874	P00918	CA2
MOL002879	RS1	REN SHEN	2pin_v	0.9469	P10828	THRB
MOL002879	RS1	REN SHEN	3bgp_v	0.9887	P11309	PIM1
MOL002879	RS1	REN SHEN	3eqm_v	0.9429	P11511	CYP19A1
MOL002879	RS1	REN SHEN	2jbp_v	0.99	P49137	1APKAPK2
MOL002879	RS1	REN SHEN	1w8m_v	0.9584	P62937	PPIA
MOL002879	RS1	REN SHEN	2ovm_v	0.9153	PRGR_HUMAN	PGR
MOL002879	RS1	REN SHEN	1zyj_v	0.9388	Q16539	MAPK14
MOL002879	RS1	REN SHEN	1q8m_v	0.9673	Q9NP99	TREM1
MOL002879	RS1	REN SHEN	1p49_v	0.9695	STS_HUMAN	STS
MOL002879	RS1	REN SHEN	1no9_v	0.9345	THRB_HUMAN	F2
MOL002879	RS1	REN SHEN	1f86_v	0.9993	TTHY_HUMAN	TTR
MOL005318	RS10	REN SHEN	2pio_v	0.9718	ANDR_HUMAN	AR
MOL005318	RS10	REN SHEN	1v83_v	0.9759	B3GA1_HUMAN	B3GAT1
MOL005318	RS10	REN SHEN	2ohp_v	0.9684	BACE1_HUMAN	BACE1
MOL005318	RS10	REN SHEN	1bzm_v	0.9481	CAH1_HUMAN	CA1
MOL005318	RS10	REN SHEN	1lyw_v	0.9487	CATD_HUMAN	CTSD
MOL005318	RS10	REN SHEN	2bkz_v	0.9419	CCNA2_HUMAN	CCNA2
MOL005318	RS10	REN SHEN	2itx_v	0.9322	EGFR_HUMAN	EGFR
MOL005318	RS10	REN SHEN	1xka_v	0.9729	FA10_HUMAN	F10
MOL005318	RS10	REN SHEN	9jdw_v	0.9791	GATM_HUMAN	GATM
MOL005318	RS10	REN SHEN	1lbk_v	0.9438	GSTP1_HUMAN	GSTP1
MOL005318	RS10	REN SHEN	1uki_v	0.9647	MK08_HUMAN	MAPK8
MOL005318	RS10	REN SHEN	1pmv_v	0.9732	MK10_HUMAN	MAPK10
MOL005318	RS10	REN SHEN	1l6m_v	0.97	NGAL_HUMAN	LCN2
MOL005318	RS10	REN SHEN	1pq9_v	0.9788	NR1H2_HUMAN	NR1H2
MOL005318	RS10	REN SHEN	1kta_v	0.9595	O15382	BCAT2
MOL005318	RS10	REN SHEN	1gyk_v	0.9254	P02743	APCS
MOL005318	RS10	REN SHEN	1smd_v	0.957	P04745	AMY1A
MOL005318	RS10	REN SHEN	1mkj_v	0.9759	P33176	KIF5B
MOL005318	RS10	REN SHEN	3cjf_v	0.9698	P35968	KDR
MOL005318	RS10	REN SHEN	1oni_v	0.9984	P52758	RIDA
MOL005318	RS10	REN SHEN	1w8m_v	0.963	P62937	PPIA
MOL005318	RS10	REN SHEN	1pjk_v	0.9324	P68400	CSNK2A1
MOL005318	RS10	REN SHEN	1l8l_v	0.9621	P78330	PSPH
MOL005318	RS10	REN SHEN	1v3q_v	0.9591	PNPH_HUMAN	PNP
MOL005318	RS10	REN SHEN	1hd2_v	0.9926	PRDX5_HUMAN	PRDX5
MOL005318	RS10	REN SHEN	1q33_v	0.9136	Q9BW91	NUDT9
MOL005318	RS10	REN SHEN	1o4n_v	0.9505	SRC_HUMAN	SRC
MOL005318	RS10	REN SHEN	1no9_v	0.947	THRB_HUMAN	F2
MOL005318	RS10	REN SHEN	1dvz_v	0.988	TTHY_HUMAN	TTR

MOL005320	RS11	REN SHEN	2oi0_v	0.9521	ADA17_HUMAN	ADAM17
MOL005320	RS11	REN SHEN	1j96_v	0.9939	AK1C2_HUMAN	AKR1C2
MOL005320	RS11	REN SHEN	1xf0_v	0.9402	AK1C3_HUMAN	AKR1C3
MOL005320	RS11	REN SHEN	1e7a_v	0.9814	ALBU_HUMAN	ALB
MOL005320	RS11	REN SHEN	2fzb_v	0.9664	ALDR_HUMAN	AKR1B1
MOL005320	RS11	REN SHEN	1xj7_v	0.9688	ANDR_HUMAN	AR
MOL005320	RS11	REN SHEN	1oj9_v	0.99	AOFB_HUMAN	MAOB
MOL005320	RS11	REN SHEN	1l6l_v	0.9777	APOA2_HUMAN	APOA2
MOL005320	RS11	REN SHEN	2q11_v	0.9218	BACE1_HUMAN	BACE1
MOL005320	RS11	REN SHEN	3bmp_v	0.9997	BMP2_HUMAN	BMP2
MOL005320	RS11	REN SHEN	2nmx_v	0.9746	CAH1_HUMAN	CA1
MOL005320	RS11	REN SHEN	1oq5_v	0.9903	CAH2_HUMAN	CA2
MOL005320	RS11	REN SHEN	3dej_v	0.9549	CASP3_HUMAN	CASP3
MOL005320	RS11	REN SHEN	1shj_v	0.9824	CASP7_HUMAN	CASP7
MOL005320	RS11	REN SHEN	1h1p_v	0.9718	CCNA2_HUMAN	CCNA2
MOL005320	RS11	REN SHEN	2cgw_v	0.9475	CHK1_HUMAN	CHEK1
MOL005320	RS11	REN SHEN	1xlu_v	0.9417	CHLE_HUMAN	BCHE
MOL005320	RS11	REN SHEN	1yb1_v	0.9801	DHB11_HUMAN	HSD17B11
MOL005320	RS11	REN SHEN	2vwu_v	0.9577	EPHB4_HUMAN	EPHB4
MOL005320	RS11	REN SHEN	1tow_v	0.9011	FABP4_HUMAN	FABP4
MOL005320	RS11	REN SHEN	1f40_v	0.9312	FKB1A_HUMAN	FKBP1A
MOL005320	RS11	REN SHEN	1lbk_v	0.9853	GSTP1_HUMAN	GSTP1
MOL005320	RS11	REN SHEN	2fky_v	0.9722	KIF11_HUMAN	KIF11
MOL005320	RS11	REN SHEN	1pme_v	0.9965	MK01_HUMAN	MAPK1
MOL005320	RS11	REN SHEN	2gmx_v	0.945	MK08_HUMAN	MAPK8
MOL005320	RS11	REN SHEN	3cgf_v	0.9932	MK10_HUMAN	MAPK10
MOL005320	RS11	REN SHEN	1eub_v	0.9787	MMP13_HUMAN	MMP13
MOL005320	RS11	REN SHEN	1qqs_v	0.9588	NGAL_HUMAN	LCN2
MOL005320	RS11	REN SHEN	1xv9_v	0.9303	NR1I3_HUMAN	NR1I3
MOL005320	RS11	REN SHEN	2pin_v	0.9717	P10828	THRB
MOL005320	RS11	REN SHEN	3eqm_v	0.9465	P11511	CYP19A1
MOL005320	RS11	REN SHEN	1e1x_v	0.9828	P24941	CDK2
MOL005320	RS11	REN SHEN	3cjf_v	0.9932	P35968	KDR
MOL005320	RS11	REN SHEN	2jbo_v	0.9868	P49137	[illegible]APKAPK2
MOL005320	RS11	REN SHEN	1oni_v	0.9663	P52758	RIDA
MOL005320	RS11	REN SHEN	1w8l_v	0.9691	P62937	PPIA
MOL005320	RS11	REN SHEN	2o65_v	0.9753	PIM1_HUMAN	PIM1
MOL005320	RS11	REN SHEN	1zgy_v	0.9699	PPARG_HUMAN	PPARG
MOL005320	RS11	REN SHEN	1hd2_v	0.9631	PRDX5_HUMAN	PRDX5
MOL005320	RS11	REN SHEN	2ovm_v	0.9203	PRGR_HUMAN	PGR
MOL005320	RS11	REN SHEN	2f6t_v	0.9938	PTN1_HUMAN	PTPN1
MOL005320	RS11	REN SHEN	1bl6_v	0.9107	Q16539	MAPK14

MOL005320	RS11	REN SHEN	1p49_v	0.9813	STS_HUMAN	STS
MOL005320	RS11	REN SHEN	2roy_v	0.9878	TTHY_HUMAN	TTR
MOL005320	RS11	REN SHEN	1j78_v	0.923	VTDB_HUMAN	GC
MOL005321	RS12	REN SHEN	1uki_v	0.9425	MK08_HUMAN	MAPK8
MOL005321	RS12	REN SHEN	1pmv_v	0.95	MK10_HUMAN	MAPK10
MOL005321	RS12	REN SHEN	1w8m_v	0.9326	P62937	PPIA
MOL005321	RS12	REN SHEN	1q8m_v	0.9261	Q9NP99	TREM1
MOL005344	RS13	REN SHEN	1j96_v	0.9832	AK1C2_HUMAN	AKR1C2
MOL005344	RS13	REN SHEN	1xf0_v	0.9132	AK1C3_HUMAN	AKR1C3
MOL005344	RS13	REN SHEN	1e7e_v	0.9908	ALBU_HUMAN	ALB
MOL005344	RS13	REN SHEN	1x97_v	0.9407	ALDR_HUMAN	AKR1B1
MOL005344	RS13	REN SHEN	2bk5_v	0.9626	AOFB_HUMAN	MAOB
MOL005344	RS13	REN SHEN	1l6l_v	0.945	APOA2_HUMAN	APOA2
MOL005344	RS13	REN SHEN	2ohs_v	0.9702	BACE1_HUMAN	BACE1
MOL005344	RS13	REN SHEN	1reu_v	0.9954	BMP2_HUMAN	BMP2
MOL005344	RS13	REN SHEN	1if4_v	0.9753	CAH2_HUMAN	CA2
MOL005344	RS13	REN SHEN	1gfw_v	0.9566	CASP3_HUMAN	CASP3
MOL005344	RS13	REN SHEN	1shj_v	0.9679	CASP7_HUMAN	CASP7
MOL005344	RS13	REN SHEN	1vyw_v	0.9898	CCNA2_HUMAN	CCNA2
MOL005344	RS13	REN SHEN	1rs0_v	0.9473	CFAB_HUMAN	CFB
MOL005344	RS13	REN SHEN	2brb_v	0.9648	CHK1_HUMAN	CHEK1
MOL005344	RS13	REN SHEN	1xlv_v	0.9657	CHLE_HUMAN	BCHE
MOL005344	RS13	REN SHEN	1vec_v	0.9555	DDX6_HUMAN	DDX6
MOL005344	RS13	REN SHEN	1yb1_v	0.9726	DHB11_HUMAN	HSD17B11
MOL005344	RS13	REN SHEN	1lbk_v	0.9905	GSTP1_HUMAN	GSTP1
MOL005344	RS13	REN SHEN	2hck_v	0.9673	HCK_HUMAN	HCK
MOL005344	RS13	REN SHEN	2pg2_v	0.9781	KIF11_HUMAN	KIF11
MOL005344	RS13	REN SHEN	2gmx_v	0.9397	MK08_HUMAN	MAPK8
MOL005344	RS13	REN SHEN	1pmv_v	0.9928	MK10_HUMAN	MAPK10
MOL005344	RS13	REN SHEN	1bm6_v	0.9228	MMP3_HUMAN	MMP3
MOL005344	RS13	REN SHEN	1pq9_v	0.9519	NR1H2_HUMAN	NR1H2
MOL005344	RS13	REN SHEN	3eko_v	0.9461	P07900	HSP90AA1
MOL005344	RS13	REN SHEN	2pin_v	0.9213	P10828	THRB
MOL005344	RS13	REN SHEN	2obj_v	0.9296	P11309	PIM1
MOL005344	RS13	REN SHEN	2jbp_v	0.9315	P49137	MAPKAPK2
MOL005344	RS13	REN SHEN	1w8l_v	0.9778	P62937	PPIA
MOL005344	RS13	REN SHEN	1a28_v	0.9701	PRGR_HUMAN	PGR
MOL005344	RS13	REN SHEN	1cb0_v	0.9311	Q13126	MTAP
MOL005344	RS13	REN SHEN	1q33_v	0.9524	Q9BW91	NUDT9
MOL005344	RS13	REN SHEN	1p49_v	1	STS_HUMAN	STS
MOL005344	RS13	REN SHEN	1f86_v	0.9499	TTHY_HUMAN	TTR
MOL005348	RS14	REN SHEN	1j96_v	1	AK1C2_HUMAN	AKR1C2

MOL005348	RS14	REN SHEN	1e7e_v	1	ALBU_HUMAN	ALB
MOL005348	RS14	REN SHEN	1t63_v	0.9516	ANDR_HUMAN	AR
MOL005348	RS14	REN SHEN	1oj9_v	1	AOFB_HUMAN	MAOB
MOL005348	RS14	REN SHEN	1l6l_v	1	APOA2_HUMAN	APOA2
MOL005348	RS14	REN SHEN	2ohp_v	0.9765	BACE1_HUMAN	BACE1
MOL005348	RS14	REN SHEN	1reu_v	1	BMP2_HUMAN	BMP2
MOL005348	RS14	REN SHEN	1okl_v	0.978	CAH2_HUMAN	CA2
MOL005348	RS14	REN SHEN	3dej_v	0.9062	CASP3_HUMAN	CASP3
MOL005348	RS14	REN SHEN	1shj_v	0.9654	CASP7_HUMAN	CASP7
MOL005348	RS14	REN SHEN	1lyw_v	0.9105	CATD_HUMAN	CTSD
MOL005348	RS14	REN SHEN	1rs0_v	0.9793	CFAB_HUMAN	CFB
MOL005348	RS14	REN SHEN	1tg6_v	0.9777	CLPP_HUMAN	CLPP
MOL005348	RS14	REN SHEN	1xdd_v	0.937	ITAL_HUMAN	ITGAL
MOL005348	RS14	REN SHEN	2pg2_v	1	KIF11_HUMAN	KIF11
MOL005348	RS14	REN SHEN	1pme_v	0.98	MK01_HUMAN	MAPK1
MOL005348	RS14	REN SHEN	1pmv_v	0.9965	MK10_HUMAN	MAPK10
MOL005348	RS14	REN SHEN	1eub_v	0.9534	MMP13_HUMAN	MMP13
MOL005348	RS14	REN SHEN	1bm6_v	0.9522	MMP3_HUMAN	MMP3
MOL005348	RS14	REN SHEN	2pin_v	1	P10828	THRB
MOL005348	RS14	REN SHEN	2p3g_v	0.9282	P49137	MAPKAPK2
MOL005348	RS14	REN SHEN	2pe0_v	0.9195	PDPK1_HUMAN	PDPK1
MOL005348	RS14	REN SHEN	2o65_v	1	PIM1_HUMAN	PIM1
MOL005348	RS14	REN SHEN	1v3q_v	0.9161	PNPH_HUMAN	PNP
MOL005348	RS14	REN SHEN	2acl_v	0.9234	RXRA_HUMAN	RXRA
MOL005348	RS14	REN SHEN	1p49_v	1	STS_HUMAN	STS
MOL005348	RS14	REN SHEN	1ijn_v	0.9533	TTHY_HUMAN	TTR
MOL005348	RS14	REN SHEN	1j78_v	0.978	VTDB_HUMAN	GC
MOL005356	RS15	REN SHEN	1j96_v	0.9616	AK1C2_HUMAN	AKR1C2
MOL005356	RS15	REN SHEN	1e7a_v	0.9166	ALBU_HUMAN	ALB
MOL005356	RS15	REN SHEN	2ohp_v	0.9546	BACE1_HUMAN	BACE1
MOL005356	RS15	REN SHEN	1reu_v	0.9886	BMP2_HUMAN	BMP2
MOL005356	RS15	REN SHEN	1pme_v	0.9559	MK01_HUMAN	MAPK1
MOL005356	RS15	REN SHEN	1eub_v	0.9577	MMP13_HUMAN	MMP13
MOL005356	RS15	REN SHEN	3eqm_v	0.9021	P11511	CYP19A1
MOL005356	RS15	REN SHEN	2p3g_v	0.979	P49137	MAPKAPK2
MOL005356	RS15	REN SHEN	2o65_v	0.9604	PIM1_HUMAN	PIM1
MOL005356	RS15	REN SHEN	1p49_v	0.9251	STS_HUMAN	STS
MOL005357	RS16	REN SHEN	1e7a_v	0.9174	ALBU_HUMAN	ALB
MOL005357	RS16	REN SHEN	1reu_v	0.9587	BMP2_HUMAN	BMP2
MOL005357	RS16	REN SHEN	1p0i_v	0.9265	CHLE_HUMAN	BCHE
MOL005357	RS16	REN SHEN	1lbk_v	0.9646	GSTP1_HUMAN	GSTP1
MOL005357	RS16	REN SHEN	1uki_v	0.9595	MK08_HUMAN	MAPK8

MOL005357	RS16	REN SHEN	1pmv_v	0.9921	MK10_HUMAN	MAPK10
MOL005357	RS16	REN SHEN	1eub_v	0.954	MMP13_HUMAN	MMP13
MOL005357	RS16	REN SHEN	1bm6_v	0.958	MMP3_HUMAN	MMP3
MOL005357	RS16	REN SHEN	2pin_v	0.917	P10828	THRB
MOL005357	RS16	REN SHEN	3gam_v	0.97	P16083	NQO2
MOL005357	RS16	REN SHEN	1w8l_v	0.942	P62937	PPIA
MOL005357	RS16	REN SHEN	2o65_v	0.918	PIM1_HUMAN	PIM1
MOL005357	RS16	REN SHEN	1v3q_v	0.9246	PNPH_HUMAN	PNP
MOL005357	RS16	REN SHEN	1dvz_v	0.9355	TTHY_HUMAN	TTR
MOL005360	RS17	REN SHEN	1j96_v	1	AK1C2_HUMAN	AKR1C2
MOL005360	RS17	REN SHEN	1e7a_v	1	ALBU_HUMAN	ALB
MOL005360	RS17	REN SHEN	1az1_v	0.917	ALDR_HUMAN	AKR1B1
MOL005360	RS17	REN SHEN	2pit_v	0.9096	ANDR_HUMAN	AR
MOL005360	RS17	REN SHEN	1hak_v	0.9034	ANXA5_HUMAN	ANXA5
MOL005360	RS17	REN SHEN	1oj9_v	1	AOFB_HUMAN	MAOB
MOL005360	RS17	REN SHEN	2q11_v	0.996	BACE1_HUMAN	BACE1
MOL005360	RS17	REN SHEN	1reu_v	1	BMP2_HUMAN	BMP2
MOL005360	RS17	REN SHEN	1czm_v	0.9827	CAH1_HUMAN	CA1
MOL005360	RS17	REN SHEN	1jd0_v	0.9437	CAH12_HUMAN	CA12
MOL005360	RS17	REN SHEN	1okl_v	0.9889	CAH2_HUMAN	CA2
MOL005360	RS17	REN SHEN	1rs0_v	0.9501	CFAB_HUMAN	CFB
MOL005360	RS17	REN SHEN	1dic_v	0.9511	CFAD_HUMAN	CFD
MOL005360	RS17	REN SHEN	2brg_v	0.9567	CHK1_HUMAN	CHEK1
MOL005360	RS17	REN SHEN	1xlv_v	0.9662	CHLE_HUMAN	BCHE
MOL005360	RS17	REN SHEN	1vec_v	0.907	DDX6_HUMAN	DDX6
MOL005360	RS17	REN SHEN	2vwu_v	0.9514	EPHB4_HUMAN	EPHB4
MOL005360	RS17	REN SHEN	1md3_v	0.97	GSTP1_HUMAN	GSTP1
MOL005360	RS17	REN SHEN	2pg2_v	1	KIF11_HUMAN	KIF11
MOL005360	RS17	REN SHEN	2h96_v	0.9576	MK08_HUMAN	MAPK8
MOL005360	RS17	REN SHEN	1pmv_v	0.9691	MK10_HUMAN	MAPK10
MOL005360	RS17	REN SHEN	1pq9_v	0.9262	NR1H2_HUMAN	NR1H2
MOL005360	RS17	REN SHEN	1le6_v	0.9317	O15496	PLA2G10
MOL005360	RS17	REN SHEN	3bgq_v	0.9718	P11309	PIM1
MOL005360	RS17	REN SHEN	2jbp_v	0.9853	P49137	MAPKAPK2
MOL005360	RS17	REN SHEN	1w8l_v	0.9772	P62937	PPIA
MOL005360	RS17	REN SHEN	1xlz_v	0.9177	PDE4B_HUMAN	PDE4B
MOL005360	RS17	REN SHEN	2pe0_v	0.9421	PDPK1_HUMAN	PDPK1
MOL005360	RS17	REN SHEN	2a4z_v	0.9356	PK3CG_HUMAN	PIK3CG
MOL005360	RS17	REN SHEN	1a28_v	0.9379	PRGR_HUMAN	PGR
MOL005360	RS17	REN SHEN	1j99_v	0.913	Q06520	SULT2A1
MOL005360	RS17	REN SHEN	1w84_v	0.9462	Q16539	MAPK14
MOL005360	RS17	REN SHEN	2afu_v	0.9633	QPCT_HUMAN	QPCT

MOL005360	RS17	REN SHEN	1p49_v	1	STS_HUMAN	STS
MOL005360	RS17	REN SHEN	1vjy_v	0.9394	TGFR1_HUMAN	TGFBR1
MOL005360	RS17	REN SHEN	1no9_v	0.9811	THRB_HUMAN	F2
MOL005360	RS17	REN SHEN	1dvz_v	0.9821	TTHY_HUMAN	TTR
MOL005360	RS17	REN SHEN	1j78_v	0.9716	VTDB_HUMAN	GC
MOL005376	RS18	REN SHEN	1j96_v	0.9988	AK1C2_HUMAN	AKR1C2
MOL005376	RS18	REN SHEN	1e7a_v	1	ALBU_HUMAN	ALB
MOL005376	RS18	REN SHEN	2pir_v	0.9607	ANDR_HUMAN	AR
MOL005376	RS18	REN SHEN	1oj9_v	0.9635	AOFB_HUMAN	MAOB
MOL005376	RS18	REN SHEN	1reu_v	1	BMP2_HUMAN	BMP2
MOL005376	RS18	REN SHEN	1okl_v	0.9535	CAH2_HUMAN	CA2
MOL005376	RS18	REN SHEN	1shj_v	0.9874	CASP7_HUMAN	CASP7
MOL005376	RS18	REN SHEN	1rs0_v	0.9404	CFAB_HUMAN	CFB
MOL005376	RS18	REN SHEN	1ya8_v	0.9106	EST1_HUMAN	CES1
MOL005376	RS18	REN SHEN	2pg2_v	0.9536	KIF11_HUMAN	KIF11
MOL005376	RS18	REN SHEN	1pme_v	0.9502	MK01_HUMAN	MAPK1
MOL005376	RS18	REN SHEN	3cgf_v	0.9633	MK10_HUMAN	MAPK10
MOL005376	RS18	REN SHEN	1eub_v	0.9801	MMP13_HUMAN	MMP13
MOL005376	RS18	REN SHEN	3bgp_v	0.9822	P11309	PIM1
MOL005376	RS18	REN SHEN	3cjf_v	0.9633	P35968	KDR
MOL005376	RS18	REN SHEN	2p3g_v	0.9894	P49137	MAPKAPK2
MOL005376	RS18	REN SHEN	3exo_v	0.9554	P56817	BACE1
MOL005376	RS18	REN SHEN	1ov4_v	0.9123	Q06520	SULT2A1
MOL005376	RS18	REN SHEN	1n83_v	0.9303	RORA_HUMAN	RORA
MOL005376	RS18	REN SHEN	1p49_v	1	STS_HUMAN	STS
MOL005376	RS18	REN SHEN	1ijn_v	0.9196	TTHY_HUMAN	TTR
MOL005376	RS18	REN SHEN	1j78_v	0.9158	VTDB_HUMAN	GC
MOL005401	RS19	REN SHEN	1j96_v	0.9928	AK1C2_HUMAN	AKR1C2
MOL005401	RS19	REN SHEN	1e7a_v	0.9926	ALBU_HUMAN	ALB
MOL005401	RS19	REN SHEN	2agt_v	0.9537	ALDR_HUMAN	AKR1B1
MOL005401	RS19	REN SHEN	2q7j_v	0.9831	ANDR_HUMAN	AR
MOL005401	RS19	REN SHEN	1oj9_v	0.9871	AOFB_HUMAN	MAOB
MOL005401	RS19	REN SHEN	1l6l_v	1	APOA2_HUMAN	APOA2
MOL005401	RS19	REN SHEN	1reu_v	1	BMP2_HUMAN	BMP2
MOL005401	RS19	REN SHEN	1okl_v	0.9864	CAH2_HUMAN	CA2
MOL005401	RS19	REN SHEN	3dej_v	0.9711	CASP3_HUMAN	CASP3
MOL005401	RS19	REN SHEN	1shj_v	0.9818	CASP7_HUMAN	CASP7
MOL005401	RS19	REN SHEN	1vyw_v	0.9384	CCNA2_HUMAN	CCNA2
MOL005401	RS19	REN SHEN	1rs0_v	0.9801	CFAB_HUMAN	CFB
MOL005401	RS19	REN SHEN	1tg6_v	0.9561	CLPP_HUMAN	CLPP
MOL005401	RS19	REN SHEN	1yb1_v	0.9191	DHB11_HUMAN	HSD17B11
MOL005401	RS19	REN SHEN	1xdd_v	0.9591	ITAL_HUMAN	ITGAL

MOL005401	RS19	REN SHEN	2pg2_v	0.991	KIF11_HUMAN	KIF11
MOL005401	RS19	REN SHEN	1pme_v	0.9617	MK01_HUMAN	MAPK1
MOL005401	RS19	REN SHEN	1pmv_v	0.9941	MK10_HUMAN	MAPK10
MOL005401	RS19	REN SHEN	1eub_v	0.9207	MMP13_HUMAN	MMP13
MOL005401	RS19	REN SHEN	2pin_v	0.9538	P10828	THRB
MOL005401	RS19	REN SHEN	3bgp_v	0.9781	P11309	PIM1
MOL005401	RS19	REN SHEN	1jvp_v	0.9025	P24941	CDK2
MOL005401	RS19	REN SHEN	2jbp_v	0.9853	P49137	MAPKAPK2
MOL005401	RS19	REN SHEN	1w8l_v	0.9853	P62937	PPIA
MOL005401	RS19	REN SHEN	1v3q_v	0.9689	PNPH_HUMAN	PNP
MOL005401	RS19	REN SHEN	1zgy_v	0.9635	PPARG_HUMAN	PPARG
MOL005401	RS19	REN SHEN	1a28_v	0.9544	PRGR_HUMAN	PGR
MOL005401	RS19	REN SHEN	1w7h_v	0.9138	Q16539	MAPK14
MOL005401	RS19	REN SHEN	1p49_v	1	STS_HUMAN	STS
MOL005401	RS19	REN SHEN	1no9_v	0.952	THRB_HUMAN	F2
MOL005401	RS19	REN SHEN	1dvz_v	0.9624	TTHY_HUMAN	TTR
MOL005401	RS19	REN SHEN	1j78_v	0.9572	VTDB_HUMAN	GC
MOL000449	RS2	REN SHEN	1j96_v	0.999	AK1C2_HUMAN	AKR1C2
MOL000449	RS2	REN SHEN	1e7e_v	0.9871	ALBU_HUMAN	ALB
MOL000449	RS2	REN SHEN	1t63_v	0.9069	ANDR_HUMAN	AR
MOL000449	RS2	REN SHEN	1oj9_v	0.9863	AOFB_HUMAN	MAOB
MOL000449	RS2	REN SHEN	1l6l_v	0.9804	APOA2_HUMAN	APOA2
MOL000449	RS2	REN SHEN	1reu_v	0.9914	BMP2_HUMAN	BMP2
MOL000449	RS2	REN SHEN	1if4_v	0.9678	CAH2_HUMAN	CA2
MOL000449	RS2	REN SHEN	1shj_v	0.968	CASP7_HUMAN	CASP7
MOL000449	RS2	REN SHEN	1yb1_v	0.9432	DHB11_HUMAN	HSD17B11
MOL000449	RS2	REN SHEN	2pg2_v	0.9585	KIF11_HUMAN	KIF11
MOL000449	RS2	REN SHEN	2pin_v	0.9721	P10828	THRB
MOL000449	RS2	REN SHEN	3bgp_v	0.989	P11309	PIM1
MOL000449	RS2	REN SHEN	2jbp_v	0.9934	P49137	MAPKAPK2
MOL000449	RS2	REN SHEN	1n83_v	0.9599	RORA_HUMAN	RORA
MOL000449	RS2	REN SHEN	1p49_v	0.9888	STS_HUMAN	STS
MOL000449	RS2	REN SHEN	1j78_v	0.9045	VTDB_HUMAN	GC
MOL005384	RS20	REN SHEN	2pir_v	0.928	ANDR_HUMAN	AR
MOL005384	RS20	REN SHEN	1lyw_v	0.9134	CATD_HUMAN	CTSD
MOL005384	RS20	REN SHEN	1unh_v	0.9909	CD5R1_HUMAN	CDK5R1
MOL005384	RS20	REN SHEN	1rs0_v	0.9375	CFAB_HUMAN	CFB
MOL005384	RS20	REN SHEN	2brb_v	0.9541	CHK1_HUMAN	CHEK1
MOL005384	RS20	REN SHEN	1vec_v	0.9609	DDX6_HUMAN	DDX6
MOL005384	RS20	REN SHEN	2ocf_v	0.9689	ESR1_HUMAN	ESR1
MOL005384	RS20	REN SHEN	2jj3_v	0.9868	ESR2_HUMAN	ESR2
MOL005384	RS20	REN SHEN	1lbk_v	0.9679	GSTP1_HUMAN	GSTP1

MOL005384	RS20	REN SHEN	2pg2_v	0.9675	KIF11_HUMAN	KIF11
MOL005384	RS20	REN SHEN	1pme_v	0.9678	MK01_HUMAN	MAPK1
MOL005384	RS20	REN SHEN	1uki_v	0.911	MK08_HUMAN	MAPK8
MOL005384	RS20	REN SHEN	1pmv_v	0.9149	MK10_HUMAN	MAPK10
MOL005384	RS20	REN SHEN	830c_v	0.9184	MMP13_HUMAN	MMP13
MOL005384	RS20	REN SHEN	3dbu_v	0.9726	P00918	CA2
MOL005384	RS20	REN SHEN	1w8l_v	0.9899	P62937	PPIA
MOL005384	RS20	REN SHEN	1yxx_v	0.9287	PIM1_HUMAN	PIM1
MOL005384	RS20	REN SHEN	1v3q_v	0.9853	PNPH_HUMAN	PNP
MOL005384	RS20	REN SHEN	1no9_v	0.9855	THRB_HUMAN	F2
MOL005399	RS5	REN SHEN	1j96_v	0.9974	AK1C2_HUMAN	AKR1C2
MOL005399	RS5	REN SHEN	1e7e_v	0.976	ALBU_HUMAN	ALB
MOL005399	RS5	REN SHEN	1oj9_v	0.998	AOFB_HUMAN	MAOB
MOL005399	RS5	REN SHEN	1l6l_v	0.9473	APOA2_HUMAN	APOA2
MOL005399	RS5	REN SHEN	3bmp_v	0.9974	BMP2_HUMAN	BMP2
MOL005399	RS5	REN SHEN	1okl_v	0.9556	CAH2_HUMAN	CA2
MOL005399	RS5	REN SHEN	1shj_v	0.9667	CASP7_HUMAN	CASP7
MOL005399	RS5	REN SHEN	1xdd_v	0.916	ITAL_HUMAN	ITGAL
MOL005399	RS5	REN SHEN	2pg2_v	0.94	KIF11_HUMAN	KIF11
MOL005399	RS5	REN SHEN	1pme_v	0.9093	MK01_HUMAN	MAPK1
MOL005399	RS5	REN SHEN	2pin_v	0.9912	P10828	THRB
MOL005399	RS5	REN SHEN	3bgp_v	0.9754	P11309	PIM1
MOL005399	RS5	REN SHEN	2jbp_v	0.9856	P49137	APKAPK2
MOL005399	RS5	REN SHEN	1p49_v	0.9733	STS_HUMAN	STS
MOL005399	RS5	REN SHEN	1j78_v	0.9647	VTDB_HUMAN	GC
MOL000787	RS3	REN SHEN	1rs0_v	0.9187	CFAB_HUMAN	CFB
MOL000787	RS3	REN SHEN	1p0i_v	0.9305	CHLE_HUMAN	BCHE
MOL000787	RS3	REN SHEN	1pmv_v	0.9438	MK10_HUMAN	MAPK10
MOL000787	RS3	REN SHEN	1hpk_v	0.9526	P00747	PLG
MOL000787	RS3	REN SHEN	1dvz_v	0.9306	TTHY_HUMAN	TTR
MOL003648	RS4	REN SHEN	1e7e_v	0.9577	ALBU_HUMAN	ALB
MOL003648	RS4	REN SHEN	2pir_v	0.9462	ANDR_HUMAN	AR
MOL003648	RS4	REN SHEN	1l6l_v	0.9796	APOA2_HUMAN	APOA2
MOL003648	RS4	REN SHEN	1reu_v	0.971	BMP2_HUMAN	BMP2
MOL003648	RS4	REN SHEN	1rs0_v	0.9792	CFAB_HUMAN	CFB
MOL003648	RS4	REN SHEN	1vec_v	0.9839	DDX6_HUMAN	DDX6
MOL003648	RS4	REN SHEN	2ocf_v	0.9618	ESR1_HUMAN	ESR1
MOL003648	RS4	REN SHEN	1md3_v	0.9717	GSTP1_HUMAN	GSTP1
MOL003648	RS4	REN SHEN	1uki_v	0.92	MK08_HUMAN	MAPK8
MOL003648	RS4	REN SHEN	1pmv_v	0.9095	MK10_HUMAN	MAPK10
MOL003648	RS4	REN SHEN	3dcw_v	0.9858	P00918	CA2
MOL003648	RS4	REN SHEN	2obj_v	0.9681	P11309	PIM1

MOL003648	RS4	REN SHEN	1p49_v	1	STS_HUMAN	STS
MOL004492	RS6	REN SHEN	1j96_v	0.9963	AK1C2_HUMAN	AKR1C2
MOL004492	RS6	REN SHEN	1e7e_v	0.9965	ALBU_HUMAN	ALB
MOL004492	RS6	REN SHEN	2ama_v	0.9314	ANDR_HUMAN	AR
MOL004492	RS6	REN SHEN	1hak_v	0.9469	ANXA5_HUMAN	ANXA5
MOL004492	RS6	REN SHEN	1oj9_v	0.9948	AOFB_HUMAN	MAOB
MOL004492	RS6	REN SHEN	1l6l_v	1	APOA2_HUMAN	APOA2
MOL004492	RS6	REN SHEN	2q11_v	0.9371	BACE1_HUMAN	BACE1
MOL004492	RS6	REN SHEN	3bmp_v	0.997	BMP2_HUMAN	BMP2
MOL004492	RS6	REN SHEN	1okl_v	0.9839	CAH2_HUMAN	CA2
MOL004492	RS6	REN SHEN	3dej_v	0.9931	CASP3_HUMAN	CASP3
MOL004492	RS6	REN SHEN	1shj_v	0.9873	CASP7_HUMAN	CASP7
MOL004492	RS6	REN SHEN	1unh_v	0.9182	CD5R1_HUMAN	CDK5R1
MOL004492	RS6	REN SHEN	1rs0_v	0.9161	CFAB_HUMAN	CFB
MOL004492	RS6	REN SHEN	2brb_v	0.9229	CHK1_HUMAN	CHEK1
MOL004492	RS6	REN SHEN	1yb1_v	0.9948	DHB11_HUMAN	HSD17B11
MOL004492	RS6	REN SHEN	1ya8_v	0.9271	EST1_HUMAN	CES1
MOL004492	RS6	REN SHEN	2j95_v	0.9006	FA10_HUMAN	F10
MOL004492	RS6	REN SHEN	1f40_v	0.9794	FKB1A_HUMAN	FKBP1A
MOL004492	RS6	REN SHEN	1xdd_v	0.996	ITAL_HUMAN	ITGAL
MOL004492	RS6	REN SHEN	2pg2_v	0.9976	KIF11_HUMAN	KIF11
MOL004492	RS6	REN SHEN	1pme_v	0.9785	MK01_HUMAN	MAPK1
MOL004492	RS6	REN SHEN	2gmx_v	0.9561	MK08_HUMAN	MAPK8
MOL004492	RS6	REN SHEN	1pmv_v	0.9631	MK10_HUMAN	MAPK10
MOL004492	RS6	REN SHEN	1eub_v	0.9987	MMP13_HUMAN	MMP13
MOL004492	RS6	REN SHEN	1bm6_v	0.933	MMP3_HUMAN	MMP3
MOL004492	RS6	REN SHEN	2pin_v	0.9977	P10828	THRB
MOL004492	RS6	REN SHEN	3bgp_v	0.9996	P11309	PIM1
MOL004492	RS6	REN SHEN	3eqm_v	0.9734	P11511	CYP19A1
MOL004492	RS6	REN SHEN	2jbp_v	0.9934	P49137	/IAPKAPK2
MOL004492	RS6	REN SHEN	1w8l_v	0.9596	P62937	PPIA
MOL004492	RS6	REN SHEN	1ih0_v	0.9135	P63316	TNNC1
MOL004492	RS6	REN SHEN	1zgy_v	0.9692	PPARG_HUMAN	PPARG
MOL004492	RS6	REN SHEN	1a28_v	0.9274	PRGR_HUMAN	PGR
MOL004492	RS6	REN SHEN	1n83_v	0.9332	RORA_HUMAN	RORA
MOL004492	RS6	REN SHEN	1p49_v	1	STS_HUMAN	STS
MOL004492	RS6	REN SHEN	1no9_v	0.9822	THRB_HUMAN	F2
MOL004492	RS6	REN SHEN	1f86_v	0.9982	TTHY_HUMAN	TTR
MOL004492	RS6	REN SHEN	1j78_v	1	VTDB_HUMAN	GC
MOL004492	RS6	REN SHEN	1t84_v	0.919	WASP_HUMAN	WAS
MOL005308	RS7	REN SHEN	1klt_v	0.9468	CMA1_HUMAN	CMA1
MOL005308	RS7	REN SHEN	2g63_v	0.9722	DPP4_HUMAN	DPP4

MOL005308	RS7	REN SHEN	1pq9_v	0.9471	NR1H2_HUMAN	NR1H2
MOL005308	RS7	REN SHEN	1qmv_v	0.9396	P32119	PRDX2
MOL005308	RS7	REN SHEN	1w8m_v	0.9435	P62937	PPIA
MOL005314	RS8	REN SHEN	1uki_v	0.9426	MK08_HUMAN	MAPK8
MOL005314	RS8	REN SHEN	1pmv_v	0.949	MK10_HUMAN	MAPK10
MOL005317	RS9	REN SHEN	1if4_v	0.9455	CAH2_HUMAN	CA2
MOL005317	RS9	REN SHEN	1lyw_v	0.9398	CATD_HUMAN	CTSD
MOL005317	RS9	REN SHEN	1rs0_v	0.9771	CFAB_HUMAN	CFB
MOL005317	RS9	REN SHEN	1p0i_v	0.9421	CHLE_HUMAN	BCHE
MOL005317	RS9	REN SHEN	1klt_v	0.9208	CMA1_HUMAN	CMA1
MOL005317	RS9	REN SHEN	1vec_v	0.9736	DDX6_HUMAN	DDX6
MOL005317	RS9	REN SHEN	2onc_v	0.9517	DPP4_HUMAN	DPP4
MOL005317	RS9	REN SHEN	1xkb_v	0.9747	FA10_HUMAN	F10
MOL005317	RS9	REN SHEN	1ogs_v	0.9686	GLCM_HUMAN	GBA
MOL005317	RS9	REN SHEN	1md3_v	0.9813	GSTP1_HUMAN	GSTP1
MOL005317	RS9	REN SHEN	1g1q_v	0.9778	LYAM3_HUMAN	SELP
MOL005317	RS9	REN SHEN	1pmv_v	0.996	MK10_HUMAN	MAPK10
MOL005317	RS9	REN SHEN	1bm6_v	0.9793	MMP3_HUMAN	MMP3
MOL005317	RS9	REN SHEN	1pq9_v	0.9575	NR1H2_HUMAN	NR1H2
MOL005317	RS9	REN SHEN	1smd_v	0.9343	P04745	AMY1A
MOL005317	RS9	REN SHEN	1w8m_v	0.9762	P62937	PPIA
MOL005317	RS9	REN SHEN	2c3i_v	0.9435	PIM1_HUMAN	PIM1
MOL005317	RS9	REN SHEN	1v3q_v	0.981	PNPH_HUMAN	PNP
MOL005317	RS9	REN SHEN	1q8m_v	0.9628	Q9NP99	TREM1
MOL005317	RS9	REN SHEN	1nub_v	0.9191	SPRC_HUMAN	SPARC
MOL005317	RS9	REN SHEN	1no9_v	0.9686	THRB_HUMAN	F2
MOL005317	RS9	REN SHEN	1a0l_v	0.9287	TRYB2_HUMAN	TPSB2

Detailed Information of 147 Genes

Source	UNIPORT	Genename
BZ2	MK08_HUMAN	MAPK8
BZ2	FKB1A_HUMAN	FKBP1A
BZ2	CHLE_HUMAN	BCHE
BZ3	MMP3_HUMAN	MMP3
BZ3	P35968	KDR
BZ3	Q9NP99	TREM1
BZ4	ITAL_HUMAN	ITGAL
FZ1	NR1I3_HUMAN	NR1I3
FZ11	KIF11_HUMAN	KIF11
FZ11	AOFB_HUMAN	MAOB
FZ11	MMP13_HUMAN	MMP13
FZ11	P49137	/APKAPK2

FZ11	PIM1_HUMAN	PIM1
FZ11	THRB_HUMAN	F2
FZ11	PDE5A_HUMAN	PDE5A
FZ11	PNMT_HUMAN	PNMT
FZ12	P32119	PRDX2
FZ12	ADH1B_HUMAN	ADH1B
FZ13	P62937	PPIA
FZ13	O15496	PLA2G10
FZ13	VGFR2_HUMAN	KDR
FZ14	PH4H_HUMAN	PAH
FZ14	UROK_HUMAN	PLAU
FZ15	TTHY_HUMAN	TTR
FZ15	P10828	THRB
FZ15	CAH1_HUMAN	CA1
FZ16	PRGR_HUMAN	PGR
FZ16	DYR_HUMAN	DHFR
FZ17	O76054	SEC14L2
FZ17	P14061	HSD17B1
FZ17	Q06520	SULT2A1
FZ17	PPARG_HUMAN	PPARG
FZ17	P00747	PLG
FZ17	WASP_HUMAN	WAS
FZ17	PDE4D_HUMAN	PDE4D
FZ17	P62508	ESRRG
FZ17	SHBG_HUMAN	SHBG
FZ17	NR1H4_HUMAN	NR1H4
FZ18	ANPRC_HUMAN	NPR3
FZ19	CLPP_HUMAN	CLPP
A1	CFAD_HUMAN	CFD
FZ3	CASP3_HUMAN	CASP3
FZ4	BMP2_HUMAN	BMP2
FZ4	NGAL_HUMAN	LCN2
FZ4	GATM_HUMAN	GATM
FZ4	FABP4_HUMAN	FABP4
FZ5	CATL2_HUMAN	CTSV
FZ5	TPA_HUMAN	PLAT
FZ6	STS_HUMAN	STS
FZ6	AK1C2_HUMAN	AKR1C2
FZ6	ALBU_HUMAN	ALB
FZ7	GSTP1_HUMAN	GSTP1
FZ9	VTDB_HUMAN	GC
GC11	Q16539	MAPK14

GC12	PK3CG_HUMAN	PIK3CG
GC12	P08069	IGF1R
GC15	EST1_HUMAN	CES1
GC21	CHK1_HUMAN	CHEK1
GC21	P11142	HSPA8
GC21	NQO1_HUMAN	NQO1
GC26	CATD_HUMAN	CTSD
GC27	GLCM_HUMAN	GBA
GC28	P11309	PIM1
GC28	SPRC_HUMAN	SPARC
GC29	P56817	BACE1
GC32	Q9BW91	NUDT9
GC32	PDE4B_HUMAN	PDE4B
GC32	ANGI_HUMAN	ANG
GC32	FCAR_HUMAN	FCAR
GC40	CMA1_HUMAN	CMA1
GC40	P00390	GSR
GC43	TGFR2_HUMAN	TGFBR2
GC45	CAH2_HUMAN	CA2
GC45	Q03181	PPARD
GC49	AK1C3_HUMAN	AKR1C3
GC49	EPHB4_HUMAN	EPHB4
GC53	NR1H2_HUMAN	NR1H2
GC54	ESR2_HUMAN	ESR2
GC54	EGFR_HUMAN	EGFR
GC54	CAH12_HUMAN	CA12
GC54	RTN4R_HUMAN	RTN4R
GC54	Q13126	MTAP
GC56	ALDR_HUMAN	AKR1B1
GC56	CATS_HUMAN	CTSS
GC59	CASP7_HUMAN	CASP7
GC62	ANDR_HUMAN	AR
GC67	MK10_HUMAN	MAPK10
GC73	HS90A_HUMAN	HSP90AA1
GC74	PTN1_HUMAN	PTPN1
GC75	P11511	CYP19A1
GC77	SEPR_HUMAN	FAP
GC77	LKHA4_HUMAN	LTA4H
GC80	HCK_HUMAN	HCK
GC80	Q62986	Esr2
GC82	DHI1_HUMAN	HSD11B1
GC84	RORA_HUMAN	RORA

GC84	MK01_HUMAN	MAPK1
GC84	ESR1_HUMAN	ESR1
GC84	P24941	CDK2
GC84	PDPK1_HUMAN	PDPK1
GJ2	CFAB_HUMAN	CFB
GJ2	PNPH_HUMAN	PNP
GJ3	CDK6_HUMAN	CDK6
C1	MCR_HUMAN	NR3C2
RS1	P00918	CA2
RS1	DDX6_HUMAN	DDX6
RS1	ADA17_HUMAN	ADAM17
RS1	CATB_HUMAN	CTSB
RS10	P52758	RIDA
RS10	PRDX5_HUMAN	PRDX5
RS10	B3GA1_HUMAN	B3GAT1
RS10	P33176	KIF5B
RS10	P78330	PSPH
RS10	O15382	BCAT2
RS10	P04745	AMY1A
RS10	SRC_HUMAN	SRC
RS10	P68400	CSNK2A1
RS10	P02743	APCS
RS13	CCNA2_HUMAN	CCNA2
RS13	P07900	HSP90AA1
RS14	RXRA_HUMAN	RXRA
RS16	P16083	NQO2
RS17	BACE1_HUMAN	BACE1
RS17	QPCT_HUMAN	QPCT
RS17	TGFR1_HUMAN	TGFBR1
RS20	CD5R1_HUMAN	CDK5R1
RS6	APOA2_HUMAN	APOA2
RS6	DHB11_HUMAN	HSD17B11
RS6	ANXA5_HUMAN	ANXA5
RS6	P63316	TNNC1
RS7	DPP4_HUMAN	DPP4
RS9	LYAM3_HUMAN	SELP
RS9	FA10_HUMAN	F10
RS9	TRYB2_HUMAN	TPSB2
