# Supplementary material for: Action Mechanism Underlying Improvement Effect of Fuzi Lizhong Decoction on Nonalcoholic Fatty Liver Disease: A Study Based on Network Pharmacology and Molecular Docking
Source: Evid Based Complement Alternat Med. 2022 Jan 20;2022:1670014. doi: 10.1155/2022/1670014 (PMC8794673; doi:10.1155/2022/1670014)
Supplement: Supplementary Materials — Supplement Table 1: detailed information about the compound-target network. Supplement Table 2: information of 2,608 NAFLD-related targets. Supplement Table 3: information of 61 intersection targets between FLD and NAFLD. Supplement Table 4: detailed information about the protein-protein network. Supplement Table 5: detailed information about functional analysis. [file 1670014.f1.zip › 1670014.f1/Supplement Table 2 Information of 2,608 NAFLD-related targets.pdf]

Information of OMIM+Genecards+Disgenet	
Gene	Source
AD7CNTP	
IBD7	
PLEKHG5	
DJ1	
KIF1B	
MTHFR	
MFN2	
GBD2	
PINK1	
ECE1	
ALPL	
RHCE	
FABP3	
YARS1	
GBD3	
ELOVL1	
FAAH	
FAAHP1	
PARK10	
PAOD1	
FOXD3	
DNAJC6	
IL23R	
TNNI3K	
ABCA4	
AGL	
DBT	
ATP1A1	
AD13	
NBLST6	
NOTCH2NLC	
ECM1	
ZNF687	
SLC27A3	
MUC1	
GBA	
PKLR	

LMNA
COPA
MPZ
FMO1
FMO4
AD14
NCF2
CELIAC7
IBD23
PARK16
INAVA
REN
IL10
RMD1
TLR5
PSEN2
ARV1
ADAM17
NBAS
APOB
HADHA
MPV17
ABCG8
PARK3
HTRA2
FABP1
CELIAC8
COPD
ZAP70
LCO
CHDS2
HOXD10
PDE11A
BMPR2
CTLA4
FARSB
IRS1
SP110
GIGYF2

ATG16L1
SAG
AGXT
PDCD1
IBD9
MYMY1
CAV3
CX3CR1
CELIAC9
HSCR6
IBD12
GBE1
CHDS5
POGLUT1
SEC61A1
RAB7
AD15
PARK21
ATP2C1
DZIP1L
GYG1
CELIAC10
MME
GNB4
EIF4G1
DNAJB11
CELIAC11
HTT
HDL3
PROM1
UCHL1
PHOX2B
AIS4
PKD2
PPM1K
SNCA
ADH1C
ELOVL6
FABP2

CELIAC6
FAT4
HSCR9
TRIM2
FAT1
GDNF
IBD18
ELOVL7
HEXB
PDE8B
APC
AITD2
CELIAC2
IBD5
PDB4
LEAP2
SAR1B
HARS1
LARS1
SH3TC2
IRGM
FAT2
FABP6
SQSTM1
HULC
ELOVL2
HFE
IBD3
VAMAS6
DHX16
HLA-B
HLA-DQA1
HLA-DQB1
HLA-DPB1
AD17
FCYT
ELOVL5
AITD1
SLC17A5

COX7A2
ELOVL4
BCKDHB
SEC63
FIG4
FABP7
ARG1
ENPP1
CELIAC12
LPA
PRKN
TBP
IL6
HNRPA2B1
GARS1
SFRP4
PGAM2
EGFR
CHCHD2
NCF1
HSPB1
CD36
ABCB4
ABCB1
PON1
PON2
IBD11
ARPC1B
RINT1
IRF5
AD10
NOS3
PRKAG2
AIS2
GATA4
CHDS9
NEFL
AD12
CMT2H

JPH1
GDAP1
FABP5
PMP2
FABP4
FABP12
CDH17
YARS
WPWS
VMLDS2
VAMAS5
VAMAS3
URBWD
TIL3
TACE
SSC2
SSC1
SRK
SPDA1
SPAT
SLEB2
SIASD
SEC61
SCA17
RTD
RP47
RHNA
PUM
PROML1
PPNAD3
PPH1
PP2CM
PON
PKHD1
PK1
PGY3
PGY1
PGAMM
PDNP1

PDE11A1
PCLD2
PARK7
PARK6
PARK5
PARK22
PARK2
PAFABP
P62
OMI
NISBD2
NIID
NBPHOX
NAG
NACP
MYMY
MTPA
MTDPS6
MEGF1
LRG47
LQT9
LMN1
LCE
LARS
KIAA1985
KIAA1441
KIAA0720
KIAA0642
KIAA0517
KIAA0274
KIAA0214
JP1
ILLBP
ILFS3
IFNB2
IFI41
IDDM12
IBMPFD2
IBD17

OMIM

IBD14
HSP27
HSCR3
HOX4D
HOPS
HLN2
HLA-H
HELO1
HEDJ
HD
HARS
GSD4
GSD15
GS
GPR13
GDE
GBD4
GARS
FRPHE
FLDB
FATP3
FAT
FARSLB
FABPB
FAAHOUT
EIF4G
E1B
DZIP2
DJC6
DEE38
DDX16
CSIF
CMTD1F
CMT4A
CMT2E
CMT2DD
CMT2B
CMT2A
CMT1G

CMT1B
CMRD
CLP46
CHDS7
CGD1
CELIAC1
CDH16
CD10
CCDD
C1orf106
BCPM
BCATE2
ASD2
ARC41
APG16L
AIS5
AIS1
AILJK
ADMD
ADH3
AD4
ABCR
AAOPD
AC133
ADMIO2
ADSD
ADTKD2
ADTKD4
ADTKD5
APBD
ARPKD
BDPLT10
BLBP
BSF2
CALLA
CELIAC3
CMH6
CMT1F
CMT2A1

CMT2A2A
CMT2EE
CMT2F
CMT2K
CMT2R
CMTDIC
CMTDID
DBP2
DJ9
DSMA4
EFABP
EMD2
ESA
ETM6
FACE
FPC
GSD10
GVHDS
HDL4
HFE1
HHD
HKLLS2
HOMGSMR2
HPT1
IBD10
IBD29
IFI1
IFI75
IKSHD
IMD71
IT15
KIAA0473
KTELC1
LDLCQ4
LFIS
LINC00505
MDR1
MDR3
MNMN

MPDT
NISBD1
NPPS
PARK1
PARK11
PARK13
PARK18
PDB3
PDB6
PDE11A2
PDJ
PKD5
PMX2B
POVD1
PSN
PTMP
PYL
RILDBC1
SAC3
SARA2
SCA38
SCA45
SLD
SLEB1
SLEB10
SMAD1
SOPH
SPG79
STGD1
STGD2
STM2
STSL1
TNSALP
USH3B
V28
VAMAS2
VSD1
ABBP2
ALPS5

ALS11
ANDD
BTPS2
CMD1V
CMT2D
CMT2W
CMTDIG
CMTRIA
CMTRIC
DSS
FCHL2
FFM
FPLD2
FTDALS3
HMN2B
HMSN6A
HSF
IBD13
IBD19
ICP3
ILFS1
ILFS2
IMD48
LOMARS
M6S1
MELIOS
MSUDMV
MVCD5
MVCD7
NBLST1
NBLST2
NDGOA
NEP
PARK19
PARK4
PDE11A3
PKD4
PRP8
PRSS25

RMD2
RP41
RUMI
STGD3
TACHD
TYRRS
VODI
YVS
YTS
TOF
TFQTL2
RP19
PPNAD2
PKD6
PCA1
NMOAS
NADGP
MGCA8
ISQMR
HMN5A
HGF
DESMD
CORD12
CMT2T
CMT2A2B
CMD1A
CLCs
CHN2
CCHS
C3orf9
ARHR2
BTOP
CD133
CORD3
DDD4
DMRV
HGPS
SCA34
SCA43

SMAJI
YRS
ARMD2
COLED
LGMDR21
MCDR2
STGD4
TNF
IL6
INS
PNPLA3
PPARG
IL1B
GGT1
TLR4
HFE
IL10
LEP
FAS
TGFB1
NR1H4
AKT1
SLC17A5
APOB
APOE
TP53
HADHA
MIR21
ALB
CAT
IRS1
APOA1
MTTP
RETN
INSR
LEPR
MIR122
MLXIPL
SERPINE1

RBP4
UCP2
DDIT3
LPL
NR1H3
GPT
MIR155
ABCB11
CCL2
PPARGC1A
ADIPOQ
IGF1
HMOX1
PPARA
IRS2
TLR2
CD36
CRP
HNF4A
AHSG
PTEN
MTOR
MIR34A
CXCL8
MIR17
ACACA
HGF
CYP2E1
F2
MIR126
APOC3
SOD2
SOCS3
MIR140
CPT2
IKBKB
MIR132
IFNG
KRT18

FASN
MIR22
MIR30A
EIF2AK3
SLC2A4
CASP3
SREBF1
MIR31
AFP
MIR192
MIR27A
IL1A
CEBPA
MAPK8
FABP1
SIRT1
MIR29C
VEGFA
MIR200B
MIR10B
MIR200A
MIRLET7D
MIR33A
CCN2
MIR200C
CTNNB1
SMPD1
MIR183
CTLA4
SERPINA1
LMNA
STAT3
MIR130A
FABP4
ACE
PIK3CA
MFN2
RELA
ATP7B

MIRLET7E
GHRL
MIR107
MIR26A1
ABCA1
NAFLD1
MIR203A
MTHFR
FASLG
TM6SF2
MSR1
EGFR
MIR16-1
MARS1
XBP1
MIR125A
MMP9
ICAM1
SOD1
CFTR
MIRLET7C
LIPA
TNFRSF1A
APP
ACOX1
CPT1A
MIR451A
NDUFS1
ABCB4
ADIPOR1
CAV1
TIMP1
MIR30D
IL18
ADIPOR2
MIR378A
STAT1
JUN
MIR483

CASP8
CP
LCAT
MAP3K5
CDKN2A
MMP2
MT-TK
ATF4
CYCS
HMGCR
TTR
NOTCH1
TF
MPO
MYC
FGF21
LDLR
TERT
SREBF2
NAFLD2
PON1
IL1RN
NFKB1
EGF
VWF
SMAD4
JAG1
SQSTM1
MIR429
SLC25A13
PIK3R1
ESR1
CCND1
NOS2
BSCL2
AKT2
CETP
VCP
GANAB

G6PC
SPP1
SERPINC1
GCG
BAX
HAMP
ABCC2
HLA-DQB1
BDNF
VIM
CYP3A4
PTPN11
GCKR
ELANE
DPP4
MMP1
CDKN1A
NDUFS4
CXCL10
HSPB1
MT-CO1
PCSK9
POMC
MIR199A1
MIR181B1
LIPC
PRMT7
PRKAG2
CXCR4
TNFRSF1B
SLC2A1
NOD2
THBD
NAMPT
NLRP3
BMP6
PNPLA2
MT-CYB
PDGFRB

SDHB
IGF1R
NGF
CRYAA
ACTB
PKLR
HSPD1
SCD
BCL2
PTGS2
TLR9
SERPINA3
ERN1
C3
TLR1
SDHD
TGFBR2
DRD2
TLR6
NDUFS3
CFH
SLC6A3
TNFSF11
ENPP1
MIR146A
LIPE
UGT1A1
FGF19
NDUFS2
AGPAT2
MDM2
CCL3
ABCB7
DNMT1
NDUFB8
DNAJB11
SDHC
CDK4
FLT1

ASS1
LPA
SLC6A4
SDHA
FN1
IFNA1
PRKAB1
PRKAA1
NDUFV1
NDUFS7
CTSD
CDKN3
APOA5
IL17A
TARDBP
CYP7A1
GRN
AKT3
CASR
PLAU
GCK
KRT8
ANO5
TGFBR1
EZH2
IFIH1
MT-CO2
PC
XDH
ALMS1
MYD88
GSR
FABP2
NDUFV2
MIR195
GH1
MIR151A
CHUK
HBB

SMARCA4
CYP1A1
SIRT3
CCL4
FOS
NDUFA13
MEG3
MIR20A
FGF23
EP300
PIK3R2
NDUFA9
NDUFB9
MIR181A1
NDUFA6
VDR
LOX
CTSB
GSK3B
AKR1D1
IDH1
NR1H2
MIR144
F3
MIR222
RAC1
GARS1
COX5A
MIR221
APOA2
NDUFA2
MT-ND6
CDH2
HTR2A
PLIN1
CRAT
MT-CO3
PIK3CD
TNFAIP3

EIF2S1
NDUFS8
MIR214
CPOX
MIR27B
CDC42
SLC40A1
GSTM1
CYP1A2
GSTP1
HIF1A
SPINK1
CCL5
SUMF1
NDUFA1
AGTR1
CHI3L1
CYBB
NDUFB11
SMAD7
NDUFS6
COX6B1
BGLAP
CHIT1
NFE2L2
MIR182
DGAT1
KRT7
ELOVL6
SERPINA6
TFR2
CRH
RXRA
BMPR1A
ERBB4
ALPP
TNFRSF11B
CYP17A1
PLA2G4A

NDUFB3
VCAM1
LRP6
SERPINF2
ETS1
HSD11B1
FGFR4
LCN2
ADH1C
ALPL
SHBG
MYF5
DSP
CA2
FABP3
KRT19
CD55
IFNA2
MIR15B
SELE
CNR1
MAPK10
HP
ERCC2
HUWE1
MAPK14
AGT
NDUFA10
UQCRC2
NDUFA12
CD44
IRF1
WNT3A
GLUD1
ALDH2
SELP
MIR154
HJV
SMAD3

PEMT
IL6R
TNFSF10
ADH1B
MT-ND3
CYP1B1
NPPB
MIR23B
AIFM1
MYH9
SLC27A1
CYC1
LNCARSR
FADS2
DRD3
SCARB1
CEL
UCP1
MMP13
ADA2
NR1I2
LIVAR
LRPPRC
HNRNPA1
PRKAA2
CPS1
TIMP2
PRSS1
CYBA
IGFBP3
MIR95
CYP2A6
ADRB3
MIR187
NDUFA11
FLI1
SHH
HSP90AA1
PRL

PARP1
UQCRQ
COX8A
FGA
HBA1
SLC27A2
IL15
LITAF
CSF1
IL1R1
GC
STAR
NPY
CES1
COX4I1
AHR
FABP12
IFNL3
CLU
DDX3X
DKK1
SLC10A1
CXCR3
CASP7
S100A9
CASP9
MIR29A
TET2
RARS1
BIRC5
HSPG2
XRCC4
CLCN2
PPIG
SP1
GHR
DNASE1
IGFBP1
GFER

TMPRSS6
ADRB2
FTL
DGAT2
HSPA5
SPARC
GAL
NR0B2
EPRS1
IL21
KL
PTPN1
MIR29B1
MAOA
FGB
CCK
GDF15
DRD1
DNMT3A
MIR149
EIF2AK4
GPX1
PIK3CB
MIR33B
ITCH
MST1
NQO1
HMGB1
COL3A1
LTBP3
PPARD
TPM2
PIK3R3
THBS1
AKR1A1
MIR9-1
APPL1
RARRES2
SDCCAG8

AGER
TMPO
EPAS1
IRF8
NR5A2
SLC25A22
CD163
SHC1
UQCRB
EIF4G1
SI
RPS6KB1
PLAUR
PALLD
TPM1
COX6A1
USF1
PEPD
CD59
CLRN1
ABCG1
NAT2
P4HB
GOLM1
MAPK9
LEPQTL1
CFLAR
NPC1L1
LGALS3
CD14
IL17RA
NDUFAB1
AREG
FDFT1
DPM1
NEAT1
TPM3
GABRG2
DICER1

PLIN2
NLRC4
MERTK
KEAP1
ATP5F1E
FOXF1
RPS27A
DHCR24
BIRC3
ATF6
SLC25A12
SLC25A17
HDAC1
UCP3
FOXC2
GSTT1
PLTP
OGG1
ALAD
GABRA2
SULT2A1
DYSF
KLF6
POSTN
IL1RAPL2
SDC1
SOST
PHB
TSPO
IRF3
E2F1
IFNAR1
PRKCQ
MIR224
EPHX1
COX4I2
NR1I3
MIR423
COL1A1

TFEB
GOT2
MLN
CD68
IL33
RPS6KA3
LPIN1
SFRP4
UQCRFS1
SERPINA7
TRAF3IP2
AXL
ANXA2
CCR1
RPL5
GGT2
BID
IL18BP
NTS
ACACB
GPC4
MAP3K11
SLC39A14
CYP2B6
CCL20
SLC39A4
NDUFB10
FTO
TYSND1
GFPT1
CXCR5
ANGPTL8
CYP4A11
IARS1
ABCC3
EIF2AK2
DNAH11
COX7B
BCL2L11

SAMM50
ATP2A2
PARVB
PHGDH
DARS1
EGR1
ENPP2
UFM1
GLP1R
NDUFA4
TGM2
IL6ST
TBP
TRIB1
COL12A1
CNR2
CANX
MYH14
LOXL2
AOC3
SAR1A
ASCL1
ACSM2B
RPS17
DRD4
CEBPB
PTX3
OPRM1
OLR1
DCLK1
DBH
POR
GSK3A
HDAC9
OGT
EPHX2
STING1
IGF2BP2
SERPINB3

NCAN
FFAR4
SPTAN1
MATN3
SRD5A2
ITGA8
RPS3A
SLC25A1
PTPRD
ALDH9A1
PDXK
TXNRD2
MATR3
TXN
SLC1A3
IL16
SF3B1
TRAF2
CPB2
ATP1A1
TKT
MBOAT7
COL13A1
GCLC
PDGFA
SCAP
PKM
KHK
RUNX3
ERLIN1
IMMT
A2M
ERLIN2
FST
ALOX12
RB1CC1
IRGM
CYP4F2
UBD

CASP2
MIR331
SIK1
COX6A2
FGL1
ITPR2
UGT1A6
HSPA1A
PTPRG
PRKAG1
LBP
VTN
RDH5
PIN1
P2RX7
XRCC5
TRIM21
CXCL11
CCL19
MIR24-1
PRKAB2
UGT1A9
DMGDH
UGT1A7
GPNMB
CCR2
SNTA1
JAZF1
H4-16
CYP4F3
CD82
CUL3
ACSL3
TNFRSF4
CDH4
LECT2
MIR30C1
UGT1A8
PRPF8

MLX	
ZNF365	
FNDC5	
NDUFA5	
FYN	
CYLD	
HYOU1	
EEA1	
NOX4	
FOXA1	
NDUFS5	
SERPINF1	
PRDX1	
EFTUD2	
YEATS2	
LPCAT3	
COX7A2	
CCT5	
CLDN2	
AFAP1-AS1	
NNMT	
YWHAE	
COX6C	
COPA	
TRAPPC9	
COMMD1	
NDUFA8	
HNRNPK	
SERPINA12	
GOT1	
DISC1	
NAT1	
TRAF1	Genecards
XRCC6	
PPP2CA	
CES2	
NDUFC2	
NDUFB6	
UQCR11	

CYP2J2
UQCRC1
TNC
CARS1
TRAP1
RTN4
PRSS2
UGT1A4
PDK4
YY1
JCAD
SIRT6
PLIN5
SNRNP200
MGAM
CDH5
YBX1
TP53BP1
NCOA3
RPL10
UGT1A3
STUB1
SOD3
UBE2D2
ROCK1
CACNA2D1
SLC34A2
AKR1B10
H4C11
LGALS3BP
H4C1
SERPINH1
SLC25A11
IFNL4
IL36A
ACE2
TXNRD1
HPS1
KHDRBS3

CFD
ITLN1
AMPH
AZGP1
POMP
SLC38A8
MMP11
H4C8
PEG10
H4C13
H4C9
NDUFA3
PRKCE
COX8C
H4C15
GSS
NDUFB7
LCN1
H4C4
H4C14
H4C12
IL18RAP
H4C3
PRKAG3
DROSHA
H4C6
H4C2
H4C5
HS3ST1
RPL6
UGT1A10
PCLO
NDUFB4
NDUFB5
CYP8B1
LCP1
SIPA1L2
PPP3CA
LOC110806262

SIRT4
APLN
HIGD1A
MAP3K7
NDUFB2
PDGFC
RORA
TPM4
COX5B
PRKDC
SLC4A4
NDUFC1
NDUFA7
TFAP2B
UQCR10
UQCRH
NDUFB1
MIR363
NDUFV3
NFIC
PZP
CFL1
YIPF1
COX7A1
HNRNPU
PUF60
CXCL16
AQP9
HSPA6
SIRT5
COX7C
ARRDC3
PTPRU
COX7A2L
LEMD2
HTR1B
AHNAK
CLTC
SLC9A9

ALPI
NDUFA4L2
HSD17B13
HDAC8
RIPK3
STEAP4
EEF1A1
MACROD2
SEMA5A
ADH5
MAPK12
CMKLR1
IL34
ADH1A
CCAR2
CCT4
ESM1
PNPLA7
ELK1
EHBP1L1
LINC01016
KAT5
PSMC2
GRK2
RPL3
CD5L
TNFRSF12A
COX7B2
CRACR2A
RPS15
COX6B2
NOS1AP
SLC25A47
PRDX5
ATF3
TRAF3
USP7
NGF-AS1
NDUFC2-KCTD14

MIR103A2
BIRC2
IL1R2
FARP1
GPN1
NRG3
CISD2
PCSK5
UQCRHL
MIR361
SDC2
EGFL6
SLC25A3
INHBA
FAM166B
ESRRG
DDX60L
AQP7
HNMT
ADAMTS9
DGCR8
RPTOR
IGHG1
CAPSL
EDA
MAP3K8
MIR103A1
TAGLN
STX17
TSHB
PSMD2
TP53BP2
UGDH
LAPTM4A
NMT2
SOCS2
PLPPR4
CX3CL1
AGAP1

RPL7
MOGAT2
YWHAG
GPLD1
CCRL2
CTBP2
UGT1A5
ARNT
CDK8
RPN1
GLO1
ST8SIA1
CREB5
YBX3
FAF2
MLXIP
AQP3
CYGB
YWHAQ
SLC46A3
FRK
CCN4
FAP
OSBPL5
TLCD4-RWDD3
SVIL
AATF
ANKK1
CWF19L1
CYP2A13
HCFC1
MIR873
CYP2F1
CALD1
HSP90AB1
ZFP90
MYO6
ZNF512
PDIA2

FETUB
BCAT1
ADRB1
PCNX3
SLC3A2
TNR
AEBP1
UBQLN4
SEL1L3
TTF2
FOXK1
ZP4
C2orf16
TEX36
PPIB
RPL7A
SORCS3
SORCS2
ARL2BPP6
ATAD3A
CAD
SLC30A6
ADAMTS9-AS2
NDRG2
VIL1
DDX1
NUCB2
CHST2
GPT2
MCM7
NFE2
MIR6090
APOF
CCNC
MYO1B
PCBP1
FAM171A1
PTBP1
LOC643339

RPL12
NFE2L1
BBC3
MYH10
FAM107B
HSPH1
IAH1
SFRP5
RPL9
TRIB3
COLGALT1
RNU6-53P
MIR372
RND3
ARPC1B
NCL
KCNK7
MACROH2A2
PCBP2
DDX5
TMBIM1
HNRNPM
DISC2
SDK1
SLC9A4
ATXN2L
PPP1CA
PFKFB3
POLR3H
ETS1-AS1
RPL27
APPL2
L13304-005
KLHL6
PSG1
NRG4
GRM4
RPL30
SMIM20

HNRNPC
MYO18A
SENP3
CTSS
KRTAP5-5
CNOT4
KIF16B
RPS4X
FOXN3
SERPINA4
AGR3
GGT3P
MYL6B
POLR2D
WWTR1
PDE7B
DDX60
DDX50
SLC6A9
piR-37045
lnc-PNPLA5-2
APLNR
RUVBL2
C1QTNF3
AP2B1
ENSG00000253111
LINC02694
MCM3
GCN1
MRTFA
SIRT7
YWHAB
TUBA1C
ONECUT2
GATAD2A
SRSF6
PWWP3A
MCM5
WSPAR

ATP5F1B
RPL13
CLRN1-AS1
GSDMD
CCT3
H1-4
MCF2L2
RPS2
TMOD3
RPLP2
MYO1C
SNHG18
NUDT4
IGF2BP1
ACTR5
YWHAZ
TSNAX-DISC1
CCT2
SLC25A10
RPS15A
lnc-PUM2-3
lnc-RHOB-11
RPS23
CILP2
LINC01679
RPS11
RESF1
C16orf95
IP6K3
MIR1296
XPO4
ENSG00000287092
LINC00917
LINC00322
RPL10A
DNAJA1
SAT1
ZNF101
QARS1

TCP1
SLC30A9
GRIK3
SP4
ENSG00000254599
lnc-CRYAA-1
lnc-JCAD-2
lnc-CRYAA-17
ENSG00000226026
RPL4
NUDT21
MYO1D
STRA8
MIR646HG
ENSG00000231487
PLPP7
A2MP1
EIF3L
RNU6-961P
ARHGAP8
IL36B
HNRNPF
SRSF1
LOC389199
RAB37
TRMT44
RPS9
ENSG00000255046
ENSG00000287292
ENSG00000222042
lnc-LTBP3-4
OTX2P1
ENSG00000237936
MN309375
BECN2
INSIG2
RPL8
GSTK1
RPS18

TLL1
ENSG00000234389
ENSG00000249631
lnc-FLI1-5
ZNF267
G0S2
MIR1183
HNRNPH1
MIR449A
C1QTNF1
LRRFIP2
ANXA9
CRTC1
SVIL-AS1
SNRPD2
SLC9A9-AS1
TEX36-AS1
LOC100130331
SLC9A9-AS2
ENSG00000257322
RNU6-908P
ENSG00000236501
ENSG00000250541
ENSG00000265994
ENSG00000207171
ENSG00000235464
RNU1-35P
EEF1A1P20
ENSG00000236389
ENSG00000287032
ENSG00000284676
lnc-DDX60L-3
ENSG00000226973
ENSG00000199378
lnc-KCNK7-1
HSALNG0076986
ENSG00000276627
piR-41195-080
lnc-UBE2N-9

lnc-EDRF1-4
piR-59297-626
LOC105372698
piR-38051-198
HSALNG0076987
LOC107986198
LOC102723733
ENSG00000270927
piR-57133-376
AB372775
RF00994-591
piR-31109-001
RF00017-1202
lnc-AIFM2-3
ENSG00000283174
ENSG00000213117
ENSG00000254939
RPL24
RNU6-976P
ENSG00000283991
lnc-TRIB1-7
LOC105375131
ENSG00000268473
STMP1
DHX9
PSME2
RBFOX1
THNSL2
LINC00355
AP2A2
TUBA8
LOC101929710
INPP4B
PABPC1
POT1-AS1
ST13P12
NLRP2
LINC02789
LINC01090

PSAPL1
ENSG00000237596
ENSG00000250001
LOC105373944
ENSG00000248537
HNRNPA1P58
LOC101060035
SLC23A2
ACTR2
FECHP1
EIF4A3
LOC101928135
KRT8P18
ACTR3
PRR5-ARHGAP8
PHF21B
ENSG00000274121
ENSG00000286071
ATP6AP1L
SLC23A1
SRSF7
STARP1
LINC00888
PBX2P1
SERPINB4
HNRNPA3
USP4
TET1
TPI1P1
LOC401864
SLC23A4P
MIR378C
MIR4772
AP2A1
CPB2-AS1
HDHD5-AS1
LINC01029
SNORA63E
LINC02392

LINC02534
ENSG00000243273
SNORA63D
LINC01818
ENSG00000260234
ENSG00000259053
ENSG00000251152
RNU6-655P
RNU6-161P
ENSG00000241679
RPL35AP33
ENSG00000232739
ENSG00000234481
ENSG00000252040
ENSG00000253092
ENSG00000249664
LGMNP1
ENSG00000253354
ENSG00000272479
LINC02822
HNRNPA3P5
RNU6-1172P
MRPL45P1
ENSG00000197462
ENSG00000235503
ENSG00000234034
LOC107987158
ENSG00000251730
ENSG00000254171
RPL23AP20
ENSG00000259080
lnc-SLC46A3-1
ENSG00000288092
lnc-TSNAX-2
SDR42E1P5
lnc-MRGPRG-1
ENSG00000233983
MN309223
ENSG00000271682

ENSG0000028772(
lnc-SLC22A18-7
lnc-RASGRP1-1
lnc-WDR91-4
piR-50443-100
ENSG0000022911(
ENSG0000022590(
HSALNG0113292
piR-61101-044
ENSG0000023198(
piR-61861-018
hsa-miR-5096-076
ENSG0000027538(
piR-48470
RF00026-283
piR-40638
piR-40663
piR-49272
piR-48794
piR-48799-012
piR-49129
piR-39926
piR-39858-368
piR-56788
piR-56842
piR-32810-118
piR-33024
piR-32825
piR-32961
ENSG0000028552(
piR-48131
piR-39137
piR-32153
piR-49710
piR-59496
piR-43106-189
piR-34682
piR-42506
piR-42270

lnc-TRIB1-8
HSALNG0031159
HSALNG0032693
piR-43926-003
piR-44231
piR-59890
piR-35359
piR-36491
lnc-XRCC4-4
piR-54702
LOC105373170
piR-45773
piR-55199
piR-46362
NONHSAG018421.[illegible]
lnc-KLHL24-6
piR-44286
piR-37903
piR-37780
piR-45939
LOC105373943
piR-61711
NONHSAG013110.[illegible]
LOC105379051
RF00017-1416
LOC107986000
ENSG00000258506
piR-30178
piR-55631
piR-30153
piR-47596
piR-30883
HSALNG0014027
piR-36047
piR-55036-040
piR-46640
ENSG00000276067
ENSG00000271078
piR-31534-137

ENSG00000279912
ENSG00000280306
ENSG00000219736
ENSG00000220748
LOC102724253
LOC107984123
LOC105377705
RPL23A
RUVBL1
IBTK
AUP1
ILF3
ARPC2
RRBP1
RTRAF
EIF4A1
SRSF4
SUGP1
LOC110366354
ENHO
AHSA1
RNF114
CTPS1
SEC16A
RPS8
TRIM33
TMSB4X
SRRM1
DUSP26
EIF3E
DDX17
CCT8
CAMKK2
EDC4
EIF3B
HKDC1
CKAP4
HNRNPUL1
MAPK13

EIF3F
RBM14
UBE2D3
FARSA
HSD17B11
PDIA4
PGAM5
FAM3A
SLC25A48
PKNOX1
TMEM97
PABPC4
SF3B3
C1QTNF9
MIR190B
CAND1
MYBBP1A
USP48
TMC4
TXNL1
HNRNPL
DEFA1
EIF4A2
LAMP3
DHX15
SUB1
TMBIM6
ILF2
SRRM2
FAM3B
RTCB
OPRD1
GLT8D2
EBNA1BP2
LATS2
GGT5
HMCES
SMOC2
PHF2

SNRNP40
FAT1
DDX46
PHF5A
KDM4B
ARPC4
NMBR
UBXN1
SLC2A8
RGS4
FAIM
USP10
CLGN
LYPLAL1
SH3BP5
STK24
IGHG4
DUSP14
SEC16B
DNAJC10
RANGAP1
H2BC18
HSP90AA2P
COP1
H2BC11
RAB32
PNLIPRP2
RAMP3
TRIM47
TRPC4AP
ZNF300
SDF2L1
BMP8B
FNDC3B
DNAJC11
FUNDC2
HIF3A
IGKV2D-23
RUBCNL

LOC109279247
LINC01684
TXNDC5
RPL17-C18orf32
PHLPP2
MIR103B2
MIR103B1
SAP18
MAS1
CTDNEP1
YIPF6
CARD6
SUCNR1
PRMT3
HERC4
CR1L
FAM174A
MIR4668
TM6SF1
TM6SF2
PNPLA3
CYP2E1
SIRT1
FGF21
LEP
NFE2L2
PPARA
ADIPOQ
NR1H4
PPARD
SREBF1
LDLR
XBP1
CAT
FAS
GNMT
PEMT
CPT1A
NR5A2

TGFB1
AHR
GSTM1
GSTP1
IL1A
JAK2
PRKCA
ABCC2
KLB
ACE
ALDH2
GSTT1
PTEN
CD14
NQO1
FOLR2
GSTA1
IL4
LIF
PDK4
PRKCD
PRKCE
VLDLR
ADH1A
ADH1B
ADH4
CSF2
CYP1A2
CYP17A1
AHCY
F2
ALDH1A1
ALDH1B1
B3GAT1
EIF2AK1
LAMA1
IL3
MMP1
SERPINB2

ABCB4
PRF1
PRKACA
TRIB3
RAG2
RDX
TNFRSF1B
IKBKG
STC2
ALDH4A1
SCARB1
GCKR
ATP5F1B
NCAN
PARVB
SAMM50
FDFT1
PTPRD
ZGLP1
PPARGC1A
NLRP3
OSBPL5
COL13A1
CRP
DPP4
ERBB4
FBL
ALB
EHBP1L1
GCG
SLC17A5
GGT1
GLP1R
GPT
HFE
APOC3
IGF1
IL1B
IL6

KRT18
LEPR
LIPA
MIR122
MIR21
MIR34A
MTTP
PIK3CA
PIK3CB
PIK3CD
PIK3CG
PPARG
GATAD2A
PRKAA1
PRKAA2
PRKAB1
RBP4
SCD
SHBG
SLC5A2
GGTLC5P
SP4
SREBF2
TLR4
TNF
GGTLC3
GGT2
GGTLC4P
CNBP
MBOAT7
EHMT1
ACTR5
ZNF512
CRACR2A
PLPP7
PWWP3A
TP63
DDX60L
XPR1

CD36
CREB5
FGF19
AHSG
FASN
GABPA
GH1
LBP
LGALS3BP
HAMP
REN
NR1H3
CYP3A4
FABP1
SIRT3
HNF4A
HSD17B13
IL17A
MLXIPL
CCL2
LOC102724197
AGTR1
MTOR
FETUB
APOE
PON1
RETN
RARRES2
UCP2
NR0B2
GGTLC1
DECR1
PCSK9
ACACA
HMGB1
APOB
IL10
LPL
MIR192

MTHFR
SERPINE1
ANGPTL8
SOD1
TP53
VDR
IRS2
TRIB1
TXNIP
PLIN2
CNR1
CYBB
AKT1
ABCD1
FOXO1
GOLGA6A
APRT
IRS1
MIR33A
MFAP1
NR3C2
SERPINA1
RCBTB1
PNPLA2
SELENOP
SOD2
STAT3
PPP1R3B
NR1I2
FADS2
CEBPA
CETP
SLC27A5
APOA5
COL1A1
KLF6
NRG4
TMC4
EGFR

EPAS1
FABP4
FGL1
FLII
GCK
IFNL3
HIF1A
HP
HSD11B1
IFNG
APP
IL18
INSR
MIR29A
MAP3K5
CHPT1
BGLAP
SPP1
TLR2
CCR2
VEGFA
FTO
CASP1
ABCB11
TNFSF10
NAMPT
FST
PRRT2
CMKLR1
CPT2
PARP1
CTNNB1
GPBAR1
CYP7A1
DLAT
AFP
AGER
AGT
ABCA1

FGFR4
SMUG1
GIP
HPGDS
GOT2
NEAT1
OR10A4
GPX1
ANPEP
NPC1L1
HMOX1
CXCL10
CYP4F3
ARNTL
MIR140
MIR155
MMP2
MT1B
ACLY
NTS
MIR378A
BCO1
PRKCB
MAPK8
PRL
MIR451A
CXCL16
ACE2
BCL2
BCL2A1
SAV1
SLC10A2
SMN1
SMN2
BNIP3
TERT
TIMP1
TIMP2
ADIPOR2

BCO2
CAV1
GDF15
BMS1
NR1I3
FAS-AS1
LINC01672
PSC
TLR6
YAP1
POSTN
CES1
CORIN
CCL27
MTMR11
CHIT1
ECD
C1QTNF1
LYPLAL1
BHLHE23
MAP3K8
COX8A
GPR119
CYBA
CYP1A1
GRK2
CYP2D6
ATN1
MLKL
ELANE
ELAVL2
ESR1
ESRRA
ACSL4
FAT1
FCGR2B
SIRT2
FOXO3
LPIN1

MLYCD
FOS
ALOX12
ALOX5
ALOX15
ALPP
FNDC5
ATRNL1
GC
GCGR
PDLIM3
GLUL
SLC13A5
GRN
SETD2
NR3C1
TBK1
PDIA3
FOXA1
ACACB
HSPA1A
HSPA1B
HSP90AA1
FFAR4
IDH2
IRGM
IGFALS
CXCL8
INS
AQP9
AR
ENHO
LCN2
LECT2
MIR126
MIR149
MIR20A
MIR200C
MIR222

MAS1
MC4R
MMP13
PLIN5
MUC1
ATF3
ATHS
CCN3
MIR375
TNFRSF11B
IL22
ACOX1
ADIPOR1
CRNKL1
HSD17B7
GDE1
ENPP1
LUC7L3
FAM3B
UGT1A1
ELOVL2
AVP
NAT10
PTPA
MEG3
MAPK1
MYDGF
AZGP1
PRTN3
PTGS1
MIR146B
PTPN1
NLRC4
BCAT1
MOK
FAM3A
ROCK1
HCC
S100A8

CEACAM1
SCP2
CCL20
SELPLG
ABCG5
IL25
SLC2A1
SLC2A2
NCF1
SLPI
SPARC
SPINK1
SPTBN1
STAT5A
STAT5B
TEK
TRPV1
DHRS11
STEAP4
CALCR
ASRGL1
CASP3
CASP8
ORAI1
TNFSF11
SOCS1
CBR1
RIPK1
CCN4
APLN
SELENBP1
SOCS3
USP10
CD5L
CD163
PPIG
CD68
MIR873
MIR190B

CD24
MIR1224
PARP2
LOC100505909
OCLN
DNM1L
ERVK-2
PARTICL
PPIF
IFNL4
LRPPRC
SLC25A13
CDK4
B4GALT1-AS1
PRMT3
PLIN3
CDK8
FSTL3
CDKN2A
ZNF267
AKR1A1
MERTK
CAP1
STK25
NCOA2
ATG7
LINC01684
PRDX4
CEL
SORBS1
AHSA1
ERLIN1
CAMKK2
CXCR6
SIRT1-AS
TNFSF13B
EBP
YME1L1
PLK4

HPSE
PGRMC1
NMU
TMED2
SLC27A2
LILRB4
RIPK3
LOC110806263
ADAMTS13
TREH
LNCARSR
DUSP12
CHRM3
VSIG4
MGLL
TXNRD3
CHUK
C1QTNF3
CIDEA
H3P10
TWIST2
CLCN2
IFTAP
CYP2R1
CPT1C
CNR2
COL3A1
SLC31A1
ADM
PPARGC1B
NADK2
CP
CPN1
CPOX
CPS1
CREB1
ATF2
CREBBP
CRK

CRMP1	
MAPK14	Disgenet
FGFBP3	
CSF3	
CSF3R	
SLC38A8	
ZFP90	
CILP2	
CTAA1	
HJV	
CTF1	
CCN2	
PDIK1L	
CTNNA1	
SIK1	
HORMAD2	
CTRL	
CTSB	
CTSD	
CTSG	
CTSS	
ADRB2	
SLC2A12	
CYP2A6	
CYP2B6	
CYP2B7P	
CYP2D7	
CYP2J2	
CYP4A11	
CYP8B1	
CYP19A1	
CYP27B1	
CYP51A1	
DBP	
TMPRSS6	
AEBP1	
DDOST	
TIMM8A	
DHCR7	

DIO3
DMD
DPYSL3
SLC26A3
JAG1
DSPP
DUSP9
EDA
S1PR1
EEF1A1
EGF
EGR1
EIF4E
A2M
LINC01554
ENO3
EPHB2
EPHB6
EPO
AKT2
ERN1
ESRRB
EZH2
F2RL1
FABP2
FABP5
ACSL1
PTK2B
UNC5B
FBN1
MARCHF8
FCGR3A
FCGR3B
JAZF1
GPC4
FGF1
FGR
MLXIP
ZHX2

ALDOB
DKK1
PHLPP2
SARM1
AKR1B1
MPRIP
MCF2L
CRTC1
SIRT4
ANGPTL2
BRD4
SRRM2
MMD
FOSB
FOSL2
DDAH1
DAPK2
MKRN1
TMEFF2
IL17RA
ZBTB38
G6PC
PRDX5
GALR1
RNF19A
WWTR1
TIPARP
OSBPL3
APPL1
POLDIP2
TOR1AIP1
PRPF31
TRPC4AP
AMD1
FGF20
AMD1P2
LATS2
AATF
GDNF

NOX1
SIGLEC7
INTU
NAAA
IL37
GPC3
GLB1
GCLC
RBMS3
GLI2
MAT2B
GLRX
GNA12
GNAI3
GNAO1
ABO
GPI
ANGPT1
BTBD8
ANGPT2
GPR31
FFAR2
GPS2
MYLIP
CTNNA3
GSK3B
GSTM2
REPIN1
CERS2
DMGDH
SLC2A8
SLC40A1
H2AX
HADHB
HADH
HCFC1
HLA-A
HLA-C
HLA-DQA1

HLA-DQB1
HLA-DQB2
HLA-DRB1
AOX1
NR4A1
FOXA2
BPIFA4P
APOF
HOXD13
AIRE
HRG
PRMT1
HSPA4
HSPA5
XIAP
HSPA8
HSPG2
APOA1
HTR2A
APOA2
TNC
HCAR2
C1QTNF9
STING1
CPP
IFNA1
IFNA2
IFNA13
MOGAT3
IGF2
IGFBP1
IGFBP2
IGFBP4
IGFBP7
KLK3
IKBKB
IL2
CXCR2
IRF2BP2

INPP5D
INSIG1
AQP7
IRF3
ITGAM
ITGB1
ITIH4
ITPR2
JAK1
JUN
JUNB
JUND
CD82
AREG
ACAT1
ARG2
KRT8
RTL1
C1QL3
LCAT
LCP1
LGALS3
LIPE
FADS1
LNPEP
LOXL2
LRP6
LINC01194
ARNT
LTF
LUM
MIR130A
MIR132
MIR136
MIR141
MIR142
MIR143
MIR144
MIR146A

MIR150
MIR15B
MIR17
MIR185
MIR200B
MIR205
MIR206
MIR212
MIR223
MIR23B
MIR24-1
MIR27A
MIR27B
MIR29B1
MIR29B2
MIR29C
MIR30B
MIR30C1
MIR30C2
ARRB1
SMAD7
MAT1A
DNAJB9
MEFV
MET
MIF
CXCL9
MMP9
MPI
MPO
MPST
MRC1
ASNS
EIF2AK4
MIR330
MIR331
MIR367
MST1
ND6

MYC
MYD88
HNRNPM
ATF4
NBN
NEU1
NHS
NNMT
NOS2
NOS3
NOTCH1
NRF1
NUCB2
CISD2
MIR361
MIR423
P2RX5
P4HA1
DUOX2
NOX4
IL20
PAPPA
PC
SOST
PCK1
ABHD5
INSIG2
MBTPS2
ISYNA1
SIRT6
PDGFRB
ARID4B
SERPINA4
SERPINB6
PIGR
PITX3
PKM
PKNOX1
PLCG1

PLG
PLTP
PMCH
DUOX1
TREM1
CCHCR1
UGT1A10
UGT1A8
UGT1A7
UGT1A6
EGLN1
UGT1A4
CROT
TET2
SLC52A1
PPP1R3C
PIWIL2
NUDT11
APPL2
SLC47A1
CWF19L1
FBXW7
CHDH
NLRP2
TRERF1
ALLC
PAG1
ACOT13
PRKDC
ERBIN
MAPK3
MAP2K7
PRLR
LGALS14
UBQLN4
PNO1
ADAMTS9
BAAT
PCBP4

SMURF1
PTBP1
PTGFRN
MIR20B
MIR193B
LRRC7
SEMA6A
ARRDC3
PTPN6
PTPRC
PTX3
BAX
PGAP6
BCHE
RARA
TRPV4
OPN1LW
RELA
ACTB
RLN2
BRD2
RPS6KB1
SORT1
S100A4
CCL5
CCL17
CCL19
CXCL5
CIDEC
SELL
SELP
SMOC2
TMBIM1
SETMAR
SFRP4
ABCG8
SFRP5
SRSF3
NSD1

XPO4
BLVRA
GORASP1
ARV1
ST3GAL4
WNK1
SLC2A4
BMP4
SLC3A2
FRTS1
SLC6A2
SNHG20
SLC10A1
BMP8B
SLC13A1
AACS
SMPD1
SIGLEC1
SNAI1
SORD
SP1
SPRR2A
SQLE
AKR1D1
STAR
STAT1
STAT6
SULT1E1
STK11
SYT1
ADAM17
MAP3K7
TAZ
MIR33B
MIR590
TCF7L2
TFAM
TFF3
TG

TGFB1I1
TSPO
THRSP
TIMP3
TLL1
TLR1
NR2E1
TM7SF2
TMPO
TNFAIP3
TNFRSF1A
TP53BP1
TPO
C3
TRAF3
DNLZ
HULC
UCP1
UCP3
SLC35A2
UGT1A
UGT2B4
USP4
UROD
UTRN
VCAM1
VEGFC
VIP
BEST1
YY1
ZBTB16
DUSP26
ELOVL6
APOO
LAP
PLA2G7
TFEB
CALCA
AIMP2

NLRX1
KCTD17
GSDMD
DHDDS
HKDC1
STAM
CPEB4
COASY
FOSL1
FGF23
SPX
SLC7A5
BAS
SESN2
TRAPPC9
ITCH
ANGPTL6
PLA2G6
CHCHD6
MAK16
DGAT2
CARD6
FAM83A
PIK3R3
CYP4F2
DENR
RUNX2
RTCA
DGAT1
ABCC3
MBTPS1
TNFSF14
DLK1
TRIM24
CES2
HDAC3
SQSTM1
CCNC
MBD2

WNT3A
CCRL2
TBL1Y
MYOM2
TIMD4
ZNF300
HACD1
NOG
GLP2R
COX5A
GRAP2
TMPRSS11D
EIF2AK3
GGPS1
ATG5
LITAF
CLOCK
CD44
ABCG1
KEAP1
PLPPR4
MFN2
CDC42
CDH1

Information of GeneCards

Gene Symbol	Description	Category	Gifts	GC Id	Relevance	GeneCards Link
TNF	Tumor Necrosis Factor	Protein Coding	51	GC06P047305	100.16	https://www.genecards.org/cgi-bin/carddisp.pl?gene=TNF
IL6	Interleukin 6	Protein Coding	50	GC07P022765	94.68	https://www.genecards.org/cgi-bin/carddisp.pl?gene=IL6
INS	Insulin	Protein Coding	48	GC11M002159	94.14	https://www.genecards.org/cgi-bin/carddisp.pl?gene=INS
PNPLA3	Patatin Like Phospholipase Domain Containing 3	Protein Coding	41	GC22P043923	91.2	https://www.genecards.org/cgi-bin/carddisp.pl?gene=PNPLA3
PPARG	Peroxisome Proliferator Activated Receptor Gamma	Protein Coding	52	GC03P012287	75.38	https://www.genecards.org/cgi-bin/carddisp.pl?gene=PPARG
IL1B	Interleukin 1 Beta	Protein Coding	48	GC02M112829	75.33	https://www.genecards.org/cgi-bin/carddisp.pl?gene=IL1B
GGT1	Gamma-Glutamyltransferase 1	Protein Coding	46	GC22P024927	73.1	https://www.genecards.org/cgi-bin/carddisp.pl?gene=GGT1
TLR4	Toll Like Receptor 4	Protein Coding	51	GC09P117704	72.22	https://www.genecards.org/cgi-bin/carddisp.pl?gene=TLR4
HFE	Homeostatic Iron Regulator	Protein Coding	43	GC06P026087	71.24	https://www.genecards.org/cgi-bin/carddisp.pl?gene=HFE
IL10	Interleukin 10	Protein Coding	47	GC01M206767	68.87	https://www.genecards.org/cgi-bin/carddisp.pl?gene=IL10
LEP	Leptin	Protein Coding	47	GC07P128241	68.42	https://www.genecards.org/cgi-bin/carddisp.pl?gene=LEP
FAS	Fas Cell Surface Death Receptor	Protein Coding	50	GC10P088969	67.65	https://www.genecards.org/cgi-bin/carddisp.pl?gene=FAS

TGFB1	Transforming Growth Factor Beta 1	Protein Coding	52	GC19M041301	67.61	https://www.genecards.org/cgi-bin/carddisp.pl?gene=TGFB1
NR1H4	Nuclear Receptor Subfamily 1 Group H Member 4	Protein Coding	48	GC12P100473	66.88	https://www.genecards.org/cgi-bin/carddisp.pl?gene=NR1H4
AKT1	AKT Serine/Threonine Kinase 1	Protein Coding	54	GC14M104769	66.1	https://www.genecards.org/cgi-bin/carddisp.pl?gene=AKT1
SLC17A5	Solute Carrier Family 17 Member 5	Protein Coding	44	GC06M073593	65.17	https://www.genecards.org/cgi-bin/carddisp.pl?gene=SLC17A5
APOB	Apolipoprotein B	Protein Coding	45	GC02M020956	64.28	https://www.genecards.org/cgi-bin/carddisp.pl?gene=APOB
APOE	Apolipoprotein E	Protein Coding	50	GC19P044906	63.18	https://www.genecards.org/cgi-bin/carddisp.pl?gene=APOE
TP53	Tumor Protein P53	Protein Coding	54	GC17M007661	60.99	https://www.genecards.org/cgi-bin/carddisp.pl?gene=TP53
HADHA	Hydroxyacyl-CoA Dehydrogenase Trifunctional Multi	Protein Coding	45	GC02M026190	59.82	https://www.genecards.org/cgi-bin/carddisp.pl?gene=HADHA
MIR21	MicroRNA 21	RNA Gene	24	GC17P059841	57.95	https://www.genecards.org/cgi-bin/carddisp.pl?gene=MIR21
ALB	Albumin	Protein Coding	50	GC04P073397	57.76	https://www.genecards.org/cgi-bin/carddisp.pl?gene=ALB
CAT	Catalase	Protein Coding	50	GC11P034460	57.41	https://www.genecards.org/cgi-bin/carddisp.pl?gene=CAT
IRS1	Insulin Receptor Substrate 1	Protein Coding	47	GC02M226731	57.25	https://www.genecards.org/cgi-bin/carddisp.pl?gene=IRS1
APOA1	Apolipoprotein A1	Protein Coding	48	GC11M116835	56.54	https://www.genecards.org/cgi-bin/carddisp.pl?gene=APOA1
MTTP	Microsomal Triglyceride Transfer Protein	Protein Coding	44	GC04P099563	56.05	https://www.genecards.org/cgi-bin/carddisp.pl?gene=MTTP
RETN	Resistin	Protein Coding	43	GC19P007669	55.73	https://www.genecards.org/cgi-bin/carddisp.pl?gene=RETN
INSR	Insulin Receptor	Protein Coding	54	GC19M007112	55.47	https://www.genecards.org/cgi-bin/carddisp.pl?gene=INSR
LEPR	Leptin Receptor	Protein Coding	49	GC01P065421	55.21	https://www.genecards.org/cgi-bin/carddisp.pl?gene=LEPR
MIR122	MicroRNA 122	RNA Gene	21	GC18P058451	54.79	https://www.genecards.org/cgi-bin/carddisp.pl?gene=MIR122
MLXIPL	MLX Interacting Protein Like	Protein Coding	41	GC07M073593	52.23	https://www.genecards.org/cgi-bin/carddisp.pl?gene=MLXIPL
SERPINE1	Serpin Family E Member 1	Protein Coding	50	GC07P101127	51.96	https://www.genecards.org/cgi-bin/carddisp.pl?gene=SERPINE1
RBP4	Retinol Binding Protein 4	Protein Coding	44	GC10M093591	51.54	https://www.genecards.org/cgi-bin/carddisp.pl?gene=RBP4
UCP2	Uncoupling Protein 2	Protein Coding	45	GC11M073974	51.5	https://www.genecards.org/cgi-bin/carddisp.pl?gene=UCP2
DDIT3	DNA Damage Inducible Transcript 3	Protein Coding	45	GC12M057516	51.45	https://www.genecards.org/cgi-bin/carddisp.pl?gene=DDIT3
LPL	Lipoprotein Lipase	Protein Coding	49	GC08P019901	51.29	https://www.genecards.org/cgi-bin/carddisp.pl?gene=LPL
NR1H3	Nuclear Receptor Subfamily 1 Group H Member 3	Protein Coding	47	GC11P047248	51.19	https://www.genecards.org/cgi-bin/carddisp.pl?gene=NR1H3
GPT	Glutamic--Pyruvic Transaminase	Protein Coding	41	GC08P144502	50.87	https://www.genecards.org/cgi-bin/carddisp.pl?gene=GPT
MIR155	MicroRNA 155	RNA Gene	18	GC21P025573	49.83	https://www.genecards.org/cgi-bin/carddisp.pl?gene=MIR155
ABCB11	ATP Binding Cassette Subfamily B Member 11	Protein Coding	45	GC02M168922	49.79	https://www.genecards.org/cgi-bin/carddisp.pl?gene=ABCB11
CCL2	C-C Motif Chemokine Ligand 2	Protein Coding	48	GC17P034255	49.4	https://www.genecards.org/cgi-bin/carddisp.pl?gene=CCL2
PPARGC1A	PPARG Coactivator 1 Alpha	Protein Coding	46	GC04M023755	49.26	https://www.genecards.org/cgi-bin/carddisp.pl?gene=PPARGC1A
ADIPOQ	Adiponectin, C1Q And Collagen Domain Containing	Protein Coding	45	GC03P186842	49.13	https://www.genecards.org/cgi-bin/carddisp.pl?gene=ADIPOQ
IGF1	Insulin Like Growth Factor 1	Protein Coding	50	GC12M102395	48.68	https://www.genecards.org/cgi-bin/carddisp.pl?gene=IGF1
HMOX1	Heme Oxygenase 1	Protein Coding	52	GC22P035380	47.4	https://www.genecards.org/cgi-bin/carddisp.pl?gene=HMOX1
PPARA	Peroxisome Proliferator Activated Receptor Alpha	Protein Coding	45	GC22P046150	47.36	https://www.genecards.org/cgi-bin/carddisp.pl?gene=PPARA
IRS2	Insulin Receptor Substrate 2	Protein Coding	44	GC13M109752	47.11	https://www.genecards.org/cgi-bin/carddisp.pl?gene=IRS2
TLR2	Toll Like Receptor 2	Protein Coding	51	GC04P153684	47.01	https://www.genecards.org/cgi-bin/carddisp.pl?gene=TLR2
CD36	CD36 Molecule	Protein Coding	48	GC07P080369	46.8	https://www.genecards.org/cgi-bin/carddisp.pl?gene=CD36
CRP	C-Reactive Protein	Protein Coding	46	GC01M159716	45.61	https://www.genecards.org/cgi-bin/carddisp.pl?gene=CRP
HNF4A	Hepatocyte Nuclear Factor 4 Alpha	Protein Coding	50	GC20P044355	45.34	https://www.genecards.org/cgi-bin/carddisp.pl?gene=HNF4A

AHSG	Alpha 2-HS Glycoprotein	Protein Coding	44	GC03P186612	45	https://www.genecards.org/cgi-bin/carddisp.pl?gene=AHSG
PTEN	Phosphatase And Tensin Homolog	Protein Coding	52	GC10P087863	44.94	https://www.genecards.org/cgi-bin/carddisp.pl?gene=PTEN
MTOR	Mechanistic Target Of Rapamycin Kinase	Protein Coding	54	GC01M011106	44.71	https://www.genecards.org/cgi-bin/carddisp.pl?gene=MTOR
MIR34A	MicroRNA 34a	RNA Gene	22	GC01M009151	43.68	https://www.genecards.org/cgi-bin/carddisp.pl?gene=MIR34A
CXCL8	C-X-C Motif Chemokine Ligand 8	Protein Coding	41	GC04P073740	43.41	https://www.genecards.org/cgi-bin/carddisp.pl?gene=CXCL8
MIR17	MicroRNA 17	RNA Gene	21	GC13P091350	41.73	https://www.genecards.org/cgi-bin/carddisp.pl?gene=MIR17
ACACA	Acetyl-CoA Carboxylase Alpha	Protein Coding	48	GC17M037084	41.51	https://www.genecards.org/cgi-bin/carddisp.pl?gene=ACACA
HGF	Hepatocyte Growth Factor	Protein Coding	52	GC07M081699	41	https://www.genecards.org/cgi-bin/carddisp.pl?gene=HGF
CYP2E1	Cytochrome P450 Family 2 Subfamily E Member 1	Protein Coding	45	GC10P133520	39.75	https://www.genecards.org/cgi-bin/carddisp.pl?gene=CYP2E1
F2	Coagulation Factor II, Thrombin	Protein Coding	48	GC11P046720	39.46	https://www.genecards.org/cgi-bin/carddisp.pl?gene=F2
MIR126	MicroRNA 126	RNA Gene	22	GC09P136670	39.17	https://www.genecards.org/cgi-bin/carddisp.pl?gene=MIR126
APOC3	Apolipoprotein C3	Protein Coding	43	GC11P116829	39.12	https://www.genecards.org/cgi-bin/carddisp.pl?gene=APOC3
SOD2	Superoxide Dismutase 2	Protein Coding	51	GC06M159669	38.91	https://www.genecards.org/cgi-bin/carddisp.pl?gene=SOD2
SOCS3	Suppressor Of Cytokine Signaling 3	Protein Coding	44	GC17M078356	38.57	https://www.genecards.org/cgi-bin/carddisp.pl?gene=SOCS3
MIR140	MicroRNA 140	RNA Gene	22	GC16P069934	38.19	https://www.genecards.org/cgi-bin/carddisp.pl?gene=MIR140
CPT2	Carnitine Palmitoyltransferase 2	Protein Coding	48	GC01P053196	38.06	https://www.genecards.org/cgi-bin/carddisp.pl?gene=CPT2
IKBKB	Inhibitor Of Nuclear Factor Kappa B Kinase Subunit B	Protein Coding	52	GC08P042271	37.72	https://www.genecards.org/cgi-bin/carddisp.pl?gene=IKBKB
MIR132	MicroRNA 132	RNA Gene	21	GC17M002049	37.46	https://www.genecards.org/cgi-bin/carddisp.pl?gene=MIR132
IFNG	Interferon Gamma	Protein Coding	48	GC12M068064	37.2	https://www.genecards.org/cgi-bin/carddisp.pl?gene=IFNG
KRT18	Keratin 18	Protein Coding	48	GC12P052948	37.06	https://www.genecards.org/cgi-bin/carddisp.pl?gene=KRT18
FASN	Fatty Acid Synthase	Protein Coding	49	GC17M082078	37.02	https://www.genecards.org/cgi-bin/carddisp.pl?gene=FASN
MIR22	MicroRNA 22	RNA Gene	20	GC17M001713	36.81	https://www.genecards.org/cgi-bin/carddisp.pl?gene=MIR22
MIR30A	MicroRNA 30a	RNA Gene	20	GC06M071403	36.47	https://www.genecards.org/cgi-bin/carddisp.pl?gene=MIR30A
EIF2AK3	Eukaryotic Translation Initiation Factor 2 Alpha Kinas	Protein Coding	48	GC02M088637	36.11	https://www.genecards.org/cgi-bin/carddisp.pl?gene=EIF2AK3
SLC2A4	Solute Carrier Family 2 Member 4	Protein Coding	45	GC17P007295	35.97	https://www.genecards.org/cgi-bin/carddisp.pl?gene=SLC2A4
CASP3	Caspase 3	Protein Coding	50	GC04M184627	35.92	https://www.genecards.org/cgi-bin/carddisp.pl?gene=CASP3
SREBF1	Sterol Regulatory Element Binding Transcription Facto	Protein Coding	44	GC17M017810	35.54	https://www.genecards.org/cgi-bin/carddisp.pl?gene=SREBF1
MIR31	MicroRNA 31	RNA Gene	20	GC09M021513	34.98	https://www.genecards.org/cgi-bin/carddisp.pl?gene=MIR31
AFP	Alpha Fetoprotein	Protein Coding	45	GC04P073431	34.9	https://www.genecards.org/cgi-bin/carddisp.pl?gene=AFP
MIR192	MicroRNA 192	RNA Gene	21	GC11M064891	34.68	https://www.genecards.org/cgi-bin/carddisp.pl?gene=MIR192
MIR27A	MicroRNA 27a	RNA Gene	22	GC19M014010	34.59	https://www.genecards.org/cgi-bin/carddisp.pl?gene=MIR27A
IL1A	Interleukin 1 Alpha	Protein Coding	44	GC02M112773	34.54	https://www.genecards.org/cgi-bin/carddisp.pl?gene=IL1A
CEBPA	CCAAT Enhancer Binding Protein Alpha	Protein Coding	47	GC19M033299	34.47	https://www.genecards.org/cgi-bin/carddisp.pl?gene=CEBPA
MAPK8	Mitogen-Activated Protein Kinase 8	Protein Coding	50	GC10P048306	34.45	https://www.genecards.org/cgi-bin/carddisp.pl?gene=MAPK8
FABP1	Fatty Acid Binding Protein 1	Protein Coding	43	GC02M088122	34.44	https://www.genecards.org/cgi-bin/carddisp.pl?gene=FABP1
SIRT1	Sirtuin 1	Protein Coding	49	GC10P067884	33.54	https://www.genecards.org/cgi-bin/carddisp.pl?gene=SIRT1
MIR29C	MicroRNA 29c	RNA Gene	18	GC01M207802	33.53	https://www.genecards.org/cgi-bin/carddisp.pl?gene=MIR29C
VEGFA	Vascular Endothelial Growth Factor A	Protein Coding	48	GC06P043770	33.2	https://www.genecards.org/cgi-bin/carddisp.pl?gene=VEGFA
MIR200B	MicroRNA 200b	RNA Gene	20	GC01P001167	32.94	https://www.genecards.org/cgi-bin/carddisp.pl?gene=MIR200B

MIR10B	MicroRNA 10b	RNA Gene	21	GC02P176150	32.91	https://www.genecards.org/cgi-bin/carddisp.pl?gene=MIR10B
MIR200A	MicroRNA 200a	RNA Gene	21	GC01P001463	32.85	https://www.genecards.org/cgi-bin/carddisp.pl?gene=MIR200A
MIRLET7D	MicroRNA Let-7d	RNA Gene	21	GC09P094178	32.79	https://www.genecards.org/cgi-bin/carddisp.pl?gene=MIRLET7D
MIR33A	MicroRNA 33a	RNA Gene	18	GC22P041900	32.74	https://www.genecards.org/cgi-bin/carddisp.pl?gene=MIR33A
CCN2	Cellular Communication Network Factor 2	Protein Coding	39	GC06M131948	32.47	https://www.genecards.org/cgi-bin/carddisp.pl?gene=CCN2
MIR200C	MicroRNA 200c	RNA Gene	21	GC12P008229	31.87	https://www.genecards.org/cgi-bin/carddisp.pl?gene=MIR200C
CTNNB1	Catenin Beta 1	Protein Coding	53	GC03P041236	31.86	https://www.genecards.org/cgi-bin/carddisp.pl?gene=CTNNB1
SMPD1	Sphingomyelin Phosphodiesterase 1	Protein Coding	47	GC11P006390	31.73	https://www.genecards.org/cgi-bin/carddisp.pl?gene=SMPD1
MIR183	MicroRNA 183	RNA Gene	18	GC07M129789	31.56	https://www.genecards.org/cgi-bin/carddisp.pl?gene=MIR183
CTLA4	Cytotoxic T-Lymphocyte Associated Protein 4	Protein Coding	45	GC02P203867	31.56	https://www.genecards.org/cgi-bin/carddisp.pl?gene=CTLA4
SERPINA1	Serpin Family A Member 1	Protein Coding	49	GC14M094376	31.54	https://www.genecards.org/cgi-bin/carddisp.pl?gene=SERPINA1
LMNA	Lamin A/C	Protein Coding	47	GC01P156082	31.44	https://www.genecards.org/cgi-bin/carddisp.pl?gene=LMNA
STAT3	Signal Transducer And Activator Of Transcription 3	Protein Coding	52	GC17M042313	31.4	https://www.genecards.org/cgi-bin/carddisp.pl?gene=STAT3
MIR130A	MicroRNA 130a	RNA Gene	20	GC11P057641	30.95	https://www.genecards.org/cgi-bin/carddisp.pl?gene=MIR130A
FABP4	Fatty Acid Binding Protein 4	Protein Coding	44	GC08M081478	30.91	https://www.genecards.org/cgi-bin/carddisp.pl?gene=FABP4
ACE	Angiotensin I Converting Enzyme	Protein Coding	49	GC17P063477	30.9	https://www.genecards.org/cgi-bin/carddisp.pl?gene=ACE
PIK3CA	Phosphatidylinositol-4,5-Bisphosphate 3-Kinase Catal	Protein Coding	52	GC03P179148	30.83	https://www.genecards.org/cgi-bin/carddisp.pl?gene=PIK3CA
MFN2	Mitofusin 2	Protein Coding	48	GC01P011980	30.81	https://www.genecards.org/cgi-bin/carddisp.pl?gene=MFN2
RELA	RELA Proto-Oncogene, NF-KB Subunit	Protein Coding	50	GC11M065653	30.6	https://www.genecards.org/cgi-bin/carddisp.pl?gene=RELA
ATP7B	ATPase Copper Transporting Beta	Protein Coding	47	GC13M051930	30.57	https://www.genecards.org/cgi-bin/carddisp.pl?gene=ATP7B
MIRLET7E	MicroRNA Let-7e	RNA Gene	20	GC19P051718	30.55	https://www.genecards.org/cgi-bin/carddisp.pl?gene=MIRLET7E
GHRL	Ghrelin And Obestatin Prepropeptide	Protein Coding	43	GC03M010285	30.53	https://www.genecards.org/cgi-bin/carddisp.pl?gene=GHRL
MIR107	MicroRNA 107	RNA Gene	19	GC10M089600	30.48	https://www.genecards.org/cgi-bin/carddisp.pl?gene=MIR107
MIR26A1	MicroRNA 26a-1	RNA Gene	20	GC03P037969	30.29	https://www.genecards.org/cgi-bin/carddisp.pl?gene=MIR26A1
ABCA1	ATP Binding Cassette Subfamily A Member 1	Protein Coding	48	GC09M104781	30.24	https://www.genecards.org/cgi-bin/carddisp.pl?gene=ABCA1
NAFLD1	Fatty Liver Disease 1, Susceptiblity To	Genetic Locus	2	GC22U901005	30.23	https://www.genecards.org/cgi-bin/carddisp.pl?gene=NAFLD1
MIR203A	MicroRNA 203a	RNA Gene	19	GC14P104655	30.22	https://www.genecards.org/cgi-bin/carddisp.pl?gene=MIR203A
MTHFR	Methylenetetrahydrofolate Reductase	Protein Coding	47	GC01M011785	29.94	https://www.genecards.org/cgi-bin/carddisp.pl?gene=MTHFR
FASLG	Fas Ligand	Protein Coding	47	GC01P172628	29.64	https://www.genecards.org/cgi-bin/carddisp.pl?gene=FASLG
TM6SF2	Transmembrane 6 Superfamily Member 2	Protein Coding	29	GC19M019264	29.55	https://www.genecards.org/cgi-bin/carddisp.pl?gene=TM6SF2
MSR1	Macrophage Scavenger Receptor 1	Protein Coding	46	GC08M016107	29.52	https://www.genecards.org/cgi-bin/carddisp.pl?gene=MSR1
EGFR	Epidermal Growth Factor Receptor	Protein Coding	54	GC07P055019	29.5	https://www.genecards.org/cgi-bin/carddisp.pl?gene=EGFR
MIR16-1	MicroRNA 16-1	RNA Gene	21	GC13M050048	29.49	https://www.genecards.org/cgi-bin/carddisp.pl?gene=MIR16-1
MARS1	Methionyl-TRNA Synthetase 1	Protein Coding	35	GC12P057476	29.42	https://www.genecards.org/cgi-bin/carddisp.pl?gene=MARS1
XBP1	X-Box Binding Protein 1	Protein Coding	45	GC22M028794	29.21	https://www.genecards.org/cgi-bin/carddisp.pl?gene=XBP1
MIR125A	MicroRNA 125a	RNA Gene	21	GC19P051720	28.9	https://www.genecards.org/cgi-bin/carddisp.pl?gene=MIR125A
MMP9	Matrix Metallopeptidase 9	Protein Coding	52	GC20P046008	28.83	https://www.genecards.org/cgi-bin/carddisp.pl?gene=MMP9
ICAM1	Intercellular Adhesion Molecule 1	Protein Coding	50	GC19P010270	28.78	https://www.genecards.org/cgi-bin/carddisp.pl?gene=ICAM1
SOD1	Superoxide Dismutase 1	Protein Coding	51	GC21P031659	28.74	https://www.genecards.org/cgi-bin/carddisp.pl?gene=SOD1

CFTR	CF Transmembrane Conductance Regulator	Protein Coding	51	GC07P117287	28.74	https://www.genecards.org/cgi-bin/carddisp.pl?gene=CFTR
MIRLET7C	MicroRNA Let-7c	RNA Gene	21	GC21P016551	28.66	https://www.genecards.org/cgi-bin/carddisp.pl?gene=MIRLET7C
LIPA	Lipase A, Lysosomal Acid Type	Protein Coding	47	GC10M089213	28.37	https://www.genecards.org/cgi-bin/carddisp.pl?gene=LIPA
TNFRSF1A	TNF Receptor Superfamily Member 1A	Protein Coding	49	GC12M006328	28.23	https://www.genecards.org/cgi-bin/carddisp.pl?gene=TNFRSF1A
APP	Amyloid Beta Precursor Protein	Protein Coding	51	GC21M025880	28.14	https://www.genecards.org/cgi-bin/carddisp.pl?gene=APP
ACOX1	Acyl-CoA Oxidase 1	Protein Coding	45	GC17M075941	28.09	https://www.genecards.org/cgi-bin/carddisp.pl?gene=ACOX1
CPT1A	Carnitine Palmitoyltransferase 1A	Protein Coding	48	GC11M068754	28.09	https://www.genecards.org/cgi-bin/carddisp.pl?gene=CPT1A
MIR451A	MicroRNA 451a	RNA Gene	17	GC17M028861	28.04	https://www.genecards.org/cgi-bin/carddisp.pl?gene=MIR451A
NDUFS1	NADH:Ubiquinone Oxidoreductase Core Subunit S1	Protein Coding	45	GC02M206114	28	https://www.genecards.org/cgi-bin/carddisp.pl?gene=NDUFS1
ABCB4	ATP Binding Cassette Subfamily B Member 4	Protein Coding	45	GC07M087401	27.87	https://www.genecards.org/cgi-bin/carddisp.pl?gene=ABCB4
ADIPOR1	Adiponectin Receptor 1	Protein Coding	44	GC01M202940	27.84	https://www.genecards.org/cgi-bin/carddisp.pl?gene=ADIPOR1
CAV1	Caveolin 1	Protein Coding	48	GC07P116524	27.79	https://www.genecards.org/cgi-bin/carddisp.pl?gene=CAV1
TIMP1	TIMP Metallopeptidase Inhibitor 1	Protein Coding	45	GC0XP047583	27.46	https://www.genecards.org/cgi-bin/carddisp.pl?gene=TIMP1
MIR30D	MicroRNA 30d	RNA Gene	17	GC08M134804	27.33	https://www.genecards.org/cgi-bin/carddisp.pl?gene=MIR30D
IL18	Interleukin 18	Protein Coding	44	GC11M112143	27.32	https://www.genecards.org/cgi-bin/carddisp.pl?gene=IL18
ADIPOR2	Adiponectin Receptor 2	Protein Coding	43	GC12P001670	27.28	https://www.genecards.org/cgi-bin/carddisp.pl?gene=ADIPOR2
MIR378A	MicroRNA 378a	RNA Gene	18	GC05P149732	27.24	https://www.genecards.org/cgi-bin/carddisp.pl?gene=MIR378A
STAT1	Signal Transducer And Activator Of Transcription 1	Protein Coding	53	GC02M190908	27.2	https://www.genecards.org/cgi-bin/carddisp.pl?gene=STAT1
JUN	Jun Proto-Oncogene, AP-1 Transcription Factor Subun	Protein Coding	49	GC01M058780	26.99	https://www.genecards.org/cgi-bin/carddisp.pl?gene=JUN
MIR483	MicroRNA 483	RNA Gene	18	GC11M002188	26.98	https://www.genecards.org/cgi-bin/carddisp.pl?gene=MIR483
CASP8	Caspase 8	Protein Coding	52	GC02P201233	26.88	https://www.genecards.org/cgi-bin/carddisp.pl?gene=CASP8
CP	Ceruloplasmin	Protein Coding	47	GC03M149162	26.83	https://www.genecards.org/cgi-bin/carddisp.pl?gene=CP
LCAT	Lecithin-Cholesterol Acyltransferase	Protein Coding	47	GC16M067939	26.82	https://www.genecards.org/cgi-bin/carddisp.pl?gene=LCAT
MAP3K5	Mitogen-Activated Protein Kinase Kinase Kinase 5	Protein Coding	47	GC06M136557	26.78	https://www.genecards.org/cgi-bin/carddisp.pl?gene=MAP3K5
CDKN2A	Cyclin Dependent Kinase Inhibitor 2A	Protein Coding	51	GC09M021967	26.72	https://www.genecards.org/cgi-bin/carddisp.pl?gene=CDKN2A
MMP2	Matrix Metallopeptidase 2	Protein Coding	53	GC16P055390	26.56	https://www.genecards.org/cgi-bin/carddisp.pl?gene=MMP2
MT-TK	Mitochondrially Encoded TRNA-Lys (AAA/G)	RNA Gene	14	GCMTP008297	26.54	https://www.genecards.org/cgi-bin/carddisp.pl?gene=MT-TK
ATF4	Activating Transcription Factor 4	Protein Coding	46	GC22P039525	26.39	https://www.genecards.org/cgi-bin/carddisp.pl?gene=ATF4
CYCS	Cytochrome C, Somatic	Protein Coding	48	GC07M025118	26.35	https://www.genecards.org/cgi-bin/carddisp.pl?gene=CYCS
HMGCR	3-Hydroxy-3-Methylglutaryl-CoA Reductase	Protein Coding	45	GC05P075336	26.33	https://www.genecards.org/cgi-bin/carddisp.pl?gene=HMGCR
TTR	Transthyretin	Protein Coding	49	GC18P031557	26.32	https://www.genecards.org/cgi-bin/carddisp.pl?gene=TTR
NOTCH1	Notch Receptor 1	Protein Coding	51	GC09M136602	26.32	https://www.genecards.org/cgi-bin/carddisp.pl?gene=NOTCH1
TF	Transferrin	Protein Coding	49	GC03P133666	26.01	https://www.genecards.org/cgi-bin/carddisp.pl?gene=TF
MPO	Myeloperoxidase	Protein Coding	50	GC17M058269	25.94	https://www.genecards.org/cgi-bin/carddisp.pl?gene=MPO
MYC	MYC Proto-Oncogene, BHLH Transcription Factor	Protein Coding	51	GC08P127735	25.92	https://www.genecards.org/cgi-bin/carddisp.pl?gene=MYC
FGF21	Fibroblast Growth Factor 21	Protein Coding	40	GC19P048766	25.91	https://www.genecards.org/cgi-bin/carddisp.pl?gene=FGF21
LDLR	Low Density Lipoprotein Receptor	Protein Coding	49	GC19P011061	25.85	https://www.genecards.org/cgi-bin/carddisp.pl?gene=LDLR
TERT	Telomerase Reverse Transcriptase	Protein Coding	51	GC05M001253	25.67	https://www.genecards.org/cgi-bin/carddisp.pl?gene=TERT
SREBF2	Sterol Regulatory Element Binding Transcription Facto	Protein Coding	43	GC22P041833	25.47	https://www.genecards.org/cgi-bin/carddisp.pl?gene=SREBF2

NAFLD2	Fatty Liver Disease, Nonalcoholic, Susceptibility To, 2	Genetic Locus	2	GC11U901533	25.45	https://www.genecards.org/cgi-bin/carddisp.pl?gene=NAFLD2
PON1	Paraoxonase 1	Protein Coding	45	GC07M095297	25.43	https://www.genecards.org/cgi-bin/carddisp.pl?gene=PON1
IL1RN	Interleukin 1 Receptor Antagonist	Protein Coding	48	GC02P115307	25.34	https://www.genecards.org/cgi-bin/carddisp.pl?gene=IL1RN
NFKB1	Nuclear Factor Kappa B Subunit 1	Protein Coding	52	GC04P102501	25.07	https://www.genecards.org/cgi-bin/carddisp.pl?gene=NFKB1
EGF	Epidermal Growth Factor	Protein Coding	51	GC04P109912	25.05	https://www.genecards.org/cgi-bin/carddisp.pl?gene=EGF
VWF	Von Willebrand Factor	Protein Coding	48	GC12M005917	25.04	https://www.genecards.org/cgi-bin/carddisp.pl?gene=VWF
SMAD4	SMAD Family Member 4	Protein Coding	50	GC18P051028	24.89	https://www.genecards.org/cgi-bin/carddisp.pl?gene=SMAD4
JAG1	Jagged Canonical Notch Ligand 1	Protein Coding	50	GC20M010637	24.62	https://www.genecards.org/cgi-bin/carddisp.pl?gene=JAG1
SQSTM1	Sequestosome 1	Protein Coding	48	GC05P179806	24.54	https://www.genecards.org/cgi-bin/carddisp.pl?gene=SQSTM1
MIR429	MicroRNA 429	RNA Gene	19	GC01P001464	24.18	https://www.genecards.org/cgi-bin/carddisp.pl?gene=MIR429
SLC25A13	Solute Carrier Family 25 Member 13	Protein Coding	46	GC07M096120	24.07	https://www.genecards.org/cgi-bin/carddisp.pl?gene=SLC25A13
PIK3R1	Phosphoinositide-3-Kinase Regulatory Subunit 1	Protein Coding	51	GC05P068215	24.02	https://www.genecards.org/cgi-bin/carddisp.pl?gene=PIK3R1
ESR1	Estrogen Receptor 1	Protein Coding	53	GC06P151656	23.91	https://www.genecards.org/cgi-bin/carddisp.pl?gene=ESR1
CCND1	Cyclin D1	Protein Coding	52	GC11P069641	23.86	https://www.genecards.org/cgi-bin/carddisp.pl?gene=CCND1
NOS2	Nitric Oxide Synthase 2	Protein Coding	49	GC17M027756	23.83	https://www.genecards.org/cgi-bin/carddisp.pl?gene=NOS2
BSCL2	BSCL2 Lipid Droplet Biogenesis Associated, Seipin	Protein Coding	42	GC11M063438	23.78	https://www.genecards.org/cgi-bin/carddisp.pl?gene=BSCL2
AKT2	AKT Serine/Threonine Kinase 2	Protein Coding	54	GC19M040230	23.61	https://www.genecards.org/cgi-bin/carddisp.pl?gene=AKT2
CETP	Cholesteryl Ester Transfer Protein	Protein Coding	46	GC16P056961	23.61	https://www.genecards.org/cgi-bin/carddisp.pl?gene=CETP
VCP	Valosin Containing Protein	Protein Coding	48	GC09M035056	23.59	https://www.genecards.org/cgi-bin/carddisp.pl?gene=VCP
GANAB	Glucosidase II Alpha Subunit	Protein Coding	44	GC11M063430	23.55	https://www.genecards.org/cgi-bin/carddisp.pl?gene=GANAB
G6PC	Glucose-6-Phosphatase Catalytic Subunit	Protein Coding	44	GC17P042900	23.37	https://www.genecards.org/cgi-bin/carddisp.pl?gene=G6PC
SPP1	Secreted Phosphoprotein 1	Protein Coding	46	GC04P087975	23.32	https://www.genecards.org/cgi-bin/carddisp.pl?gene=SPP1
SERPINC1	Serpin Family C Member 1	Protein Coding	48	GC01M174153	23.18	https://www.genecards.org/cgi-bin/carddisp.pl?gene=SERPINC1
GCG	Glucagon	Protein Coding	41	GC02M162142	23.09	https://www.genecards.org/cgi-bin/carddisp.pl?gene=GCG
BAX	BCL2 Associated X, Apoptosis Regulator	Protein Coding	48	GC19P048954	22.98	https://www.genecards.org/cgi-bin/carddisp.pl?gene=BAX
HAMP	Hepcidin Antimicrobial Peptide	Protein Coding	44	GC19P038216	22.95	https://www.genecards.org/cgi-bin/carddisp.pl?gene=HAMP
ABCC2	ATP Binding Cassette Subfamily C Member 2	Protein Coding	47	GC10P099782	22.91	https://www.genecards.org/cgi-bin/carddisp.pl?gene=ABCC2
HLA-DQB1	Major Histocompatibility Complex, Class II, DQ Beta	Protein Coding	44	GC06M032804	22.64	https://www.genecards.org/cgi-bin/carddisp.pl?gene=HLA-DQB1
BDNF	Brain Derived Neurotrophic Factor	Protein Coding	47	GC11M027654	22.63	https://www.genecards.org/cgi-bin/carddisp.pl?gene=BDNF
VIM	Vimentin	Protein Coding	50	GC10P017227	22.61	https://www.genecards.org/cgi-bin/carddisp.pl?gene=VIM
CYP3A4	Cytochrome P450 Family 3 Subfamily A Member 4	Protein Coding	48	GC07M099759	22.59	https://www.genecards.org/cgi-bin/carddisp.pl?gene=CYP3A4
PTPN11	Protein Tyrosine Phosphatase Non-Receptor Type 11	Protein Coding	53	GC12P112418	22.48	https://www.genecards.org/cgi-bin/carddisp.pl?gene=PTPN11
GCKR	Glucokinase Regulator	Protein Coding	40	GC02P027496	22.36	https://www.genecards.org/cgi-bin/carddisp.pl?gene=GCKR
ELANE	Elastase, Neutrophil Expressed	Protein Coding	46	GC19P000854	22.29	https://www.genecards.org/cgi-bin/carddisp.pl?gene=ELANE
DPP4	Dipeptidyl Peptidase 4	Protein Coding	50	GC02M161992	22.16	https://www.genecards.org/cgi-bin/carddisp.pl?gene=DPP4
MMP1	Matrix Metallopeptidase 1	Protein Coding	51	GC11M102810	22.13	https://www.genecards.org/cgi-bin/carddisp.pl?gene=MMP1
CDKN1A	Cyclin Dependent Kinase Inhibitor 1A	Protein Coding	50	GC06P047460	22.12	https://www.genecards.org/cgi-bin/carddisp.pl?gene=CDKN1A
NDUFS4	NADH:Ubiquinone Oxidoreductase Subunit S4	Protein Coding	44	GC05P053560	21.72	https://www.genecards.org/cgi-bin/carddisp.pl?gene=NDUFS4
CXCL10	C-X-C Motif Chemokine Ligand 10	Protein Coding	44	GC04M076021	21.67	https://www.genecards.org/cgi-bin/carddisp.pl?gene=CXCL10

HSPB1	Heat Shock Protein Family B (Small) Member 1	Protein Coding	51	GC07P076302	21.54	https://www.genecards.org/cgi-bin/carddisp.pl?gene=HSPB1
MT-CO1	Mitochondrially Encoded Cytochrome C Oxidase I	Protein Coding	32	GCMTP005906	21.52	https://www.genecards.org/cgi-bin/carddisp.pl?gene=MT-CO1
PCSK9	Proprotein Convertase Subtilisin/Kexin Type 9	Protein Coding	51	GC01P055039	21.39	https://www.genecards.org/cgi-bin/carddisp.pl?gene=PCSK9
POMC	Proopiomelanocortin	Protein Coding	48	GC02M025160	21.37	https://www.genecards.org/cgi-bin/carddisp.pl?gene=POMC
MIR199A1	MicroRNA 199a-1	RNA Gene	18	GC19M010792	21.3	https://www.genecards.org/cgi-bin/carddisp.pl?gene=MIR199A1
MIR181B1	MicroRNA 181b-1	RNA Gene	19	GC01M198858	21.27	https://www.genecards.org/cgi-bin/carddisp.pl?gene=MIR181B1
LIPC	Lipase C, Hepatic Type	Protein Coding	44	GC15P058410	21.22	https://www.genecards.org/cgi-bin/carddisp.pl?gene=LIPC
PRMT7	Protein Arginine Methyltransferase 7	Protein Coding	44	GC16P068339	21.17	https://www.genecards.org/cgi-bin/carddisp.pl?gene=PRMT7
PRKAG2	Protein Kinase AMP-Activated Non-Catalytic Subunit	Protein Coding	50	GC07M151556	20.96	https://www.genecards.org/cgi-bin/carddisp.pl?gene=PRKAG2
CXCR4	C-X-C Motif Chemokine Receptor 4	Protein Coding	52	GC02M136114	20.95	https://www.genecards.org/cgi-bin/carddisp.pl?gene=CXCR4
TNFRSF1B	TNF Receptor Superfamily Member 1B	Protein Coding	47	GC01P012167	20.91	https://www.genecards.org/cgi-bin/carddisp.pl?gene=TNFRSF1B
SLC2A1	Solute Carrier Family 2 Member 1	Protein Coding	52	GC01M042925	20.86	https://www.genecards.org/cgi-bin/carddisp.pl?gene=SLC2A1
NOD2	Nucleotide Binding Oligomerization Domain Containi	Protein Coding	48	GC16P050693	20.75	https://www.genecards.org/cgi-bin/carddisp.pl?gene=NOD2
THBD	Thrombomodulin	Protein Coding	44	GC20M023026	20.7	https://www.genecards.org/cgi-bin/carddisp.pl?gene=THBD
NAMPT	Nicotinamide Phosphoribosyltransferase	Protein Coding	48	GC07M106248	20.7	https://www.genecards.org/cgi-bin/carddisp.pl?gene=NAMPT
NLRP3	NLR Family Pyrin Domain Containing 3	Protein Coding	47	GC01P247415	20.61	https://www.genecards.org/cgi-bin/carddisp.pl?gene=NLRP3
BMP6	Bone Morphogenetic Protein 6	Protein Coding	43	GC06P007726	20.56	https://www.genecards.org/cgi-bin/carddisp.pl?gene=BMP6
PNPLA2	Patatin Like Phospholipase Domain Containing 2	Protein Coding	44	GC11P000896	20.52	https://www.genecards.org/cgi-bin/carddisp.pl?gene=PNPLA2
MT-CYB	Mitochondrially Encoded Cytochrome B	Protein Coding	31	GCMTP014749	20.46	https://www.genecards.org/cgi-bin/carddisp.pl?gene=MT-CYB
PDGFRB	Platelet Derived Growth Factor Receptor Beta	Protein Coding	55	GC05M150113	20.44	https://www.genecards.org/cgi-bin/carddisp.pl?gene=PDGFRB
SDHB	Succinate Dehydrogenase Complex Iron Sulfur Subuni	Protein Coding	47	GC01M017020	20.44	https://www.genecards.org/cgi-bin/carddisp.pl?gene=SDHB
IGF1R	Insulin Like Growth Factor 1 Receptor	Protein Coding	54	GC15P098648	20.4	https://www.genecards.org/cgi-bin/carddisp.pl?gene=IGF1R
NGF	Nerve Growth Factor	Protein Coding	50	GC01M115285	20.34	https://www.genecards.org/cgi-bin/carddisp.pl?gene=NGF
CRYAA	Crystallin Alpha A	Protein Coding	44	GC21P043169	20.31	https://www.genecards.org/cgi-bin/carddisp.pl?gene=CRYAA
ACTB	Actin Beta	Protein Coding	49	GC07M005527	20.28	https://www.genecards.org/cgi-bin/carddisp.pl?gene=ACTB
PKLR	Pyruvate Kinase L/R	Protein Coding	47	GC01M155289	20.22	https://www.genecards.org/cgi-bin/carddisp.pl?gene=PKLR
HSPD1	Heat Shock Protein Family D (Hsp60) Member 1	Protein Coding	47	GC02M197486	20.2	https://www.genecards.org/cgi-bin/carddisp.pl?gene=HSPD1
SCD	Stearoyl-CoA Desaturase	Protein Coding	48	GC10P100347	20.2	https://www.genecards.org/cgi-bin/carddisp.pl?gene=SCD
BCL2	BCL2 Apoptosis Regulator	Protein Coding	51	GC18M063123	20.17	https://www.genecards.org/cgi-bin/carddisp.pl?gene=BCL2
PTGS2	Prostaglandin-Endoperoxide Synthase 2	Protein Coding	48	GC01M186640	19.98	https://www.genecards.org/cgi-bin/carddisp.pl?gene=PTGS2
TLR9	Toll Like Receptor 9	Protein Coding	45	GC03M052222	19.95	https://www.genecards.org/cgi-bin/carddisp.pl?gene=TLR9
SERPINA3	Serpin Family A Member 3	Protein Coding	43	GC14P094612	19.8	https://www.genecards.org/cgi-bin/carddisp.pl?gene=SERPINA3
ERN1	Endoplasmic Reticulum To Nucleus Signaling 1	Protein Coding	45	GC17M064039	19.72	https://www.genecards.org/cgi-bin/carddisp.pl?gene=ERN1
C3	Complement C3	Protein Coding	47	GC19M006677	19.67	https://www.genecards.org/cgi-bin/carddisp.pl?gene=C3
TLR1	Toll Like Receptor 1	Protein Coding	47	GC04M038797	19.64	https://www.genecards.org/cgi-bin/carddisp.pl?gene=TLR1
SDHD	Succinate Dehydrogenase Complex Subunit D	Protein Coding	44	GC11P112087	19.58	https://www.genecards.org/cgi-bin/carddisp.pl?gene=SDHD
TGFBR2	Transforming Growth Factor Beta Receptor 2	Protein Coding	51	GC03P030623	19.48	https://www.genecards.org/cgi-bin/carddisp.pl?gene=TGFBR2
DRD2	Dopamine Receptor D2	Protein Coding	50	GC11M113409	19.46	https://www.genecards.org/cgi-bin/carddisp.pl?gene=DRD2
TLR6	Toll Like Receptor 6	Protein Coding	44	GC04M038828	19.29	https://www.genecards.org/cgi-bin/carddisp.pl?gene=TLR6

NDUFS3	NADH:Ubiquinone Oxidoreductase Core Subunit S3	Protein Coding	46	GC11P047567	19.27	https://www.genecards.org/cgi-bin/carddisp.pl?gene=NDUFS3
CFH	Complement Factor H	Protein Coding	45	GC01P196621	19.24	https://www.genecards.org/cgi-bin/carddisp.pl?gene=CFH
SLC6A3	Solute Carrier Family 6 Member 3	Protein Coding	49	GC05M001392	19.22	https://www.genecards.org/cgi-bin/carddisp.pl?gene=SLC6A3
TNFSF11	TNF Superfamily Member 11	Protein Coding	47	GC13P042562	19.21	https://www.genecards.org/cgi-bin/carddisp.pl?gene=TNFSF11
ENPP1	Ectonucleotide Pyrophosphatase/Phosphodiesterase 1	Protein Coding	47	GC06P131808	19.11	https://www.genecards.org/cgi-bin/carddisp.pl?gene=ENPP1
MIR146A	MicroRNA 146a	RNA Gene	22	GC05P160485	18.96	https://www.genecards.org/cgi-bin/carddisp.pl?gene=MIR146A
LIPE	Lipase E, Hormone Sensitive Type	Protein Coding	47	GC19M042401	18.95	https://www.genecards.org/cgi-bin/carddisp.pl?gene=LIPE
UGT1A1	UDP Glucuronosyltransferase Family 1 Member A1	Protein Coding	48	GC02P233760	18.9	https://www.genecards.org/cgi-bin/carddisp.pl?gene=UGT1A1
FGF19	Fibroblast Growth Factor 19	Protein Coding	44	GC11M069699	18.86	https://www.genecards.org/cgi-bin/carddisp.pl?gene=FGF19
NDUFS2	NADH:Ubiquinone Oxidoreductase Core Subunit S2	Protein Coding	44	GC01P161197	18.7	https://www.genecards.org/cgi-bin/carddisp.pl?gene=NDUFS2
AGPAT2	1-Acylglycerol-3-Phosphate O-Acyltransferase 2	Protein Coding	45	GC09M136673	18.68	https://www.genecards.org/cgi-bin/carddisp.pl?gene=AGPAT2
MDM2	MDM2 Proto-Oncogene	Protein Coding	52	GC12P068808	18.67	https://www.genecards.org/cgi-bin/carddisp.pl?gene=MDM2
CCL3	C-C Motif Chemokine Ligand 3	Protein Coding	39	GC17M036088	18.58	https://www.genecards.org/cgi-bin/carddisp.pl?gene=CCL3
ABCB7	ATP Binding Cassette Subfamily B Member 7	Protein Coding	43	GC0XM075053	18.58	https://www.genecards.org/cgi-bin/carddisp.pl?gene=ABCB7
DNMT1	DNA Methyltransferase 1	Protein Coding	50	GC19M010133	18.55	https://www.genecards.org/cgi-bin/carddisp.pl?gene=DNMT1
NDUFB8	NADH:Ubiquinone Oxidoreductase Subunit B8	Protein Coding	43	GC10M100523	18.54	https://www.genecards.org/cgi-bin/carddisp.pl?gene=NDUFB8
DNAJB11	DnaJ Heat Shock Protein Family (Hsp40) Member B11	Protein Coding	40	GC03P186567	18.46	https://www.genecards.org/cgi-bin/carddisp.pl?gene=DNAJB11
SDHC	Succinate Dehydrogenase Complex Subunit C	Protein Coding	44	GC01P161314	18.41	https://www.genecards.org/cgi-bin/carddisp.pl?gene=SDHC
CDK4	Cyclin Dependent Kinase 4	Protein Coding	54	GC12M057743	18.36	https://www.genecards.org/cgi-bin/carddisp.pl?gene=CDK4
FLT1	Fms Related Receptor Tyrosine Kinase 1	Protein Coding	51	GC13M028300	18.29	https://www.genecards.org/cgi-bin/carddisp.pl?gene=FLT1
ASS1	Argininosuccinate Synthase 1	Protein Coding	48	GC09P130444	18.18	https://www.genecards.org/cgi-bin/carddisp.pl?gene=ASS1
LPA	Lipoprotein(A)	Protein Coding	41	GC06M160531	18.16	https://www.genecards.org/cgi-bin/carddisp.pl?gene=LPA
SLC6A4	Solute Carrier Family 6 Member 4	Protein Coding	47	GC17M030194	18.16	https://www.genecards.org/cgi-bin/carddisp.pl?gene=SLC6A4
SDHA	Succinate Dehydrogenase Complex Flavoprotein Subu	Protein Coding	46	GC05P000208	18.09	https://www.genecards.org/cgi-bin/carddisp.pl?gene=SDHA
FN1	Fibronectin 1	Protein Coding	50	GC02M215360	18.05	https://www.genecards.org/cgi-bin/carddisp.pl?gene=FN1
IFNA1	Interferon Alpha 1	Protein Coding	39	GC09P021494	17.95	https://www.genecards.org/cgi-bin/carddisp.pl?gene=IFNA1
PRKAB1	Protein Kinase AMP-Activated Non-Catalytic Subunit	Protein Coding	47	GC12P119632	17.87	https://www.genecards.org/cgi-bin/carddisp.pl?gene=PRKAB1
PRKAA1	Protein Kinase AMP-Activated Catalytic Subunit Alph	Protein Coding	47	GC05M040759	17.74	https://www.genecards.org/cgi-bin/carddisp.pl?gene=PRKAA1
NDUFV1	NADH:Ubiquinone Oxidoreductase Core Subunit V1	Protein Coding	45	GC11P067632	17.65	https://www.genecards.org/cgi-bin/carddisp.pl?gene=NDUFV1
NDUFS7	NADH:Ubiquinone Oxidoreductase Core Subunit S7	Protein Coding	46	GC19P001490	17.63	https://www.genecards.org/cgi-bin/carddisp.pl?gene=NDUFS7
CTSD	Cathepsin D	Protein Coding	52	GC11M001752	17.61	https://www.genecards.org/cgi-bin/carddisp.pl?gene=CTSD
CDKN3	Cyclin Dependent Kinase Inhibitor 3	Protein Coding	42	GC14P054398	17.57	https://www.genecards.org/cgi-bin/carddisp.pl?gene=CDKN3
APOA5	Apolipoprotein A5	Protein Coding	44	GC11M116789	17.55	https://www.genecards.org/cgi-bin/carddisp.pl?gene=APOA5
IL17A	Interleukin 17A	Protein Coding	42	GC06P052186	17.52	https://www.genecards.org/cgi-bin/carddisp.pl?gene=IL17A
TARDBP	TAR DNA Binding Protein	Protein Coding	45	GC01P011013	17.46	https://www.genecards.org/cgi-bin/carddisp.pl?gene=TARDBP
CYP7A1	Cytochrome P450 Family 7 Subfamily A Member 1	Protein Coding	42	GC08M058476	17.44	https://www.genecards.org/cgi-bin/carddisp.pl?gene=CYP7A1
GRN	Granulin Precursor	Protein Coding	47	GC17P044345	17.41	https://www.genecards.org/cgi-bin/carddisp.pl?gene=GRN
AKT3	AKT Serine/Threonine Kinase 3	Protein Coding	52	GC01M243488	17.36	https://www.genecards.org/cgi-bin/carddisp.pl?gene=AKT3
CASR	Calcium Sensing Receptor	Protein Coding	50	GC03P122183	17.35	https://www.genecards.org/cgi-bin/carddisp.pl?gene=CASR

PLAU	Plasminogen Activator, Urokinase	Protein Coding	51	GC10P073909	17.32	https://www.genecards.org/cgi-bin/carddisp.pl?gene=PLAU
GCK	Glucokinase	Protein Coding	50	GC07M044145	17.2	https://www.genecards.org/cgi-bin/carddisp.pl?gene=GCK
KRT8	Keratin 8	Protein Coding	47	GC12M052897	17.15	https://www.genecards.org/cgi-bin/carddisp.pl?gene=KRT8
ANO5	Anoctamin 5	Protein Coding	38	GC11P021799	17.13	https://www.genecards.org/cgi-bin/carddisp.pl?gene=ANO5
TGFBR1	Transforming Growth Factor Beta Receptor 1	Protein Coding	52	GC09P099104	17.12	https://www.genecards.org/cgi-bin/carddisp.pl?gene=TGFBR1
EZH2	Enhancer Of Zeste 2 Polycomb Repressive Complex 2	Protein Coding	54	GC07M148807	17.09	https://www.genecards.org/cgi-bin/carddisp.pl?gene=EZH2
IFIH1	Interferon Induced With Helicase C Domain 1	Protein Coding	47	GC02M162267	16.93	https://www.genecards.org/cgi-bin/carddisp.pl?gene=IFIH1
MT-CO2	Mitochondrially Encoded Cytochrome C Oxidase II	Protein Coding	32	GCMTP007587	16.92	https://www.genecards.org/cgi-bin/carddisp.pl?gene=MT-CO2
PC	Pyruvate Carboxylase	Protein Coding	47	GC11M066848	16.83	https://www.genecards.org/cgi-bin/carddisp.pl?gene=PC
XDH	Xanthine Dehydrogenase	Protein Coding	47	GC02M031294	16.82	https://www.genecards.org/cgi-bin/carddisp.pl?gene=XDH
ALMS1	ALMS1 Centrosome And Basal Body Associated Prote	Protein Coding	41	GC02P073385	16.78	https://www.genecards.org/cgi-bin/carddisp.pl?gene=ALMS1
MYD88	MYD88 Innate Immune Signal Transduction Adaptor	Protein Coding	50	GC03P038179	16.75	https://www.genecards.org/cgi-bin/carddisp.pl?gene=MYD88
GSR	Glutathione-Disulfide Reductase	Protein Coding	48	GC08M030678	16.67	https://www.genecards.org/cgi-bin/carddisp.pl?gene=GSR
FABP2	Fatty Acid Binding Protein 2	Protein Coding	43	GC04M119317	16.57	https://www.genecards.org/cgi-bin/carddisp.pl?gene=FABP2
NDUFV2	NADH:Ubiquinone Oxidoreductase Core Subunit V2	Protein Coding	44	GC18P009092	16.51	https://www.genecards.org/cgi-bin/carddisp.pl?gene=NDUFV2
MIR195	MicroRNA 195	RNA Gene	19	GC17M007018	16.44	https://www.genecards.org/cgi-bin/carddisp.pl?gene=MIR195
GH1	Growth Hormone 1	Protein Coding	44	GC17M063917	16.43	https://www.genecards.org/cgi-bin/carddisp.pl?gene=GH1
MIR151A	MicroRNA 151a	RNA Gene	17	GC08M140733	16.43	https://www.genecards.org/cgi-bin/carddisp.pl?gene=MIR151A
CHUK	Component Of Inhibitor Of Nuclear Factor Kappa B K	Protein Coding	52	GC10M100188	16.32	https://www.genecards.org/cgi-bin/carddisp.pl?gene=CHUK
HBB	Hemoglobin Subunit Beta	Protein Coding	45	GC11M005352	16.3	https://www.genecards.org/cgi-bin/carddisp.pl?gene=HBB
SMARCA4	SWI/SNF Related, Matrix Associated, Actin Depender	Protein Coding	50	GC19P010932	16.28	https://www.genecards.org/cgi-bin/carddisp.pl?gene=SMARCA4
CYP1A1	Cytochrome P450 Family 1 Subfamily A Member 1	Protein Coding	47	GC15M074719	16.27	https://www.genecards.org/cgi-bin/carddisp.pl?gene=CYP1A1
SIRT3	Sirtuin 3	Protein Coding	48	GC11M000215	16.26	https://www.genecards.org/cgi-bin/carddisp.pl?gene=SIRT3
CCL4	C-C Motif Chemokine Ligand 4	Protein Coding	40	GC17P036103	16.21	https://www.genecards.org/cgi-bin/carddisp.pl?gene=CCL4
FOS	Fos Proto-Oncogene, AP-1 Transcription Factor Subun	Protein Coding	50	GC14P075278	16.19	https://www.genecards.org/cgi-bin/carddisp.pl?gene=FOS
NDUFA13	NADH:Ubiquinone Oxidoreductase Subunit A13	Protein Coding	44	GC19P019515	16.07	https://www.genecards.org/cgi-bin/carddisp.pl?gene=NDUFA13
MEG3	Maternally Expressed 3	RNA Gene	29	GC14P104771	16.05	https://www.genecards.org/cgi-bin/carddisp.pl?gene=MEG3
MIR20A	MicroRNA 20a	RNA Gene	19	GC13P091434	16.05	https://www.genecards.org/cgi-bin/carddisp.pl?gene=MIR20A
FGF23	Fibroblast Growth Factor 23	Protein Coding	45	GC12M004368	16	https://www.genecards.org/cgi-bin/carddisp.pl?gene=FGF23
EP300	E1A Binding Protein P300	Protein Coding	50	GC22P041091	15.96	https://www.genecards.org/cgi-bin/carddisp.pl?gene=EP300
PIK3R2	Phosphoinositide-3-Kinase Regulatory Subunit 2	Protein Coding	49	GC19P018153	15.93	https://www.genecards.org/cgi-bin/carddisp.pl?gene=PIK3R2
NDUFA9	NADH:Ubiquinone Oxidoreductase Subunit A9	Protein Coding	43	GC12P004649	15.86	https://www.genecards.org/cgi-bin/carddisp.pl?gene=NDUFA9
NDUFB9	NADH:Ubiquinone Oxidoreductase Subunit B9	Protein Coding	44	GC08P124539	15.85	https://www.genecards.org/cgi-bin/carddisp.pl?gene=NDUFB9
MIR181A1	MicroRNA 181a-1	RNA Gene	18	GC01M198860	15.84	https://www.genecards.org/cgi-bin/carddisp.pl?gene=MIR181A1
NDUFA6	NADH:Ubiquinone Oxidoreductase Subunit A6	Protein Coding	44	GC22M042085	15.84	https://www.genecards.org/cgi-bin/carddisp.pl?gene=NDUFA6
VDR	Vitamin D Receptor	Protein Coding	51	GC12M047841	15.83	https://www.genecards.org/cgi-bin/carddisp.pl?gene=VDR
LOX	Lysyl Oxidase	Protein Coding	44	GC05M122063	15.82	https://www.genecards.org/cgi-bin/carddisp.pl?gene=LOX
CTSB	Cathepsin B	Protein Coding	51	GC08M011842	15.81	https://www.genecards.org/cgi-bin/carddisp.pl?gene=CTSB
GSK3B	Glycogen Synthase Kinase 3 Beta	Protein Coding	50	GC03M119821	15.79	https://www.genecards.org/cgi-bin/carddisp.pl?gene=GSK3B

AKR1D1	Aldo-Keto Reductase Family 1 Member D1	Protein Coding	46	GC07P138003	15.78	https://www.genecards.org/cgi-bin/carddisp.pl?gene=AKR1D1
IDH1	Isocitrate Dehydrogenase (NADP(+)) 1	Protein Coding	52	GC02M208236	15.76	https://www.genecards.org/cgi-bin/carddisp.pl?gene=IDH1
NR1H2	Nuclear Receptor Subfamily 1 Group H Member 2	Protein Coding	48	GC19P050329	15.76	https://www.genecards.org/cgi-bin/carddisp.pl?gene=NR1H2
MIR144	MicroRNA 144	RNA Gene	16	GC17M029965	15.73	https://www.genecards.org/cgi-bin/carddisp.pl?gene=MIR144
F3	Coagulation Factor III, Tissue Factor	Protein Coding	45	GC01M094530	15.71	https://www.genecards.org/cgi-bin/carddisp.pl?gene=F3
MIR222	MicroRNA 222	RNA Gene	21	GC0XM045747	15.71	https://www.genecards.org/cgi-bin/carddisp.pl?gene=MIR222
RAC1	Rac Family Small GTPase 1	Protein Coding	49	GC07P006380	15.69	https://www.genecards.org/cgi-bin/carddisp.pl?gene=RAC1
GARS1	Glycyl-TRNA Synthetase 1	Protein Coding	36	GC07P030595	15.64	https://www.genecards.org/cgi-bin/carddisp.pl?gene=GARS1
COX5A	Cytochrome C Oxidase Subunit 5A	Protein Coding	43	GC15M074919	15.51	https://www.genecards.org/cgi-bin/carddisp.pl?gene=COX5A
MIR221	MicroRNA 221	RNA Gene	20	GC0XM045746	15.44	https://www.genecards.org/cgi-bin/carddisp.pl?gene=MIR221
APOA2	Apolipoprotein A2	Protein Coding	44	GC01M161222	15.41	https://www.genecards.org/cgi-bin/carddisp.pl?gene=APOA2
NDUFA2	NADH:Ubiquinone Oxidoreductase Subunit A2	Protein Coding	42	GC05M140640	15.31	https://www.genecards.org/cgi-bin/carddisp.pl?gene=NDUFA2
MT-ND6	Mitochondrially Encoded NADH:Ubiquinone Oxidore	Protein Coding	32	GCMTM014151	15.22	https://www.genecards.org/cgi-bin/carddisp.pl?gene=MT-ND6
CDH2	Cadherin 2	Protein Coding	50	GC18M027950	15.2	https://www.genecards.org/cgi-bin/carddisp.pl?gene=CDH2
HTR2A	5-Hydroxytryptamine Receptor 2A	Protein Coding	47	GC13M046831	15.2	https://www.genecards.org/cgi-bin/carddisp.pl?gene=HTR2A
PLIN1	Perilipin 1	Protein Coding	44	GC15M089664	15.13	https://www.genecards.org/cgi-bin/carddisp.pl?gene=PLIN1
CRAT	Carnitine O-Acetyltransferase	Protein Coding	44	GC09M129094	15.08	https://www.genecards.org/cgi-bin/carddisp.pl?gene=CRAT
MT-CO3	Mitochondrially Encoded Cytochrome C Oxidase III	Protein Coding	30	GCMTP009209	15.08	https://www.genecards.org/cgi-bin/carddisp.pl?gene=MT-CO3
PIK3CD	Phosphatidylinositol-4,5-Bisphosphate 3-Kinase Cataly	Protein Coding	53	GC01P009629	15.07	https://www.genecards.org/cgi-bin/carddisp.pl?gene=PIK3CD
TNFAIP3	TNF Alpha Induced Protein 3	Protein Coding	48	GC06P137866	15.05	https://www.genecards.org/cgi-bin/carddisp.pl?gene=TNFAIP3
EIF2S1	Eukaryotic Translation Initiation Factor 2 Subunit Alpl	Protein Coding	44	GC14P067359	14.99	https://www.genecards.org/cgi-bin/carddisp.pl?gene=EIF2S1
NDUFS8	NADH:Ubiquinone Oxidoreductase Core Subunit S8	Protein Coding	45	GC11P068030	14.98	https://www.genecards.org/cgi-bin/carddisp.pl?gene=NDUFS8
MIR214	MicroRNA 214	RNA Gene	20	GC01M172234	14.95	https://www.genecards.org/cgi-bin/carddisp.pl?gene=MIR214
CPOX	Coproporphyrinogen Oxidase	Protein Coding	43	GC03M098576	14.82	https://www.genecards.org/cgi-bin/carddisp.pl?gene=CPOX
MIR27B	MicroRNA 27b	RNA Gene	21	GC09P095097	14.78	https://www.genecards.org/cgi-bin/carddisp.pl?gene=MIR27B
CDC42	Cell Division Cycle 42	Protein Coding	51	GC01P022057	14.77	https://www.genecards.org/cgi-bin/carddisp.pl?gene=CDC42
SLC40A1	Solute Carrier Family 40 Member 1	Protein Coding	46	GC02M189560	14.76	https://www.genecards.org/cgi-bin/carddisp.pl?gene=SLC40A1
GSTM1	Glutathione S-Transferase Mu 1	Protein Coding	41	GC01P109687	14.72	https://www.genecards.org/cgi-bin/carddisp.pl?gene=GSTM1
CYP1A2	Cytochrome P450 Family 1 Subfamily A Member 2	Protein Coding	45	GC15P074748	14.63	https://www.genecards.org/cgi-bin/carddisp.pl?gene=CYP1A2
GSTP1	Glutathione S-Transferase Pi 1	Protein Coding	50	GC11P067583	14.62	https://www.genecards.org/cgi-bin/carddisp.pl?gene=GSTP1
HIF1A	Hypoxia Inducible Factor 1 Subunit Alpha	Protein Coding	47	GC14P061695	14.6	https://www.genecards.org/cgi-bin/carddisp.pl?gene=HIF1A
SPINK1	Serine Peptidase Inhibitor Kazal Type 1	Protein Coding	43	GC05M147825	14.55	https://www.genecards.org/cgi-bin/carddisp.pl?gene=SPINK1
CCL5	C-C Motif Chemokine Ligand 5	Protein Coding	43	GC17M035871	14.52	https://www.genecards.org/cgi-bin/carddisp.pl?gene=CCL5
SUMF1	Sulfatase Modifying Factor 1	Protein Coding	43	GC03M003700	14.48	https://www.genecards.org/cgi-bin/carddisp.pl?gene=SUMF1
NDUFA1	NADH:Ubiquinone Oxidoreductase Subunit A1	Protein Coding	44	GC0XP119871	14.44	https://www.genecards.org/cgi-bin/carddisp.pl?gene=NDUFA1
AGTR1	Angiotensin II Receptor Type 1	Protein Coding	51	GC03P148697	14.42	https://www.genecards.org/cgi-bin/carddisp.pl?gene=AGTR1
CHI3L1	Chitinase 3 Like 1	Protein Coding	43	GC01M203148	14.39	https://www.genecards.org/cgi-bin/carddisp.pl?gene=CHI3L1
CYBB	Cytochrome B-245 Beta Chain	Protein Coding	47	GC0XP037780	14.36	https://www.genecards.org/cgi-bin/carddisp.pl?gene=CYBB
NDUFB11	NADH:Ubiquinone Oxidoreductase Subunit B11	Protein Coding	39	GC0XM047142	14.35	https://www.genecards.org/cgi-bin/carddisp.pl?gene=NDUFB11

SMAD7	SMAD Family Member 7	Protein Coding	43	GC18M048919	14.34	https://www.genecards.org/cgi-bin/carddisp.pl?gene=SMAD7
NDUFS6	NADH:Ubiquinone Oxidoreductase Subunit S6	Protein Coding	43	GC05P001801	14.3	https://www.genecards.org/cgi-bin/carddisp.pl?gene=NDUFS6
COX6B1	Cytochrome C Oxidase Subunit 6B1	Protein Coding	43	GC19P038224	14.25	https://www.genecards.org/cgi-bin/carddisp.pl?gene=COX6B1
BGLAP	Bone Gamma-Carboxyglutamate Protein	Protein Coding	40	GC01P156242	14.23	https://www.genecards.org/cgi-bin/carddisp.pl?gene=BGLAP
CHIT1	Chitinase 1	Protein Coding	43	GC01M203181	14.22	https://www.genecards.org/cgi-bin/carddisp.pl?gene=CHIT1
NFE2L2	Nuclear Factor, Erythroid 2 Like 2	Protein Coding	48	GC02M177227	14.2	https://www.genecards.org/cgi-bin/carddisp.pl?gene=NFE2L2
MIR182	MicroRNA 182	RNA Gene	20	GC07M129770	14.19	https://www.genecards.org/cgi-bin/carddisp.pl?gene=MIR182
DGAT1	Diacylglycerol O-Acyltransferase 1	Protein Coding	47	GC08M144316	14.17	https://www.genecards.org/cgi-bin/carddisp.pl?gene=DGAT1
KRT7	Keratin 7	Protein Coding	41	GC12P052232	14.1	https://www.genecards.org/cgi-bin/carddisp.pl?gene=KRT7
ELOVL6	ELOVL Fatty Acid Elongase 6	Protein Coding	40	GC04M110045	14.06	https://www.genecards.org/cgi-bin/carddisp.pl?gene=ELOVL6
SERPINA6	Serpin Family A Member 6	Protein Coding	43	GC14M094341	14.06	https://www.genecards.org/cgi-bin/carddisp.pl?gene=SERPINA6
TFR2	Transferrin Receptor 2	Protein Coding	44	GC07M100620	14.02	https://www.genecards.org/cgi-bin/carddisp.pl?gene=TFR2
CRH	Corticotropin Releasing Hormone	Protein Coding	44	GC08M066176	14	https://www.genecards.org/cgi-bin/carddisp.pl?gene=CRH
RXRA	Retinoid X Receptor Alpha	Protein Coding	49	GC09P134317	13.99	https://www.genecards.org/cgi-bin/carddisp.pl?gene=RXRA
BMPR1A	Bone Morphogenetic Protein Receptor Type 1A	Protein Coding	51	GC10P086756	13.97	https://www.genecards.org/cgi-bin/carddisp.pl?gene=BMPR1A
ERBB4	Erb-B2 Receptor Tyrosine Kinase 4	Protein Coding	55	GC02M211375	13.93	https://www.genecards.org/cgi-bin/carddisp.pl?gene=ERBB4
ALPP	Alkaline Phosphatase, Placental	Protein Coding	46	GC02P232378	13.93	https://www.genecards.org/cgi-bin/carddisp.pl?gene=ALPP
TNFRSF11B	TNF Receptor Superfamily Member 11b	Protein Coding	47	GC08M118923	13.93	https://www.genecards.org/cgi-bin/carddisp.pl?gene=TNFRSF11B
CYP17A1	Cytochrome P450 Family 17 Subfamily A Member 1	Protein Coding	48	GC10M102830	13.87	https://www.genecards.org/cgi-bin/carddisp.pl?gene=CYP17A1
PLA2G4A	Phospholipase A2 Group IVA	Protein Coding	49	GC01P186798	13.82	https://www.genecards.org/cgi-bin/carddisp.pl?gene=PLA2G4A
NDUFB3	NADH:Ubiquinone Oxidoreductase Subunit B3	Protein Coding	42	GC02P201071	13.82	https://www.genecards.org/cgi-bin/carddisp.pl?gene=NDUFB3
VCAM1	Vascular Cell Adhesion Molecule 1	Protein Coding	45	GC01P100719	13.8	https://www.genecards.org/cgi-bin/carddisp.pl?gene=VCAM1
LRP6	LDL Receptor Related Protein 6	Protein Coding	48	GC12M013893	13.8	https://www.genecards.org/cgi-bin/carddisp.pl?gene=LRP6
SERPINF2	Serpin Family F Member 2	Protein Coding	44	GC17P001742	13.73	https://www.genecards.org/cgi-bin/carddisp.pl?gene=SERPINF2
ETS1	ETS Proto-Oncogene 1, Transcription Factor	Protein Coding	49	GC11M128458	13.72	https://www.genecards.org/cgi-bin/carddisp.pl?gene=ETS1
HSD11B1	Hydroxysteroid 11-Beta Dehydrogenase 1	Protein Coding	49	GC01P209686	13.7	https://www.genecards.org/cgi-bin/carddisp.pl?gene=HSD11B1
FGFR4	Fibroblast Growth Factor Receptor 4	Protein Coding	51	GC05P177086	13.69	https://www.genecards.org/cgi-bin/carddisp.pl?gene=FGFR4
LCN2	Lipocalin 2	Protein Coding	43	GC09P128149	13.67	https://www.genecards.org/cgi-bin/carddisp.pl?gene=LCN2
ADH1C	Alcohol Dehydrogenase 1C (Class I), Gamma Polypep	Protein Coding	42	GC04M099336	13.67	https://www.genecards.org/cgi-bin/carddisp.pl?gene=ADH1C
ALPL	Alkaline Phosphatase, Biomineralization Associated	Protein Coding	50	GC01P021508	13.66	https://www.genecards.org/cgi-bin/carddisp.pl?gene=ALPL
SHBG	Sex Hormone Binding Globulin	Protein Coding	40	GC17P007613	13.61	https://www.genecards.org/cgi-bin/carddisp.pl?gene=SHBG
MYF5	Myogenic Factor 5	Protein Coding	40	GC12P080716	13.61	https://www.genecards.org/cgi-bin/carddisp.pl?gene=MYF5
DSP	Desmoplakin	Protein Coding	49	GC06P007541	13.6	https://www.genecards.org/cgi-bin/carddisp.pl?gene=DSP
CA2	Carbonic Anhydrase 2	Protein Coding	51	GC08P085463	13.6	https://www.genecards.org/cgi-bin/carddisp.pl?gene=CA2
FABP3	Fatty Acid Binding Protein 3	Protein Coding	44	GC01M031365	13.59	https://www.genecards.org/cgi-bin/carddisp.pl?gene=FABP3
KRT19	Keratin 19	Protein Coding	45	GC17M041523	13.59	https://www.genecards.org/cgi-bin/carddisp.pl?gene=KRT19
CD55	CD55 Molecule (Cromer Blood Group)	Protein Coding	47	GC01P207321	13.52	https://www.genecards.org/cgi-bin/carddisp.pl?gene=CD55
IFNA2	Interferon Alpha 2	Protein Coding	41	GC09M021384	13.51	https://www.genecards.org/cgi-bin/carddisp.pl?gene=IFNA2
MIR15B	MicroRNA 15b	RNA Gene	18	GC03P160404	13.51	https://www.genecards.org/cgi-bin/carddisp.pl?gene=MIR15B

SELE	Selectin E	Protein Coding	44	GC01M169722	13.41	https://www.genecards.org/cgi-bin/carddisp.pl?gene=SELE
CNR1	Cannabinoid Receptor 1	Protein Coding	46	GC06M088139	13.4	https://www.genecards.org/cgi-bin/carddisp.pl?gene=CNR1
MAPK10	Mitogen-Activated Protein Kinase 10	Protein Coding	51	GC04M085990	13.39	https://www.genecards.org/cgi-bin/carddisp.pl?gene=MAPK10
HP	Haptoglobin	Protein Coding	44	GC16P072089	13.38	https://www.genecards.org/cgi-bin/carddisp.pl?gene=HP
ERCC2	ERCC Excision Repair 2, TFIIH Core Complex Helica	Protein Coding	47	GC19M045349	13.35	https://www.genecards.org/cgi-bin/carddisp.pl?gene=ERCC2
HUWE1	HECT, UBA And WWE Domain Containing E3 Ubiqu	Protein Coding	44	GC0XM053532	13.29	https://www.genecards.org/cgi-bin/carddisp.pl?gene=HUWE1
MAPK14	Mitogen-Activated Protein Kinase 14	Protein Coding	51	GC06P047451	13.27	https://www.genecards.org/cgi-bin/carddisp.pl?gene=MAPK14
AGT	Angiotensinogen	Protein Coding	49	GC01M230702	13.23	https://www.genecards.org/cgi-bin/carddisp.pl?gene=AGT
NDUFA10	NADH:Ubiquinone Oxidoreductase Subunit A10	Protein Coding	44	GC02M239893	13.16	https://www.genecards.org/cgi-bin/carddisp.pl?gene=NDUFA10
UQCRC2	Ubiquinol-Cytochrome C Reductase Core Protein 2	Protein Coding	46	GC16P021963	13.12	https://www.genecards.org/cgi-bin/carddisp.pl?gene=UQCRC2
NDUFA12	NADH:Ubiquinone Oxidoreductase Subunit A12	Protein Coding	44	GC12M094898	13.11	https://www.genecards.org/cgi-bin/carddisp.pl?gene=NDUFA12
CD44	CD44 Molecule (Indian Blood Group)	Protein Coding	47	GC11P035139	13.1	https://www.genecards.org/cgi-bin/carddisp.pl?gene=CD44
IRF1	Interferon Regulatory Factor 1	Protein Coding	47	GC05M132481	13.06	https://www.genecards.org/cgi-bin/carddisp.pl?gene=IRF1
WNT3A	Wnt Family Member 3A	Protein Coding	47	GC01P228127	13.04	https://www.genecards.org/cgi-bin/carddisp.pl?gene=WNT3A
GLUD1	Glutamate Dehydrogenase 1	Protein Coding	50	GC10M087050	13.02	https://www.genecards.org/cgi-bin/carddisp.pl?gene=GLUD1
ALDH2	Aldehyde Dehydrogenase 2 Family Member	Protein Coding	50	GC12P111766	13.01	https://www.genecards.org/cgi-bin/carddisp.pl?gene=ALDH2
SELP	Selectin P	Protein Coding	45	GC01M169558	12.95	https://www.genecards.org/cgi-bin/carddisp.pl?gene=SELP
MIR154	MicroRNA 154	RNA Gene	18	GC14P104778	12.94	https://www.genecards.org/cgi-bin/carddisp.pl?gene=MIR154
HJV	Hemojuvelin BMP Co-Receptor	Protein Coding	35	GC01M146018	12.91	https://www.genecards.org/cgi-bin/carddisp.pl?gene=HJV
SMAD3	SMAD Family Member 3	Protein Coding	49	GC15P067063	12.88	https://www.genecards.org/cgi-bin/carddisp.pl?gene=SMAD3
PEMT	Phosphatidylethanolamine N-Methyltransferase	Protein Coding	41	GC17M017506	12.86	https://www.genecards.org/cgi-bin/carddisp.pl?gene=PEMT
IL6R	Interleukin 6 Receptor	Protein Coding	48	GC01P154405	12.85	https://www.genecards.org/cgi-bin/carddisp.pl?gene=IL6R
TNFSF10	TNF Superfamily Member 10	Protein Coding	46	GC03M172505	12.81	https://www.genecards.org/cgi-bin/carddisp.pl?gene=TNFSF10
ADH1B	Alcohol Dehydrogenase 1B (Class I), Beta Polypeptide	Protein Coding	43	GC04M099304	12.77	https://www.genecards.org/cgi-bin/carddisp.pl?gene=ADH1B
MT-ND3	Mitochondrially Encoded NADH:Ubiquinone Oxidore	Protein Coding	31	GCMTP010061	12.75	https://www.genecards.org/cgi-bin/carddisp.pl?gene=MT-ND3
CYP1B1	Cytochrome P450 Family 1 Subfamily B Member 1	Protein Coding	48	GC02M038034	12.7	https://www.genecards.org/cgi-bin/carddisp.pl?gene=CYP1B1
NPPB	Natriuretic Peptide B	Protein Coding	44	GC01M011858	12.66	https://www.genecards.org/cgi-bin/carddisp.pl?gene=NPPB
MIR23B	MicroRNA 23b	RNA Gene	20	GC09P095085	12.56	https://www.genecards.org/cgi-bin/carddisp.pl?gene=MIR23B
AIFM1	Apoptosis Inducing Factor Mitochondria Associated 1	Protein Coding	49	GC0XM130129	12.51	https://www.genecards.org/cgi-bin/carddisp.pl?gene=AIFM1
MYH9	Myosin Heavy Chain 9	Protein Coding	49	GC22M036281	12.51	https://www.genecards.org/cgi-bin/carddisp.pl?gene=MYH9
SLC27A1	Solute Carrier Family 27 Member 1	Protein Coding	41	GC19P023305	12.5	https://www.genecards.org/cgi-bin/carddisp.pl?gene=SLC27A1
CYC1	Cytochrome C1	Protein Coding	45	GC08P144095	12.48	https://www.genecards.org/cgi-bin/carddisp.pl?gene=CYC1
LNCARSR	LncRNA Regulator Of Akt Signaling Associated With	RNA Gene	10	GC09M079507	12.45	https://www.genecards.org/cgi-bin/carddisp.pl?gene=LNCARSR
FADS2	Fatty Acid Desaturase 2	Protein Coding	44	GC11P061792	12.44	https://www.genecards.org/cgi-bin/carddisp.pl?gene=FADS2
DRD3	Dopamine Receptor D3	Protein Coding	44	GC03M114128	12.44	https://www.genecards.org/cgi-bin/carddisp.pl?gene=DRD3
SCARB1	Scavenger Receptor Class B Member 1	Protein Coding	45	GC12M124776	12.43	https://www.genecards.org/cgi-bin/carddisp.pl?gene=SCARB1
CEL	Carboxyl Ester Lipase	Protein Coding	46	GC09P133061	12.37	https://www.genecards.org/cgi-bin/carddisp.pl?gene=CEL
UCP1	Uncoupling Protein 1	Protein Coding	44	GC04M140559	12.34	https://www.genecards.org/cgi-bin/carddisp.pl?gene=UCP1
MMP13	Matrix Metallopeptidase 13	Protein Coding	50	GC11M102942	12.29	https://www.genecards.org/cgi-bin/carddisp.pl?gene=MMP13

ADA2	Adenosine Deaminase 2	Protein Coding	34	GC22M017179	12.29	https://www.genecards.org/cgi-bin/carddisp.pl?gene=ADA2
NR1I2	Nuclear Receptor Subfamily 1 Group I Member 2	Protein Coding	45	GC03P119780	12.16	https://www.genecards.org/cgi-bin/carddisp.pl?gene=NR1I2
LIVAR	Liver Cell Viability Associated LncRNA	RNA Gene	10	GC18M070336	12.11	https://www.genecards.org/cgi-bin/carddisp.pl?gene=LIVAR
LRPPRC	Leucine Rich Pentatricopeptide Repeat Containing	Protein Coding	42	GC02M043850	12.1	https://www.genecards.org/cgi-bin/carddisp.pl?gene=LRPPRC
HNRNPA1	Heterogeneous Nuclear Ribonucleoprotein A1	Protein Coding	45	GC12P054280	12.1	https://www.genecards.org/cgi-bin/carddisp.pl?gene=HNRNPA1
PRKAA2	Protein Kinase AMP-Activated Catalytic Subunit Alph	Protein Coding	50	GC01P056645	12.06	https://www.genecards.org/cgi-bin/carddisp.pl?gene=PRKAA2
CPS1	Carbamoyl-Phosphate Synthase 1	Protein Coding	45	GC02P210477	12.05	https://www.genecards.org/cgi-bin/carddisp.pl?gene=CPS1
TIMP2	TIMP Metallopeptidase Inhibitor 2	Protein Coding	44	GC17M078852	12.04	https://www.genecards.org/cgi-bin/carddisp.pl?gene=TIMP2
PRSS1	Serine Protease 1	Protein Coding	45	GC07P144938	12	https://www.genecards.org/cgi-bin/carddisp.pl?gene=PRSS1
CYBA	Cytochrome B-245 Alpha Chain	Protein Coding	47	GC16M088643	11.98	https://www.genecards.org/cgi-bin/carddisp.pl?gene=CYBA
IGFBP3	Insulin Like Growth Factor Binding Protein 3	Protein Coding	45	GC07M045912	11.96	https://www.genecards.org/cgi-bin/carddisp.pl?gene=IGFBP3
MIR95	MicroRNA 95	RNA Gene	16	GC04M008007	11.95	https://www.genecards.org/cgi-bin/carddisp.pl?gene=MIR95
CYP2A6	Cytochrome P450 Family 2 Subfamily A Member 6	Protein Coding	47	GC19M040843	11.92	https://www.genecards.org/cgi-bin/carddisp.pl?gene=CYP2A6
ADRB3	Adrenoceptor Beta 3	Protein Coding	45	GC08M037962	11.92	https://www.genecards.org/cgi-bin/carddisp.pl?gene=ADRB3
MIR187	MicroRNA 187	RNA Gene	17	GC18M035904	11.82	https://www.genecards.org/cgi-bin/carddisp.pl?gene=MIR187
NDUFA11	NADH:Ubiquinone Oxidoreductase Subunit A11	Protein Coding	37	GC19M005891	11.75	https://www.genecards.org/cgi-bin/carddisp.pl?gene=NDUFA11
FLI1	Fli-1 Proto-Oncogene, ETS Transcription Factor	Protein Coding	49	GC11P128686	11.73	https://www.genecards.org/cgi-bin/carddisp.pl?gene=FLI1
SHH	Sonic Hedgehog Signaling Molecule	Protein Coding	50	GC07M155799	11.69	https://www.genecards.org/cgi-bin/carddisp.pl?gene=SHH
HSP90AA1	Heat Shock Protein 90 Alpha Family Class A Member	Protein Coding	48	GC14M102080	11.63	https://www.genecards.org/cgi-bin/carddisp.pl?gene=HSP90AA1
PRL	Prolactin	Protein Coding	44	GC06M022230	11.62	https://www.genecards.org/cgi-bin/carddisp.pl?gene=PRL
PARP1	Poly(ADP-Ribose) Polymerase 1	Protein Coding	49	GC01M226360	11.61	https://www.genecards.org/cgi-bin/carddisp.pl?gene=PARP1
UQCRQ	Ubiquinol-Cytochrome C Reductase Complex III Subu	Protein Coding	41	GC05P132866	11.6	https://www.genecards.org/cgi-bin/carddisp.pl?gene=UQCRQ
COX8A	Cytochrome C Oxidase Subunit 8A	Protein Coding	41	GC11P063977	11.57	https://www.genecards.org/cgi-bin/carddisp.pl?gene=COX8A
FGA	Fibrinogen Alpha Chain	Protein Coding	47	GC04M154583	11.44	https://www.genecards.org/cgi-bin/carddisp.pl?gene=FGA
HBA1	Hemoglobin Subunit Alpha 1	Protein Coding	42	GC16P001370	11.37	https://www.genecards.org/cgi-bin/carddisp.pl?gene=HBA1
SLC27A2	Solute Carrier Family 27 Member 2	Protein Coding	45	GC15P050182	11.34	https://www.genecards.org/cgi-bin/carddisp.pl?gene=SLC27A2
IL15	Interleukin 15	Protein Coding	40	GC04P141636	11.28	https://www.genecards.org/cgi-bin/carddisp.pl?gene=IL15
LITAF	Lipopolysaccharide Induced TNF Factor	Protein Coding	44	GC16M011547	11.25	https://www.genecards.org/cgi-bin/carddisp.pl?gene=LITAF
CSF1	Colony Stimulating Factor 1	Protein Coding	43	GC01P109911	11.22	https://www.genecards.org/cgi-bin/carddisp.pl?gene=CSF1
IL1R1	Interleukin 1 Receptor Type 1	Protein Coding	45	GC02P102136	11.19	https://www.genecards.org/cgi-bin/carddisp.pl?gene=IL1R1
GC	GC Vitamin D Binding Protein	Protein Coding	43	GC04M071741	11.17	https://www.genecards.org/cgi-bin/carddisp.pl?gene=GC
STAR	Steroidogenic Acute Regulatory Protein	Protein Coding	46	GC08M038145	11.13	https://www.genecards.org/cgi-bin/carddisp.pl?gene=STAR
NPY	Neuropeptide Y	Protein Coding	45	GC07P024290	11.13	https://www.genecards.org/cgi-bin/carddisp.pl?gene=NPY
CES1	Carboxylesterase 1	Protein Coding	46	GC16M055836	11.11	https://www.genecards.org/cgi-bin/carddisp.pl?gene=CES1
COX4I1	Cytochrome C Oxidase Subunit 4I1	Protein Coding	44	GC16P085798	11.11	https://www.genecards.org/cgi-bin/carddisp.pl?gene=COX4I1
AHR	Aryl Hydrocarbon Receptor	Protein Coding	48	GC07P016916	11.09	https://www.genecards.org/cgi-bin/carddisp.pl?gene=AHR
FABP12	Fatty Acid Binding Protein 12	Protein Coding	31	GC08M081524	11.09	https://www.genecards.org/cgi-bin/carddisp.pl?gene=FABP12
IFNL3	Interferon Lambda 3	Protein Coding	35	GC19M039243	11.07	https://www.genecards.org/cgi-bin/carddisp.pl?gene=IFNL3
CLU	Clusterin	Protein Coding	46	GC08M027596	11.07	https://www.genecards.org/cgi-bin/carddisp.pl?gene=CLU

DDX3X	DEAD-Box Helicase 3 X-Linked	Protein Coding	47	GC0XP041333	11.05	https://www.genecards.org/cgi-bin/carddisp.pl?gene=DDX3X
DKK1	Dickkopf WNT Signaling Pathway Inhibitor 1	Protein Coding	45	GC10P052314	11.05	https://www.genecards.org/cgi-bin/carddisp.pl?gene=DKK1
SLC10A1	Solute Carrier Family 10 Member 1	Protein Coding	43	GC14M069775	11.04	https://www.genecards.org/cgi-bin/carddisp.pl?gene=SLC10A1
CXCR3	C-X-C Motif Chemokine Receptor 3	Protein Coding	44	GC0XM071615	10.96	https://www.genecards.org/cgi-bin/carddisp.pl?gene=CXCR3
CASP7	Caspase 7	Protein Coding	50	GC10P113679	10.84	https://www.genecards.org/cgi-bin/carddisp.pl?gene=CASP7
S100A9	S100 Calcium Binding Protein A9	Protein Coding	43	GC01P153357	10.83	https://www.genecards.org/cgi-bin/carddisp.pl?gene=S100A9
CASP9	Caspase 9	Protein Coding	48	GC01M015491	10.82	https://www.genecards.org/cgi-bin/carddisp.pl?gene=CASP9
MIR29A	MicroRNA 29a	RNA Gene	21	GC07M130876	10.78	https://www.genecards.org/cgi-bin/carddisp.pl?gene=MIR29A
TET2	Tet Methylcytosine Dioxygenase 2	Protein Coding	44	GC04P105145	10.78	https://www.genecards.org/cgi-bin/carddisp.pl?gene=TET2
RARS1	Arginyl-TRNA Synthetase 1	Protein Coding	35	GC05P168487	10.77	https://www.genecards.org/cgi-bin/carddisp.pl?gene=RARS1
BIRC5	Baculoviral IAP Repeat Containing 5	Protein Coding	47	GC17P078214	10.75	https://www.genecards.org/cgi-bin/carddisp.pl?gene=BIRC5
HSPG2	Heparan Sulfate Proteoglycan 2	Protein Coding	45	GC01M021822	10.73	https://www.genecards.org/cgi-bin/carddisp.pl?gene=HSPG2
XRCC4	X-Ray Repair Cross Complementing 4	Protein Coding	43	GC05P083077	10.73	https://www.genecards.org/cgi-bin/carddisp.pl?gene=XRCC4
CLCN2	Chloride Voltage-Gated Channel 2	Protein Coding	45	GC03M184346	10.72	https://www.genecards.org/cgi-bin/carddisp.pl?gene=CLCN2
PPIG	Peptidylprolyl Isomerase G	Protein Coding	41	GC02P169584	10.71	https://www.genecards.org/cgi-bin/carddisp.pl?gene=PPIG
SP1	Sp1 Transcription Factor	Protein Coding	44	GC12P053380	10.7	https://www.genecards.org/cgi-bin/carddisp.pl?gene=SP1
GHR	Growth Hormone Receptor	Protein Coding	47	GC05P042429	10.68	https://www.genecards.org/cgi-bin/carddisp.pl?gene=GHR
DNASE1	Deoxyribonuclease 1	Protein Coding	41	GC16P003611	10.66	https://www.genecards.org/cgi-bin/carddisp.pl?gene=DNASE1
IGFBP1	Insulin Like Growth Factor Binding Protein 1	Protein Coding	43	GC07P046552	10.65	https://www.genecards.org/cgi-bin/carddisp.pl?gene=IGFBP1
GFER	Growth Factor, Augmenter Of Liver Regeneration	Protein Coding	45	GC16P001984	10.65	https://www.genecards.org/cgi-bin/carddisp.pl?gene=GFER
TMPRSS6	Transmembrane Serine Protease 6	Protein Coding	44	GC22M037066	10.64	https://www.genecards.org/cgi-bin/carddisp.pl?gene=TMPRSS6
ADRB2	Adrenoceptor Beta 2	Protein Coding	48	GC05P148825	10.62	https://www.genecards.org/cgi-bin/carddisp.pl?gene=ADRB2
FTL	Ferritin Light Chain	Protein Coding	47	GC19P048965	10.61	https://www.genecards.org/cgi-bin/carddisp.pl?gene=FTL
DGAT2	Diacylglycerol O-Acyltransferase 2	Protein Coding	43	GC11P075759	10.58	https://www.genecards.org/cgi-bin/carddisp.pl?gene=DGAT2
HSPA5	Heat Shock Protein Family A (Hsp70) Member 5	Protein Coding	47	GC09M125234	10.56	https://www.genecards.org/cgi-bin/carddisp.pl?gene=HSPA5
SPARC	Secreted Protein Acidic And Cysteine Rich	Protein Coding	50	GC05M151639	10.54	https://www.genecards.org/cgi-bin/carddisp.pl?gene=SPARC
GAL	Galanin And GMAP Prepropeptide	Protein Coding	44	GC11P068684	10.51	https://www.genecards.org/cgi-bin/carddisp.pl?gene=GAL
NR0B2	Nuclear Receptor Subfamily 0 Group B Member 2	Protein Coding	43	GC01M026922	10.51	https://www.genecards.org/cgi-bin/carddisp.pl?gene=NR0B2
EPRS1	Glutamyl-Prolyl-TRNA Synthetase 1	Protein Coding	36	GC01M219969	10.42	https://www.genecards.org/cgi-bin/carddisp.pl?gene=EPRS1
IL21	Interleukin 21	Protein Coding	43	GC04M122612	10.41	https://www.genecards.org/cgi-bin/carddisp.pl?gene=IL21
KL	Klotho	Protein Coding	44	GC13P033016	10.37	https://www.genecards.org/cgi-bin/carddisp.pl?gene=KL
PTPN1	Protein Tyrosine Phosphatase Non-Receptor Type 1	Protein Coding	51	GC20P050510	10.34	https://www.genecards.org/cgi-bin/carddisp.pl?gene=PTPN1
MIR29B1	MicroRNA 29b-1	RNA Gene	21	GC07M130877	10.31	https://www.genecards.org/cgi-bin/carddisp.pl?gene=MIR29B1
MAOA	Monoamine Oxidase A	Protein Coding	50	GC0XP043654	10.3	https://www.genecards.org/cgi-bin/carddisp.pl?gene=MAOA
FGB	Fibrinogen Beta Chain	Protein Coding	45	GC04P154564	10.3	https://www.genecards.org/cgi-bin/carddisp.pl?gene=FGB
CCK	Cholecystokinin	Protein Coding	41	GC03M042274	10.19	https://www.genecards.org/cgi-bin/carddisp.pl?gene=CCK
GDF15	Growth Differentiation Factor 15	Protein Coding	41	GC19P023329	10.18	https://www.genecards.org/cgi-bin/carddisp.pl?gene=GDF15
DRD1	Dopamine Receptor D1	Protein Coding	44	GC05M175440	10.17	https://www.genecards.org/cgi-bin/carddisp.pl?gene=DRD1
DNMT3A	DNA Methyltransferase 3 Alpha	Protein Coding	51	GC02M025228	10.13	https://www.genecards.org/cgi-bin/carddisp.pl?gene=DNMT3A

MIR149	MicroRNA 149	RNA Gene	21	GC02P240456	10.12	https://www.genecards.org/cgi-bin/carddisp.pl?gene=MIR149
EIF2AK4	Eukaryotic Translation Initiation Factor 2 Alpha Kinas	Protein Coding	44	GC15P039934	10.09	https://www.genecards.org/cgi-bin/carddisp.pl?gene=EIF2AK4
GPX1	Glutathione Peroxidase 1	Protein Coding	48	GC03M049368	10.07	https://www.genecards.org/cgi-bin/carddisp.pl?gene=GPX1
PIK3CB	Phosphatidylinositol-4,5-Bisphosphate 3-Kinase Catal	Protein Coding	47	GC03M138652	10.07	https://www.genecards.org/cgi-bin/carddisp.pl?gene=PIK3CB
MIR33B	MicroRNA 33b	RNA Gene	18	GC17M017813	9.99	https://www.genecards.org/cgi-bin/carddisp.pl?gene=MIR33B
ITCH	Itchy E3 Ubiquitin Protein Ligase	Protein Coding	45	GC20P034363	9.92	https://www.genecards.org/cgi-bin/carddisp.pl?gene=ITCH
MST1	Macrophage Stimulating 1	Protein Coding	44	GC03M049683	9.91	https://www.genecards.org/cgi-bin/carddisp.pl?gene=MST1
NQO1	NAD(P)H Quinone Dehydrogenase 1	Protein Coding	49	GC16M069706	9.89	https://www.genecards.org/cgi-bin/carddisp.pl?gene=NQO1
HMGB1	High Mobility Group Box 1	Protein Coding	44	GC13M030456	9.86	https://www.genecards.org/cgi-bin/carddisp.pl?gene=HMGB1
COL3A1	Collagen Type III Alpha 1 Chain	Protein Coding	47	GC02P188974	9.83	https://www.genecards.org/cgi-bin/carddisp.pl?gene=COL3A1
LTBP3	Latent Transforming Growth Factor Beta Binding Prot	Protein Coding	41	GC11M065538	9.8	https://www.genecards.org/cgi-bin/carddisp.pl?gene=LTBP3
PPARD	Peroxisome Proliferator Activated Receptor Delta	Protein Coding	47	GC06P047433	9.77	https://www.genecards.org/cgi-bin/carddisp.pl?gene=PPARD
TPM2	Tropomyosin 2	Protein Coding	44	GC09M035672	9.72	https://www.genecards.org/cgi-bin/carddisp.pl?gene=TPM2
PIK3R3	Phosphoinositide-3-Kinase Regulatory Subunit 3	Protein Coding	43	GC01M046041	9.72	https://www.genecards.org/cgi-bin/carddisp.pl?gene=PIK3R3
THBS1	Thrombospondin 1	Protein Coding	44	GC15P039581	9.68	https://www.genecards.org/cgi-bin/carddisp.pl?gene=THBS1
AKR1A1	Aldo-Keto Reductase Family 1 Member A1	Protein Coding	44	GC01P045550	9.66	https://www.genecards.org/cgi-bin/carddisp.pl?gene=AKR1A1
MIR9-1	MicroRNA 9-1	RNA Gene	20	GC01M156420	9.65	https://www.genecards.org/cgi-bin/carddisp.pl?gene=MIR9-1
APPL1	Adaptor Protein, Phosphotyrosine Interacting With PH	Protein Coding	45	GC03P057227	9.65	https://www.genecards.org/cgi-bin/carddisp.pl?gene=APPL1
RARRES2	Retinoic Acid Receptor Responder 2	Protein Coding	39	GC07M150333	9.6	https://www.genecards.org/cgi-bin/carddisp.pl?gene=RARRES2
SDCCAG8	SHH Signaling And Ciliogenesis Regulator SDCCAG	Protein Coding	41	GC01P243255	9.6	https://www.genecards.org/cgi-bin/carddisp.pl?gene=SDCCAG8
AGER	Advanced Glycosylation End-Product Specific Recept	Protein Coding	44	GC06M032180	9.57	https://www.genecards.org/cgi-bin/carddisp.pl?gene=AGER
TMPO	Thymopoietin	Protein Coding	45	GC12P098515	9.56	https://www.genecards.org/cgi-bin/carddisp.pl?gene=TMPO
EPAS1	Endothelial PAS Domain Protein 1	Protein Coding	48	GC02P046293	9.56	https://www.genecards.org/cgi-bin/carddisp.pl?gene=EPAS1
IRF8	Interferon Regulatory Factor 8	Protein Coding	45	GC16P085898	9.55	https://www.genecards.org/cgi-bin/carddisp.pl?gene=IRF8
NR5A2	Nuclear Receptor Subfamily 5 Group A Member 2	Protein Coding	45	GC01P199996	9.53	https://www.genecards.org/cgi-bin/carddisp.pl?gene=NR5A2
SLC25A22	Solute Carrier Family 25 Member 22	Protein Coding	41	GC11M000791	9.51	https://www.genecards.org/cgi-bin/carddisp.pl?gene=SLC25A22
CD163	CD163 Molecule	Protein Coding	42	GC12M007471	9.5	https://www.genecards.org/cgi-bin/carddisp.pl?gene=CD163
SHC1	SHC Adaptor Protein 1	Protein Coding	45	GC01M154962	9.5	https://www.genecards.org/cgi-bin/carddisp.pl?gene=SHC1
UQCRB	Ubiquinol-Cytochrome C Reductase Binding Protein	Protein Coding	43	GC08M096225	9.46	https://www.genecards.org/cgi-bin/carddisp.pl?gene=UQCRB
EIF4G1	Eukaryotic Translation Initiation Factor 4 Gamma 1	Protein Coding	45	GC03P184314	9.42	https://www.genecards.org/cgi-bin/carddisp.pl?gene=EIF4G1
SI	Sucrase-Isomaltase	Protein Coding	43	GC03M164978	9.38	https://www.genecards.org/cgi-bin/carddisp.pl?gene=SI
RPS6KB1	Ribosomal Protein S6 Kinase B1	Protein Coding	49	GC17P059893	9.35	https://www.genecards.org/cgi-bin/carddisp.pl?gene=RPS6KB1
PLAUR	Plasminogen Activator, Urokinase Receptor	Protein Coding	44	GC19M043646	9.34	https://www.genecards.org/cgi-bin/carddisp.pl?gene=PLAUR
PALLD	Palladin, Cytoskeletal Associated Protein	Protein Coding	42	GC04P168497	9.33	https://www.genecards.org/cgi-bin/carddisp.pl?gene=PALLD
TPM1	Tropomyosin 1	Protein Coding	48	GC15P073930	9.32	https://www.genecards.org/cgi-bin/carddisp.pl?gene=TPM1
COX6A1	Cytochrome C Oxidase Subunit 6A1	Protein Coding	44	GC12P120439	9.31	https://www.genecards.org/cgi-bin/carddisp.pl?gene=COX6A1
USF1	Upstream Transcription Factor 1	Protein Coding	43	GC01M161039	9.3	https://www.genecards.org/cgi-bin/carddisp.pl?gene=USF1
PEPD	Peptidase D	Protein Coding	45	GC19M033386	9.26	https://www.genecards.org/cgi-bin/carddisp.pl?gene=PEPD
CD59	CD59 Molecule (CD59 Blood Group)	Protein Coding	46	GC11M033704	9.25	https://www.genecards.org/cgi-bin/carddisp.pl?gene=CD59

CLRN1	Clarin 1	Protein Coding	35	GC03M150926	9.15	https://www.genecards.org/cgi-bin/carddisp.pl?gene=CLRN1
ABCG1	ATP Binding Cassette Subfamily G Member 1	Protein Coding	43	GC21P042199	9.08	https://www.genecards.org/cgi-bin/carddisp.pl?gene=ABCG1
NAT2	N-Acetyltransferase 2	Protein Coding	43	GC08P018391	9.07	https://www.genecards.org/cgi-bin/carddisp.pl?gene=NAT2
P4HB	Prolyl 4-Hydroxylase Subunit Beta	Protein Coding	49	GC17M081843	9.07	https://www.genecards.org/cgi-bin/carddisp.pl?gene=P4HB
GOLM1	Golgi Membrane Protein 1	Protein Coding	39	GC09M086026	9.06	https://www.genecards.org/cgi-bin/carddisp.pl?gene=GOLM1
MAPK9	Mitogen-Activated Protein Kinase 9	Protein Coding	48	GC05M180234	9.02	https://www.genecards.org/cgi-bin/carddisp.pl?gene=MAPK9
LEPQTL1	Leptin, Serum Levels Of	Genetic Locus	3	GC02U903086	9.01	https://www.genecards.org/cgi-bin/carddisp.pl?gene=LEPQTL1
CFLAR	CASP8 And FADD Like Apoptosis Regulator	Protein Coding	46	GC02P201117	9	https://www.genecards.org/cgi-bin/carddisp.pl?gene=CFLAR
NPC1L1	NPC1 Like Intracellular Cholesterol Transporter 1	Protein Coding	44	GC07M044512	8.99	https://www.genecards.org/cgi-bin/carddisp.pl?gene=NPC1L1
LGALS3	Galectin 3	Protein Coding	44	GC14P055124	8.97	https://www.genecards.org/cgi-bin/carddisp.pl?gene=LGALS3
CD14	CD14 Molecule	Protein Coding	44	GC05M140631	8.97	https://www.genecards.org/cgi-bin/carddisp.pl?gene=CD14
IL17RA	Interleukin 17 Receptor A	Protein Coding	44	GC22P017086	8.89	https://www.genecards.org/cgi-bin/carddisp.pl?gene=IL17RA
NDUFAB1	NADH:Ubiquinone Oxidoreductase Subunit AB1	Protein Coding	40	GC16M023582	8.85	https://www.genecards.org/cgi-bin/carddisp.pl?gene=NDUFAB1
AREG	Amphiregulin	Protein Coding	43	GC04P074445	8.78	https://www.genecards.org/cgi-bin/carddisp.pl?gene=AREG
FDFT1	Farnesyl-Diphosphate Farnesyltransferase 1	Protein Coding	44	GC08P011795	8.71	https://www.genecards.org/cgi-bin/carddisp.pl?gene=FDFT1
DPM1	Dolichyl-Phosphate Mannosyltransferase Subunit 1, Ca	Protein Coding	44	GC20M050934	8.71	https://www.genecards.org/cgi-bin/carddisp.pl?gene=DPM1
NEAT1	Nuclear Paraspeckle Assembly Transcript 1	RNA Gene	23	GC11P065794	8.7	https://www.genecards.org/cgi-bin/carddisp.pl?gene=NEAT1
TPM3	Tropomyosin 3	Protein Coding	47	GC01M154127	8.69	https://www.genecards.org/cgi-bin/carddisp.pl?gene=TPM3
GABRG2	Gamma-Aminobutyric Acid Type A Receptor Subunit	Protein Coding	46	GC05P162000	8.67	https://www.genecards.org/cgi-bin/carddisp.pl?gene=GABRG2
DICER1	Dicer 1, Ribonuclease III	Protein Coding	47	GC14M095086	8.66	https://www.genecards.org/cgi-bin/carddisp.pl?gene=DICER1
PLIN2	Perilipin 2	Protein Coding	43	GC09M019115	8.64	https://www.genecards.org/cgi-bin/carddisp.pl?gene=PLIN2
NLRC4	NLR Family CARD Domain Containing 4	Protein Coding	44	GC02M032224	8.63	https://www.genecards.org/cgi-bin/carddisp.pl?gene=NLRC4
MERTK	MER Proto-Oncogene, Tyrosine Kinase	Protein Coding	50	GC02P111898	8.62	https://www.genecards.org/cgi-bin/carddisp.pl?gene=MERTK
KEAP1	Kelch Like ECH Associated Protein 1	Protein Coding	48	GC19M010486	8.58	https://www.genecards.org/cgi-bin/carddisp.pl?gene=KEAP1
ATP5F1E	ATP Synthase F1 Subunit Epsilon	Protein Coding	32	GC20M059026	8.51	https://www.genecards.org/cgi-bin/carddisp.pl?gene=ATP5F1E
FOXF1	Forkhead Box F1	Protein Coding	41	GC16P086510	8.49	https://www.genecards.org/cgi-bin/carddisp.pl?gene=FOXF1
RPS27A	Ribosomal Protein S27a	Protein Coding	43	GC02P055231	8.47	https://www.genecards.org/cgi-bin/carddisp.pl?gene=RPS27A
DHCR24	24-Dehydrocholesterol Reductase	Protein Coding	45	GC01M054849	8.47	https://www.genecards.org/cgi-bin/carddisp.pl?gene=DHCR24
BIRC3	Baculoviral IAP Repeat Containing 3	Protein Coding	46	GC11P102317	8.45	https://www.genecards.org/cgi-bin/carddisp.pl?gene=BIRC3
ATF6	Activating Transcription Factor 6	Protein Coding	47	GC01P161766	8.42	https://www.genecards.org/cgi-bin/carddisp.pl?gene=ATF6
SLC25A12	Solute Carrier Family 25 Member 12	Protein Coding	46	GC02M171783	8.42	https://www.genecards.org/cgi-bin/carddisp.pl?gene=SLC25A12
SLC25A17	Solute Carrier Family 25 Member 17	Protein Coding	39	GC22M045405	8.39	https://www.genecards.org/cgi-bin/carddisp.pl?gene=SLC25A17
HDAC1	Histone Deacetylase 1	Protein Coding	49	GC01P032292	8.35	https://www.genecards.org/cgi-bin/carddisp.pl?gene=HDAC1
UCP3	Uncoupling Protein 3	Protein Coding	43	GC11M074000	8.32	https://www.genecards.org/cgi-bin/carddisp.pl?gene=UCP3
FOXC2	Forkhead Box C2	Protein Coding	44	GC16P086567	8.32	https://www.genecards.org/cgi-bin/carddisp.pl?gene=FOXC2
GSTT1	Glutathione S-Transferase Theta 1	Protein Coding	32	GC22Mi00270	8.28	https://www.genecards.org/cgi-bin/carddisp.pl?gene=GSTT1
PLTP	Phospholipid Transfer Protein	Protein Coding	43	GC20M045898	8.24	https://www.genecards.org/cgi-bin/carddisp.pl?gene=PLTP
OGG1	8-Oxoguanine DNA Glycosylase	Protein Coding	47	GC03P009751	8.24	https://www.genecards.org/cgi-bin/carddisp.pl?gene=OGG1
ALAD	Aminolevulinate Dehydratase	Protein Coding	46	GC09M113386	8.21	https://www.genecards.org/cgi-bin/carddisp.pl?gene=ALAD

GABRA2	Gamma-Aminobutyric Acid Type A Receptor Subunit	Protein Coding	45	GC04M046167	8.19	https://www.genecards.org/cgi-bin/carddisp.pl?gene=GABRA2
SULT2A1	Sulfotransferase Family 2A Member 1	Protein Coding	44	GC19M047870	8.19	https://www.genecards.org/cgi-bin/carddisp.pl?gene=SULT2A1
DYSF	Dysferlin	Protein Coding	43	GC02P071453	8.17	https://www.genecards.org/cgi-bin/carddisp.pl?gene=DYSF
KLF6	Kruppel Like Factor 6	Protein Coding	44	GC10M003779	8.16	https://www.genecards.org/cgi-bin/carddisp.pl?gene=KLF6
POSTN	Periostin	Protein Coding	43	GC13M037562	8.15	https://www.genecards.org/cgi-bin/carddisp.pl?gene=POSTN
IL1RAPL2	Interleukin 1 Receptor Accessory Protein Like 2	Protein Coding	37	GC0XP104566	8.14	https://www.genecards.org/cgi-bin/carddisp.pl?gene=IL1RAPL2
SDC1	Syndecan 1	Protein Coding	43	GC02M020200	8.08	https://www.genecards.org/cgi-bin/carddisp.pl?gene=SDC1
SOST	Sclerostin	Protein Coding	44	GC17M043753	8.08	https://www.genecards.org/cgi-bin/carddisp.pl?gene=SOST
PHB	Prohibitin	Protein Coding	47	GC17M049404	8.03	https://www.genecards.org/cgi-bin/carddisp.pl?gene=PHB
TSPO	Translocator Protein	Protein Coding	43	GC22P043151	8.02	https://www.genecards.org/cgi-bin/carddisp.pl?gene=TSPO
IRF3	Interferon Regulatory Factor 3	Protein Coding	47	GC19M049659	8.02	https://www.genecards.org/cgi-bin/carddisp.pl?gene=IRF3
E2F1	E2F Transcription Factor 1	Protein Coding	43	GC20M033675	8.02	https://www.genecards.org/cgi-bin/carddisp.pl?gene=E2F1
IFNAR1	Interferon Alpha And Beta Receptor Subunit 1	Protein Coding	45	GC21P033324	8.02	https://www.genecards.org/cgi-bin/carddisp.pl?gene=IFNAR1
PRKCQ	Protein Kinase C Theta	Protein Coding	49	GC10M006393	8	https://www.genecards.org/cgi-bin/carddisp.pl?gene=PRKCQ
MIR224	MicroRNA 224	RNA Gene	17	GC0XM151958	7.97	https://www.genecards.org/cgi-bin/carddisp.pl?gene=MIR224
EPHX1	Epoxide Hydrolase 1	Protein Coding	47	GC01P225810	7.95	https://www.genecards.org/cgi-bin/carddisp.pl?gene=EPHX1
COX4I2	Cytochrome C Oxidase Subunit 4I2	Protein Coding	41	GC20P031637	7.91	https://www.genecards.org/cgi-bin/carddisp.pl?gene=COX4I2
NR1I3	Nuclear Receptor Subfamily 1 Group I Member 3	Protein Coding	44	GC01M161229	7.87	https://www.genecards.org/cgi-bin/carddisp.pl?gene=NR1I3
MIR423	MicroRNA 423	RNA Gene	18	GC17P030117	7.86	https://www.genecards.org/cgi-bin/carddisp.pl?gene=MIR423
COL1A1	Collagen Type I Alpha 1 Chain	Protein Coding	50	GC17M050183	7.86	https://www.genecards.org/cgi-bin/carddisp.pl?gene=COL1A1
TFEB	Transcription Factor EB	Protein Coding	42	GC06M042295	7.82	https://www.genecards.org/cgi-bin/carddisp.pl?gene=TFEB
GOT2	Glutamic-Oxaloacetic Transaminase 2	Protein Coding	45	GC16M058707	7.8	https://www.genecards.org/cgi-bin/carddisp.pl?gene=GOT2
MLN	Motilin	Protein Coding	35	GC06M033794	7.8	https://www.genecards.org/cgi-bin/carddisp.pl?gene=MLN
CD68	CD68 Molecule	Protein Coding	40	GC17P007579	7.78	https://www.genecards.org/cgi-bin/carddisp.pl?gene=CD68
IL33	Interleukin 33	Protein Coding	40	GC09P006206	7.78	https://www.genecards.org/cgi-bin/carddisp.pl?gene=IL33
RPS6KA3	Ribosomal Protein S6 Kinase A3	Protein Coding	52	GC0XM020149	7.77	https://www.genecards.org/cgi-bin/carddisp.pl?gene=RPS6KA3
LPIN1	Lipin 1	Protein Coding	48	GC02P011649	7.76	https://www.genecards.org/cgi-bin/carddisp.pl?gene=LPIN1
SFRP4	Secreted Frizzled Related Protein 4	Protein Coding	43	GC07M037912	7.76	https://www.genecards.org/cgi-bin/carddisp.pl?gene=SFRP4
UQCRFS1	Ubiquinol-Cytochrome C Reductase, Rieske Iron-Sulfu	Protein Coding	45	GC19M029205	7.74	https://www.genecards.org/cgi-bin/carddisp.pl?gene=UQCRFS1
SERPINA7	Serpin Family A Member 7	Protein Coding	39	GC0XM106032	7.72	https://www.genecards.org/cgi-bin/carddisp.pl?gene=SERPINA7
TRAF3IP2	TRAF3 Interacting Protein 2	Protein Coding	43	GC06M111555	7.69	https://www.genecards.org/cgi-bin/carddisp.pl?gene=TRAF3IP2
AXL	AXL Receptor Tyrosine Kinase	Protein Coding	51	GC19P041219	7.67	https://www.genecards.org/cgi-bin/carddisp.pl?gene=AXL
ANXA2	Annexin A2	Protein Coding	48	GC15M060347	7.67	https://www.genecards.org/cgi-bin/carddisp.pl?gene=ANXA2
CCR1	C-C Motif Chemokine Receptor 1	Protein Coding	45	GC03M046218	7.65	https://www.genecards.org/cgi-bin/carddisp.pl?gene=CCR1
RPL5	Ribosomal Protein L5	Protein Coding	47	GC01P092832	7.65	https://www.genecards.org/cgi-bin/carddisp.pl?gene=RPL5
GGT2	Gamma-Glutamyltransferase 2	Protein Coding	31	GC22M021207	7.64	https://www.genecards.org/cgi-bin/carddisp.pl?gene=GGT2
BID	BH3 Interacting Domain Death Agonist	Protein Coding	45	GC22M017734	7.63	https://www.genecards.org/cgi-bin/carddisp.pl?gene=BID
IL18BP	Interleukin 18 Binding Protein	Protein Coding	39	GC11P071998	7.61	https://www.genecards.org/cgi-bin/carddisp.pl?gene=IL18BP
NTS	Neurotensin	Protein Coding	40	GC12P085876	7.6	https://www.genecards.org/cgi-bin/carddisp.pl?gene=NTS

ACACB	Acetyl-CoA Carboxylase Beta	Protein Coding	45	GC12P109116	7.6	https://www.genecards.org/cgi-bin/carddisp.pl?gene=ACACB
GPC4	Glypican 4	Protein Coding	45	GC0XM133300	7.54	https://www.genecards.org/cgi-bin/carddisp.pl?gene=GPC4
MAP3K11	Mitogen-Activated Protein Kinase Kinase Kinase 11	Protein Coding	46	GC11M065598	7.5	https://www.genecards.org/cgi-bin/carddisp.pl?gene=MAP3K11
SLC39A14	Solute Carrier Family 39 Member 14	Protein Coding	43	GC08P022367	7.49	https://www.genecards.org/cgi-bin/carddisp.pl?gene=SLC39A14
CYP2B6	Cytochrome P450 Family 2 Subfamily B Member 6	Protein Coding	47	GC19P040991	7.48	https://www.genecards.org/cgi-bin/carddisp.pl?gene=CYP2B6
CCL20	C-C Motif Chemokine Ligand 20	Protein Coding	43	GC02P227813	7.46	https://www.genecards.org/cgi-bin/carddisp.pl?gene=CCL20
SLC39A4	Solute Carrier Family 39 Member 4	Protein Coding	43	GC08M144409	7.44	https://www.genecards.org/cgi-bin/carddisp.pl?gene=SLC39A4
NDUFB10	NADH:Ubiquinone Oxidoreductase Subunit B10	Protein Coding	41	GC16P002430	7.43	https://www.genecards.org/cgi-bin/carddisp.pl?gene=NDUFB10
FTO	FTO Alpha-Ketoglutarate Dependent Dioxygenase	Protein Coding	44	GC16P053737	7.4	https://www.genecards.org/cgi-bin/carddisp.pl?gene=FTO
TYSND1	Trypsin Domain Containing 1	Protein Coding	34	GC10M070137	7.39	https://www.genecards.org/cgi-bin/carddisp.pl?gene=TYSND1
GFPT1	Glutamine--Fructose-6-Phosphate Transaminase 1	Protein Coding	46	GC02M069283	7.37	https://www.genecards.org/cgi-bin/carddisp.pl?gene=GFPT1
CXCR5	C-X-C Motif Chemokine Receptor 5	Protein Coding	41	GC11P118898	7.36	https://www.genecards.org/cgi-bin/carddisp.pl?gene=CXCR5
ANGPTL8	Angiopoietin Like 8	Protein Coding	28	GC19P011238	7.36	https://www.genecards.org/cgi-bin/carddisp.pl?gene=ANGPTL8
CYP4A11	Cytochrome P450 Family 4 Subfamily A Member 11	Protein Coding	43	GC01M046929	7.34	https://www.genecards.org/cgi-bin/carddisp.pl?gene=CYP4A11
IARS1	Isoleucyl-TRNA Synthetase 1	Protein Coding	35	GC09M092211	7.28	https://www.genecards.org/cgi-bin/carddisp.pl?gene=IARS1
ABCC3	ATP Binding Cassette Subfamily C Member 3	Protein Coding	45	GC17P050634	7.27	https://www.genecards.org/cgi-bin/carddisp.pl?gene=ABCC3
EIF2AK2	Eukaryotic Translation Initiation Factor 2 Alpha Kinas	Protein Coding	45	GC02M037099	7.24	https://www.genecards.org/cgi-bin/carddisp.pl?gene=EIF2AK2
DNAH11	Dynein Axonemal Heavy Chain 11	Protein Coding	41	GC07P021543	7.22	https://www.genecards.org/cgi-bin/carddisp.pl?gene=DNAH11
COX7B	Cytochrome C Oxidase Subunit 7B	Protein Coding	40	GC0XP077899	7.22	https://www.genecards.org/cgi-bin/carddisp.pl?gene=COX7B
BCL2L11	BCL2 Like 11	Protein Coding	45	GC02P111119	7.21	https://www.genecards.org/cgi-bin/carddisp.pl?gene=BCL2L11
SAMM50	SAMM50 Sorting And Assembly Machinery Compone	Protein Coding	37	GC22P043955	7.2	https://www.genecards.org/cgi-bin/carddisp.pl?gene=SAMM50
ATP2A2	ATPase Sarcoplasmic/Endoplasmic Reticulum Ca2+ T	Protein Coding	51	GC12P110280	7.16	https://www.genecards.org/cgi-bin/carddisp.pl?gene=ATP2A2
PARVB	Parvin Beta	Protein Coding	39	GC22P043999	7.16	https://www.genecards.org/cgi-bin/carddisp.pl?gene=PARVB
PHGDH	Phosphoglycerate Dehydrogenase	Protein Coding	49	GC01P119660	7.14	https://www.genecards.org/cgi-bin/carddisp.pl?gene=PHGDH
DARS1	Aspartyl-TRNA Synthetase 1	Protein Coding	36	GC02M135905	7.12	https://www.genecards.org/cgi-bin/carddisp.pl?gene=DARS1
EGR1	Early Growth Response 1	Protein Coding	44	GC05P138465	7.1	https://www.genecards.org/cgi-bin/carddisp.pl?gene=EGR1
ENPP2	Ectonucleotide Pyrophosphatase/Phosphodiesterase 2	Protein Coding	44	GC08M119556	7.08	https://www.genecards.org/cgi-bin/carddisp.pl?gene=ENPP2
UFM1	Ubiquitin Fold Modifier 1	Protein Coding	40	GC13P038349	7.07	https://www.genecards.org/cgi-bin/carddisp.pl?gene=UFM1
GLP1R	Glucagon Like Peptide 1 Receptor	Protein Coding	46	GC06P039048	7.06	https://www.genecards.org/cgi-bin/carddisp.pl?gene=GLP1R
NDUFA4	NDUFA4 Mitochondrial Complex Associated	Protein Coding	42	GC07M010938	6.99	https://www.genecards.org/cgi-bin/carddisp.pl?gene=NDUFA4
TGM2	Transglutaminase 2	Protein Coding	48	GC20M038127	6.96	https://www.genecards.org/cgi-bin/carddisp.pl?gene=TGM2
IL6ST	Interleukin 6 Signal Transducer	Protein Coding	45	GC05M055935	6.96	https://www.genecards.org/cgi-bin/carddisp.pl?gene=IL6ST
TBP	TATA-Box Binding Protein	Protein Coding	48	GC06P170554	6.95	https://www.genecards.org/cgi-bin/carddisp.pl?gene=TBP
TRIB1	Tribbles Pseudokinase 1	Protein Coding	37	GC08P125430	6.94	https://www.genecards.org/cgi-bin/carddisp.pl?gene=TRIB1
COL12A1	Collagen Type XII Alpha 1 Chain	Protein Coding	41	GC06M075084	6.92	https://www.genecards.org/cgi-bin/carddisp.pl?gene=COL12A1
CNR2	Cannabinoid Receptor 2	Protein Coding	45	GC01M023870	6.92	https://www.genecards.org/cgi-bin/carddisp.pl?gene=CNR2
CANX	Calnexin	Protein Coding	44	GC05P179678	6.92	https://www.genecards.org/cgi-bin/carddisp.pl?gene=CANX
MYH14	Myosin Heavy Chain 14	Protein Coding	45	GC19P050192	6.85	https://www.genecards.org/cgi-bin/carddisp.pl?gene=MYH14
LOXL2	Lysyl Oxidase Like 2	Protein Coding	44	GC08M023296	6.84	https://www.genecards.org/cgi-bin/carddisp.pl?gene=LOXL2

AOC3	Amine Oxidase Copper Containing 3	Protein Coding	44	GC17P042851	6.8	https://www.genecards.org/cgi-bin/carddisp.pl?gene=AOC3
SAR1A	Secretion Associated Ras Related GTPase 1A	Protein Coding	41	GC10M070147	6.8	https://www.genecards.org/cgi-bin/carddisp.pl?gene=SAR1A
ASCL1	Achaete-Scute Family BHLH Transcription Factor 1	Protein Coding	44	GC12P102957	6.79	https://www.genecards.org/cgi-bin/carddisp.pl?gene=ASCL1
ACSM2B	Acyl-CoA Synthetase Medium Chain Family Member	Protein Coding	36	GC16M020547	6.76	https://www.genecards.org/cgi-bin/carddisp.pl?gene=ACSM2B
RPS17	Ribosomal Protein S17	Protein Coding	42	GC15M082536	6.75	https://www.genecards.org/cgi-bin/carddisp.pl?gene=RPS17
DRD4	Dopamine Receptor D4	Protein Coding	46	GC11P000660	6.75	https://www.genecards.org/cgi-bin/carddisp.pl?gene=DRD4
CEBPB	CCAAT Enhancer Binding Protein Beta	Protein Coding	43	GC20P050190	6.75	https://www.genecards.org/cgi-bin/carddisp.pl?gene=CEBPB
PTX3	Pentraxin 3	Protein Coding	41	GC03P157436	6.71	https://www.genecards.org/cgi-bin/carddisp.pl?gene=PTX3
OPRM1	Opioid Receptor Mu 1	Protein Coding	48	GC06P154075	6.69	https://www.genecards.org/cgi-bin/carddisp.pl?gene=OPRM1
OLR1	Oxidized Low Density Lipoprotein Receptor 1	Protein Coding	44	GC12M013864	6.68	https://www.genecards.org/cgi-bin/carddisp.pl?gene=OLR1
DCLK1	Doublecortin Like Kinase 1	Protein Coding	43	GC13M035768	6.66	https://www.genecards.org/cgi-bin/carddisp.pl?gene=DCLK1
DBH	Dopamine Beta-Hydroxylase	Protein Coding	50	GC09P133636	6.66	https://www.genecards.org/cgi-bin/carddisp.pl?gene=DBH
POR	Cytochrome P450 Oxidoreductase	Protein Coding	48	GC07P075899	6.62	https://www.genecards.org/cgi-bin/carddisp.pl?gene=POR
GSK3A	Glycogen Synthase Kinase 3 Alpha	Protein Coding	48	GC19M042230	6.61	https://www.genecards.org/cgi-bin/carddisp.pl?gene=GSK3A
HDAC9	Histone Deacetylase 9	Protein Coding	46	GC07P018086	6.61	https://www.genecards.org/cgi-bin/carddisp.pl?gene=HDAC9
OGT	O-Linked N-Acetylglucosamine (GlcNAc) Transferase	Protein Coding	44	GC0XP071534	6.6	https://www.genecards.org/cgi-bin/carddisp.pl?gene=OGT
EPHX2	Epoxide Hydrolase 2	Protein Coding	47	GC08P027490	6.55	https://www.genecards.org/cgi-bin/carddisp.pl?gene=EPHX2
STING1	Stimulator Of Interferon Response CGAMP Interactor	Protein Coding	34	GC05M139476	6.5	https://www.genecards.org/cgi-bin/carddisp.pl?gene=STING1
IGF2BP2	Insulin Like Growth Factor 2 MRNA Binding Protein [illegible]	Protein Coding	44	GC03M185643	6.49	https://www.genecards.org/cgi-bin/carddisp.pl?gene=IGF2BP2
SERPINB3	Serpin Family B Member 3	Protein Coding	42	GC18M063655	6.48	https://www.genecards.org/cgi-bin/carddisp.pl?gene=SERPINB3
NCAN	Neurocan	Protein Coding	41	GC19P023336	6.44	https://www.genecards.org/cgi-bin/carddisp.pl?gene=NCAN
FFAR4	Free Fatty Acid Receptor 4	Protein Coding	38	GC10P093566	6.42	https://www.genecards.org/cgi-bin/carddisp.pl?gene=FFAR4
SPTAN1	Spectrin Alpha, Non-Erythrocytic 1	Protein Coding	47	GC09P128552	6.4	https://www.genecards.org/cgi-bin/carddisp.pl?gene=SPTAN1
MATN3	Matrilin 3	Protein Coding	43	GC02M019992	6.39	https://www.genecards.org/cgi-bin/carddisp.pl?gene=MATN3
SRD5A2	Steroid 5 Alpha-Reductase 2	Protein Coding	43	GC02M031522	6.34	https://www.genecards.org/cgi-bin/carddisp.pl?gene=SRD5A2
ITGA8	Integrin Subunit Alpha 8	Protein Coding	42	GC10M015513	6.34	https://www.genecards.org/cgi-bin/carddisp.pl?gene=ITGA8
RPS3A	Ribosomal Protein S3A	Protein Coding	41	GC04P151099	6.31	https://www.genecards.org/cgi-bin/carddisp.pl?gene=RPS3A
SLC25A1	Solute Carrier Family 25 Member 1	Protein Coding	46	GC22M019176	6.3	https://www.genecards.org/cgi-bin/carddisp.pl?gene=SLC25A1
PTPRD	Protein Tyrosine Phosphatase Receptor Type D	Protein Coding	44	GC09M008307	6.27	https://www.genecards.org/cgi-bin/carddisp.pl?gene=PTPRD
ALDH9A1	Aldehyde Dehydrogenase 9 Family Member A1	Protein Coding	41	GC01M165667	6.26	https://www.genecards.org/cgi-bin/carddisp.pl?gene=ALDH9A1
PDXK	Pyridoxal Kinase	Protein Coding	47	GC21P043719	6.24	https://www.genecards.org/cgi-bin/carddisp.pl?gene=PDXK
TXNRD2	Thioredoxin Reductase 2	Protein Coding	46	GC22M019863	6.23	https://www.genecards.org/cgi-bin/carddisp.pl?gene=TXNRD2
MATR3	Matrin 3	Protein Coding	41	GC05P139274	6.22	https://www.genecards.org/cgi-bin/carddisp.pl?gene=MATR3
TXN	Thioredoxin	Protein Coding	45	GC09M110243	6.22	https://www.genecards.org/cgi-bin/carddisp.pl?gene=TXN
SLC1A3	Solute Carrier Family 1 Member 3	Protein Coding	50	GC05P036606	6.21	https://www.genecards.org/cgi-bin/carddisp.pl?gene=SLC1A3
IL16	Interleukin 16	Protein Coding	42	GC15P081159	6.19	https://www.genecards.org/cgi-bin/carddisp.pl?gene=IL16
SF3B1	Splicing Factor 3b Subunit 1	Protein Coding	44	GC02M197389	6.18	https://www.genecards.org/cgi-bin/carddisp.pl?gene=SF3B1
TRAF2	TNF Receptor Associated Factor 2	Protein Coding	44	GC09P136881	6.18	https://www.genecards.org/cgi-bin/carddisp.pl?gene=TRAF2
CPB2	Carboxypeptidase B2	Protein Coding	44	GC13M046053	6.16	https://www.genecards.org/cgi-bin/carddisp.pl?gene=CPB2

ATP1A1	ATPase Na+/K+ Transporting Subunit Alpha 1	Protein Coding	49	GC01P116372	6.16	https://www.genecards.org/cgi-bin/carddisp.pl?gene=ATP1A1
TKT	Transketolase	Protein Coding	46	GC03M053224	6.15	https://www.genecards.org/cgi-bin/carddisp.pl?gene=TKT
MBOAT7	Membrane Bound O-Acyltransferase Domain Containi	Protein Coding	40	GC19M054173	6.12	https://www.genecards.org/cgi-bin/carddisp.pl?gene=MBOAT7
COL13A1	Collagen Type XIII Alpha 1 Chain	Protein Coding	41	GC10P069801	6.12	https://www.genecards.org/cgi-bin/carddisp.pl?gene=COL13A1
GCLC	Glutamate-Cysteine Ligase Catalytic Subunit	Protein Coding	43	GC06M053497	6.1	https://www.genecards.org/cgi-bin/carddisp.pl?gene=GCLC
PDGFA	Platelet Derived Growth Factor Subunit A	Protein Coding	44	GC07M000497	6.09	https://www.genecards.org/cgi-bin/carddisp.pl?gene=PDGFA
SCAP	SREBF Chaperone	Protein Coding	41	GC03M047413	6.08	https://www.genecards.org/cgi-bin/carddisp.pl?gene=SCAP
PKM	Pyruvate Kinase M1/2	Protein Coding	45	GC15M072199	6.05	https://www.genecards.org/cgi-bin/carddisp.pl?gene=PKM
KHK	Ketohexokinase	Protein Coding	44	GC02P027086	6.05	https://www.genecards.org/cgi-bin/carddisp.pl?gene=KHK
RUNX3	RUNX Family Transcription Factor 3	Protein Coding	43	GC01M024899	6.05	https://www.genecards.org/cgi-bin/carddisp.pl?gene=RUNX3
ERLIN1	ER Lipid Raft Associated 1	Protein Coding	41	GC10M100150	6.05	https://www.genecards.org/cgi-bin/carddisp.pl?gene=ERLIN1
IMMT	Inner Membrane Mitochondrial Protein	Protein Coding	39	GC02M086144	6.04	https://www.genecards.org/cgi-bin/carddisp.pl?gene=IMMT
A2M	Alpha-2-Macroglobulin	Protein Coding	45	GC12M009067	6.02	https://www.genecards.org/cgi-bin/carddisp.pl?gene=A2M
ERLIN2	ER Lipid Raft Associated 2	Protein Coding	40	GC08P037736	6.01	https://www.genecards.org/cgi-bin/carddisp.pl?gene=ERLIN2
FST	Follistatin	Protein Coding	46	GC05P053480	6	https://www.genecards.org/cgi-bin/carddisp.pl?gene=FST
ALOX12	Arachidonate 12-Lipoxygenase, 12S Type	Protein Coding	43	GC17P006995	5.99	https://www.genecards.org/cgi-bin/carddisp.pl?gene=ALOX12
RB1CC1	RB1 Inducible Coiled-Coil 1	Protein Coding	42	GC08M052622	5.96	https://www.genecards.org/cgi-bin/carddisp.pl?gene=RB1CC1
IRGM	Immunity Related GTPase M	Protein Coding	38	GC05P150846	5.93	https://www.genecards.org/cgi-bin/carddisp.pl?gene=IRGM
CYP4F2	Cytochrome P450 Family 4 Subfamily F Member 2	Protein Coding	44	GC19M015878	5.91	https://www.genecards.org/cgi-bin/carddisp.pl?gene=CYP4F2
UBD	Ubiquitin D	Protein Coding	39	GC06M029556	5.88	https://www.genecards.org/cgi-bin/carddisp.pl?gene=UBD
CASP2	Caspase 2	Protein Coding	49	GC07P144979	5.87	https://www.genecards.org/cgi-bin/carddisp.pl?gene=CASP2
MIR331	MicroRNA 331	RNA Gene	18	GC12P095308	5.86	https://www.genecards.org/cgi-bin/carddisp.pl?gene=MIR331
SIK1	Salt Inducible Kinase 1	Protein Coding	46	GC21M043414	5.82	https://www.genecards.org/cgi-bin/carddisp.pl?gene=SIK1
COX6A2	Cytochrome C Oxidase Subunit 6A2	Protein Coding	38	GC16M031439	5.81	https://www.genecards.org/cgi-bin/carddisp.pl?gene=COX6A2
FGL1	Fibrinogen Like 1	Protein Coding	40	GC08M017864	5.78	https://www.genecards.org/cgi-bin/carddisp.pl?gene=FGL1
ITPR2	Inositol 1,4,5-Trisphosphate Receptor Type 2	Protein Coding	45	GC12M026336	5.76	https://www.genecards.org/cgi-bin/carddisp.pl?gene=ITPR2
UGT1A6	UDP Glucuronosyltransferase Family 1 Member A6	Protein Coding	40	GC02P233691	5.73	https://www.genecards.org/cgi-bin/carddisp.pl?gene=UGT1A6
HSPA1A	Heat Shock Protein Family A (Hsp70) Member 1A	Protein Coding	43	GC06P047326	5.73	https://www.genecards.org/cgi-bin/carddisp.pl?gene=HSPA1A
PTPRG	Protein Tyrosine Phosphatase Receptor Type G	Protein Coding	41	GC03P061522	5.72	https://www.genecards.org/cgi-bin/carddisp.pl?gene=PTPRG
PRKAG1	Protein Kinase AMP-Activated Non-Catalytic Subunit	Protein Coding	48	GC12M049002	5.71	https://www.genecards.org/cgi-bin/carddisp.pl?gene=PRKAG1
LBP	Lipopolysaccharide Binding Protein	Protein Coding	44	GC20P038346	5.7	https://www.genecards.org/cgi-bin/carddisp.pl?gene=LBP
VTN	Vitronectin	Protein Coding	44	GC17M029955	5.7	https://www.genecards.org/cgi-bin/carddisp.pl?gene=VTN
RDH5	Retinol Dehydrogenase 5	Protein Coding	46	GC12P055720	5.69	https://www.genecards.org/cgi-bin/carddisp.pl?gene=RDH5
PIN1	Peptidylprolyl Cis/Trans Isomerase, NIMA-Interacting	Protein Coding	47	GC19P009835	5.68	https://www.genecards.org/cgi-bin/carddisp.pl?gene=PIN1
P2RX7	Purinergic Receptor P2X 7	Protein Coding	45	GC12P122829	5.68	https://www.genecards.org/cgi-bin/carddisp.pl?gene=P2RX7
XRCC5	X-Ray Repair Cross Complementing 5	Protein Coding	44	GC02P216107	5.68	https://www.genecards.org/cgi-bin/carddisp.pl?gene=XRCC5
TRIM21	Tripartite Motif Containing 21	Protein Coding	42	GC11M004384	5.67	https://www.genecards.org/cgi-bin/carddisp.pl?gene=TRIM21
CXCL11	C-X-C Motif Chemokine Ligand 11	Protein Coding	40	GC04M076033	5.66	https://www.genecards.org/cgi-bin/carddisp.pl?gene=CXCL11
CCL19	C-C Motif Chemokine Ligand 19	Protein Coding	41	GC09M034692	5.64	https://www.genecards.org/cgi-bin/carddisp.pl?gene=CCL19

MIR24-1	MicroRNA 24-1	RNA Gene	18	GC09P095086	5.63	https://www.genecards.org/cgi-bin/carddisp.pl?gene=MIR24-1
PRKAB2	Protein Kinase AMP-Activated Non-Catalytic Subunit	Protein Coding	46	GC01M147155	5.59	https://www.genecards.org/cgi-bin/carddisp.pl?gene=PRKAB2
UGT1A9	UDP Glucuronosyltransferase Family 1 Member A9	Protein Coding	43	GC02P233671	5.58	https://www.genecards.org/cgi-bin/carddisp.pl?gene=UGT1A9
DMGDH	Dimethylglycine Dehydrogenase	Protein Coding	42	GC05M078997	5.57	https://www.genecards.org/cgi-bin/carddisp.pl?gene=DMGDH
UGT1A7	UDP Glucuronosyltransferase Family 1 Member A7	Protein Coding	39	GC02P233681	5.56	https://www.genecards.org/cgi-bin/carddisp.pl?gene=UGT1A7
GPNMB	Glycoprotein Nmb	Protein Coding	44	GC07P023238	5.56	https://www.genecards.org/cgi-bin/carddisp.pl?gene=GPNMB
CCR2	C-C Motif Chemokine Receptor 2	Protein Coding	45	GC03P046356	5.53	https://www.genecards.org/cgi-bin/carddisp.pl?gene=CCR2
SNTA1	Syntrophin Alpha 1	Protein Coding	43	GC20M033407	5.53	https://www.genecards.org/cgi-bin/carddisp.pl?gene=SNTA1
JAZF1	JAZF Zinc Finger 1	Protein Coding	39	GC07M027830	5.5	https://www.genecards.org/cgi-bin/carddisp.pl?gene=JAZF1
H4-16	H4 Histone 16	Protein Coding	33	GC12M014768	5.5	https://www.genecards.org/cgi-bin/carddisp.pl?gene=H4-16
CYP4F3	Cytochrome P450 Family 4 Subfamily F Member 3	Protein Coding	42	GC19P015640	5.49	https://www.genecards.org/cgi-bin/carddisp.pl?gene=CYP4F3
CD82	CD82 Molecule	Protein Coding	43	GC11P044586	5.48	https://www.genecards.org/cgi-bin/carddisp.pl?gene=CD82
CUL3	Cullin 3	Protein Coding	46	GC02M224470	5.47	https://www.genecards.org/cgi-bin/carddisp.pl?gene=CUL3
ACSL3	Acyl-CoA Synthetase Long Chain Family Member 3	Protein Coding	40	GC02P222860	5.46	https://www.genecards.org/cgi-bin/carddisp.pl?gene=ACSL3
TNFRSF4	TNF Receptor Superfamily Member 4	Protein Coding	43	GC01M001211	5.39	https://www.genecards.org/cgi-bin/carddisp.pl?gene=TNFRSF4
CDH4	Cadherin 4	Protein Coding	41	GC20P061252	5.38	https://www.genecards.org/cgi-bin/carddisp.pl?gene=CDH4
LECT2	Leukocyte Cell Derived Chemotaxin 2	Protein Coding	36	GC05M135922	5.37	https://www.genecards.org/cgi-bin/carddisp.pl?gene=LECT2
MIR30C1	MicroRNA 30c-1	RNA Gene	21	GC01P040757	5.35	https://www.genecards.org/cgi-bin/carddisp.pl?gene=MIR30C1
UGT1A8	UDP Glucuronosyltransferase Family 1 Member A8	Protein Coding	35	GC02P233618	5.34	https://www.genecards.org/cgi-bin/carddisp.pl?gene=UGT1A8
PRPF8	Pre-MRNA Processing Factor 8	Protein Coding	41	GC17M001650	5.33	https://www.genecards.org/cgi-bin/carddisp.pl?gene=PRPF8
MLX	MAX Dimerization Protein MLX	Protein Coding	43	GC17P042567	5.29	https://www.genecards.org/cgi-bin/carddisp.pl?gene=MLX
ZNF365	Zinc Finger Protein 365	Protein Coding	37	GC10P062374	5.29	https://www.genecards.org/cgi-bin/carddisp.pl?gene=ZNF365
FNDC5	Fibronectin Type III Domain Containing 5	Protein Coding	34	GC01M032864	5.29	https://www.genecards.org/cgi-bin/carddisp.pl?gene=FNDC5
NDUFA5	NADH:Ubiquinone Oxidoreductase Subunit A5	Protein Coding	41	GC07M123536	5.29	https://www.genecards.org/cgi-bin/carddisp.pl?gene=NDUFA5
FYN	FYN Proto-Oncogene, Src Family Tyrosine Kinase	Protein Coding	48	GC06M111660	5.26	https://www.genecards.org/cgi-bin/carddisp.pl?gene=FYN
CYLD	CYLD Lysine 63 Deubiquitinase	Protein Coding	48	GC16P050742	5.23	https://www.genecards.org/cgi-bin/carddisp.pl?gene=CYLD
HYOU1	Hypoxia Up-Regulated 1	Protein Coding	43	GC11M119045	5.21	https://www.genecards.org/cgi-bin/carddisp.pl?gene=HYOU1
EEA1	Early Endosome Antigen 1	Protein Coding	43	GC12M092770	5.2	https://www.genecards.org/cgi-bin/carddisp.pl?gene=EEA1
NOX4	NADPH Oxidase 4	Protein Coding	42	GC11M089324	5.19	https://www.genecards.org/cgi-bin/carddisp.pl?gene=NOX4
FOXA1	Forkhead Box A1	Protein Coding	43	GC14M037589	5.17	https://www.genecards.org/cgi-bin/carddisp.pl?gene=FOXA1
NDUFS5	NADH:Ubiquinone Oxidoreductase Subunit S5	Protein Coding	40	GC01P039026	5.17	https://www.genecards.org/cgi-bin/carddisp.pl?gene=NDUFS5
SERPINF1	Serpin Family F Member 1	Protein Coding	44	GC17P001761	5.16	https://www.genecards.org/cgi-bin/carddisp.pl?gene=SERPINF1
PRDX1	Peroxiredoxin 1	Protein Coding	49	GC01M045511	5.16	https://www.genecards.org/cgi-bin/carddisp.pl?gene=PRDX1
EFTUD2	Elongation Factor Tu GTP Binding Domain Containin	Protein Coding	42	GC17M044852	5.16	https://www.genecards.org/cgi-bin/carddisp.pl?gene=EFTUD2
YEATS2	YEATS Domain Containing 2	Protein Coding	36	GC03P183698	5.15	https://www.genecards.org/cgi-bin/carddisp.pl?gene=YEATS2
LPCAT3	Lysophosphatidylcholine Acyltransferase 3	Protein Coding	36	GC12M006976	5.12	https://www.genecards.org/cgi-bin/carddisp.pl?gene=LPCAT3
COX7A2	Cytochrome C Oxidase Subunit 7A2	Protein Coding	38	GC06M075237	5.12	https://www.genecards.org/cgi-bin/carddisp.pl?gene=COX7A2
CCT5	Chaperonin Containing TCP1 Subunit 5	Protein Coding	43	GC05P010236	5.1	https://www.genecards.org/cgi-bin/carddisp.pl?gene=CCT5
CLDN2	Claudin 2	Protein Coding	41	GC0XP106900	5.09	https://www.genecards.org/cgi-bin/carddisp.pl?gene=CLDN2

AFAP1-AS1	AFAP1 Antisense RNA 1	RNA Gene	17	GC04P007756	5.09	https://www.genecards.org/cgi-bin/carddisp.pl?gene=AFAP1-AS1
NNMT	Nicotinamide N-Methyltransferase	Protein Coding	45	GC11P114257	5.09	https://www.genecards.org/cgi-bin/carddisp.pl?gene=NNMT
YWHAE	Tyrosine 3-Monooxygenase/Tryptophan 5-Monooxyge	Protein Coding	50	GC17M001346	5.08	https://www.genecards.org/cgi-bin/carddisp.pl?gene=YWHAE
COX6C	Cytochrome C Oxidase Subunit 6C	Protein Coding	39	GC08M099899	5.03	https://www.genecards.org/cgi-bin/carddisp.pl?gene=COX6C
COPA	COPI Coat Complex Subunit Alpha	Protein Coding	41	GC01M160288	5.01	https://www.genecards.org/cgi-bin/carddisp.pl?gene=COPA
TRAPPC9	Trafficking Protein Particle Complex 9	Protein Coding	39	GC08M139728	5.01	https://www.genecards.org/cgi-bin/carddisp.pl?gene=TRAPPC9
COMMD1	Copper Metabolism Domain Containing 1	Protein Coding	42	GC02P061888	5	https://www.genecards.org/cgi-bin/carddisp.pl?gene=COMMD1
NDUFA8	NADH:Ubiquinone Oxidoreductase Subunit A8	Protein Coding	41	GC09M122132	4.99	https://www.genecards.org/cgi-bin/carddisp.pl?gene=NDUFA8
HNRNPK	Heterogeneous Nuclear Ribonucleoprotein K	Protein Coding	45	GC09M084056	4.98	https://www.genecards.org/cgi-bin/carddisp.pl?gene=HNRNPK
SERPINA12	Serpin Family A Member 12	Protein Coding	38	GC14M094526	4.96	https://www.genecards.org/cgi-bin/carddisp.pl?gene=SERPINA12
GOT1	Glutamic-Oxaloacetic Transaminase 1	Protein Coding	47	GC10M099396	4.96	https://www.genecards.org/cgi-bin/carddisp.pl?gene=GOT1
DISC1	DISC1 Scaffold Protein	Protein Coding	43	GC01P231626	4.95	https://www.genecards.org/cgi-bin/carddisp.pl?gene=DISC1
NAT1	N-Acetyltransferase 1	Protein Coding	44	GC08P018179	4.94	https://www.genecards.org/cgi-bin/carddisp.pl?gene=NAT1
TRAF1	TNF Receptor Associated Factor 1	Protein Coding	43	GC09M120902	4.93	https://www.genecards.org/cgi-bin/carddisp.pl?gene=TRAF1
XRCC6	X-Ray Repair Cross Complementing 6	Protein Coding	45	GC22P041622	4.91	https://www.genecards.org/cgi-bin/carddisp.pl?gene=XRCC6
PPP2CA	Protein Phosphatase 2 Catalytic Subunit Alpha	Protein Coding	48	GC05M134194	4.89	https://www.genecards.org/cgi-bin/carddisp.pl?gene=PPP2CA
CES2	Carboxylesterase 2	Protein Coding	43	GC16P066934	4.88	https://www.genecards.org/cgi-bin/carddisp.pl?gene=CES2
NDUFC2	NADH:Ubiquinone Oxidoreductase Subunit C2	Protein Coding	39	GC11M078068	4.87	https://www.genecards.org/cgi-bin/carddisp.pl?gene=NDUFC2
NDUFB6	NADH:Ubiquinone Oxidoreductase Subunit B6	Protein Coding	40	GC09M032553	4.87	https://www.genecards.org/cgi-bin/carddisp.pl?gene=NDUFB6
UQCR11	Ubiquinol-Cytochrome C Reductase, Complex III Subu	Protein Coding	37	GC19M001597	4.86	https://www.genecards.org/cgi-bin/carddisp.pl?gene=UQCR11
CYP2J2	Cytochrome P450 Family 2 Subfamily J Member 2	Protein Coding	44	GC01M059893	4.83	https://www.genecards.org/cgi-bin/carddisp.pl?gene=CYP2J2
UQCRC1	Ubiquinol-Cytochrome C Reductase Core Protein 1	Protein Coding	42	GC03M048598	4.83	https://www.genecards.org/cgi-bin/carddisp.pl?gene=UQCRC1
TNC	Tenascin C	Protein Coding	48	GC09M115019	4.82	https://www.genecards.org/cgi-bin/carddisp.pl?gene=TNC
CARS1	Cysteinyl-TRNA Synthetase 1	Protein Coding	32	GC11M003000	4.82	https://www.genecards.org/cgi-bin/carddisp.pl?gene=CARS1
TRAP1	TNF Receptor Associated Protein 1	Protein Coding	43	GC16M003652	4.78	https://www.genecards.org/cgi-bin/carddisp.pl?gene=TRAP1
RTN4	Reticulon 4	Protein Coding	44	GC02M054934	4.78	https://www.genecards.org/cgi-bin/carddisp.pl?gene=RTN4
PRSS2	Serine Protease 2	Protein Coding	39	GC07P144959	4.77	https://www.genecards.org/cgi-bin/carddisp.pl?gene=PRSS2
UGT1A4	UDP Glucuronosyltransferase Family 1 Member A4	Protein Coding	41	GC02P233718	4.75	https://www.genecards.org/cgi-bin/carddisp.pl?gene=UGT1A4
PDK4	Pyruvate Dehydrogenase Kinase 4	Protein Coding	45	GC07M095583	4.74	https://www.genecards.org/cgi-bin/carddisp.pl?gene=PDK4
YY1	YY1 Transcription Factor	Protein Coding	48	GC14P100238	4.72	https://www.genecards.org/cgi-bin/carddisp.pl?gene=YY1
JCAD	Junctional Cadherin 5 Associated	Protein Coding	26	GC10M030012	4.72	https://www.genecards.org/cgi-bin/carddisp.pl?gene=JCAD
SIRT6	Sirtuin 6	Protein Coding	45	GC19M004174	4.71	https://www.genecards.org/cgi-bin/carddisp.pl?gene=SIRT6
PLIN5	Perilipin 5	Protein Coding	33	GC19M004522	4.7	https://www.genecards.org/cgi-bin/carddisp.pl?gene=PLIN5
SNRNP200	Small Nuclear Ribonucleoprotein U5 Subunit 200	Protein Coding	41	GC02M096275	4.67	https://www.genecards.org/cgi-bin/carddisp.pl?gene=SNRNP200
MGAM	Maltase-Glucoamylase	Protein Coding	41	GC07P145052	4.65	https://www.genecards.org/cgi-bin/carddisp.pl?gene=MGAM
CDH5	Cadherin 5	Protein Coding	47	GC16P066366	4.63	https://www.genecards.org/cgi-bin/carddisp.pl?gene=CDH5
YBX1	Y-Box Binding Protein 1	Protein Coding	39	GC01P042682	4.63	https://www.genecards.org/cgi-bin/carddisp.pl?gene=YBX1
TP53BP1	Tumor Protein P53 Binding Protein 1	Protein Coding	44	GC15M043403	4.62	https://www.genecards.org/cgi-bin/carddisp.pl?gene=TP53BP1
NCOA3	Nuclear Receptor Coactivator 3	Protein Coding	45	GC20P047501	4.61	https://www.genecards.org/cgi-bin/carddisp.pl?gene=NCOA3

RPL10	Ribosomal Protein L10	Protein Coding	45	GC0XP154389	4.6	https://www.genecards.org/cgi-bin/carddisp.pl?gene=RPL10
UGT1A3	UDP Glucuronosyltransferase Family 1 Member A3	Protein Coding	38	GC02P233729	4.59	https://www.genecards.org/cgi-bin/carddisp.pl?gene=UGT1A3
STUB1	STIP1 Homology And U-Box Containing Protein 1	Protein Coding	45	GC16P001405	4.57	https://www.genecards.org/cgi-bin/carddisp.pl?gene=STUB1
SOD3	Superoxide Dismutase 3	Protein Coding	40	GC04P024798	4.56	https://www.genecards.org/cgi-bin/carddisp.pl?gene=SOD3
UBE2D2	Ubiquitin Conjugating Enzyme E2 D2	Protein Coding	44	GC05P139526	4.55	https://www.genecards.org/cgi-bin/carddisp.pl?gene=UBE2D2
ROCK1	Rho Associated Coiled-Coil Containing Protein Kinase	Protein Coding	50	GC18M020946	4.55	https://www.genecards.org/cgi-bin/carddisp.pl?gene=ROCK1
CACNA2D1	Calcium Voltage-Gated Channel Auxiliary Subunit Al	Protein Coding	45	GC07M081946	4.55	https://www.genecards.org/cgi-bin/carddisp.pl?gene=CACNA2D1
SLC34A2	Solute Carrier Family 34 Member 2	Protein Coding	45	GC04P025657	4.54	https://www.genecards.org/cgi-bin/carddisp.pl?gene=SLC34A2
AKR1B10	Aldo-Keto Reductase Family 1 Member B10	Protein Coding	43	GC07P134527	4.53	https://www.genecards.org/cgi-bin/carddisp.pl?gene=AKR1B10
H4C11	H4 Clustered Histone 11	Protein Coding	30	GC06P028800	4.52	https://www.genecards.org/cgi-bin/carddisp.pl?gene=H4C11
LGALS3BP	Galectin 3 Binding Protein	Protein Coding	41	GC17M078971	4.52	https://www.genecards.org/cgi-bin/carddisp.pl?gene=LGALS3BP
H4C1	H4 Clustered Histone 1	Protein Coding	31	GC06P026041	4.51	https://www.genecards.org/cgi-bin/carddisp.pl?gene=H4C1
SERPINH1	Serpin Family H Member 1	Protein Coding	45	GC11P075562	4.51	https://www.genecards.org/cgi-bin/carddisp.pl?gene=SERPINH1
SLC25A11	Solute Carrier Family 25 Member 11	Protein Coding	43	GC17M004937	4.48	https://www.genecards.org/cgi-bin/carddisp.pl?gene=SLC25A11
IFNL4	Interferon Lambda 4 (Gene/Pseudogene)	Protein Coding	19	GC19M039246	4.46	https://www.genecards.org/cgi-bin/carddisp.pl?gene=IFNL4
IL36A	Interleukin 36 Alpha	Protein Coding	37	GC02P113005	4.46	https://www.genecards.org/cgi-bin/carddisp.pl?gene=IL36A
ACE2	Angiotensin I Converting Enzyme 2	Protein Coding	48	GC0XM015562	4.45	https://www.genecards.org/cgi-bin/carddisp.pl?gene=ACE2
TXNRD1	Thioredoxin Reductase 1	Protein Coding	47	GC12P104215	4.43	https://www.genecards.org/cgi-bin/carddisp.pl?gene=TXNRD1
HPS1	HPS1 Biogenesis Of Lysosomal Organelles Complex 3	Protein Coding	41	GC10M098416	4.43	https://www.genecards.org/cgi-bin/carddisp.pl?gene=HPS1
KHDRBS3	KH RNA Binding Domain Containing, Signal Transdu	Protein Coding	37	GC08P135457	4.42	https://www.genecards.org/cgi-bin/carddisp.pl?gene=KHDRBS3
CFD	Complement Factor D	Protein Coding	44	GC19P000859	4.42	https://www.genecards.org/cgi-bin/carddisp.pl?gene=CFD
ITLN1	Intelectin 1	Protein Coding	39	GC01M160876	4.4	https://www.genecards.org/cgi-bin/carddisp.pl?gene=ITLN1
AMPH	Amphiphysin	Protein Coding	44	GC07M038666	4.4	https://www.genecards.org/cgi-bin/carddisp.pl?gene=AMPH
AZGP1	Alpha-2-Glycoprotein 1, Zinc-Binding	Protein Coding	43	GC07M099967	4.38	https://www.genecards.org/cgi-bin/carddisp.pl?gene=AZGP1
POMP	Proteasome Maturation Protein	Protein Coding	41	GC13P028659	4.35	https://www.genecards.org/cgi-bin/carddisp.pl?gene=POMP
SLC38A8	Solute Carrier Family 38 Member 8	Protein Coding	36	GC16M084009	4.33	https://www.genecards.org/cgi-bin/carddisp.pl?gene=SLC38A8
MMP11	Matrix Metallopeptidase 11	Protein Coding	45	GC22P023768	4.32	https://www.genecards.org/cgi-bin/carddisp.pl?gene=MMP11
H4C8	H4 Clustered Histone 8	Protein Coding	31	GC06M027323	4.31	https://www.genecards.org/cgi-bin/carddisp.pl?gene=H4C8
PEG10	Paternally Expressed 10	Protein Coding	40	GC07P094656	4.31	https://www.genecards.org/cgi-bin/carddisp.pl?gene=PEG10
H4C13	H4 Clustered Histone 13	Protein Coding	29	GC06M028372	4.3	https://www.genecards.org/cgi-bin/carddisp.pl?gene=H4C13
H4C9	H4 Clustered Histone 9	Protein Coding	31	GC06P028910	4.29	https://www.genecards.org/cgi-bin/carddisp.pl?gene=H4C9
NDUFA3	NADH:Ubiquinone Oxidoreductase Subunit A3	Protein Coding	39	GC19P054102	4.29	https://www.genecards.org/cgi-bin/carddisp.pl?gene=NDUFA3
PRKCE	Protein Kinase C Epsilon	Protein Coding	50	GC02P045651	4.28	https://www.genecards.org/cgi-bin/carddisp.pl?gene=PRKCE
COX8C	Cytochrome C Oxidase Subunit 8C	Protein Coding	32	GC14P093348	4.27	https://www.genecards.org/cgi-bin/carddisp.pl?gene=COX8C
H4C15	H4 Clustered Histone 15	Protein Coding	27	GC01M149964	4.27	https://www.genecards.org/cgi-bin/carddisp.pl?gene=H4C15
GSS	Glutathione Synthetase	Protein Coding	47	GC20M034928	4.26	https://www.genecards.org/cgi-bin/carddisp.pl?gene=GSS
NDUFB7	NADH:Ubiquinone Oxidoreductase Subunit B7	Protein Coding	39	GC19M014566	4.25	https://www.genecards.org/cgi-bin/carddisp.pl?gene=NDUFB7
LCN1	Lipocalin 1	Protein Coding	39	GC09P135521	4.24	https://www.genecards.org/cgi-bin/carddisp.pl?gene=LCN1
H4C4	H4 Clustered Histone 4	Protein Coding	28	GC06M027321	4.22	https://www.genecards.org/cgi-bin/carddisp.pl?gene=H4C4

H4C14	H4 Clustered Histone 14	Protein Coding	28	GC01P149832	4.22	https://www.genecards.org/cgi-bin/carddisp.pl?gene=H4C14
H4C12	H4 Clustered Histone 12	Protein Coding	29	GC06M028370	4.22	https://www.genecards.org/cgi-bin/carddisp.pl?gene=H4C12
IL18RAP	Interleukin 18 Receptor Accessory Protein	Protein Coding	39	GC02P102418	4.21	https://www.genecards.org/cgi-bin/carddisp.pl?gene=IL18RAP
H4C3	H4 Clustered Histone 3	Protein Coding	31	GC06P028681	4.21	https://www.genecards.org/cgi-bin/carddisp.pl?gene=H4C3
PRKAG3	Protein Kinase AMP-Activated Non-Catalytic Subunit	Protein Coding	43	GC02M218823	4.2	https://www.genecards.org/cgi-bin/carddisp.pl?gene=PRKAG3
DROSHA	Drosha Ribonuclease III	Protein Coding	41	GC05M031401	4.19	https://www.genecards.org/cgi-bin/carddisp.pl?gene=DROSHA
H4C6	H4 Clustered Histone 6	Protein Coding	29	GC06P028909	4.19	https://www.genecards.org/cgi-bin/carddisp.pl?gene=H4C6
H4C2	H4 Clustered Histone 2	Protein Coding	31	GC06M026026	4.18	https://www.genecards.org/cgi-bin/carddisp.pl?gene=H4C2
H4C5	H4 Clustered Histone 5	Protein Coding	28	GC06P028907	4.18	https://www.genecards.org/cgi-bin/carddisp.pl?gene=H4C5
HS3ST1	Heparan Sulfate-Glucosamine 3-Sulfotransferase 1	Protein Coding	42	GC04M011394	4.17	https://www.genecards.org/cgi-bin/carddisp.pl?gene=HS3ST1
RPL6	Ribosomal Protein L6	Protein Coding	39	GC12M112320	4.17	https://www.genecards.org/cgi-bin/carddisp.pl?gene=RPL6
UGT1A10	UDP Glucuronosyltransferase Family 1 Member A10	Protein Coding	40	GC02P233636	4.15	https://www.genecards.org/cgi-bin/carddisp.pl?gene=UGT1A10
PCLO	Piccolo Presynaptic Cytomatrix Protein	Protein Coding	41	GC07M082754	4.14	https://www.genecards.org/cgi-bin/carddisp.pl?gene=PCLO
NDUFB4	NADH:Ubiquinone Oxidoreductase Subunit B4	Protein Coding	40	GC03P120596	4.13	https://www.genecards.org/cgi-bin/carddisp.pl?gene=NDUFB4
NDUFB5	NADH:Ubiquinone Oxidoreductase Subunit B5	Protein Coding	36	GC03P179604	4.13	https://www.genecards.org/cgi-bin/carddisp.pl?gene=NDUFB5
CYP8B1	Cytochrome P450 Family 8 Subfamily B Member 1	Protein Coding	40	GC03M042856	4.12	https://www.genecards.org/cgi-bin/carddisp.pl?gene=CYP8B1
LCP1	Lymphocyte Cytosolic Protein 1	Protein Coding	41	GC13M046132	4.11	https://www.genecards.org/cgi-bin/carddisp.pl?gene=LCP1
SIPA1L2	Signal Induced Proliferation Associated 1 Like 2	Protein Coding	38	GC01M232397	4.1	https://www.genecards.org/cgi-bin/carddisp.pl?gene=SIPA1L2
PPP3CA	Protein Phosphatase 3 Catalytic Subunit Alpha	Protein Coding	52	GC04M101024	4.1	https://www.genecards.org/cgi-bin/carddisp.pl?gene=PPP3CA
LOC110806262	Solute Carrier Family 6 Member 4 Gene Promoter	Biological Region	1	GC17P030235	4.1	https://www.genecards.org/cgi-bin/carddisp.pl?gene=LOC11080626
SIRT4	Sirtuin 4	Protein Coding	40	GC12P120291	4.1	https://www.genecards.org/cgi-bin/carddisp.pl?gene=SIRT4
APLN	Apelin	Protein Coding	37	GC0XM129645	4.1	https://www.genecards.org/cgi-bin/carddisp.pl?gene=APLN
HIGD1A	HIG1 Hypoxia Inducible Domain Family Member 1A	Protein Coding	38	GC03M042782	4.1	https://www.genecards.org/cgi-bin/carddisp.pl?gene=HIGD1A
MAP3K7	Mitogen-Activated Protein Kinase Kinase Kinase 7	Protein Coding	51	GC06M090513	4.09	https://www.genecards.org/cgi-bin/carddisp.pl?gene=MAP3K7
NDUFB2	NADH:Ubiquinone Oxidoreductase Subunit B2	Protein Coding	39	GC07P140690	4.08	https://www.genecards.org/cgi-bin/carddisp.pl?gene=NDUFB2
PDGFC	Platelet Derived Growth Factor C	Protein Coding	43	GC04M156760	4.08	https://www.genecards.org/cgi-bin/carddisp.pl?gene=PDGFC
RORA	RAR Related Orphan Receptor A	Protein Coding	48	GC15M060488	4.07	https://www.genecards.org/cgi-bin/carddisp.pl?gene=RORA
TPM4	Tropomyosin 4	Protein Coding	40	GC19P023264	4.07	https://www.genecards.org/cgi-bin/carddisp.pl?gene=TPM4
COX5B	Cytochrome C Oxidase Subunit 5B	Protein Coding	41	GC02P097628	4.06	https://www.genecards.org/cgi-bin/carddisp.pl?gene=COX5B
PRKDC	Protein Kinase, DNA-Activated, Catalytic Subunit	Protein Coding	49	GC08M047773	4.05	https://www.genecards.org/cgi-bin/carddisp.pl?gene=PRKDC
SLC4A4	Solute Carrier Family 4 Member 4	Protein Coding	46	GC04P071063	4.01	https://www.genecards.org/cgi-bin/carddisp.pl?gene=SLC4A4
NDUFC1	NADH:Ubiquinone Oxidoreductase Subunit C1	Protein Coding	36	GC04M139267	4.01	https://www.genecards.org/cgi-bin/carddisp.pl?gene=NDUFC1
NDUFA7	NADH:Ubiquinone Oxidoreductase Subunit A7	Protein Coding	37	GC19M008308	4.01	https://www.genecards.org/cgi-bin/carddisp.pl?gene=NDUFA7
TFAP2B	Transcription Factor AP-2 Beta	Protein Coding	44	GC06P050894	4	https://www.genecards.org/cgi-bin/carddisp.pl?gene=TFAP2B
UQCR10	Ubiquinol-Cytochrome C Reductase, Complex III Subu	Protein Coding	37	GC22P029767	3.97	https://www.genecards.org/cgi-bin/carddisp.pl?gene=UQCR10
UQCRH	Ubiquinol-Cytochrome C Reductase Hinge Protein	Protein Coding	39	GC01P046303	3.96	https://www.genecards.org/cgi-bin/carddisp.pl?gene=UQCRH
NDUFB1	NADH:Ubiquinone Oxidoreductase Subunit B1	Protein Coding	37	GC14M092116	3.95	https://www.genecards.org/cgi-bin/carddisp.pl?gene=NDUFB1
MIR363	MicroRNA 363	RNA Gene	15	GC0XM134205	3.95	https://www.genecards.org/cgi-bin/carddisp.pl?gene=MIR363
NDUFV3	NADH:Ubiquinone Oxidoreductase Subunit V3	Protein Coding	40	GC21P042879	3.93	https://www.genecards.org/cgi-bin/carddisp.pl?gene=NDUFV3

NFIC	Nuclear Factor I C	Protein Coding	40	GC19P003314	3.92	https://www.genecards.org/cgi-bin/carddisp.pl?gene=NFIC
PZP	PZP Alpha-2-Macroglobulin Like	Protein Coding	39	GC12M009148	3.91	https://www.genecards.org/cgi-bin/carddisp.pl?gene=PZP
CFL1	Cofilin 1	Protein Coding	45	GC11M065823	3.91	https://www.genecards.org/cgi-bin/carddisp.pl?gene=CFL1
YIPF1	Yip1 Domain Family Member 1	Protein Coding	36	GC01M053851	3.9	https://www.genecards.org/cgi-bin/carddisp.pl?gene=YIPF1
COX7A1	Cytochrome C Oxidase Subunit 7A1	Protein Coding	37	GC19M042546	3.88	https://www.genecards.org/cgi-bin/carddisp.pl?gene=COX7A1
HNRNPU	Heterogeneous Nuclear Ribonucleoprotein U	Protein Coding	42	GC01M244844	3.88	https://www.genecards.org/cgi-bin/carddisp.pl?gene=HNRNPU
PUF60	Poly(U) Binding Splicing Factor 60	Protein Coding	41	GC08M143816	3.88	https://www.genecards.org/cgi-bin/carddisp.pl?gene=PUF60
CXCL16	C-X-C Motif Chemokine Ligand 16	Protein Coding	40	GC17M004733	3.87	https://www.genecards.org/cgi-bin/carddisp.pl?gene=CXCL16
AQP9	Aquaporin 9	Protein Coding	43	GC15P058138	3.87	https://www.genecards.org/cgi-bin/carddisp.pl?gene=AQP9
HSPA6	Heat Shock Protein Family A (Hsp70) Member 6	Protein Coding	44	GC01P161524	3.87	https://www.genecards.org/cgi-bin/carddisp.pl?gene=HSPA6
SIRT5	Sirtuin 5	Protein Coding	45	GC06P013574	3.86	https://www.genecards.org/cgi-bin/carddisp.pl?gene=SIRT5
COX7C	Cytochrome C Oxidase Subunit 7C	Protein Coding	38	GC05P086617	3.86	https://www.genecards.org/cgi-bin/carddisp.pl?gene=COX7C
ARRDC3	Arrestin Domain Containing 3	Protein Coding	37	GC05M091368	3.84	https://www.genecards.org/cgi-bin/carddisp.pl?gene=ARRDC3
PTPRU	Protein Tyrosine Phosphatase Receptor Type U	Protein Coding	41	GC01P029236	3.83	https://www.genecards.org/cgi-bin/carddisp.pl?gene=PTPRU
COX7A2L	Cytochrome C Oxidase Subunit 7A2 Like	Protein Coding	40	GC02M042312	3.82	https://www.genecards.org/cgi-bin/carddisp.pl?gene=COX7A2L
LEMD2	LEM Domain Nuclear Envelope Protein 2	Protein Coding	39	GC06M033772	3.82	https://www.genecards.org/cgi-bin/carddisp.pl?gene=LEMD2
HTR1B	5-Hydroxytryptamine Receptor 1B	Protein Coding	45	GC06M077478	3.81	https://www.genecards.org/cgi-bin/carddisp.pl?gene=HTR1B
AHNAK	AHNAK Nucleoprotein	Protein Coding	36	GC11M063360	3.8	https://www.genecards.org/cgi-bin/carddisp.pl?gene=AHNAK
CLTC	Clathrin Heavy Chain	Protein Coding	46	GC17P059619	3.8	https://www.genecards.org/cgi-bin/carddisp.pl?gene=CLTC
SLC9A9	Solute Carrier Family 9 Member A9	Protein Coding	39	GC03M143265	3.8	https://www.genecards.org/cgi-bin/carddisp.pl?gene=SLC9A9
ALPI	Alkaline Phosphatase, Intestinal	Protein Coding	43	GC02P232456	3.79	https://www.genecards.org/cgi-bin/carddisp.pl?gene=ALPI
NDUFA4L2	NDUFA4 Mitochondrial Complex Associated Like 2	Protein Coding	36	GC12M057234	3.79	https://www.genecards.org/cgi-bin/carddisp.pl?gene=NDUFA4L2
HSD17B13	Hydroxysteroid 17-Beta Dehydrogenase 13	Protein Coding	36	GC04M087303	3.78	https://www.genecards.org/cgi-bin/carddisp.pl?gene=HSD17B13
HDAC8	Histone Deacetylase 8	Protein Coding	47	GC0XM072329	3.77	https://www.genecards.org/cgi-bin/carddisp.pl?gene=HDAC8
RIPK3	Receptor Interacting Serine/Threonine Kinase 3	Protein Coding	43	GC14M024336	3.77	https://www.genecards.org/cgi-bin/carddisp.pl?gene=RIPK3
STEAP4	STEAP4 Metalloreductase	Protein Coding	40	GC07M088288	3.77	https://www.genecards.org/cgi-bin/carddisp.pl?gene=STEAP4
EEF1A1	Eukaryotic Translation Elongation Factor 1 Alpha 1	Protein Coding	43	GC06M073515	3.74	https://www.genecards.org/cgi-bin/carddisp.pl?gene=EEF1A1
MACROD2	Mono-ADP Ribosylhydrolase 2	Protein Coding	35	GC20P013925	3.73	https://www.genecards.org/cgi-bin/carddisp.pl?gene=MACROD2
SEMA5A	Semaphorin 5A	Protein Coding	41	GC05M009036	3.72	https://www.genecards.org/cgi-bin/carddisp.pl?gene=SEMA5A
ADH5	Alcohol Dehydrogenase 5 (Class III), Chi Polypeptide	Protein Coding	45	GC04M099070	3.71	https://www.genecards.org/cgi-bin/carddisp.pl?gene=ADH5
MAPK12	Mitogen-Activated Protein Kinase 12	Protein Coding	48	GC22M050246	3.7	https://www.genecards.org/cgi-bin/carddisp.pl?gene=MAPK12
CMKLR1	Chemerin Chemokine-Like Receptor 1	Protein Coding	40	GC12M108288	3.69	https://www.genecards.org/cgi-bin/carddisp.pl?gene=CMKLR1
IL34	Interleukin 34	Protein Coding	39	GC16P070613	3.65	https://www.genecards.org/cgi-bin/carddisp.pl?gene=IL34
ADH1A	Alcohol Dehydrogenase 1A (Class I), Alpha Polypepti	Protein Coding	42	GC04M099276	3.64	https://www.genecards.org/cgi-bin/carddisp.pl?gene=ADH1A
CCAR2	Cell Cycle And Apoptosis Regulator 2	Protein Coding	36	GC08P022604	3.63	https://www.genecards.org/cgi-bin/carddisp.pl?gene=CCAR2
CCT4	Chaperonin Containing TCP1 Subunit 4	Protein Coding	39	GC02M061868	3.63	https://www.genecards.org/cgi-bin/carddisp.pl?gene=CCT4
ESM1	Endothelial Cell Specific Molecule 1	Protein Coding	40	GC05M054977	3.63	https://www.genecards.org/cgi-bin/carddisp.pl?gene=ESM1
PNPLA7	Patatin Like Phospholipase Domain Containing 7	Protein Coding	35	GC09M137459	3.62	https://www.genecards.org/cgi-bin/carddisp.pl?gene=PNPLA7
ELK1	ETS Transcription Factor ELK1	Protein Coding	44	GC0XM047635	3.59	https://www.genecards.org/cgi-bin/carddisp.pl?gene=ELK1

EHBP1L1	EH Domain Binding Protein 1 Like 1	Protein Coding	32	GC11P065814	3.58	https://www.genecards.org/cgi-bin/carddisp.pl?gene=EHBP1L1
LINC01016	Long Intergenic Non-Protein Coding RNA 1016	RNA Gene	13	GC06M042348	3.56	https://www.genecards.org/cgi-bin/carddisp.pl?gene=LINC01016
KAT5	Lysine Acetyltransferase 5	Protein Coding	46	GC11P065711	3.56	https://www.genecards.org/cgi-bin/carddisp.pl?gene=KAT5
PSMC2	Proteasome 26S Subunit, ATPase 2	Protein Coding	40	GC07P103344	3.55	https://www.genecards.org/cgi-bin/carddisp.pl?gene=PSMC2
GRK2	G Protein-Coupled Receptor Kinase 2	Protein Coding	37	GC11P067266	3.55	https://www.genecards.org/cgi-bin/carddisp.pl?gene=GRK2
RPL3	Ribosomal Protein L3	Protein Coding	41	GC22M045394	3.54	https://www.genecards.org/cgi-bin/carddisp.pl?gene=RPL3
CD5L	CD5 Molecule Like	Protein Coding	39	GC01M157800	3.54	https://www.genecards.org/cgi-bin/carddisp.pl?gene=CD5L
TNFRSF12A	TNF Receptor Superfamily Member 12A	Protein Coding	44	GC16P003018	3.54	https://www.genecards.org/cgi-bin/carddisp.pl?gene=TNFRSF12A
COX7B2	Cytochrome C Oxidase Subunit 7B2	Protein Coding	32	GC04M046734	3.54	https://www.genecards.org/cgi-bin/carddisp.pl?gene=COX7B2
CRACR2A	Calcium Release Activated Channel Regulator 2A	Protein Coding	31	GC12M004102	3.52	https://www.genecards.org/cgi-bin/carddisp.pl?gene=CRACR2A
RPS15	Ribosomal Protein S15	Protein Coding	41	GC19P001438	3.51	https://www.genecards.org/cgi-bin/carddisp.pl?gene=RPS15
COX6B2	Cytochrome C Oxidase Subunit 6B2	Protein Coding	32	GC19M055350	3.51	https://www.genecards.org/cgi-bin/carddisp.pl?gene=COX6B2
NOS1AP	Nitric Oxide Synthase 1 Adaptor Protein	Protein Coding	37	GC01P162069	3.5	https://www.genecards.org/cgi-bin/carddisp.pl?gene=NOS1AP
SLC25A47	Solute Carrier Family 25 Member 47	Protein Coding	35	GC14P100323	3.5	https://www.genecards.org/cgi-bin/carddisp.pl?gene=SLC25A47
PRDX5	Peroxiredoxin 5	Protein Coding	46	GC11P064317	3.5	https://www.genecards.org/cgi-bin/carddisp.pl?gene=PRDX5
ATF3	Activating Transcription Factor 3	Protein Coding	44	GC01P212565	3.49	https://www.genecards.org/cgi-bin/carddisp.pl?gene=ATF3
TRAF3	TNF Receptor Associated Factor 3	Protein Coding	47	GC14P104639	3.49	https://www.genecards.org/cgi-bin/carddisp.pl?gene=TRAF3
USP7	Ubiquitin Specific Peptidase 7	Protein Coding	47	GC16M008892	3.49	https://www.genecards.org/cgi-bin/carddisp.pl?gene=USP7
NGF-AS1	NGF Antisense RNA 1	RNA Gene	9	GC01P115287	3.49	https://www.genecards.org/cgi-bin/carddisp.pl?gene=NGF-AS1
NDUFC2-KCTD14	NDUFC2-KCTD14 Readthrough	Protein Coding	21	GC11M078015	3.49	https://www.genecards.org/cgi-bin/carddisp.pl?gene=NDUFC2-KC
MIR103A2	MicroRNA 103a-2	RNA Gene	18	GC20P003917	3.48	https://www.genecards.org/cgi-bin/carddisp.pl?gene=MIR103A2
BIRC2	Baculoviral IAP Repeat Containing 2	Protein Coding	45	GC11P102347	3.47	https://www.genecards.org/cgi-bin/carddisp.pl?gene=BIRC2
IL1R2	Interleukin 1 Receptor Type 2	Protein Coding	45	GC02P101991	3.46	https://www.genecards.org/cgi-bin/carddisp.pl?gene=IL1R2
FARP1	FERM, ARH/RhoGEF And Pleckstrin Domain Protein	Protein Coding	38	GC13P098142	3.46	https://www.genecards.org/cgi-bin/carddisp.pl?gene=FARP1
GPN1	GPN-Loop GTPase 1	Protein Coding	37	GC02P027628	3.46	https://www.genecards.org/cgi-bin/carddisp.pl?gene=GPN1
NRG3	Neuregulin 3	Protein Coding	41	GC10P083672	3.45	https://www.genecards.org/cgi-bin/carddisp.pl?gene=NRG3
CISD2	CDGSH Iron Sulfur Domain 2	Protein Coding	41	GC04P102868	3.45	https://www.genecards.org/cgi-bin/carddisp.pl?gene=CISD2
PCSK5	Proprotein Convertase Subtilisin/Kexin Type 5	Protein Coding	41	GC09P075890	3.45	https://www.genecards.org/cgi-bin/carddisp.pl?gene=PCSK5
UQCRHL	Ubiquinol-Cytochrome C Reductase Hinge Protein Lik	Protein Coding	23	GC01M015808	3.45	https://www.genecards.org/cgi-bin/carddisp.pl?gene=UQCRHL
MIR361	MicroRNA 361	RNA Gene	16	GC0XM085903	3.43	https://www.genecards.org/cgi-bin/carddisp.pl?gene=MIR361
SDC2	Syndecan 2	Protein Coding	45	GC08P096494	3.42	https://www.genecards.org/cgi-bin/carddisp.pl?gene=SDC2
EGFL6	EGF Like Domain Multiple 6	Protein Coding	38	GC0XP013569	3.42	https://www.genecards.org/cgi-bin/carddisp.pl?gene=EGFL6
SLC25A3	Solute Carrier Family 25 Member 3	Protein Coding	45	GC12P098593	3.42	https://www.genecards.org/cgi-bin/carddisp.pl?gene=SLC25A3
INHBA	Inhibin Subunit Beta A	Protein Coding	45	GC07M041668	3.41	https://www.genecards.org/cgi-bin/carddisp.pl?gene=INHBA
FAM166B	Family With Sequence Similarity 166 Member B	Protein Coding	30	GC09M035554	3.41	https://www.genecards.org/cgi-bin/carddisp.pl?gene=FAM166B
ESRRG	Estrogen Related Receptor Gamma	Protein Coding	45	GC01M216503	3.4	https://www.genecards.org/cgi-bin/carddisp.pl?gene=ESRRG
DDX60L	DExD/H-Box 60 Like	Protein Coding	33	GC04M168356	3.4	https://www.genecards.org/cgi-bin/carddisp.pl?gene=DDX60L
AQP7	Aquaporin 7	Protein Coding	44	GC09M033384	3.4	https://www.genecards.org/cgi-bin/carddisp.pl?gene=AQP7
HNMT	Histamine N-Methyltransferase	Protein Coding	45	GC02P137964	3.39	https://www.genecards.org/cgi-bin/carddisp.pl?gene=HNMT

ADAMTS9	ADAM Metallopeptidase With Thrombospondin Type	Protein Coding	37	GC03M064501	3.39	https://www.genecards.org/cgi-bin/carddisp.pl?gene=ADAMTS9
DGCR8	DGCR8 Microprocessor Complex Subunit	Protein Coding	40	GC22P020080	3.39	https://www.genecards.org/cgi-bin/carddisp.pl?gene=DGCR8
RPTOR	Regulatory Associated Protein Of MTOR Complex 1	Protein Coding	44	GC17P080544	3.38	https://www.genecards.org/cgi-bin/carddisp.pl?gene=RPTOR
IGHG1	Immunoglobulin Heavy Constant Gamma 1 (G1m Mar	Protein Coding	32	GC14M105736	3.38	https://www.genecards.org/cgi-bin/carddisp.pl?gene=IGHG1
CAPSL	Calcyphosine Like	Protein Coding	34	GC05M035905	3.37	https://www.genecards.org/cgi-bin/carddisp.pl?gene=CAPSL
EDA	Ectodysplasin A	Protein Coding	41	GC0XP069618	3.37	https://www.genecards.org/cgi-bin/carddisp.pl?gene=EDA
MAP3K8	Mitogen-Activated Protein Kinase Kinase Kinase 8	Protein Coding	47	GC10P030458	3.37	https://www.genecards.org/cgi-bin/carddisp.pl?gene=MAP3K8
MIR103A1	MicroRNA 103a-1	RNA Gene	17	GC05M168560	3.35	https://www.genecards.org/cgi-bin/carddisp.pl?gene=MIR103A1
TAGLN	Transgelin	Protein Coding	43	GC11P117199	3.33	https://www.genecards.org/cgi-bin/carddisp.pl?gene=TAGLN
STX17	Syntaxin 17	Protein Coding	36	GC09P099906	3.33	https://www.genecards.org/cgi-bin/carddisp.pl?gene=STX17
TSHB	Thyroid Stimulating Hormone Subunit Beta	Protein Coding	42	GC01P115029	3.33	https://www.genecards.org/cgi-bin/carddisp.pl?gene=TSHB
PSMD2	Proteasome 26S Subunit, Non-ATPase 2	Protein Coding	43	GC03P184298	3.32	https://www.genecards.org/cgi-bin/carddisp.pl?gene=PSMD2
TP53BP2	Tumor Protein P53 Binding Protein 2	Protein Coding	42	GC01M223779	3.32	https://www.genecards.org/cgi-bin/carddisp.pl?gene=TP53BP2
UGDH	UDP-Glucose 6-Dehydrogenase	Protein Coding	45	GC04M039502	3.31	https://www.genecards.org/cgi-bin/carddisp.pl?gene=UGDH
LAPTM4A	Lysosomal Protein Transmembrane 4 Alpha	Protein Coding	38	GC02M020032	3.31	https://www.genecards.org/cgi-bin/carddisp.pl?gene=LAPTM4A
NMT2	N-Myristoyltransferase 2	Protein Coding	40	GC10M015144	3.31	https://www.genecards.org/cgi-bin/carddisp.pl?gene=NMT2
SOCS2	Suppressor Of Cytokine Signaling 2	Protein Coding	43	GC12P093569	3.3	https://www.genecards.org/cgi-bin/carddisp.pl?gene=SOCS2
PLPPR4	Phospholipid Phosphatase Related 4	Protein Coding	29	GC01P099262	3.3	https://www.genecards.org/cgi-bin/carddisp.pl?gene=PLPPR4
CX3CL1	C-X3-C Motif Chemokine Ligand 1	Protein Coding	42	GC16P057372	3.29	https://www.genecards.org/cgi-bin/carddisp.pl?gene=CX3CL1
AGAP1	ArfGAP With GTPase Domain, Ankyrin Repeat And P	Protein Coding	39	GC02P235494	3.28	https://www.genecards.org/cgi-bin/carddisp.pl?gene=AGAP1
RPL7	Ribosomal Protein L7	Protein Coding	41	GC08M073290	3.28	https://www.genecards.org/cgi-bin/carddisp.pl?gene=RPL7
MOGAT2	Monoacylglycerol O-Acyltransferase 2	Protein Coding	38	GC11P075717	3.26	https://www.genecards.org/cgi-bin/carddisp.pl?gene=MOGAT2
YWHAG	Tyrosine 3-Monooxygenase/Tryptophan 5-Monooxyge	Protein Coding	49	GC07M076327	3.25	https://www.genecards.org/cgi-bin/carddisp.pl?gene=YWHAG
GPLD1	Glycosylphosphatidylinositol Specific Phospholipase D	Protein Coding	41	GC06M024425	3.25	https://www.genecards.org/cgi-bin/carddisp.pl?gene=GPLD1
CCRL2	C-C Motif Chemokine Receptor Like 2	Protein Coding	39	GC03P046407	3.23	https://www.genecards.org/cgi-bin/carddisp.pl?gene=CCRL2
CTBP2	C-Terminal Binding Protein 2	Protein Coding	44	GC10M124984	3.22	https://www.genecards.org/cgi-bin/carddisp.pl?gene=CTBP2
UGT1A5	UDP Glucuronosyltransferase Family 1 Member A5	Protein Coding	33	GC02P233712	3.21	https://www.genecards.org/cgi-bin/carddisp.pl?gene=UGT1A5
ARNT	Aryl Hydrocarbon Receptor Nuclear Translocator	Protein Coding	43	GC01M150809	3.2	https://www.genecards.org/cgi-bin/carddisp.pl?gene=ARNT
CDK8	Cyclin Dependent Kinase 8	Protein Coding	47	GC13P026254	3.2	https://www.genecards.org/cgi-bin/carddisp.pl?gene=CDK8
RPN1	Ribophorin I	Protein Coding	42	GC03M128619	3.19	https://www.genecards.org/cgi-bin/carddisp.pl?gene=RPN1
GLO1	Glyoxalase I	Protein Coding	45	GC06M042239	3.17	https://www.genecards.org/cgi-bin/carddisp.pl?gene=GLO1
ST8SIA1	ST8 Alpha-N-Acetyl-Neuraminide Alpha-2,8-Sialyltra	Protein Coding	41	GC12M022063	3.16	https://www.genecards.org/cgi-bin/carddisp.pl?gene=ST8SIA1
CREB5	CAMP Responsive Element Binding Protein 5	Protein Coding	41	GC07P028305	3.16	https://www.genecards.org/cgi-bin/carddisp.pl?gene=CREB5
YBX3	Y-Box Binding Protein 3	Protein Coding	39	GC12M013875	3.16	https://www.genecards.org/cgi-bin/carddisp.pl?gene=YBX3
FAF2	Fas Associated Factor Family Member 2	Protein Coding	38	GC05P176447	3.15	https://www.genecards.org/cgi-bin/carddisp.pl?gene=FAF2
MLXIP	MLX Interacting Protein	Protein Coding	33	GC12P122078	3.14	https://www.genecards.org/cgi-bin/carddisp.pl?gene=MLXIP
AQP3	Aquaporin 3 (Gill Blood Group)	Protein Coding	47	GC09M033431	3.14	https://www.genecards.org/cgi-bin/carddisp.pl?gene=AQP3
CYGB	Cytoglobin	Protein Coding	38	GC17M076527	3.13	https://www.genecards.org/cgi-bin/carddisp.pl?gene=CYGB
YWHAQ	Tyrosine 3-Monooxygenase/Tryptophan 5-Monooxyge	Protein Coding	47	GC02M009583	3.12	https://www.genecards.org/cgi-bin/carddisp.pl?gene=YWHAQ

SLC46A3	Solute Carrier Family 46 Member 3	Protein Coding	36	GC13M028700	3.11	https://www.genecards.org/cgi-bin/carddisp.pl?gene=SLC46A3
FRK	Fyn Related Src Family Tyrosine Kinase	Protein Coding	44	GC06M115931	3.1	https://www.genecards.org/cgi-bin/carddisp.pl?gene=FRK
CCN4	Cellular Communication Network Factor 4	Protein Coding	31	GC08P133192	3.1	https://www.genecards.org/cgi-bin/carddisp.pl?gene=CCN4
FAP	Fibroblast Activation Protein Alpha	Protein Coding	42	GC02M162170	3.1	https://www.genecards.org/cgi-bin/carddisp.pl?gene=FAP
OSBPL5	Oxysterol Binding Protein Like 5	Protein Coding	40	GC11M003088	3.09	https://www.genecards.org/cgi-bin/carddisp.pl?gene=OSBPL5
TLCD4-RWDD3	TLCD4-RWDD3 Readthrough	Protein Coding	16	GC01P095119	3.07	https://www.genecards.org/cgi-bin/carddisp.pl?gene=TLCD4-RWD
SVIL	Supervillin	Protein Coding	39	GC10M030482	3.03	https://www.genecards.org/cgi-bin/carddisp.pl?gene=SVIL
AATF	Apoptosis Antagonizing Transcription Factor	Protein Coding	40	GC17P036948	3.03	https://www.genecards.org/cgi-bin/carddisp.pl?gene=AATF
ANKK1	Ankyrin Repeat And Kinase Domain Containing 1	Protein Coding	37	GC11P113387	3.03	https://www.genecards.org/cgi-bin/carddisp.pl?gene=ANKK1
CWF19L1	CWF19 Like Cell Cycle Control Factor 1	Protein Coding	39	GC10M100232	3.01	https://www.genecards.org/cgi-bin/carddisp.pl?gene=CWF19L1
CYP2A13	Cytochrome P450 Family 2 Subfamily A Member 13	Protein Coding	41	GC19P041088	3.01	https://www.genecards.org/cgi-bin/carddisp.pl?gene=CYP2A13
HCFC1	Host Cell Factor C1	Protein Coding	46	GC0XM153947	3	https://www.genecards.org/cgi-bin/carddisp.pl?gene=HCFC1
MIR873	MicroRNA 873	RNA Gene	16	GC09M028880	3	https://www.genecards.org/cgi-bin/carddisp.pl?gene=MIR873
CYP2F1	Cytochrome P450 Family 2 Subfamily F Member 1	Protein Coding	41	GC19P041114	3	https://www.genecards.org/cgi-bin/carddisp.pl?gene=CYP2F1
CALD1	Caldesmon 1	Protein Coding	42	GC07P134744	3	https://www.genecards.org/cgi-bin/carddisp.pl?gene=CALD1
HSP90AB1	Heat Shock Protein 90 Alpha Family Class B Member	Protein Coding	45	GC06P044246	2.99	https://www.genecards.org/cgi-bin/carddisp.pl?gene=HSP90AB1
ZFP90	ZFP90 Zinc Finger Protein	Protein Coding	36	GC16P068530	2.98	https://www.genecards.org/cgi-bin/carddisp.pl?gene=ZFP90
MYO6	Myosin VI	Protein Coding	45	GC06P075749	2.97	https://www.genecards.org/cgi-bin/carddisp.pl?gene=MYO6
ZNF512	Zinc Finger Protein 512	Protein Coding	32	GC02P027582	2.97	https://www.genecards.org/cgi-bin/carddisp.pl?gene=ZNF512
PDIA2	Protein Disulfide Isomerase Family A Member 2	Protein Coding	38	GC16P001384	2.96	https://www.genecards.org/cgi-bin/carddisp.pl?gene=PDIA2
FETUB	Fetuin B	Protein Coding	36	GC03P186635	2.95	https://www.genecards.org/cgi-bin/carddisp.pl?gene=FETUB
BCAT1	Branched Chain Amino Acid Transaminase 1	Protein Coding	46	GC12M024732	2.95	https://www.genecards.org/cgi-bin/carddisp.pl?gene=BCAT1
ADRB1	Adrenoceptor Beta 1	Protein Coding	48	GC10P114044	2.94	https://www.genecards.org/cgi-bin/carddisp.pl?gene=ADRB1
PCNX3	Pecanex 3	Protein Coding	25	GC11P065830	2.93	https://www.genecards.org/cgi-bin/carddisp.pl?gene=PCNX3
SLC3A2	Solute Carrier Family 3 Member 2	Protein Coding	42	GC11P062856	2.93	https://www.genecards.org/cgi-bin/carddisp.pl?gene=SLC3A2
TNR	Tenascin R	Protein Coding	41	GC01M175291	2.92	https://www.genecards.org/cgi-bin/carddisp.pl?gene=TNR
AEBP1	AE Binding Protein 1	Protein Coding	39	GC07P044106	2.92	https://www.genecards.org/cgi-bin/carddisp.pl?gene=AEBP1
UBQLN4	Ubiquilin 4	Protein Coding	40	GC01M156033	2.91	https://www.genecards.org/cgi-bin/carddisp.pl?gene=UBQLN4
SEL1L3	SEL1L Family Member 3	Protein Coding	33	GC04M025715	2.9	https://www.genecards.org/cgi-bin/carddisp.pl?gene=SEL1L3
TTF2	Transcription Termination Factor 2	Protein Coding	39	GC01P117060	2.9	https://www.genecards.org/cgi-bin/carddisp.pl?gene=TTF2
FOXK1	Forkhead Box K1	Protein Coding	35	GC07P004682	2.9	https://www.genecards.org/cgi-bin/carddisp.pl?gene=FOXK1
ZP4	Zona Pellucida Glycoprotein 4	Protein Coding	31	GC01M237877	2.88	https://www.genecards.org/cgi-bin/carddisp.pl?gene=ZP4
C2orf16	Chromosome 2 Open Reading Frame 16	Protein Coding	28	GC02P027546	2.88	https://www.genecards.org/cgi-bin/carddisp.pl?gene=C2orf16
TEX36	Testis Expressed 36	Protein Coding	29	GC10M125576	2.88	https://www.genecards.org/cgi-bin/carddisp.pl?gene=TEX36
PPIB	Peptidylprolyl Isomerase B	Protein Coding	50	GC15M064155	2.87	https://www.genecards.org/cgi-bin/carddisp.pl?gene=PPIB
RPL7A	Ribosomal Protein L7a	Protein Coding	42	GC09P133348	2.87	https://www.genecards.org/cgi-bin/carddisp.pl?gene=RPL7A
SORCS3	Sortilin Related VPS10 Domain Containing Receptor 3	Protein Coding	36	GC10P104642	2.87	https://www.genecards.org/cgi-bin/carddisp.pl?gene=SORCS3
SORCS2	Sortilin Related VPS10 Domain Containing Receptor 2	Protein Coding	36	GC04P007196	2.87	https://www.genecards.org/cgi-bin/carddisp.pl?gene=SORCS2
ARL2BPP6	ADP Ribosylation Factor Like GTPase 2 Binding Prote	Pseudogene	6	GC05P175290	2.87	https://www.genecards.org/cgi-bin/carddisp.pl?gene=ARL2BPP6

ATAD3A	ATPase Family AAA Domain Containing 3A	Protein Coding	39	GC01P001570	2.85	https://www.genecards.org/cgi-bin/carddisp.pl?gene=ATAD3A
CAD	Carbamoyl-Phosphate Synthetase 2, Aspartate Transca	Protein Coding	50	GC02P027217	2.84	https://www.genecards.org/cgi-bin/carddisp.pl?gene=CAD
SLC30A6	Solute Carrier Family 30 Member 6	Protein Coding	38	GC02P032166	2.84	https://www.genecards.org/cgi-bin/carddisp.pl?gene=SLC30A6
ADAMTS9-AS2	ADAMTS9 Antisense RNA 2	RNA Gene	16	GC03P064671	2.84	https://www.genecards.org/cgi-bin/carddisp.pl?gene=ADAMTS9-A
NDRG2	NDRG Family Member 2	Protein Coding	38	GC14M021016	2.83	https://www.genecards.org/cgi-bin/carddisp.pl?gene=NDRG2
VIL1	Villin 1	Protein Coding	39	GC02P218419	2.83	https://www.genecards.org/cgi-bin/carddisp.pl?gene=VIL1
DDX1	DEAD-Box Helicase 1	Protein Coding	43	GC02P015591	2.82	https://www.genecards.org/cgi-bin/carddisp.pl?gene=DDX1
NUCB2	Nucleobindin 2	Protein Coding	38	GC11P017221	2.82	https://www.genecards.org/cgi-bin/carddisp.pl?gene=NUCB2
CHST2	Carbohydrate Sulfotransferase 2	Protein Coding	37	GC03P143119	2.81	https://www.genecards.org/cgi-bin/carddisp.pl?gene=CHST2
GPT2	Glutamic--Pyruvic Transaminase 2	Protein Coding	46	GC16P046885	2.8	https://www.genecards.org/cgi-bin/carddisp.pl?gene=GPT2
MCM7	Minichromosome Maintenance Complex Component 7	Protein Coding	44	GC07M100092	2.8	https://www.genecards.org/cgi-bin/carddisp.pl?gene=MCM7
NFE2	Nuclear Factor, Erythroid 2	Protein Coding	40	GC12M054292	2.79	https://www.genecards.org/cgi-bin/carddisp.pl?gene=NFE2
MIR6090	MicroRNA 6090	RNA Gene	10	GC11P128522	2.79	https://www.genecards.org/cgi-bin/carddisp.pl?gene=MIR6090
APOF	Apolipoprotein F	Protein Coding	37	GC12M056361	2.78	https://www.genecards.org/cgi-bin/carddisp.pl?gene=APOF
CCNC	Cyclin C	Protein Coding	40	GC06M099542	2.78	https://www.genecards.org/cgi-bin/carddisp.pl?gene=CCNC
MYO1B	Myosin IB	Protein Coding	37	GC02P191246	2.78	https://www.genecards.org/cgi-bin/carddisp.pl?gene=MYO1B
PCBP1	Poly(RC) Binding Protein 1	Protein Coding	41	GC02P070087	2.78	https://www.genecards.org/cgi-bin/carddisp.pl?gene=PCBP1
FAM171A1	Family With Sequence Similarity 171 Member A1	Protein Coding	33	GC10M015254	2.78	https://www.genecards.org/cgi-bin/carddisp.pl?gene=FAM171A1
PTBP1	Polypyrimidine Tract Binding Protein 1	Protein Coding	41	GC19P000797	2.77	https://www.genecards.org/cgi-bin/carddisp.pl?gene=PTBP1
LOC643339	Uncharacterized LOC643339	RNA Gene	9	GC12M093004	2.77	https://www.genecards.org/cgi-bin/carddisp.pl?gene=LOC643339
RPL12	Ribosomal Protein L12	Protein Coding	41	GC09M127447	2.77	https://www.genecards.org/cgi-bin/carddisp.pl?gene=RPL12
NFE2L1	Nuclear Factor, Erythroid 2 Like 1	Protein Coding	43	GC17P048049	2.75	https://www.genecards.org/cgi-bin/carddisp.pl?gene=NFE2L1
BBC3	BCL2 Binding Component 3	Protein Coding	41	GC19M047220	2.74	https://www.genecards.org/cgi-bin/carddisp.pl?gene=BBC3
MYH10	Myosin Heavy Chain 10	Protein Coding	46	GC17M008474	2.74	https://www.genecards.org/cgi-bin/carddisp.pl?gene=MYH10
FAM107B	Family With Sequence Similarity 107 Member B	Protein Coding	35	GC10M014519	2.73	https://www.genecards.org/cgi-bin/carddisp.pl?gene=FAM107B
HSPH1	Heat Shock Protein Family H (Hsp110) Member 1	Protein Coding	43	GC13M031134	2.71	https://www.genecards.org/cgi-bin/carddisp.pl?gene=HSPH1
IAH1	Isoamyl Acetate Hydrolyzing Esterase 1 (Putative)	Protein Coding	33	GC02P009473	2.71	https://www.genecards.org/cgi-bin/carddisp.pl?gene=IAH1
SFRP5	Secreted Frizzled Related Protein 5	Protein Coding	39	GC10M097766	2.7	https://www.genecards.org/cgi-bin/carddisp.pl?gene=SFRP5
RPL9	Ribosomal Protein L9	Protein Coding	41	GC04M039452	2.69	https://www.genecards.org/cgi-bin/carddisp.pl?gene=RPL9
TRIB3	Tribbles Pseudokinase 3	Protein Coding	43	GC20P000361	2.69	https://www.genecards.org/cgi-bin/carddisp.pl?gene=TRIB3
COLGALT1	Collagen Beta(1-O)Galactosyltransferase 1	Protein Coding	36	GC19P017555	2.69	https://www.genecards.org/cgi-bin/carddisp.pl?gene=COLGALT1
RNU6-53P	RNA, U6 Small Nuclear 53, Pseudogene	Pseudogene	8	GC13M028703	2.69	https://www.genecards.org/cgi-bin/carddisp.pl?gene=RNU6-53P
MIR372	MicroRNA 372	RNA Gene	16	GC19P054631	2.68	https://www.genecards.org/cgi-bin/carddisp.pl?gene=MIR372
RND3	Rho Family GTPase 3	Protein Coding	41	GC02M150468	2.68	https://www.genecards.org/cgi-bin/carddisp.pl?gene=RND3
ARPC1B	Actin Related Protein 2/3 Complex Subunit 1B	Protein Coding	43	GC07P099374	2.68	https://www.genecards.org/cgi-bin/carddisp.pl?gene=ARPC1B
NCL	Nucleolin	Protein Coding	42	GC02M231453	2.67	https://www.genecards.org/cgi-bin/carddisp.pl?gene=NCL
KCNK7	Potassium Two Pore Domain Channel Subfamily K Me	Protein Coding	37	GC11M065592	2.67	https://www.genecards.org/cgi-bin/carddisp.pl?gene=KCNK7
MACROH2A2	MacroH2A.2 Histone	Protein Coding	29	GC10P070053	2.67	https://www.genecards.org/cgi-bin/carddisp.pl?gene=MACROH2A
PCBP2	Poly(RC) Binding Protein 2	Protein Coding	41	GC12P053452	2.67	https://www.genecards.org/cgi-bin/carddisp.pl?gene=PCBP2

DDX5	DEAD-Box Helicase 5	Protein Coding	46	GC17M064498	2.66	https://www.genecards.org/cgi-bin/carddisp.pl?gene=DDX5
TMBIM1	Transmembrane BAX Inhibitor Motif Containing 1	Protein Coding	35	GC02M218274	2.66	https://www.genecards.org/cgi-bin/carddisp.pl?gene=TMBIM1
HNRNPM	Heterogeneous Nuclear Ribonucleoprotein M	Protein Coding	39	GC19P008444	2.65	https://www.genecards.org/cgi-bin/carddisp.pl?gene=HNRNPM
DISC2	Disrupted In Schizophrenia 2	RNA Gene	18	GC01M231814	2.65	https://www.genecards.org/cgi-bin/carddisp.pl?gene=DISC2
SDK1	Sidekick Cell Adhesion Molecule 1	Protein Coding	36	GC07P003307	2.64	https://www.genecards.org/cgi-bin/carddisp.pl?gene=SDK1
SLC9A4	Solute Carrier Family 9 Member A4	Protein Coding	36	GC02P102456	2.64	https://www.genecards.org/cgi-bin/carddisp.pl?gene=SLC9A4
ATXN2L	Ataxin 2 Like	Protein Coding	37	GC16P029016	2.63	https://www.genecards.org/cgi-bin/carddisp.pl?gene=ATXN2L
PPP1CA	Protein Phosphatase 1 Catalytic Subunit Alpha	Protein Coding	48	GC11M067415	2.63	https://www.genecards.org/cgi-bin/carddisp.pl?gene=PPP1CA
PFKFB3	6-Phosphofructo-2-Kinase/Fructose-2,6-Biphosphatase	Protein Coding	44	GC10P006144	2.62	https://www.genecards.org/cgi-bin/carddisp.pl?gene=PFKFB3
POLR3H	RNA Polymerase III Subunit H	Protein Coding	37	GC22M041525	2.62	https://www.genecards.org/cgi-bin/carddisp.pl?gene=POLR3H
ETS1-AS1	ETS1 Antisense RNA 1	RNA Gene	11	GC11P128527	2.62	https://www.genecards.org/cgi-bin/carddisp.pl?gene=ETS1-AS1
RPL27	Ribosomal Protein L27	Protein Coding	42	GC17P042998	2.62	https://www.genecards.org/cgi-bin/carddisp.pl?gene=RPL27
APPL2	Adaptor Protein, Phosphotyrosine Interacting With PH	Protein Coding	35	GC12M105173	2.61	https://www.genecards.org/cgi-bin/carddisp.pl?gene=APPL2
L13304-005		RNA Gene	4	GC11M065320	2.61	https://www.genecards.org/cgi-bin/carddisp.pl?gene=L13304-005
KLHL6	Kelch Like Family Member 6	Protein Coding	37	GC03M183487	2.6	https://www.genecards.org/cgi-bin/carddisp.pl?gene=KLHL6
PSG1	Pregnancy Specific Beta-1-Glycoprotein 1	Protein Coding	39	GC19M042866	2.59	https://www.genecards.org/cgi-bin/carddisp.pl?gene=PSG1
NRG4	Neuregulin 4	Protein Coding	39	GC15M075935	2.59	https://www.genecards.org/cgi-bin/carddisp.pl?gene=NRG4
GRM4	Glutamate Metabotropic Receptor 4	Protein Coding	44	GC06M042205	2.58	https://www.genecards.org/cgi-bin/carddisp.pl?gene=GRM4
RPL30	Ribosomal Protein L30	Protein Coding	40	GC08M098024	2.58	https://www.genecards.org/cgi-bin/carddisp.pl?gene=RPL30
SMIM20	Small Integral Membrane Protein 20	Protein Coding	29	GC04P025865	2.58	https://www.genecards.org/cgi-bin/carddisp.pl?gene=SMIM20
HNRNPC	Heterogeneous Nuclear Ribonucleoprotein C	Protein Coding	41	GC14M021210	2.57	https://www.genecards.org/cgi-bin/carddisp.pl?gene=HNRNPC
MYO18A	Myosin XVIIIA	Protein Coding	37	GC17M029968	2.57	https://www.genecards.org/cgi-bin/carddisp.pl?gene=MYO18A
SENP3	SUMO Specific Peptidase 3	Protein Coding	40	GC17P008040	2.56	https://www.genecards.org/cgi-bin/carddisp.pl?gene=SENP3
CTSS	Cathepsin S	Protein Coding	45	GC01M150730	2.56	https://www.genecards.org/cgi-bin/carddisp.pl?gene=CTSS
KRTAP5-5	Keratin Associated Protein 5-5	Protein Coding	27	GC11P001629	2.56	https://www.genecards.org/cgi-bin/carddisp.pl?gene=KRTAP5-5
CNOT4	CCR4-NOT Transcription Complex Subunit 4	Protein Coding	40	GC07M135361	2.55	https://www.genecards.org/cgi-bin/carddisp.pl?gene=CNOT4
KIF16B	Kinesin Family Member 16B	Protein Coding	37	GC20M016272	2.54	https://www.genecards.org/cgi-bin/carddisp.pl?gene=KIF16B
RPS4X	Ribosomal Protein S4 X-Linked	Protein Coding	39	GC0XM072255	2.54	https://www.genecards.org/cgi-bin/carddisp.pl?gene=RPS4X
FOXN3	Forkhead Box N3	Protein Coding	38	GC14M094049	2.54	https://www.genecards.org/cgi-bin/carddisp.pl?gene=FOXN3
SERPINA4	Serpin Family A Member 4	Protein Coding	41	GC14P094561	2.54	https://www.genecards.org/cgi-bin/carddisp.pl?gene=SERPINA4
AGR3	Anterior Gradient 3, Protein Disulphide Isomerase Fan	Protein Coding	37	GC07M016854	2.53	https://www.genecards.org/cgi-bin/carddisp.pl?gene=AGR3
GGT3P	Gamma-Glutamyltransferase 3 Pseudogene	Pseudogene	18	GC22M018773	2.52	https://www.genecards.org/cgi-bin/carddisp.pl?gene=GGT3P
MYL6B	Myosin Light Chain 6B	Protein Coding	39	GC12P056201	2.52	https://www.genecards.org/cgi-bin/carddisp.pl?gene=MYL6B
POLR2D	RNA Polymerase II Subunit D	Protein Coding	41	GC02M127981	2.52	https://www.genecards.org/cgi-bin/carddisp.pl?gene=POLR2D
WWTR1	WW Domain Containing Transcription Regulator 1	Protein Coding	41	GC03M149517	2.52	https://www.genecards.org/cgi-bin/carddisp.pl?gene=WWTR1
PDE7B	Phosphodiesterase 7B	Protein Coding	39	GC06P135795	2.51	https://www.genecards.org/cgi-bin/carddisp.pl?gene=PDE7B
DDX60	DExD/H-Box Helicase 60	Protein Coding	35	GC04M168216	2.51	https://www.genecards.org/cgi-bin/carddisp.pl?gene=DDX60
DDX50	DExD-Box Helicase 50	Protein Coding	39	GC10P068901	2.51	https://www.genecards.org/cgi-bin/carddisp.pl?gene=DDX50
SLC6A9	Solute Carrier Family 6 Member 9	Protein Coding	48	GC01M043991	2.51	https://www.genecards.org/cgi-bin/carddisp.pl?gene=SLC6A9

piR-37045		RNA Gene	2	GC22P044002	2.5	https://www.genecards.org/cgi-bin/carddisp.pl?gene=piR-37045
lnc-PNPLA5-2		RNA Gene	2	GC22M045578	2.5	https://www.genecards.org/cgi-bin/carddisp.pl?gene=lnc-PNPLA5-
APLNR	Apelin Receptor	Protein Coding	43	GC11M057233	2.49	https://www.genecards.org/cgi-bin/carddisp.pl?gene=APLNR
RUVBL2	RuvB Like AAA ATPase 2	Protein Coding	44	GC19P048993	2.49	https://www.genecards.org/cgi-bin/carddisp.pl?gene=RUVBL2
C1QTNF3	C1q And TNF Related 3	Protein Coding	38	GC05M034017	2.47	https://www.genecards.org/cgi-bin/carddisp.pl?gene=C1QTNF3
AP2B1	Adaptor Related Protein Complex 2 Subunit Beta 1	Protein Coding	42	GC17P035578	2.47	https://www.genecards.org/cgi-bin/carddisp.pl?gene=AP2B1
ENSG00000253111		RNA Gene	7	GC08P125466	2.47	https://www.genecards.org/cgi-bin/carddisp.pl?gene=ENSG0000002
LINC02694	Long Intergenic Non-Protein Coding RNA 2694	RNA Gene	24	GC15P038630	2.46	https://www.genecards.org/cgi-bin/carddisp.pl?gene=LINC02694
MCM3	Minichromosome Maintenance Complex Component 3	Protein Coding	45	GC06M052264	2.46	https://www.genecards.org/cgi-bin/carddisp.pl?gene=MCM3
GCN1	GCN1 Activator Of EIF2AK4	Protein Coding	28	GC12M120128	2.46	https://www.genecards.org/cgi-bin/carddisp.pl?gene=GCN1
MRTFA	Myocardin Related Transcription Factor A	Protein Coding	34	GC22M045655	2.45	https://www.genecards.org/cgi-bin/carddisp.pl?gene=MRTFA
SIRT7	Sirtuin 7	Protein Coding	41	GC17M081911	2.45	https://www.genecards.org/cgi-bin/carddisp.pl?gene=SIRT7
YWHAB	Tyrosine 3-Monooxygenase/Tryptophan 5-Monooxyge	Protein Coding	48	GC20P044885	2.45	https://www.genecards.org/cgi-bin/carddisp.pl?gene=YWHAB
TUBA1C	Tubulin Alpha 1c	Protein Coding	41	GC12P049188	2.45	https://www.genecards.org/cgi-bin/carddisp.pl?gene=TUBA1C
ONECUT2	One Cut Homeobox 2	Protein Coding	35	GC18P057436	2.45	https://www.genecards.org/cgi-bin/carddisp.pl?gene=ONECUT2
GATAD2A	GATA Zinc Finger Domain Containing 2A	Protein Coding	37	GC19P023337	2.45	https://www.genecards.org/cgi-bin/carddisp.pl?gene=GATAD2A
SRSF6	Serine And Arginine Rich Splicing Factor 6	Protein Coding	39	GC20P043457	2.44	https://www.genecards.org/cgi-bin/carddisp.pl?gene=SRSF6
PWWP3A	PWWP Domain Containing 3A, DNA Repair Factor	Protein Coding	20	GC19P001364	2.44	https://www.genecards.org/cgi-bin/carddisp.pl?gene=PWWP3A
MCM5	Minichromosome Maintenance Complex Component 5	Protein Coding	44	GC22P035400	2.44	https://www.genecards.org/cgi-bin/carddisp.pl?gene=MCM5
WSPAR	WNT Signaling Pathway Activating Non-Coding RNA	RNA Gene	11	GC05P133914	2.43	https://www.genecards.org/cgi-bin/carddisp.pl?gene=WSPAR
ATP5F1B	ATP Synthase F1 Subunit Beta	Protein Coding	33	GC12M056639	2.43	https://www.genecards.org/cgi-bin/carddisp.pl?gene=ATP5F1B
RPL13	Ribosomal Protein L13	Protein Coding	41	GC16P089640	2.43	https://www.genecards.org/cgi-bin/carddisp.pl?gene=RPL13
CLRN1-AS1	CLRN1 Antisense RNA 1	RNA Gene	16	GC03P150852	2.42	https://www.genecards.org/cgi-bin/carddisp.pl?gene=CLRN1-AS1
GSDMD	Gasdermin D	Protein Coding	39	GC08P143553	2.41	https://www.genecards.org/cgi-bin/carddisp.pl?gene=GSDMD
CCT3	Chaperonin Containing TCP1 Subunit 3	Protein Coding	41	GC01M156308	2.41	https://www.genecards.org/cgi-bin/carddisp.pl?gene=CCT3
H1-4	H1.4 Linker Histone, Cluster Member	Protein Coding	33	GC06P028871	2.41	https://www.genecards.org/cgi-bin/carddisp.pl?gene=H1-4
MCF2L2	MCF.2 Cell Line Derived Transforming Sequence-Lik	Protein Coding	34	GC03M183183	2.4	https://www.genecards.org/cgi-bin/carddisp.pl?gene=MCF2L2
RPS2	Ribosomal Protein S2	Protein Coding	43	GC16M002198	2.4	https://www.genecards.org/cgi-bin/carddisp.pl?gene=RPS2
TMOD3	Tropomodulin 3	Protein Coding	39	GC15P051829	2.4	https://www.genecards.org/cgi-bin/carddisp.pl?gene=TMOD3
RPLP2	Ribosomal Protein Lateral Stalk Subunit P2	Protein Coding	41	GC11P000891	2.4	https://www.genecards.org/cgi-bin/carddisp.pl?gene=RPLP2
MYO1C	Myosin IC	Protein Coding	43	GC17M001464	2.4	https://www.genecards.org/cgi-bin/carddisp.pl?gene=MYO1C
SNHG18	Small Nucleolar RNA Host Gene 18	RNA Gene	14	GC05P009562	2.4	https://www.genecards.org/cgi-bin/carddisp.pl?gene=SNHG18
NUDT4	Nudix Hydrolase 4	Protein Coding	39	GC12P093392	2.39	https://www.genecards.org/cgi-bin/carddisp.pl?gene=NUDT4
IGF2BP1	Insulin Like Growth Factor 2 MRNA Binding Protein 1	Protein Coding	40	GC17P048997	2.39	https://www.genecards.org/cgi-bin/carddisp.pl?gene=IGF2BP1
ACTR5	Actin Related Protein 5	Protein Coding	35	GC20P038748	2.39	https://www.genecards.org/cgi-bin/carddisp.pl?gene=ACTR5
YWHAZ	Tyrosine 3-Monooxygenase/Tryptophan 5-Monooxyge	Protein Coding	48	GC08M100917	2.38	https://www.genecards.org/cgi-bin/carddisp.pl?gene=YWHAZ
TSNAX-DISC1	TSNAX-DISC1 Readthrough (NMD Candidate)	RNA Gene	19	GC01P231528	2.38	https://www.genecards.org/cgi-bin/carddisp.pl?gene=TSNAX-DISC
CCT2	Chaperonin Containing TCP1 Subunit 2	Protein Coding	40	GC12P069585	2.38	https://www.genecards.org/cgi-bin/carddisp.pl?gene=CCT2
SLC25A10	Solute Carrier Family 25 Member 10	Protein Coding	42	GC17P081712	2.38	https://www.genecards.org/cgi-bin/carddisp.pl?gene=SLC25A10

RPS15A	Ribosomal Protein S15a	Protein Coding	41	GC16M018781	2.38	https://www.genecards.org/cgi-bin/carddisp.pl?gene=RPS15A
lnc-PUM2-3		RNA Gene	4	GC02M020202	2.38	https://www.genecards.org/cgi-bin/carddisp.pl?gene=lnc-PUM2-3
lnc-RHOB-11		RNA Gene	4	GC02P020182	2.38	https://www.genecards.org/cgi-bin/carddisp.pl?gene=lnc-RHOB-11
RPS23	Ribosomal Protein S23	Protein Coding	40	GC05M082273	2.38	https://www.genecards.org/cgi-bin/carddisp.pl?gene=RPS23
CILP2	Cartilage Intermediate Layer Protein 2	Protein Coding	35	GC19P019538	2.37	https://www.genecards.org/cgi-bin/carddisp.pl?gene=CILP2
LINC01679	Long Intergenic Non-Protein Coding RNA 1679	RNA Gene	9	GC21M043360	2.37	https://www.genecards.org/cgi-bin/carddisp.pl?gene=LINC01679
RPS11	Ribosomal Protein S11	Protein Coding	39	GC19P049496	2.37	https://www.genecards.org/cgi-bin/carddisp.pl?gene=RPS11
RESF1	Retroelement Silencing Factor 1	Protein Coding	25	GC12P031982	2.37	https://www.genecards.org/cgi-bin/carddisp.pl?gene=RESF1
C16orf95	Chromosome 16 Open Reading Frame 95	Protein Coding	26	GC16M087119	2.37	https://www.genecards.org/cgi-bin/carddisp.pl?gene=C16orf95
IP6K3	Inositol Hexakisphosphate Kinase 3	Protein Coding	39	GC06M033721	2.36	https://www.genecards.org/cgi-bin/carddisp.pl?gene=IP6K3
MIR1296	MicroRNA 1296	RNA Gene	16	GC10M063372	2.36	https://www.genecards.org/cgi-bin/carddisp.pl?gene=MIR1296
XPO4	Exportin 4	Protein Coding	37	GC13M020777	2.36	https://www.genecards.org/cgi-bin/carddisp.pl?gene=XPO4
ENSG00000287092		RNA Gene	5	GC06M155911	2.35	https://www.genecards.org/cgi-bin/carddisp.pl?gene=ENSG000002
LINC00917	Long Intergenic Non-Protein Coding RNA 917	RNA Gene	14	GC16M086366	2.35	https://www.genecards.org/cgi-bin/carddisp.pl?gene=LINC00917
LINC00322	Long Intergenic Non-Protein Coding RNA 322	RNA Gene	17	GC21M043322	2.34	https://www.genecards.org/cgi-bin/carddisp.pl?gene=LINC00322
RPL10A	Ribosomal Protein L10a	Protein Coding	41	GC06P047437	2.34	https://www.genecards.org/cgi-bin/carddisp.pl?gene=RPL10A
DNAJA1	DnaJ Heat Shock Protein Family (Hsp40) Member A1	Protein Coding	41	GC09P033025	2.34	https://www.genecards.org/cgi-bin/carddisp.pl?gene=DNAJA1
SAT1	Spermidine/Spermine N1-Acetyltransferase 1	Protein Coding	45	GC0XP023784	2.34	https://www.genecards.org/cgi-bin/carddisp.pl?gene=SAT1
ZNF101	Zinc Finger Protein 101	Protein Coding	34	GC19P023339	2.33	https://www.genecards.org/cgi-bin/carddisp.pl?gene=ZNF101
QARS1	Glutaminyl-TRNA Synthetase 1	Protein Coding	26	GC03M049240	2.33	https://www.genecards.org/cgi-bin/carddisp.pl?gene=QARS1
TCP1	T-Complex 1	Protein Coding	41	GC06M159778	2.33	https://www.genecards.org/cgi-bin/carddisp.pl?gene=TCP1
SLC30A9	Solute Carrier Family 30 Member 9	Protein Coding	41	GC04P041992	2.33	https://www.genecards.org/cgi-bin/carddisp.pl?gene=SLC30A9
GRIK3	Glutamate Ionotropic Receptor Kainate Type Subunit 3	Protein Coding	43	GC01M036795	2.32	https://www.genecards.org/cgi-bin/carddisp.pl?gene=GRIK3
SP4	Sp4 Transcription Factor	Protein Coding	41	GC07P021434	2.32	https://www.genecards.org/cgi-bin/carddisp.pl?gene=SP4
ENSG00000254599		RNA Gene	5	GC11P098676	2.32	https://www.genecards.org/cgi-bin/carddisp.pl?gene=ENSG000002
lnc-CRYAA-1		RNA Gene	4	GC21P043356	2.32	https://www.genecards.org/cgi-bin/carddisp.pl?gene=lnc-CRYAA-1
lnc-JCAD-2		RNA Gene	4	GC10M030507	2.32	https://www.genecards.org/cgi-bin/carddisp.pl?gene=lnc-JCAD-2
lnc-CRYAA-17		RNA Gene	3	GC21P043341	2.32	https://www.genecards.org/cgi-bin/carddisp.pl?gene=lnc-CRYAA-1
ENSG00000226026		Uncategorized	8	GC01M095162	2.32	https://www.genecards.org/cgi-bin/carddisp.pl?gene=ENSG000002
RPL4	Ribosomal Protein L4	Protein Coding	41	GC15M066498	2.31	https://www.genecards.org/cgi-bin/carddisp.pl?gene=RPL4
NUDT21	Nudix Hydrolase 21	Protein Coding	37	GC16M056429	2.3	https://www.genecards.org/cgi-bin/carddisp.pl?gene=NUDT21
MYO1D	Myosin ID	Protein Coding	40	GC17M032492	2.3	https://www.genecards.org/cgi-bin/carddisp.pl?gene=MYO1D
STRA8	Stimulated By Retinoic Acid 8	Protein Coding	35	GC07P135231	2.3	https://www.genecards.org/cgi-bin/carddisp.pl?gene=STRA8
MIR646HG	MIR646 Host Gene	RNA Gene	14	GC20P060088	2.3	https://www.genecards.org/cgi-bin/carddisp.pl?gene=MIR646HG
ENSG00000231487		RNA Gene	6	GC11M001683	2.3	https://www.genecards.org/cgi-bin/carddisp.pl?gene=ENSG000002
PLPP7	Phospholipid Phosphatase 7 (Inactive)	Protein Coding	28	GC09P131290	2.3	https://www.genecards.org/cgi-bin/carddisp.pl?gene=PLPP7
A2MP1	Alpha-2-Macroglobulin Pseudogene 1	Pseudogene	12	GC12M009228	2.29	https://www.genecards.org/cgi-bin/carddisp.pl?gene=A2MP1
EIF3L	Eukaryotic Translation Initiation Factor 3 Subunit L	Protein Coding	37	GC22P037848	2.29	https://www.genecards.org/cgi-bin/carddisp.pl?gene=EIF3L
RNU6-961P	RNA, U6 Small Nuclear 961, Pseudogene	Pseudogene	8	GC02P020175	2.28	https://www.genecards.org/cgi-bin/carddisp.pl?gene=RNU6-961P

ARHGAP8	Rho GTPase Activating Protein 8	Protein Coding	34	GC22P044752	2.28	https://www.genecards.org/cgi-bin/carddisp.pl?gene=ARHGAP8
IL36B	Interleukin 36 Beta	Protein Coding	35	GC02M113022	2.28	https://www.genecards.org/cgi-bin/carddisp.pl?gene=IL36B
HNRNPF	Heterogeneous Nuclear Ribonucleoprotein F	Protein Coding	40	GC10M043385	2.28	https://www.genecards.org/cgi-bin/carddisp.pl?gene=HNRNPF
SRSF1	Serine And Arginine Rich Splicing Factor 1	Protein Coding	41	GC17M058000	2.28	https://www.genecards.org/cgi-bin/carddisp.pl?gene=SRSF1
LOC389199	Uncharacterized LOC389199	Protein Coding	17	GC04P007939	2.27	https://www.genecards.org/cgi-bin/carddisp.pl?gene=LOC389199
RAB37	RAB37, Member RAS Oncogene Family	Protein Coding	37	GC17P074671	2.27	https://www.genecards.org/cgi-bin/carddisp.pl?gene=RAB37
TRMT44	TRNA Methyltransferase 44 Homolog	Protein Coding	32	GC04P008439	2.26	https://www.genecards.org/cgi-bin/carddisp.pl?gene=TRMT44
RPS9	Ribosomal Protein S9	Protein Coding	41	GC19P055552	2.26	https://www.genecards.org/cgi-bin/carddisp.pl?gene=RPS9
ENSG00000255046		RNA Gene	8	GC08M011885	2.26	https://www.genecards.org/cgi-bin/carddisp.pl?gene=ENSG000002
ENSG00000287292		RNA Gene	6	GC04P148445	2.26	https://www.genecards.org/cgi-bin/carddisp.pl?gene=ENSG000002
ENSG00000222042		RNA Gene	6	GC21M025169	2.26	https://www.genecards.org/cgi-bin/carddisp.pl?gene=ENSG000002
lnc-LTBP3-4		RNA Gene	5	GC11M065572	2.26	https://www.genecards.org/cgi-bin/carddisp.pl?gene=lnc-LTBP3-4
OTX2P1	OTX2 Pseudogene 1	Pseudogene	5	GC09M075724	2.26	https://www.genecards.org/cgi-bin/carddisp.pl?gene=OTX2P1
ENSG00000237936		Pseudogene	4	GC10M029891	2.26	https://www.genecards.org/cgi-bin/carddisp.pl?gene=ENSG000002
MN309375		RNA Gene	2	GC11M065581	2.26	https://www.genecards.org/cgi-bin/carddisp.pl?gene=MN309375
BECN2	Beclin 2	Protein Coding	22	GC01P241957	2.25	https://www.genecards.org/cgi-bin/carddisp.pl?gene=BECN2
INSIG2	Insulin Induced Gene 2	Protein Coding	40	GC02P118088	2.25	https://www.genecards.org/cgi-bin/carddisp.pl?gene=INSIG2
RPL8	Ribosomal Protein L8	Protein Coding	41	GC08M144790	2.25	https://www.genecards.org/cgi-bin/carddisp.pl?gene=RPL8
GSTK1	Glutathione S-Transferase Kappa 1	Protein Coding	40	GC07P144971	2.24	https://www.genecards.org/cgi-bin/carddisp.pl?gene=GSTK1
RPS18	Ribosomal Protein S18	Protein Coding	40	GC06P047372	2.24	https://www.genecards.org/cgi-bin/carddisp.pl?gene=RPS18
TLL1	Tolloid Like 1	Protein Coding	43	GC04P165873	2.22	https://www.genecards.org/cgi-bin/carddisp.pl?gene=TLL1
ENSG00000234389		RNA Gene	8	GC02P102438	2.22	https://www.genecards.org/cgi-bin/carddisp.pl?gene=ENSG000002
ENSG00000249631		RNA Gene	7	GC04P013496	2.22	https://www.genecards.org/cgi-bin/carddisp.pl?gene=ENSG000002
lnc-FLI1-5		RNA Gene	4	GC11P128414	2.22	https://www.genecards.org/cgi-bin/carddisp.pl?gene=lnc-FLI1-5
ZNF267	Zinc Finger Protein 267	Protein Coding	36	GC16P031885	2.22	https://www.genecards.org/cgi-bin/carddisp.pl?gene=ZNF267
G0S2	G0/G1 Switch 2	Protein Coding	32	GC01P209675	2.22	https://www.genecards.org/cgi-bin/carddisp.pl?gene=G0S2
MIR1183	MicroRNA 1183	RNA Gene	14	GC07P021510	2.21	https://www.genecards.org/cgi-bin/carddisp.pl?gene=MIR1183
HNRNPH1	Heterogeneous Nuclear Ribonucleoprotein H1	Protein Coding	38	GC05M179614	2.2	https://www.genecards.org/cgi-bin/carddisp.pl?gene=HNRNPH1
MIR449A	MicroRNA 449a	RNA Gene	20	GC05M055171	2.2	https://www.genecards.org/cgi-bin/carddisp.pl?gene=MIR449A
C1QTNF1	C1q And TNF Related 1	Protein Coding	39	GC17P079022	2.19	https://www.genecards.org/cgi-bin/carddisp.pl?gene=C1QTNF1
LRRFIP2	LRR Binding FLII Interacting Protein 2	Protein Coding	38	GC03M037052	2.19	https://www.genecards.org/cgi-bin/carddisp.pl?gene=LRRFIP2
ANXA9	Annexin A9	Protein Coding	39	GC01P150982	2.19	https://www.genecards.org/cgi-bin/carddisp.pl?gene=ANXA9
CRTC1	CREB Regulated Transcription Coactivator 1	Protein Coding	42	GC19P023333	2.18	https://www.genecards.org/cgi-bin/carddisp.pl?gene=CRTC1
SVIL-AS1	SVIL Antisense RNA 1	RNA Gene	12	GC10P029410	2.18	https://www.genecards.org/cgi-bin/carddisp.pl?gene=SVIL-AS1
SNRPD2	Small Nuclear Ribonucleoprotein D2 Polypeptide	Protein Coding	40	GC19M045688	2.18	https://www.genecards.org/cgi-bin/carddisp.pl?gene=SNRPD2
SLC9A9-AS1	SLC9A9 Antisense RNA 1	RNA Gene	13	GC03P143342	2.18	https://www.genecards.org/cgi-bin/carddisp.pl?gene=SLC9A9-AS1
TEX36-AS1	TEX36 Antisense RNA 1	RNA Gene	10	GC10P125574	2.18	https://www.genecards.org/cgi-bin/carddisp.pl?gene=TEX36-AS1
LOC100130331	POTE Ankyrin Domain Family, Member F Pseudogen	Pseudogene	9	GC01P237862	2.18	https://www.genecards.org/cgi-bin/carddisp.pl?gene=LOC100130331
SLC9A9-AS2	SLC9A9 Antisense RNA 2	RNA Gene	8	GC03P143381	2.18	https://www.genecards.org/cgi-bin/carddisp.pl?gene=SLC9A9-AS2

ENSG00000257322		RNA Gene	8	GC12M093003	2.18	https://www.genecards.org/cgi-bin/carddisp.pl?gene=ENSG000002
RNU6-908P	RNA, U6 Small Nuclear 908, Pseudogene	Pseudogene	7	GC10P029711	2.18	https://www.genecards.org/cgi-bin/carddisp.pl?gene=RNU6-908P
ENSG00000236501		RNA Gene	7	GC02M176990	2.18	https://www.genecards.org/cgi-bin/carddisp.pl?gene=ENSG000002
ENSG00000250541		RNA Gene	6	GC04P025771	2.18	https://www.genecards.org/cgi-bin/carddisp.pl?gene=ENSG000002
ENSG00000265994		RNA Gene	6	GC18P028638	2.18	https://www.genecards.org/cgi-bin/carddisp.pl?gene=ENSG000002
ENSG00000207171		RNA Gene	5	GC04P168471	2.18	https://www.genecards.org/cgi-bin/carddisp.pl?gene=ENSG000002
ENSG00000235464		RNA Gene	5	GC07M035496	2.18	https://www.genecards.org/cgi-bin/carddisp.pl?gene=ENSG000002
RNU1-35P	RNA, U1 Small Nuclear 35, Pseudogene	Pseudogene	5	GC08M135742	2.18	https://www.genecards.org/cgi-bin/carddisp.pl?gene=RNU1-35P
EEF1A1P20	Eukaryotic Translation Elongation Factor 1 Alpha 1 Ps	Pseudogene	5	GC05P099996	2.18	https://www.genecards.org/cgi-bin/carddisp.pl?gene=EEF1A1P20
ENSG00000236389		RNA Gene	5	GC06M134706	2.18	https://www.genecards.org/cgi-bin/carddisp.pl?gene=ENSG000002
ENSG00000287032		RNA Gene	4	GC07M035614	2.18	https://www.genecards.org/cgi-bin/carddisp.pl?gene=ENSG000002
ENSG00000284676		RNA Gene	4	GC01P029755	2.18	https://www.genecards.org/cgi-bin/carddisp.pl?gene=ENSG000002
lnc-DDX60L-3		RNA Gene	4	GC04M168468	2.18	https://www.genecards.org/cgi-bin/carddisp.pl?gene=lnc-DDX60L-
ENSG00000226973		Pseudogene	4	GC01P115471	2.18	https://www.genecards.org/cgi-bin/carddisp.pl?gene=ENSG000002
ENSG00000199378		RNA Gene	4	GC05P100021	2.18	https://www.genecards.org/cgi-bin/carddisp.pl?gene=ENSG000001
lnc-KCNK7-1		RNA Gene	4	GC11M065582	2.18	https://www.genecards.org/cgi-bin/carddisp.pl?gene=lnc-KCNK7-1
HSALNG0076986		RNA Gene	4	GC10M030503	2.18	https://www.genecards.org/cgi-bin/carddisp.pl?gene=HSALNG007
ENSG00000276627		RNA Gene	4	GC20P060761	2.18	https://www.genecards.org/cgi-bin/carddisp.pl?gene=ENSG000002
piR-41195-080		RNA Gene	3	GC08M011894	2.18	https://www.genecards.org/cgi-bin/carddisp.pl?gene=piR-41195-08
lnc-UBE2N-9		RNA Gene	3	GC12M093168	2.18	https://www.genecards.org/cgi-bin/carddisp.pl?gene=lnc-UBE2N-9
lnc-EDRF1-4		RNA Gene	3	GC10P125590	2.18	https://www.genecards.org/cgi-bin/carddisp.pl?gene=lnc-EDRF1-4
piR-59297-626		RNA Gene	3	GC08P011819	2.18	https://www.genecards.org/cgi-bin/carddisp.pl?gene=piR-59297-62
LOC105372698	Uncharacterized LOC105372698	Protein Coding	3	GC20P060257	2.18	https://www.genecards.org/cgi-bin/carddisp.pl?gene=LOC1053726
piR-38051-198		RNA Gene	3	GC04M025834	2.18	https://www.genecards.org/cgi-bin/carddisp.pl?gene=piR-38051-19
HSALNG0076987		RNA Gene	3	GC10P029810	2.18	https://www.genecards.org/cgi-bin/carddisp.pl?gene=HSALNG007
LOC107986198	Uncharacterized LOC107986198	RNA Gene	3	GC04M168488	2.18	https://www.genecards.org/cgi-bin/carddisp.pl?gene=LOC1079861
LOC102723733	Uncharacterized LOC102723733	RNA Gene	3	GC04P025862	2.18	https://www.genecards.org/cgi-bin/carddisp.pl?gene=LOC1027237
ENSG00000270927		Pseudogene	3	GC01M029904	2.18	https://www.genecards.org/cgi-bin/carddisp.pl?gene=ENSG000002
piR-57133-376		RNA Gene	2	GC20P060683	2.18	https://www.genecards.org/cgi-bin/carddisp.pl?gene=piR-57133-37
AB372775		RNA Gene	2	GC10P069827	2.18	https://www.genecards.org/cgi-bin/carddisp.pl?gene=AB372775
RF00994-591		RNA Gene	2	GC20P060710	2.18	https://www.genecards.org/cgi-bin/carddisp.pl?gene=RF00994-591
piR-31109-001		RNA Gene	2	GC10P125633	2.18	https://www.genecards.org/cgi-bin/carddisp.pl?gene=piR-31109-00
RF00017-1202		RNA Gene	2	GC12P093145	2.18	https://www.genecards.org/cgi-bin/carddisp.pl?gene=RF00017-120
lnc-AIFM2-3		RNA Gene	2	GC10M069839	2.18	https://www.genecards.org/cgi-bin/carddisp.pl?gene=lnc-AIFM2-3
ENSG00000283174		Pseudogene	1	GC01M237940	2.18	https://www.genecards.org/cgi-bin/carddisp.pl?gene=ENSG000002
ENSG00000213117		Pseudogene	1	GC06P134898	2.18	https://www.genecards.org/cgi-bin/carddisp.pl?gene=ENSG000002
ENSG00000254939		Pseudogene	1	GC11M098568	2.18	https://www.genecards.org/cgi-bin/carddisp.pl?gene=ENSG000002
RPL24	Ribosomal Protein L24	Protein Coding	39	GC03M101681	2.17	https://www.genecards.org/cgi-bin/carddisp.pl?gene=RPL24
RNU6-976P	RNA, U6 Small Nuclear 976, Pseudogene	Pseudogene	6	GC14P084739	2.17	https://www.genecards.org/cgi-bin/carddisp.pl?gene=RNU6-976P

ENSG00000283991		RNA Gene	4	GC07M004130	2.17	https://www.genecards.org/cgi-bin/carddisp.pl?gene=ENSG0000002
lnc-TRIB1-7		RNA Gene	4	GC08P125495	2.17	https://www.genecards.org/cgi-bin/carddisp.pl?gene=lnc-TRIB1-7
LOC105375131	Uncharacterized LOC105375131	RNA Gene	4	GC07M004126	2.17	https://www.genecards.org/cgi-bin/carddisp.pl?gene=LOC1053751
ENSG00000268473		Pseudogene	2	GC16M086434	2.17	https://www.genecards.org/cgi-bin/carddisp.pl?gene=ENSG0000002
STMP1	Short Transmembrane Mitochondrial Protein 1	Protein Coding	22	GC07P135663	2.17	https://www.genecards.org/cgi-bin/carddisp.pl?gene=STMP1
DHX9	DExH-Box Helicase 9	Protein Coding	40	GC01P182839	2.16	https://www.genecards.org/cgi-bin/carddisp.pl?gene=DHX9
PSME2	Proteasome Activator Subunit 2	Protein Coding	43	GC14M024143	2.16	https://www.genecards.org/cgi-bin/carddisp.pl?gene=PSME2
RBFOX1	RNA Binding Fox-1 Homolog 1	Protein Coding	37	GC16P005240	2.16	https://www.genecards.org/cgi-bin/carddisp.pl?gene=RBFOX1
THNSL2	Threonine Synthase Like 2	Protein Coding	33	GC02P088170	2.15	https://www.genecards.org/cgi-bin/carddisp.pl?gene=THNSL2
LINC00355	Long Intergenic Non-Protein Coding RNA 355	RNA Gene	14	GC13M063838	2.14	https://www.genecards.org/cgi-bin/carddisp.pl?gene=LINC00355
AP2A2	Adaptor Related Protein Complex 2 Subunit Alpha 2	Protein Coding	37	GC11P000915	2.14	https://www.genecards.org/cgi-bin/carddisp.pl?gene=AP2A2
TUBA8	Tubulin Alpha 8	Protein Coding	45	GC22P018110	2.13	https://www.genecards.org/cgi-bin/carddisp.pl?gene=TUBA8
LOC101929710	Uncharacterized LOC101929710	RNA Gene	10	GC05P095962	2.12	https://www.genecards.org/cgi-bin/carddisp.pl?gene=LOC1019297
INPP4B	Inositol Polyphosphate-4-Phosphatase Type II B	Protein Coding	39	GC04M142023	2.12	https://www.genecards.org/cgi-bin/carddisp.pl?gene=INPP4B
PABPC1	Poly(A) Binding Protein Cytoplasmic 1	Protein Coding	41	GC08M100685	2.12	https://www.genecards.org/cgi-bin/carddisp.pl?gene=PABPC1
POT1-AS1	POT1 Antisense RNA 1	RNA Gene	16	GC07P124929	2.12	https://www.genecards.org/cgi-bin/carddisp.pl?gene=POT1-AS1
ST13P12	ST13, Hsp70 Interacting Protein Pseudogene 12	Pseudogene	6	GC05P082968	2.12	https://www.genecards.org/cgi-bin/carddisp.pl?gene=ST13P12
NLRP2	NLR Family Pyrin Domain Containing 2	Protein Coding	41	GC19P054953	2.11	https://www.genecards.org/cgi-bin/carddisp.pl?gene=NLRP2
LINC02789	Long Intergenic Non-Protein Coding RNA 2789	RNA Gene	8	GC01P199149	2.11	https://www.genecards.org/cgi-bin/carddisp.pl?gene=LINC02789
LINC01090	Long Intergenic Non-Protein Coding RNA 1090	RNA Gene	13	GC02M187712	2.11	https://www.genecards.org/cgi-bin/carddisp.pl?gene=LINC01090
PSAPL1	Prosaposin Like 1	Protein Coding	32	GC04M007434	2.11	https://www.genecards.org/cgi-bin/carddisp.pl?gene=PSAPL1
ENSG00000237596		Uncategorized	7	GC06M135991	2.11	https://www.genecards.org/cgi-bin/carddisp.pl?gene=ENSG0000002
ENSG00000250001		RNA Gene	7	GC05M009519	2.11	https://www.genecards.org/cgi-bin/carddisp.pl?gene=ENSG0000002
LOC105373944	Translation Initiation Factor IF-2-Like	Protein Coding	6	GC02M236161	2.11	https://www.genecards.org/cgi-bin/carddisp.pl?gene=LOC1053739
ENSG00000248537		RNA Gene	6	GC05P009363	2.11	https://www.genecards.org/cgi-bin/carddisp.pl?gene=ENSG0000002
HNRNPA1P58	Heterogeneous Nuclear Ribonucleoprotein A1 Pseudog	Pseudogene	4	GC06P024003	2.11	https://www.genecards.org/cgi-bin/carddisp.pl?gene=HNRNPA1P5
LOC101060035	DnaJ Homolog Subfamily C Member 24 Pseudogene	Pseudogene	4	GC05M133720	2.11	https://www.genecards.org/cgi-bin/carddisp.pl?gene=LOC1010600
SLC23A2	Solute Carrier Family 23 Member 2	Protein Coding	42	GC20M004852	2.1	https://www.genecards.org/cgi-bin/carddisp.pl?gene=SLC23A2
ACTR2	Actin Related Protein 2	Protein Coding	43	GC02P065227	2.09	https://www.genecards.org/cgi-bin/carddisp.pl?gene=ACTR2
FECHP1	Ferrochelatase Pseudogene 1	Pseudogene	6	GC03P034873	2.09	https://www.genecards.org/cgi-bin/carddisp.pl?gene=FECHP1
EIF4A3	Eukaryotic Translation Initiation Factor 4A3	Protein Coding	43	GC17M080135	2.08	https://www.genecards.org/cgi-bin/carddisp.pl?gene=EIF4A3
LOC101928135	Uncharacterized LOC101928135	RNA Gene	6	GC03M034875	2.08	https://www.genecards.org/cgi-bin/carddisp.pl?gene=LOC1019281
KRT8P18	Keratin 8 Pseudogene 18	Pseudogene	5	GC03M035233	2.08	https://www.genecards.org/cgi-bin/carddisp.pl?gene=KRT8P18
ACTR3	Actin Related Protein 3	Protein Coding	42	GC02P113889	2.08	https://www.genecards.org/cgi-bin/carddisp.pl?gene=ACTR3
PRR5-ARHGAP8	PRR5-ARHGAP8 Readthrough	Protein Coding	27	GC22P044702	2.08	https://www.genecards.org/cgi-bin/carddisp.pl?gene=PRR5-ARHG
PHF21B	PHD Finger Protein 21B	Protein Coding	35	GC22M044881	2.08	https://www.genecards.org/cgi-bin/carddisp.pl?gene=PHF21B
ENSG00000274121		RNA Gene	5	GC01P231814	2.07	https://www.genecards.org/cgi-bin/carddisp.pl?gene=ENSG0000002
ENSG00000286071		RNA Gene	4	GC01M231767	2.07	https://www.genecards.org/cgi-bin/carddisp.pl?gene=ENSG0000002
ATP6AP1L	ATPase H+ Transporting Accessory Protein 1 Like	Protein Coding	26	GC05P082279	2.07	https://www.genecards.org/cgi-bin/carddisp.pl?gene=ATP6AP1L

SLC23A1	Solute Carrier Family 23 Member 1	Protein Coding	43	GC05M139377	2.07	https://www.genecards.org/cgi-bin/carddisp.pl?gene=SLC23A1
SRSF7	Serine And Arginine Rich Splicing Factor 7	Protein Coding	39	GC02M038709	2.07	https://www.genecards.org/cgi-bin/carddisp.pl?gene=SRSF7
STARP1	Steroidogenic Acute Regulatory Protein Pseudogene 1	Pseudogene	7	GC13M065280	2.06	https://www.genecards.org/cgi-bin/carddisp.pl?gene=STARP1
LINC00888	Long Intergenic Non-Protein Coding RNA 888	RNA Gene	13	GC03P183447	2.05	https://www.genecards.org/cgi-bin/carddisp.pl?gene=LINC00888
PBX2P1	PBX Homeobox 2 Pseudogene 1	Pseudogene	11	GC03P143176	2.05	https://www.genecards.org/cgi-bin/carddisp.pl?gene=PBX2P1
SERPINB4	Serpin Family B Member 4	Protein Coding	38	GC18M063637	2.05	https://www.genecards.org/cgi-bin/carddisp.pl?gene=SERPINB4
HNRNPA3	Heterogeneous Nuclear Ribonucleoprotein A3	Protein Coding	37	GC02P177212	2.04	https://www.genecards.org/cgi-bin/carddisp.pl?gene=HNRNPA3
USP4	Ubiquitin Specific Peptidase 4	Protein Coding	43	GC03M049277	2.04	https://www.genecards.org/cgi-bin/carddisp.pl?gene=USP4
TET1	Tet Methylcytosine Dioxygenase 1	Protein Coding	38	GC10P068560	2.04	https://www.genecards.org/cgi-bin/carddisp.pl?gene=TET1
TPI1P1	Triosephosphate Isomerase 1 Pseudogene 1	Pseudogene	7	GC01P076699	2.04	https://www.genecards.org/cgi-bin/carddisp.pl?gene=TPI1P1
LOC401864	Chloride Intracellular Channel 1 Pseudogene	Pseudogene	5	GC16M086468	2.03	https://www.genecards.org/cgi-bin/carddisp.pl?gene=LOC401864
SLC23A4P	Solute Carrier Family 23 Member 4, Pseudogene	Pseudogene	5	GC07M135270	2.03	https://www.genecards.org/cgi-bin/carddisp.pl?gene=SLC23A4P
MIR378C	MicroRNA 378c	RNA Gene	13	GC10M130962	2.03	https://www.genecards.org/cgi-bin/carddisp.pl?gene=MIR378C
MIR4772	MicroRNA 4772	RNA Gene	13	GC02P102432	2.03	https://www.genecards.org/cgi-bin/carddisp.pl?gene=MIR4772
AP2A1	Adaptor Related Protein Complex 2 Subunit Alpha 1	Protein Coding	38	GC19P049766	2.03	https://www.genecards.org/cgi-bin/carddisp.pl?gene=AP2A1
CPB2-AS1	CPB2 Antisense RNA 1	RNA Gene	14	GC13P046052	2.03	https://www.genecards.org/cgi-bin/carddisp.pl?gene=CPB2-AS1
HDHD5-AS1	HDHD5 Antisense RNA 1	RNA Gene	12	GC22P017160	2.03	https://www.genecards.org/cgi-bin/carddisp.pl?gene=HDHD5-AS1
LINC01029	Long Intergenic Non-Protein Coding RNA 1029	RNA Gene	12	GC18M077972	2.03	https://www.genecards.org/cgi-bin/carddisp.pl?gene=LINC01029
SNORA63E	Small Nucleolar RNA, H/ACA Box 63E	RNA Gene	9	GC03P183457	2.03	https://www.genecards.org/cgi-bin/carddisp.pl?gene=SNORA63E
LINC02392	Long Intergenic Non-Protein Coding RNA 2392	RNA Gene	9	GC12P090282	2.03	https://www.genecards.org/cgi-bin/carddisp.pl?gene=LINC02392
LINC02534	Long Intergenic Non-Protein Coding RNA 2534	RNA Gene	9	GC06M115635	2.03	https://www.genecards.org/cgi-bin/carddisp.pl?gene=LINC02534
ENSG00000243273		RNA Gene	8	GC03P150890	2.03	https://www.genecards.org/cgi-bin/carddisp.pl?gene=ENSG000002
SNORA63D	Small Nucleolar RNA, H/ACA Box 63D	RNA Gene	8	GC03P183467	2.03	https://www.genecards.org/cgi-bin/carddisp.pl?gene=SNORA63D
LINC01818	Long Intergenic Non-Protein Coding RNA 1818	RNA Gene	8	GC02P150170	2.03	https://www.genecards.org/cgi-bin/carddisp.pl?gene=LINC01818
ENSG00000260234		Protein Coding	8	GC03M150882	2.03	https://www.genecards.org/cgi-bin/carddisp.pl?gene=ENSG000002
ENSG00000259053		RNA Gene	7	GC14M094051	2.03	https://www.genecards.org/cgi-bin/carddisp.pl?gene=ENSG000002
ENSG00000251152		RNA Gene	7	GC04P013479	2.03	https://www.genecards.org/cgi-bin/carddisp.pl?gene=ENSG000002
RNU6-655P	RNA, U6 Small Nuclear 655, Pseudogene	Pseudogene	6	GC18P078036	2.03	https://www.genecards.org/cgi-bin/carddisp.pl?gene=RNU6-655P
RNU6-161P	RNA, U6 Small Nuclear 161, Pseudogene	Pseudogene	6	GC01P076753	2.03	https://www.genecards.org/cgi-bin/carddisp.pl?gene=RNU6-161P
ENSG00000241679		RNA Gene	6	GC03P143123	2.03	https://www.genecards.org/cgi-bin/carddisp.pl?gene=ENSG000002
RPL35AP33	Ribosomal Protein L35a Pseudogene 33	Pseudogene	6	GC16M068478	2.03	https://www.genecards.org/cgi-bin/carddisp.pl?gene=RPL35AP33
ENSG00000232739		RNA Gene	6	GC10P015237	2.03	https://www.genecards.org/cgi-bin/carddisp.pl?gene=ENSG000002
ENSG00000234481		RNA Gene	6	GC01P036773	2.03	https://www.genecards.org/cgi-bin/carddisp.pl?gene=ENSG000002
ENSG00000252040		RNA Gene	6	GC16M068630	2.03	https://www.genecards.org/cgi-bin/carddisp.pl?gene=ENSG000002
ENSG00000253092		RNA Gene	6	GC03P183452	2.03	https://www.genecards.org/cgi-bin/carddisp.pl?gene=ENSG000002
ENSG00000249664		RNA Gene	6	GC05M083012	2.03	https://www.genecards.org/cgi-bin/carddisp.pl?gene=ENSG000002
LGMNP1	Legumain Pseudogene 1	Pseudogene	6	GC13M064957	2.03	https://www.genecards.org/cgi-bin/carddisp.pl?gene=LGMNP1
ENSG00000253354		RNA Gene	6	GC08P040370	2.03	https://www.genecards.org/cgi-bin/carddisp.pl?gene=ENSG000002
ENSG00000272479		RNA Gene	6	GC08M040519	2.03	https://www.genecards.org/cgi-bin/carddisp.pl?gene=ENSG000002

LINC02822	Long Intergenic Non-Protein Coding RNA 2822	RNA Gene	6	GC12P090577	2.03	https://www.genecards.org/cgi-bin/carddisp.pl?gene=LINC02822
HNRNPA3P5	Heterogeneous Nuclear Ribonucleoprotein A3 Pseudog	Pseudogene	5	GC13M065787	2.03	https://www.genecards.org/cgi-bin/carddisp.pl?gene=HNRNPA3P5
RNU6-1172P	RNA, U6 Small Nuclear 1172, Pseudogene	Pseudogene	5	GC08M096431	2.03	https://www.genecards.org/cgi-bin/carddisp.pl?gene=RNU6-1172P
MRPL45P1	Mitochondrial Ribosomal Protein L45 Pseudogene 1	Pseudogene	5	GC02M088402	2.03	https://www.genecards.org/cgi-bin/carddisp.pl?gene=MRPL45P1
ENSG00000197462		RNA Gene	5	GC07P125917	2.03	https://www.genecards.org/cgi-bin/carddisp.pl?gene=ENSG000001
ENSG00000235503		RNA Gene	5	GC07P083355	2.03	https://www.genecards.org/cgi-bin/carddisp.pl?gene=ENSG000002
ENSG00000234034		RNA Gene	5	GC21M022500	2.03	https://www.genecards.org/cgi-bin/carddisp.pl?gene=ENSG000002
LOC107987158	Uncharacterized LOC107987158	Protein Coding	5	GC11M003142	2.03	https://www.genecards.org/cgi-bin/carddisp.pl?gene=LOC1079871
ENSG00000251730		RNA Gene	5	GC03P183454	2.03	https://www.genecards.org/cgi-bin/carddisp.pl?gene=ENSG000002
ENSG00000254171		RNA Gene	5	GC05P166128	2.03	https://www.genecards.org/cgi-bin/carddisp.pl?gene=ENSG000002
RPL23AP20	Ribosomal Protein L23a Pseudogene 20	Pseudogene	5	GC01P241916	2.03	https://www.genecards.org/cgi-bin/carddisp.pl?gene=RPL23AP20
ENSG00000259080		RNA Gene	5	GC02P027583	2.03	https://www.genecards.org/cgi-bin/carddisp.pl?gene=ENSG000002
lnc-SLC46A3-1		RNA Gene	4	GC13M028701	2.03	https://www.genecards.org/cgi-bin/carddisp.pl?gene=lnc-SLC46A3
ENSG00000288092		RNA Gene	4	GC12P103013	2.03	https://www.genecards.org/cgi-bin/carddisp.pl?gene=ENSG000002
lnc-TSNAX-2		RNA Gene	4	GC01P231693	2.03	https://www.genecards.org/cgi-bin/carddisp.pl?gene=lnc-TSNAX-2
SDR42E1P5	Short Chain Dehydrogenase/Reductase Family 42E, M	Pseudogene	4	GC02P102412	2.03	https://www.genecards.org/cgi-bin/carddisp.pl?gene=SDR42E1P5
lnc-MRGPRG-1		RNA Gene	4	GC11M003146	2.03	https://www.genecards.org/cgi-bin/carddisp.pl?gene=lnc-MRGPRG
ENSG00000233983		Pseudogene	4	GC01P099464	2.03	https://www.genecards.org/cgi-bin/carddisp.pl?gene=ENSG000002
MN309223		RNA Gene	4	GC11P003129	2.03	https://www.genecards.org/cgi-bin/carddisp.pl?gene=MN309223
ENSG00000271682		Pseudogene	4	GC03M183462	2.03	https://www.genecards.org/cgi-bin/carddisp.pl?gene=ENSG000002
ENSG00000287720		RNA Gene	3	GC03P003918	2.03	https://www.genecards.org/cgi-bin/carddisp.pl?gene=ENSG000002
lnc-SLC22A18-7		RNA Gene	3	GC11P003146	2.03	https://www.genecards.org/cgi-bin/carddisp.pl?gene=lnc-SLC22A1
lnc-RASGRP1-1		RNA Gene	3	GC15M038754	2.03	https://www.genecards.org/cgi-bin/carddisp.pl?gene=lnc-RASGRP
lnc-WDR91-4		RNA Gene	3	GC07M135225	2.03	https://www.genecards.org/cgi-bin/carddisp.pl?gene=lnc-WDR91-4
piR-50443-100		RNA Gene	3	GC13P046157	2.03	https://www.genecards.org/cgi-bin/carddisp.pl?gene=piR-50443-10
ENSG00000229119		Pseudogene	3	GC05P166383	2.03	https://www.genecards.org/cgi-bin/carddisp.pl?gene=ENSG000002
ENSG00000225906		Pseudogene	3	GC21P022882	2.03	https://www.genecards.org/cgi-bin/carddisp.pl?gene=ENSG000002
HSALNG0113292		RNA Gene	3	GC16P086387	2.03	https://www.genecards.org/cgi-bin/carddisp.pl?gene=HSALNG011
piR-61101-044		RNA Gene	3	GC10M015274	2.03	https://www.genecards.org/cgi-bin/carddisp.pl?gene=piR-61101-04
ENSG00000231984		Pseudogene	3	GC01P199753	2.03	https://www.genecards.org/cgi-bin/carddisp.pl?gene=ENSG000002
piR-61861-018		RNA Gene	3	GC22P044789	2.03	https://www.genecards.org/cgi-bin/carddisp.pl?gene=piR-61861-01
hsa-miR-5096-076		RNA Gene	3	GC07M004224	2.03	https://www.genecards.org/cgi-bin/carddisp.pl?gene=hsa-miR-5096
ENSG00000275383		RNA Gene	3	GC16P068591	2.03	https://www.genecards.org/cgi-bin/carddisp.pl?gene=ENSG000002
piR-48470		RNA Gene	2	GC06M043135	2.03	https://www.genecards.org/cgi-bin/carddisp.pl?gene=piR-48470
RF00026-283		RNA Gene	2	GC13M028704	2.03	https://www.genecards.org/cgi-bin/carddisp.pl?gene=RF00026-283
piR-40638		RNA Gene	2	GC06M042634	2.03	https://www.genecards.org/cgi-bin/carddisp.pl?gene=piR-40638
piR-40663		RNA Gene	2	GC06M042635	2.03	https://www.genecards.org/cgi-bin/carddisp.pl?gene=piR-40663
piR-49272		RNA Gene	2	GC06M043149	2.03	https://www.genecards.org/cgi-bin/carddisp.pl?gene=piR-49272
piR-48794		RNA Gene	2	GC06M043141	2.03	https://www.genecards.org/cgi-bin/carddisp.pl?gene=piR-48794

piR-48799-012		RNA Gene	2	GC10P015269	2.03	https://www.genecards.org/cgi-bin/carddisp.pl?gene=piR-48799-01
piR-49129		RNA Gene	2	GC06M043147	2.03	https://www.genecards.org/cgi-bin/carddisp.pl?gene=piR-49129
piR-39926		RNA Gene	2	GC06M042624	2.03	https://www.genecards.org/cgi-bin/carddisp.pl?gene=piR-39926
piR-39858-368		RNA Gene	2	GC20M016549	2.03	https://www.genecards.org/cgi-bin/carddisp.pl?gene=piR-39858-36
piR-56788		RNA Gene	2	GC06M043295	2.03	https://www.genecards.org/cgi-bin/carddisp.pl?gene=piR-56788
piR-56842		RNA Gene	2	GC06M043296	2.03	https://www.genecards.org/cgi-bin/carddisp.pl?gene=piR-56842
piR-32810-118		RNA Gene	2	GC07M021535	2.03	https://www.genecards.org/cgi-bin/carddisp.pl?gene=piR-32810-11
piR-33024		RNA Gene	2	GC06M042499	2.03	https://www.genecards.org/cgi-bin/carddisp.pl?gene=piR-33024
piR-32825		RNA Gene	2	GC06M042490	2.03	https://www.genecards.org/cgi-bin/carddisp.pl?gene=piR-32825
piR-32961		RNA Gene	2	GC06M042497	2.03	https://www.genecards.org/cgi-bin/carddisp.pl?gene=piR-32961
ENSG00000285529		RNA Gene	2	GC10P130525	2.03	https://www.genecards.org/cgi-bin/carddisp.pl?gene=ENSG000002
piR-48131		RNA Gene	2	GC06M043131	2.03	https://www.genecards.org/cgi-bin/carddisp.pl?gene=piR-48131
piR-39137		RNA Gene	2	GC06M042608	2.03	https://www.genecards.org/cgi-bin/carddisp.pl?gene=piR-39137
piR-32153		RNA Gene	2	GC06M042468	2.03	https://www.genecards.org/cgi-bin/carddisp.pl?gene=piR-32153
piR-49710		RNA Gene	2	GC06M043154	2.03	https://www.genecards.org/cgi-bin/carddisp.pl?gene=piR-49710
piR-59496		RNA Gene	2	GC06M043336	2.03	https://www.genecards.org/cgi-bin/carddisp.pl?gene=piR-59496
piR-43106-189		RNA Gene	2	GC07P135282	2.03	https://www.genecards.org/cgi-bin/carddisp.pl?gene=piR-43106-18
piR-34682		RNA Gene	2	GC06M042530	2.03	https://www.genecards.org/cgi-bin/carddisp.pl?gene=piR-34682
piR-42506		RNA Gene	2	GC06M042681	2.03	https://www.genecards.org/cgi-bin/carddisp.pl?gene=piR-42506
piR-42270		RNA Gene	2	GC06M042676	2.03	https://www.genecards.org/cgi-bin/carddisp.pl?gene=piR-42270
lnc-TRIB1-8		RNA Gene	2	GC08P125497	2.03	https://www.genecards.org/cgi-bin/carddisp.pl?gene=lnc-TRIB1-8
HSALNG0031159		RNA Gene	2	GC03P183480	2.03	https://www.genecards.org/cgi-bin/carddisp.pl?gene=HSALNG003
HSALNG0032693		RNA Gene	2	GC04M007714	2.03	https://www.genecards.org/cgi-bin/carddisp.pl?gene=HSALNG003
piR-43926-003		RNA Gene	2	GC06M043041	2.03	https://www.genecards.org/cgi-bin/carddisp.pl?gene=piR-43926-00
piR-44231		RNA Gene	2	GC06M043046	2.03	https://www.genecards.org/cgi-bin/carddisp.pl?gene=piR-44231
piR-59890		RNA Gene	2	GC06M043338	2.03	https://www.genecards.org/cgi-bin/carddisp.pl?gene=piR-59890
piR-35359		RNA Gene	2	GC06M042546	2.03	https://www.genecards.org/cgi-bin/carddisp.pl?gene=piR-35359
piR-36491		RNA Gene	2	GC06M042566	2.03	https://www.genecards.org/cgi-bin/carddisp.pl?gene=piR-36491
lnc-XRCC4-4		RNA Gene	2	GC05P083016	2.03	https://www.genecards.org/cgi-bin/carddisp.pl?gene=lnc-XRCC4-4
piR-54702		RNA Gene	2	GC06M043257	2.03	https://www.genecards.org/cgi-bin/carddisp.pl?gene=piR-54702
LOC105373170	Uncharacterized LOC105373170	RNA Gene	2	GC01M231811	2.03	https://www.genecards.org/cgi-bin/carddisp.pl?gene=LOC1053731
piR-45773		RNA Gene	2	GC06M043079	2.03	https://www.genecards.org/cgi-bin/carddisp.pl?gene=piR-45773
piR-55199		RNA Gene	2	GC06M043268	2.03	https://www.genecards.org/cgi-bin/carddisp.pl?gene=piR-55199
piR-46362		RNA Gene	2	GC06M043091	2.03	https://www.genecards.org/cgi-bin/carddisp.pl?gene=piR-46362
NONHSAG018421.2		RNA Gene	2	GC16P004337	2.03	https://www.genecards.org/cgi-bin/carddisp.pl?gene=NONHSAG01
lnc-KLHL24-6		RNA Gene	2	GC03P183484	2.03	https://www.genecards.org/cgi-bin/carddisp.pl?gene=lnc-KLHL24-
piR-44286		RNA Gene	2	GC06M043047	2.03	https://www.genecards.org/cgi-bin/carddisp.pl?gene=piR-44286
piR-37903		RNA Gene	2	GC06M042587	2.03	https://www.genecards.org/cgi-bin/carddisp.pl?gene=piR-37903
piR-37780		RNA Gene	2	GC06M042585	2.03	https://www.genecards.org/cgi-bin/carddisp.pl?gene=piR-37780

piR-45939		RNA Gene	2	GC06M043081	2.03	https://www.genecards.org/cgi-bin/carddisp.pl?gene=piR-45939
LOC105373943	Uncharacterized LOC105373943	RNA Gene	2	GC02M235676	2.03	https://www.genecards.org/cgi-bin/carddisp.pl?gene=LOC1053739
piR-61711		RNA Gene	2	GC06M043376	2.03	https://www.genecards.org/cgi-bin/carddisp.pl?gene=piR-61711
NONHSAG013110.2		RNA Gene	2	GC13P028704	2.03	https://www.genecards.org/cgi-bin/carddisp.pl?gene=NONHSAG01
LOC105379051	Uncharacterized LOC105379051	RNA Gene	2	GC05M082913	2.03	https://www.genecards.org/cgi-bin/carddisp.pl?gene=LOC1053790
RF00017-1416		RNA Gene	2	GC13P046128	2.03	https://www.genecards.org/cgi-bin/carddisp.pl?gene=RF00017-141
LOC107986000	Uncharacterized LOC107986000	RNA Gene	2	GC02P235730	2.03	https://www.genecards.org/cgi-bin/carddisp.pl?gene=LOC1079860
ENSG00000258506		Pseudogene	2	GC14P084951	2.03	https://www.genecards.org/cgi-bin/carddisp.pl?gene=ENSG000002
piR-30178		RNA Gene	2	GC06M042432	2.03	https://www.genecards.org/cgi-bin/carddisp.pl?gene=piR-30178
piR-55631		RNA Gene	2	GC06M043276	2.03	https://www.genecards.org/cgi-bin/carddisp.pl?gene=piR-55631
piR-30153		RNA Gene	2	GC06M042431	2.03	https://www.genecards.org/cgi-bin/carddisp.pl?gene=piR-30153
piR-47596		RNA Gene	2	GC06M043104	2.03	https://www.genecards.org/cgi-bin/carddisp.pl?gene=piR-47596
piR-30883		RNA Gene	2	GC06M042449	2.03	https://www.genecards.org/cgi-bin/carddisp.pl?gene=piR-30883
HSALNG0014027		RNA Gene	2	GC02M031425	2.03	https://www.genecards.org/cgi-bin/carddisp.pl?gene=HSALNG001
piR-36047		RNA Gene	2	GC06M042559	2.03	https://www.genecards.org/cgi-bin/carddisp.pl?gene=piR-36047
piR-55036-040		RNA Gene	2	GC02P031489	2.03	https://www.genecards.org/cgi-bin/carddisp.pl?gene=piR-55036-04
piR-46640		RNA Gene	2	GC06M043094	2.03	https://www.genecards.org/cgi-bin/carddisp.pl?gene=piR-46640
ENSG00000276067		Pseudogene	2	GC07M135246	2.03	https://www.genecards.org/cgi-bin/carddisp.pl?gene=ENSG000002
ENSG00000271078		Pseudogene	2	GC15M032251	2.03	https://www.genecards.org/cgi-bin/carddisp.pl?gene=ENSG000002
piR-31534-137		RNA Gene	2	GC13M028707	2.03	https://www.genecards.org/cgi-bin/carddisp.pl?gene=piR-31534-13
ENSG00000279912		Uncategorized	2	GC15P032182	2.03	https://www.genecards.org/cgi-bin/carddisp.pl?gene=ENSG000002
ENSG00000280306		Uncategorized	2	GC16P020487	2.03	https://www.genecards.org/cgi-bin/carddisp.pl?gene=ENSG000002
ENSG00000219736		Pseudogene	1	GC06M074850	2.03	https://www.genecards.org/cgi-bin/carddisp.pl?gene=ENSG000002
ENSG00000220748		Pseudogene	1	GC06P023972	2.03	https://www.genecards.org/cgi-bin/carddisp.pl?gene=ENSG000002
LOC102724253	Uncharacterized LOC102724253	RNA Gene	1	GC15M038755	2.03	https://www.genecards.org/cgi-bin/carddisp.pl?gene=LOC1027242
LOC107984123	Uncharacterized LOC107984123	RNA Gene	1	GC07M135263	2.03	https://www.genecards.org/cgi-bin/carddisp.pl?gene=LOC1079841
LOC105377705	Uncharacterized LOC105377705	RNA Gene	1	GC05M166230	2.03	https://www.genecards.org/cgi-bin/carddisp.pl?gene=LOC1053777
RPL23A	Ribosomal Protein L23a	Protein Coding	41	GC17P028719	2.03	https://www.genecards.org/cgi-bin/carddisp.pl?gene=RPL23A
RUVBL1	RuvB Like AAA ATPase 1	Protein Coding	44	GC03M128064	2.02	https://www.genecards.org/cgi-bin/carddisp.pl?gene=RUVBL1
IBTK	Inhibitor Of Bruton Tyrosine Kinase	Protein Coding	39	GC06M082169	2.02	https://www.genecards.org/cgi-bin/carddisp.pl?gene=IBTK
AUP1	AUP1 Lipid Droplet Regulating VLDL Assembly Fact	Protein Coding	37	GC02M074526	2.02	https://www.genecards.org/cgi-bin/carddisp.pl?gene=AUP1
ILF3	Interleukin Enhancer Binding Factor 3	Protein Coding	38	GC19P010625	2.01	https://www.genecards.org/cgi-bin/carddisp.pl?gene=ILF3
ARPC2	Actin Related Protein 2/3 Complex Subunit 2	Protein Coding	43	GC02P218217	1.99	https://www.genecards.org/cgi-bin/carddisp.pl?gene=ARPC2
RRBP1	Ribosome Binding Protein 1	Protein Coding	39	GC20M017613	1.99	https://www.genecards.org/cgi-bin/carddisp.pl?gene=RRBP1
RTRAF	RNA Transcription, Translation And Transport Factor	Protein Coding	28	GC14P051992	1.99	https://www.genecards.org/cgi-bin/carddisp.pl?gene=RTRAF
EIF4A1	Eukaryotic Translation Initiation Factor 4A1	Protein Coding	44	GC17P007572	1.99	https://www.genecards.org/cgi-bin/carddisp.pl?gene=EIF4A1
SRSF4	Serine And Arginine Rich Splicing Factor 4	Protein Coding	38	GC01M029147	1.98	https://www.genecards.org/cgi-bin/carddisp.pl?gene=SRSF4
SUGP1	SURP And G-Patch Domain Containing 1	Protein Coding	35	GC19M019276	1.98	https://www.genecards.org/cgi-bin/carddisp.pl?gene=SUGP1
LOC110366354	CYP3A4 5' Regulatory Region	Biological Region	1	GC07P099784	1.97	https://www.genecards.org/cgi-bin/carddisp.pl?gene=LOC1103663

ENHO	Energy Homeostasis Associated	Protein Coding	30	GC09M034516	1.97	https://www.genecards.org/cgi-bin/carddisp.pl?gene=ENHO
AHSA1	Activator Of HSP90 ATPase Activity 1	Protein Coding	39	GC14P077457	1.96	https://www.genecards.org/cgi-bin/carddisp.pl?gene=AHSA1
RNF114	Ring Finger Protein 114	Protein Coding	40	GC20P049936	1.96	https://www.genecards.org/cgi-bin/carddisp.pl?gene=RNF114
CTPS1	CTP Synthase 1	Protein Coding	44	GC01P040979	1.96	https://www.genecards.org/cgi-bin/carddisp.pl?gene=CTPS1
SEC16A	SEC16 Homolog A, Endoplasmic Reticulum Export Fa	Protein Coding	36	GC09M136440	1.96	https://www.genecards.org/cgi-bin/carddisp.pl?gene=SEC16A
RPS8	Ribosomal Protein S8	Protein Coding	39	GC01P044775	1.95	https://www.genecards.org/cgi-bin/carddisp.pl?gene=RPS8
TRIM33	Tripartite Motif Containing 33	Protein Coding	44	GC01M114392	1.95	https://www.genecards.org/cgi-bin/carddisp.pl?gene=TRIM33
TMSB4X	Thymosin Beta 4 X-Linked	Protein Coding	40	GC0XP012975	1.95	https://www.genecards.org/cgi-bin/carddisp.pl?gene=TMSB4X
SRRM1	Serine And Arginine Repetitive Matrix 1	Protein Coding	37	GC01P024631	1.93	https://www.genecards.org/cgi-bin/carddisp.pl?gene=SRRM1
DUSP26	Dual Specificity Phosphatase 26	Protein Coding	36	GC08M033591	1.93	https://www.genecards.org/cgi-bin/carddisp.pl?gene=DUSP26
EIF3E	Eukaryotic Translation Initiation Factor 3 Subunit E	Protein Coding	38	GC08M108201	1.93	https://www.genecards.org/cgi-bin/carddisp.pl?gene=EIF3E
DDX17	DEAD-Box Helicase 17	Protein Coding	40	GC22M038483	1.92	https://www.genecards.org/cgi-bin/carddisp.pl?gene=DDX17
CCT8	Chaperonin Containing TCP1 Subunit 8	Protein Coding	37	GC21M029055	1.91	https://www.genecards.org/cgi-bin/carddisp.pl?gene=CCT8
CAMKK2	Calcium/Calmodulin Dependent Protein Kinase Kinase	Protein Coding	45	GC12M121309	1.91	https://www.genecards.org/cgi-bin/carddisp.pl?gene=CAMKK2
EDC4	Enhancer Of MRNA Decapping 4	Protein Coding	38	GC16P067873	1.9	https://www.genecards.org/cgi-bin/carddisp.pl?gene=EDC4
EIF3B	Eukaryotic Translation Initiation Factor 3 Subunit B	Protein Coding	38	GC07P002354	1.9	https://www.genecards.org/cgi-bin/carddisp.pl?gene=EIF3B
HKDC1	Hexokinase Domain Containing 1	Protein Coding	40	GC10P069220	1.9	https://www.genecards.org/cgi-bin/carddisp.pl?gene=HKDC1
CKAP4	Cytoskeleton Associated Protein 4	Protein Coding	37	GC12M106237	1.9	https://www.genecards.org/cgi-bin/carddisp.pl?gene=CKAP4
HNRNPUL1	Heterogeneous Nuclear Ribonucleoprotein U Like 1	Protein Coding	37	GC19P041262	1.9	https://www.genecards.org/cgi-bin/carddisp.pl?gene=HNRNPUL1
MAPK13	Mitogen-Activated Protein Kinase 13	Protein Coding	48	GC06P047452	1.9	https://www.genecards.org/cgi-bin/carddisp.pl?gene=MAPK13
EIF3F	Eukaryotic Translation Initiation Factor 3 Subunit F	Protein Coding	42	GC11P007966	1.89	https://www.genecards.org/cgi-bin/carddisp.pl?gene=EIF3F
RBM14	RNA Binding Motif Protein 14	Protein Coding	36	GC11P066631	1.89	https://www.genecards.org/cgi-bin/carddisp.pl?gene=RBM14
UBE2D3	Ubiquitin Conjugating Enzyme E2 D3	Protein Coding	47	GC04M102794	1.88	https://www.genecards.org/cgi-bin/carddisp.pl?gene=UBE2D3
FARSA	Phenylalanyl-TRNA Synthetase Subunit Alpha	Protein Coding	40	GC19M012922	1.88	https://www.genecards.org/cgi-bin/carddisp.pl?gene=FARSA
HSD17B11	Hydroxysteroid 17-Beta Dehydrogenase 11	Protein Coding	40	GC04M087336	1.87	https://www.genecards.org/cgi-bin/carddisp.pl?gene=HSD17B11
PDIA4	Protein Disulfide Isomerase Family A Member 4	Protein Coding	42	GC07M149003	1.86	https://www.genecards.org/cgi-bin/carddisp.pl?gene=PDIA4
PGAM5	PGAM Family Member 5, Mitochondrial Serine/Threo	Protein Coding	34	GC12P132710	1.86	https://www.genecards.org/cgi-bin/carddisp.pl?gene=PGAM5
FAM3A	FAM3 Metabolism Regulating Signaling Molecule A	Protein Coding	35	GC0XM154506	1.85	https://www.genecards.org/cgi-bin/carddisp.pl?gene=FAM3A
SLC25A48	Solute Carrier Family 25 Member 48	Protein Coding	32	GC05P135579	1.84	https://www.genecards.org/cgi-bin/carddisp.pl?gene=SLC25A48
PKNOX1	PBX/Knotted 1 Homeobox 1	Protein Coding	40	GC21P042974	1.84	https://www.genecards.org/cgi-bin/carddisp.pl?gene=PKNOX1
TMEM97	Transmembrane Protein 97	Protein Coding	35	GC17P028319	1.83	https://www.genecards.org/cgi-bin/carddisp.pl?gene=TMEM97
PABPC4	Poly(A) Binding Protein Cytoplasmic 4	Protein Coding	41	GC01M039560	1.83	https://www.genecards.org/cgi-bin/carddisp.pl?gene=PABPC4
SF3B3	Splicing Factor 3b Subunit 3	Protein Coding	39	GC16P070523	1.83	https://www.genecards.org/cgi-bin/carddisp.pl?gene=SF3B3
C1QTNF9	C1q And TNF Related 9	Protein Coding	33	GC13P024307	1.82	https://www.genecards.org/cgi-bin/carddisp.pl?gene=C1QTNF9
MIR190B	MicroRNA 190b	RNA Gene	14	GC01M154193	1.8	https://www.genecards.org/cgi-bin/carddisp.pl?gene=MIR190B
CAND1	Cullin Associated And Neddylation Dissociated 1	Protein Coding	37	GC12P067270	1.8	https://www.genecards.org/cgi-bin/carddisp.pl?gene=CAND1
MYBBP1A	MYB Binding Protein 1a	Protein Coding	39	GC17M004538	1.79	https://www.genecards.org/cgi-bin/carddisp.pl?gene=MYBBP1A
USP48	Ubiquitin Specific Peptidase 48	Protein Coding	39	GC01M021678	1.79	https://www.genecards.org/cgi-bin/carddisp.pl?gene=USP48
TMC4	Transmembrane Channel Like 4	Protein Coding	35	GC19M054160	1.79	https://www.genecards.org/cgi-bin/carddisp.pl?gene=TMC4

TXNL1	Thioredoxin Like 1	Protein Coding	39	GC18M056597	1.78	https://www.genecards.org/cgi-bin/carddisp.pl?gene=TXNL1
HNRNPL	Heterogeneous Nuclear Ribonucleoprotein L	Protein Coding	39	GC19M038836	1.78	https://www.genecards.org/cgi-bin/carddisp.pl?gene=HNRNPL
DEFA1	Defensin Alpha 1	Protein Coding	40	GC08M006977	1.78	https://www.genecards.org/cgi-bin/carddisp.pl?gene=DEFA1
EIF4A2	Eukaryotic Translation Initiation Factor 4A2	Protein Coding	45	GC03P186783	1.78	https://www.genecards.org/cgi-bin/carddisp.pl?gene=EIF4A2
LAMP3	Lysosomal Associated Membrane Protein 3	Protein Coding	39	GC03M183122	1.77	https://www.genecards.org/cgi-bin/carddisp.pl?gene=LAMP3
DHX15	DEAH-Box Helicase 15	Protein Coding	38	GC04M024519	1.76	https://www.genecards.org/cgi-bin/carddisp.pl?gene=DHX15
SUB1	SUB1 Regulator Of Transcription	Protein Coding	37	GC05P032533	1.76	https://www.genecards.org/cgi-bin/carddisp.pl?gene=SUB1
TMBIM6	Transmembrane BAX Inhibitor Motif Containing 6	Protein Coding	36	GC12P049707	1.76	https://www.genecards.org/cgi-bin/carddisp.pl?gene=TMBIM6
ILF2	Interleukin Enhancer Binding Factor 2	Protein Coding	38	GC01M153661	1.74	https://www.genecards.org/cgi-bin/carddisp.pl?gene=ILF2
SRRM2	Serine/Arginine Repetitive Matrix 2	Protein Coding	33	GC16P004234	1.73	https://www.genecards.org/cgi-bin/carddisp.pl?gene=SRRM2
FAM3B	FAM3 Metabolism Regulating Signaling Molecule B	Protein Coding	39	GC21P041304	1.73	https://www.genecards.org/cgi-bin/carddisp.pl?gene=FAM3B
RTCB	RNA 2',3'-Cyclic Phosphate And 5'-OH Ligase	Protein Coding	35	GC22M032387	1.72	https://www.genecards.org/cgi-bin/carddisp.pl?gene=RTCB
OPRD1	Opioid Receptor Delta 1	Protein Coding	44	GC01P028812	1.71	https://www.genecards.org/cgi-bin/carddisp.pl?gene=OPRD1
GLT8D2	Glycosyltransferase 8 Domain Containing 2	Protein Coding	36	GC12M103988	1.71	https://www.genecards.org/cgi-bin/carddisp.pl?gene=GLT8D2
EBNA1BP2	EBNA1 Binding Protein 2	Protein Coding	37	GC01M043165	1.7	https://www.genecards.org/cgi-bin/carddisp.pl?gene=EBNA1BP2
LATS2	Large Tumor Suppressor Kinase 2	Protein Coding	44	GC13M020973	1.7	https://www.genecards.org/cgi-bin/carddisp.pl?gene=LATS2
GGT5	Gamma-Glutamyltransferase 5	Protein Coding	39	GC22M024219	1.68	https://www.genecards.org/cgi-bin/carddisp.pl?gene=GGT5
HMCES	5-Hydroxymethylcytosine Binding, ES Cell Specific	Protein Coding	32	GC03P129278	1.63	https://www.genecards.org/cgi-bin/carddisp.pl?gene=HMCES
SMOC2	SPARC Related Modular Calcium Binding 2	Protein Coding	41	GC06P168441	1.62	https://www.genecards.org/cgi-bin/carddisp.pl?gene=SMOC2
PHF2	PHD Finger Protein 2	Protein Coding	39	GC09P093576	1.6	https://www.genecards.org/cgi-bin/carddisp.pl?gene=PHF2
SNRNP40	Small Nuclear Ribonucleoprotein U5 Subunit 40	Protein Coding	36	GC01M031259	1.6	https://www.genecards.org/cgi-bin/carddisp.pl?gene=SNRNP40
FAT1	FAT Atypical Cadherin 1	Protein Coding	39	GC04M186587	1.59	https://www.genecards.org/cgi-bin/carddisp.pl?gene=FAT1
DDX46	DEAD-Box Helicase 46	Protein Coding	39	GC05P134758	1.56	https://www.genecards.org/cgi-bin/carddisp.pl?gene=DDX46
PHF5A	PHD Finger Protein 5A	Protein Coding	36	GC22M041459	1.56	https://www.genecards.org/cgi-bin/carddisp.pl?gene=PHF5A
KDM4B	Lysine Demethylase 4B	Protein Coding	43	GC19P004969	1.56	https://www.genecards.org/cgi-bin/carddisp.pl?gene=KDM4B
ARPC4	Actin Related Protein 2/3 Complex Subunit 4	Protein Coding	38	GC03P009792	1.55	https://www.genecards.org/cgi-bin/carddisp.pl?gene=ARPC4
NMBR	Neuromedin B Receptor	Protein Coding	42	GC06M142059	1.53	https://www.genecards.org/cgi-bin/carddisp.pl?gene=NMBR
UBXN1	UBX Domain Protein 1	Protein Coding	36	GC11M063437	1.53	https://www.genecards.org/cgi-bin/carddisp.pl?gene=UBXN1
SLC2A8	Solute Carrier Family 2 Member 8	Protein Coding	39	GC09P127398	1.53	https://www.genecards.org/cgi-bin/carddisp.pl?gene=SLC2A8
RGS4	Regulator Of G Protein Signaling 4	Protein Coding	43	GC01P163038	1.5	https://www.genecards.org/cgi-bin/carddisp.pl?gene=RGS4
FAIM	Fas Apoptotic Inhibitory Molecule	Protein Coding	39	GC03P138608	1.5	https://www.genecards.org/cgi-bin/carddisp.pl?gene=FAIM
USP10	Ubiquitin Specific Peptidase 10	Protein Coding	44	GC16P084734	1.49	https://www.genecards.org/cgi-bin/carddisp.pl?gene=USP10
CLGN	Calmegin	Protein Coding	37	GC04M140388	1.47	https://www.genecards.org/cgi-bin/carddisp.pl?gene=CLGN
LYPLAL1	Lysophospholipase Like 1	Protein Coding	37	GC01P219173	1.43	https://www.genecards.org/cgi-bin/carddisp.pl?gene=LYPLAL1
SH3BP5	SH3 Domain Binding Protein 5	Protein Coding	40	GC03M015805	1.42	https://www.genecards.org/cgi-bin/carddisp.pl?gene=SH3BP5
STK24	Serine/Threonine Kinase 24	Protein Coding	44	GC13M098445	1.42	https://www.genecards.org/cgi-bin/carddisp.pl?gene=STK24
IGHG4	Immunoglobulin Heavy Constant Gamma 4 (G4m Mar	Protein Coding	26	GC14M109516	1.41	https://www.genecards.org/cgi-bin/carddisp.pl?gene=IGHG4
DUSP14	Dual Specificity Phosphatase 14	Protein Coding	37	GC17P037489	1.38	https://www.genecards.org/cgi-bin/carddisp.pl?gene=DUSP14
SEC16B	SEC16 Homolog B, Endoplasmic Reticulum Export Fa	Protein Coding	37	GC01M177923	1.38	https://www.genecards.org/cgi-bin/carddisp.pl?gene=SEC16B

DNAJC10	DnaJ Heat Shock Protein Family (Hsp40) Member C1(	Protein Coding	37	GC02P182716	1.37	https://www.genecards.org/cgi-bin/carddisp.pl?gene=DNAJC10
RANGAP1	Ran GTPase Activating Protein 1	Protein Coding	41	GC22M041244	1.37	https://www.genecards.org/cgi-bin/carddisp.pl?gene=RANGAP1
H2BC18	H2B Clustered Histone 18	Protein Coding	27	GC01M149955	1.35	https://www.genecards.org/cgi-bin/carddisp.pl?gene=H2BC18
HSP90AA2P	Heat Shock Protein 90 Alpha Family Class A Member	Pseudogene	20	GC11M027888	1.34	https://www.genecards.org/cgi-bin/carddisp.pl?gene=HSP90AA2P
COP1	COP1 E3 Ubiquitin Ligase	Protein Coding	33	GC01M175944	1.33	https://www.genecards.org/cgi-bin/carddisp.pl?gene=COP1
H2BC11	H2B Clustered Histone 11	Protein Coding	27	GC06M027291	1.31	https://www.genecards.org/cgi-bin/carddisp.pl?gene=H2BC11
RAB32	RAB32, Member RAS Oncogene Family	Protein Coding	37	GC06P146543	1.3	https://www.genecards.org/cgi-bin/carddisp.pl?gene=RAB32
PNLIPRP2	Pancreatic Lipase Related Protein 2 (Gene/Pseudogene	Protein Coding	37	GC10P116620	1.3	https://www.genecards.org/cgi-bin/carddisp.pl?gene=PNLIPRP2
RAMP3	Receptor Activity Modifying Protein 3	Protein Coding	40	GC07P045163	1.28	https://www.genecards.org/cgi-bin/carddisp.pl?gene=RAMP3
TRIM47	Tripartite Motif Containing 47	Protein Coding	34	GC17M075874	1.28	https://www.genecards.org/cgi-bin/carddisp.pl?gene=TRIM47
TRPC4AP	Transient Receptor Potential Cation Channel Subfamil	Protein Coding	37	GC20M035002	1.27	https://www.genecards.org/cgi-bin/carddisp.pl?gene=TRPC4AP
ZNF300	Zinc Finger Protein 300	Protein Coding	36	GC05M150894	1.26	https://www.genecards.org/cgi-bin/carddisp.pl?gene=ZNF300
SDF2L1	Stromal Cell Derived Factor 2 Like 1	Protein Coding	35	GC22P024225	1.25	https://www.genecards.org/cgi-bin/carddisp.pl?gene=SDF2L1
BMP8B	Bone Morphogenetic Protein 8b	Protein Coding	39	GC01M039757	1.24	https://www.genecards.org/cgi-bin/carddisp.pl?gene=BMP8B
FNDC3B	Fibronectin Type III Domain Containing 3B	Protein Coding	36	GC03P172039	1.23	https://www.genecards.org/cgi-bin/carddisp.pl?gene=FNDC3B
DNAJC11	DnaJ Heat Shock Protein Family (Hsp40) Member C11	Protein Coding	35	GC01M006634	1.22	https://www.genecards.org/cgi-bin/carddisp.pl?gene=DNAJC11
FUNDC2	FUN14 Domain Containing 2	Protein Coding	33	GC0XP155025	1.22	https://www.genecards.org/cgi-bin/carddisp.pl?gene=FUNDC2
HIF3A	Hypoxia Inducible Factor 3 Subunit Alpha	Protein Coding	39	GC19P046297	1.21	https://www.genecards.org/cgi-bin/carddisp.pl?gene=HIF3A
IGKV2D-23	Immunoglobulin Kappa Variable 2D-23 (Pseudogene)	Pseudogene	5	GC02P090495	1.16	https://www.genecards.org/cgi-bin/carddisp.pl?gene=IGKV2D-23
RUBCNL	Rubicon Like Autophagy Enhancer	Protein Coding	25	GC13M046343	1.14	https://www.genecards.org/cgi-bin/carddisp.pl?gene=RUBCNL
LOC109279247	FGF21/FUT1 Promoter Region	Biological Region	1	GC19P048768	1.12	https://www.genecards.org/cgi-bin/carddisp.pl?gene=LOC1092792
LINC01684	Long Intergenic Non-Protein Coding RNA 1684	RNA Gene	9	GC21P024306	1.11	https://www.genecards.org/cgi-bin/carddisp.pl?gene=LINC01684
TXNDC5	Thioredoxin Domain Containing 5	Protein Coding	39	GC06M007893	1.1	https://www.genecards.org/cgi-bin/carddisp.pl?gene=TXNDC5
RPL17-C18orf32	RPL17-C18orf32 Readthrough	Protein Coding	21	GC18M049481	1.09	https://www.genecards.org/cgi-bin/carddisp.pl?gene=RPL17-C18or
PHLPP2	PH Domain And Leucine Rich Repeat Protein Phospha	Protein Coding	41	GC16M071637	1.09	https://www.genecards.org/cgi-bin/carddisp.pl?gene=PHLPP2
MIR103B2	MicroRNA 103b-2	RNA Gene	9	GC20M003899	1.06	https://www.genecards.org/cgi-bin/carddisp.pl?gene=MIR103B2
MIR103B1	MicroRNA 103b-1	RNA Gene	8	GC05P168560	1.06	https://www.genecards.org/cgi-bin/carddisp.pl?gene=MIR103B1
SAP18	Sin3A Associated Protein 18	Protein Coding	39	GC13P021140	0.99	https://www.genecards.org/cgi-bin/carddisp.pl?gene=SAP18
MAS1	MAS1 Proto-Oncogene, G Protein-Coupled Receptor	Protein Coding	40	GC06P159906	0.98	https://www.genecards.org/cgi-bin/carddisp.pl?gene=MAS1
CTDNEP1	CTD Nuclear Envelope Phosphatase 1	Protein Coding	35	GC17M007243	0.98	https://www.genecards.org/cgi-bin/carddisp.pl?gene=CTDNEP1
YIPF6	Yip1 Domain Family Member 6	Protein Coding	34	GC0XP068498	0.97	https://www.genecards.org/cgi-bin/carddisp.pl?gene=YIPF6
CARD6	Caspase Recruitment Domain Family Member 6	Protein Coding	38	GC05P040841	0.94	https://www.genecards.org/cgi-bin/carddisp.pl?gene=CARD6
SUCNR1	Succinate Receptor 1	Protein Coding	40	GC03P151873	0.93	https://www.genecards.org/cgi-bin/carddisp.pl?gene=SUCNR1
PRMT3	Protein Arginine Methyltransferase 3	Protein Coding	41	GC11P020409	0.9	https://www.genecards.org/cgi-bin/carddisp.pl?gene=PRMT3
HERC4	HECT And RLD Domain Containing E3 Ubiquitin Pro	Protein Coding	36	GC10M067921	0.85	https://www.genecards.org/cgi-bin/carddisp.pl?gene=HERC4
CR1L	Complement C3b/C4b Receptor 1 Like	Protein Coding	34	GC01P207646	0.83	https://www.genecards.org/cgi-bin/carddisp.pl?gene=CR1L
FAM174A	Family With Sequence Similarity 174 Member A	Protein Coding	32	GC05P100535	0.75	https://www.genecards.org/cgi-bin/carddisp.pl?gene=FAM174A
MIR4668	MicroRNA 4668	RNA Gene	12	GC09P111932	0.58	https://www.genecards.org/cgi-bin/carddisp.pl?gene=MIR4668
TM6SF1	Transmembrane 6 Superfamily Member 1	Protein Coding	32	GC15P083244	0.33	https://www.genecards.org/cgi-bin/carddisp.pl?gene=TM6SF1

Information of Disgenet											
**Disease**	**Disease_id**	**Gene**	**Gene_i**	**UniProt**	**ne_Full_Na**	**rotein_Cla**	**_diseases_**	**DSI_g**	**DPI_g**	**pLI**	**Score_gda**
Non-alcoholic Fatty	C0400966	TM6SF2	53345	Q9BZW4	transmembrane 6 supe		51	0.638	0.462	3.61E-05	0.6
Non-alcoholic Fatty	C0400966	PNPLA3	80339	Q9NST1	patatin like	Enzyme	132	0.556	0.692	5.74E-08	0.5
Non-alcoholic Fatty	C0400966	CYP2E1	1571	P05181	cytochrome P450 fam		306	0.459	0.885	8.07E-09	0.4
Non-alcoholic Fatty	C0400966	SIRT1	23411	Q96EB6	sirtuin 1	Epigenetic	675	0.378	0.885	0.087326	0.4
Non-alcoholic Fatty	C0400966	FGF21	26291	Q9NSA1	fibroblast g	Signaling	236	0.485	0.769	0.029439	0.4
Non-alcoholic Fatty	C0400966	LEP	3952	P41159	leptin		931	0.349	0.846	0.46491	0.4
Non-alcoholic Fatty	C0400966	NFE2L2	4780	Q16236	nuclear fac	Enzyme	823	0.357	0.885	0.003572	0.4
Non-alcoholic Fatty	C0400966	PPARA	5465	Q07869	peroxisome	Nuclear re	408	0.432	0.885	0.030182	0.4
Non-alcoholic Fatty	C0400966	ADIPOQ	9370	Q15848	adiponectin, C1Q and		679	0.376	0.885	4.64E-09	0.4
Non-alcoholic Fatty	C0400966	NR1H4	9971	Q96RI1	nuclear rec	Nuclear re	187	0.513	0.808	0.000939	0.4
Non-alcoholic Fatty	C0400966	PPARD	5467	Q03181	peroxisome	Nuclear re	197	0.513	0.846	0.99497	0.38
Non-alcoholic Fatty	C0400966	SREBF1	6720	P36956	sterol regulatory elem		159	0.518	0.808	0.014708	0.38
Non-alcoholic Fatty	C0400966	LDLR	3949	P01130	low density lipoprotei		336	0.449	0.885	9.84E-24	0.37
Non-alcoholic Fatty	C0400966	XBP1	7494	P17861	X-box binding protein		234	0.477	0.846	0.032014	0.37
Non-alcoholic Fatty	C0400966	CAT	847	P04040	catalase	Enzyme	794	0.359	0.962	1.63E-10	0.37
Non-alcoholic Fatty	C0400966	FAS	355	P25445	Fas cell surface death		754	0.372	0.923	0.8117	0.36
Non-alcoholic Fatty	C0400966	GNMT	27232	Q14749	glycine N-methyltrans		57	0.626	0.462	0.002815	0.35
Non-alcoholic Fatty	C0400966	PEMT	10400	Q9UBM1	phosphatidylethanolar		43	0.653	0.538	0.23456	0.34
Non-alcoholic Fatty	C0400966	CPT1A	1374	P50416	carnitine pa	Enzyme	143	0.555	0.808	5.11E-09	0.34
Non-alcoholic Fatty	C0400966	NR5A2	2494	O00482	nuclear rec	Nuclear re	116	0.563	0.731	0.23807	0.34
Non-alcoholic Fatty	C0400966	TGFB1	7040	P01137	transformi	Signaling	1558	0.287	0.962	0.03685	0.34
Non-alcoholic Fatty	C0400966	AHR	196	P35869	aryl hydroc	Transcripti	532	0.41	0.923	0.99999	0.33
Non-alcoholic Fatty	C0400966	GSTM1	2944	P09488	glutathione S-transfera		627	0.38	0.923	0.002064	0.33
Non-alcoholic Fatty	C0400966	GSTP1	2950	P09211	glutathione S-transfera		610	0.383	0.923	0.014155	0.33
Non-alcoholic Fatty	C0400966	IL1A	3552	P01583	interleukin 1 alpha		1002	0.333	0.962	0.00016	0.33
Non-alcoholic Fatty	C0400966	JAK2	3717	O60674	Janus kinas	Kinase	644	0.385	0.885	0.65372	0.33
Non-alcoholic Fatty	C0400966	PRKCA	5578	P17252	protein kin	Kinase	368	0.44	0.846	0.3677	0.33
Non-alcoholic Fatty	C0400966	ABCC2	1244	Q92887	ATP bindi	Transporte	254	0.483	0.808	5.2E-47	0.32
Non-alcoholic Fatty	C0400966	KLB	#####	Q86Z14	klotho beta	Enzyme	53	0.647	0.5	0.036433	0.32
Non-alcoholic Fatty	C0400966	ACE	1636	P12821	angiotensi	Enzyme	1082	0.328	0.923	1.03E-37	0.32
Non-alcoholic Fatty	C0400966	ALDH2	217	P05091	aldehyde d	Enzyme	337	0.457	0.885	3.4E-10	0.32
Non-alcoholic Fatty	C0400966	GSTT1	2952	P30711	glutathione S-transfera		541	0.393	0.923	0.000148	0.32
Non-alcoholic Fatty	C0400966	PTEN	5728	P60484	phosphatas	Enzyme	1349	0.305	0.923	0.25651	0.32
Non-alcoholic Fatty	C0400966	CD14	929	P08571	CD14 molecule		551	0.392	0.885	4.88E-05	0.32
Non-alcoholic Fatty	C0400966	NQO1	1728	P15559	NAD(P)H quinone de		368	0.434	0.885	1.19E-09	0.31
Non-alcoholic Fatty	C0400966	FOLR2	2350	P14207	folate receptor beta		105	0.559	0.654	5.48E-06	0.31

Non-alcoholic Fatty	C0400966	GSTA1	2938	P08263	glutathione S-transfera		108	0.561	0.731	1.73E-06	0.31
Non-alcoholic Fatty	C0400966	IL4	3565	P05112	interleukin 4		996	0.332	0.962	0.004737	0.31
Non-alcoholic Fatty	C0400966	LIF	3976	P15018	LIF interle	Signaling	236	0.483	0.769	0.5242	0.31
Non-alcoholic Fatty	C0400966	PDK4	5166	Q16654	pyruvate de	Kinase	85	0.578	0.577	9.14E-12	0.31
Non-alcoholic Fatty	C0400966	PRKCD	5580	Q05655	protein kin	Kinase	287	0.48	0.846	0.99995	0.31
Non-alcoholic Fatty	C0400966	PRKCE	5581	Q02156	protein kin	Kinase	112	0.597	0.692	0.99988	0.31
Non-alcoholic Fatty	C0400966	VLDLR	7436	P98155	very low density lipop		143	0.558	0.692	1.96E-11	0.31
Non-alcoholic Fatty	C0400966	ADH1A	124	P07327	alcohol del	Enzyme	50	0.647	0.577	1.71E-11	0.3
Non-alcoholic Fatty	C0400966	ADH1B	125	P00325	alcohol del	Enzyme	224	0.502	0.846	9.93E-07	0.3
Non-alcoholic Fatty	C0400966	ADH4	127	P08319	alcohol del	Enzyme	53	0.641	0.462	8.07E-07	0.3
Non-alcoholic Fatty	C0400966	CSF2	1437	P04141	colony stin	Signaling	1028	0.33	0.962	0.83468	0.3
Non-alcoholic Fatty	C0400966	CYP1A2	1544	P05177	cytochrome	Enzyme	218	0.494	0.923	2.48E-10	0.3
Non-alcoholic Fatty	C0400966	CYP17A1	1586	P05093	cytochrome P450 fam		326	0.462	0.769	0.000263	0.3
Non-alcoholic Fatty	C0400966	AHCY	191	P23526	adenosylho	Enzyme	107	0.582	0.692	0.035922	0.3
Non-alcoholic Fatty	C0400966	F2	2147	P00734	coagulation	Enzyme	490	0.415	0.885	0.001112	0.3
Non-alcoholic Fatty	C0400966	ALDH1A1	216	P00352	aldehyde d	Enzyme	270	0.46	0.846	0.95448	0.3
Non-alcoholic Fatty	C0400966	ALDH1B1	219	P30837	aldehyde d	Enzyme	31	0.691	0.462	2.7E-06	0.3
Non-alcoholic Fatty	C0400966	B3GAT1	27087	Q9P2W7	beta-1,3-gl	Enzyme	199	0.494	0.769	0.13521	0.3
Non-alcoholic Fatty	C0400966	EIF2AK1	27102	Q9BQI3	eukaryotic	Kinase	56	0.633	0.577	7.01E-07	0.3
Non-alcoholic Fatty	C0400966	LAMA1	#####	P25391	laminin subunit alpha		107	0.582	0.808	3.22E-32	0.3
Non-alcoholic Fatty	C0400966	IL3	3562	P08700	interleukin 3		211	0.489	0.731	2.59E-09	0.3
Non-alcoholic Fatty	C0400966	MMP1	4312	P03956	matrix met	Enzyme	589	0.385	0.885	7.72E-18	0.3
Non-alcoholic Fatty	C0400966	SERPINB2	5055	P05120	serpin fam	Enzyme m	183	0.502	0.808	6.38E-12	0.3
Non-alcoholic Fatty	C0400966	ABCB4	5244	P21439	ATP bindi	Transporte	157	0.529	0.615	2.21E-07	0.3
Non-alcoholic Fatty	C0400966	PRF1	5551	P14222	perforin 1		163	0.54	0.692	5.49E-06	0.3
Non-alcoholic Fatty	C0400966	PRKACA	5566	P17612	protein kin	Kinase	88	0.606	0.692	0.97142	0.3
Non-alcoholic Fatty	C0400966	TRIB3	57761	Q96RU7	tribbles ps	Kinase	104	0.573	0.692	1.16E-13	0.3
Non-alcoholic Fatty	C0400966	RAG2	5897	P55895	recombination activati		207	0.507	0.769	0.020287	0.3
Non-alcoholic Fatty	C0400966	RDX	5962	P35241	radixin	Cellular st	80	0.593	0.615	0.85245	0.3
Non-alcoholic Fatty	C0400966	TNFRSF1B	7133	P20333	TNF receptor superfa		417	0.425	0.885	0.50201	0.3
Non-alcoholic Fatty	C0400966	IKBKG	8517	Q9Y6K9	inhibitor of	Kinase	252	0.493	0.769	0.084853	0.3
Non-alcoholic Fatty	C0400966	STC2	8614	O76061	stanniocalc	Signaling	88	0.573	0.692	0.26588	0.3
Non-alcoholic Fatty	C0400966	ALDH4A1	8659	P30038	aldehyde dehydrogena		12	0.792	0.231	1.37E-05	0.3
Non-alcoholic Fatty	C0400966	SCARB1	949	Q8WTV0	scavenger	Receptor	116	0.559	0.731	0.066228	0.3
Non-alcoholic Fatty	C0400966	GCKR	2646	Q14397	glucokinase regulator		136	0.604	0.615	8.54E-19	0.2
Non-alcoholic Fatty	C0400966	ATP5F1B	506	P06576	ATP synth	Transporte	62	0.626	0.654	0.97679	0.2
Non-alcoholic Fatty	C0400966	NCAN	1463	O14594	neurocan	Extracellul	44	0.67	0.5	0.068	0.18
Non-alcoholic Fatty	C0400966	PARVB	29780	Q9HBI1	parvin beta	Cellular st	25	0.736	0.385	2.38E-13	0.14
Non-alcoholic Fatty	C0400966	SAMM50	25813	Q9Y512	SAMM50 sorting and		14	0.78	0.269	4.88E-09	0.13

Non-alcoholic Fatty	C0400966	FDFT1	2222	P37268	farnesyl-diphosphate f		62	0.621	0.615	4.1E-15	0.12
Non-alcoholic Fatty	C0400966	PTPRD	5789	P23468	protein tyro	Enzyme	93	0.584	0.731	1	0.11
Non-alcoholic Fatty	C0400966	ZGLP1	#####	P0C6A0	zinc finger	GATA like	186	0.512	0.808	0.3868	0.1
Non-alcoholic Fatty	C0400966	PPARGC1A	10891	Q9UBK2	PPARG cc	Transcripti	350	0.453	0.885	0.9973	0.1
Non-alcoholic Fatty	C0400966	NLRP3	#####	Q96P20	NLR family pyrin dom		805	0.361	0.962	0.000811	0.1
Non-alcoholic Fatty	C0400966	OSBPL5	#####	Q9H0X9	oxysterol binding prot		14	0.792	0.346	4.65E-06	0.1
Non-alcoholic Fatty	C0400966	COL13A1	1305	Q5TAT6	collagen type XIII alp		139	0.608	0.654	3.74E-14	0.1
Non-alcoholic Fatty	C0400966	CRP	1401	P02741	C-reactive protein		1483	0.299	0.962	0.003697	0.1
Non-alcoholic Fatty	C0400966	DPP4	1803	P27487	dipeptidyl	Enzyme	451	0.42	0.808	7.28E-09	0.1
Non-alcoholic Fatty	C0400966	ERBB4	2066	Q15303	erb-b2 rece	Kinase	317	0.455	0.846	0.99912	0.1
Non-alcoholic Fatty	C0400966	FBL	2091	P22087	fibrillarin	Enzyme	94	0.572	0.654	0.99319	0.1
Non-alcoholic Fatty	C0400966	ALB	213	P02768	albumin	Transporte	1198	0.317	0.962	0.64329	0.1
Non-alcoholic Fatty	C0400966	EHBP1L1	#####	Q8N3D4	EH domain binding pr		12	0.89	0.115	7.12E-13	0.1
Non-alcoholic Fatty	C0400966	GCG	2641	P01275	glucagon		441	0.431	0.885	0.034814	0.1
Non-alcoholic Fatty	C0400966	SLC17A5	26503	Q9NRA2	solute carri	Transporte	252	0.491	0.808	6.85E-10	0.1
Non-alcoholic Fatty	C0400966	GGT1	2678	P19440	gamma-glu	Enzyme	209	0.496	0.885	7.53E-05	0.1
Non-alcoholic Fatty	C0400966	GLP1R	2740	P43220	glucagon li	G-protein c	288	0.471	0.846	0.29347	0.1
Non-alcoholic Fatty	C0400966	GPT	2875	P24298	glutamic--p	Enzyme	511	0.403	0.923	3.59E-19	0.1
Non-alcoholic Fatty	C0400966	HFE	3077	Q30201	homeostatic iron regul		415	0.436	0.846	2.56E-08	0.1
Non-alcoholic Fatty	C0400966	APOC3	345	P02656	apolipoprotein C3		153	0.531	0.731	0.010024	0.1
Non-alcoholic Fatty	C0400966	IGF1	3479	P05019	insulin like growth fac		1206	0.318	0.885	0.2716	0.1
Non-alcoholic Fatty	C0400966	IL1B	3553	P01584	interleukin 1 beta		1801	0.276	0.962	0.13005	0.1
Non-alcoholic Fatty	C0400966	IL6	3569	P05231	interleukin 6		2367	0.248	0.962	0.31536	0.1
Non-alcoholic Fatty	C0400966	KRT18	3875	P05783	keratin 18		151	0.53	0.692	0.73477	0.1
Non-alcoholic Fatty	C0400966	LEPR	3953	P48357	leptin recep	Signaling	416	0.433	0.808	0.99475	0.1
Non-alcoholic Fatty	C0400966	LIPA	3988	P38571	lipase A, ly	Enzyme	130	0.564	0.654	5.53E-07	0.1
Non-alcoholic Fatty	C0400966	MIR122	#####		microRNA 122		250	0.468	0.808		0.1
Non-alcoholic Fatty	C0400966	MIR21	#####		microRNA 21		726	0.363	0.846		0.1
Non-alcoholic Fatty	C0400966	MIR34A	#####		microRNA 34a		428	0.421	0.846		0.1
Non-alcoholic Fatty	C0400966	MTTP	4547	P55157	microsoma	Transporte	209	0.505	0.846	1.04E-05	0.1
Non-alcoholic Fatty	C0400966	PIK3CA	5290	P42336	phosphatid	Kinase	1511	0.292	0.923	1	0.1
Non-alcoholic Fatty	C0400966	PIK3CB	5291	P42338	phosphatid	Kinase	1083	0.322	0.885	0.99964	0.1
Non-alcoholic Fatty	C0400966	PIK3CD	5293	O00329	phosphatid	Kinase	1119	0.319	0.885	0.99999	0.1
Non-alcoholic Fatty	C0400966	PIK3CG	5294	P48736	phosphatid	Kinase	1101	0.32	0.885	1.41E-06	0.1
Non-alcoholic Fatty	C0400966	PPARG	5468	P37231	peroxisome	Nuclear re	877	0.358	0.885	0.029194	0.1
Non-alcoholic Fatty	C0400966	GATAD2A	54815	Q86YP4	GATA zinc	Nucleic ac	19	0.792	0.231	0.99957	0.1
Non-alcoholic Fatty	C0400966	PRKAA1	5562	Q13131	protein kin	Kinase	325	0.453	0.846	0.13725	0.1
Non-alcoholic Fatty	C0400966	PRKAA2	5563	P54646	protein kin	Kinase	317	0.456	0.846	3.53E-08	0.1
Non-alcoholic Fatty	C0400966	PRKAB1	5564	Q9Y478	protein kin	Kinase	296	0.46	0.846	0.005257	0.1

Non-alcoholic Fatty	C0400966	RBP4	5950	P02753	retinol bin	Transporte	217	0.493	0.808	0.51951	0.1
Non-alcoholic Fatty	C0400966	SCD	6319	O00767	stearoyl-CoA desatura		225	0.49	0.808	0.96968	0.1
Non-alcoholic Fatty	C0400966	SHBG	6462	P04278	sex hormone binding g		368	0.438	0.808	1.25E-06	0.1
Non-alcoholic Fatty	C0400966	SLC5A2	6524	P31639	solute carri	Transporte	214	0.499	0.769	4.92E-14	0.1
Non-alcoholic Fatty	C0400966	GGTLC5P	#####		gamma-glutamyltrans		165	0.513	0.846		0.1
Non-alcoholic Fatty	C0400966	SP4	6671	Q02446	Sp4 transc	Nucleic ac	30	0.711	0.423	0.99288	0.1
Non-alcoholic Fatty	C0400966	SREBF2	6721	Q12772	sterol regulatory elem		112	0.566	0.577	0.20734	0.1
Non-alcoholic Fatty	C0400966	TLR4	7099	O00206	toll like receptor 4		1174	0.321	0.962	4.61E-09	0.1
Non-alcoholic Fatty	C0400966	TNF	7124	P01375	tumor necr	Signaling	2724	0.231	0.962	0.8033	0.1
Non-alcoholic Fatty	C0400966	GGTLC3	#####	B5MD39	gamma-glutamyltrans		165	0.513	0.846		0.1
Non-alcoholic Fatty	C0400966	GGT2	#####	P36268	gamma-glutamyltrans		165	0.513	0.846	0.00013	0.1
Non-alcoholic Fatty	C0400966	GGTLC4P	#####		gamma-glutamyltrans		165	0.513	0.846		0.1
Non-alcoholic Fatty	C0400966	CNBP	7555	P62633	CCHC-type zinc finge		125	0.556	0.692	0.95994	0.1
Non-alcoholic Fatty	C0400966	MBOAT7	79143	Q96N66	membrane	Enzyme	83	0.612	0.5	0.113	0.1
Non-alcoholic Fatty	C0400966	EHMT1	79813	Q9H9B1	euchromati	Epigenetic	371	0.46	0.808	1	0.1
Non-alcoholic Fatty	C0400966	ACTR5	79913	Q9H9F9	actin relate	Cellular st	2	0.931	0.077	1.14E-09	0.1
Non-alcoholic Fatty	C0400966	ZNF512	84450	Q96ME7	zinc finger protein 51		15	0.839	0.192	0.9765	0.1
Non-alcoholic Fatty	C0400966	CRACR2A	84766	Q9BSW2	calcium release activa		9	0.792	0.231	7.43E-12	0.1
Non-alcoholic Fatty	C0400966	PLPP7	84814	Q8NBV4	phospholip	Enzyme	1	1	0.038	0.007998	0.1
Non-alcoholic Fatty	C0400966	PWWP3A	84939	Q2TAK8	PWWP domain contai		49	0.636	0.385	0.002152	0.1
Non-alcoholic Fatty	C0400966	TP63	8626	Q9H3D4	tumor prot	Transcripti	816	0.362	0.769	0.99728	0.1
Non-alcoholic Fatty	C0400966	DDX60L	91351	Q5H9U9	DExD/H-box 60 like		4	0.861	0.115	1.07E-38	0.1
Non-alcoholic Fatty	C0400966	XPR1	9213	Q9UBH6	xenotropic	Transporte	217	0.488	0.731	0.99999	0.1
Non-alcoholic Fatty	C0400966	CD36	948	P16671	CD36 mol	Receptor	351	0.446	0.846	1.09E-60	0.1
Non-alcoholic Fatty	C0400966	CREB5	9586	Q02930	cAMP resp	Transcripti	48	0.682	0.538	0.22337	0.1
Non-alcoholic Fatty	C0400966	FGF19	9965	O95750	fibroblast g	Signaling	123	0.548	0.615	0.57022	0.1
Non-alcoholic Fatty	C0400966	AHSG	197	P02765	alpha 2-HS	Enzyme m	204	0.506	0.846	3.21E-10	0.09
Non-alcoholic Fatty	C0400966	FASN	2194	P49327	fatty acid s	Enzyme	245	0.474	0.846	1	0.09
Non-alcoholic Fatty	C0400966	GABPA	2551	Q06546	GA bindin	Transcripti	632	0.379	0.885	0.99812	0.09
Non-alcoholic Fatty	C0400966	GH1	2688	P01241	growth hor	Signaling	686	0.373	0.923	0.030057	0.09
Non-alcoholic Fatty	C0400966	LBP	3929	P18428	lipopolysaccharide bi		156	0.525	0.808	4.19E-15	0.09
Non-alcoholic Fatty	C0400966	LGALS3BP	3959	Q08380	galectin 3 binding pro		127	0.546	0.769	2.68E-07	0.09
Non-alcoholic Fatty	C0400966	HAMP	57817	P81172	hepcidin antimicrobia		377	0.433	0.846	0.00818	0.09
Non-alcoholic Fatty	C0400966	REN	5972	P00797	renin	Enzyme	721	0.37	0.923	2.43E-07	0.09
Non-alcoholic Fatty	C0400966	NR1H3	10062	Q13133	nuclear rec	Nuclear re	102	0.566	0.692	0.90374	0.08
Non-alcoholic Fatty	C0400966	CYP3A4	1576	P08684	cytochrom	Enzyme	291	0.462	0.885	5.8E-11	0.08
Non-alcoholic Fatty	C0400966	FABP1	2168	P07148	fatty acid binding prot		85	0.588	0.692	9.8E-07	0.08
Non-alcoholic Fatty	C0400966	SIRT3	23410	Q9NTG7	sirtuin 3	Epigenetic	227	0.488	0.731	0.000163	0.08
Non-alcoholic Fatty	C0400966	HNF4A	3172	P41235	hepatocyte	Nuclear re	340	0.461	0.808	0.15285	0.08

Non-alcoholic Fatty	C0400966	HSD17B13	#####	Q7Z5P4	hydroxysteroid 17-bet		58	0.623	0.538	2.74E-05	0.08
Non-alcoholic Fatty	C0400966	IL17A	3605	Q16552	interleukin 17A		1074	0.324	0.923	0.043049	0.08
Non-alcoholic Fatty	C0400966	MLXIPL	51085	Q9NP71	MLX interacting prote		165	0.554	0.731	0.047771	0.08
Non-alcoholic Fatty	C0400966	CCL2	6347	P13500	C-C motif	Signaling	1157	0.321	0.962	0.60786	0.08
Non-alcoholic Fatty	C0400966	LOC102724197	#####		inactive glutathione hy		105	0.559	0.731		0.07
Non-alcoholic Fatty	C0400966	AGTR1	185	P30556	angiotensi	G-protein c	440	0.423	0.846	0.000532	0.07
Non-alcoholic Fatty	C0400966	MTOR	2475	P42345	mechanisti	Kinase	960	0.343	0.885	1	0.07
Non-alcoholic Fatty	C0400966	FETUB	26998	Q9UGM5	fetuin B	Enzyme m	38	0.686	0.308	8.88E-08	0.07
Non-alcoholic Fatty	C0400966	APOE	348	P02649	apolipoprotein E		1049	0.338	0.962	0.001869	0.07
Non-alcoholic Fatty	C0400966	PON1	5444	P27169	paraoxonase 1		496	0.409	0.885	9.81E-11	0.07
Non-alcoholic Fatty	C0400966	RETN	56729	Q9HD89	resistin		302	0.454	0.808	0.036545	0.07
Non-alcoholic Fatty	C0400966	RARRES2	5919	Q99969	retinoic acid receptor r		180	0.519	0.731	2.72E-05	0.07
Non-alcoholic Fatty	C0400966	UCP2	7351	P55851	uncoupling	Transporte	235	0.493	0.808	1.55E-10	0.07
Non-alcoholic Fatty	C0400966	NR0B2	8431	Q15466	nuclear rec	Nuclear re	251	0.474	0.808	8.34E-06	0.07
Non-alcoholic Fatty	C0400966	GGTLC1	92086	Q9BX51	gamma-glu	Enzyme	105	0.559	0.731	3.34E-05	0.07
Non-alcoholic Fatty	C0400966	DECR1	1666	Q16698	2,4-dienoy	Enzyme	399	0.426	0.885	4.47E-14	0.06
Non-alcoholic Fatty	C0400966	PCSK9	#####	Q8NBP7	proprotein	Enzyme	257	0.482	0.808	2.71E-17	0.06
Non-alcoholic Fatty	C0400966	ACACA	31	Q13085	acetyl-CoA carboxyla		241	0.48	0.846	1	0.06
Non-alcoholic Fatty	C0400966	HMGB1	3146	P09429	high mobil	Nucleic ac	724	0.368	0.923	0.82035	0.06
Non-alcoholic Fatty	C0400966	APOB	338	P04114	apolipoprotein B		339	0.453	0.808	1.92E-16	0.06
Non-alcoholic Fatty	C0400966	IL10	3586	P22301	interleukin 10		1679	0.281	0.923	0.005887	0.06
Non-alcoholic Fatty	C0400966	LPL	4023	P06858	lipoprotein	Enzyme	290	0.474	0.808	1.27E-09	0.06
Non-alcoholic Fatty	C0400966	MIR192	#####		microRNA 192		173	0.503	0.846		0.06
Non-alcoholic Fatty	C0400966	MTHFR	4524	P42898	methylenetetrahydrofo		985	0.337	0.885	3.21E-10	0.06
Non-alcoholic Fatty	C0400966	SERPINE1	5054	P05121	serpin fam	Enzyme m	770	0.359	0.885	0.043609	0.06
Non-alcoholic Fatty	C0400966	ANGPTL8	55908	Q6UXH0	angiopoietin like 8		54	0.65	0.577	6.88E-12	0.06
Non-alcoholic Fatty	C0400966	SOD1	6647	P00441	superoxide	Enzyme	689	0.379	0.962	0.1773	0.06
Non-alcoholic Fatty	C0400966	TP53	7157	P04637	tumor prote	Transcripti	2494	0.236	0.962	0.53235	0.06
Non-alcoholic Fatty	C0400966	VDR	7421	P11473	vitamin D	Nuclear re	852	0.352	0.885	1.68E-05	0.06
Non-alcoholic Fatty	C0400966	IRS2	8660	Q9Y4H2	insulin receptor substr		152	0.535	0.654	0.99032	0.06
Non-alcoholic Fatty	C0400966	TRIB1	10221	Q96RU8	tribbles pse	Kinase	61	0.617	0.577	0.83343	0.05
Non-alcoholic Fatty	C0400966	TXNIP	10628	Q9H3M7	thioredoxin interacting		208	0.49	0.731	0.15918	0.05
Non-alcoholic Fatty	C0400966	PLIN2	123	Q99541	perilipin 2	Transporte	100	0.564	0.692	7.53E-09	0.05
Non-alcoholic Fatty	C0400966	CNR1	1268	P21554	cannabinoi	G-protein c	393	0.446	0.808	0.50652	0.05
Non-alcoholic Fatty	C0400966	CYBB	1536	P04839	cytochrom	Ion channe	343	0.452	0.885	0.99904	0.05
Non-alcoholic Fatty	C0400966	AKT1	207	P31749	AKT serin	Kinase	1250	0.311	0.962	0.9759	0.05
Non-alcoholic Fatty	C0400966	ABCD1	215	P33897	ATP bindi	Transporte	154	0.563	0.731	0.99896	0.05
Non-alcoholic Fatty	C0400966	FOXO1	2308	Q12778	forkhead box O1		380	0.433	0.846	0.99725	0.05
Non-alcoholic Fatty	C0400966	GOLGA6A	#####	Q9NYA3	golgin A6 family mem		90	0.592	0.654	1.61E-05	0.05

Non-alcoholic Fatty	C0400966	APRT	353	P07741	adenine phosphoribos		384	0.423	0.846	5.45E-13	0.05
Non-alcoholic Fatty	C0400966	IRS1	3667	P35568	insulin receptor substr		233	0.488	0.769	0.023645	0.05
Non-alcoholic Fatty	C0400966	MIR33A	#####		microRNA 33a		100	0.56	0.654		0.05
Non-alcoholic Fatty	C0400966	MFAP1	4236	P55081	microfibril	Extracellul	357	0.431	0.846	0.79602	0.05
Non-alcoholic Fatty	C0400966	NR3C2	4306	P08235	nuclear rec	Nuclear re	287	0.469	0.846	0.83739	0.05
Non-alcoholic Fatty	C0400966	SERPINA1	5265	P01009	serpin fam	Enzyme m	482	0.41	0.923	6.85E-08	0.05
Non-alcoholic Fatty	C0400966	RCBTB1	55213	Q8NDN9	RCC1 and	Enzyme m	112	0.572	0.692	1.56E-06	0.05
Non-alcoholic Fatty	C0400966	PNPLA2	57104	Q96AD5	patatin like	Enzyme	199	0.511	0.846	0.004107	0.05
Non-alcoholic Fatty	C0400966	SELENOP	6414	P49908	selenoprot	Extracellul	138	0.545	0.769	8.29E-07	0.05
Non-alcoholic Fatty	C0400966	SOD2	6648	P04179	superoxide	Enzyme	668	0.379	0.923	0.15499	0.05
Non-alcoholic Fatty	C0400966	STAT3	6774	P40763	signal tran	Nucleic ac	1193	0.32	0.923	1	0.05
Non-alcoholic Fatty	C0400966	PPP1R3B	79660	Q86XI6	protein pho	Enzyme m	31	0.686	0.462	0.065025	0.05
Non-alcoholic Fatty	C0400966	NR1I2	8856	O75469	nuclear rec	Nuclear re	462	0.418	0.846	7.27E-09	0.05
Non-alcoholic Fatty	C0400966	FADS2	9415	O95864	fatty acid desaturase 2		140	0.575	0.692	0.99815	0.05
Non-alcoholic Fatty	C0400966	CEBPA	1050	P49715	CCAAT en	Transcripti	201	0.496	0.692	0.55365	0.04
Non-alcoholic Fatty	C0400966	CETP	1071	P11597	cholesteryl ester trans		188	0.512	0.731	1.19E-23	0.04
Non-alcoholic Fatty	C0400966	SLC27A5	10998	Q9Y2P5	solute carri	Transporte	111	0.563	0.769	8.37E-15	0.04
Non-alcoholic Fatty	C0400966	APOA5	#####	Q6Q788	apolipoprotein A5		107	0.587	0.615	5.58E-10	0.04
Non-alcoholic Fatty	C0400966	COL1A1	1277	P02452	collagen type I alpha 1		487	0.43	0.808	1	0.04
Non-alcoholic Fatty	C0400966	KLF6	1316	Q99612	Kruppel lik	Nucleic ac	204	0.501	0.846	0.97946	0.04
Non-alcoholic Fatty	C0400966	NRG4	#####	Q8WWG1	neuregulin	Signaling	71	0.623	0.654	0.027981	0.04
Non-alcoholic Fatty	C0400966	TMC4	#####	Q7Z404	transmemb	Ion channe	12	0.805	0.192	1.72E-13	0.04
Non-alcoholic Fatty	C0400966	EGFR	1956	P00533	epidermal	Kinase	1394	0.295	0.885	0.36837	0.04
Non-alcoholic Fatty	C0400966	EPAS1	2034	Q99814	endothelial	Transcripti	293	0.454	0.731	0.5961	0.04
Non-alcoholic Fatty	C0400966	FABP4	2167	P15090	fatty acid binding prot		194	0.5	0.808	5.18E-05	0.04
Non-alcoholic Fatty	C0400966	FGL1	2267	Q08830	fibrinogen	Signaling	53	0.638	0.692	1.68E-25	0.04
Non-alcoholic Fatty	C0400966	FLII	2314	Q13045	FLII actin remodeling		198	0.52	0.808	5.53E-12	0.04
Non-alcoholic Fatty	C0400966	GCK	2645	P35557	glucokinas	Kinase	210	0.523	0.808	0.63464	0.04
Non-alcoholic Fatty	C0400966	IFNL3	#####	Q8IZI9	interferon lambda 3		175	0.506	0.808	2.42E-08	0.04
Non-alcoholic Fatty	C0400966	HIF1A	3091	Q16665	hypoxia in	Transcripti	1044	0.327	0.923	0.9777	0.04
Non-alcoholic Fatty	C0400966	HP	3240	P00738	haptoglobi	Enzyme	464	0.412	0.885	1.74E-05	0.04
Non-alcoholic Fatty	C0400966	HSD11B1	3290	P28845	hydroxysteroid 11-bet		163	0.52	0.731	0.41696	0.04
Non-alcoholic Fatty	C0400966	IFNG	3458	P01579	interferon gamma		1519	0.288	0.962	0.47156	0.04
Non-alcoholic Fatty	C0400966	APP	351	P05067	amyloid be	Enzyme m	485	0.422	0.846	0.046544	0.04
Non-alcoholic Fatty	C0400966	IL18	3606	Q14116	interleukin 18		750	0.365	0.923	0.030717	0.04
Non-alcoholic Fatty	C0400966	INSR	3643	P06213	insulin rec	Kinase	452	0.432	0.846	3.61E-05	0.04
Non-alcoholic Fatty	C0400966	MIR29A	#####		microRNA 29a		278	0.459	0.808		0.04
Non-alcoholic Fatty	C0400966	MAP3K5	4217	Q99683	mitogen-ac	Kinase	170	0.513	0.808	0.51964	0.04
Non-alcoholic Fatty	C0400966	CHPT1	56994	Q8WUD6	choline ph	Enzyme	253	0.474	0.846	0.000243	0.04

Non-alcoholic Fatty	C0400966	BGLAP	632	P02818	bone gamma-carboxyg		274	0.462	0.846	4.62E-08	0.04
Non-alcoholic Fatty	C0400966	SPP1	6696	P10451	secreted phosphoprote		824	0.353	0.885	2.15E-06	0.04
Non-alcoholic Fatty	C0400966	TLR2	7097	O60603	toll like receptor 2		749	0.361	0.923	1.02E-06	0.04
Non-alcoholic Fatty	C0400966	CCR2	#####	P41597	C-C motif chemokine		436	0.418	0.846	0.023412	0.04
Non-alcoholic Fatty	C0400966	VEGFA	7422	P15692	vascular er	Signaling	1899	0.266	0.923	2.41E-05	0.04
Non-alcoholic Fatty	C0400966	FTO	79068	Q9C0B1	FTO alpha-ketoglutara		286	0.486	0.885	8.36E-08	0.04
Non-alcoholic Fatty	C0400966	CASP1	834	P29466	caspase 1	Enzyme	444	0.413	0.885	3.52E-05	0.04
Non-alcoholic Fatty	C0400966	ABCB11	8647	O95342	ATP bindi	Transporte	126	0.566	0.654	1.56E-12	0.04
Non-alcoholic Fatty	C0400966	TNFSF10	8743	P50591	TNF super	Signaling	445	0.413	0.885	0.13495	0.04
Non-alcoholic Fatty	C0400966	NAMPT	10135	P43490	nicotinami	Signaling	199	0.502	0.808	0.97914	0.03
Non-alcoholic Fatty	C0400966	FST	10468	P19883	follistatin	Enzyme m	182	0.513	0.731	0.98014	0.03
Non-alcoholic Fatty	C0400966	PRRT2	#####	Q7Z6L0	proline rich	transmem	353	0.448	0.808	0.57919	0.03
Non-alcoholic Fatty	C0400966	CMKLR1	1240	Q99788	chemerin c	G-protein c	75	0.612	0.692	0.010767	0.03
Non-alcoholic Fatty	C0400966	CPT2	1376	P23786	carnitine pa	Enzyme	187	0.542	0.769	4.6E-10	0.03
Non-alcoholic Fatty	C0400966	PARP1	142	P09874	poly(ADP-ribose) poly		565	0.389	0.923	0.000334	0.03
Non-alcoholic Fatty	C0400966	CTNNB1	1499	P35222	catenin beta 1		1368	0.303	0.885	0.99999	0.03
Non-alcoholic Fatty	C0400966	GPBAR1	#####	Q8TDU6	G protein-c	G-protein c	122	0.548	0.731	1.36E-09	0.03
Non-alcoholic Fatty	C0400966	CYP7A1	1581	P22680	cytochrom	Enzyme	91	0.576	0.615	0.000245	0.03
Non-alcoholic Fatty	C0400966	DLAT	1737	P10515	dihydrolipoamide S-ac		93	0.576	0.731	2.3E-08	0.03
Non-alcoholic Fatty	C0400966	AFP	174	P02771	alpha fetop	Transporte	392	0.429	0.885	1.14E-11	0.03
Non-alcoholic Fatty	C0400966	AGER	177	Q15109	advanced g	Receptor	450	0.42	0.885	6.35E-16	0.03
Non-alcoholic Fatty	C0400966	AGT	183	P01019	angiotensi	Enzyme m	765	0.367	0.923	4.97E-08	0.03
Non-alcoholic Fatty	C0400966	ABCA1	19	O95477	ATP bindi	Transporte	291	0.467	0.846	1.45E-13	0.03
Non-alcoholic Fatty	C0400966	FGFR4	2264	P22455	fibroblast g	Kinase	220	0.49	0.769	7.19E-13	0.03
Non-alcoholic Fatty	C0400966	SMUG1	23583	Q53HV7	single-strand-selective		1034	0.322	0.923	0.000452	0.03
Non-alcoholic Fatty	C0400966	GIP	2695	P09681	gastric inhibitory poly		110	0.57	0.654	0.27108	0.03
Non-alcoholic Fatty	C0400966	HPGDS	27306	O60760	hematopoietic prostag		570	0.388	0.923	1.81E-06	0.03
Non-alcoholic Fatty	C0400966	GOT2	2806	P00505	glutamic-oxaloacetic t		148	0.529	0.731	0.014233	0.03
Non-alcoholic Fatty	C0400966	NEAT1	#####		nuclear paraspeckle as		179	0.502	0.769		0.03
Non-alcoholic Fatty	C0400966	OR10A4	#####	Q9H209	olfactory re	G-protein c	128	0.54	0.808	1.68E-05	0.03
Non-alcoholic Fatty	C0400966	GPX1	2876	P07203	glutathione	Enzyme	290	0.458	0.923	3.85E-05	0.03
Non-alcoholic Fatty	C0400966	ANPEP	290	P15144	alanyl ami	Enzyme	280	0.462	0.769	3.75E-09	0.03
Non-alcoholic Fatty	C0400966	NPC1L1	29881	Q9UHC9	NPC1 like	Transporte	40	0.656	0.423	2.31E-20	0.03
Non-alcoholic Fatty	C0400966	HMOX1	3162	P09601	heme oxygenase 1		666	0.381	0.923	0.009682	0.03
Non-alcoholic Fatty	C0400966	CXCL10	3627	P02778	C-X-C mo	Signaling	631	0.378	0.885	0.36865	0.03
Non-alcoholic Fatty	C0400966	CYP4F3	4051	Q08477	cytochrom	Enzyme	114	0.551	0.808	3.54E-16	0.03
Non-alcoholic Fatty	C0400966	ARNTL	406	O00327	aryl hydro	Transcripti	200	0.51	0.885	0.99444	0.03
Non-alcoholic Fatty	C0400966	MIR140	#####		microRNA 140		144	0.522	0.769		0.03
Non-alcoholic Fatty	C0400966	MIR155	#####		microRNA 155		584	0.384	0.885		0.03

Non-alcoholic Fatty	C0400966	MMP2	4313	P08253	matrix met	Enzyme	1021	0.333	0.923	0.83878	0.03
Non-alcoholic Fatty	C0400966	MT1B	4490	P07438	metallothionein 1B		89	0.585	0.731	0.005378	0.03
Non-alcoholic Fatty	C0400966	ACLY	47	P53396	ATP citrate	Enzyme	109	0.563	0.692	0.083556	0.03
Non-alcoholic Fatty	C0400966	NTS	4922	P30990	neurotensin		184	0.517	0.769	3.85E-05	0.03
Non-alcoholic Fatty	C0400966	MIR378A	#####		microRNA 378a		127	0.532	0.808		0.03
Non-alcoholic Fatty	C0400966	BCO1	53630	Q9HAY6	beta-carote	Enzyme	29	0.705	0.462	5.5E-12	0.03
Non-alcoholic Fatty	C0400966	PRKCB	5579	P05771	protein kin	Kinase	317	0.453	0.885	1	0.03
Non-alcoholic Fatty	C0400966	MAPK8	5599	P45983	mitogen-ac	Kinase	520	0.397	0.885	0.99823	0.03
Non-alcoholic Fatty	C0400966	PRL	5617	P01236	prolactin	Signaling	506	0.406	0.885	2.9E-07	0.03
Non-alcoholic Fatty	C0400966	MIR451A	#####		microRNA 451a		177	0.505	0.769		0.03
Non-alcoholic Fatty	C0400966	CXCL16	58191	Q9H2A7	C-X-C mo	Signaling	178	0.502	0.769	3.45E-05	0.03
Non-alcoholic Fatty	C0400966	ACE2	59272	Q9BYF1	angiotensi	Enzyme	253	0.477	0.769	0.99769	0.03
Non-alcoholic Fatty	C0400966	BCL2	596	P10415	BCL2 apo	Signaling	1456	0.291	0.885	0.55903	0.03
Non-alcoholic Fatty	C0400966	BCL2A1	597	Q16548	BCL2 relat	Signaling	150	0.529	0.731	0.20785	0.03
Non-alcoholic Fatty	C0400966	SAV1	60485	Q9H4B6	salvador family WW		42	0.659	0.5	0.000904	0.03
Non-alcoholic Fatty	C0400966	SLC10A2	6555	Q12908	solute carri	Transporte	66	0.621	0.538	9.58E-17	0.03
Non-alcoholic Fatty	C0400966	SMN1	6606	Q16637	survival of	Epigenetic	317	0.454	0.769	0.015829	0.03
Non-alcoholic Fatty	C0400966	SMN2	6607	Q16637	survival of motor neur		299	0.459	0.769	0.10337	0.03
Non-alcoholic Fatty	C0400966	BNIP3	664	Q12983	BCL2 interacting prot		158	0.52	0.731	1.97E-05	0.03
Non-alcoholic Fatty	C0400966	TERT	7015	O14746	telomerase	Enzyme	703	0.374	0.846	0.99046	0.03
Non-alcoholic Fatty	C0400966	TIMP1	7076	P01033	TIMP meta	Enzyme m	603	0.38	0.885	0.4993	0.03
Non-alcoholic Fatty	C0400966	TIMP2	7077	P16035	TIMP meta	Enzyme m	404	0.425	0.846	0.78893	0.03
Non-alcoholic Fatty	C0400966	ADIPOR2	79602	Q86V24	adiponecti	Receptor	103	0.564	0.731	0.36218	0.03
Non-alcoholic Fatty	C0400966	BCO2	83875	Q9BYV7	beta-carotene oxygena		19	0.78	0.231	3.78E-18	0.03
Non-alcoholic Fatty	C0400966	CAV1	857	Q03135	caveolin 1	Enzyme m	633	0.388	0.885	0.008274	0.03
Non-alcoholic Fatty	C0400966	GDF15	9518	Q99988	growth diff	Signaling	389	0.429	0.808	3.01E-07	0.03
Non-alcoholic Fatty	C0400966	BMS1	9790	Q14692	BMS1 ribosome bioge		192	0.499	0.846	0.000175	0.03
Non-alcoholic Fatty	C0400966	NR1I3	9970	Q14994	nuclear rec	Nuclear re	318	0.448	0.846	3.53E-08	0.03
Non-alcoholic Fatty	C0400966	FAS-AS1	#####		FAS antisense RNA 1		29	0.695	0.538		0.02
Non-alcoholic Fatty	C0400966	LINC01672	#####		long intergenic non-pr		268	0.462	0.846		0.02
Non-alcoholic Fatty	C0400966	PSC	#####		Cholangitis, primary s		67	0.603	0.731		0.02
Non-alcoholic Fatty	C0400966	TLR6	10333	Q9Y2C9	toll like receptor 6		106	0.556	0.769	2.25E-05	0.02
Non-alcoholic Fatty	C0400966	YAP1	10413	P46937	Yes associ	Enzyme m	374	0.432	0.808	0.99918	0.02
Non-alcoholic Fatty	C0400966	POSTN	10631	Q15063	periostin	Signaling	378	0.428	0.885	1.07E-09	0.02
Non-alcoholic Fatty	C0400966	CES1	1066	P23141	carboxyles	Enzyme	80	0.59	0.692	3.32E-15	0.02
Non-alcoholic Fatty	C0400966	CORIN	10699	Q9Y5Q5	corin, serine peptidase		55	0.695	0.538	6.51E-35	0.02
Non-alcoholic Fatty	C0400966	CCL27	10850	Q9Y4X3	C-C motif	Signaling	185	0.502	0.846	0.013511	0.02
Non-alcoholic Fatty	C0400966	MTMR11	10903	A4FU01	myotubula	Enzyme	32	0.691	0.538	4.77E-23	0.02
Non-alcoholic Fatty	C0400966	CHIT1	1118	Q13231	chitinase 1		142	0.525	0.692	7.17E-16	0.02

Non-alcoholic Fatty	C0400966	ECD	11319	O95905	ecdysoneless cell cycl		40	0.656	0.538	2.34E-12	0.02
Non-alcoholic Fatty	C0400966	C1QTNF1	#####	Q9BXJ1	C1q and TNF related		27	0.7	0.346	0.000424	0.02
Non-alcoholic Fatty	C0400966	LYPLAL1	#####	Q5VWZ2	lysophospholipase like		8	0.821	0.192	2.64E-05	0.02
Non-alcoholic Fatty	C0400966	BHLHE23	#####	Q8NDY6	basic helix	Enzyme	22	0.722	0.385	0.19596	0.02
Non-alcoholic Fatty	C0400966	MAP3K8	1326	P41279	mitogen-ac	Kinase	168	0.516	0.846	0.96825	0.02
Non-alcoholic Fatty	C0400966	COX8A	1351	P10176	cytochrome	Enzyme	526	0.4	0.846	0.11606	0.02
Non-alcoholic Fatty	C0400966	GPR119	#####	Q8TDV5	G protein-c	G-protein c	27	0.695	0.346	0.008618	0.02
Non-alcoholic Fatty	C0400966	CYBA	1535	P13498	cytochrome b-245 alp		177	0.52	0.769	8.87E-06	0.02
Non-alcoholic Fatty	C0400966	CYP1A1	1543	P04798	cytochrome	Enzyme	379	0.436	0.846	1.06E-17	0.02
Non-alcoholic Fatty	C0400966	GRK2	156	P25098	G protein-c	Kinase	159	0.532	0.654	0.99998	0.02
Non-alcoholic Fatty	C0400966	CYP2D6	1565	P10635	cytochrome P450 fam		432	0.437	0.885	1.08E-25	0.02
Non-alcoholic Fatty	C0400966	ATN1	1822	P54259	atrophin 1		499	0.409	0.885	0.99992	0.02
Non-alcoholic Fatty	C0400966	MLKL	#####	Q8NB16	mixed linea	Kinase	93	0.57	0.731	2.09E-14	0.02
Non-alcoholic Fatty	C0400966	ELANE	1991	P08246	elastase, ne	Enzyme	346	0.447	0.846	0.001227	0.02
Non-alcoholic Fatty	C0400966	ELAVL2	1993	Q12926	ELAV like	RNA bind	199	0.486	0.808	0.99514	0.02
Non-alcoholic Fatty	C0400966	ESR1	2099	P03372	estrogen re	Nuclear re	1101	0.324	0.962	0.9992	0.02
Non-alcoholic Fatty	C0400966	ESRRA	2101	P11474	estrogen re	Nuclear re	112	0.548	0.654	0.59696	0.02
Non-alcoholic Fatty	C0400966	ACSL4	2182	O60488	acyl-CoA s	Enzyme	136	0.561	0.769	0.98103	0.02
Non-alcoholic Fatty	C0400966	FAT1	2195	Q14517	FAT atypical cadherin		178	0.508	0.808	1.2E-12	0.02
Non-alcoholic Fatty	C0400966	FCGR2B	2213	P31994	Fc fragmen	Cell adhes	155	0.522	0.846	0.45637	0.02
Non-alcoholic Fatty	C0400966	SIRT2	22933	Q8IXJ6	sirtuin 2	Epigenetic	172	0.51	0.769	2.33E-08	0.02
Non-alcoholic Fatty	C0400966	FOXO3	2309	O43524	forkhead box O3		381	0.431	0.808	0.98806	0.02
Non-alcoholic Fatty	C0400966	LPIN1	23175	Q14693	lipin 1		86	0.601	0.692	6.45E-13	0.02
Non-alcoholic Fatty	C0400966	MLYCD	23417	O95822	malonyl-CoA decarbo		79	0.617	0.769	0.000648	0.02
Non-alcoholic Fatty	C0400966	FOS	2353	P01100	Fos proto-c	Transcripti	528	0.411	0.885	0.26176	0.02
Non-alcoholic Fatty	C0400966	ALOX12	239	P18054	arachidona	Enzyme	119	0.558	0.692	1.44E-15	0.02
Non-alcoholic Fatty	C0400966	ALOX5	240	P09917	arachidona	Enzyme	342	0.447	0.885	1.21E-06	0.02
Non-alcoholic Fatty	C0400966	ALOX15	246	P16050	arachidona	Enzyme	205	0.502	0.846	1.14E-23	0.02
Non-alcoholic Fatty	C0400966	ALPP	250	P05187	alkaline ph	Enzyme	209	0.487	0.808	1.81E-13	0.02
Non-alcoholic Fatty	C0400966	FNDC5	#####	Q8NAU1	fibronectin type III do		61	0.631	0.538	0.5458	0.02
Non-alcoholic Fatty	C0400966	ATRNL1	26033	Q5VV63	attractin lik	Enzyme m	117	0.546	0.769	0.99915	0.02
Non-alcoholic Fatty	C0400966	GC	2638	P02774	GC vitamin	Transporte	219	0.489	0.885	2.73E-11	0.02
Non-alcoholic Fatty	C0400966	GCGR	2642	P47871	glucagon r	G-protein c	47	0.656	0.308	2.22E-06	0.02
Non-alcoholic Fatty	C0400966	PDLIM3	27295	Q53GG5	PDZ and L	Cellular str	118	0.547	0.769	3.03E-07	0.02
Non-alcoholic Fatty	C0400966	GLUL	2752	P15104	glutamate-	Enzyme	200	0.513	0.731	0.9957	0.02
Non-alcoholic Fatty	C0400966	SLC13A5	#####	Q86YT5	solute carri	Transporte	106	0.608	0.577	0.000506	0.02
Non-alcoholic Fatty	C0400966	GRN	2896	P28799	granulin precursor		412	0.435	0.846	0.069666	0.02
Non-alcoholic Fatty	C0400966	SETD2	29072	Q9BYW2	SET domai	Epigenetic	447	0.424	0.846	1	0.02
Non-alcoholic Fatty	C0400966	NR3C1	2908	P04150	nuclear rec	Nuclear re	590	0.393	0.885	0.96958	0.02

Non-alcoholic Fatty	C0400966	TBK1	29110	Q9UHD2	TANK bin	Kinase	207	0.513	0.846	0.075174	0.02
Non-alcoholic Fatty	C0400966	PDIA3	2923	P30101	protein disulfide isom		100	0.566	0.769	0.99608	0.02
Non-alcoholic Fatty	C0400966	FOXA1	3169	P55317	forkhead b	Transcripti	138	0.522	0.731	0.22227	0.02
Non-alcoholic Fatty	C0400966	ACACB	32	O00763	acetyl-CoA carboxyla		49	0.644	0.654	7.65E-47	0.02
Non-alcoholic Fatty	C0400966	HSPA1A	3303	P0DMV8;P0DMV	heat shock protein fan		229	0.486	0.769	0.030228	0.02
Non-alcoholic Fatty	C0400966	HSPA1B	3304	P0DMV8;P0DMV	heat shock protein fan		251	0.474	0.808	0.005346	0.02
Non-alcoholic Fatty	C0400966	HSP90AA1	3320	P07900	heat shock	Chaperone	455	0.411	0.923	0.86025	0.02
Non-alcoholic Fatty	C0400966	FFAR4	#####	Q5NUL3	free fatty acid recepto		67	0.621	0.577	1.36E-05	0.02
Non-alcoholic Fatty	C0400966	IDH2	3418	P48735	isocitrate dehydrogena		380	0.434	0.808	0.88271	0.02
Non-alcoholic Fatty	C0400966	IRGM	#####	A1A4Y4	immunity related GTF		57	0.617	0.538		0.02
Non-alcoholic Fatty	C0400966	IGFALS	3483	P35858	insulin like	Receptor	203	0.511	0.846	5.44E-11	0.02
Non-alcoholic Fatty	C0400966	CXCL8	3576	P10145	C-X-C mo	Signaling	1254	0.31	0.962	0.000274	0.02
Non-alcoholic Fatty	C0400966	INS	3630	P01308	insulin		405	0.445	0.923	0.30091	0.02
Non-alcoholic Fatty	C0400966	AQP9	366	O43315	aquaporin !	Ion channe	100	0.565	0.808	0.24027	0.02
Non-alcoholic Fatty	C0400966	AR	367	P10275	androgen r	Nuclear re	854	0.351	0.846	0.98837	0.02
Non-alcoholic Fatty	C0400966	ENHO	#####	Q6UWT2	energy homeostasis as		71	0.599	0.769	0.10526	0.02
Non-alcoholic Fatty	C0400966	LCN2	3934	P80188	lipocalin 2	Enzyme	497	0.405	0.885	1.14E-09	0.02
Non-alcoholic Fatty	C0400966	LECT2	3950	O14960	leukocyte (	Signaling	47	0.653	0.5	0.001481	0.02
Non-alcoholic Fatty	C0400966	MIR126	#####		microRNA 126		305	0.447	0.846		0.02
Non-alcoholic Fatty	C0400966	MIR149	#####		microRNA 149		140	0.524	0.769		0.02
Non-alcoholic Fatty	C0400966	MIR20A	#####		microRNA 20a		180	0.498	0.769		0.02
Non-alcoholic Fatty	C0400966	MIR200C	#####		microRNA 200c		202	0.486	0.846		0.02
Non-alcoholic Fatty	C0400966	MIR222	#####		microRNA 222		210	0.486	0.808		0.02
Non-alcoholic Fatty	C0400966	MAS1	4142	P04201	MAS1 pro	G-protein (	142	0.533	0.769	8.25E-05	0.02
Non-alcoholic Fatty	C0400966	MC4R	4160	P32245	melanocort	G-protein (	149	0.535	0.692	0.000917	0.02
Non-alcoholic Fatty	C0400966	MMP13	4322	P45452	matrix met	Enzyme	328	0.452	0.923	7.88E-18	0.02
Non-alcoholic Fatty	C0400966	PLIN5	#####	Q00G26	perilipin 5		27	0.695	0.346	1.05E-11	0.02
Non-alcoholic Fatty	C0400966	MUC1	4582	P15941	mucin 1, cell surface a		594	0.384	0.885	0.018774	0.02
Non-alcoholic Fatty	C0400966	ATF3	467	P18847	activating t	Transcripti	228	0.494	0.885	0.089296	0.02
Non-alcoholic Fatty	C0400966	ATHS	470		atherosclerosis suscep		112	0.551	0.769		0.02
Non-alcoholic Fatty	C0400966	CCN3	4856	P48745	cellular co	Signaling	117	0.554	0.615	0.006644	0.02
Non-alcoholic Fatty	C0400966	MIR375	#####		microRNA 375		226	0.476	0.769		0.02
Non-alcoholic Fatty	C0400966	TNFRSF11B	4982	O00300	TNF receptor superfa		443	0.422	0.808	0.16171	0.02
Non-alcoholic Fatty	C0400966	IL22	50616	Q9GZX6	interleukin 22		551	0.393	0.885	0.000188	0.02
Non-alcoholic Fatty	C0400966	ACOX1	51	Q15067	acyl-CoA (	Enzyme	95	0.61	0.731	0.062095	0.02
Non-alcoholic Fatty	C0400966	ADIPOR1	51094	Q96A54	adiponecti	Receptor	148	0.529	0.808	0.6584	0.02
Non-alcoholic Fatty	C0400966	CRNKL1	51340	Q9BZJ0	crooked ne	Nucleic ac	24	0.711	0.385	2.72E-06	0.02
Non-alcoholic Fatty	C0400966	HSD17B7	51478	P56937	hydroxysteroid 17-bet		81	0.582	0.5	6.65E-06	0.02
Non-alcoholic Fatty	C0400966	GDE1	51573	Q9NZC3	glycerophosphodiester		228	0.481	0.808	6.12E-06	0.02

Non-alcoholic Fatty	C0400966	ENPP1	5167	P22413	ectonucleo	Enzyme	247	0.498	0.808	4.99E-08	0.02
Non-alcoholic Fatty	C0400966	LUC7L3	51747	O95232	LUC7 like 3 pre-mRN		26	0.691	0.5	0.99999	0.02
Non-alcoholic Fatty	C0400966	FAM3B	54097	P58499	FAM3 metabolism reg		38	0.682	0.385	2.43E-08	0.02
Non-alcoholic Fatty	C0400966	UGT1A1	54658	P22309	UDP glucuronosyltran		302	0.464	0.769	3.26E-06	0.02
Non-alcoholic Fatty	C0400966	ELOVL2	54898	Q9NXB9	ELOVL fa	Enzyme	26	0.722	0.423	8.37E-10	0.02
Non-alcoholic Fatty	C0400966	AVP	551	P01185	arginine va	Signaling	454	0.437	0.846	0.074413	0.02
Non-alcoholic Fatty	C0400966	NAT10	55226	Q9H0A0	N-acetyltransferase 10		133	0.536	0.769	4.12E-22	0.02
Non-alcoholic Fatty	C0400966	PTPA	5524	Q15257	protein pho	Enzyme m	245	0.48	0.846	0.99057	0.02
Non-alcoholic Fatty	C0400966	MEG3	55384		maternally expressed 3		239	0.471	0.846		0.02
Non-alcoholic Fatty	C0400966	MAPK1	5594	P28482	mitogen-ac	Kinase	1059	0.33	0.923	0.99698	0.02
Non-alcoholic Fatty	C0400966	MYDGF	56005	Q969H8	myeloid derived grow		295	0.451	0.846	3.49E-06	0.02
Non-alcoholic Fatty	C0400966	AZGP1	563	P25311	alpha-2-glycoprotein 1		101	0.566	0.769	4.15E-06	0.02
Non-alcoholic Fatty	C0400966	PRTN3	5657	P24158	proteinase	Enzyme	215	0.503	0.808	3.8E-05	0.02
Non-alcoholic Fatty	C0400966	PTGS1	5742	P23219	prostagland	Enzyme	318	0.46	0.885	6.24E-07	0.02
Non-alcoholic Fatty	C0400966	MIR146B	#####		microRNA 146b		187	0.496	0.769		0.02
Non-alcoholic Fatty	C0400966	PTPN1	5770	P18031	protein tyrosine phosp		169	0.523	0.731	0.81293	0.02
Non-alcoholic Fatty	C0400966	NLRC4	58484	Q9NPP4	NLR family CARD do		145	0.556	0.769	9.87E-12	0.02
Non-alcoholic Fatty	C0400966	BCAT1	586	P54687	branched c	Enzyme	62	0.606	0.538	3.8E-08	0.02
Non-alcoholic Fatty	C0400966	MOK	5891	Q9UQ07	MOK prote	Kinase	251	0.476	0.769	8.84E-22	0.02
Non-alcoholic Fatty	C0400966	FAM3A	60343	P98173	FAM3 metabolism reg		8	0.805	0.269	0.66899	0.02
Non-alcoholic Fatty	C0400966	ROCK1	6093	Q13464	Rho associ	Kinase	157	0.513	0.731	1	0.02
Non-alcoholic Fatty	C0400966	HCC	#####		thyroid carcinoma, Hu		45	0.644	0.423		0.02
Non-alcoholic Fatty	C0400966	S100A8	6279	P05109	S100 calciu	Calcium-b	369	0.43	0.962	0.13761	0.02
Non-alcoholic Fatty	C0400966	CEACAM1	634	P13688	CEA cell adhesion mo		167	0.525	0.731	0.000788	0.02
Non-alcoholic Fatty	C0400966	SCP2	6342	P22307	sterol carrier protein 2		50	0.644	0.5	8.75E-07	0.02
Non-alcoholic Fatty	C0400966	CCL20	6364	P78556	C-C motif	Signaling	242	0.474	0.846	0.000242	0.02
Non-alcoholic Fatty	C0400966	SELPLG	6404	Q14242	selectin P ligand		113	0.564	0.692	0.1768	0.02
Non-alcoholic Fatty	C0400966	ABCG5	64240	Q9H222	ATP bindi	Transporte	108	0.592	0.577	3.08E-21	0.02
Non-alcoholic Fatty	C0400966	IL25	64806	Q9H293	interleukin 25		139	0.529	0.846	0.000923	0.02
Non-alcoholic Fatty	C0400966	SLC2A1	6513	P11166	solute carri	Transporte	687	0.388	0.808	0.9941	0.02
Non-alcoholic Fatty	C0400966	SLC2A2	6514	P11168	solute carri	Transporte	123	0.572	0.692	3.43E-08	0.02
Non-alcoholic Fatty	C0400966	NCF1	#####	P14598	neutrophil cytosolic fa		170	0.521	0.808	2.83E-05	0.02
Non-alcoholic Fatty	C0400966	SLPI	6590	P03973	secretory le	Enzyme m	310	0.449	0.846	0.000897	0.02
Non-alcoholic Fatty	C0400966	SPARC	6678	P09486	secreted pr	Signaling	344	0.445	0.846	0.89148	0.02
Non-alcoholic Fatty	C0400966	SPINK1	6690	P00995	serine peptidase inhib		216	0.492	0.808	0.31476	0.02
Non-alcoholic Fatty	C0400966	SPTBN1	6711	Q01082	spectrin beta, non-eryt		76	0.619	0.538	1	0.02
Non-alcoholic Fatty	C0400966	STAT5A	6776	P42229	signal trans	Nucleic ac	318	0.445	0.808	0.99994	0.02
Non-alcoholic Fatty	C0400966	STAT5B	6777	P51692	signal trans	Nucleic ac	357	0.442	0.808	0.99988	0.02
Non-alcoholic Fatty	C0400966	TEK	7010	Q02763	TEK recep	Kinase	300	0.462	0.692	1	0.02

Non-alcoholic Fatty	C0400966	TRPV1	7442	Q8NER1	transient re	Ion channe	404	0.446	0.885	1.01E-16	0.02
Non-alcoholic Fatty	C0400966	DHRS11	79154	Q6UWP2	dehydroge	Enzyme	43	0.653	0.423	2.24E-06	0.02
Non-alcoholic Fatty	C0400966	STEAP4	79689	Q687X5	STEAP4 metalloreduc		34	0.686	0.462	1.2E-07	0.02
Non-alcoholic Fatty	C0400966	CALCR	799	P30988	calcitonin	G-protein	282	0.464	0.846	3.12E-09	0.02
Non-alcoholic Fatty	C0400966	ASRGL1	80150	Q7L266	asparagina	Enzyme	205	0.494	0.808	2.76E-05	0.02
Non-alcoholic Fatty	C0400966	CASP3	836	P42574	caspase 3	Enzyme	819	0.351	0.923	0.10017	0.02
Non-alcoholic Fatty	C0400966	CASP8	841	Q14790	caspase 8	Enzyme	480	0.404	0.923	3.72E-06	0.02
Non-alcoholic Fatty	C0400966	ORAI1	84876	Q96D31	ORAI calc	Ion channe	195	0.52	0.808	1.91E-05	0.02
Non-alcoholic Fatty	C0400966	TNFSF11	8600	O14788	TNF super	Signaling	327	0.453	0.846	0.004916	0.02
Non-alcoholic Fatty	C0400966	SOCS1	8651	O15524	suppressor	Enzyme m	315	0.445	0.808	0.58544	0.02
Non-alcoholic Fatty	C0400966	CBR1	873	P16152	carbonyl reductase 1		100	0.572	0.654	6.28E-05	0.02
Non-alcoholic Fatty	C0400966	RIPK1	8737	Q13546	receptor in	Kinase	250	0.479	0.769	0.007944	0.02
Non-alcoholic Fatty	C0400966	CCN4	8840	O95388	cellular co	Signaling	114	0.554	0.692	6.39E-07	0.02
Non-alcoholic Fatty	C0400966	APLN	8862	Q9ULZ1	apelin		226	0.497	0.769	0.45897	0.02
Non-alcoholic Fatty	C0400966	SELENBP1	8991	Q13228	selenium b	Immune re	214	0.493	0.846	3.23E-16	0.02
Non-alcoholic Fatty	C0400966	SOCS3	9021	O14543	suppressor	Enzyme m	375	0.434	0.808	0.74613	0.02
Non-alcoholic Fatty	C0400966	USP10	9100	Q14694	ubiquitin s	Enzyme	40	0.663	0.462	0.99411	0.02
Non-alcoholic Fatty	C0400966	CD5L	922	O43866	CD5 mole	Enzyme	78	0.593	0.846	4.23E-17	0.02
Non-alcoholic Fatty	C0400966	CD163	9332	Q86VB7	CD163 mo	Enzyme	369	0.427	0.846	0.000834	0.02
Non-alcoholic Fatty	C0400966	PPIG	9360	Q13427	peptidylprolyl isomera		203	0.497	0.846	0.96292	0.02
Non-alcoholic Fatty	C0400966	CD68	968	P34810	CD68 mol	Transporte	452	0.408	0.808	1.41E-06	0.02
Non-alcoholic Fatty	C0400966	MIR873	#####		microRNA 873		67	0.601	0.538		0.01
Non-alcoholic Fatty	C0400966	MIR190B	#####		microRNA 190b		22	0.722	0.385		0.01
Non-alcoholic Fatty	C0400966	CD24	#####	P25063	CD24 molecule		244	0.472	0.769		0.01
Non-alcoholic Fatty	C0400966	MIR1224	#####		microRNA 1224		36	0.666	0.385		0.01
Non-alcoholic Fatty	C0400966	PARP2	10038	Q9UGN5	poly(ADP-ribose) pol		40	0.663	0.538	2.33E-13	0.01
Non-alcoholic Fatty	C0400966	LOC100505909	#####		histidine-rich glycopro		17	0.722	0.385		0.01
Non-alcoholic Fatty	C0400966	OCLN	#####	Q16625	occludin		195	0.507	0.846	8.56E-07	0.01
Non-alcoholic Fatty	C0400966	DNM1L	10059	O00429	dynamin 1	Enzyme m	273	0.475	0.808	0.000568	0.01
Non-alcoholic Fatty	C0400966	ERVK-2	#####		endogenous retrovirus		37	0.674	0.423		0.01
Non-alcoholic Fatty	C0400966	PARTICL	#####		promoter of MAT2A		9	0.792	0.269		0.01
Non-alcoholic Fatty	C0400966	PPIF	10105	P30405	peptidylprolyl isomera		78	0.593	0.692	0.001992	0.01
Non-alcoholic Fatty	C0400966	IFNL4	#####	K9M1U5	interferon lambda 4 (		39	0.67	0.577		0.01
Non-alcoholic Fatty	C0400966	LRPPRC	10128	P42704	leucine rich pentatrico		220	0.493	0.769	2.3E-10	0.01
Non-alcoholic Fatty	C0400966	SLC25A13	10165	Q9UJS0	solute carr	Transporte	109	0.617	0.5	1.28E-24	0.01
Non-alcoholic Fatty	C0400966	CDK4	1019	P11802	cyclin dep	Kinase	433	0.412	0.808	0.062909	0.01
Non-alcoholic Fatty	C0400966	B4GALT1-AS1	#####		B4GALT1 antisense		13	0.792	0.192		0.01
Non-alcoholic Fatty	C0400966	PRMT3	10196	O60678	protein arg	Epigenetic	17	0.743	0.423	4.9E-25	0.01
Non-alcoholic Fatty	C0400966	PLIN3	10226	O60664	perilipin 3	Transporte	14	0.736	0.423	1.18E-07	0.01

Non-alcoholic Fatty	C0400966	CDK8	1024	P49336	cyclin depe	Kinase	82	0.597	0.654	0.40964	0.01
Non-alcoholic Fatty	C0400966	FSTL3	10272	O95633	follistatin l	Enzyme m	33	0.695	0.423	0.68658	0.01
Non-alcoholic Fatty	C0400966	CDKN2A	1029	P42771;Q8N726	cyclin dependent kina		1314	0.3	0.885	0.39474	0.01
Non-alcoholic Fatty	C0400966	ZNF267	10308	Q14586	zinc finger protein 267		15	0.769	0.192	0.017108	0.01
Non-alcoholic Fatty	C0400966	AKR1A1	10327	P14550	aldo-keto r	Enzyme	185	0.51	0.846	6.66E-07	0.01
Non-alcoholic Fatty	C0400966	MERTK	10461	Q12866	MER proto	Kinase	154	0.531	0.692	2.66E-11	0.01
Non-alcoholic Fatty	C0400966	CAP1	10487	Q01518	cyclase ass	Cellular st	149	0.529	0.808	0.019817	0.01
Non-alcoholic Fatty	C0400966	STK25	10494	O00506	serine/thre	Kinase	29	0.7	0.346	0.051875	0.01
Non-alcoholic Fatty	C0400966	NCOA2	10499	Q15596	nuclear rec	Enzyme	95	0.575	0.615	1	0.01
Non-alcoholic Fatty	C0400966	ATG7	10533	O95352	autophagy	Enzyme	179	0.505	0.769	7.38E-12	0.01
Non-alcoholic Fatty	C0400966	LINC01684	#####		long intergenic non-pr		2	1	0.038		0.01
Non-alcoholic Fatty	C0400966	PRDX4	10549	Q13162	peroxiredo	Enzyme	59	0.619	0.692	0.026755	0.01
Non-alcoholic Fatty	C0400966	CEL	1056	P19835	carboxyl ester lipase		172	0.514	0.808	1.54E-06	0.01
Non-alcoholic Fatty	C0400966	SORBS1	10580	Q9BX66	sorbin and SH3 domai		124	0.554	0.769	1.53E-12	0.01
Non-alcoholic Fatty	C0400966	AHSA1	10598	O95433	activator of HSP90 AT		526	0.396	0.923	0.99896	0.01
Non-alcoholic Fatty	C0400966	ERLIN1	10613	O75477	ER lipid raft associate		33	0.736	0.308	0.17751	0.01
Non-alcoholic Fatty	C0400966	CAMKK2	10645	Q96RR4	calcium/ca	Kinase	62	0.617	0.654	0.091625	0.01
Non-alcoholic Fatty	C0400966	CXCR6	10663	O00574	C-X-C mo	G-protein c	325	0.448	0.885	5.72E-05	0.01
Non-alcoholic Fatty	C0400966	SIRT1-AS	#####		SIRT1 antisense RNA		44	0.67	0.615		0.01
Non-alcoholic Fatty	C0400966	TNFSF13B	10673	Q9Y275	TNF superfamily men		282	0.46	0.731	0.98193	0.01
Non-alcoholic Fatty	C0400966	EBP	10682	Q15125	EBP chole	Enzyme	243	0.494	0.846	0.93645	0.01
Non-alcoholic Fatty	C0400966	YME1L1	10730	Q96TA2	YME1 like	Enzyme	60	0.633	0.654	0.028988	0.01
Non-alcoholic Fatty	C0400966	PLK4	10733	O00444	polo like k	Kinase	135	0.551	0.731	7.08E-06	0.01
Non-alcoholic Fatty	C0400966	HPSE	10855	Q9Y251	heparanase	Enzyme	393	0.425	0.808	2.27E-14	0.01
Non-alcoholic Fatty	C0400966	PGRMC1	10857	O00264	progestero	Signaling	79	0.588	0.654	0.6711	0.01
Non-alcoholic Fatty	C0400966	NMU	10874	P48645	neuromedin U		68	0.61	0.808	0.000415	0.01
Non-alcoholic Fatty	C0400966	TMED2	10959	Q15363	transmemb	Transporte	73	0.61	0.692	0.89231	0.01
Non-alcoholic Fatty	C0400966	SLC27A2	11001	O14975	solute carri	Transporte	27	0.7	0.462	6.36E-09	0.01
Non-alcoholic Fatty	C0400966	LILRB4	11006	Q8NHJ6	leukocyte i	Receptor	46	0.659	0.423	4.71E-13	0.01
Non-alcoholic Fatty	C0400966	RIPK3	11035	Q9Y572	receptor in	Kinase	196	0.497	0.885	1.19E-14	0.01
Non-alcoholic Fatty	C0400966	LOC110806263	#####		TERT 5' regulatory re		245	0.464	0.731		0.01
Non-alcoholic Fatty	C0400966	ADAMTS13	11093	Q76LX8	ADAM me	Enzyme	229	0.496	0.769	5.14E-15	0.01
Non-alcoholic Fatty	C0400966	TREH	11181	O43280	trehalase		42	0.67	0.654	8.96E-15	0.01
Non-alcoholic Fatty	C0400966	LNCARSR	#####		lncRNA regulator of A		19	0.722	0.269		0.01
Non-alcoholic Fatty	C0400966	DUSP12	11266	Q9UNI6	dual specificity phosp		17	0.78	0.269	0.000139	0.01
Non-alcoholic Fatty	C0400966	CHRM3	1131	P20309	cholinergic	G-protein c	284	0.464	0.885	0.99253	0.01
Non-alcoholic Fatty	C0400966	VSIG4	11326	Q9Y279	V-set and immunoglol		51	0.628	0.615	1.5E-09	0.01
Non-alcoholic Fatty	C0400966	MGLL	11343	Q99685	monoglyce	Enzyme	125	0.564	0.731	0.020016	0.01
Non-alcoholic Fatty	C0400966	TXNRD3	#####	Q86VQ6	thioredoxin	Enzyme	37	0.653	0.615	8.34E-08	0.01

Non-alcoholic Fatty	C0400966	CHUK	1147	O15111	component	Kinase	125	0.556	0.731	0.99403	0.01
Non-alcoholic Fatty	C0400966	C1QTNF3	#####	Q9BXJ4	C1q and TNF related		26	0.695	0.577	1.81E-07	0.01
Non-alcoholic Fatty	C0400966	CIDEA	1149	O60543	cell death inducing DI		16	0.751	0.269	5.58E-06	0.01
Non-alcoholic Fatty	C0400966	H3P10	#####		H3 histone pseudogen		769	0.35	0.846		0.01
Non-alcoholic Fatty	C0400966	TWIST2	#####	Q8WVJ9	twist famil	Transcripti	198	0.518	0.731	0.56004	0.01
Non-alcoholic Fatty	C0400966	CLCN2	1181	P51788	chloride vo	Ion channe	67	0.626	0.462	5.55E-12	0.01
Non-alcoholic Fatty	C0400966	IFTAP	#####	Q86VG3	intraflagellar transport		2	0.931	0.154	7.61E-06	0.01
Non-alcoholic Fatty	C0400966	CYP2R1	#####	Q6VVX0	cytochrome P450 fam		139	0.563	0.731	3.79E-10	0.01
Non-alcoholic Fatty	C0400966	CPT1C	#####	Q8TCG5	carnitine pa	Enzyme	51	0.691	0.5	2.19E-09	0.01
Non-alcoholic Fatty	C0400966	CNR2	1269	P34972	cannabinoi	G-protein c	197	0.509	0.846	6.54E-06	0.01
Non-alcoholic Fatty	C0400966	COL3A1	1281	P02461	collagen type III alpha		301	0.477	0.808	1	0.01
Non-alcoholic Fatty	C0400966	SLC31A1	1317	O15431	solute carri	Transporte	71	0.612	0.577	0.065398	0.01
Non-alcoholic Fatty	C0400966	ADM	133	P35318	adrenomed	Signaling	405	0.436	0.808	0.040831	0.01
Non-alcoholic Fatty	C0400966	PPARGC1B	#####	Q86YN6	PPARG co	Transcripti	102	0.569	0.731	0.50321	0.01
Non-alcoholic Fatty	C0400966	NADK2	#####	Q4G0N4	NAD kinas	Kinase	42	0.691	0.346	0.065215	0.01
Non-alcoholic Fatty	C0400966	CP	1356	P00450	ceruloplasr	Enzyme	283	0.466	0.846	4.2E-10	0.01
Non-alcoholic Fatty	C0400966	CPN1	1369	P15169	carboxypeptidase N su		31	0.729	0.423	8.7E-08	0.01
Non-alcoholic Fatty	C0400966	CPOX	1371	P36551	coproporpl	Enzyme	246	0.488	0.846	0.17152	0.01
Non-alcoholic Fatty	C0400966	CPS1	1373	P31327	carbamoyl-	Enzyme	133	0.59	0.615	1.09E-09	0.01
Non-alcoholic Fatty	C0400966	CREB1	1385	P16220	cAMP responsive eler		294	0.463	0.885	0.99673	0.01
Non-alcoholic Fatty	C0400966	ATF2	1386	P15336	activating t	Transcripti	122	0.551	0.808	0.99131	0.01
Non-alcoholic Fatty	C0400966	CREBBP	1387	Q92793	CREB binding proteir		438	0.428	0.808	1	0.01
Non-alcoholic Fatty	C0400966	CRK	1398	P46108	CRK proto-oncogene,		544	0.394	0.923	0.95936	0.01
Non-alcoholic Fatty	C0400966	CRMP1	1400	Q14194	collapsin re	Enzyme	148	0.525	0.808	0.99173	0.01
Non-alcoholic Fatty	C0400966	MAPK14	1432	Q16539	mitogen-ac	Kinase	626	0.379	0.923	0.37466	0.01
Non-alcoholic Fatty	C0400966	FGFBP3	#####	Q8TAT2	fibroblast growth facto		8	0.821	0.154	0.001028	0.01
Non-alcoholic Fatty	C0400966	CSF3	1440	P09919	colony stimulating fac		687	0.377	0.885	0.38046	0.01
Non-alcoholic Fatty	C0400966	CSF3R	1441	Q99062	colony stin	Signaling	120	0.55	0.692	1.52E-10	0.01
Non-alcoholic Fatty	C0400966	SLC38A8	#####	A6NNN8	solute carri	Transporte	19	0.743	0.269	3.32E-24	0.01
Non-alcoholic Fatty	C0400966	ZFP90	#####	Q8TF47	ZFP90 zinc	Transcripti	13	0.78	0.231	0.031662	0.01
Non-alcoholic Fatty	C0400966	CILP2	#####	Q8IUL8	cartilage intermediate		15	0.76	0.346	8.03E-08	0.01
Non-alcoholic Fatty	C0400966	CTAA1	1483		cataract, anterior polar		106	0.568	0.731		0.01
Non-alcoholic Fatty	C0400966	HJV	#####	Q6ZVN8	hemojuvelin BMP co-		71	0.621	0.654	0.004236	0.01
Non-alcoholic Fatty	C0400966	CTF1	1489	Q16619	cardiotrophin 1		60	0.636	0.423	0.041899	0.01
Non-alcoholic Fatty	C0400966	CCN2	1490	P29279	cellular cor	Signaling	518	0.399	0.846	0.000502	0.01
Non-alcoholic Fatty	C0400966	PDIK1L	#####	Q8N165	PDLIM1 in	Kinase	138	0.534	0.769	0.26436	0.01
Non-alcoholic Fatty	C0400966	CTNNA1	1495	P35221	catenin alp	Cellular str	82	0.584	0.538	0.96962	0.01
Non-alcoholic Fatty	C0400966	SIK1	#####	A0A0B4J2F2;P57	salt inducil	Kinase	157	0.544	0.731	0.93716	0.01
Non-alcoholic Fatty	C0400966	HORMAD2	#####	Q8N7B1	HORMA domain cont		22	0.716	0.385	7.14E-05	0.01

Non-alcoholic Fatty	C0400966	CTRL	1506	P40313	chymotryp	Enzyme	76	0.599	0.846	1.1E-09	0.01
Non-alcoholic Fatty	C0400966	CTSB	1508	P07858	cathepsin I	Enzyme	304	0.457	0.846	5.88E-11	0.01
Non-alcoholic Fatty	C0400966	CTSD	1509	P07339	cathepsin I	Enzyme	242	0.478	0.846	0.000917	0.01
Non-alcoholic Fatty	C0400966	CTSG	1511	P08311	cathepsin (	Enzyme	95	0.563	0.731	9.56E-06	0.01
Non-alcoholic Fatty	C0400966	CTSS	1520	P25774	cathepsin S	Enzyme	131	0.539	0.731	0.34178	0.01
Non-alcoholic Fatty	C0400966	ADRB2	154	P07550	adrenocept	G-protein (	387	0.442	0.923	0.52545	0.01
Non-alcoholic Fatty	C0400966	SLC2A12	#####	Q8TD20	solute carri	Transporte	26	0.729	0.5	5.55E-05	0.01
Non-alcoholic Fatty	C0400966	CYP2A6	1548	P11509	cytochrome	P450 fam	133	0.556	0.731	5.4E-09	0.01
Non-alcoholic Fatty	C0400966	CYP2B6	1555	P20813	cytochrome	P450 fam	388	0.428	0.846	1.93E-10	0.01
Non-alcoholic Fatty	C0400966	CYP2B7P	1556		cytochrome	P450 fam	16	0.76	0.385		0.01
Non-alcoholic Fatty	C0400966	CYP2D7	1564	A0A087X1C5	cytochrome	P450 fam	42	0.659	0.577		0.01
Non-alcoholic Fatty	C0400966	CYP2J2	1573	P51589	cytochrome	P450 fam	65	0.628	0.615	1.62E-15	0.01
Non-alcoholic Fatty	C0400966	CYP4A11	1579	Q02928	cytochrome	Enzyme	34	0.695	0.462	1.09E-24	0.01
Non-alcoholic Fatty	C0400966	CYP8B1	1582	Q9UNU6	cytochrome	P450 fam	32	0.711	0.269	2.06E-05	0.01
Non-alcoholic Fatty	C0400966	CYP19A1	1588	P11511	cytochrome	Enzyme	519	0.41	0.885	1.7E-05	0.01
Non-alcoholic Fatty	C0400966	CYP27B1	1594	O15528	cytochrome	Enzyme	229	0.502	0.769	1.92E-07	0.01
Non-alcoholic Fatty	C0400966	CYP51A1	1595	Q16850	cytochrome	Enzyme	42	0.653	0.615	1.46E-07	0.01
Non-alcoholic Fatty	C0400966	DBP	1628	Q10586	D-box binc	Nucleic ac	105	0.556	0.731	0.23376	0.01
Non-alcoholic Fatty	C0400966	TMPRSS6	#####	Q8IU80	transmemb	Enzyme	113	0.584	0.654	4.14E-10	0.01
Non-alcoholic Fatty	C0400966	AEBP1	165	Q8IUX7	AE binding protein 1		69	0.631	0.654	7.35E-11	0.01
Non-alcoholic Fatty	C0400966	DDOST	1650	P39656	dolichyl-di	Enzyme	65	0.644	0.692	0.000614	0.01
Non-alcoholic Fatty	C0400966	TIMM8A	1678	O60220	translocase of inner m		200	0.51	0.846	0.65195	0.01
Non-alcoholic Fatty	C0400966	DHCR7	1717	Q9UBM7	7-dehydroc	Enzyme	266	0.5	0.846	3.95E-13	0.01
Non-alcoholic Fatty	C0400966	DIO3	1735	P55073	iodothyronine deiodin		80	0.593	0.731	0.62331	0.01
Non-alcoholic Fatty	C0400966	DMD	1756	P11532	dystrophin		484	0.423	0.923	1	0.01
Non-alcoholic Fatty	C0400966	DPYSL3	1809	Q14195	dihydropyr	Enzyme	52	0.628	0.462	0.99887	0.01
Non-alcoholic Fatty	C0400966	SLC26A3	1811	P40879	solute carri	Transporte	128	0.554	0.692	1.37E-13	0.01
Non-alcoholic Fatty	C0400966	JAG1	182	P78504	jagged canonical Notc		420	0.429	0.808	1	0.01
Non-alcoholic Fatty	C0400966	DSPP	1834	Q9NZW4	dentin sialophosphopr		114	0.556	0.769	1.12E-07	0.01
Non-alcoholic Fatty	C0400966	DUSP9	1852	Q99956	dual specificity phosp		26	0.705	0.308	0.81981	0.01
Non-alcoholic Fatty	C0400966	EDA	1896	Q92838	ectodysplasin A		150	0.553	0.808	0.97391	0.01
Non-alcoholic Fatty	C0400966	S1PR1	1901	P21453	sphingosin	G-protein (	126	0.546	0.769	0.73269	0.01
Non-alcoholic Fatty	C0400966	EEF1A1	1915	P68104	eukaryotic	Enzyme	88	0.584	0.654	0.97863	0.01
Non-alcoholic Fatty	C0400966	EGF	1950	P01133	epidermal growth fact		774	0.357	0.923	8.53E-17	0.01
Non-alcoholic Fatty	C0400966	EGR1	1958	P18146	early grow	Nucleic ac	378	0.433	0.923	0.3509	0.01
Non-alcoholic Fatty	C0400966	EIF4E	1977	P06730	eukaryotic	Nucleic ac	317	0.448	0.846	0.94133	0.01
Non-alcoholic Fatty	C0400966	A2M	2	P01023	alpha-2-ma	Enzyme m	147	0.529	0.769	4.52E-11	0.01
Non-alcoholic Fatty	C0400966	LINC01554	#####	Q52M75	long intergenic non-pr		4	0.931	0.077	0.076951	0.01
Non-alcoholic Fatty	C0400966	ENO3	2027	P13929	enolase 3	Enzyme	24	0.769	0.308	1.57E-19	0.01

Non-alcoholic Fatty	C0400966	EPHB2	2048	P29323	EPH recep	Kinase	649	0.374	0.846	0.99997	0.01
Non-alcoholic Fatty	C0400966	EPHB6	2051	O15197	EPH recep	Kinase	60	0.619	0.538	1.2E-07	0.01
Non-alcoholic Fatty	C0400966	EPO	2056	P01588	erythropoietin		646	0.381	0.885	0.01125	0.01
Non-alcoholic Fatty	C0400966	AKT2	208	P31751	AKT serin	Kinase	264	0.474	0.769	0.64432	0.01
Non-alcoholic Fatty	C0400966	ERN1	2081	O75460	endoplasm	Kinase	163	0.513	0.808	0.99027	0.01
Non-alcoholic Fatty	C0400966	ESRRB	2103	O95718	estrogen re	Nuclear re	102	0.565	0.846	0.12609	0.01
Non-alcoholic Fatty	C0400966	EZH2	2146	Q15910	enhancer o	Epigenetic	653	0.376	0.885	1	0.01
Non-alcoholic Fatty	C0400966	F2RL1	2150	P55085	F2R like tr	G-protein c	256	0.48	0.769	0.000148	0.01
Non-alcoholic Fatty	C0400966	FABP2	2169	P12104	fatty acid binding prot		65	0.608	0.654	0.004981	0.01
Non-alcoholic Fatty	C0400966	FABP5	2171	Q01469	fatty acid binding prot		85	0.585	0.731	0.047008	0.01
Non-alcoholic Fatty	C0400966	ACSL1	2180	P33121	acyl-CoA s	Enzyme	68	0.617	0.577	0.001532	0.01
Non-alcoholic Fatty	C0400966	PTK2B	2185	Q14289	protein tyro	Kinase	319	0.448	0.808	0.93536	0.01
Non-alcoholic Fatty	C0400966	UNC5B	#####	Q8IZJ1	unc-5 netri	Receptor	38	0.682	0.462	2.69E-07	0.01
Non-alcoholic Fatty	C0400966	FBN1	2200	P35555	fibrillin 1	Calcium-b	552	0.417	0.846	1	0.01
Non-alcoholic Fatty	C0400966	MARCHF8	#####	Q5T0T0	membrane associated		87	0.59	0.692	5.87E-07	0.01
Non-alcoholic Fatty	C0400966	FCGR3A	2214	P08637	Fc fragmer	Cell adhes	352	0.432	0.962	3.7E-06	0.01
Non-alcoholic Fatty	C0400966	FCGR3B	2215	O75015	Fc fragmer	Cell adhes	291	0.452	0.962	3.03E-07	0.01
Non-alcoholic Fatty	C0400966	JAZF1	#####	Q86VZ6	JAZF zinc finger 1		102	0.582	0.654	0.89582	0.01
Non-alcoholic Fatty	C0400966	GPC4	2239	O75487	glypican 4		181	0.547	0.769	0.92613	0.01
Non-alcoholic Fatty	C0400966	FGF1	2246	P05230	fibroblast g	Signaling	184	0.507	0.846	0.44406	0.01
Non-alcoholic Fatty	C0400966	FGR	2268	P09769	FGR proto	Kinase	56	0.647	0.615	0.48408	0.01
Non-alcoholic Fatty	C0400966	MLXIP	22877	Q9HAP2	MLX interacting prote		70	0.597	0.692	0.98932	0.01
Non-alcoholic Fatty	C0400966	ZHX2	22882	Q9Y6X8	zinc finger	Transcripti	172	0.51	0.654	0.03372	0.01
Non-alcoholic Fatty	C0400966	ALDOB	229	P05062	aldolase, fructose-bisp		80	0.623	0.538	7.88E-07	0.01
Non-alcoholic Fatty	C0400966	DKK1	22943	O94907	dickkopf	WNT signal	372	0.439	0.885	0.17423	0.01
Non-alcoholic Fatty	C0400966	PHLPP2	23035	Q6ZVD8	PH domain and leucin		45	0.641	0.346	1.21E-06	0.01
Non-alcoholic Fatty	C0400966	SARM1	23098	Q6SZW1	sterile alpha and TIR r		40	0.682	0.346	1.7E-10	0.01
Non-alcoholic Fatty	C0400966	AKR1B1	231	P15121	aldo-keto r	Enzyme	220	0.491	0.885	2.02E-05	0.01
Non-alcoholic Fatty	C0400966	MPRIP	23164	Q6WCQ1	myosin pho	Enzyme m	113	0.556	0.885	0.98078	0.01
Non-alcoholic Fatty	C0400966	MCF2L	23263	O15068	MCF.2 cel	Signaling	124	0.559	0.769	2.72E-07	0.01
Non-alcoholic Fatty	C0400966	CRTC1	23373	Q6UUV9	CREB regu	Transcripti	115	0.565	0.692	0.531	0.01
Non-alcoholic Fatty	C0400966	SIRT4	23409	Q9Y6E7	sirtuin 4	Epigenetic	74	0.603	0.654	6.86E-10	0.01
Non-alcoholic Fatty	C0400966	ANGPTL2	23452	Q9UKU9	angiopoieti	Signaling	125	0.545	0.731	0.001471	0.01
Non-alcoholic Fatty	C0400966	BRD4	23476	O60885	bromodom	Epigenetic	335	0.446	0.885	1	0.01
Non-alcoholic Fatty	C0400966	SRRM2	23524	Q9UQ35	serine/arginine repetit		101	0.564	0.692	1	0.01
Non-alcoholic Fatty	C0400966	MMD	23531	Q15546	monocyte t	Receptor	58	0.647	0.577	0.000134	0.01
Non-alcoholic Fatty	C0400966	FOSB	2354	P53539	FosB protc	Transcripti	278	0.463	0.846	0.97553	0.01
Non-alcoholic Fatty	C0400966	FOSL2	2355	P15408	FOS like 2	Transcripti	83	0.595	0.692	0.98293	0.01
Non-alcoholic Fatty	C0400966	DDAH1	23576	O94760	dimethylar	Enzyme	73	0.604	0.615	0.002797	0.01

Non-alcoholic Fatty	C0400966	DAPK2	23604	Q9UIK4	death assoc	Kinase	130	0.536	0.692	5.01E-10	0.01
Non-alcoholic Fatty	C0400966	MKRN1	23608	Q9UHC7	makorin ri	Enzyme	13	0.805	0.269	0.99421	0.01
Non-alcoholic Fatty	C0400966	TMEFF2	23671	Q9UIK5	transmemb	Enzyme m	63	0.619	0.5	7.39E-05	0.01
Non-alcoholic Fatty	C0400966	IL17RA	23765	Q96F46	interleukin	17 recepto	159	0.529	0.808	0.000356	0.01
Non-alcoholic Fatty	C0400966	ZBTB38	#####	Q8NAP3	zinc finger	Transcripti	60	0.663	0.615	0.99949	0.01
Non-alcoholic Fatty	C0400966	G6PC	2538	P35575	glucose-6-phosphatase		132	0.56	0.692	1.84E-05	0.01
Non-alcoholic Fatty	C0400966	PRDX5	25824	P30044	peroxiredoxin 5		153	0.522	0.808	0.000179	0.01
Non-alcoholic Fatty	C0400966	GALR1	2587	P47211	galanin rec	G-protein c	44	0.656	0.5	0.000532	0.01
Non-alcoholic Fatty	C0400966	RNF19A	25897	Q9NV58	ring finger	Enzyme	523	0.397	0.923	0.025305	0.01
Non-alcoholic Fatty	C0400966	WWTR1	25937	Q9GZV5	WW doma	Enzyme m	134	0.529	0.615	0.93914	0.01
Non-alcoholic Fatty	C0400966	TIPARP	25976	Q7Z3E1	TCDD inducible poly		25	0.711	0.346	0.99991	0.01
Non-alcoholic Fatty	C0400966	OSBPL3	26031	Q9H4L5	oxysterol binding prot		10	0.839	0.192	1.59E-06	0.01
Non-alcoholic Fatty	C0400966	APPL1	26060	Q9UKG1	adaptor protein, phosp		63	0.626	0.5	0.7952	0.01
Non-alcoholic Fatty	C0400966	POLDIP2	26073	Q9Y2S7	DNA polymerase delt		530	0.396	0.923	0.41407	0.01
Non-alcoholic Fatty	C0400966	TOR1AIP1	26092	Q5JTV8	torsin 1A interacting p		39	0.729	0.346	1.27E-09	0.01
Non-alcoholic Fatty	C0400966	PRPF31	26121	Q8WWY3	pre-mRNA	Nucleic ac	150	0.531	0.731	0.98313	0.01
Non-alcoholic Fatty	C0400966	TRPC4AP	26133	Q8TEL6	transient receptor pote		19	0.76	0.231	0.55209	0.01
Non-alcoholic Fatty	C0400966	AMD1	262	P17707	adenosylm	Enzyme	84	0.593	0.577	0.98227	0.01
Non-alcoholic Fatty	C0400966	FGF20	26281	Q9NP95	fibroblast g	Signaling	45	0.663	0.577	0.81379	0.01
Non-alcoholic Fatty	C0400966	AMD1P2	263		adenosylmethionine d		71	0.608	0.538		0.01
Non-alcoholic Fatty	C0400966	LATS2	26524	Q9NRM7	large tumo	Kinase	106	0.554	0.654	0.98807	0.01
Non-alcoholic Fatty	C0400966	AATF	26574	Q9NY61	apoptosis a	Transcripti	44	0.641	0.731	2.6E-12	0.01
Non-alcoholic Fatty	C0400966	GDNF	2668	P39905	glial cell d	Signaling	409	0.434	0.885	0.2035	0.01
Non-alcoholic Fatty	C0400966	NOX1	27035	Q9Y5S8	NADPH oxidase 1		142	0.536	0.808	8.66E-08	0.01
Non-alcoholic Fatty	C0400966	SIGLEC7	27036	Q9Y286	sialic acid	Receptor	137	0.535	0.846	2.39E-07	0.01
Non-alcoholic Fatty	C0400966	INTU	27152	Q9ULD6	inturned planar cell po		134	0.555	0.769	7.7E-10	0.01
Non-alcoholic Fatty	C0400966	NAAA	27163	Q02083	N-acyletha	Enzyme	90	0.579	0.692	2.07E-10	0.01
Non-alcoholic Fatty	C0400966	IL37	27178	Q9NZH6	interleukin 37		280	0.459	0.846	0.000123	0.01
Non-alcoholic Fatty	C0400966	GPC3	2719	P51654	glypican 3		311	0.466	0.808	0.9982	0.01
Non-alcoholic Fatty	C0400966	GLB1	2720	P16278	galactosida	Enzyme	385	0.448	0.808	7.92E-10	0.01
Non-alcoholic Fatty	C0400966	GCLC	2729	P48506	glutamate-	Enzyme	198	0.509	0.885	0.42718	0.01
Non-alcoholic Fatty	C0400966	RBMS3	27303	Q6XE24	RNA binding motif si		116	0.553	0.692	0.2128	0.01
Non-alcoholic Fatty	C0400966	GLI2	2736	P10070	GLI family zinc finge		351	0.446	0.846	0.97303	0.01
Non-alcoholic Fatty	C0400966	MAT2B	27430	Q9NZL9	methionine adenosyltr		48	0.644	0.5	0.046226	0.01
Non-alcoholic Fatty	C0400966	GLRX	2745	P35754	glutaredoxin		65	0.619	0.577	5.67E-07	0.01
Non-alcoholic Fatty	C0400966	GNA12	2768	Q03113	G protein s	Enzyme m	130	0.547	0.769	0.96994	0.01
Non-alcoholic Fatty	C0400966	GNAI3	2773	P08754	G protein s	Enzyme m	75	0.653	0.615	0.038287	0.01
Non-alcoholic Fatty	C0400966	GNAO1	2775	P09471	G protein s	Enzyme m	188	0.523	0.808	0.98963	0.01
Non-alcoholic Fatty	C0400966	ABO	28	P16442	ABO, alph	Enzyme	443	0.427	0.885		0.01

Non-alcoholic Fatty	C0400966	GPI	2821	P06744	glucose-6-		Enzyme	218	0.491	0.769	2.84E-06	0.01
Non-alcoholic Fatty	C0400966	ANGPT1	284	Q15389	angiopoieti	Signaling	340	0.446	0.808	0.95208	0.01
Non-alcoholic Fatty	C0400966	BTBD8	#####	Q5XKL5	BTB domain containi		125	0.547	0.692	6.08E-07	0.01
Non-alcoholic Fatty	C0400966	ANGPT2	285	O15123	angiopoieti	Signaling	457	0.41	0.846	0.83208	0.01
Non-alcoholic Fatty	C0400966	GPR31	2853	O00270	G protein-c	G-protein c	7	0.839	0.115	0.020514	0.01
Non-alcoholic Fatty	C0400966	FFAR2	2867	O15552	free fatty a	G-protein c	45	0.686	0.462	0.009424	0.01
Non-alcoholic Fatty	C0400966	GPS2	2874	Q13227	G protein p	Enzyme m	25	0.691	0.346	0.053179	0.01
Non-alcoholic Fatty	C0400966	MYLIP	29116	Q8WY64	myosin regulatory ligh		79	0.582	0.692	0.18431	0.01
Non-alcoholic Fatty	C0400966	CTNNA3	29119	Q9UI47	catenin alp	Cellular str	73	0.612	0.692	1.02E-12	0.01
Non-alcoholic Fatty	C0400966	GSK3B	2932	P49841	glycogen s	Kinase	393	0.43	0.846	0.95571	0.01
Non-alcoholic Fatty	C0400966	GSTM2	2946	P28161	glutathione S-transfera		195	0.488	0.808	3.39E-07	0.01
Non-alcoholic Fatty	C0400966	REPIN1	29803	Q9BWE0	replication	Transcripti	13	0.792	0.231	0.09473	0.01
Non-alcoholic Fatty	C0400966	CERS2	29956	Q96G23	ceramide synthase 2		40	0.691	0.5	0.47978	0.01
Non-alcoholic Fatty	C0400966	DMGDH	29958	Q9UI17	dimethylgl	Enzyme	15	0.89	0.154	2.05E-24	0.01
Non-alcoholic Fatty	C0400966	SLC2A8	29988	Q9NY64	solute carri	Transporte	10	0.821	0.346	8.02E-07	0.01
Non-alcoholic Fatty	C0400966	SLC40A1	30061	Q9NP59	solute carri	Transporte	101	0.57	0.731	0.98835	0.01
Non-alcoholic Fatty	C0400966	H2AX	3014	P16104	H2A.X vai	Nucleic ac	212	0.484	0.808	0.17652	0.01
Non-alcoholic Fatty	C0400966	HADHB	3032	P55084	hydroxyacy	Enzyme	101	0.619	0.692	1.52E-11	0.01
Non-alcoholic Fatty	C0400966	HADH	3033	Q16836	hydroxyacyl-CoA deh		72	0.65	0.538	6.56E-05	0.01
Non-alcoholic Fatty	C0400966	HCFC1	3054	P51610	host cell factor C1		100	0.601	0.692	1	0.01
Non-alcoholic Fatty	C0400966	HLA-A	3105	P04439	major histocompatibil		672	0.37	0.846	0.00133	0.01
Non-alcoholic Fatty	C0400966	HLA-C	3107	P10321	major histocompatibil		435	0.415	0.846	1.57E-06	0.01
Non-alcoholic Fatty	C0400966	HLA-DQA1	3117	P01909	major histo	Immune re	427	0.416	0.846	0.023947	0.01
Non-alcoholic Fatty	C0400966	HLA-DQB1	3119	P01920	major histo	Immune re	504	0.407	0.885	0.008926	0.01
Non-alcoholic Fatty	C0400966	HLA-DQB2	3120	P05538	major histo	Immune re	91	0.573	0.731	0.042679	0.01
Non-alcoholic Fatty	C0400966	HLA-DRB1	3123	P01911	major histo	Immune re	1018	0.333	0.923	0.0011	0.01
Non-alcoholic Fatty	C0400966	AOX1	316	Q06278	aldehyde o	Enzyme	26	0.716	0.308	1.17E-31	0.01
Non-alcoholic Fatty	C0400966	NR4A1	3164	P22736	nuclear rec	Nuclear re	216	0.49	0.731	0.37074	0.01
Non-alcoholic Fatty	C0400966	FOXA2	3170	Q9Y261	forkhead b	Transcripti	143	0.528	0.769	0.74225	0.01
Non-alcoholic Fatty	C0400966	BPIFA4P	#####	Q86YQ2	BPI fold containing fa		32	0.711	0.654		0.01
Non-alcoholic Fatty	C0400966	APOF	319	Q13790	apolipoprotein F		9	0.805	0.231	0.010295	0.01
Non-alcoholic Fatty	C0400966	HOXD13	3239	P35453	homeobox D13		259	0.483	0.808	0.008606	0.01
Non-alcoholic Fatty	C0400966	AIRE	326	O43918	autoimmune regulator		175	0.516	0.808	4.05E-12	0.01
Non-alcoholic Fatty	C0400966	HRG	3273	P04196	histidine ri	Enzyme m	72	0.603	0.577	5.89E-14	0.01
Non-alcoholic Fatty	C0400966	PRMT1	3276	Q99873	protein arg	Epigenetic	110	0.552	0.808	0.99907	0.01
Non-alcoholic Fatty	C0400966	HSPA4	3308	P34932	heat shock protein fan		550	0.394	0.923	0.99954	0.01
Non-alcoholic Fatty	C0400966	HSPA5	3309	P11021	heat shock protein fan		350	0.434	0.885	0.77341	0.01
Non-alcoholic Fatty	C0400966	XIAP	331	P98170	X-linked ir	Enzyme m	321	0.447	0.808	0.91837	0.01
Non-alcoholic Fatty	C0400966	HSPA8	3312	P11142	heat shock protein fan		124	0.553	0.808	0.99863	0.01

Non-alcoholic Fatty	C0400966	HSPG2	3339	P98160	heparan su	Enzyme m	405	0.438	0.885	1.04E-19	0.01
Non-alcoholic Fatty	C0400966	APOA1	335	P02647	apolipoprotein A1		416	0.429	0.885	0.000549	0.01
Non-alcoholic Fatty	C0400966	HTR2A	3356	P28223	5-hydroxyt	G-protein (	289	0.473	0.846	0.51124	0.01
Non-alcoholic Fatty	C0400966	APOA2	336	P02652	apolipopro	Transporte	77	0.582	0.769	0.0448	0.01
Non-alcoholic Fatty	C0400966	TNC	3371	P24821	tenascin C		369	0.433	0.846	1.26E-07	0.01
Non-alcoholic Fatty	C0400966	HCAR2	#####	Q8TDS4	hydroxycarboxylic aci		65	0.631	0.692	0.000189	0.01
Non-alcoholic Fatty	C0400966	C1QTNF9	#####	P0C862	C1q and TNF related		30	0.695	0.385	1.14E-05	0.01
Non-alcoholic Fatty	C0400966	STING1	#####	Q86WV6	stimulator of interfero		159	0.522	0.769	0.001207	0.01
Non-alcoholic Fatty	C0400966	CPP	#####		ceruloplasmin pseudo		55	0.631	0.731		0.01
Non-alcoholic Fatty	C0400966	IFNA1	3439	P01562	interferon alpha 1		662	0.371	0.923		0.01
Non-alcoholic Fatty	C0400966	IFNA2	3440	P01563	interferon alpha 2		355	0.442	0.885		0.01
Non-alcoholic Fatty	C0400966	IFNA13	3447	P01562	interferon alpha 13		646	0.374	0.923		0.01
Non-alcoholic Fatty	C0400966	MOGAT3	#####	Q86VF5	monoacylglycerol O-a		2	0.931	0.115	1.84E-12	0.01
Non-alcoholic Fatty	C0400966	IGF2	3481	P01344	insulin like growth fac		604	0.39	0.885	0.044127	0.01
Non-alcoholic Fatty	C0400966	IGFBP1	3484	P08833	insulin like	Enzyme m	191	0.502	0.808	0.000947	0.01
Non-alcoholic Fatty	C0400966	IGFBP2	3485	P18065	insulin like	Enzyme m	241	0.478	0.769	0.11898	0.01
Non-alcoholic Fatty	C0400966	IGFBP4	3487	P22692	insulin like	Enzyme m	94	0.572	0.731	0.023057	0.01
Non-alcoholic Fatty	C0400966	IGFBP7	3490	Q16270	insulin like growth fac		241	0.476	0.769	0.001287	0.01
Non-alcoholic Fatty	C0400966	KLK3	354	P07288	kallikrein r	Enzyme	358	0.443	0.885	4.43E-06	0.01
Non-alcoholic Fatty	C0400966	IKBKB	3551	O14920	inhibitor of	Kinase	191	0.506	0.846	0.99192	0.01
Non-alcoholic Fatty	C0400966	IL2	3558	P60568	interleukin 2		950	0.336	0.885	0.47968	0.01
Non-alcoholic Fatty	C0400966	CXCR2	3579	P25025	C-X-C mot	G-protein (	335	0.445	0.923	0.013736	0.01
Non-alcoholic Fatty	C0400966	IRF2BP2	#####	Q7Z5L9	interferon regulatory f		72	0.641	0.5	0.84558	0.01
Non-alcoholic Fatty	C0400966	INPP5D	3635	Q92835	inositol polyphosphate		109	0.558	0.731	0.97198	0.01
Non-alcoholic Fatty	C0400966	INSIG1	3638	O15503	insulin induced gene 1		29	0.695	0.5	0.001283	0.01
Non-alcoholic Fatty	C0400966	AQP7	364	O14520	aquaporin 7	Ion channe	34	0.67	0.462	4.05E-16	0.01
Non-alcoholic Fatty	C0400966	IRF3	3661	Q14653	interferon r	Transcripti	174	0.517	0.808	0.000116	0.01
Non-alcoholic Fatty	C0400966	ITGAM	3684	P11215	integrin subunit alpha		343	0.443	0.808	3.31E-11	0.01
Non-alcoholic Fatty	C0400966	ITGB1	3688	P05556	integrin su	Receptor	210	0.496	0.808	0.98208	0.01
Non-alcoholic Fatty	C0400966	ITIH4	3700	Q14624	inter-alpha	Enzyme m	129	0.559	0.769	6.44E-13	0.01
Non-alcoholic Fatty	C0400966	ITPR2	3709	Q14571	inositol 1,4	Ion channe	55	0.631	0.654	5.71E-29	0.01
Non-alcoholic Fatty	C0400966	JAK1	3716	P23458	Janus kinas	Kinase	239	0.474	0.769	0.99998	0.01
Non-alcoholic Fatty	C0400966	JUN	3725	P05412	Jun proto-c	Transcripti	344	0.442	0.885	0.05962	0.01
Non-alcoholic Fatty	C0400966	JUNB	3726	P17275	JunB proto	Transcripti	264	0.467	0.769	0.86299	0.01
Non-alcoholic Fatty	C0400966	JUND	3727	P17535	JunD proto	Transcripti	242	0.475	0.808	0.70054	0.01
Non-alcoholic Fatty	C0400966	CD82	3732	P27701	CD82 molecule		172	0.507	0.731	1.27E-08	0.01
Non-alcoholic Fatty	C0400966	AREG	374	P15514	amphiregu	Signaling	221	0.487	0.769	0.35407	0.01
Non-alcoholic Fatty	C0400966	ACAT1	38	P24752	acetyl-CoA	Enzyme	176	0.538	0.692	9.85E-08	0.01
Non-alcoholic Fatty	C0400966	ARG2	384	P78540	arginase 2	Enzyme	103	0.569	0.731	0.000182	0.01

Non-alcoholic Fatty	C0400966	KRT8	3856	P05787	keratin 8	166	0.517	0.654	2.36E-08	0.01
Non-alcoholic Fatty	C0400966	RTL1	#####	A6NKG5	retrotransposon Gag li	119	0.554	0.808	0.005614	0.01
Non-alcoholic Fatty	C0400966	C1QL3	#####	Q5VWW1	complement C1q like	27	0.705	0.423	0.51567	0.01
Non-alcoholic Fatty	C0400966	LCAT	3931	P04180	lecithin-ch Enzyme	94	0.588	0.654	0.08102	0.01
Non-alcoholic Fatty	C0400966	LCP1	3936	P13796	lymphocyt Cellular st	44	0.663	0.654	0.55261	0.01
Non-alcoholic Fatty	C0400966	LGALS3	3958	P17931	galectin 3 Signaling	557	0.392	0.846	2.32E-05	0.01
Non-alcoholic Fatty	C0400966	LIPE	3991	Q05469	lipase E, hormone sen	85	0.599	0.5	9.75E-12	0.01
Non-alcoholic Fatty	C0400966	FADS1	3992	O60427	fatty acid desaturase 1	125	0.582	0.769	0.37384	0.01
Non-alcoholic Fatty	C0400966	LNPEP	4012	Q9UIQ6	leucyl and Enzyme	174	0.514	0.731	0.99957	0.01
Non-alcoholic Fatty	C0400966	LOXL2	4017	Q9Y4K0	lysyl oxidase like 2	149	0.527	0.731	3.38E-07	0.01
Non-alcoholic Fatty	C0400966	LRP6	4040	O75581	LDL receptor related	134	0.536	0.808	0.69664	0.01
Non-alcoholic Fatty	C0400966	LINC01194	#####		long intergenic non-pr	249	0.47	0.769		0.01
Non-alcoholic Fatty	C0400966	ARNT	405	P27540	aryl hydroc Transcripti	104	0.575	0.692	0.99581	0.01
Non-alcoholic Fatty	C0400966	LTF	4057	P02788	lactotransf Enzyme	133	0.543	0.846	1.27E-21	0.01
Non-alcoholic Fatty	C0400966	LUM	4060	P51884	lumican	123	0.543	0.731	0.07696	0.01
Non-alcoholic Fatty	C0400966	MIR130A	#####		microRNA 130a	141	0.522	0.808		0.01
Non-alcoholic Fatty	C0400966	MIR132	#####		microRNA 132	222	0.485	0.846		0.01
Non-alcoholic Fatty	C0400966	MIR136	#####		microRNA 136	79	0.584	0.731		0.01
Non-alcoholic Fatty	C0400966	MIR141	#####		microRNA 141	179	0.497	0.808		0.01
Non-alcoholic Fatty	C0400966	MIR142	#####		microRNA 142	220	0.48	0.846		0.01
Non-alcoholic Fatty	C0400966	MIR143	#####		microRNA 143	267	0.462	0.885		0.01
Non-alcoholic Fatty	C0400966	MIR144	#####		microRNA 144	175	0.5	0.769		0.01
Non-alcoholic Fatty	C0400966	MIR146A	#####		microRNA 146a	505	0.398	0.885		0.01
Non-alcoholic Fatty	C0400966	MIR150	#####		microRNA 150	282	0.455	0.808		0.01
Non-alcoholic Fatty	C0400966	MIR15B	#####		microRNA 15b	134	0.532	0.769		0.01
Non-alcoholic Fatty	C0400966	MIR17	#####		microRNA 17	270	0.459	0.846		0.01
Non-alcoholic Fatty	C0400966	MIR185	#####		microRNA 185	106	0.551	0.808		0.01
Non-alcoholic Fatty	C0400966	MIR200B	#####		microRNA 200b	180	0.5	0.769		0.01
Non-alcoholic Fatty	C0400966	MIR205	#####		microRNA 205	210	0.482	0.846		0.01
Non-alcoholic Fatty	C0400966	MIR206	#####		microRNA 206	226	0.477	0.846		0.01
Non-alcoholic Fatty	C0400966	MIR212	#####		microRNA 212	126	0.535	0.808		0.01
Non-alcoholic Fatty	C0400966	MIR223	#####		microRNA 223	348	0.433	0.808		0.01
Non-alcoholic Fatty	C0400966	MIR23B	#####		microRNA 23b	137	0.523	0.808		0.01
Non-alcoholic Fatty	C0400966	MIR24-1	#####		microRNA 24-1	74	0.584	0.731		0.01
Non-alcoholic Fatty	C0400966	MIR27A	#####		microRNA 27a	248	0.466	0.846		0.01
Non-alcoholic Fatty	C0400966	MIR27B	#####		microRNA 27b	141	0.529	0.769		0.01
Non-alcoholic Fatty	C0400966	MIR29B1	#####		microRNA 29b-1	212	0.485	0.846		0.01
Non-alcoholic Fatty	C0400966	MIR29B2	#####		microRNA 29b-2	214	0.484	0.885		0.01
Non-alcoholic Fatty	C0400966	MIR29C	#####		microRNA 29c	183	0.496	0.808		0.01

Non-alcoholic Fatty	C0400966	MIR30B	#####		microRNA 30b		109	0.555	0.808		0.01
Non-alcoholic Fatty	C0400966	MIR30C1	#####		microRNA 30c-1		109	0.552	0.769		0.01
Non-alcoholic Fatty	C0400966	MIR30C2	#####		microRNA 30c-2		115	0.546	0.769		0.01
Non-alcoholic Fatty	C0400966	ARRB1	408	P49407	arrestin bet	Enzyme m	92	0.588	0.846	0.99394	0.01
Non-alcoholic Fatty	C0400966	SMAD7	4092	O15105	SMAD fan	Transcripti	269	0.47	0.769	0.54854	0.01
Non-alcoholic Fatty	C0400966	MAT1A	4143	Q00266	methionine	Enzyme	103	0.582	0.577	0.00081	0.01
Non-alcoholic Fatty	C0400966	DNAJB9	4189	Q9UBS3	DnaJ heat shock prote		27	0.7	0.385	0.36507	0.01
Non-alcoholic Fatty	C0400966	MEFV	4210	O15553	MEFV innate immuity		410	0.44	0.885	1.02E-14	0.01
Non-alcoholic Fatty	C0400966	MET	4233	P08581	MET protc	Kinase	594	0.38	0.846	0.96998	0.01
Non-alcoholic Fatty	C0400966	MIF	4282	P14174	macrophage migration		455	0.412	0.885	0.012262	0.01
Non-alcoholic Fatty	C0400966	CXCL9	4283	Q07325	C-X-C mo	Signaling	269	0.462	0.808	0.00293	0.01
Non-alcoholic Fatty	C0400966	MMP9	4318	P14780	matrix met	Enzyme	1337	0.305	0.923	1.89E-17	0.01
Non-alcoholic Fatty	C0400966	MPI	4351	P34949	mannose p	Enzyme	101	0.585	0.654	1.17E-07	0.01
Non-alcoholic Fatty	C0400966	MPO	4353	P05164	myeloperox	Enzyme	653	0.377	0.923	7.11E-15	0.01
Non-alcoholic Fatty	C0400966	MPST	4357	P25325	mercaptopy	Enzyme	81	0.588	0.654	3.47E-05	0.01
Non-alcoholic Fatty	C0400966	MRC1	4360	P22897	mannose receptor C-ty		431	0.413	0.846	0.94335	0.01
Non-alcoholic Fatty	C0400966	ASNS	440	P08243	asparagine	Enzyme	90	0.595	0.692	9.36E-06	0.01
Non-alcoholic Fatty	C0400966	EIF2AK4	#####	Q9P2K8	eukaryotic translation		89	0.603	0.577	1.24E-15	0.01
Non-alcoholic Fatty	C0400966	MIR330	#####		microRNA 330		81	0.584	0.692		0.01
Non-alcoholic Fatty	C0400966	MIR331	#####		microRNA 331		63	0.612	0.615		0.01
Non-alcoholic Fatty	C0400966	MIR367	#####		microRNA 367		71	0.59	0.654		0.01
Non-alcoholic Fatty	C0400966	MST1	4485	P26927	macrophag	Enzyme	281	0.465	0.885	4.78E-19	0.01
Non-alcoholic Fatty	C0400966	ND6	4541	P03923	NADH dehydrogenase		224	0.544	0.808		0.01
Non-alcoholic Fatty	C0400966	MYC	4609	P01106	MYC protc	Transcripti	821	0.344	0.923	0.99801	0.01
Non-alcoholic Fatty	C0400966	MYD88	4615	Q99836	MYD88 in	Enzyme m	480	0.414	0.923	0.12443	0.01
Non-alcoholic Fatty	C0400966	HNRNPM	4670	P52272	heterogene	Nucleic ac	26	0.729	0.346	0.99999	0.01
Non-alcoholic Fatty	C0400966	ATF4	468	P18848	activating transcription		178	0.507	0.769	0.015659	0.01
Non-alcoholic Fatty	C0400966	NBN	4683	O60934	nibrin	Nucleic ac	291	0.467	0.808	2.18E-16	0.01
Non-alcoholic Fatty	C0400966	NEU1	4758	Q99519	neuraminic	Enzyme	207	0.513	0.769	7.34E-05	0.01
Non-alcoholic Fatty	C0400966	NHS	4810	Q6T4R5	NHS actin remodeling		295	0.466	0.846	0.99999	0.01
Non-alcoholic Fatty	C0400966	NNMT	4837	P40261	nicotinami	Enzyme	103	0.559	0.654	5.28E-08	0.01
Non-alcoholic Fatty	C0400966	NOS2	4843	P35228	nitric oxide synthase 2		783	0.364	0.923	4.03E-15	0.01
Non-alcoholic Fatty	C0400966	NOS3	4846	P29474	nitric oxide synthase 3		706	0.378	0.885	6.72E-07	0.01
Non-alcoholic Fatty	C0400966	NOTCH1	4851	P46531	notch receptor 1		693	0.369	0.885	1	0.01
Non-alcoholic Fatty	C0400966	NRF1	4899	Q16656	nuclear respiratory fac		74	0.603	0.808	0.99922	0.01
Non-alcoholic Fatty	C0400966	NUCB2	4925	P80303	nucleobind	Calcium-b	140	0.54	0.731	2E-10	0.01
Non-alcoholic Fatty	C0400966	CISD2	#####	Q8N5K1	CDGSH iron sulfur dc		106	0.59	0.692	0.41583	0.01
Non-alcoholic Fatty	C0400966	MIR361	#####		microRNA 361		79	0.578	0.654		0.01
Non-alcoholic Fatty	C0400966	MIR423	#####		microRNA 423		91	0.566	0.769		0.01

Non-alcoholic Fatty	C0400966	P2RX5	5026	Q93086	purinergic	Ion channe	132	0.548	0.769	2.85E-13	0.01
Non-alcoholic Fatty	C0400966	P4HA1	5033	P13674	prolyl 4-hydroxylase s		27	0.736	0.385	0.93166	0.01
Non-alcoholic Fatty	C0400966	DUOX2	50506	Q9NRD8	dual oxidas	Enzyme	150	0.533	0.769	4.5E-56	0.01
Non-alcoholic Fatty	C0400966	NOX4	50507	Q9NPH5	NADPH o:	Enzyme	260	0.471	0.885	5.76E-20	0.01
Non-alcoholic Fatty	C0400966	IL20	50604	Q9NYY1	interleukin 20		111	0.556	0.769	1.34E-06	0.01
Non-alcoholic Fatty	C0400966	PAPPA	5069	Q13219	pappalysin 1		125	0.558	0.692	0.1613	0.01
Non-alcoholic Fatty	C0400966	PC	5091	P11498	pyruvate ca	Enzyme	183	0.515	0.808	0.013834	0.01
Non-alcoholic Fatty	C0400966	SOST	50964	Q9BQB4	sclerostin		270	0.474	0.808	0.86731	0.01
Non-alcoholic Fatty	C0400966	PCK1	5105	P35558	phosphoen	Kinase	73	0.608	0.538	8.75E-15	0.01
Non-alcoholic Fatty	C0400966	ABHD5	51099	Q8WTS1	abhydrolase domain c		76	0.641	0.538	6.42E-07	0.01
Non-alcoholic Fatty	C0400966	INSIG2	51141	Q9Y5U4	insulin induced gene 2		45	0.653	0.5	0.059986	0.01
Non-alcoholic Fatty	C0400966	MBTPS2	51360	O43462	membrane	Enzyme	205	0.513	0.769	0.99752	0.01
Non-alcoholic Fatty	C0400966	ISYNA1	51477	Q9NPH2	inositol-3-phosphate s		168	0.51	0.769	2.49E-05	0.01
Non-alcoholic Fatty	C0400966	SIRT6	51548	Q8N6T7	sirtuin 6	Epigenetic	195	0.496	0.846	0.039119	0.01
Non-alcoholic Fatty	C0400966	PDGFRB	5159	P09619	platelet de	Kinase	492	0.416	0.846	0.9041	0.01
Non-alcoholic Fatty	C0400966	ARID4B	51742	Q4LE39	AT-rich in	Transcripti	62	0.631	0.615	1	0.01
Non-alcoholic Fatty	C0400966	SERPINA4	5267	P29622	serpin fam	Enzyme m	45	0.641	0.654	9.73E-09	0.01
Non-alcoholic Fatty	C0400966	SERPINB6	5269	P35237	serpin fam	Enzyme m	128	0.547	0.769	0.00011	0.01
Non-alcoholic Fatty	C0400966	PIGR	5284	P01833	polymeric immunoglo		71	0.615	0.808	0.81331	0.01
Non-alcoholic Fatty	C0400966	PITX3	5309	O75364	paired like homeodom		93	0.585	0.692	0.81276	0.01
Non-alcoholic Fatty	C0400966	PKM	5315	P14618	pyruvate ki	Kinase	237	0.482	0.808	0.8549	0.01
Non-alcoholic Fatty	C0400966	PKNOX1	5316	P55347	PBX/knott	Enzyme	34	0.674	0.538	0.99401	0.01
Non-alcoholic Fatty	C0400966	PLCG1	5335	P19174	phospholip	Enzyme	116	0.564	0.692	7.93E-05	0.01
Non-alcoholic Fatty	C0400966	PLG	5340	P00747	plasminoge	Enzyme	586	0.389	0.923	0.010345	0.01
Non-alcoholic Fatty	C0400966	PLTP	5360	P55058	phospholipid transfer		62	0.633	0.654	9E-09	0.01
Non-alcoholic Fatty	C0400966	PMCH	5367	P20382	pro-melanin concentra		57	0.615	0.808	0.11486	0.01
Non-alcoholic Fatty	C0400966	DUOX1	53905	Q9NRD9	dual oxidas	Enzyme	97	0.57	0.692	6.22E-33	0.01
Non-alcoholic Fatty	C0400966	TREM1	54210	Q9NP99	triggering receptor ex		172	0.515	0.808	0.006278	0.01
Non-alcoholic Fatty	C0400966	CCHCR1	54535	Q8TD31	coiled-coil alpha-helic		193	0.505	0.808	1.01E-16	0.01
Non-alcoholic Fatty	C0400966	UGT1A10	54575	Q9HAW8	UDP glucuronosyltran		88	0.588	0.615	4.59E-19	0.01
Non-alcoholic Fatty	C0400966	UGT1A8	54576	Q9HAW9	UDP glucuronosyltran		90	0.588	0.654	1.31E-09	0.01
Non-alcoholic Fatty	C0400966	UGT1A7	54577	Q9HAW7	UDP glucuronosyltran		125	0.55	0.692	8.66E-14	0.01
Non-alcoholic Fatty	C0400966	UGT1A6	54578	P19224	UDP glucuronosyltran		115	0.56	0.654	3.05E-07	0.01
Non-alcoholic Fatty	C0400966	EGLN1	54583	Q9GZT9	egl-9 family hypoxia i		130	0.54	0.731	0.97403	0.01
Non-alcoholic Fatty	C0400966	UGT1A4	54657	P22310	UDP glucuronosyltran		84	0.595	0.654	5.98E-07	0.01
Non-alcoholic Fatty	C0400966	CROT	54677	Q9UKG9	carnitine C	Enzyme	11	0.805	0.346	4.43E-25	0.01
Non-alcoholic Fatty	C0400966	TET2	54790	Q6N021	tet methylcytosine dio		362	0.458	0.808	4.33E-54	0.01
Non-alcoholic Fatty	C0400966	SLC52A1	55065	Q9NWF4	solute carri	Transporte	160	0.526	0.769	3.52E-06	0.01
Non-alcoholic Fatty	C0400966	PPP1R3C	5507	Q9UQK1	protein pho	Enzyme m	25	0.711	0.5	0.00264	0.01

Non-alcoholic Fatty	C0400966	PIWIL2	55124	Q8TC59	piwi like RNA-mediat		65	0.612	0.577	2.71E-08	0.01
Non-alcoholic Fatty	C0400966	NUDT11	55190	Q96G61	nudix hydr	Enzyme	37	0.647	0.615	0.10316	0.01
Non-alcoholic Fatty	C0400966	APPL2	55198	Q8NEU8	adaptor protein, phosp		36	0.7	0.423	1.16E-18	0.01
Non-alcoholic Fatty	C0400966	SLC47A1	55244	Q96FL8	solute carri	Transporte	28	0.729	0.462	1.96E-14	0.01
Non-alcoholic Fatty	C0400966	CWF19L1	55280	Q69YN2	CWF19 like cell cycle		51	0.691	0.346	1.44E-15	0.01
Non-alcoholic Fatty	C0400966	FBXW7	55294	Q969H0	F-box and	Enzyme m	257	0.465	0.808	0.99949	0.01
Non-alcoholic Fatty	C0400966	CHDH	55349	Q8NE62	choline del	Enzyme	158	0.529	0.769	1.69E-06	0.01
Non-alcoholic Fatty	C0400966	NLRP2	55655	Q9NX02	NLR family pyrin don		155	0.515	0.731	1.42E-38	0.01
Non-alcoholic Fatty	C0400966	TRERF1	55809	Q96PN7	transcriptional regulat		22	0.729	0.346	1	0.01
Non-alcoholic Fatty	C0400966	ALLC	55821	Q8N6M5	allantoicase		28	0.695	0.385	4.23E-16	0.01
Non-alcoholic Fatty	C0400966	PAG1	55824	Q9NWQ8	phosphoprotein memb		174	0.505	0.808	0.017764	0.01
Non-alcoholic Fatty	C0400966	ACOT13	55856	Q9NPJ3	acyl-CoA thioesterase		17	0.729	0.423	3.13E-06	0.01
Non-alcoholic Fatty	C0400966	PRKDC	5591	P78527	protein kin	Kinase	131	0.54	0.808	1	0.01
Non-alcoholic Fatty	C0400966	ERBIN	55914	Q96RT1	erbb2 interacting prot		31	0.7	0.231	0.99402	0.01
Non-alcoholic Fatty	C0400966	MAPK3	5595	P27361	mitogen-ac	Kinase	647	0.379	0.885	0.036884	0.01
Non-alcoholic Fatty	C0400966	MAP2K7	5609	O14733	mitogen-ac	Kinase	408	0.419	0.808	0.99552	0.01
Non-alcoholic Fatty	C0400966	PRLR	5618	P16471	prolactin re	Signaling	110	0.572	0.692	0.98732	0.01
Non-alcoholic Fatty	C0400966	LGALS14	56891	Q8TCE9	galectin 14	Signaling	35	0.695	0.423	0.000275	0.01
Non-alcoholic Fatty	C0400966	UBQLN4	56893	Q9NRR5	ubiquilin 4		11	0.805	0.308	0.98252	0.01
Non-alcoholic Fatty	C0400966	PNO1	56902	Q9NRX1	partner of NOB1 hom		99	0.564	0.615	0.22652	0.01
Non-alcoholic Fatty	C0400966	ADAMTS9	56999	Q9P2N4	ADAM me	Enzyme	53	0.633	0.692	1E-06	0.01
Non-alcoholic Fatty	C0400966	BAAT	570	Q14032	bile acid-CoA:amino a		98	0.578	0.654	7.87E-08	0.01
Non-alcoholic Fatty	C0400966	PCBP4	57060	P57723	poly(rC) bi	Enzyme	147	0.523	0.808	0.002371	0.01
Non-alcoholic Fatty	C0400966	SMURF1	57154	Q9HCE7	SMAD spe	Enzyme	70	0.595	0.615	0.6118	0.01
Non-alcoholic Fatty	C0400966	PTBP1	5725	P26599	polypyrimidine tract b		172	0.517	0.769	0.99961	0.01
Non-alcoholic Fatty	C0400966	PTGFRN	5738	Q9P2B2	prostaglandin F2 recep		14	0.76	0.269	0.000476	0.01
Non-alcoholic Fatty	C0400966	MIR20B	#####		microRNA 20b		84	0.581	0.615		0.01
Non-alcoholic Fatty	C0400966	MIR193B	#####		microRNA 193b		117	0.536	0.808		0.01
Non-alcoholic Fatty	C0400966	LRRC7	57554	Q96NW7	leucine rich repeat cor		26	0.722	0.462	1	0.01
Non-alcoholic Fatty	C0400966	SEMA6A	57556	Q9H2E6	semaphori	Signaling	211	0.486	0.808	0.99976	0.01
Non-alcoholic Fatty	C0400966	ARRDC3	57561	Q96B67	arrestin domain contai		24	0.711	0.308	0.98715	0.01
Non-alcoholic Fatty	C0400966	PTPN6	5777	P29350	protein tyrosine phosp		218	0.485	0.769	0.99999	0.01
Non-alcoholic Fatty	C0400966	PTPRC	5788	P08575	protein tyr	Enzyme	366	0.435	0.808	1	0.01
Non-alcoholic Fatty	C0400966	PTX3	5806	P26022	pentraxin 3		296	0.457	0.808	6.98E-05	0.01
Non-alcoholic Fatty	C0400966	BAX	581	Q07812	BCL2 asso	Signaling	420	0.417	0.885	0.31746	0.01
Non-alcoholic Fatty	C0400966	PGAP6	58986	Q9HCN3	post-glyco	Cell adhes	3	1	0.038	4.37E-10	0.01
Non-alcoholic Fatty	C0400966	BCHE	590	P06276	butyrylcholinesterase		392	0.447	0.923	1.06E-13	0.01
Non-alcoholic Fatty	C0400966	RARA	5914	P10276	retinoic aci	Nuclear re	274	0.47	0.846	0.96305	0.01
Non-alcoholic Fatty	C0400966	TRPV4	59341	Q9HBA0	transient re	Ion channe	422	0.457	0.808	2.24E-16	0.01

Non-alcoholic Fatty	C0400966	OPN1LW	5956	P04000	opsin 1, lo	G-protein	197	0.497	0.808	0.97535	0.01
Non-alcoholic Fatty	C0400966	RELA	5970	Q04206	RELA prot	Transcripti	483	0.406	0.885	0.99948	0.01
Non-alcoholic Fatty	C0400966	ACTB	60	P60709	actin beta	Cellular st	1110	0.325	0.923	0.98564	0.01
Non-alcoholic Fatty	C0400966	RLN2	6019	P04090	relaxin 2	Signaling	54	0.626	0.615	0.0009	0.01
Non-alcoholic Fatty	C0400966	BRD2	6046	P25440	bromodom	Epigenetic	303	0.45	0.808	0.99957	0.01
Non-alcoholic Fatty	C0400966	RPS6KB1	6198	P23443	ribosomal	Kinase	169	0.511	0.731	0.97285	0.01
Non-alcoholic Fatty	C0400966	SORT1	6272	Q99523	sortilin 1	Transporte	111	0.573	0.615	0.99987	0.01
Non-alcoholic Fatty	C0400966	S100A4	6275	P26447	S100 calci	Calcium-b	308	0.448	0.769	0.006184	0.01
Non-alcoholic Fatty	C0400966	CCL5	6352	P13501	C-C motif	Signaling	514	0.403	0.885	0.001752	0.01
Non-alcoholic Fatty	C0400966	CCL17	6361	Q92583	C-C motif	Signaling	162	0.518	0.808	0.000139	0.01
Non-alcoholic Fatty	C0400966	CCL19	6363	Q99731	C-C motif	Signaling	126	0.542	0.692	0.31691	0.01
Non-alcoholic Fatty	C0400966	CXCL5	6374	P42830	C-X-C mo	Signaling	160	0.519	0.769	5.04E-06	0.01
Non-alcoholic Fatty	C0400966	CIDEC	63924	Q96AQ7	cell death inducing Dl		43	0.666	0.462	0.008966	0.01
Non-alcoholic Fatty	C0400966	SELL	6402	P14151	selectin L		171	0.505	0.846	4.96E-07	0.01
Non-alcoholic Fatty	C0400966	SELP	6403	P16109	selectin P		293	0.46	0.769	5.04E-32	0.01
Non-alcoholic Fatty	C0400966	SMOC2	64094	Q9H3U7	SPARC re	Calcium-b	42	0.67	0.462	0.002528	0.01
Non-alcoholic Fatty	C0400966	TMBIM1	64114	Q969X1	transmembrane BAX		30	0.711	0.154	1.36E-08	0.01
Non-alcoholic Fatty	C0400966	SETMAR	6419	Q53H47	SET doma	Epigenetic	61	0.617	0.692	0.000102	0.01
Non-alcoholic Fatty	C0400966	SFRP4	6424	Q6FHJ7	secreted frizzled relat		155	0.524	0.654	9.26E-05	0.01
Non-alcoholic Fatty	C0400966	ABCG8	64241	Q9H221	ATP bindi	Transporte	116	0.573	0.538	2.38E-20	0.01
Non-alcoholic Fatty	C0400966	SFRP5	6425	Q5T4F7	secreted frizzled relat		106	0.559	0.692	5.18E-06	0.01
Non-alcoholic Fatty	C0400966	SRSF3	6428	P84103	serine and	Nucleic ac	60	0.619	0.615	0.96323	0.01
Non-alcoholic Fatty	C0400966	NSD1	64324	Q96L73	nuclear rec	Epigenetic	247	0.505	0.769	1	0.01
Non-alcoholic Fatty	C0400966	XPO4	64328	Q9C0E2	exportin 4	Transporte	17	0.751	0.192	1	0.01
Non-alcoholic Fatty	C0400966	BLVRA	644	P53004	biliverdin	Enzyme	34	0.663	0.423	0.000348	0.01
Non-alcoholic Fatty	C0400966	GORASP1	64689	Q9BQQ3	golgi reassembly stacl		369	0.428	0.846	0.019543	0.01
Non-alcoholic Fatty	C0400966	ARV1	64801	Q9H2C2	ARV1 homolog, fatty		57	0.691	0.423	3.59E-06	0.01
Non-alcoholic Fatty	C0400966	ST3GAL4	6484	Q11206	ST3 beta-galactoside		115	0.588	0.692	0.039802	0.01
Non-alcoholic Fatty	C0400966	WNK1	65125	Q9H4A3	WNK lysir	Kinase	460	0.416	0.846	1	0.01
Non-alcoholic Fatty	C0400966	SLC2A4	6517	P14672	solute carri	Transporte	158	0.528	0.769	4.71E-05	0.01
Non-alcoholic Fatty	C0400966	BMP4	652	P12644	bone morp	Signaling	423	0.422	0.808	0.9564	0.01
Non-alcoholic Fatty	C0400966	SLC3A2	6520	P08195	solute carri	Transporte	108	0.565	0.731	4.22E-08	0.01
Non-alcoholic Fatty	C0400966	FRTS1	65211		Fanconi renotubular s		69	0.599	0.654		0.01
Non-alcoholic Fatty	C0400966	SLC6A2	6530	P23975	solute carri	Transporte	238	0.49	0.846	0.081524	0.01
Non-alcoholic Fatty	C0400966	SNHG20	#####		small nucleolar RNA		41	0.65	0.346		0.01
Non-alcoholic Fatty	C0400966	SLC10A1	6554	Q14973	solute carri	Transporte	54	0.626	0.462	4.79E-16	0.01
Non-alcoholic Fatty	C0400966	BMP8B	656	P34820	bone morp	Signaling	12	0.792	0.192	0.00019	0.01
Non-alcoholic Fatty	C0400966	SLC13A1	6561	Q9BZW2	solute carri	Transporte	7	0.861	0.269	1.23E-17	0.01
Non-alcoholic Fatty	C0400966	AACS	65985	Q86V21	acetoacety	Enzyme	12	0.751	0.308	9.13E-17	0.01

Non-alcoholic Fatty	C0400966	SMPD1	6609	P17405	sphingomy	Enzyme	247	0.499	0.885	3.89E-10	0.01
Non-alcoholic Fatty	C0400966	SIGLEC1	6614	Q9BZZ2	sialic acid binding Ig l		75	0.597	0.654	5.33E-46	0.01
Non-alcoholic Fatty	C0400966	SNAI1	6615	O95863	snail family transcript		243	0.474	0.808	0.21754	0.01
Non-alcoholic Fatty	C0400966	SORD	6652	Q00796	sorbitol de	Enzyme	113	0.552	0.769	4.49E-08	0.01
Non-alcoholic Fatty	C0400966	SP1	6667	P08047	Sp1 transc	Nucleic ac	209	0.493	0.769	0.99997	0.01
Non-alcoholic Fatty	C0400966	SPRR2A	6700	P35326	small proline rich prot		67	0.612	0.731		0.01
Non-alcoholic Fatty	C0400966	SQLE	6713	Q14534	squalene e	Enzyme	36	0.674	0.577	0.97594	0.01
Non-alcoholic Fatty	C0400966	AKR1D1	6718	P51857	aldo-keto r	Enzyme	40	0.711	0.385	1.72E-07	0.01
Non-alcoholic Fatty	C0400966	STAR	6770	P49675	steroidogenic acute re		177	0.513	0.808	8.77E-11	0.01
Non-alcoholic Fatty	C0400966	STAT1	6772	P42224	signal trans	Nucleic ac	531	0.399	0.885	0.99999	0.01
Non-alcoholic Fatty	C0400966	STAT6	6778	P42226	signal trans	Nucleic ac	281	0.475	0.808	0.98849	0.01
Non-alcoholic Fatty	C0400966	SULT1E1	6783	P49888	sulfotransferase famil		171	0.508	0.808	1.97E-07	0.01
Non-alcoholic Fatty	C0400966	STK11	6794	Q15831	serine/thre	Kinase	372	0.435	0.808	0.9934	0.01
Non-alcoholic Fatty	C0400966	SYT1	6857	P21579	synaptotag	Transporte	460	0.412	0.846	0.60038	0.01
Non-alcoholic Fatty	C0400966	ADAM17	6868	P78536	ADAM metallopeptid		316	0.453	0.808	0.99528	0.01
Non-alcoholic Fatty	C0400966	MAP3K7	6885	O43318	mitogen-ac	Kinase	273	0.477	0.808	0.99971	0.01
Non-alcoholic Fatty	C0400966	TAZ	6901	Q16635	tafazzin	Enzyme	252	0.477	0.808	0.72591	0.01
Non-alcoholic Fatty	C0400966	MIR33B	#####		microRNA 33b		61	0.601	0.615		0.01
Non-alcoholic Fatty	C0400966	MIR590	#####		microRNA 590		99	0.553	0.692		0.01
Non-alcoholic Fatty	C0400966	TCF7L2	6934	Q9NQB0	transcription factor 7 l		257	0.485	0.846	0.99537	0.01
Non-alcoholic Fatty	C0400966	TFAM	7019	Q00059	transcription factor A,		147	0.529	0.731	0.17259	0.01
Non-alcoholic Fatty	C0400966	TFF3	7033	Q07654	trefoil fact	Signaling	163	0.518	0.885	0.01591	0.01
Non-alcoholic Fatty	C0400966	TG	7038	P01266	thyroglobu	Enzyme m	240	0.48	0.808	2.88E-59	0.01
Non-alcoholic Fatty	C0400966	TGFB1I1	7041	O43294	transformi	Cellular st	47	0.638	0.423	0.027032	0.01
Non-alcoholic Fatty	C0400966	TSPO	706	P30536	translocator protein		341	0.444	0.885	0.000274	0.01
Non-alcoholic Fatty	C0400966	THRSP	7069	Q92748	thyroid hormone respo		33	0.695	0.462	0.002924	0.01
Non-alcoholic Fatty	C0400966	TIMP3	7078	P35625	TIMP meta	Enzyme m	325	0.445	0.923	0.63037	0.01
Non-alcoholic Fatty	C0400966	TLL1	7092	O43897	tolloid like 1		24	0.716	0.5	0.17306	0.01
Non-alcoholic Fatty	C0400966	TLR1	7096	Q15399	toll like receptor 1		198	0.496	0.808	5.87E-23	0.01
Non-alcoholic Fatty	C0400966	NR2E1	7101	Q9Y466	nuclear rec	Nuclear re	57	0.628	0.538	0.99864	0.01
Non-alcoholic Fatty	C0400966	TM7SF2	7108	O76062	transmemb	Enzyme	217	0.486	0.808	1.37E-07	0.01
Non-alcoholic Fatty	C0400966	TMPO	7112	P42166;P42167	thymopoie	Signaling	58	0.631	0.577	1.55E-07	0.01
Non-alcoholic Fatty	C0400966	TNFAIP3	7128	P21580	TNF alpha	Enzyme	212	0.481	0.808	0.99972	0.01
Non-alcoholic Fatty	C0400966	TNFRSF1A	7132	P19438	TNF receptor superfa		487	0.409	0.923	0.9949	0.01
Non-alcoholic Fatty	C0400966	TP53BP1	7158	Q12888	tumor prot	Epigenetic	158	0.52	0.731	0.99985	0.01
Non-alcoholic Fatty	C0400966	TPO	7173	P07202	thyroid per	Enzyme	306	0.455	0.846	1.82E-21	0.01
Non-alcoholic Fatty	C0400966	C3	718	P01024	compleme	Enzyme m	343	0.445	0.885	0.90421	0.01
Non-alcoholic Fatty	C0400966	TRAF3	7187	Q13114	TNF recep	Signaling	129	0.541	0.769	0.99869	0.01
Non-alcoholic Fatty	C0400966	DNLZ	#####	Q5SXM8	DNL-type zinc finger		43	0.663	0.538	0.000247	0.01

Non-alcoholic Fatty	C0400966	HULC	#####		hepatocellular carcino		72	0.606	0.538		0.01
Non-alcoholic Fatty	C0400966	UCP1	7350	P25874	uncoupling	Transporte	124	0.545	0.769	1.69E-11	0.01
Non-alcoholic Fatty	C0400966	UCP3	7352	P55916	uncoupling	Transporte	66	0.619	0.577	9.55E-10	0.01
Non-alcoholic Fatty	C0400966	SLC35A2	7355	P78381	solute carri	Transporte	197	0.525	0.808	0.71792	0.01
Non-alcoholic Fatty	C0400966	UGT1A	7361		UDP glucuronosyltrar		117	0.54	0.654		0.01
Non-alcoholic Fatty	C0400966	UGT2B4	7363	P06133	UDP glucuronosyltrar		16	0.751	0.231	4.38E-09	0.01
Non-alcoholic Fatty	C0400966	USP4	7375	Q13107	ubiquitin s	Enzyme	40	0.686	0.308	1.61E-09	0.01
Non-alcoholic Fatty	C0400966	UROD	7389	P06132	uroporphyrinogen dec		166	0.521	0.769	1.26E-08	0.01
Non-alcoholic Fatty	C0400966	UTRN	7402	P46939	utrophin		152	0.525	0.692	1E-19	0.01
Non-alcoholic Fatty	C0400966	VCAM1	7412	P19320	vascular cell adhesion		406	0.422	0.808	0.79791	0.01
Non-alcoholic Fatty	C0400966	VEGFC	7424	P49767	vascular er	Signaling	291	0.459	0.769	0.49565	0.01
Non-alcoholic Fatty	C0400966	VIP	7432	P01282	vasoactive	Signaling	376	0.437	0.846	0.00042	0.01
Non-alcoholic Fatty	C0400966	BEST1	7439	O76090	bestrophin	Ion channe	292	0.465	0.885	1.82E-12	0.01
Non-alcoholic Fatty	C0400966	YY1	7528	P25490	YY1 trans	Transcripti	245	0.481	0.808	0.9938	0.01
Non-alcoholic Fatty	C0400966	ZBTB16	7704	Q05516	zinc finger	Transcripti	154	0.545	0.808	0.99907	0.01
Non-alcoholic Fatty	C0400966	DUSP26	78986	Q9BV47	dual specificity phosp		35	0.682	0.346	0.00023	0.01
Non-alcoholic Fatty	C0400966	ELOVL6	79071	Q9H5J4	ELOVL fa	Enzyme	51	0.623	0.731	0.96581	0.01
Non-alcoholic Fatty	C0400966	APOO	79135	Q9BUR5	apolipoprotein O		6	0.839	0.192	0.60201	0.01
Non-alcoholic Fatty	C0400966	LAP	7939		Laryngeal adductor pa		50	0.619	0.731		0.01
Non-alcoholic Fatty	C0400966	PLA2G7	7941	Q13093	phospholip	Enzyme	136	0.537	0.769	5.33E-22	0.01
Non-alcoholic Fatty	C0400966	TFEB	7942	P19484	transcription factor EI		150	0.527	0.731	0.89931	0.01
Non-alcoholic Fatty	C0400966	CALCA	796	P01258;P06881	calcitonin	Signaling	467	0.416	0.923	0.015562	0.01
Non-alcoholic Fatty	C0400966	AIMP2	7965	Q13155	aminoacyl	tRNA synth	555	0.393	0.923	0.008024	0.01
Non-alcoholic Fatty	C0400966	NLRX1	79671	Q86UT6	NLR famil	Enzyme	51	0.641	0.654	7.73E-31	0.01
Non-alcoholic Fatty	C0400966	KCTD17	79734	Q8N5Z5	potassium channel tet		22	0.743	0.308	0.017683	0.01
Non-alcoholic Fatty	C0400966	GSDMD	79792	P57764	gasdermin D		50	0.641	0.692	1.08E-13	0.01
Non-alcoholic Fatty	C0400966	DHDDS	79947	Q86SQ9	dehydrodo	Enzyme	308	0.468	0.885	0.24533	0.01
Non-alcoholic Fatty	C0400966	HKDC1	80201	Q2TB90	hexokinase	Kinase	26	0.743	0.269	1.62E-14	0.01
Non-alcoholic Fatty	C0400966	STAM	8027	Q92783	signal transducing ada		20	0.736	0.269	0.85048	0.01
Non-alcoholic Fatty	C0400966	CPEB4	80315	Q17RY0	cytoplasmi	Nucleic ac	70	0.621	0.462	0.98555	0.01
Non-alcoholic Fatty	C0400966	COASY	80347	Q13057	Coenzyme	Enzyme	125	0.551	0.654	8.09E-10	0.01
Non-alcoholic Fatty	C0400966	FOSL1	8061	P15407	FOS like 1	Transcripti	150	0.519	0.846	0.15849	0.01
Non-alcoholic Fatty	C0400966	FGF23	8074	Q9GZV9	fibroblast g	Signaling	305	0.46	0.731	0.031014	0.01
Non-alcoholic Fatty	C0400966	SPX	80763	Q9BT56	spexin hormone		33	0.695	0.462	0.00011	0.01
Non-alcoholic Fatty	C0400966	SLC7A5	8140	Q01650	solute carri	Transporte	173	0.513	0.808	0.23505	0.01
Non-alcoholic Fatty	C0400966	BAS	8213		Beta-adrenergic stimu		29	0.711	0.346		0.01
Non-alcoholic Fatty	C0400966	SESN2	83667	P58004	sestrin 2	Enzyme	169	0.508	0.808	4.19E-07	0.01
Non-alcoholic Fatty	C0400966	TRAPPC9	83696	Q96Q05	trafficking protein par		111	0.603	0.577	2.29E-14	0.01
Non-alcoholic Fatty	C0400966	ITCH	83737	Q96J02	itchy E3 ul	Enzyme	123	0.556	0.769	0.99997	0.01

Non-alcoholic Fatty	C0400966	ANGPTL6	83854	Q8NI99	angiopoieti	Signaling	48	0.656	0.462	2.37E-08	0.01
Non-alcoholic Fatty	C0400966	PLA2G6	8398	O60733	phospholip	Enzyme m	350	0.476	0.846	2.48E-10	0.01
Non-alcoholic Fatty	C0400966	CHCHD6	84303	Q9BRQ6	coiled-coil-helix-coile		7	0.839	0.269	5.57E-09	0.01
Non-alcoholic Fatty	C0400966	MAK16	84549	Q9BXY0	MAK16 homolog		96	0.566	0.615	0.96452	0.01
Non-alcoholic Fatty	C0400966	DGAT2	84649	Q96PD7	diacylglyce	Enzyme	41	0.686	0.423	8.36E-12	0.01
Non-alcoholic Fatty	C0400966	CARD6	84674	Q9BX69	caspase recruitment do		6	0.861	0.115	3.39E-13	0.01
Non-alcoholic Fatty	C0400966	FAM83A	84985	Q86UY5	family with sequence		20	0.76	0.192	3.03E-07	0.01
Non-alcoholic Fatty	C0400966	PIK3R3	8503	Q92569	phosphoino	Enzyme m	101	0.556	0.692	3.36E-10	0.01
Non-alcoholic Fatty	C0400966	CYP4F2	8529	P78329	cytochrom	Enzyme	44	0.674	0.308	7.58E-17	0.01
Non-alcoholic Fatty	C0400966	DENR	8562	O43583	density reg	Enzyme	121	0.543	0.692	0.87514	0.01
Non-alcoholic Fatty	C0400966	RUNX2	860	Q13950	RUNX fan	Transcripti	405	0.437	0.846	0.94884	0.01
Non-alcoholic Fatty	C0400966	RTCA	8634	O00442	RNA 3'-ter	Enzyme	22	0.736	0.385	3.09E-09	0.01
Non-alcoholic Fatty	C0400966	DGAT1	8694	O75907	diacylglyce	Enzyme	64	0.621	0.615	1.41E-17	0.01
Non-alcoholic Fatty	C0400966	ABCC3	8714	O15438	ATP bindi	Transporte	160	0.517	0.769	1.21E-26	0.01
Non-alcoholic Fatty	C0400966	MBTPS1	8720	Q14703	membrane	Enzyme	148	0.532	0.692	7.59E-09	0.01
Non-alcoholic Fatty	C0400966	TNFSF14	8740	O43557	TNF super	Signaling	143	0.529	0.769	0.051663	0.01
Non-alcoholic Fatty	C0400966	DLK1	8788	P80370	delta like n	Signaling	144	0.527	0.692	0.034576	0.01
Non-alcoholic Fatty	C0400966	TRIM24	8805	O15164	tripartite motif contain		79	0.581	0.615	1	0.01
Non-alcoholic Fatty	C0400966	CES2	8824	O00748	carboxyles	Enzyme	87	0.584	0.731	3.58E-16	0.01
Non-alcoholic Fatty	C0400966	HDAC3	8841	O15379	histone dea	Epigenetic	179	0.511	0.731	0.56737	0.01
Non-alcoholic Fatty	C0400966	SQSTM1	8878	Q13501	sequestosome 1		470	0.428	0.885	0.00086	0.01
Non-alcoholic Fatty	C0400966	CCNC	892	P24863	cyclin C	Enzyme m	24	0.695	0.462	0.99914	0.01
Non-alcoholic Fatty	C0400966	MBD2	8932	Q9UBB5	methyl-Cp	Epigenetic	256	0.466	0.846	0.34957	0.01
Non-alcoholic Fatty	C0400966	WNT3A	89780	P56704	Wnt family	Signaling	141	0.532	0.769	0.96491	0.01
Non-alcoholic Fatty	C0400966	CCRL2	9034	O00421	C-C motif	G-protein c	59	0.621	0.654	0.36861	0.01
Non-alcoholic Fatty	C0400966	TBL1Y	90665	Q9BQ87	transducin beta like 1		22	0.705	0.385	4.03E-13	0.01
Non-alcoholic Fatty	C0400966	MYOM2	9172	P54296	myomesin 2		103	0.57	0.692	7.01E-85	0.01
Non-alcoholic Fatty	C0400966	TIMD4	91937	Q96H15	T cell immunoglobulin		49	0.644	0.654	2.64E-10	0.01
Non-alcoholic Fatty	C0400966	ZNF300	91975	Q96RE9	zinc finger protein 300		16	0.76	0.115	1.12E-05	0.01
Non-alcoholic Fatty	C0400966	HACD1	9200	B0YJ81	3-hydroxyacyl-CoA de		160	0.539	0.846	1.29E-09	0.01
Non-alcoholic Fatty	C0400966	NOG	9241	Q13253	noggin		206	0.518	0.731	0.89026	0.01
Non-alcoholic Fatty	C0400966	GLP2R	9340	O95838	glucagon li	G-protein c	28	0.705	0.269	2.64E-11	0.01
Non-alcoholic Fatty	C0400966	COX5A	9377	P20674	cytochrom	Enzyme	171	0.515	0.846	0.49407	0.01
Non-alcoholic Fatty	C0400966	GRAP2	9402	O75791	GRB2 related adaptor		538	0.394	0.923	0.000153	0.01
Non-alcoholic Fatty	C0400966	TMPRSS11D	9407	O60235	transmemb	Enzyme	48	0.636	0.538	1.2E-05	0.01
Non-alcoholic Fatty	C0400966	EIF2AK3	9451	Q9NZJ5	eukaryotic	Kinase	231	0.501	0.808	0.00276	0.01
Non-alcoholic Fatty	C0400966	GGPS1	9453	O95749	geranylgeranyl diphos		44	0.647	0.577	0.26866	0.01
Non-alcoholic Fatty	C0400966	ATG5	9474	Q9H1Y0	autophagy	Transporte	282	0.462	0.885	0.97758	0.01
Non-alcoholic Fatty	C0400966	LITAF	9516	Q99732	lipopolysac	Transcripti	59	0.65	0.462	0.030752	0.01

Non-alcoholic Fatty	C0400966	CLOCK	9575	O15516	clock circadian regula		309	0.463	0.885	0.99957	0.01
Non-alcoholic Fatty	C0400966	CD44	960	P16070	CD44 molecule (India		711	0.363	0.962	2.45E-07	0.01
Non-alcoholic Fatty	C0400966	ABCG1	9619	P45844	ATP bindi	Transporte	118	0.56	0.692	0.11182	0.01
Non-alcoholic Fatty	C0400966	KEAP1	9817	Q14145	kelch like ECH associ		251	0.475	0.808	0.000388	0.01
Non-alcoholic Fatty	C0400966	PLPPR4	9890	Q7Z2D5	phospholip	Enzyme	10	0.839	0.154	0.86104	0.01
Non-alcoholic Fatty	C0400966	MFN2	9927	O95140	mitofusin 2	Enzyme	334	0.471	0.846	0.99415	0.01
Non-alcoholic Fatty	C0400966	CDC42	998	P60953	cell divisio	Enzyme m	327	0.458	0.846	0.78728	0.01
Non-alcoholic Fatty	C0400966	CDH1	999	P12830	cadherin 1		508	0.401	0.808	0.14631	0.01

Information of OMIM

OMIM Search - 'non-alcoholic fatty liver '

Downloaded: Dec 14, 2020

Copyright (c) 1966-2020 Johns Hopkins University OMIM, data are provided for research purposes only.

MIM Number	Title	Included Titles	Cytoge	Genomic Coordin	Entrez Gene ID
%613282	FATTY LIVER DISEASE, NONALCOHOLIC, SUSC	LIVER DISEASE,	22q13	22:37200000-508	100380876
%613387	FATTY LIVER DISEASE, NONALCOHOLIC, SUSC		11q23	11:110600000-12	100462725
#603471	CITRULLINEMIA, TYPE II, ADULT-ONSET; CTLN		7q21.3		
*601739	MEIS HOMEOBOX 1; MEIS1		2p14	2:66435124-6657	4211
%228100	VISCERAL STEATOSIS, CONGENITAL				
*107720	APOLIPOPROTEIN C-III; APOC3	APOC3/APOA1 FU	11q23.	11:116829906-11	345
*134650	FATTY ACID-BINDING PROTEIN 1; FABP1		2p11.2	2:88122981-8812	2168
*603030	TOLL-LIKE RECEPTOR 4; TLR4		9q33.1	9:117704402-117	7099
*138030	GLUCAGON; GCG	GLUCAGON-LIKE	2q24.2	2:162142881-162	2641
*600953	INTERLEUKIN 18; IL18		11q23.	11:112143250-11	3606
*609567	PATATIN-LIKE PHOSPHOLIPASE DOMAIN-CON		22q13.	22:43923804-439	80339
*603598	TUMOR NECROSIS FACTOR LIGAND SUPERFAM		3q26.3	3:172505507-172	8743
%612364	ALANINE AMINOTRANSFERASE, PLASMA LEV				
*147265	INOSITOL 1,4,5-TRIPHOSPHATE RECEPTOR, TY		3p26.1	3:4493347-48475	3708
*613524	SEROLOGICALLY DEFINED COLON CANCER AI		1q43-q	1:243255417-243	10806
#144250	HYPERLIPIDEMIA, FAMILIAL COMBINED, 3; FC		8p21.3		
#615993	BARDET-BIEDL SYNDROME 16; BBS16		1q43-q		
%604499	HYPERLIPIDEMIA, FAMILIAL COMBINED, 2; FC		11p		28515
*134651	FATTY ACID-BINDING PROTEIN 3; FABP3		1p35.2	1:31359594-3137	2170
*600691	SOLUTE CARRIER FAMILY 27 (FATTY ACID TR		19p13.	19:17468744-175	376497
*603247	SOLUTE CARRIER FAMILY 27 (FATTY ACID TR		15q21.	15:50182195-502	11001
*605168	FATTY ACID-BINDING PROTEIN 5; FABP5		8q21.1	8:81280535-8128	2171
*611026	FATTY ACID 2-HYDROXYLASE; FA2H		16q23.	16:74712954-747	79152
*611546	ELONGATION OF VERY LONG CHAIN FATTY A		4q25	4:110045845-110	79071

#240600	GLYCOGEN STORAGE DISEASE 0, LIVER; GSD0		12p12.		
#270200	SJOGREN-LARSSON SYNDROME; SLS		17p11.		
*173510	CD36 ANTIGEN; CD36		7q21.1	7:80602206-8067	948
*134640	FATTY ACID-BINDING PROTEIN 2; FABP2		4q26	4:119317249-119	2169
*600422	FATTY ACID-BINDING PROTEIN 6; FABP6		5q33.3	5:160187380-160	2172
*600434	FATTY ACID-BINDING PROTEIN 4; FABP4		8q21.1	8:81478418-8148	2167
*600976	FAT ATYPICAL CADHERIN 1; FAT1		4q35.2	4:186587788-186	2195
*602965	FATTY ACID-BINDING PROTEIN 7; FABP7		6q22.3	6:122749200-122	2173
*603314	SOLUTE CARRIER FAMILY 27 (FATTY ACID TR.		19q13.	19:58498332-585	10998
*604193	SOLUTE CARRIER FAMILY 27 (FATTY ACID TR.		1q21.3	1:153775406-153	11000
*604194	SOLUTE CARRIER FAMILY 27 (FATTY ACID TR.		9q34.1	9:128340515-128	10999
*604196	SOLUTE CARRIER FAMILY 27 (FATTY ACID TR.				28965
*604269	FAT ATYPICAL CADHERIN 2; FAT2		5q33.1	5:151504091-151	2196
*152425	ACYL-CoA SYNTHETASE LONG CHAIN FAMILY		4q35.1	4:184755594-184	2180
*612028	FAT STORAGE-INDUCING TRANSMEMBRANE I		14q12	14:24130658-241	161247
*612029	FAT STORAGE-INDUCING TRANSMEMBRANE I		20q13.	20:44302839-443	128486
*612411	FAT ATYPICAL CADHERIN 4; FAT4		4q28.1	4:125314917-125	79633
*612483	FAT ATYPICAL CADHERIN 3; FAT3		11q14.	11:92224635-928	120114
*614451	ELONGATION OF VERY LONG CHAIN FATTY A		5q12.1	5:60751790-6084	79993
#114550	HEPATOCELLULAR CARCINOMA	HEPATOBLASTO	2q33.1		
#306000	GLYCOGEN STORAGE DISEASE IXa1; GSD9A1	GLYCOGEN STOI	Xp22.1		
#261750	GLYCOGEN STORAGE DISEASE IXb; GSD9B		16q12.		
#615486	INTERSTITIAL LUNG AND LIVER DISEASE; ILLI		12q13.		
*171760	ALKALINE PHOSPHATASE, LIVER; ALPL		1p36.1	1:21508983-2157	249
*171860	PHOSPHOFRUCTOKINASE, LIVER TYPE; PFKL		21q22.	21:44300039-443	5211
*138571	GLYCOGEN SYNTHASE 2; GYS2		12p12.	12:21531526-216	2998
*189965	CCAAT/ENHANCER-BINDING PROTEIN, BETA; (		20q13.	20:50190582-501	1051
*600528	CARNITINE PALMITOYLTRANSFERASE I, LIVEI		11q13.	11:68754619-688	1374
*604426	CYTOCHROME P450, FAMILY 4, SUBFAMILY F,		19p13.	19:15878022-158	8529
*609712	PYRUVATE KINASE, LIVER AND RED BLOOD C		1q22	1:155289292-155	5313
*613009	O-PHOSPHOSERINE tRNA-SELENOCYSTEINE tR		4p15.2	4:25120013-2516	51091
#144650	HYPERLIPOPROTEINEMIA, TYPE V		11q23.		
#221770	POLYCYSTIC LIPOMEMBRANOUS OSTEODYSP.		19q13.		
#275630	CHANARIN-DORFMAN SYNDROME; CDS		3p21.3		
#612319	SPASTIC PARAPLEGIA 35, AUTOSOMAL RECES		16q23.		
#616154	PEROXISOMAL FATTY ACYL-CoA REDUCTASE		11p15.		
%612795	POLYUNSATURATED FATTY ACIDS PLASMA L		11q12-	11:55800000-661	100302559
138920	GRANDDAD SYNDROME				
603376	LONG CHAIN FATTY ACIDS, DEFECT IN TRANS				

*134660	GLUTATHIONE S-TRANSFERASE, PI; GSTP1	GLUTATHIONE S	11q13.	11:67583811-675	2950
*600212	FATTY ACID SYNTHASE; FASN		17q25.	17:82078337-820	2194
*601310	CYTOCHROME P450, SUBFAMILY IVA, POLYPE		1p33	1:46929176-4694	1579
*602281	MILK FAT GLOBULE-EGF FACTOR 8; MFGE8	MEDIN, INCLUDI	15q26.	15:88898682-889	4240
*602371	ACYL-CoA SYNTHETASE LONG CHAIN FAMILY		2q36.1	2:222861035-222	2181
*602935	FATTY ACID AMIDE HYDROLASE; FAAH		1p33	1:46394316-4641	2166
*300072	UBIQUITIN-SPECIFIC PROTEASE 9, X-LINKED; U		Xp11.4	X:41085419-4123	8239
*300157	ACYL-CoA SYNTHETASE LONG CHAIN FAMILY		Xq23	X:109641334-109	2182
*300654	FATTY ACID AMIDE HYDROLASE 2; FAAH2		Xp11.2	X:57121860-5748	158584
*400005	UBIQUITIN-SPECIFIC PROTEASE 9, Y CHROMOS		Yq11.2	Y:12701230-1286	8287
*603820	FREE FATTY ACID RECEPTOR 1; FFAR1		19q13.	19:35351541-353	2864
*603821	FREE FATTY ACID RECEPTOR 3; FFAR3		19q13.	19:35358105-353	2865
*603823	FREE FATTY ACID RECEPTOR 2; FFAR2		19q13.	19:35448256-354	2867
*604443	ACYL-CoA SYNTHETASE LONG CHAIN FAMILY	ACS2/ETV6 FUSIO	5q31.1	5:131949972-132	23305
*605512	ELONGATION OF VERY LONG CHAIN FATTY A		6q14.1	6:79914813-7994	6785
*605677	ACYL-CoA SYNTHETASE LONG CHAIN FAMILY		10q25.	10:112374115-11	51703
*606148	FATTY ACID DESATURASE 1; FADS1		11q12.	11:61799626-618	3992
*606149	FATTY ACID DESATURASE 2; FADS2		11q12.	11:61816202-618	9415
*606150	FATTY ACID DESATURASE 3; FADS3		11q12.	11:61873525-618	3995
*606803	ACYL-CoA THIOESTERASE 11; ACOT11	BFIT1, INCLUDEI	1p32.3	1:54548227-5463	26027
*609044	FREE FATTY ACID RECEPTOR 4; FFAR4		10q23.	10:93566177-935	338557
*609196	MELANOCORTIN 2 RECEPTOR ACCESSORY PRO		21q22.	21:32291812-323	56246
*609523	ALDEHYDE DEHYDROGENASE, FAMILY 3, SUB		17p11.	17:19648135-196	224
*610966	FTO ALPHA-KETOGLUTARATE-DEPENDENT DI		16q12.	16:53703962-541	79068
*611647	ARV1 HOMOLOG, FATTY ACID HOMEOSTASIS		1q42.2	1:230979081-231	64801
*611805	ELONGATION OF VERY LONG CHAIN FATTY A		6p12.1	6:53267403-5334	60481
*611813	ELONGATION OF VERY LONG CHAIN FATTY A		1p34.2	1:43363400-4336	64834
*611814	ELONGATION OF VERY LONG CHAIN FATTY A		6p24.2	6:10980758-1104	54898
*611815	ELONGATION OF VERY LONG CHAIN FATTY A		10q24.	10:102224766-10	83401
*612120	CELL DEATH-INDUCING DFFA-LIKE EFFECTOR		3p25.3	3:9866709-98802	63924
*613409	LACCASE (MULTICOPPER REDUCTASE) DOMA		13q14.	13:43879177-438	144811
*614359	ACYL-CoA SYNTHETASE MEDIUM CHAIN FAM		16p12.	16:20536225-205	348158
*616107	FATTY ACYL CoA REDUCTASE 1; FAR1		11p15.	11:13668665-137	84188
*616156	FATTY ACYL CoA REDUCTASE 2; FAR2		12p11.	12:29149100-293	55711
*618375	FATTY ACID AMIDE HYDROLASE PSEUDOGEN		1p33	1:46433826-4644	729041
*618923	FATTY ACID-BINDING PROTEIN 12; FABP12		8q21.1	8:81524672-8159	646486
+100650	ALDEHYDE DEHYDROGENASE 2 FAMILY; ALD	SUBLINGUAL NI	12q24.	12:111766932-11	217
+142830	MAJOR HISTOCOMPATIBILITY COMPLEX, CLA	ABACAVIR HYPE	6p21.3	6:31353874-3135	3106
#173900	POLYCYSTIC KIDNEY DISEASE 1 WITH OR WIT		16p13.		

#174050	POLYCYSTIC LIVER DISEASE 1 WITH OR WITH(		19p13.		
#142330	HEPATIC ADENOMAS, FAMILIAL		12q24.		
#203700	MITOCHONDRIAL DNA DEPLETION SYNDROM		15q26.		
#600666	POLYCYSTIC KIDNEY DISEASE 3 WITH OR WIT		11q12.		
#232700	GLYCOGEN STORAGE DISEASE VI; GSD6		14q22.		
#253250	MULIBREY NANISM		17q22		
#263200	POLYCYSTIC KIDNEY DISEASE 4 WITH OR WIT	HEPATIC FIBROS	6p12.3		
#613070	LIVER FAILURE, INFANTILE, TRANSIENT; LFIT		22q13.		
#613095	POLYCYSTIC KIDNEY DISEASE 2 WITH OR WIT		4q22.1		
#613658	RAJAB INTERSTITIAL LUNG DISEASE WITH BR		2q36.1		
#615438	INFANTILE LIVER FAILURE SYNDROME 1; ILFS		5q32		
#616483	INFANTILE LIVER FAILURE SYNDROME 2; ILFS		2p24.3		
#616719	SPINOCEREBELLAR ATAXIA, AUTOSOMAL RE(		11q13.		
#617004	POLYCYSTIC LIVER DISEASE 2 WITH OR WITH(		6q21		
#617874	POLYCYSTIC LIVER DISEASE 3 WITH OR WITH(		11q14.		
#617875	POLYCYSTIC LIVER DISEASE 4 WITH OR WITH(		11q13.		
#618061	POLYCYSTIC KIDNEY DISEASE 6 WITH OR WIT		3q27.3		
#618641	INFANTILE LIVER FAILURE SYNDROME 3; ILFS		7q22.3		
%189800	PREECLAMPSIA/ECLAMPSIA 1; PEE1	HYPERTENSION,	2p13	2:68400000-7480	5177
123997	CYTOCHROME c OXIDASE, SUBUNIT 7A2, PSEU				1348
602114	NEPHROPATHY, PROGRESSIVE TUBULOINTER:				
605944	LIVER FIBROCYSTIC DISEASE AND POLYDACT				
*100640	ALDEHYDE DEHYDROGENASE 1 FAMILY, MEM		9q21.1	9:72900670-7295	216
*114835	CARBOXYLESTERASE 1; CES1		16q12.	16:55802850-558	1066
*123996	CYTOCHROME c OXIDASE, SUBUNIT 7A2; COX		6q14.1	6:75237674-7525	1347
*601394	CHEMOKINE, CC MOTIF, LIGAND 16; CCL16		17q12	17:35976492-359	6360
*165320	LIVER CANCER ONCOGENE; LCO		2q14-q	2:112200000-136	
*172471	PHOSPHORYLASE KINASE, TESTIS/LIVER, GAM		16p11.	16:30748424-307	5261
*136130	FLAVIN-CONTAINING MONOOXYGENASE 1; FM		1q24.3	1:171248472-171	2326
*136131	FLAVIN-CONTAINING MONOOXYGENASE 4; FM		1q24.3	1:171314130-171	2329
*138079	GLUCOKINASE; GCK		7p13	7:44143212-4418	2645
*138160	SOLUTE CARRIER FAMILY 2 (FACILITATED GL		3q26.2	3:170996340-171	6514
*138200	GLUTAMATE PYRUVATE TRANSAMINASE; GPT		8q24.3	8:144503067-144	2875
*138350	GLUTATHIONE S-TRANSFERASE, MU-1; GSTM1		1p13.3	1:109687816-109	2944
*138360	GLUTATHIONE S-TRANSFERASE, ALPHA-2; GST		6p12.2	6:52750086-5276	2939
*182790	SPECTRIN, BETA, NONERYTHROCYTIC, 1; SPTE		2p16.2	2:54456326-5467	6711
*189907	HNF1 HOMEOBOX B; HNF1B		17q12	17:37686430-377	6928
*192090	CADHERIN 1; CDH1		16q22.	16:68737291-688	999
*600043	SULFOTRANSFERASE FAMILY 1E, ESTROGEN-I	ARYL SULFOTRA	4q13.3	4:69841211-6986	6783

*600924	GROWTH FACTOR, ERV1-LIKE; GFER	16p13.	16:1984192-1987	2671
*601584	PROTEIN-TYROSINE PHOSPHATASE, TYPE 4A, :	1p35.2	1:31906420-3193	8073
*601585	PROTEIN-TYROSINE PHOSPHATASE, TYPE 4A, :	6q12	6:63516439-6358	7803
*602072	CYTOCHROME c OXIDASE, SUBUNIT 6A1; COX(	12q24.	12:120438112-12	1337
*602131	PLECKSTRIN HOMOLOGY-LIKE DOMAIN, FAMI	11p15.	11:2928272-2929	7262
*602423	NUCLEAR RECEPTOR SUBFAMILY 1, GROUP H,	11p11.	11:47248299-472	10062
*602587	ACYL-CoA THIOESTERASE 7; ACOT7	1p36.3	1:6264271-63937	11332
*603017	CADHERIN 17; CDH17	8q22.1	8:94127161-9421	1015
*300151	SOLUTE CARRIER FAMILY 25 (MITOCHONDRIA	Xp22.3	X:1386151-13921	293
*300689	START DOMAIN-CONTAINING PROTEIN 8; STA	Xq13.1	X:68647665-6872	9754
*300798	PHOSPHORYLASE KINASE, LIVER, ALPHA-2 SU	Xp22.1	X:18892297-1898	5256
*304040	GAP JUNCTION PROTEIN, BETA-1; GJB1	Xq13.1	X:71215238-7122	2705
*311790	6-PHOSPHOFRUCTO-2-KINASE/FRUCTOSE-2,6-B	Xp11.2	X:54932960-5500	5207
*604258	DLC1 RHO GTPase ACTIVATING PROTEIN; DLC1	8p22	8:13083360-1360	10395
*604453	NUCLEAR RECEPTOR SUBFAMILY 5, GROUP A,	1q32.1	1:200027709-200	2494
*604843	SOLUTE CARRIER ORGANIC ANION TRANSPOR	12p12.	12:21131193-212	10599
*605872	C-TYPE LECTIN DOMAIN FAMILY 4, MEMBER M	19p13.	19:7763242-7769	10332
*606365	GLUTAMINASE 2; GLS2	12q13.	12:56470951-564	27165
*606464	HEPCIDIN ANTIMICROBIAL PEPTIDE; HAMP	19q13.	19:35282527-352	57817
*606567	TRANSMEMBRANE 4 SUPERFAMILY, MEMBER	3q25.1	3:149474696-149	7104
*607620	COLLECTIN 10; COLEC10	8q24.1	8:118952232-119	10584
*608313	ARGINASE 1; ARG1	6q23.2	6:131573225-131	383
*608603	GLIOMEDIN; GLDN	15q21.	15:51341654-514	342035
*609330	ONCOPROTEIN-INDUCED TRANSCRIPT 3, MOU	10q22.	10:72893738-729	170392
*609866	START DOMAIN-CONTAINING PROTEIN 13; STA	13q13.	13:33103136-336	90627
*610160	ZINC FINGER PROTEIN 367; ZNF367	9q22.3	9:96385940-9641	195828
*610541	PROTEIN PHOSPHATASE 1, REGULATORY SUBU	8p23.1	8:9136254-91515	79660
*610550	METHIONINE ADENOSYLTRANSFERASE I, ALP	10q22.	10:80271819-802	4143
*611327	DNAJ/HSP40 HOMOLOG, SUBFAMILY B, MEMBI	1p31.1	1:77979174-7801	11080
*611373	LIVER-EXPRESSED ANTIMICROBIAL PEPTIDE 2	5q31.1	5:132873667-132	116842
*611570	FRUCTOSE-1,6-BISPHOSPHATASE 1; FBP1	9q22.3	9:94603132-9464	2203
*612203	NUCLEOSOME ASSEMBLY PROTEIN 1-LIKE 5; N	4q22.1	4:88695912-8869	266812
*612210	HIGHLY UPREGULATED IN LIVER CANCER; HU	6p24.3	6:8652208-86538	728655
*613039	CHROMODOMAIN HELICASE DNA-BINDING PR	1q21.1	1:147172746-147	9557
*613741	GLYCOGEN PHOSPHORYLASE, LIVER; PYGL	14q22.	14:50905216-509	5836
*616065	PILR-ALPHA-ASSOCIATED NEURAL PROTEIN; I	12p13.	12:6689704-6700	196500
*616256	C-TYPE LECTIN DOMAIN FAMILY 4, MEMBER (	19p13.	19:7728956-7732	339390
*616594	ARF GTPase-ACTIVATING PROTEIN WITH SH3 I	1p36.1	1:23428562-2348	55616
*617084	TRANSMEMBRANE PROTEIN 59; TMEM59	1p32.3	1:54026680-5405	9528

*103720	ALCOHOL DEHYDROGENASE 1B, CLASS I, BET		4q23	4:99304970-9932	125
*608759	CYTOGLOBIN; CYGB		17q25.	17:76527355-765	114757
#167800	PANCREATITIS, HEREDITARY; PCTT	PANCREATITIS, (	1p36.2		
*601728	PHOSPHATASE AND TENSIN HOMOLOG; PTEN		10q23.	10:87863624-879	5728
*103730	ALCOHOL DEHYDROGENASE 1C, GAMMA POL		4q23	4:99336496-9935	126
*601532	CASPASE 6, APOPTOSIS-RELATED CYSTEINE PI		4q25	4:109688627-109	839
#239850	CANTU SYNDROME		12p12.		
%277730	WERNICKE-KORSAKOFF SYNDROME				
*601487	PEROXISOME PROLIFERATOR-ACTIVATED RE(	PPARG1, INCLUD	3p25.2	3:12287367-1243	5468
+170998	PEROXISOME PROLIFERATOR-ACTIVATED RE(	HYPERAPOBETA	22q13.	22:46150525-462	5465
*164160	LEPTIN; LEP		7q32.1	7:128241200-128	3952
*604517	PEROXISOME PROLIFERATOR-ACTIVATED RE(		4p15.2	4:23792020-2447	10891
#609016	LONG-CHAIN 3-HYDROXYACYL-CoA DEHYDR(		2p23.3		
#103780	ALCOHOL DEPENDENCE	ALCOHOL DEPEN	4p12, 4		
#188890	TOBACCO ADDICTION, SUSCEPTIBILITY TO		5p15.3		
*126450	DOPAMINE RECEPTOR D2; DRD2		11q23.	11:113409604-11	1813
*600890	HYDROXYACYL-CoA DEHYDROGENASE/3-KET		2p23.3	2:26190634-2624	3030
*601860	17-BETA-HYDROXYSTEROID DEHYDROGENAS		5q23.1	5:119452496-119	3295
#231680	MULTIPLE ACYL-CoA DEHYDROGENASE DEFI(	GLUTARIC ACID	4q32.1		
#214100	PEROXISOME BIOGENESIS DISORDER 1A (ZELI	PEROXISOME BI(	7q21.2		

This search returned 6,302 entries which exceeded the maximum of 200 so the results were truncated
You can register for MIMmatch (https://omim.org/mimmatch/) to raise this limit to 1,000
You can register for downloads (https://omim.org/downloads) and/or the API (https://omim.org/api) for more search/download options
