# Supplementary material for: Action Mechanism Underlying Improvement Effect of Fuzi Lizhong Decoction on Nonalcoholic Fatty Liver Disease: A Study Based on Network Pharmacology and Molecular Docking
Source: Evid Based Complement Alternat Med. 2022 Jan 20;2022:1670014. doi: 10.1155/2022/1670014 (PMC8794673; doi:10.1155/2022/1670014)
Supplement: Supplementary Materials — Supplement Table 1: detailed information about the compound-target network. Supplement Table 2: information of 2,608 NAFLD-related targets. Supplement Table 3: information of 61 intersection targets between FLD and NAFLD. Supplement Table 4: detailed information about the protein-protein network. Supplement Table 5: detailed information about functional analysis. [file 1670014.f1.zip › 1670014.f1/Supplement Table 3 Information of 61 intersection targets between FLD and NAFLD.pdf]

Intersection targets
MAPK8
BCHE
TREM1
NR1I3
MMP13
F2
ADH1B
PLAU
TTR
SULT2A1
PPARG
PLG
ESRRG
SHBG
NR1H4
CFD
CASP3
LCN2
FABP4
ALB
GSTP1
GC
MAPK14
PIK3CG
IGF1R
CES1
HSPA8
NQO1
CTSD
GBA
SPARC
GSR
TGFBR2
CA2
PPARD
NR1H2
EGFR
AKR1B1
CTSS
CASP7
AR
MAPK10
HSP90AA1
PTPN1
CYP19A1
FAP
HSD11B1
RORA
MAPK1
ESR1
NR3C2
ADAM17
CTSB
PRDX5
B3GAT1
RXRA
TGFBR1
APOA2
HSD17B11
DPP4
SELP
