# Supplementary material for: Action Mechanism Underlying Improvement Effect of Fuzi Lizhong Decoction on Nonalcoholic Fatty Liver Disease: A Study Based on Network Pharmacology and Molecular Docking
Source: Evid Based Complement Alternat Med. 2022 Jan 20;2022:1670014. doi: 10.1155/2022/1670014 (PMC8794673; doi:10.1155/2022/1670014)
Supplement: Supplementary Materials — Supplement Table 1: detailed information about the compound-target network. Supplement Table 2: information of 2,608 NAFLD-related targets. Supplement Table 3: information of 61 intersection targets between FLD and NAFLD. Supplement Table 4: detailed information about the protein-protein network. Supplement Table 5: detailed information about functional analysis. [file 1670014.f1.zip › 1670014.f1/Supplement Table 4 Detailed information about the protein-protein network.pdf]

#node1	node2	combined_score
ADAM17	MAPK1	0.654
ADAM17	MAPK14	0.672
ADAM17	MMP13	0.463
ADAM17	EGFR	0.846
ADAM17	ALB	0.63
ADAM17	F2	0.588
ADAM17	CASP3	0.408
ADAM17	MAPK8	0.441
ADH1B	GSTP1	0.743
AKR1B1	GSR	0.615
AKR1B1	EGFR	0.426
AKR1B1	CASP3	0.4
AKR1B1	MAPK8	0.434
AKR1B1	PTPN1	0.439
AKR1B1	NQO1	0.448
AKR1B1	ALB	0.576
AKR1B1	GSTP1	0.646
ALB	MAPK1	0.687
ALB	GSR	0.672
ALB	SULT2A1	0.513
ALB	MAPK14	0.593
ALB	SPARC	0.95
ALB	CTSD	0.585
ALB	TTR	0.93
ALB	FABP4	0.456
ALB	MMP13	0.495
ALB	SELP	0.619
ALB	BCHE	0.757
ALB	IGF1R	0.502
ALB	EGFR	0.767
ALB	CA2	0.567
ALB	PPARG	0.588

ALB	TGFBR1	0.4
ALB	PTPN1	0.4
ALB	GSTP1	0.404
ALB	CASP7	0.42
ALB	CES1	0.492
ALB	GBA	0.495
ALB	CYP19A1	0.496
ALB	PLAU	0.502
ALB	NR3C2	0.506
ALB	MAPK10	0.512
ALB	NQO1	0.523
ALB	HSPA8	0.559
ALB	HSP90AA1	0.56
ALB	GC	0.57
ALB	CTSB	0.604
ALB	AR	0.622
ALB	DPP4	0.671
ALB	LCN2	0.677
ALB	MAPK8	0.686
ALB	ESR1	0.7
ALB	SHBG	0.738
ALB	CASP3	0.835
ALB	RXRA	0.917
ALB	CFD	0.929
ALB	NR1H4	0.953
ALB	F2	0.96
ALB	PLG	0.978
ALB	APOA2	0.981
APOA2	TTR	0.743
APOA2	F2	0.838
APOA2	PLG	0.405
APOA2	GC	0.536
APOA2	RXRA	0.905

AR	MAPK1	0.782
AR	SULT2A1	0.577
AR	MAPK14	0.945
AR	IGF1R	0.783
AR	EGFR	0.803
AR	CA2	0.5
AR	CASP3	0.746
AR	HSP90AA1	0.988
AR	NR3C2	0.945
AR	CASP7	0.456
AR	ESR1	0.587
AR	GSTP1	0.659
AR	SHBG	0.742
AR	CYP19A1	0.879
AR	HSPA8	0.936
AR	MAPK8	0.945
BCHE	TTR	0.549
BCHE	F2	0.701
CA2	GC	0.565
CASP3	MAPK1	0.814
CASP3	GSR	0.59
CASP3	MAPK14	0.989
CASP3	CTSD	0.556
CASP3	MMP13	0.454
CASP3	SELP	0.407
CASP3	IGF1R	0.583
CASP3	EGFR	0.751
CASP3	PPARG	0.578
CASP3	F2	0.451
CASP3	PLG	0.497
CASP3	LCN2	0.433
CASP3	CYP19A1	0.436
CASP3	HSPA8	0.531

CASP3	NQO1	0.566
CASP3	CTSB	0.631
CASP3	ESR1	0.665
CASP3	HSP90AA1	0.676
CASP3	MAPK10	0.733
CASP3	MAPK8	0.901
CASP3	CASP7	0.935
CASP7	MAPK1	0.652
CASP7	MAPK14	0.548
CASP7	EGFR	0.475
CASP7	HSP90AA1	0.407
CASP7	MAPK8	0.689
CES1	NR1I3	0.425
CES1	NR1H4	0.606
CFD	SPARC	0.925
CFD	FABP4	0.69
CFD	PPARG	0.597
CFD	PLG	0.907
CTSB	CTSD	0.97
CTSB	EGFR	0.43
CTSB	PLG	0.58
CTSB	PPARD	0.421
CTSB	GBA	0.682
CTSB	HSP90AA1	0.421
CTSB	MAPK8	0.4
CTSB	PLAU	0.721
CTSB	CTSS	0.868
CTSD	PLAU	0.488
CTSD	GBA	0.503
CTSD	HSPA8	0.523
CTSD	EGFR	0.526
CTSD	PLG	0.554
CTSD	LCN2	0.923

CTSD	ESR1	0.959
CTSD	CTSS	0.959
CYP19A1	SULT2A1	0.669
CYP19A1	IGF1R	0.562
CYP19A1	EGFR	0.648
CYP19A1	PPARG	0.5
CYP19A1	HSD11B1	0.51
CYP19A1	SHBG	0.796
CYP19A1	ESR1	0.962
DPP4	FAP	0.436
DPP4	PPARG	0.51
EGFR	MAPK1	0.965
EGFR	SULT2A1	0.42
EGFR	MAPK14	0.81
EGFR	MMP13	0.601
EGFR	IGF1R	0.648
EGFR	F2	0.413
EGFR	TGFBR2	0.528
EGFR	PIK3CG	0.53
EGFR	PLG	0.567
EGFR	TGFBR1	0.588
EGFR	MAPK8	0.593
EGFR	GSTP1	0.634
EGFR	PPARG	0.787
EGFR	ESR1	0.943
EGFR	PLAU	0.951
EGFR	HSPA8	0.959
EGFR	PTPN1	0.988
EGFR	HSP90AA1	0.989
ESR1	MAPK1	0.971
ESR1	SULT2A1	0.521
ESR1	MAPK14	0.962
ESR1	IGF1R	0.989

ESR1	PLG	0.451
ESR1	NQO1	0.4
ESR1	HSP90AA1	0.979
ESR1	MAPK10	0.852
ESR1	PTPN1	0.484
ESR1	PLAU	0.451
ESR1	SHBG	0.683
ESR1	MAPK8	0.908
ESR1	GSTP1	0.562
ESR1	NR1H4	0.501
ESR1	HSPA8	0.558
F2	MAPK1	0.931
F2	TTR	0.667
F2	MMP13	0.465
F2	SELP	0.832
F2	NR1H4	0.464
F2	PLG	0.473
F2	GC	0.616
F2	PLAU	0.952
FABP4	LCN2	0.451
FABP4	PPARD	0.456
FABP4	RXRA	0.945
FABP4	PPARG	0.991
GBA	HSP90AA1	0.493
GC	GSR	0.646
GC	TTR	0.88
GC	PLG	0.571
GC	NR1H4	0.697
GSR	MAPK1	0.47
GSR	MAPK8	0.432
GSR	HSP90AA1	0.475
GSR	NQO1	0.717
GSR	PRDX5	0.82

GSR	GSTP1	0.82
GSTP1	MAPK1	0.485
GSTP1	NQO1	0.723
GSTP1	MAPK8	0.858
HSD11B1	SULT2A1	0.402
HSD11B1	PPARG	0.945
HSD11B1	NR3C2	0.721
HSD11B1	RXRA	0.905
HSD17B11	SULT2A1	0.417
HSP90AA1	MAPK1	0.979
HSP90AA1	MAPK14	0.589
HSP90AA1	IGF1R	0.67
HSP90AA1	PPARD	0.518
HSP90AA1	NQO1	0.478
HSP90AA1	NR1I3	0.52
HSP90AA1	TGFBR2	0.567
HSP90AA1	TGFBR1	0.612
HSP90AA1	MAPK8	0.834
HSP90AA1	NR3C2	0.967
HSP90AA1	HSPA8	0.999
HSPA8	MAPK1	0.425
HSPA8	NR3C2	0.919
HSPA8	MAPK10	0.641
HSPA8	MAPK8	0.767
IGF1R	MAPK1	0.934
IGF1R	MAPK14	0.504
IGF1R	TTR	0.592
IGF1R	PIK3CG	0.573
IGF1R	MAPK8	0.633
IGF1R	PTPN1	0.999
LCN2	PPARG	0.56
MAPK1	NQO1	0.4
MAPK1	PLAU	0.427

MAPK1	PLG	0.464
MAPK1	PIK3CG	0.496
MAPK1	PPARG	0.672
MAPK1	MMP13	0.688
MAPK1	PTPN1	0.76
MAPK1	MAPK14	0.889
MAPK1	TTR	0.915
MAPK1	RXRA	0.941
MAPK1	MAPK8	0.947
MAPK10	MAPK14	0.91
MAPK10	MAPK8	0.903
MAPK14	PIK3CG	0.414
MAPK14	PLG	0.418
MAPK14	PPARG	0.588
MAPK14	MMP13	0.603
MAPK14	TGFBR1	0.631
MAPK14	PTPN1	0.652
MAPK14	MAPK8	0.943
MAPK8	NR1H2	0.412
MAPK8	MMP13	0.728
MAPK8	PPARG	0.82
MAPK8	PLG	0.487
MAPK8	NQO1	0.515
MAPK8	PTPN1	0.705
MAPK8	PLAU	0.632
MAPK8	TGFBR1	0.543
MAPK8	NR1H4	0.44
MAPK8	RXRA	0.923
MMP13	SPARC	0.444
MMP13	PLAU	0.454
MMP13	PLG	0.949
NR1H2	RXRA	0.985
NR1H4	SULT2A1	0.634

NR1H4	RXRA	0.992
NR1I3	SULT2A1	0.726
NR1I3	RXRA	0.773
PLAU	PLG	0.951
PLG	SPARC	0.954
PLG	TTR	0.554
PLG	SELP	0.684
PLG	SHBG	0.42
PPARD	RXRA	0.955
PPARG	PTPN1	0.46
PPARG	RXRA	0.997
RXRA	SULT2A1	0.921
SHBG	SULT2A1	0.921
TGFBR1	TGFBR2	0.995
