# Supplementary material for: Action Mechanism Underlying Improvement Effect of Fuzi Lizhong Decoction on Nonalcoholic Fatty Liver Disease: A Study Based on Network Pharmacology and Molecular Docking
Source: Evid Based Complement Alternat Med. 2022 Jan 20;2022:1670014. doi: 10.1155/2022/1670014 (PMC8794673; doi:10.1155/2022/1670014)
Supplement: Supplementary Materials — Supplement Table 1: detailed information about the compound-target network. Supplement Table 2: information of 2,608 NAFLD-related targets. Supplement Table 3: information of 61 intersection targets between FLD and NAFLD. Supplement Table 4: detailed information about the protein-protein network. Supplement Table 5: detailed information about functional analysis. [file 1670014.f1.zip › 1670014.f1/Supplement Table 5 Detailed information about functional analysis.pdf]

Information of Biological process			
Pathway	Enricihment	P-value	Count
GO:0071407:cellular response to organic cyclic compound	0.032520325	5.91732E-15	16
GO:0008202:steroid metabolic process	0.042424242	9.71462E-15	14
GO:0048545:response to steroid hormone	0.042168675	1.05569E-14	14
GO:0031667:response to nutrient levels	0.030612245	1.12737E-13	15
GO:0009991:response to extracellular stimulus	0.029126214	2.31182E-13	15
GO:0006367:transcription initiation from RNA polymerase II promoter	0.058823529	2.60556E-13	11
GO:0045055:regulated exocytosis	0.021794872	4.88667E-13	17
GO:0002444:myeloid leukocyte mediated immunity	0.027173913	6.26557E-13	15
GO:0071396:cellular response to lipid	0.026548673	8.74461E-13	15
GO:0002446:neutrophil mediated immunity	0.028	2.74785E-12	14
GO:0006352:DNA-templated transcription, initiation	0.044176707	5.92396E-12	11
GO:0002274:myeloid leukocyte activation	0.022727273	7.97283E-12	15
GO:0032870:cellular response to hormone stimulus	0.022556391	8.86979E-12	15
GO:0030522:intracellular receptor signaling pathway	0.039711191	1.86948E-11	11
GO:0042060:wound healing	0.023850085	2.32515E-11	14
GO:0032963:collagen metabolic process	0.073394495	9.71377E-11	8
GO:0043299:leukocyte degranulation	0.024208566	1.10015E-10	13
GO:0010817:regulation of hormone levels	0.024163569	1.12559E-10	13
GO:0002275:myeloid cell activation involved in immune response	0.023636364	1.47551E-10	13
GO:0043627:response to estrogen	0.098591549	1.88397E-10	7

Information of Cellular component			
Pathway	Erichment	Pvalue	Count
GO:0031983:vesicle lumen	0.038235294	3.63803E-13	13
GO:0034774:secretory granule lumen	0.037267081	4.34675E-12	12
GO:1904813:ficolin-1-rich granule lumen	0.072580645	6.6862E-12	9
GO:0060205:cytoplasmic vesicle lumen	0.03539823	7.92298E-12	12
GO:0005775:vacuolar lumen	0.052023121	1.34419E-10	9
GO:0101002:ficolin-1-rich granule	0.048648649	2.44312E-10	9
GO:0043202:lysosomal lumen	0.072916667	1.61899E-09	7
GO:0072562:blood microparticle	0.047945205	3.02757E-08	7

GO:0000323:lytic vacuole	0.015602837	3.03115E-07	11
GO:0005764:lysosome	0.015602837	3.03115E-07	11
GO:0045121:membrane raft	0.024615385	5.03441E-07	8
GO:0098857:membrane microdomain	0.024615385	5.03441E-07	8
GO:0044437:vacuolar part	0.016977929	5.18034E-07	10
GO:0098589:membrane region	0.023668639	6.76365E-07	8
GO:0005773:vacuole	0.013767209	1.03746E-06	11
GO:0031091:platelet alpha granule	0.054945055	1.63393E-06	5
GO:0031093:platelet alpha granule lumen	0.059701493	1.38406E-05	4
GO:0043235:receptor complex	0.014814815	2.10842E-05	8
GO:0005925:focal adhesion	0.016706444	3.3739E-05	7
GO:0005924:cell-substrate adherens junction	0.016627078	3.47737E-05	7

Information of Molecular Function

Pathway	Erichment	Pvalue	Count
GO:0004879:nuclear receptor activity	0.211538462	1.08121E-19	11
GO:0098531:transcription factor activity, direct ligand regulated sequence	0.211538462	1.08121E-19	11
GO:0008289:lipid binding	0.024173028	2.39568E-15	19
GO:0005496:steroid binding	0.1	1.57454E-14	10
GO:0004175:endopeptidase activity	0.029411765	9.87877E-12	13
GO:0033293:monocarboxylic acid binding	0.0875	4.43265E-10	7
GO:0070011:peptidase activity, acting on L-amino acid peptides	0.021103896	5.8798E-10	13
GO:0008233:peptidase activity	0.020440252	8.66006E-10	13
GO:0003707:steroid hormone receptor activity	0.192307692	2.57432E-09	5
GO:0005504:fatty acid binding	0.128205128	2.20515E-08	5
GO:0031406:carboxylic acid binding	0.036363636	2.53927E-08	8
GO:0043177:organic acid binding	0.034482759	3.83176E-08	8
GO:0004707:MAP kinase activity	0.2	9.44089E-08	4
GO:0001223:transcription coactivator binding	0.153846154	2.88493E-07	4
GO:0030235:nitric-oxide synthase regulator activity	0.333333333	8.07195E-07	3
GO:0008134:transcription factor binding	0.016025641	8.74154E-07	10
GO:0016209:antioxidant activity	0.057471264	1.30687E-06	5
GO:0004252:serine-type endopeptidase activity	0.035294118	1.87909E-06	6

GO:0051427:hormone receptor binding	0.034682081	2.07983E-06	6
GO:0042562:hormone binding	0.050505051	2.47986E-06	5

Information of KEGG

Pathway	Erichment	P-value	Count
hsa05200:Pathways in cancer	0.032986111	8.15617E-18	19
hsa04659:Th17 cell differentiation	0.080357143	2.64146E-12	9
hsa04657:IL-17 signaling pathway	0.084210526	3.18178E-11	8
hsa04068:foxo signaling pathway	0.058394161	6.09699E-10	8
hsa04210:Apoptosis	0.052980132	1.32278E-09	8
hsa01522:Endocrine resistance	0.072916667	1.61899E-09	7
hsa04010:MAPK signaling pathway	0.03125	1.70088E-09	10
hsa04933:AGE-RAGE signaling pathway in diabetic complications	0.065420561	3.47698E-09	7
hsa04140:Autophagy - animal	0.045454545	4.27613E-07	6
hsa03320:PPAR signaling pathway	0.058139535	1.23381E-06	5
hsa04976:Bile secretion	0.053333333	2.16654E-05	4
hsa04918:Thyroid hormone synthesis	0.037974684	0.000682115	3
hsa00980:Metabolism of xenobiotics by cytochrome P450	0.052631579	2.28321E-05	4
hsa04932:Non-alcoholic fatty liver disease	0.029585799	3.34152E-05	5
hsa04668:TNF signaling pathway	0.052173913	1.89083E-07	6
hsa04920:Adipocytokine signaling pathway	0.041666667	0.000520012	3
